# Lewis Acid‐Catalyzed Strain‐Enabled (3+3) Annulation of Bicyclo[1.1.0]Butanes With Indolylalcohols: Synthesis of Indole‐Fused Aza‐Bicyclo[3.1.1]Heptanes

**DOI:** 10.1002/advs.78354

**Published:** 2026-10-18

**Authors:** Mainak Dey, Tuhin Subhra Das Mahapatra, Pragya Sharma, Manoj V. Mane, Chinmoy Kumar Hazra

**Affiliations:** ^1^ Department of Chemistry Indian Institute of Technology New Delhi India; ^2^ Centre For Nano and Material Sciences Jain (Deemed‐to‐be University) Bangalore Karnataka India

**Keywords:** aza‐bicyclo[1.1.0]heptanes, indolyalcohol, isostere, lewis acid catalysis

## Abstract

Strain release has long inspired the discovery of unique reactivity, enabling diverse synthetically valuable transformations. Bicyclo[1.1.0]butanes (BCBs), due to their high ring strain, have emerged as versatile building blocks for constructing complex bicyclic architectures. Herein, we report a Lewis acid‐catalyzed formal (3 + 3) annulation of BCBs with indolyl alcohols, providing rapid access to indole‐fused aza‐bicyclo[3.1.1]heptanes. The reaction proceeds under mild conditions, tolerates a broad range of functional groups, and delivers rigid, sp^3^‐rich scaffolds efficiently. Mechanistic studies, including control experiments and computational analysis, indicate a stepwise pathway in which Lewis acid activation of indolyl alcohols generates reactive intermediates, while strain‐release of BCBs drives C–C bond formation, followed by C–N bond formation via ring expansion to furnish the fused Aza‐bicyclic products.

## Graphical abstract

Bicyclo[1.1.0]butanes undergo a Lewis acid‐catalyzed formal (3 + 3) annulation with indolyl alcohols to furnish indole‐fused aza‐bicyclo[3.1.1]heptanes. Strain‐release‐driven C‐C bond formation, followed by C‐N bond formation via ring expansion, enables the rapid assembly of rigid, sp^3^‐rich bicyclic architectures under mild reaction conditions.

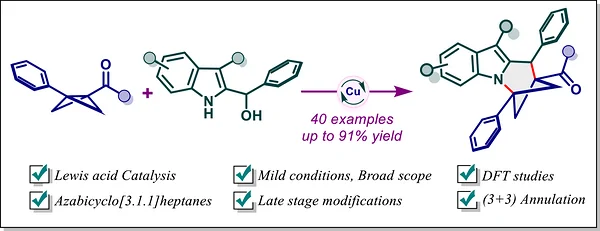

## 1 Introduction

Arene rings are prevalent structural motifs in bioactive molecules; however, they often impart undesirable pharmaceutical properties, including limited aqueous solubility and metabolic instability [1]. Consequently, the identification of suitable bioisosteres to replace aromatic systems has emerged as a central objective in medicinal chemistry. In this context, the strategic incorporation of three‐dimensional (3D), C(sp^3^)‐enriched frameworks in place of planar aromatic rings has gained significant attention. Such structural modifications can markedly improve pharmacokinetic and physicochemical properties, including molecular stability, solubility, lipophilicity, and the specificity of protein–ligand interactions, while retaining or enhancing biological activity [2, 3]. This design philosophy aligns with the concept of ‘'escaping from flatland'’ [4, 5, 6], which emphasizes the transition toward structurally complex and conformationally rich molecular architectures [7, 8, 9, 10, 11, 12, 13, 14, 15, 16, 17, 18, 19].

Among the various structural classes explored as arene bioisosteres, saturated bicyclic scaffolds, particularly bicyclo[n.1.1]alkanes such as bicyclo[1.1.1]pentanes [20, 21, 22, 23], bicyclo[2.1.1]hexanes, and bicyclo[3.1.1]heptanes (BCHeps) have demonstrated considerable promise due to their rigid, highly strained frameworks. More recently, the incorporation of heteroatoms into these bicyclic systems has enabled the development of bioisosteres that mimic heteroaromatic motifs, thereby expanding their applicability in pharmaceutical and materials chemistry.

Within this domain, bicyclo[1.1.0]butanes (BCBs) have emerged as uniquely reactive intermediates owing to their exceptionally high ring strain (∼64 kcal·mol^−^^1^) [24, 25, 26, 27, 28, 29, 30]. This strain renders the central carbon–carbon σ bond highly susceptible to diverse transformations, including electrophilic and nucleophilic additions, radical processes, and insertion reactions with carbenes and π‐systems. As a result, BCBs serve as versatile building blocks for the construction of C(sp^3^)‐rich, rigidified molecular frameworks. It is worth noting that BCBs exhibit similar behavior as donor‐acceptor (D‐A)cyclopropanes and can be considered a special case of D‐A cyclopropanes but with higher strain energy [31, 32, 33, 34]. Notably, energy transfer‐catalyzed [2π + 2σ] cycloadditions of BCBs with alkenes, as reported by Glorius [35] and Brown [36], have enabled efficient access to bicyclo[2.1.1]hexane derivatives [35, 36, 37, 38]. In addition, bicyclo[3.1.1]heptane scaffolds can be synthesized through (3 + 3) annulation strategies employing photocatalytic [39, 40, 41, 42], transition‐metal‐catalyzed [43, 44, 45], or Lewis acid‐mediated approaches [46, 47, 48, 49, 50, 51, 52, 53, 54, 55, 56, 57, 58, 59]. Despite these advances, the development of structurally well‐defined heteroaromatic bioisosteres remains comparatively underexplored, largely due to the challenges associated with incorporating heteroatoms while maintaining structural integrity and desired geometry (Figure 1A). Nitrogen‐fused heterocycles, particularly pyrido[1,2‐a]indoles, constitute an important class of compounds widely encountered in natural products and bioactive molecules. These frameworks are present in alkaloids [60] such as canthin‐6‐ones [61], dibenzopyrrocolines [62], and homofascaplysins B and C [63], (‐)‐goniomitine [64] which exhibit diverse biological activities, including anticancer [65, 66], anti‐inflammatory [67, 68], and immunomodulatory effects [69]. Beyond their medicinal relevance, pyrido[1,2‐a]indoles have also found applications in fluorescent materials, molecular sensing [70, 71], and organic electronics [72] (Figure 1B).

**FIGURE 1:**
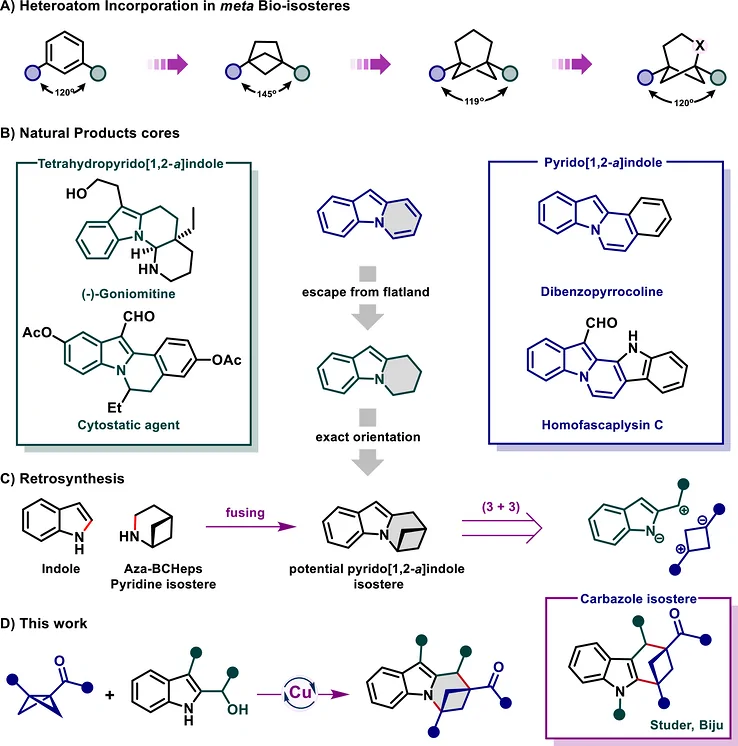
(A) The development of *meta*‐substituted arene bioisosteres based on bridged bicyclo[n.1.1] frameworks. (B) Importance of Pyrido[1,2‐*a*]indole core. (C) Retrosynthetic analysis. D) Annulation of BCBs with indoles (previous works) and Lewis acid catalyzed (3 + 3) annulation of indolyl alcohols with BCB (Present Work).

Synthetic access to these frameworks has been extensively explored, with numerous transition‐metal‐catalyzed annulation strategies developed using π‐extending reagents such as diazoenals, *o*‐haloaryl alkynes, and internal alkynes via C–H or N–H activation processes [73, 74, 75, 76, 77, 78]. Despite significant progress in BCB‐mediated transformations the synthesis of indole‐fused bicyclo[n.1.1]alkane frameworks remain largely underdeveloped. The construction of such sterically congested and conformationally rigid architectures presents considerable synthetic challenges, yet these structures hold substantial promise for enhancing molecular diversity and drug‐like properties [79]. In this regard, indolylalcohols represent versatile three‐atom synthons capable of participating in annulation reactions through the generation of reactive intermediates under mild Brønsted acid or solvent‐mediated conditions. This reactivity enables the efficient assembly of complex polycyclic indole frameworks with high atom economy. While (3 + 2) cycloaddition strategies involving indoles and BCBs have been successfully employed to access diverse 3D indole scaffolds [80, 81], formal (3 + 3) cycloadditions between indolylalcohols and BCBs remain rare. A notable advancement was reported in 2025 by Studer and co‐workers, who described a Brønsted acid‐catalyzed formal (3 + 3) cycloaddition of 3‐indolylalcohols with BCBs, affording tetrahydro‐1,3‐methanocarbazole derivatives with high stereocontrol [82]. This transformation proceeds via an initial (3 + 2) cycloaddition of a 3‐vinylindole intermediate, followed by a 1,2‐migration to generate the fused scaffold (Figure 1D). Complementarily, Biju and co‐workers developed a hexafluoroisopropanol (HFIP)‐mediated activation strategy that enables simultaneous activation of both coupling partners under mild, metal‐free conditions, leading to indole‐fused bicyclo[3.1.1]heptane derivatives [83]. Despite some reports on (3+3) annulation involving 3‐indolylalcohols and BCBs, 2‐indolylalcohols remain largely unexplored, despite their potential as versatile partners in direct (3+3) annulation (Figure 1C). Although these advances highlight the effectiveness of strain‐release strategies in accessing C(sp^3^)‐rich indole architectures, the development of general and modular approaches to heterobicyclo[n.1.1]alkanes remain limited. Addressing this challenge would enable the synthesis of rigid isosteric mimetics and facilitate the development of three‐dimensional heterocyclic scaffolds with significant implications in medicinal and materials chemistry. Herein, we report a synthetic method that allows straightforward and efficient access to novel, Indole‐fused Aza‐bicyclo[3.1.1]heptane's scaffolds (Figure 1D).

## 2 Results and Discussion

The investigation of the present studies was initiated using methyl 3‐phenylbicyclo[1.1.0]butane‐1‐carboxylate (**1a**) and (3‐methyl‐1*H*‐indol‐2‐yl)(phenyl)methanol (**2a**) as model substrates for the formal (3 + 3)‐cycloaddition reaction. The treatment of methyl 3‐phenylbicyclo[1.1.0]butane‐1‐carboxylate (**1a**) with (3‐methyl‐1*H*‐indol‐2‐yl)(phenyl)methanol (**2a**) in the presence of 10.0 mol% Sc(OTf)_3_ as catalyst in CH_2_Cl_2_ at 25°C, anticipated (3 + 3) annulated product **3a** in 20% yield (Table 1, entry 1). To thoroughly investigate the influence of temperature on our reactions, we elevated the temperature to 40°C, resulting in 42% increase in yield (entry 2). Additionally, we explored various Lewis acids (as detailed in Table 1, entries 3–7), but unfortunately, these alternatives yielded only moderate to low yields. This decline can be attributed to the moisture‐sensitive characteristics of the Lewis acid catalysts, which led to quenching and diminished effectiveness. However, by switching to a more moisture‐tolerant catalyst, Cu(OTf)_2_, we achieved a significant increase in product yield to 58% (entry 8). This demonstrates the critical importance of catalyst selection for optimizing yield in our reactions. We conducted a detailed investigation of a range of solvents, including acetonitrile, 1,4‐dioxane, HFIP, and chloroform (entries 9–12). Among these, chloroform proved to be the most effective, affording product **3a** in 68% yield. Encouragingly, increasing the reaction temperature to 60°C further improved the reaction efficiency, delivering **3a** in an excellent 83% isolated yield (entry 13). This striking outcome underscores the effectiveness of temperature in enhancing product yield. Additionally, reducing solvent concentration does not impact the optimized yield (entry 14). However, lowering the catalyst loading to 5.0 mol% yields 62% of **3a** (entry 15). The excess bicyclo[1.1.0]butane was converted into the corresponding cyclobutene, and no dimerization of the BCB was observed [84].

**TABLE 1:** Optimization.

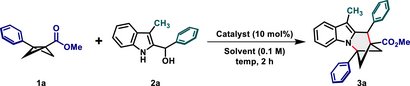				
Entry	Catalyst	Temp.	Solvent	Yield of 3a [%]^b^
1	Sc(OTf)_3_	25°C	CH_2_Cl_2_	20
2	Sc(OTf)_3_	40 ^o^C	CH_2_Cl_2_	42
3	Bi(OTf)_2_	40 ^o^C	CH_2_Cl_2_	33
4	Yb(OTf)_2_	40 ^o^C	CH_2_Cl_2_	30
5	Ni(OTf)_2_	40 ^o^C	CH_2_Cl_2_	32
6	Co(OTf)_2_	40 ^o^C	CH_2_Cl_2_	55
7	Zn(OTf)_2_	40 ^o^C	CH_2_Cl_2_	54
8	Cu(OTf)_2_	40 ^o^C	CH_2_Cl_2_	58
9	Cu(OTf)_2_	40 ^o^C	CH_3_CN	64
10	Cu(OTf)_2_	40 ^o^C	1,4‐Dioxane	56
11	Cu(OTf)_2_	40 ^o^C	HFIP	<5
12	Cu(OTf)_2_	40 ^o^C	CHCl_3_	68
13	Cu(OTf)_2_	60 ^o^C	CHCl_3_	86 (83)
14^c^	Cu(OTf)_2_	60 ^o^C	CHCl_3_	84
15^d^	Cu(OTf)_2_	60 ^o^C	CHCl_3_	62

^a^ Reaction conditions: 1a (0.15 mmol), 2a (0.1 mmol), catalyst (0.01 mmol), solvent (0.1 M, 1 mL) for 2 h.

^b^ The ^1^H NMR yield of the crude products was determined using 1,1,2‐trichloroethylene as the internal standard and the isolated yields are given in parenthesis.

^c^ Solvent concentration 0.05 M.

^d^ 5.0 mol% Catalyst was used.

With optimized conditions, the general applicability of the current transformation was systematically investigated utilizing a diverse array of 2‐indolylalcohols (Figure 2). Initially, the focus was directed toward variations at the benzylic aryl moiety. The unsubstituted phenyl analogue served as a representative example, yielding **3a** with 83% yield. The structure of **3a** was confirmed by X‐ray crystallography [85]. 2‐Indolylalcohols with substituents at the *para* and *meta* positions of the aryl group proved to be viable under the optimized conditions, affording the tetracyclic products in good yields (**3b**‐**3d**). Moreover, 2‐indolylalcohols bearing an electron‐donating group, specifically ‐OMe at the *para* and *meta* position (**3e**‐**3f**), underwent smooth transformation to the corresponding products, yielding 55% and 71% respectively. Additionally, halogen substituents exhibited favourable tolerance, the desired products **3g‐3k** moderate to good yields (43%–77%). Moreover, 2‐thienyl substituted 2‐indolylalcohol also delivered the anticipated product **3l** in 70% yield. We conducted an in‐depth exploration of substitution on the indole core, revealing promising results. Variations at the C3 position of indole with a phenyl group produced **3m** with a 66% yield. The reaction with a 4‐methoxy group, carried out under standard conditions, also yielded **3n** at 54%. The methodology demonstrated exceptional flexibility, as halogen substituents, such as fluorine and chlorine, at the *para* position of the aryl group were also well tolerated, yielding **3o** (60%) and **3p** (67%), respectively. Additionally, incorporating a naphthyl group at the C3 position was effective, delivering **3q** in 57% yield. Furthermore, C5‐substituted indoles bearing electron‐donating group, such as OMe, afforded **3s** in 70% yield, whereas electron‐withdrawing groups, such as fluoro and bromo, afforded **3t** and **3u** in 51% and 53% yields, respectively, underscoring the broad functional‐group tolerance of our innovative approach.

**FIGURE 2:**
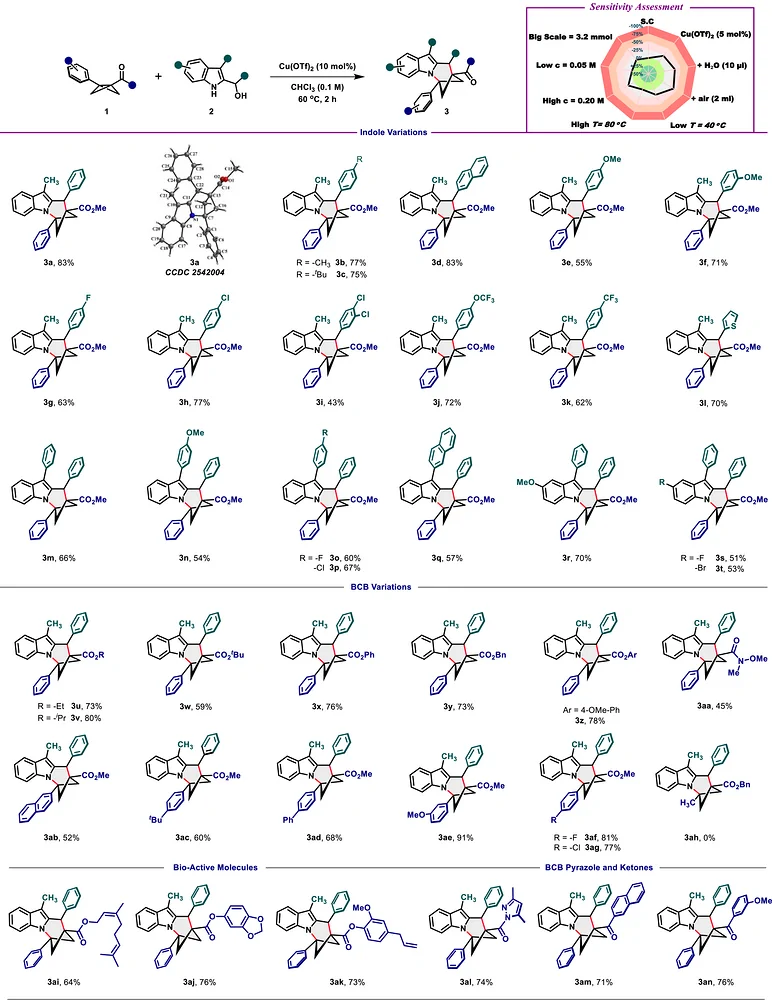
Substrate Scopes. Reaction conditions: **1** (0.3 mmol), **2** (0.2 mmol), catalyst Cu(OTf)_2_ (0.02 mmol), CHCl_3_ (0.1 M, 2 mL) at 60°C, 2 h. Yields of the isolated products are given.

Having addressed the scope of the indolyl component, we varied the BCB reaction partner using **2a** as the indole moiety. The investigation focused on the effectiveness of bicyclic compounds (BCBs) for the synthesis of indole‐fused bicyclo[3.1.1]heptanes **3**, as illustrated in Figure 2. BCB esters, including Et, *i*Pr, *^t^* Bu, Ph, Bn, and 4‐methoxy‐phenyl esters, delivered the products **3u‐3z** in good to high yields (59%–80%). The Weinreb‐BCB amide provided **3aa** in 45% yield. Subsequently, the substituents on the aryl moiety were modified. BCBs possessing electronically diverse substituents located at the 3‐ and 4‐positions of the aryl ring effectively participated in the (3 + 3) annulation, leading to the formation of tetracyclic indoles in commendable yields (**3ab‐3ag**). Both alkyl (‐*^t^* Bu) and aryl substituents at the 4‐position of the aryl ring (**3ab‐3ad**) were found to be compatible, yielding favourable results (52%–68%). Furthermore, electron‐donating groups, such as 3‐OMe, were successfully incorporated under standard reaction conditions, yielding the desired product **3ae** with a 91% yield. Additionally, halogen substituents at the 4‐position of the aryl rings demonstrated robust reactivity, yielding products **3af** (81%) and **3ag** (77%).3‐Methyl BCB benzyl ester give the desired product **3ah** in trace amount.

The methodology has been significantly enhanced to enable the synthesis of BCB‐based biologically active compounds, showcasing its robust and innovative nature. Notably, BCBs derived from natural products such as Nerol, Sesamol, and Eugenol have successfully undergone the (3 + 3) annulation process, yielding the desired products (**3ai‐3ak**) with commendable yields (64%–76%). This adaptability underscores the methodology's versatility and its potential for broader applications. It is also noteworthy that our protocol extends beyond ester‐substituted BCBs; both disubstituted pyrazole and keto BCBs have demonstrated reactivity with compound **2a**, yielding tetracyclic products (**3al‐3an**) in satisfactory yields. This capability underscores the methodology's value in advancing the synthesis of complex biologically active compounds. Lastly, reaction‐condition‐based sensitivity assessment (See Detailed analysis in Supporting Information) revealed that high oxygen levels and water lowered the yield by up to 50% (Figure 2, radar graph) [86].

The scalability and practicality of the reaction were demonstrated by performing the formal (3 + 3)‐cycloaddition at 3.2 mmol scale, yielding 1.02 g (78%) of **3a** (Figure 3A). With a larger quantity of compound **3a** available, we proceeded to conduct synthetic transformations as outlined in Figure 3B. The reaction of **3a** with phenylmagnesium bromide (PhMgBr) yielded carbinol **4** with 84% yield. Treatment of compound **3a** with LiAlH_4_ in THF led to the reduction of the ester group to an alcohol, resulting in the formation of compound **5** in 92% yield. The transformation of alcohol **5** into benzyl bromide **6** was achieved with 64% yield, facilitated by *N*‐bromosuccinimide (NBS) in the presence of triphenylphosphine (PPh_3_). Furthermore, the 5‐bromo derivative **3u** successfully underwent a smooth Suzuki reaction, resulting in the formation of phenyl‐substituted indole **7** with a yield of 95%. The Weinreb amide **3ab** underwent a reductive transformation to form aldehyde **8** using lithium aluminum hydride (LiAlH_4_) at 0°C. In addition, compound **3ab** was converted into yne‐one **9** with a yield of 62% through the reaction of phenylacetylene with *n‐*butyllithium (*n‐*BuLi).

**FIGURE 3:**
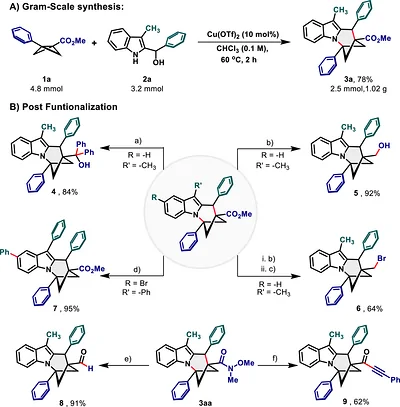
A) Gram‐scale synthesis of 3a. B) Post functionalization.

### 2.1 Control Experiments

To elucidate the reaction pathway and to identify potential reaction intermediates, a series of control experiments was conducted. The reaction of BCB **1a** under standard conditions, in the absence of indole, yielded cyclobutene with a remarkable 96% yield. However, subsequent treatment of cyclobutene with indolyl alcohol under the same standard conditions did not yield compound **3a**. These findings effectively eliminate the possibility that cyclobutene acts as a reaction intermediate, as detailed in Figure 4A. The unsubstituted indolyl alcohol at the 3‐position does not yield the desired product, highlighting the significance of protecting the 3‐position on indolyl alcohol (Figure 4B).

**FIGURE 4:**
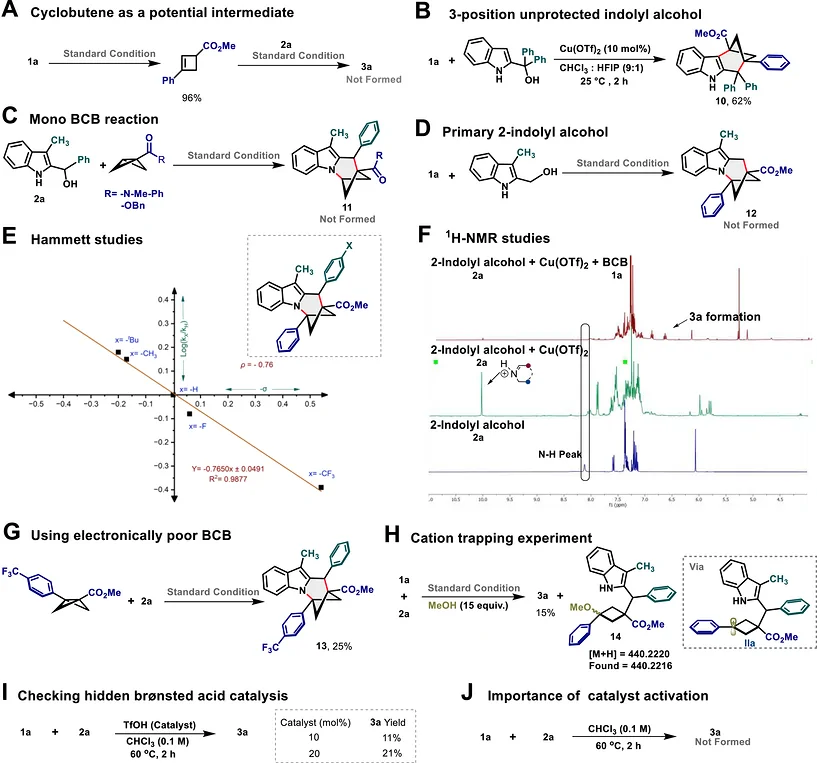
Mechanistic Experiments.

Unsubstituted BCB benzyl esters and amides (as depicted in Figure 4C) did not yield the expected products **11** or the corresponding cyclobutene. This outcome is likely due to the reduced nucleophilicity of the strained BCB *σ*‐bond resulting from the absence of substituents. These findings underline the importance of structural modifications in enhancing reaction efficacy and highlight the intricacies of carbocation behaviour in cycloaddition reactions. When indolylalcohol (primary alcohol) was subjected to the optimized conditions, the desired product **12** was not formed, likely due to the instability of the cationic indolenium species in this case (Figure 4D). This observation highlights the requirement of substitution at the indolyl moiety for stabilizing the cationic indolenium species [87], generated after activation of indolyl alcohols with Cu(OTf)_2_.

To investigate the substituent effects in the 1,3‐dipolar cycloaddition reaction, a Hammett analysis [88] was conducted by assessing the reaction rates of individual substrates bearing various *para*‐substituents on the aryl moiety of 2‐indolylalcohol **2a** (Figure 4E). Kinetic studies indicated that 2‐indolylalcohol containing electron‐donating alkyl groups, such as 4‐*tert*‐butyl (4‐*^t^* Bu) or 4‐methyl (4‐Me), exhibited faster reaction rates compared to those with electron‐withdrawing substituents, such as 4‐trifluoromethyl (4‐CF_3_) or 4‐fluoro (4‐F). A notable negative linear correlation was observed when log(*k_X_/k_H_*) was plotted against the σ parameter, which suggests a linear free‐energy relationship (*ρ* = ‐0.76). This analysis likely indicates the formation of a partial positive charge at the transition state (**Ia**) during the cycloaddition. To further investigate the existence of intermediate **Ia**, we conducted a nuclear magnetic resonance (NMR) study, as illustrated in Figure 4F.

Initially, we acquired the ^1^H NMR spectrum of the pure compound **2a**. Upon adding the catalyst, copper triflate, to the same NMR tube containing **2a**, we recorded the NMR spectrum, which showed a proton shift from 8.1 ppm to 10.0 ppm. This shift provides evidence for the formation of intermediate **Ia**. Further, upon introducing BCB to the reaction mixture, the peak at 10.0 ppm disappeared, and the NMR spectrum showed peaks corresponding to the desired product **3a**. This sequence of observations confirms that the reaction proceeds via intermediate **Ia**, which was also observed in HRMS (see in SI.). To critically evaluate the role of a carbocation intermediate in a nonconcerted cycloaddition, the electron‐deficient 3‐(*p*‐trifluoromethyl)benzyl‐carbamate (BCB) was subjected to reaction under standard conditions. However, the corresponding product **13** was obtained in lower yield (Figure 4G), which is likely due to the instability of the potential carbocation intermediate at C‐3 of the BCB.

To convincingly establish the presence of a carbocation intermediate in a non‐concerted cycloaddition, we performed a cation‐trapping experiment with methanol, as shown in Figure 4H. Performing the reaction in the presence of an excess of methanol (15 equiv.) led to the detection of the methanol‐trapped product **14** by HRMS. This finding strongly suggests the formation of the cyclobutyl cation **IIa**, reinforcing its potential role as an intermediate in our reaction pathway. To thoroughly evaluate the role of the catalyst, we performed the reaction with triflic acid (Brønsted acid) that may form in‐situ in the reaction medium. The notable low yield of the desired product underscores that the reaction is predominantly driven by the Lewis acid catalyst, copper triflate, rather than by the Brønsted acid. This finding reinforces the importance of selecting the appropriate catalyst for optimal outcomes (See Figure 4I). Next, we performed a reaction in the absence of a catalyst and, as anticipated, no product formation was observed. This outcome highlights the significant role of Lewis acid activation in the interaction between BCB and indolyl alcohol (Figure 4J).

### 2.2 Mechanism

Based on control experiments and previous studies, we propose the mechanism illustrated in Figure 5A. The reaction is initiated by the simultaneous activation of **1a** and **2a** with Cu(OTf)_2_, resulting in the formation of the dearomatized indolenium ion **Ia** from **2a** and the ester enolate **Ib** from **1a**. The BCB intermediate **Ib** subsequently attacks the methylene carbon of intermediate **Ia**, leading to the generation of the carbocationic aromatic intermediate **IIa**. Intermediate **IIa** has the potential to undergo cyclization at the C2 position of the indole ring, resulting in the formation of spiroindolenine **IIIab**. Furthermore, intermediate **IIIab** may undergo a ring‐enlarging 1,2‐alkyl shift to yield the desired product **3a** exclusively, with no evidence of **3a'** being observed. To investigate whether the spiro‐intermediate **IIIab** facilitates selective migration (either tertiary or secondary alkyl migration, as indicated by the blue or red arrows), we have performed quantum chemical calculations using density functional theory (DFT) (full details in the Supporting Information).**IIa** undergoes cyclization, giving the spiro‐intermediate **IIIab** (‐57.0 kcal/mol). The reaction will be complete upon enlargement of the spiro‐intermediate **IIIab**, which undergoes selective tertiary or secondary migration of the group to generate the desired products. The transition‐state barrier for **TS_1_** (transfer of a tertiary group) is 6.8 kcal/mol lower than that for **TS_2_** (transfer of a secondary group). Finally, the deprotonation gives the highly stable desired product **3a** over **3a'**.

**FIGURE 5:**
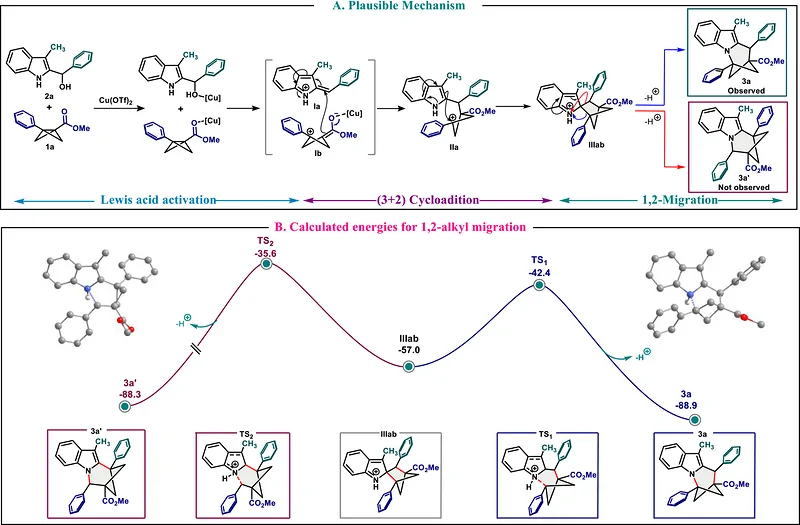
A) Plausible Mechanism, B) Energy (kcal/mol) calculation of 1,2‐alkyl migration. Free energy values are at the M06(SMD)/def2‐TZVP//PBE0‐BJD3/def2‐SVP level of theory using CHCl_3_ as solvent. Non‐essential hydrogen atoms have been omitted for the sake of clarity.

## 3 Conclusion

In conclusion, we disclose a Lewis acid‐catalyzedformal (3 + 3) annulation of bicyclo[1.1.0]butanes with indolyl alcohols that enables rapid access to indole‐fused aza‐bicyclo[3.1.1]heptanes. Enabled by rigorous optimization, this transformation proceeds under mild conditions with broad substrate generality, delivering sp^3^‐rich, conformationally constrained scaffolds. Mechanistic interrogation, supported by control experiments and DFT calculations, reveals a stepwise pathway involving Lewis acid activation, strain‐release‐driven C–C bond formation, and ring expansion. The method is compatible with late‐stage functionalization and supports diverse downstream derivatizations. By merging strain‐release reactivity with Lewis acid catalysis, this work establishes a versatile platform for the efficient construction and diversification of complex nitrogen‐containing bicyclic architectures.

## Author Contributions

**Mainak Dey**: conceptualization, investigation, methodology, validation, writing – original draft, writing – review and editing. **Tuhin Subhra Das Mahapatra**: methodology. **Pragya Sharma**: writing – review and editing, writing – original draft. **Manoj V. Mane**: software. **Chinmoy Kumar Hazra**: conceptualization, funding acquisition, validation, visualization, supervision, writing – review and editing.

## Conflicts of Interest

The authors declare no conflicts of interest.

## Supporting information




**Supporting File**: advs78354‐sup‐0001‐SuppMat.pdf.

## Data Availability

The data that supports the findings of this study are available in the supplementary material of this article.
