# Supplementary material for: Lewis Acid‐Catalyzed Strain‐Enabled (3+3) Annulation of Bicyclo[1.1.0]Butanes With Indolylalcohols: Synthesis of Indole‐Fused Aza‐Bicyclo[3.1.1]Heptanes
Source: Adv Sci (Weinh). 2026 Oct 18:e78354. Online ahead of print. doi: 10.1002/advs.78354 (PMC13676991; doi:10.1002/advs.78354)

Supporting Information

Lewis Acid-Catalyzed Strain-enabled (3+3) Annulation of Bicyclo[1.1.0]butanes with Indolylalcohols: Synthesis of Indole-fused Aza-bicyclo[3.1.1]heptanes

Mainak Dey,[a] Tuhin Subhra Das Mahapatra,[a]‡ Pragya Sharma,[a]‡ Manoj V. Mane,[b] and Chinmoy Kumar Hazra[a]*

a. Department of Chemistry
Indian Institute of Technology, Delhi
Hauz Khas, New Delhi, 110016, India. Phone Number +91 11 2654 8408
b. Centre for Nano and Material Sciences, Jain (Deemed-to-be University), Bangalore, Karnataka
‡ Both authors contributed equally

1	General Information	3-4
2	Synthesis and analytical data of Starting materials	5-33
	2.1 Synthesis of BCBs	5-18
	2.8 Synthesis of indolylalcohols Derivatives	18-33
3	Reaction Optimization	34-38
4	Reproducibility test	38-39
5	Synthesis and analytical data of Indole-fused Aza-bicyclo[3.1.1]heptanes derivatives	40-60
6	Scale-up synthesis	71
7	Synthetic applications	71-76
8	Mechanistic experiments	77-84
9	DFT studies	85-97
10	X-Ray diffraction analysis	98-99
11	References	100-101
12	NMR data of the compounds	101-211

1. General Information

All reagents and solvents were of pure analytical grade. All experiments were carried out in a round bottom flask equipped with a stirring bar. Chemicals were purchased from Sigma-Aldrich, TCI, Alfa Aesar and Sisco Research Laboratories (SRL) and used without further purification. Analytical thin layer chromatography (TLC) was carried out using 0.2 mm commercial silica gel plates (silica gel 60, F254, EMD Chemical). Visualization of the developed TLC plate was performed by irradiation with UV light. High-resolution mass spectra (HRMS) were recorded on a mass spectrometer using electrospray ionization-time of-flight (ESITOF) reflectron experiments. ^{1}H-NMR and ^{13}C-NMR were recorded on 400 MHz, and 500 MHz spectrometers, using $CDCl_3$ as a solvent the chemical shifts are reported as parts per million (ppm) referenced to residual protium or carbon of the solvents; $CDCl_3$ δ H (7.26 ppm). Coupling constants are reported in Hertz (Hz). Data for ^{1}H NMR spectra are reported as follows: chemical shift (ppm, referenced to protium; s = singlet, d = doublet, t = triplet, q = quartet, quin = quintet, sext = sextet, dd = doublet of doublets, td = triplet of doublets, ddd = doublet of doublet of doublets, m = multiplet, coupling constant (Hz), and integration).All bicyclobutanes (BCBs) were synthesized following the literature procedure. [1-4] The indolyl alcohols were synthesized by following the literature procedure. [5-7]

List of BCBs synthesized:

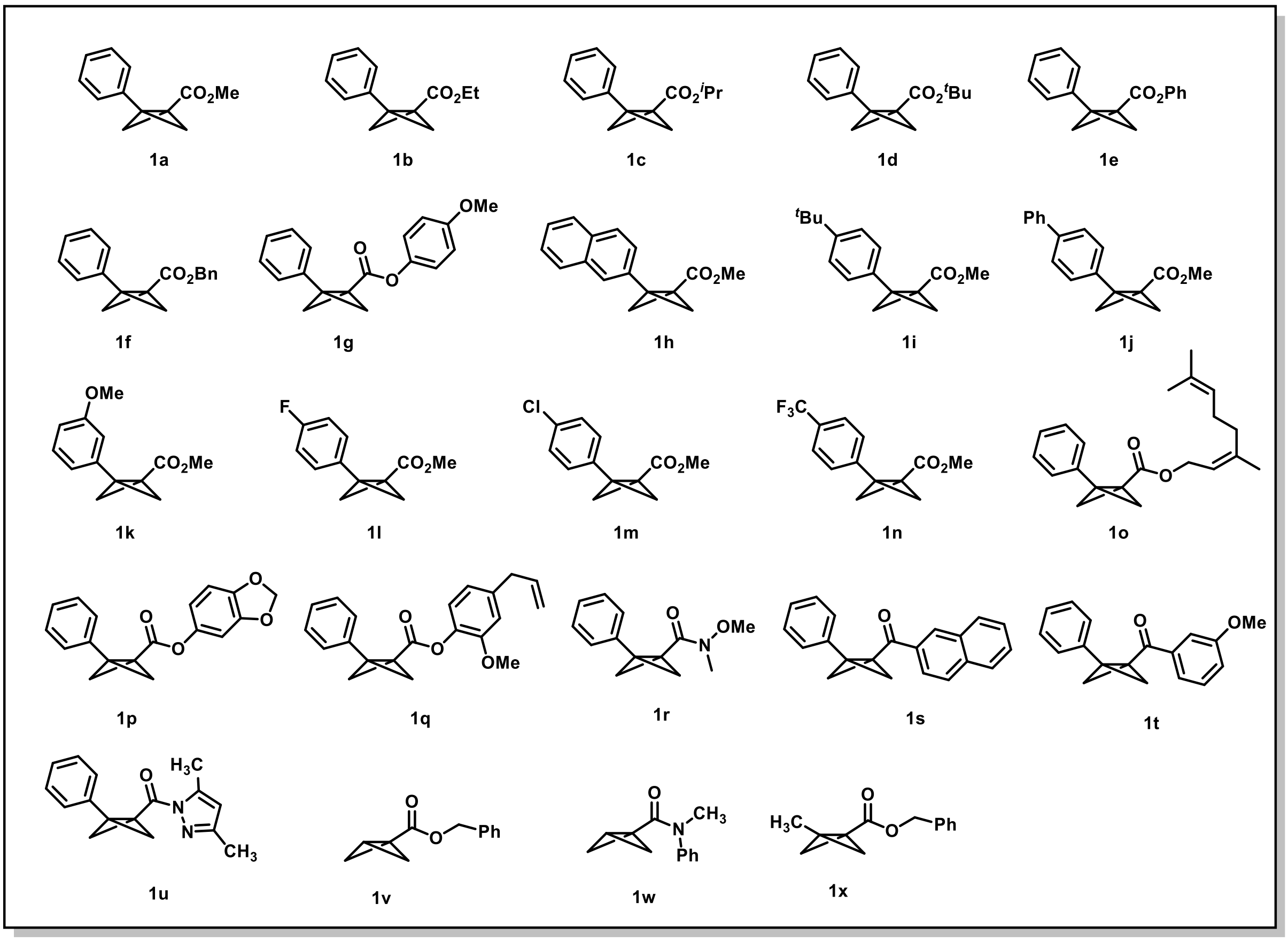

List of indolyl methanol derivatives synthesized:

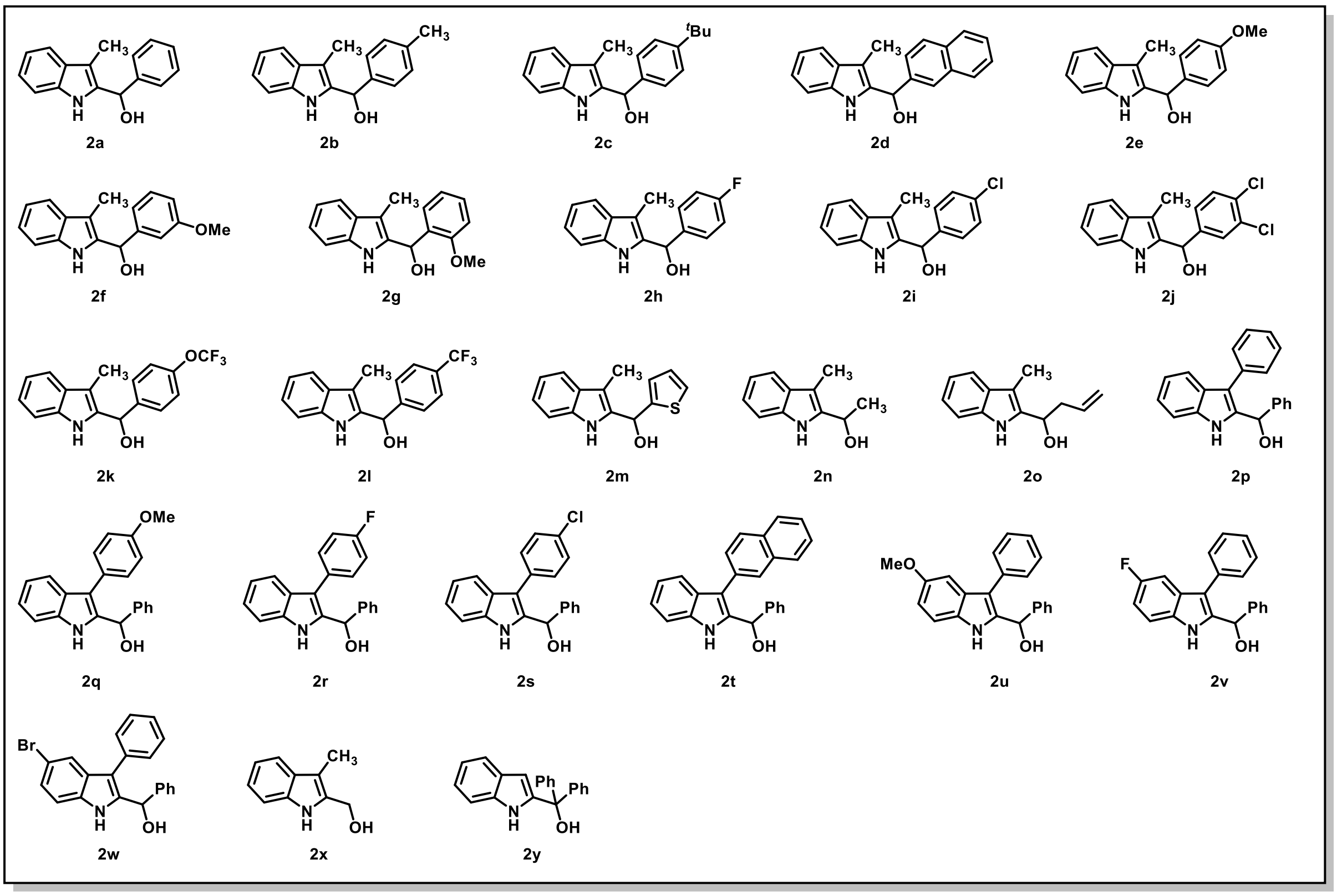

Failed Scopes:

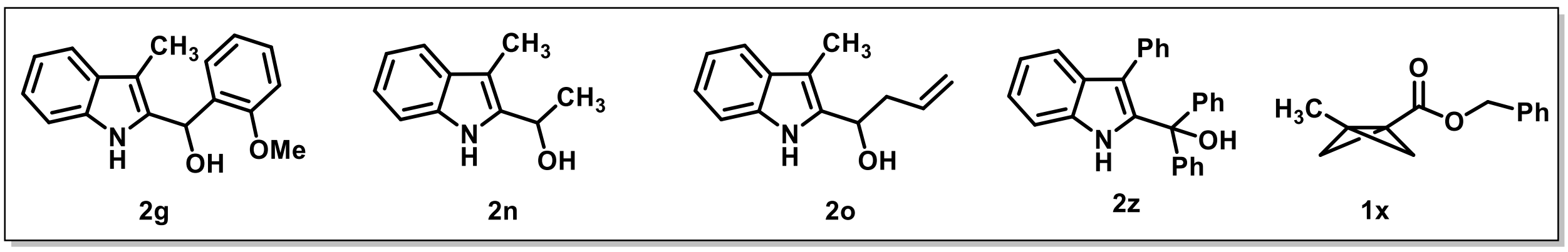

2. General procedure-1 (GP-1)

2.1. Synthesis of 3-arylbicyclo[1.1.0]butane-1-carboxylates:

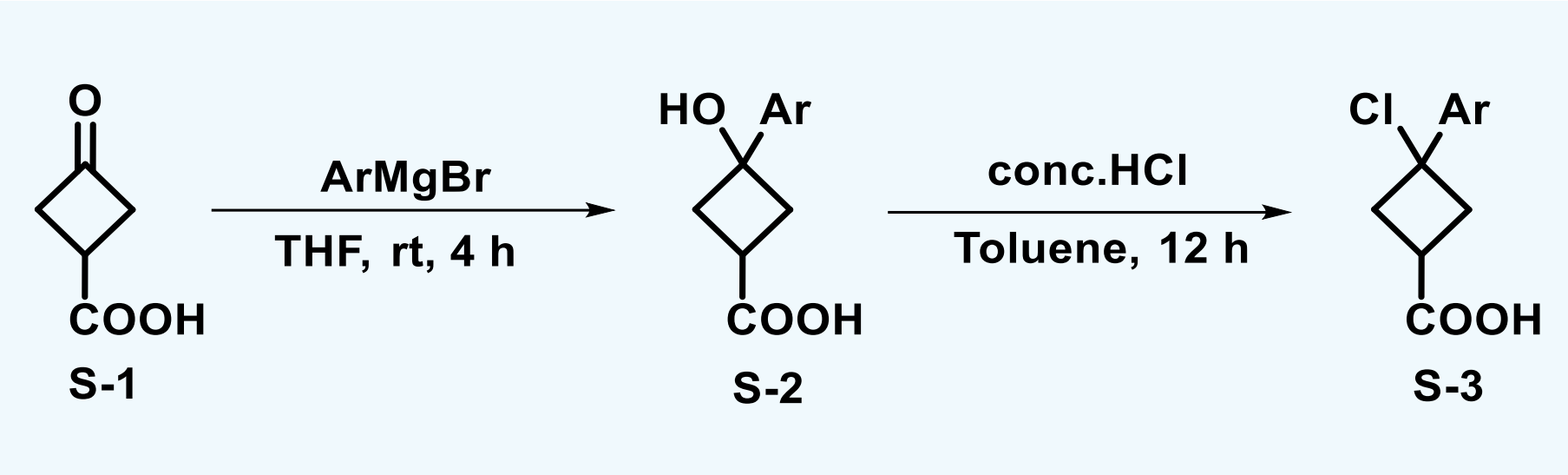

ArMgBr (3.0 M in THF, 106 mmol, 2.2 equiv.) was added dropwise to THF solution of **S-1** (5.50 g, 48 mmol, 1 equiv.) at 0 °C and stirred for 4 h under a nitrogen atmosphere. The reaction mixture was quenched with a saturated aqueous solution of NH_4Cl and extracted with ethyl acetate (3 × 30 mL). The combined organic layer was washed with brine (50 mL), dried over $MgSO_4$, and concentrated in a vacuum. A diastereomeric mixture (*syn*- and *anti*- isomers, 8 g, 42 mmol) was obtained and taken directly for the next step without further purification.

To a solution of **S-2** (8 g, 42 mmol, 1 equiv.) in toluene (100 mL), concentrated HCl (51 mL, 624 mmol, 15 equiv.) was added dropwise using a dropping funnel, and the reaction was stirred for 12 h at rt. The reaction mixture was diluted with 100 mL of brine solution, and the organic layer was extracted with EtOAc (3 × 100 mL). The combined organic phase was washed with water, $NaHCO_3$ (100 mL) and brine solution (100 mL), dried over $MgSO_4$, and concentrated in a vacuum. The residue (crude **S-3**) was taken into the next step without further purification.

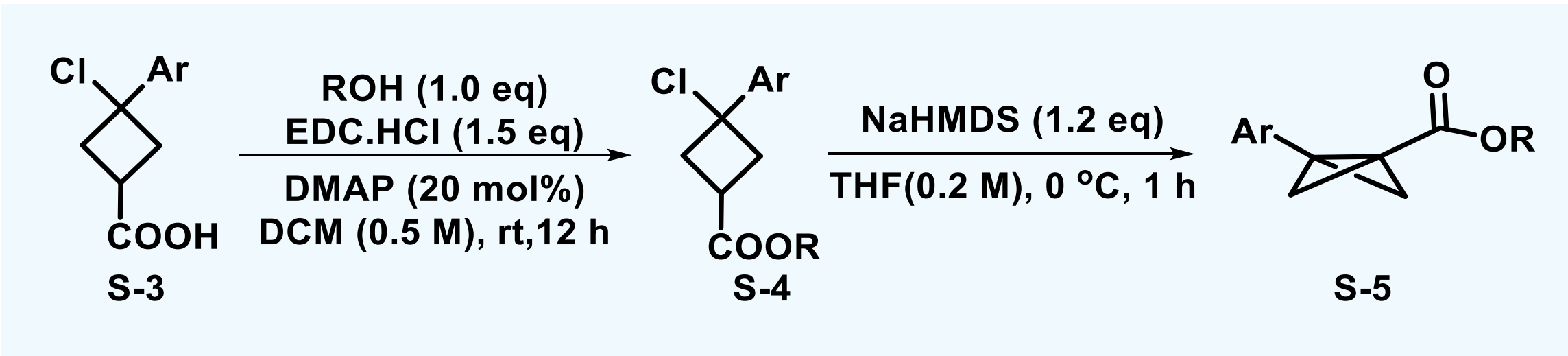

To the stirred solution of crude **S-3** acid (1.0 equiv., 2.0 mmol) in DCM (0.5 M, 4.0 mL), EDC-HCl (1.5 equiv., 3.00 mmol, 0.683 g) and DMAP (20 mol%, 0.400 mmol, 49.2 mg) were added sequentially. After stirring for 5 minutes, ROH (1.0 equiv., 2.0 mmol) was added and the reaction was allowed to stir at room temperature for 12 hours. Upon completion, the reaction was quenched with aqueous NH_4Cl solution and the aqueous layer was extracted with DCM (3 x 20 mL). The combined organic layers were dried over anhydrous Na_2SO_4, filtered, and concentrated by rotary evaporation. The crude residue **S-4** was used without further purification.

In the final step, an oven-dried 50 mL round bottom flask equipped with a stir bar was cooled under a vacuum, backfilled with N_2 (x 3), and capped with a septum. The crude **S-4** (1 equiv., assuming 2.0

mmol) and THF (0.2 M, 10 mL) were added under N_2 atmosphere. The reaction mixture was cooled to 0 °C and NaHMDS (2 M in THF, 1.2 mL, 1.2 equiv.) was added dropwise. The reaction mixture was allowed to stir for 1 h at the same temperature before quenching with 10 ml NH_4Cl solution. The aqueous layer was extracted with EtOAc (3 x 20 mL). The combined organic layers were dried over anhydrous Na_2SO_4, filtered, and concentrated by rotary evaporation. The residue was purified by FCC on silica gel (Hexane/ethyl acetate) to give 3-arylbicyclo[1.1.0]butane-1-carboxylate **1a-1q**.

Methyl-3-phenylbicyclo[1.1.0]butane-1-carboxylate (1a):

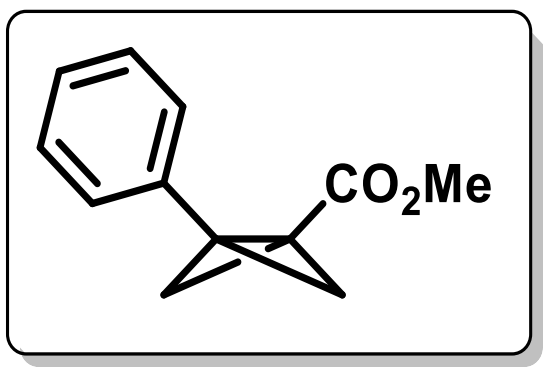

Following the general procedure (GP1), **1a** was isolated as a colourless solid (0.215 g, 57% yield over two steps). The physical data are matching with the reported value.

^{1}H NMR ((400 MHz, $CDCl_3$) δ (ppm)) δ 7.45 – 7.09 (m, 5H), 3.45 (s, 3H), 2.91 (t, J = 1.2 Hz, 2H), 1.58 (t, J = 1.1 Hz, 2H).

^{13}C {^{1}H} NMR ((101 MHz, $CDCl_3$) δ (ppm)) δ 170.10, 133.73, 128.56, 127.13, 126.02, 51.86, 35.91, 32.97, 23.36.

Ethyl 3-phenylbicyclo[1.1.0]butane-1-carboxylate (1b):

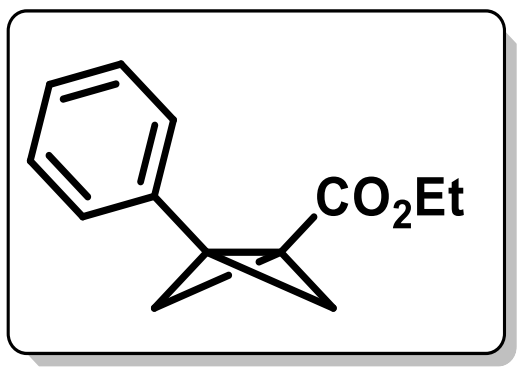

Following the general procedure (GP1), **1b** was isolated as a colorless solid (0.250 g, 62% yield over two steps). The physical data are matching with the reported value.

^{1}H NMR ((400 MHz, $CDCl_3$) δ (ppm)) δ 7.41 – 6.73 (m, 5H), 3.76 (q, J = 7.1 Hz, 2H), 2.85 – 2.75 (m, 2H), 1.45 – 1.36 (m, 2H), 0.81 – 0.73 (m, 3H).

^{13}C {^{1}H} NMR ((101 MHz, $CDCl_3$) δ (ppm)) δ 169.12, 133.68, 128.35, 126.88, 125.87, 60.19, 35.64, 32.38, 23.14, 14.36.

Isopropyl 3-phenylbicyclo[1.1.0]butane-1-carboxylate (1c):

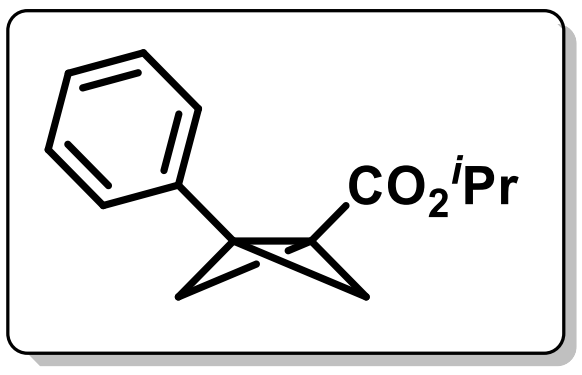

Following the general procedure (GP1), **1c** was isolated as a colorless solid (0.311 g, 72% yield over two steps). The physical data are matching with the reported value.

^{1}H NMR ((400 MHz, $CDCl_3$) δ (ppm)) δ 7.32 – 7.21 (m, 4H), 7.20 (ddt, J = 5.7, 4.2, 2.6 Hz, 1H), 4.77 (hept, J = 6.3 Hz, 1H), 2.93 (t, J = 1.2 Hz, 2H), 1.57 (t, J = 1.2 Hz, 2H), 0.92 (d, J = 6.3 Hz, 6H).

^{13}C {^{1}H} NMR ((101 MHz, $CDCl_3$) δ (ppm)) δ 169.25, 133.77, 128.39, 126.89, 125.90, 67.75, 35.78, 32.43, 23.20, 21.87.

***tert*-butyl 3-phenylbicyclo[1.1.0]butane-1-carboxylate (1d):**

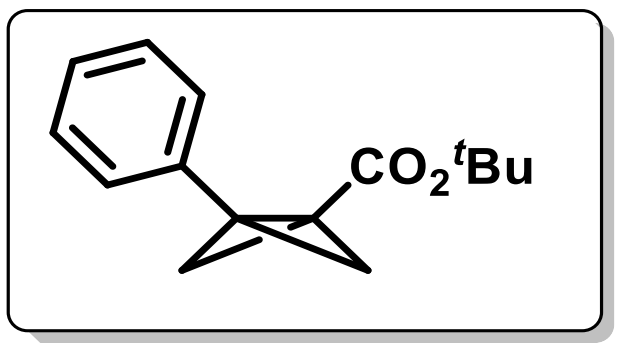

Following the general procedure (GP1), **1d** was isolated as a colourless solid (0.290 g, 63% yield over two steps). The physical data are matching with the reported value.

^{1}H NMR ((400 MHz, $CDCl_3$) δ (ppm)) δ 7.32 – 7.10 (m, 5H), 2.92 – 2.81 (m, 2H), 1.53 – 1.47 (m, 2H), 1.10 (s, 9H).

^{13}C {^{1}H} NMR ((101 MHz, $CDCl_3$) δ (ppm)) δ 168.84, 134.01, 128.33, 126.80, 125.77, 80.33, 35.76, 31.78, 27.94, 23.78.

Phenyl 3-phenylbicyclo[1.1.0]butane-1-carboxylate (1e):

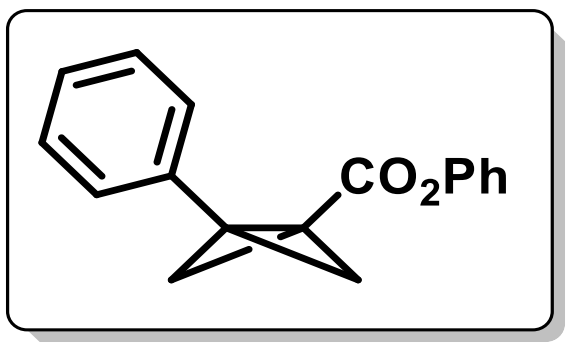

Following the general procedure (GP1), **1e** was isolated as a colorless solid (0.185 g, 37% yield over two steps). The physical data are matching with the reported value.

^{1}H NMR ((400 MHz, $CDCl_3$) δ (ppm)) δ 7.44 – 7.30 (m, 5H), 7.23 – 7.14 (m, 2H), 7.14 – 7.05 (m, 1H), 6.56 – 6.48 (m, 2H), 3.13 (t, J = 1.4 Hz, 2H), 1.78 (t, J = 1.3 Hz, 2H).

^{13}C {^{1}H} NMR ((101 MHz, $CDCl_3$) δ (ppm)) δ 168.70, 150.74, 133.22, 129.31, 128.71, 127.48, 126.12, 125.63, 121.79, 36.69, 34.70, 23.18.

Benzyl 3-phenylbicyclo[1.1.0]butane-1-carboxylate (1f):

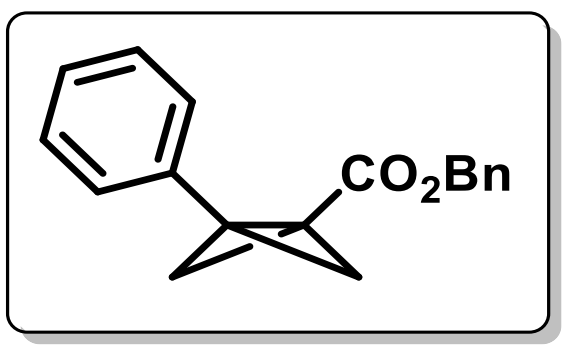

Following the general procedure (GP1), **1f** was isolated as a colorless solid (0.253 g, 48% yield over two steps). The physical data are matching with the reported value.

^{1}H NMR ((400 MHz, $CDCl_3$) δ (ppm)) δ 7.34 – 7.18 (m, 8H), 7.06 – 6.96 (m, 2H), 4.95 (d, J = 0.7 Hz, 2H), 2.99 (t, J = 1.2 Hz, 2H), 1.63 (t, J = 1.2 Hz, 2H).

^{13}C {^{1}H} NMR ((101 MHz, $CDCl_3$) δ (ppm)) δ 169.73, 136.28, 133.55, 128.63, 128.46, 127.93, 127.85, 127.20, 126.11, 66.26, 35.99, 33.50, 23.29.

4-Methoxyphenyl 3-phenylbicyclo[1.1.0]butane-1-carboxylate (1g):

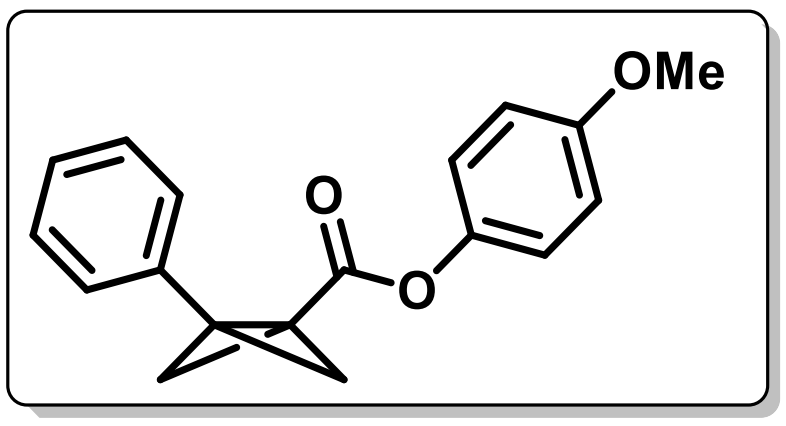

Following the general procedure (GP1), **1g** was isolated as a colorless solid (0.381 g, 68% yield over two steps). The physical data are matching with the reported value.

^{1}H NMR ((400 MHz, $CDCl_3$) δ (ppm)) δ 7.43 – 7.27 (m, 5H), 6.76 – 6.66 (m, 2H), 6.47 – 6.38 (m, 2H), 3.71 (s, 3H), 3.12 (t, J = 1.4 Hz, 2H), 1.76 (t, J = 1.3 Hz, 2H).

^{13}C {^{1}H} NMR ((101 MHz, $CDCl_3$) δ (ppm)) δ 169.09, 157.14, 144.22, 133.30, 128.68, 127.43, 126.12, 122.49, 116.10, 114.87, 114.37, 55.60, 36.66, 34.51, 23.14.

Methyl-3-(naphthalen-2-yl)bicyclo[1.1.0]butane-1-carboxylate (1h):

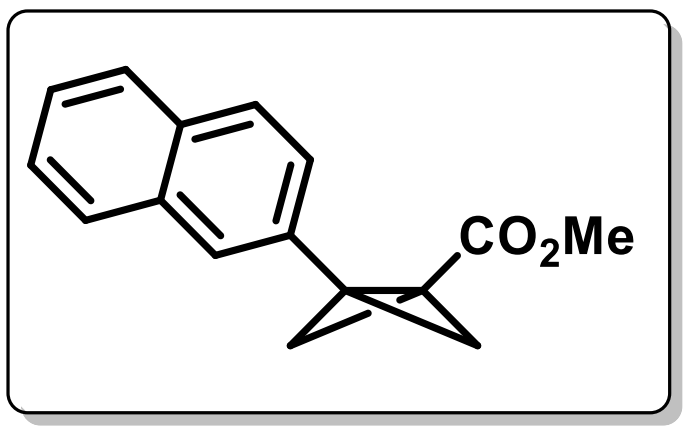

Following the general procedure (GP1), **1h** was isolated as a colorless solid (0.157 g, 33% yield over two steps). The physical data are matching with the reported value.

^{1}H NMR ((400 MHz, $CDCl_3$) δ (ppm)) δ 7.83 – 7.73 (m, 4H), 7.51 – 7.34 (m, 3H), 3.45 (s, 3H), 3.05 (t, J = 1.2 Hz, 2H), 1.68 (t, J = 1.2 Hz, 2H).

^{13}C {^{1}H} NMR ((101 MHz, $CDCl_3$) δ (ppm)) δ 170.13, 133.48, 132.58, 131.36, 128.29, 127.79, 127.65, 126.45, 125.84, 125.73, 123.32, 51.95, 36.07, 33.50, 23.61.

Methyl-3-(4-(*tert*-butyl)phenyl)bicyclo[1.1.0]butane-1-carboxylate (1i):

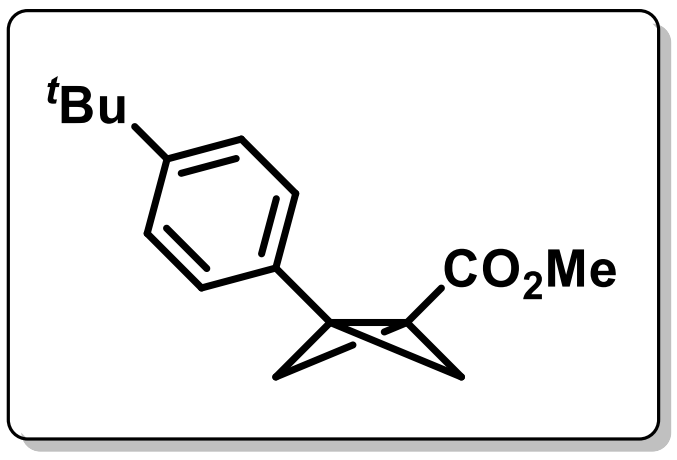

Following the general procedure (GP1), **1i** was isolated as a colorless solid (0.283 g, 58% yield over two steps). The physical data are matching with the reported value.

^{1}H NMR ((400 MHz, $CDCl_3$) δ (ppm)) δ 7.39 – 7.18 (m, 4H), 3.49 (s, 3H), 2.94 – 2.86 (m, 2H), 1.57 (t, J = 1.1 Hz, 2H), 1.36 – 1.20 (m, 9H).

^{13}C {^{1}H} NMR ((101 MHz, $CDCl_3$) δ (ppm)) δ 170.44, 150.10, 130.43, 125.83, 125.53, 51.86, 35.86, 34.58, 32.70, 31.36, 22.91.

Methyl 3-([1,1'-biphenyl]-4-yl)bicyclo[1.1.0]butane-1-carboxylate (1j):

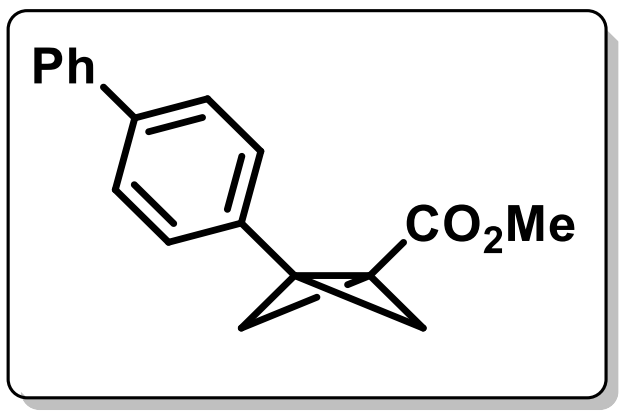

Following the general procedure (GP1), **1j** was isolated as a colorless solid (0.259 g, 49% yield over two steps). The physical data are matching with the reported value.

^{1}H NMR ((400 MHz, $CDCl_3$) δ (ppm)) δ 7.61 – 7.49 (m, 4H), 7.46 – 7.39 (m, 2H), 7.38 – 7.28 (m, 3H), 3.50 (s, 3H), 2.96 (t, J = 1.2 Hz, 2H), 1.64 (t, J = 1.2 Hz, 2H).

^{13}C {^{1}H} NMR ((101 MHz, $CDCl_3$) δ (ppm)) δ 170.20, 140.64, 139.89, 132.81, 128.90, 127.42, 127.27, 127.01, 126.48, 51.99, 36.02, 33.08, 23.65.

Methyl-3-(3-methoxyphenyl)bicyclo[1.1.0]butane-1-carboxylate (1k):

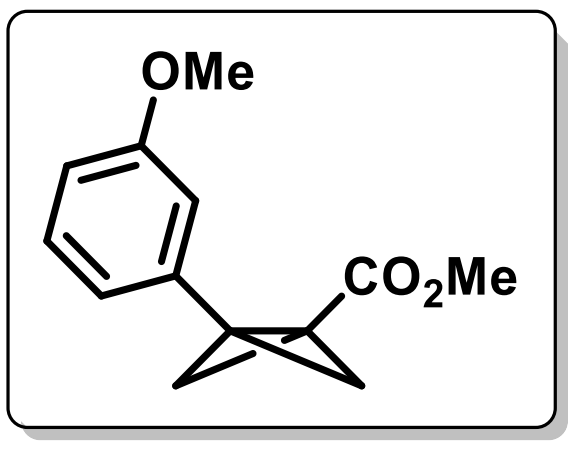

Following the general procedure (GP1), **1k** was isolated as a colorless solid (0.201 g, 46% yield over two steps). The physical data are matching with the reported value.

^{1}H NMR ((400 MHz, $CDCl_3$) δ (ppm)) δ 7.17 (t, J = 7.9 Hz, 1H), 6.90 – 6.70 (m, 3H), 3.74 (s, 3H), 3.46 (s, 3H), 2.87 (t, J = 1.2 Hz, 2H), 1.54 (t, J = 1.2 Hz, 2H).

^{13}C {^{1}H} NMR ((101 MHz, $CDCl_3$) δ (ppm)) δ 170.07, 159.77, 135.32, 129.57, 118.46, 112.59, 111.87, 55.20, 35.89, 32.94.

Methyl-3-(4-fluorophenyl)bicyclo[1.1.0]butane-1-carboxylate (1l):

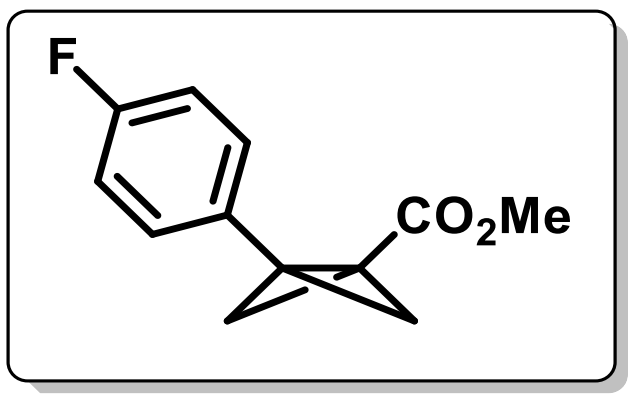

Following the general procedure (GP1), **1l** was isolated as a colorless solid (0.132 g, 32% yield over two steps). The physical data are matching with the reported value.

^{1}H NMR ((400 MHz, $CDCl_3$) δ (ppm)) δ 7.28 – 7.18 (m, 2H), 7.03 – 6.91 (m, 2H), 3.47 (s, 3H), 2.86 (t, J = 1.2 Hz, 2H), 1.57 (t, J = 1.2 Hz, 2H).

^{13}C {^{1}H} NMR ((101 MHz, $CDCl_3$) δ (ppm)) δ 170.03, 162.20 (d, J = 246.1 Hz), 129.52 (d, J = 3.4 Hz), 127.59 (d, J = 8.2 Hz), 115.60 (d, J = 21.7 Hz), 51.90, 35.98, 32.38, 22.93.

^{19}F NMR ((376 MHz, $CDCl_3$) δ (ppm)) δ -114.99.

Methyl-3-(4-chlorophenyl)bicyclo[1.1.0]butane-1-carboxylate (1m):

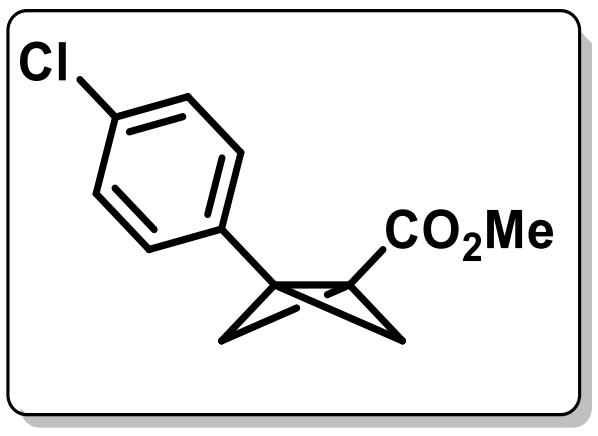

Following the general procedure (GP1), **1m** was isolated as a colorless solid (0.302 g, 68% yield over two steps). The physical data are matching with the reported value.

^{1}H NMR ((400 MHz, $CDCl_3$) δ (ppm)) δ 7.27 – 7.12 (m, 4H), 3.44 (s, 3H), 2.84 (t, J = 1.2 Hz, 2H), 1.55 (t, J = 1.3 Hz, 2H).

^{13}C {^{1}H} NMR ((100 MHz, $CDCl_3$) δ (ppm)) δ 169.76, 132.91, 132.48, 128.75, 127.28, 51.95, 35.95, 32.17, 23.61.

Methyl-3-(4-(trifluoromethyl)phenyl)bicyclo[1.1.0]butane-1-carboxylate (1n):

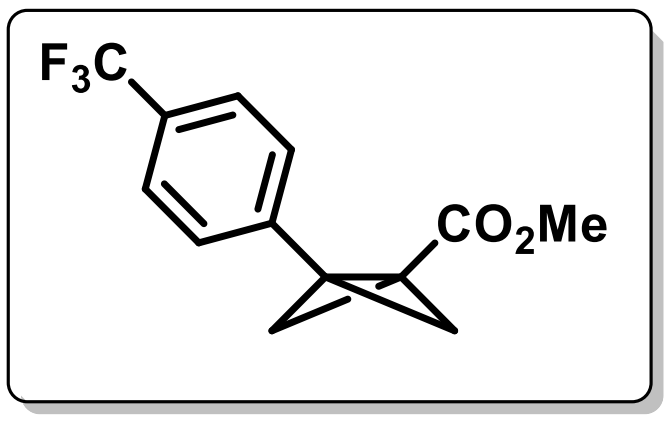

Following the general procedure (GP1), **1n** was isolated as a colorless solid (0.230 g, 45% yield over two steps). The physical data are matching with the reported value.

^{1}H NMR ((400 MHz, $CDCl_3$) δ (ppm)) δ 7.56 – 7.49 (m, 2H), 7.40 – 7.32 (m, 2H), 3.47 (d, *J* = 0.5 Hz, 3H), 2.93 (t, *J* = 1.2 Hz, 2H), 1.64 (t, *J* = 1.2 Hz, 2H).

^{13}C {^{1}H} NMR ((101 MHz, $CDCl_3$) δ (ppm)) 169.5, 139.2, 128.2 (q, *J* = 32 Hz), 126.52, 126.48, 125.3 (q, *J* = 4.0 Hz, 2C), 124.1 (q, *J* = 272 Hz), 51.1, 35.4, 30.8, 24.3.

^{19}F NMR ((376 MHz, $CDCl_3$) δ (ppm)) δ -62.38.

(*Z*)-3,7-dimethylocta-2,6-dien-1-yl 3-phenylbicyclo[1.1.0]butane-1-carboxylate (1o):

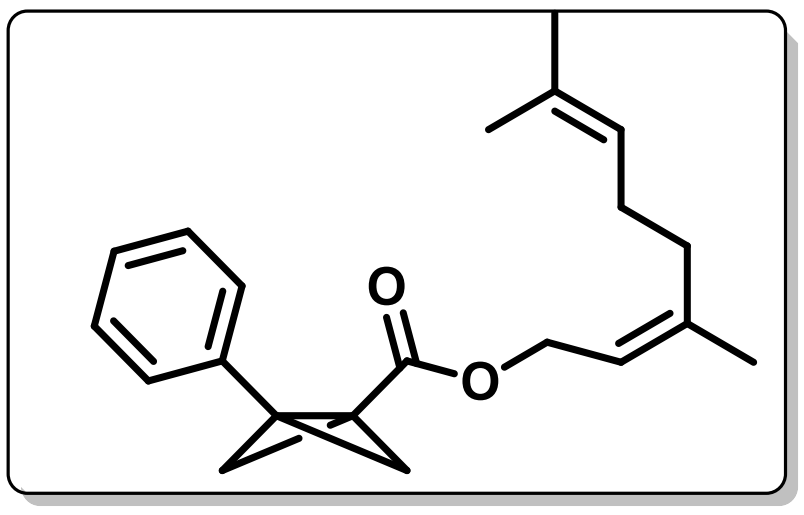

Following the general procedure (GP1), **1o** was isolated as a colorless solid (0.415 g, 67% yield over two steps). The physical data are matching with the reported value.

^{1}H NMR ((400 MHz, $CDCl_3$) δ (ppm)) δ 7.32 – 7.16 (m, 5H), 5.06 – 4.92 (m, 2H), 4.35 (dd, *J* = 7.2, 1.1 Hz, 2H), 2.93 (d, *J* = 1.2 Hz, 2H), 1.95 (s, 4H), 1.76 – 1.49 (m, 11H).

^{13}C {^{1}H} NMR ((101 MHz, $CDCl_3$) δ (ppm)) δ 169.84, 141.96, 133.68, 132.11, 128.47, 127.00, 126.05, 123.73, 119.69, 61.19, 35.93, 32.87, 32.10, 26.68, 25.76, 23.43, 23.20, 17.73.

HRMS (ESI) Calcd. for $C_{21}H_{26}O_2$ $[M+H]^+$, 311.2006, found: 311.2012.

Benzo[d][1,3]dioxol-5-yl-3-phenylbicyclo[1.1.0]butane-1-carboxylate (1p):

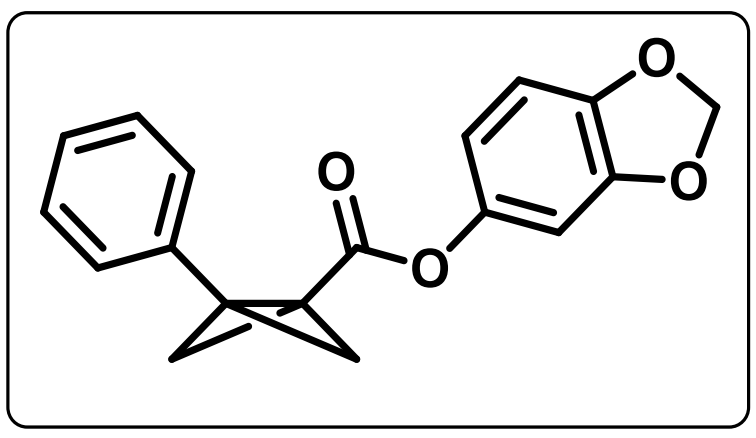

Following the general procedure (GP1), **1p** was isolated as a colorless solid (0.417 g, 71% yield over two steps).

^{1}H NMR((400 MHz, $CDCl_3$) δ (ppm)) δ 7.68 – 7.24 (m, 5H), 6.61 (d, *J* = 8.3 Hz, 1H), 6.03 (dd, *J* = 8.4, 2.3 Hz, 1H), 5.97 (d, *J* = 2.3 Hz, 1H), 5.86 (s, 2H), 3.13 (t, *J* = 1.3 Hz, 2H), 1.77 (t, *J* = 1.3 Hz, 2H).

^{13}C {^{1}H} NMR ((101 MHz, $CDCl_3$) δ (ppm)) δ 169.01, 147.84, 145.27, 145.04, 133.23, 128.80, 127.59, 126.11, 114.18, 107.90, 103.98, 101.68, 36.76, 34.74, 23.15.

HRMS (ESI) Calcd. for $C_{18}H_{14}O_4$ $[M+H]^+$, 295.0965, found: 295.0980.

4-Allyl-2-methoxyphenyl 3-phenylbicyclo[1.1.0]butane-1-carboxylate (1q):

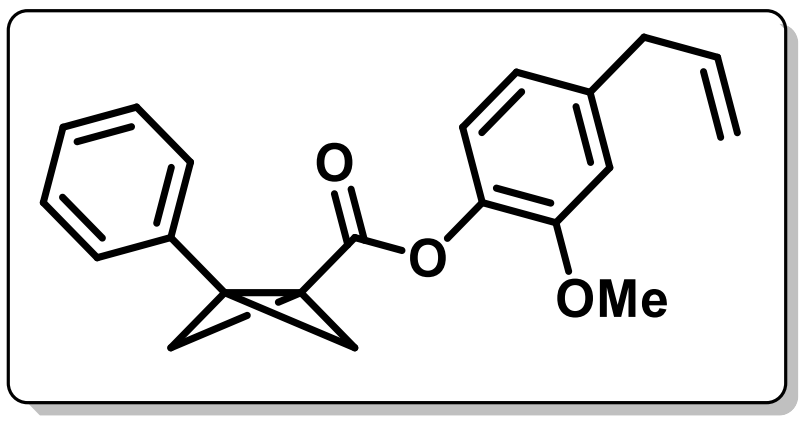

Following the general procedure (GP1), **1q** was isolated as a colorless solid (0.371 g, 58% yield over two steps). The physical data are matching with the reported value.

^{1}H NMR ((400 MHz, $CDCl_3$) δ (ppm)) δ 7.49 – 7.27 (m, 6H), 6.68 (d, *J* = 1.9 Hz, 1H), 6.62 (dd, *J* = 8.0, 1.9 Hz, 1H), 6.40 (d, *J* = 8.0 Hz, 1H), 6.04 – 5.85 (m, 1H), 5.29 – 4.97 (m, 2H), 3.53 (s, 3H), 3.32 (dt, *J* = 6.7, 1.5 Hz, 2H), 3.13 (t, *J* = 1.3 Hz, 2H), 1.76 (t, *J* = 1.2 Hz, 2H).

^{13}C {^{1}H} NMR ((101 MHz, $CDCl_3$) δ (ppm)) δ 168.11, 151.02, 138.80, 137.99, 137.26, 133.57, 128.60, 127.26, 126.52, 122.78, 120.60, 116.14, 112.71, 55.66, 40.16, 36.41, 34.76, 23.54.

HRMS (ESI) Calcd. for $C_{21}H_{20}O_3$ $[M+H]^+$, 321.1485, found: 321.1499.

2.2. Synthesis of *N*-Methoxy-*N*-methyl-3-phenylbicyclo[1.1.0]butane-1-carboxamide (1r) :

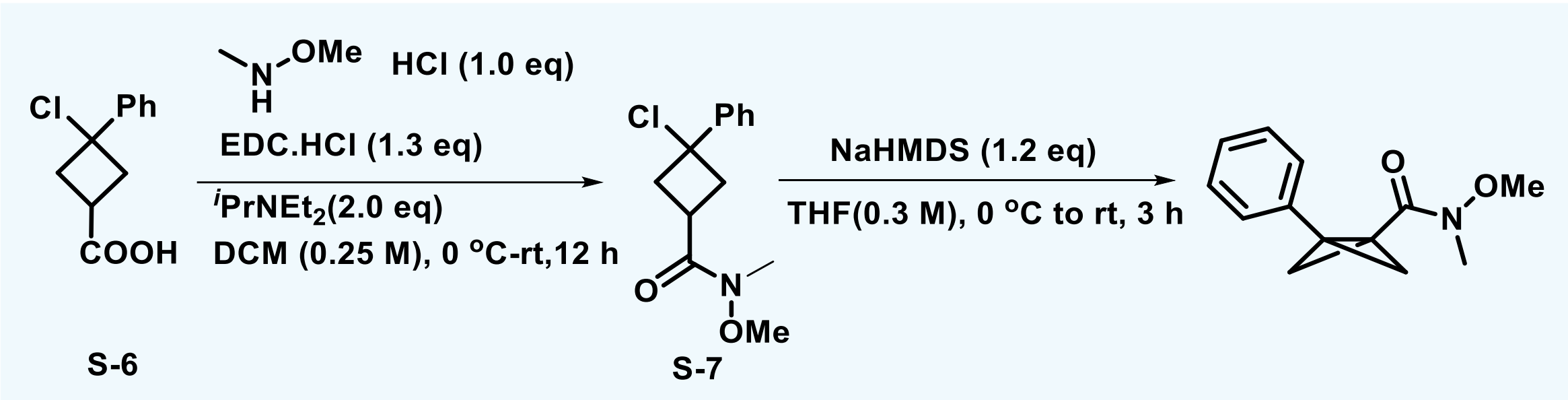

To the stirred solution of 3-chloro-3-phenylcyclobutane-1-carboxylic acid **S-6** (2.03 g, 1.0 equiv., 9.65 mmol) in DCM (0.25 M, 24.0 mL), N,O-dimethylhydroxylamine hydrochloride (0.988 g, 1.0 equiv., 9.65 mmol) and ^{i}PrNEt$_2$ (3.36 mL, 20.0 mmol) were added sequentially. After stirring for 5 minutes, EDC-HCl (2.03 g, 1.3 equiv., 13.0 mmol) was added at 0 °C and the reaction was allowed to stir at rt for 12 hours. Upon completion, the reaction was quenched with aqueous NH_4Cl solution and the aqueous layer was extracted with DCM (3 x 20 mL). The combined organic layers were dried over anhydrous Na_2SO_4, filtered, and concentrated by rotary evaporation. The crude residue was used without any further purification.

In the final step, an oven-dried 50 mL round bottom flask equipped with a stir bar was cooled under a vacuum, backfilled with nitrogen (x 3), and capped with a septum. **S-7** (1 equiv., 9.65 mmol, assuming 100% reaction) and THF (0.3 M, 28 mL) were added under nitrogen atmosphere. The reaction mixture was cooled to 0 °C and NaHMDS (2M in THF, 5.7 mL, 2.0 equiv.) was added dropwise. The reaction mixture was allowed to stir for 30 minutes at same temperature, followed by 2.5 h at the room temperature before quenching with 30 mL NH_4Cl solution. The aqueous layer was extracted with EtOAc (3 x 30 mL). The combined organic layers were dried over anhydrous Na_2SO_4, filtered, and concentrated by rotary evaporation. The residue was purified by FCC on silica gel (pentane/ethyl acetate) to give **1r** (0.88 g, 43%). The physical and analytical data are matching with the reported value.

^{1}H NMR ((400 MHz, $CDCl_3$) δ (ppm)) δ 7.33 – 7.23 (m, 4H), 7.22 – 7.14 (m, 1H), 3.61 (s, 3H), 3.08 (s, 3H), 2.97 (t, *J* = 0.8 Hz, 2H), 1.57 (t, *J* = 0.8 Hz, 2H).

^{13}C {^{1}H} NMR ((101 MHz, $CDCl_3$) δ (ppm)) δ 169.30, 134.17, 128.54, 126.73, 126.37, 61.34, 36.70, 32.83, 22.72.

2.3.1 Naphthalen-2-yl(3-phenylbicyclo[1.1.0]butan-1-yl)methanone (1s):

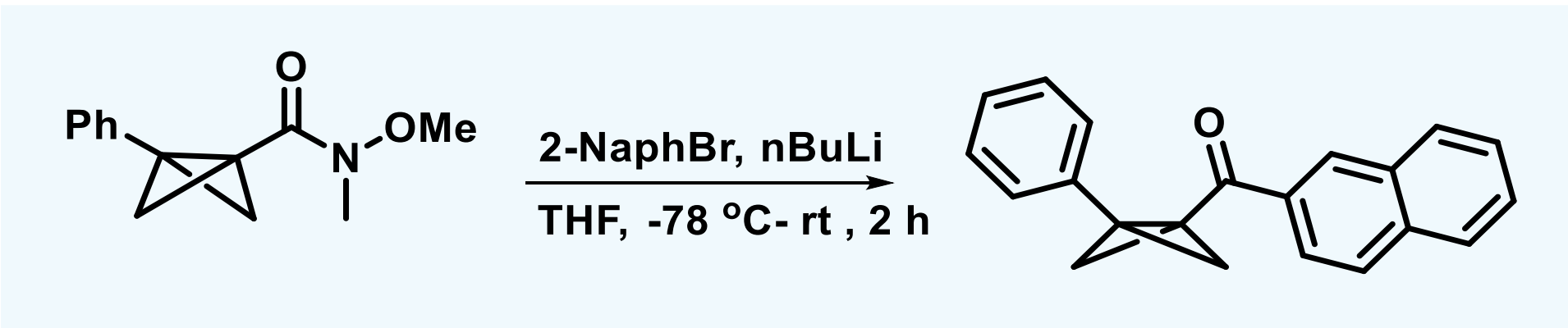

An oven-dried 250 mL round bottom flask equipped with a stir bar was cooled under vacuum. After backfilled with N_2 (x 3) and capped with a septum, 2-NaphBr (3.41 g, 16.5 mmol, 1.10 eq.) and THF (75 mL) were added. The solution was cooled to -78 ºC in a dry ice/acetone bath and nBuLi (2.5 M in Hex, 6.0 mL, 15 mmol, 1.0 eq.) was added. After stirred at the same temperature for 0.5 h, a solution of above crude amide (assuming 15 mmol, 1.0 eq.) in THF (15 mL) was added. After 0.5 h, the dry ice/acetone bath was removed, and the reaction was stirred at room temperature for 1.5 h before quenched with saturated NH_4Cl solution (50 mL). The aqueous layer was extracted with EtOAc (3 x 30 mL). The combined organic layers were washed with brine (100 mL), dried over anhydrous $MgSO_4$, filtered and concentrated by rotary evaporation. The residue was purified by FCC (Hex:EtOAc = 19:1 to 9:1) as white solid (70% yield).

^{1}H NMR ((400 MHz, $CDCl_3$) δ (ppm)) δ 8.13 (dt, *J* = 1.5, 0.7 Hz, 1H), 7.88 (ddt, *J* = 7.6, 1.3, 0.7 Hz, 1H), 7.85 – 7.80 (m, 1H), 7.76 (dq, *J* = 8.4, 0.6 Hz, 1H), 7.57 – 7.47 (m, 3H), 7.24 – 7.17 (m, 3H), 7.16 – 7.11 (m, 2H), 3.23 (t, *J* = 1.3 Hz, 2H), 1.97 (t, *J* = 1.3 Hz, 2H).

^{13}C {^{1}H} NMR ((101 MHz, $CDCl_3$) δ (ppm)) δ 196.53, 135.73, 135.07, 132.89, 132.29, 129.87, 129.36, 128.51, 128.05, 128.02, 127.86, 127.34, 126.69, 126.15, 124.53, 38.90, 37.95, 31.58.

2.3.2 (3-Methoxyphenyl)(3-phenylbicyclo[1.1.0]butan-1-yl)methanone (1t):

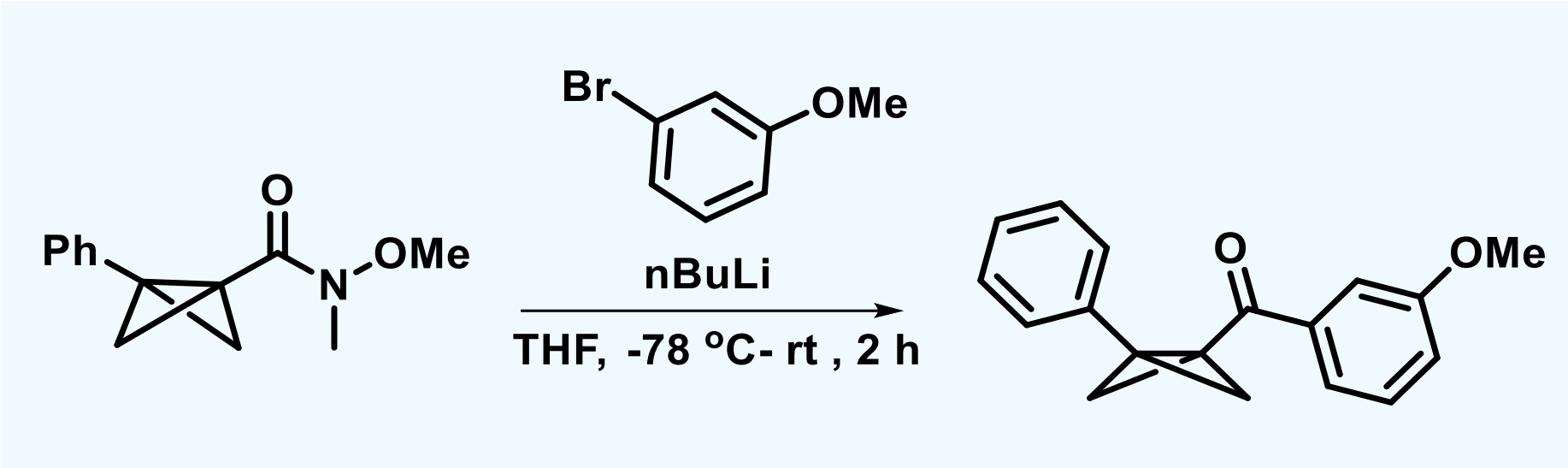

An oven-dried 250 mL round bottom flask equipped with a stir bar was cooled under vacuum. After backfilled with N_2 (x 3) and capped with a septum, 1-bromo-3-methoxybenzene (1.96 ml, 16.5 mmol, 1.10 eq.) and THF (75 mL) were added. The solution was cooled to -78 °C in a dry ice/acetone bath and nBuLi (2.5 M in Hex, 6.0 mL, 15 mmol, 1.0 eq.) was added. After stirred at the same temperature for 0.5 h, a solution of above crude amide (assuming 15 mmol, 1.0 eq.) in THF (15 mL) was added. After 0.5 h, the dry ice/acetone bath was removed, and the reaction was stirred at room temperature for 1.5 h before quenched with saturated NH_4Cl solution (50 mL). The aqueous layer was extracted with EtOAc (3 x 30 mL). The combined organic layers were washed with brine (100 mL), dried over anhydrous $MgSO_4$, filtered and concentrated by rotary evaporation. The residue was purified by FCC (Hexane : ethyl acetate = 19:1 to 9:1) as a white solid (73% yield).

^{1}H NMR ((400 MHz, $CDCl_3$) δ (ppm)) δ 7.30 – 7.10 (m, 7H), 7.02 – 6.91 (m, 2H), 3.70 (dd, *J* = 2.8, 1.2 Hz, 3H), 3.16 (t, *J* = 1.3 Hz, 2H), 1.89 (t, *J* = 1.2 Hz, 2H).

^{13}C {^{1}H} NMR ((101 MHz, $CDCl_3$) δ (ppm)) δ 196.41, 159.38, 139.71, 132.93, 129.05, 128.49, 127.31, 126.15, 121.31, 118.41, 112.44, 55.40, 38.83, 37.81, 31.48.

2.4. Synthesis of *N*-Methoxy-*N*-methyl-3-phenylbicyclo[1.1.0]butane-1-carboxamide (1u) :

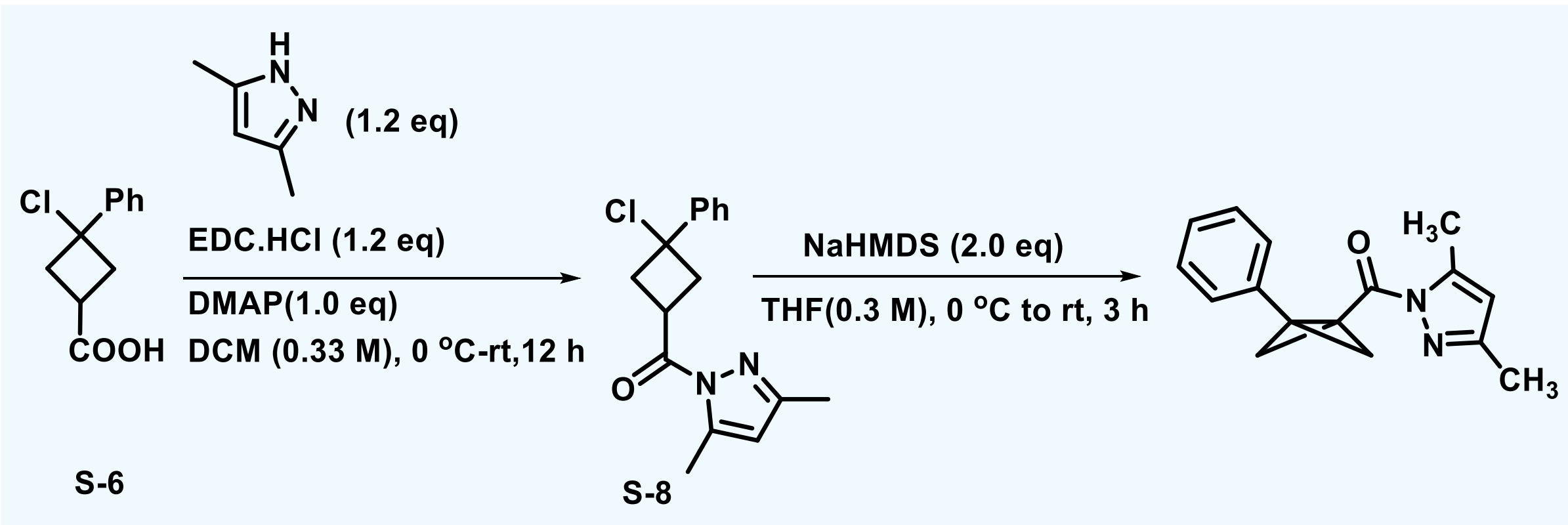

To a stirred solution of 3-chloro-3-phenylcyclobutane-1-carboxylic acid (1.053 g, 1.0 equiv., 0.005 mol) in DCM (0.33 M, 15 mL), 3,5-dimethyl-1H-pyrazol (1.2 equiv., 6.0 mmol) and DMAP (0.62 g, 5.00 mmol) were added sequentially. After stirring for 5 minutes, EDC-HCl (1.20 g, 1.2 equiv., 6.00 mmol) was added at 0 °C and the reaction was allowed to stir at rt for 12 hours. Upon completion, the reaction

was quenched with aqueous NH_4Cl solution and the aqueous layer was extracted with DCM (3 x 20 mL). The combined organic layers were dried over anhydrous Na_2SO_4, filtered, and concentrated by rotary evaporation. The crude residue was purified by FCC (hexane/ethyl acetate) to provide **S-8** (0.88 g, 62%).

In the final step, an oven-dried 50 mL round bottom flask equipped with a stir bar was cooled under a vacuum, backfilled with N_2 (x 3), and capped with a septum. **S-8** (1 equiv., 3.1 mmol) and THF (0.3 M, 10 mL) were added under N_2 atmosphere. The reaction mixture was cooled to 0 °C and NaHMDS (2M in THF, 3.1 mL, 2.0 equiv.) was added dropwise. The reaction mixture was allowed to stir for 30 minutes at same temperature, followed by 2.5 h at the room temperature before quenching with 10 mL NH_4Cl solution. The aqueous layer was extracted with EtOAc (3 x 20 mL). The combined organic layers were dried over anhydrous Na_2SO_4, filtered, and concentrated by rotary evaporation. The residue was purified by FCC on silica gel (hexane/ethyl acetate) to give **1m** (0.71 g, 87%). The physical and analytical data are matching with the reported value.[5]

^{1}H NMR ((400 MHz, $CDCl_3$) δ (ppm)) δ 7.33 – 7.22 (m, 2H), 7.23 – 7.17 (m, 2H), 7.16 – 7.11 (m, 1H), 5.76 (d, J = 1.1 Hz, 1H), 3.36 (t, J = 1.2 Hz, 2H), 2.18 (s, 3H), 2.11 (d, J = 1.0 Hz, 3H), 1.83 (t, J = 1.2 Hz, 2H).

^{13}C {^{1}H} NMR ((101 MHz, $CDCl_3$) δ (ppm)) δ 169.05, 151.40, 143.61, 133.24, 128.45, 127.25, 126.35, 110.09, 38.83, 26.56, 13.89, 13.87.

2.5. Synthesis of Benzyl-bicyclo[1.1.0]butane-1-carboxylate (1v):

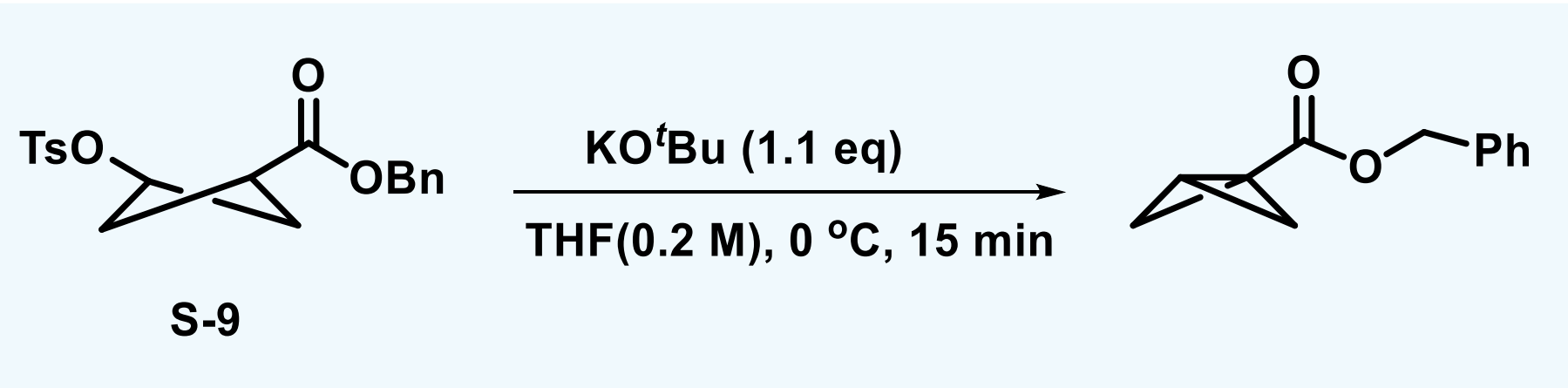

Note: **S-9** was prepared following a known procedure.[3]

An oven-dried 100 mL round bottom flask equipped with a stir bar was cooled under a vacuum, backfilled with N_2 (x 3), and capped with a septum. **S-9** (1.0 g, 1.0 equiv., 2.77 mmol) and THF (0.2 M, 14 mL) were added under nitrogen atmosphere. The reaction mixture was cooled to 0 °C and KO^{t}Bu (0.73M in THF, 4.0 mL, 1.1 equiv.) was added dropwise. The reaction mixture was allowed to stir for 15 minutes at the same temperature before quenching with 10 mL NH_4Cl solution. The aqueous layer was extracted with EtOAc (3 x 30 mL). The combined organic layers were dried over anhydrous Na_2SO_4, filtered, and concentrated by rotary evaporation. The crude residue was purified by FCC on silica gel (hexane/ethyl acetate) to give **1v** (0.338 g, 65%) as colorless oil. The physical and analytical data are matching with the reported value.[9]

^{1}H NMR ((400 MHz, $CDCl_3$) δ (ppm)) δ 7.41 – 7.24 (m, 5H), 5.17 (s, 2H), 2.40 (dt, J = 3.5, 1.1 Hz, 2H), 2.10 (pd, J = 3.3, 0.8 Hz, 1H), 1.18 (dt, J = 2.8, 1.0 Hz, 2H).

^{13}C {^{1}H} NMR ((101 MHz, $CDCl_3$) δ (ppm)) δ 172.99, 136.40, 128.63, 128.21, 128.08, 66.39, 35.77, 16.83, 9.22.

2.6. *N*-Methyl-*N*-phenylbicyclo[1.1.0]butane-1-carboxamide (1w):

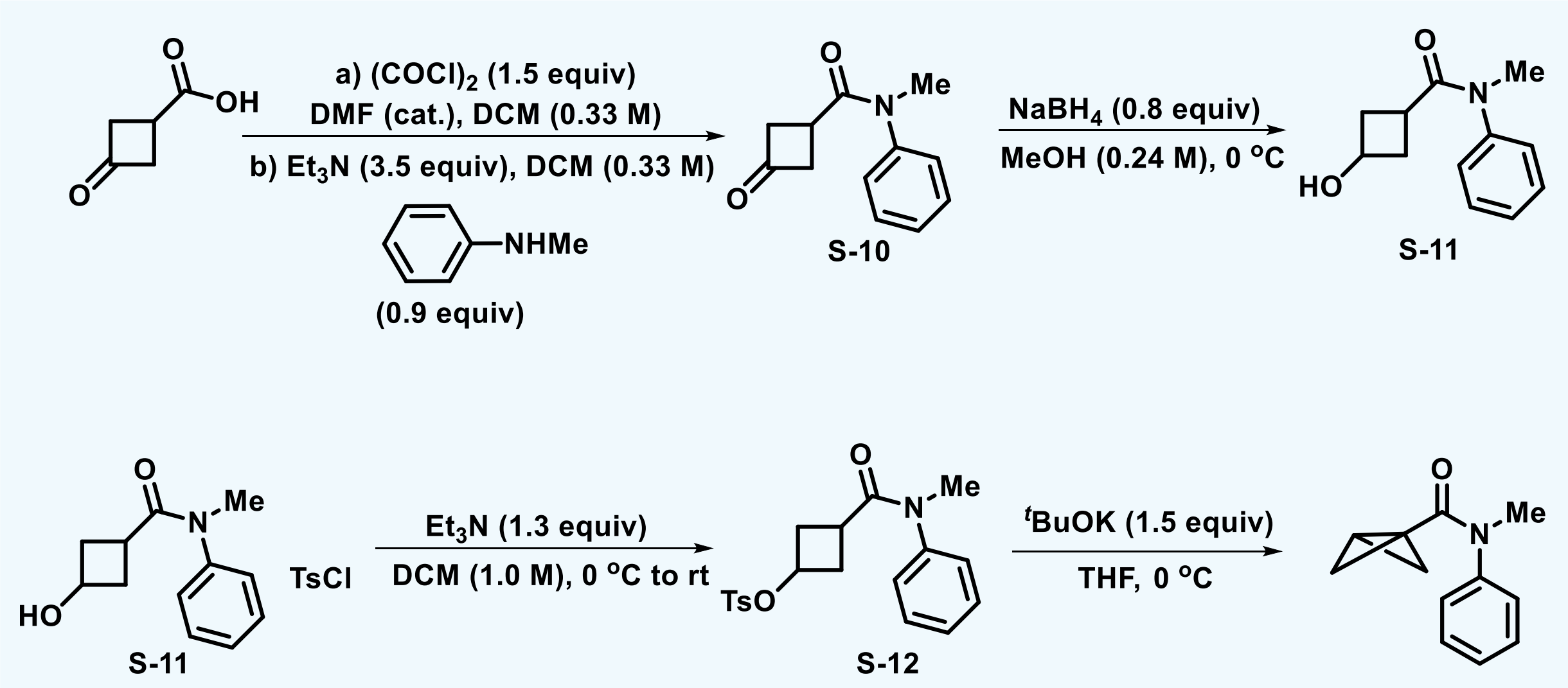

a) Following a slightly modified procedure,[4] to a 50 mL round bottom flask under inert condition, a solution of 3-oxocyclobutane-1-carboxylic acid (570 mg, 5.00 mmol, 1.00 equiv) in dry dichloromethane (15 mL, 0.33 M) was prepared and cooled down to 0 °C. To this solution, DMF (3 drops) and oxalyl chloride (0.64 mL, 7.5 mmol, 1.5 equiv) were added dropwise and stirred for 2 hours. Further, the reaction mixture was stirred for 1 hour at room temperature. Next, the solvent and remaining oxalyl chloride were evaporated under reduced pressure to obtain the crude acid chloride, which was used in the next step without further purification.

b) Under an inert condition, N-methylaniline (0.49 mL, 4.5 mmol, 0.90 equiv) and triethylamine (2.40 mL, 17.5 mmol, 3.50 equiv) were taken into a 50 mL round bottom flask and dissolved in dry DCM (15 mL, 0.33 M) and cooled down to 0 °C. To this solution, crude acyl chloride was added dropwise. Then, the reaction mixture was warmed to room temperature and stirred overnight. Next, the reaction mixture was quenched with a saturated aqueous $NaHCO_3$ solution (20 mL) and extracted with DCM (3×30 mL). The combined organic layers were dried over $MgSO_4$, and the solvent was evaporated under reduced pressure to obtain the compound **S-10** as a brown liquid (744 mg, 3.66 mmol, 73%). This crude compound was used in the next step without further purification.

Step II: A 50 mL round bottom flask was charged with ketone (744 mg, 3.66 mmol, 1.00 equiv) and MeOH (15 mL, 0.24 M). The resulting solution was cooled to 0 °C, and $NaBH_4$ (111 mg, 2.92 mmol, 0.800 equiv) was added portion wise. After 1 hour stirring at 0 °C, the reaction mixture was quenched with a saturated aqueous NH_4Cl solution (20 mL) and extracted with DCM (3×10 mL). The combined

organic phases were washed with brine (25 mL) and dried over Na_2SO_4. The solvent was removed under vacuum to afford the product **S-11** as light-yellow oil (713 mg, 3.47 mmol, 95%). The crude alcohol was used in the tosylation step without further purification.

Step III: A 50 mL two-neck round bottom flask charged with crude alcohol (713 mg, 3.47 mmol, 1.00 equiv) and dry DCM (10 mL, 0.35 M) under inert atmosphere. Then, the solution was cooled to 0 °C. To this solution, 4-toluenesulfonyl chloride (1.2 g, 6.2 mmol, 1.8 equiv) and Et_3N (0.63 mL, 4.5 mmol, 1.3 equiv) were added. After overnight stirring at room temperature, the reaction was quenched with saturated aqueous NH_4Cl (20 mL). The organic phases were extracted with DCM (3×30 mL) and dried over Na_2SO_4. The solvent was removed using rotary evaporator to afford desired product **S-12** as a pale-yellow oil (861 mg, 2.40 mmol, 69%). The crude product was used in the next step without purification.

Step IV: A 50 mL two-neck round bottom flask was charged with the tosylated product (861 mg, 2.40 mmol, 1.00 equiv) and THF (12 mL, 0.20 M) under inert atmosphere. Then, the solution was cooled to 0 °C and a solution of KO^{t}Bu (403 mg, 3.59 mmol, 1.50 equiv) in THF (5 mL, 0.73 M) prepared in an inert atmosphere was added dropwise and stirred vigorously for 30- 60 mins. The reaction mixture was monitored by TLC to confirm the complete consumption of the starting material. Then, the reaction mixture was quenched with a saturated aqueous NH_4Cl solution (20 mL) and extracted with Et_2O (3×30 mL). The combined organic phases were dried over Na_2SO_4. The solvent was removed under vacuum at room temperature. The crude product was purified by flash chromatography (silica, 1.5:8.5 v/v EtOAc:Hexane) to afford the desired product **1w** as a white solid (259 mg, 1.38 mmol, 58%).

^{1}H NMR ((400 MHz, $CDCl_3$) δ (ppm)) δ 7.41 – 7.29 (m, 2H), 7.29 – 7.17 (m, 3H), 3.33 (d, J = 0.6 Hz, 3H), 2.00 (ddd, J = 5.8, 3.3, 2.5 Hz, 1H), 1.82 – 1.76 (m, 2H), 0.78 – 0.73 (m, 2H).

^{13}C {^{1}H} NMR ((101 MHz, $CDCl_3$) δ (ppm)) δ 171.81, 145.22, 129.21, 126.96, 126.53, 38.00, 37.17, 16.95, 10.09.

2.7. Synthesis of benzyl 3-methylbicyclo[1.1.0]butane-1-carboxylate (1x):

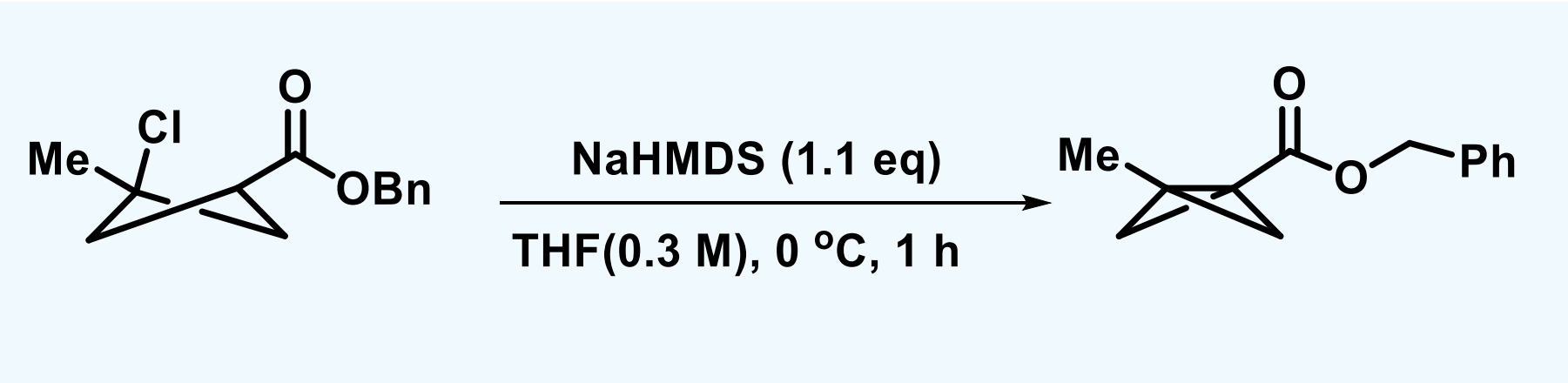

An oven-dried 100 mL round bottom flask equipped with a stir bar was cooled under a vacuum, backfilled with Ar (x 3), and capped with a septum. benzyl-3-chloro-3-methylcyclobutane-1-carboxylate[2] (3.19 g, 1.0 equiv., 13.3 mmol) and THF (0.3 M, 40 mL) were added under Ar atmosphere. The reaction mixture was cooled to 0 oC and NaHMDS (2M in THF, 7.3 mL, 1.1 equiv.) was added dropwise. The reaction mixture was allowed to stir for 1 h at the same temperature before quenching

with 30 mL NH4Cl solution. The aqueous layer was extracted with EtOAc (3 x 30 mL). The combined organic layers were dried over anhydrous Na2SO4, filtered, and concentrated by rotary evaporation. The crude residue was purified by FCC on silica gel (pentane/ethyl acetate) to give 1s (1.19 g, 44%) as colorless oil. The physical and analytical data are matching with the reported value.2

^{1}H NMR ((400 MHz, $CDCl_3$) δ (ppm)) δ 7.43 – 7.20 (m, 5H), 5.15 (s, 2H), 2.22 (t, J = 1.0 Hz, 2H), 1.48 (s, 3H), 1.24 (t, J = 1.0 Hz, 2H).

^{13}C {^{1}H} NMR ((101 MHz, $CDCl_3$) δ (ppm)) δ 172.00, 136.78, 128.57, 128.18, 128.15, 66.20, 27.88, 13.16, 12.75.

2.8. Synthesis of (3-substituted-1*H*-indol-2-yl)(aryl)methanols : (General procedure-2)

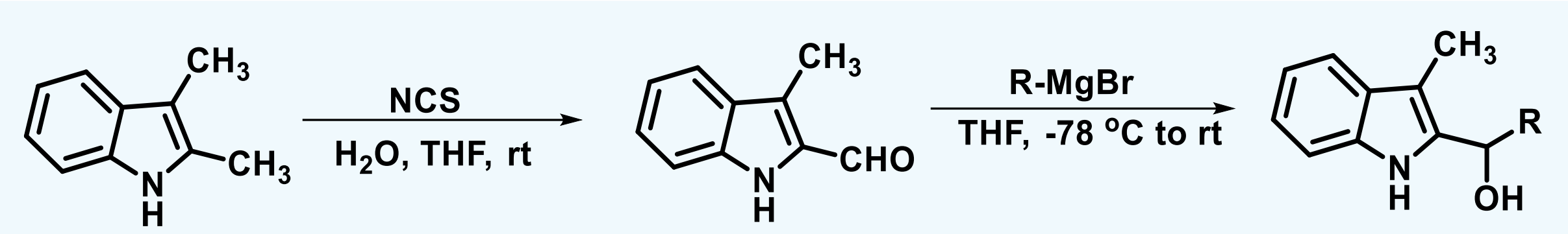

To the solution of 2,3-dimethylindole(7.0 mmol, 1.0 equiv.) in THF (35 mL) and H_2O (126.0 µL, 7.0 mmol, 1.0 equiv.) was added NCS (14.7 mmol, 1.96 g, 2.1 equiv.), and then stirred at room temperature. After the completion of the reaction, as indicated by TLC, the reaction was quenched with water. The resulting mixture was extracted with ethyl acetate and the combined organic layers were dried over Na_2SO_4 and evaporated under reduced pressure. The residue was purified by column chromatography (hexane/ethyl acetate = 50:1) on silica gel to afford desired product (858.0 mg, 77% yield)

To a solution of aldehyde (1.0 g, 6.28 mmol, 1 equiv) in THF (20 ml) Grignard reagent [25.16 mmol, 4 equiv, strength of Grignard 1 M; generated from corresponding aryl bromide (25.16 mmol) and Mg (1.22 g, 50.3 mmol) in 25 ml THF] was added dropwise at -78 °C. Then, the cooling bath was removed and the reaction mixture was stirred for 40 min. The reaction mixture was cooled to 0 °C, saturated aq. NH_4Cl was added to the reaction mixture and extracted with EtOAc (2 x 75). Solvent was evaporated under reduced pressure at 25 °C and residue was purified by flash chromatography over silica gel column using hexane/EtOAc (5-15%) as eluent.

(3-Methyl-1*H*-indol-2-yl)(phenyl)methanol (2a):

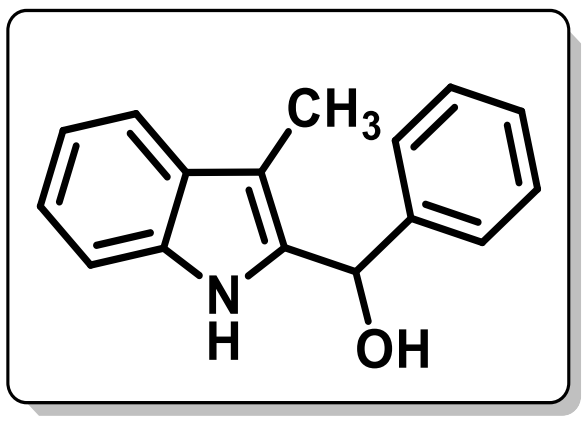

Following the general procedure (GP2), **2a** was isolated as a yellow solid (1.310 g, 88% yield). The physical data are matching with the reported value.

^{1}H NMR ((400 MHz, $CDCl_3$) δ (ppm)) δ 8.12 (s, 1H), 7.59 (ddt, *J* = 7.4, 1.6, 0.8 Hz, 1H), 7.44 – 7.32 (m, 4H), 7.24 – 7.20 (m, 1H), 7.20 – 7.12 (m, 2H), 6.07 (s, 1H), 2.89 (s, 1H), 2.28 (s, 3H).

^{13}C {^{1}H} NMR ((101 MHz, $CDCl_3$) δ (ppm)) δ 141.92, 135.53, 135.09, 129.13, 128.79, 128.00, 126.43, 122.25, 119.38, 118.90, 111.14, 108.41, 68.72, 8.62.

HRMS (ESI) Calcd. for $C_{16}H_{15}NO$ $[M-H]^-$, 236.1081, found: 236.1076

(3-Methyl-1*H*-indol-2-yl)(*p*-tolyl)methanol (2b):

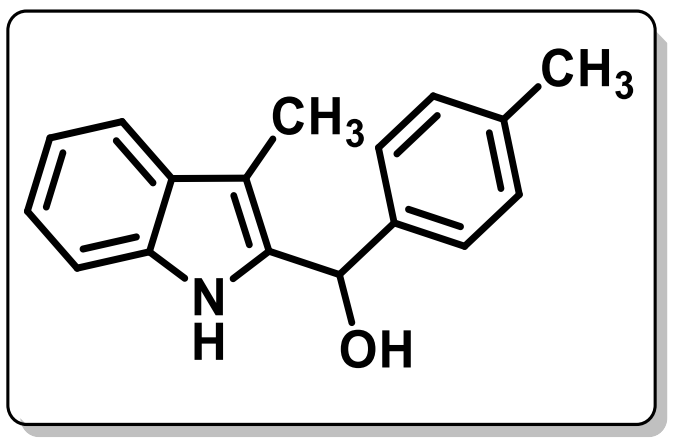

Following the general procedure (GP2), **2b** was isolated as a yellow solid (1.277 g, 81% yield). The physical data are matching with the reported value.

^{1}H NMR ((400 MHz, $CDCl_3$) δ (ppm)) δ 8.06 (s, 1H), 7.53 (ddt, *J* = 7.7, 1.5, 0.8 Hz, 1H), 7.34 – 7.22 (m, 4H), 7.19 – 7.05 (m, 4H), 6.11 (s, 1H), 2.34 (s, 3H), 2.27 (s, 3H).

^{13}C {^{1}H} NMR ((101 MHz, $CDCl_3$) δ (ppm)) δ 138.97, 137.79, 135.37, 135.23, 129.45, 129.18, 126.33, 122.12, 119.29, 118.80, 110.98, 108.19, 68.72, 21.21, 8.59.

HRMS (ESI) Calcd. for $C_{17}H_{17}NO$ $[M-OH]^+$, 234.1277, found: 234.1275

(4-(*tert*-butyl)phenyl)(3-methyl-1*H*-indol-2-yl)methanol (2c):

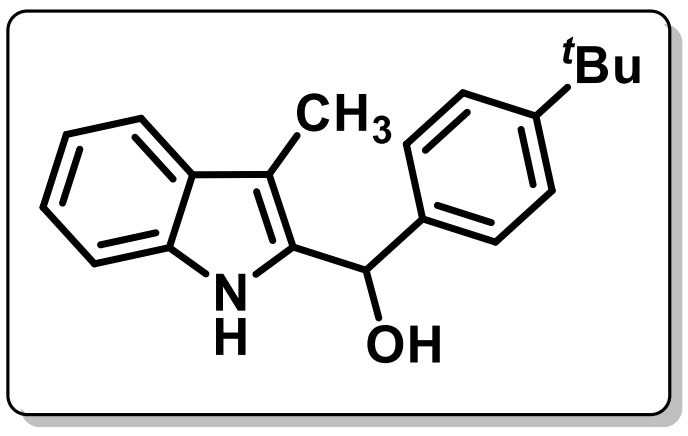

Following the general procedure (GP2), **2c** was isolated as a yellow solid (1.436 g, 78% yield). The physical data are matching with the reported value.

^{1}H NMR ((400 MHz, $CDCl_3$) δ (ppm)) δ 8.09 (s, 1H), 7.54 (dtt, *J* = 7.8, 1.5, 0.7 Hz, 1H), 7.41 – 7.29 (m, 4H), 7.28 – 7.21 (m, 1H), 7.19 – 7.05 (m, 3H), 6.12 (d, *J* = 3.1 Hz, 1H), 2.28 (s, 3H), 1.34 – 1.28 (s, 9H).

^{13}C {^{1}H} NMR ((101 MHz, $CDCl_3$) δ (ppm)) δ 151.01, 138.95, 135.42, 135.24, 129.15, 126.07, 125.69, 122.12, 119.28, 118.81, 111.00, 108.22, 68.61, 34.65, 31.42, 8.62.

HRMS (ESI) Calcd. for $C_{20}H_{23}NO$ $[M-OH]^+$, 276.1247, found: 276.1755

(3-Methyl-1*H*-indol-2-yl)(naphthalen-2-yl)methanol (2d):

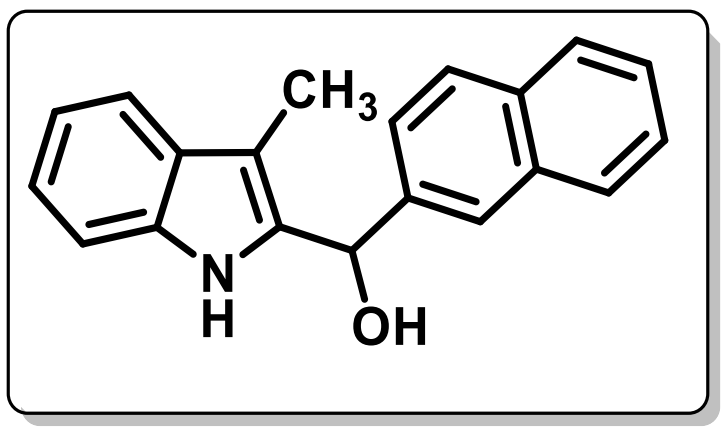

Following the general procedure (GP2), **2d** was isolated as a yellow liquid (1.478 g, 82% yield). The physical data are matching with the reported value.

^{1}H NMR (400 MHz, DMSO-D_6) δ 10.65 (s, 1H), 7.95 (d, *J* = 1.7 Hz, 1H), 7.91 – 7.76 (m, 3H), 7.53 – 7.34 (m, 4H), 7.23 (m, 1H), 7.00 – 6.84 (m, 2H), 6.15 – 6.06 (m, 2H), 3.29 (d, *J* = 0.8 Hz, 1H), 2.46 (p, *J* = 1.8 Hz, 6H), 2.25 (s, 3H).

^{13}C {^{1}H} NMR (101 MHz, DMSO-D_6) δ 142.17, 137.96, 136.00, 133.35, 132.72, 129.01, 128.36, 128.14, 128.02, 126.61, 126.18, 125.48, 124.57, 121.21, 118.55, 118.46, 111.63, 105.61, 67.96, 9.11.

HRMS (ESI) Calcd. for $C_{20}H_{17}NO$ $[M\text{-}OH]^+$, 270.1277, found: 270.1283

(4-Methoxyphenyl)(3-methyl-1*H*-indol-2-yl)methanol (2e):

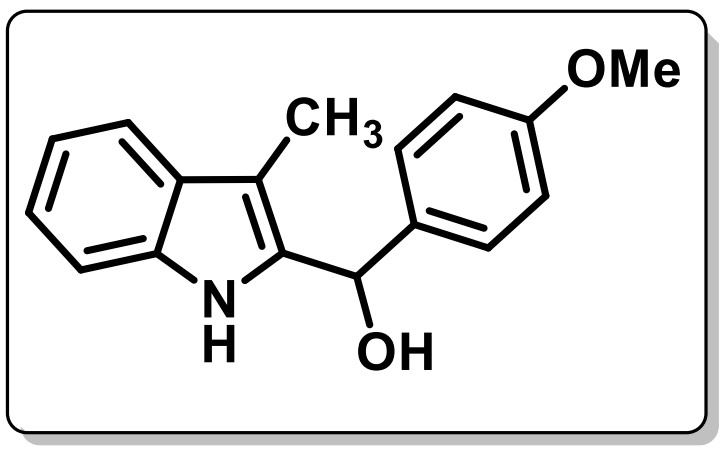

Following the general procedure (GP2), **2e** was isolated as a yellow solid (1.107 g, 66% yield). The physical data are matching with the reported value.

^{1}H NMR ((400 MHz, $CDCl_3$) δ (ppm)) δ 8.18 (s, 1H), 7.56 (ddt, *J* = 7.5, 1.6, 0.8 Hz, 1H), 7.28 – 7.24 (m, 2H), 7.24 – 7.10 (m, 3H), 6.89 – 6.81 (m, 2H), 6.01 (s, 1H), 3.78 (s, 3H), 2.23 (s, 3H).

^{13}C {^{1}H} NMR ((101 MHz, $CDCl_3$) δ (ppm)) δ 159.32, 135.40, 135.35, 134.19, 129.21, 127.81, 122.09, 119.31, 118.80, 114.11, 111.10, 108.01, 68.50, 55.42, 8.59.

HRMS (ESI) Calcd. for $C_{17}H_{17}NO_2$ $[M\text{-}OH]^+$, 250.1226, found: 250.1232.

(3-Methoxyphenyl)(3-methyl-1*H*-indol-2-yl)methanol (2f):

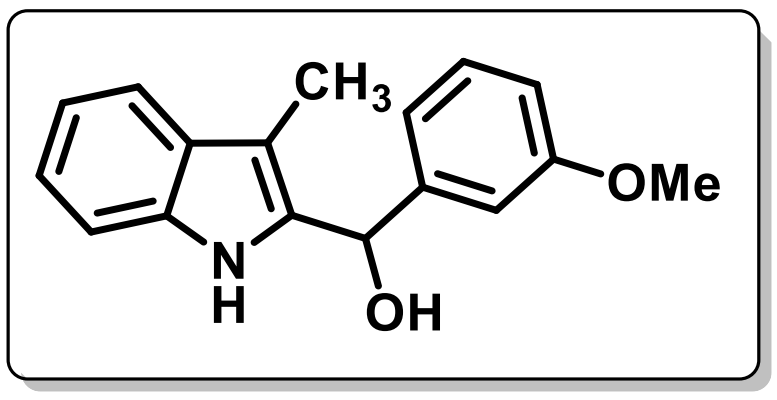

Following the general procedure (GP2), **2f** was isolated as a yellow liquid (1.308 g, 78% yield). The physical data are matching with the reported value.

^{1}H NMR ((400 MHz, $CDCl_3$) δ (ppm)) δ 8.07 (s, 1H), 7.56 (ddt, *J* = 7.6, 1.5, 0.8 Hz, 1H), 7.31 – 7.08 (m, 5H), 7.03 – 6.91 (m, 2H), 6.84 (ddd, *J* = 8.2, 2.6, 1.0 Hz, 1H), 6.05 (s, 1H), 3.77 (s, 3H), 2.65 (s, 1H), 2.29 (s, 3H).

^{13}C {^{1}H} NMR ((101 MHz, $CDCl_3$) δ (ppm)) δ 159.99, 143.57, 135.51, 134.97, 129.84, 129.11, 122.25, 119.34, 118.89, 118.69, 113.26, 112.08, 111.09, 108.46, 68.58, 55.37, 8.61.

HRMS (ESI) Calcd. for $C_{17}H_{17}NO_2$ $[M+H]^+$, 268.1332, found: 268.1345.

(2-Methoxyphenyl)(3-methyl-1*H*-indol-2-yl)methanol (2g):

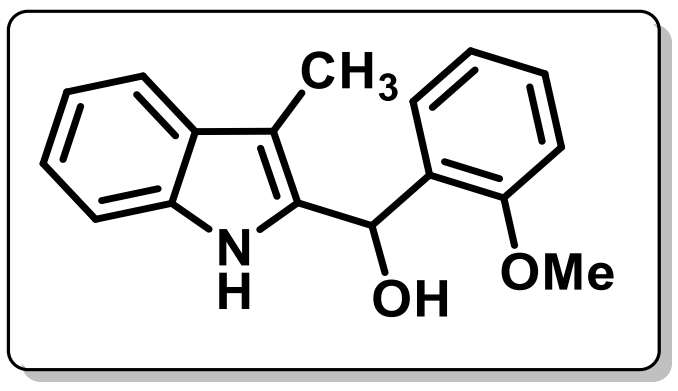

Following the general procedure (GP2), **2g** was isolated as a yellow solid (0.905 g, 54% yield). The physical data are matching with the reported value.

^{1}H NMR ((400 MHz, $CDCl_3$) δ (ppm)) δ 8.35 (s, 1H), 7.57 (ddt, *J* = 7.7, 1.5, 0.8 Hz, 1H), 7.35 – 7.26 (m, 2H), 7.21 – 7.03 (m, 3H), 6.97 – 6.88 (m, 2H), 6.38 (s, 1H), 3.89 (s, 3H), 2.25 (s, 3H).

^{13}C {^{1}H} NMR ((101 MHz, $CDCl_3$) δ (ppm)) δ 156.87, 135.42, 134.21, 130.25, 129.32, 129.25, 127.72, 121.82, 121.13, 119.14, 118.72, 110.98, 110.79, 107.98, 65.74, 55.64, 8.56.

HRMS (ESI) Calcd. for $C_{17}H_{17}NO_2$ $[M-OH]^+$, 250.1226, found: 250.1238.

(4-Fluorophenyl)(3-methyl-1*H*-indol-2-yl)methanol (2h):

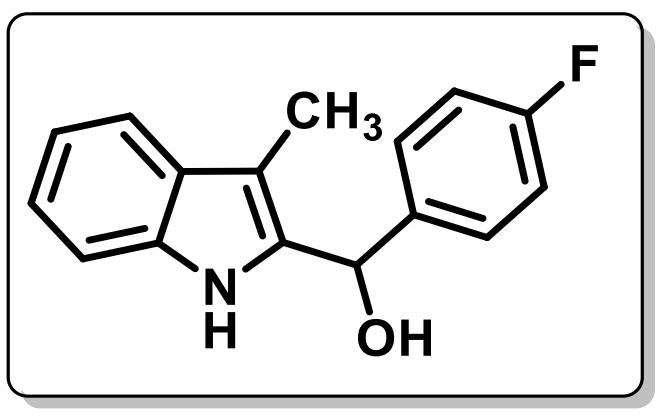

Following the general procedure (GP2), **2h** was isolated as a yellow solid (1.249 g, 78% yield). The physical data are matching with the reported value.

^{1}H NMR ((400 MHz, $CDCl_3$) δ (ppm)) δ 8.18 (s, 1H), 7.67 – 7.59 (m, 1H), 7.31 – 7.26 (m, 2H), 7.25 – 7.15 (m, 3H), 7.09 – 6.98 (m, 2H), 5.97 (d, *J* = 1.0 Hz, 1H), 2.27 (s, 3H).

^{13}C {^{1}H} NMR ((101 MHz, $CDCl_3$) δ (ppm)) δ 162.45 (d, *J* = 246.3 Hz), 137.67 (d, *J* = 3.3 Hz), 135.60, 134.86, 129.11, 128.21 (d, *J* = 8.2 Hz), 122.46, 119.58, 119.02, 115.71, δ 115.61 (d, *J* = 21.4 Hz)., 111.25, 108.49, 68.12, 8.60.

^{19}F NMR ((376 MHz, $CDCl_3$) δ (ppm)) δ -114.28.

HRMS (ESI) Calcd. for $C_{16}H_{14}NOF$ $[M-H]^-$, 254.0987, found: 254.0984.

(4-Chlorophenyl)(3-methyl-1*H*-indol-2-yl)methanol (2i):

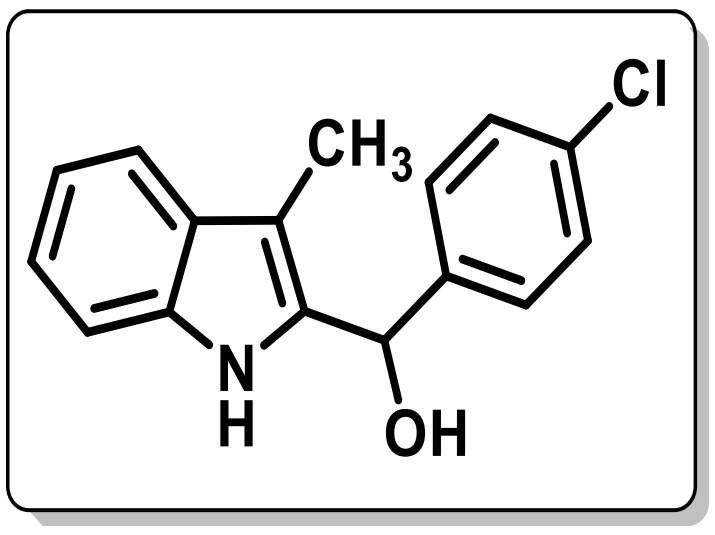

Following the general procedure (GP2), **2i** was isolated as a yellow liquid (1.361 g, 80% yield). The physical data are matching with the reported value.

^{1}H NMR ((400 MHz, $CDCl_3$) δ (ppm)) δ 8.13 (s, 1H), 7.65 – 7.57 (m, 1H), 7.35 – 7.24 (m, 3H), 7.23 – 7.13 (m, 3H), 5.95 (s, 1H), 2.26 (s, 3H).

^{13}C {^{1}H} NMR ((101 MHz, $CDCl_3$) δ (ppm)) δ 140.36, 135.53, 134.63, 133.71, 129.01, 128.85, 127.77, 122.44, 119.48, 118.93, 111.06, 108.60, 67.99, 8.60.

HRMS (ESI) Calcd. for $C_{16}H_{14}NOCl$ $[M-H]^-$, 270.0691, found: 270.0692.

(3,4-dichlorophenyl)(3-methyl-1*H*-indol-2-yl)methanol (2j):

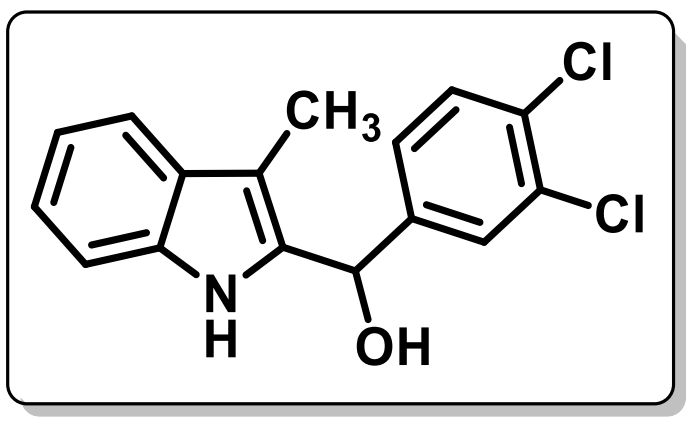

Following the general procedure (GP2), **2j** was isolated as a yellow liquid (1.245 g, 65% yield). The physical data are matching with the reported value.

^{1}H NMR ((400 MHz, $CDCl_3$) δ (ppm)) δ 8.10 (s, 1H), 7.58 (ddt, J = 7.7, 1.5, 0.9 Hz, 1H), 7.49 (dd, J = 2.2, 0.8 Hz, 1H), 7.35 (d, J = 8.3 Hz, 1H), 7.24 – 7.19 (m, 2H), 7.14 (m, 1H), 7.14 – 7.06 (m, 1H), 5.95 (d, J = 0.8 Hz, 1H), 2.27 (s, 3H).

^{13}C {^{1}H} NMR ((101 MHz, $CDCl_3$) δ (ppm)) δ 142.14, 135.69, 134.09, 132.83, 131.83, 130.63, 128.89, 128.25, 125.75, 122.70, 119.64, 119.08, 111.21, 108.96, 67.36, 8.65.

HRMS (ESI) Calcd. for $C_{16}H_{13}NOCl_2$ $[M-H]^-$, 304.0301, found: 304.0299.

(3-Methyl-1*H*-indol-2-yl)(4-(trifluoromethoxy)phenyl)methanol (2k):

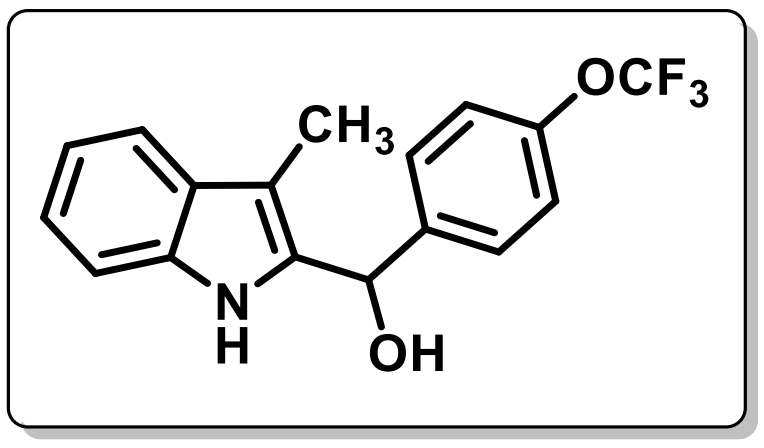

Following the general procedure (GP2), **2k** was isolated as a yellow solid (1.552 g, 77% yield). The physical data are matching with the reported value.

^{1}H NMR ((400 MHz, $CDCl_3$) δ (ppm)) δ 8.11 (s, 1H), 7.64 – 7.57 (m, 1H), 7.40 – 7.29 (m, 2H), 7.27 – 7.12 (m, 5H), 6.02 (s, 1H), 2.27 (s, 3H).

^{13}C {^{1}H} NMR ((101 MHz, $CDCl_3$) δ (ppm)) δ 148.82 (q, J = 7 Hz), 140.45, 135.65, 134.52, 128.98, 127.83, 122.58, 121.16, 119.62, 119.03, 111.18, 108.76, 67.91, 8.53.

^{19}F NMR ((376 MHz, $CDCl_3$) δ (ppm)) δ -57.67

HRMS (ESI) Calcd. for $C_{17}H_{14}NO_2F_3$ $[M-H]^-$, 320.0904, found: 320.0906.

(3-Methyl-1*H*-indol-2-yl)(4-(trifluoromethyl)phenyl)methanol (2l):

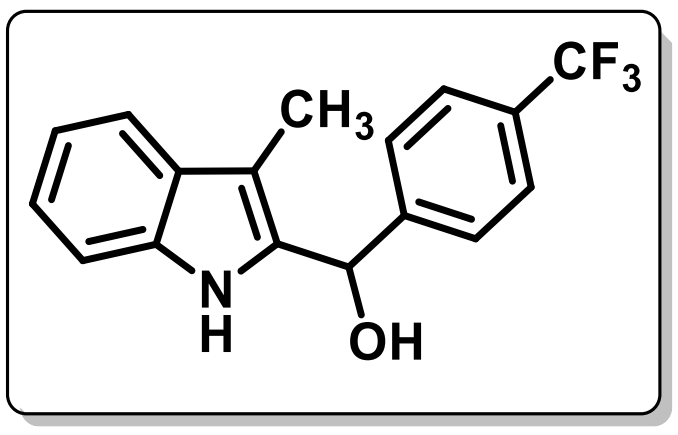

Following the general procedure (GP2), **2l** was isolated as a yellow solid (1.609 g, 84% yield). The physical data are matching with the reported value.

^{1}H NMR ((400 MHz, $CDCl_3$) δ (ppm)) δ 8.26 (s, 1H), 7.83 – 7.71 (m, 1H), 7.58 (ddt, J = 8.8, 7.4, 0.8 Hz, 3H), 7.46 (dtd, J = 8.3, 1.6, 0.8 Hz, 2H), 7.26 – 7.19 (m, 2H), 6.09 (d, J = 1.2 Hz, 1H), 2.29 (s, 3H).

^{13}C {^1H} NMR ((101 MHz, $CDCl_3$) δ (ppm)) δ 145.85, 135.73, 134.45, 130.15 (q, *J* = 225 Hz), 128.93, 126.64, 125.61 (q, *J* = 3.8 Hz), 122.62, 121.79 (q, *J* = 118 Hz), 119.61, 119.06, 111.20, 108.87, 67.97, 8.58.

^{19}F NMR ((376 MHz, $CDCl_3$) δ (ppm)) δ -62.26.

HRMS (ESI) Calcd. for $C_{17}H_{14}NOF_3$ $[M-H]^-$, 304.0955, found: 304.0951.

(3-Methyl-1*H*-indol-2-yl)(thiophen-2-yl)methanol (2m):

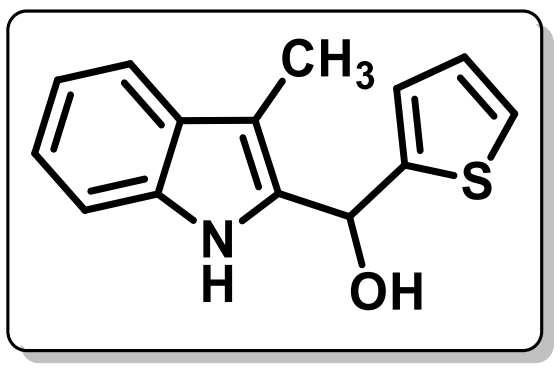

Following the general procedure (GP2), **2m** was isolated as a yellow solid (0.992 g, 65% yield). The physical data are matching with the reported value.

^1H NMR ((400 MHz, $CDCl_3$) δ (ppm)) δ 8.27 (s, 1H), 7.55 (ddt, *J* = 7.8, 1.4, 0.8 Hz, 1H), 7.31 – 7.25 (m, 2H), 7.18 (ddd, *J* = 8.2, 7.0, 1.3 Hz, 1H), 7.11 (ddd, *J* = 8.1, 7.0, 1.1 Hz, 1H), 6.94 (dd, *J* = 5.0, 3.5 Hz, 1H), 6.89 (dt, *J* = 3.5, 1.1 Hz, 1H), 6.34 (d, *J* = 1.0 Hz, 1H), 2.27 (s, 3H).

^{13}C {^1H} NMR ((101 MHz, $CDCl_3$) δ (ppm)) δ 146.01, 135.43, 134.35, 129.01, 126.95, 125.65, 124.94, 122.41, 119.40, 118.96, 111.14, 108.38, 65.34, 8.55.

HRMS (ESI) Calcd. for $C_{14}H_{13}NOS$ $[M-H]^-$, 242.0645, found: 242.0647.

1-(3-Methyl-1*H*-indol-2-yl)ethan-1-ol (2n):

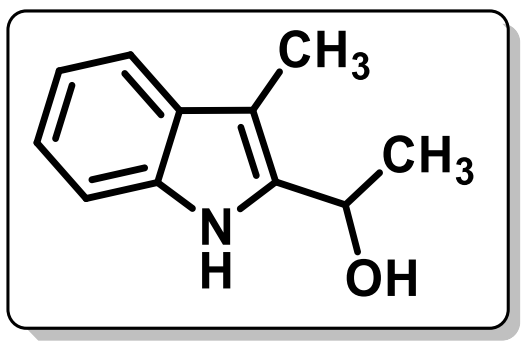

Following the general procedure (GP2), **2n** was isolated as a yellow semi-solid (0.934 g, 85%). The physical data are matching with the reported value.

^1H NMR ((400 MHz, $CDCl_3$) δ (ppm)) δ 8.46 (s, 1H), 7.57 (ddt, *J* = 7.6, 1.5, 0.8 Hz, 1H), 7.39 – 7.10 (m, 4H), 5.17 (q, *J* = 6.6 Hz, 1H), 2.27 (s, 3H), 1.53 (d, *J* = 6.6 Hz, 3H).

^{13}C {^1H} NMR ((101 MHz, $CDCl_3$) δ (ppm)) δ 137.20, 135.38, 129.12, 121.98, 119.29, 118.73, 111.08, 106.32, 63.03, 23.44, 8.43.

HRMS (ESI) Calcd. for $C_{11}H_{13}NO$ $[M-H]^-$, 174.0924, found: 174.0924.

1-(3-Methyl-1*H*-indol-2-yl)but-3-en-1-ol (2o):

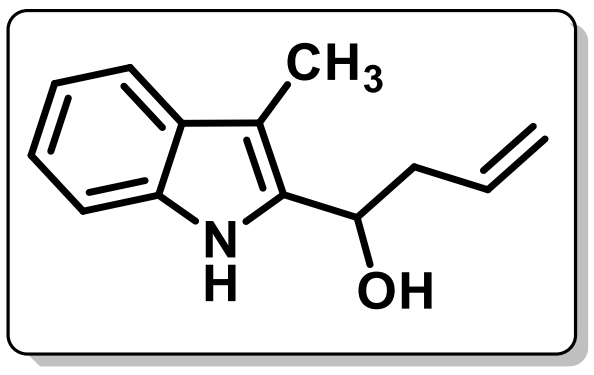

Following the general procedure (GP2), **2o** was isolated as a yellow solid (1.023 g, 81% yield). The physical data are matching with the reported value.

^{1}H NMR ((400 MHz, $CDCl_3$) δ (ppm)) δ 8.31 (s, 1H), 7.54 (ddt, J = 7.7, 1.4, 0.7 Hz, 1H), 7.29 (dt, J = 8.0, 1.1 Hz, 1H), 7.22 – 7.07 (m, 2H), 5.81 (dddd, J = 16.9, 10.2, 7.3, 6.8 Hz, 1H), 5.23 – 5.14 (m, 2H), 5.05 (dd, J = 7.0, 6.3 Hz, 1H), 2.64 – 2.55 (m, 2H), 2.26 (s, 3H).

^{13}C {^{1}H} NMR ((101 MHz, $CDCl_3$) δ (ppm)) δ 135.50, 135.38, 133.98, 129.12, 121.95, 119.24, 118.78, 118.68, 110.95, 107.02, 66.25, 42.20, 8.55.

HRMS (ESI) Calcd. for $C_{13}H_{15}NO$ $[M-H]^-$, 200.1081, found: 200.1079.

2.9. Synthesis of substrates 2p-2t: (General procedure-3)

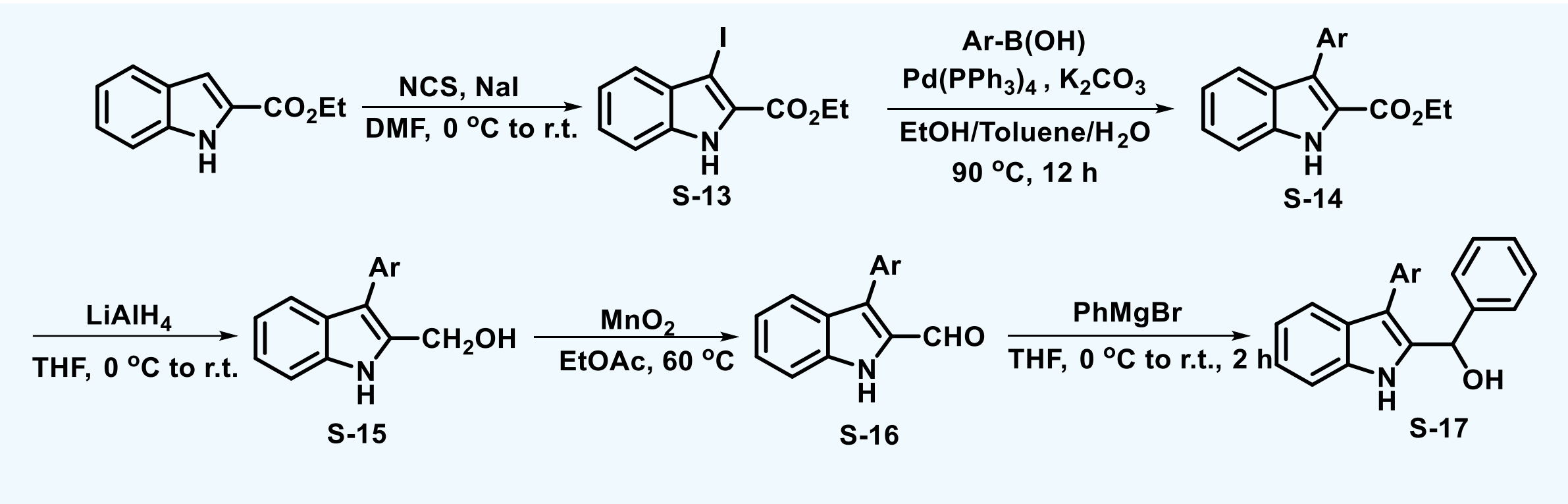

A mixture of ethyl 1*H*-indole-2-carboxylate (15 mmol, 1.0 equiv.), N-chlorosuccinimide (1.2 equiv.), and NaI (1.2 equiv.) in anhydrous DMF (15 mL) was stirred at 25 °C for 1 h under N_2 in amber glassware (monitored by TLC). The reaction was quenched with saturated $Na_2S_2O_3$ (aq.) at 0 °C and stirred for 15 min. The precipitate was collected by filtration, washed sequentially with cold H_2O and petroleum ether, and dried in vacuo to afford ethyl 3-iodo-1H-indole-2-carboxylate.

A flame-dried three-neck round-bottom flask equipped with a condenser and magnetic stir bar was charged with $Pd(PPh_3)_4$ (5 mol%) under argon. Anhydrous EtOH/toluene/H_2O (3:1:1, v/v, 40 mL) was added, followed by ethyl 3-iodo-1H-indole-2-carboxylate (8 mmol, 1.0 equiv.), Ar-$B(OH)_2$ (3.0 equiv.), and K_2CO_3 (3.0 equiv.). Degasse the mixture and heat it to reflux under nitrogen protection for 12 h (monitored by TLC). After cooling to 25 °C, the mixture was diluted with DCM and filtered through a pad of Celite® (washed with DCM). The filtrate was washed with brine, dried over anhydrous Na_2SO_4,

filtered, and concentrated under reduced pressure. Product **S-14** was purification by flash column chromatography.

A flame-dried, three-necked, round-bottom flask equipped with a magnetic stir bar was charged with **S-14** (8 mmol, 1.0 equiv.) dissolved in anhydrous THF (0.2 M) under N_2. A solution of $LiAlH_4$ (1.1 equiv.) in anhydrous THF (1.0 M) was added dropwise at 0 °C. The mixture was warmed to room temperature and stirred until TLC analysis indicated complete consumption of the starting material. The reaction mixture was recooled to 0 °C and carefully quenched by dropwise addition of 80% aqueous MeOH. The mixture was diluted with EtOAc and washed sequentially with 10 wt % $NaHSO_4$ (aq.) solution and brine. The organic layer was dried over Na_2SO_4, filtered, and concentrated under reduced pressure. The crude product **S-15** was used directly in the next step without further purification.

To a solution of **S-15** (5 mmol, 1.0 equiv.) in EtOAc (0.5 M) was added MnO_2 (10.0 equiv.) at room temperature. The slurry was stirred at 80 °C until complete consumption of **S-15** (monitored by TLC). After cooling to room temperature, the mixture was filtered through a pad of Celite® (washed with EtOAc). The filtrate was concentrated in vacuo to afford **S-16**.

A flame-dried Schlenk flask was charged with PhMgBr (2.0 equiv) in anhydrous THF (1.0 M) under N_2. A solution of **S-16** (3 mmol, 1.0 equiv.) in anhydrous THF (0.2 M) was added dropwise at 0 °C. The reaction mixture was warmed to room temperature and stirred for 2 h (monitored by TLC). After cooling to 0 °C, the reaction was carefully quenched by dropwise addition of saturated NH_4Cl (aq.). The aqueous layer was extracted with EtOAc. The combined organic extracts were washed with brine, dried over anhydrous Na_2SO_4, filtered, and concentrated under reduced pressure. Purification by flash column chromatography afforded substrates **2p-2t.**

Phenyl(3-phenyl-1*H*-indol-2-yl)methanol (2p):

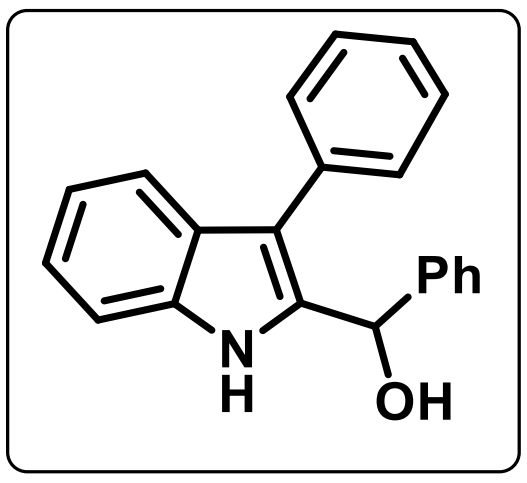

Following the general procedure (GP3), **2p** was isolated as a yellow solid (0.646 g, 72% yield). The physical data are matching with the reported value.

^{1}H NMR ((400 MHz, $CDCl_3$) δ (ppm)) δ 8.35 (s, 1H), 7.71 (ddt, *J* = 7.9, 1.3, 0.8 Hz, 1H), 7.54 – 7.39 (m, 4H), 7.39 – 7.26 (m, 7H), 7.25 – 7.11 (m, 2H), 6.16 (s, 1H).

^{13}C {^{1}H} NMR ((101 MHz, $CDCl_3$) δ (ppm)) δ 141.86, 135.71, 135.43, 134.47, 129.77, 128.78, 128.04, 126.67, 126.55, 126.25, 122.89, 120.32, 119.87, 116.25, 111.36, 68.46.

HRMS (ESI) Calcd. for $C_{21}H_{17}NO$ $[M\text{-}OH]^+$, 282.1277, found: 282.1283.

(3-(4-Methoxyphenyl)-1*H*-indol-2-yl)(phenyl)methanol (2q):

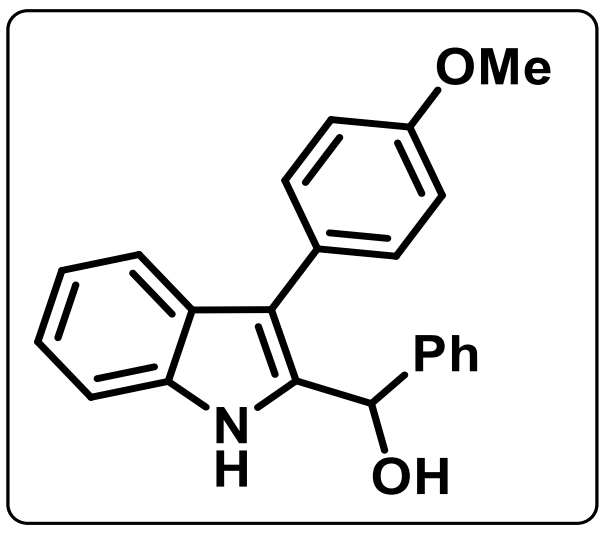

Following the general procedure (GP3), **2q** was isolated as a yellow solid (0.730 g, 74% yield). The physical data are matching with the reported value.

^{1}H NMR ((400 MHz, $CDCl_3$) δ (ppm)) δ 8.33 (s, 1H), 7.66 (ddt, *J* = 7.8, 1.3, 0.7 Hz, 1H), 7.47 – 7.38 (m, 2H), 7.37 – 7.32 (m, 3H), 7.31 – 7.26 (m, 2H), 7.21 (ddd, *J* = 8.1, 6.9, 1.3 Hz, 1H), 7.13 (ddd, *J* = 8.0, 6.9, 1.2 Hz, 1H), 6.13 (s, 1H), 3.85 (s, 3H).

^{13}C {^{1}H} NMR ((101 MHz, $CDCl_3$) δ (ppm)) δ 158.46, 141.95, 135.65, 135.16, 130.83, 128.76, 127.99, 127.70, 126.77, 126.53, 122.78, 120.16, 119.83, 115.82, 114.25, 111.32, 68.46, 55.44.

HRMS (ESI) Calcd. for $C_{22}H_{19}NO_2$ $[M\text{-}OH]^+$, 312.1383, found: 312.1388.

(3-(4-Fluorophenyl)-1*H*-indol-2-yl)(phenyl)methanol (2r):

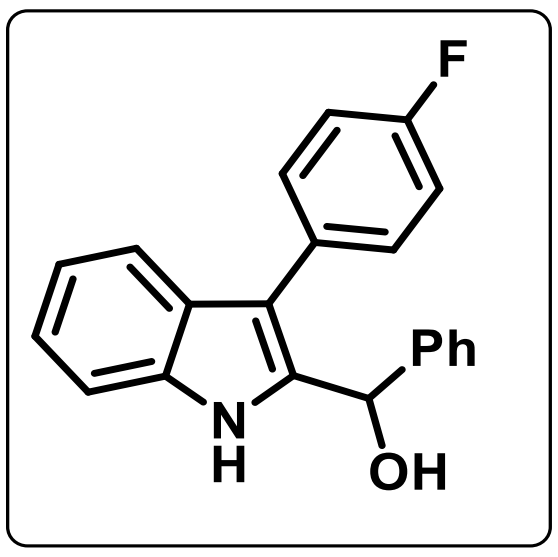

Following the general procedure (GP3), **2r** was isolated as a yellow liquid (0.770 g, 81% yield). The physical data are matching with the reported value.

^{1}H NMR ((400 MHz, $CDCl_3$) δ (ppm)) δ 8.47 (s, 1H), 7.69 – 7.58 (m, 1H), 7.44 – 7.37 (m, 2H), 7.31 (d, *J* = 1.1 Hz, 5H), 7.26 – 7.22 (m, 2H), 7.22 – 7.16 (m, 1H), 7.15 – 7.04 (m, 2H), 6.06 (s, 1H).

^{13}C {^{1}H} NMR ((101 MHz, $CDCl_3$) δ (ppm)) δ 161.86 (d, *J* = 245.2 Hz), 141.81, 135.60, 135.41, 131.25 (d, *J* = 7.8 Hz), 130.37, 128.84, 128.17, 127.54, 126.59, 126.27, 122.95, 120.00 (d, *J* = 83.5 Hz), 115.69 (d, *J* = 21.2 Hz), 115.06, 111.46, 68.58.

^{19}F NMR ((376 MHz, $CDCl_3$) δ (ppm)) δ -115.75.

HRMS (ESI) Calcd. for $C_{21}H_{16}NOF$ $[M\text{-}OH]^+$, 300.1183, found: 300.1208.

(3-(4-Chlorophenyl)-1*H*-indol-2-yl)(phenyl)methanol (2s):

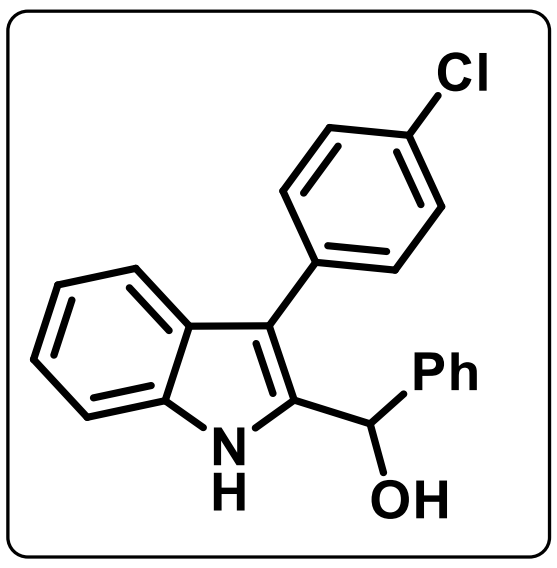

Following the general procedure (GP3), **2s** was isolated as a yellow liquid (0.789 g, 79% yield). The physical data are matching with the reported value.

^{1}H NMR ((400 MHz, $CDCl_3$) δ (ppm)) δ 8.53 (s, 1H), 7.70 (dd, *J* = 8.2, 1.7 Hz, 1H), 7.45 – 7.35 (m, 4H), 7.34 – 7.26 (m, 5H), 7.26 – 7.17 (m, 3H), 6.03 (s, 1H).

^{13}C {^{1}H} NMR ((101 MHz, $CDCl_3$) δ (ppm)) δ 141.67, 135.70, 135.48, 133.00, 132.51, 130.96, 129.03, 128.93, 128.29, 127.31, 126.66, 123.10, 120.62, 119.62, 114.90, 111.60, 68.64.

HRMS (ESI) Calcd. for $C_{21}H_{16}NOCl$ $[M-OH]^+$, 316.0888, found: 316.0891.

(3-(Naphthalen-2-yl)-1*H*-indol-2-yl)(phenyl)methanol (2t):

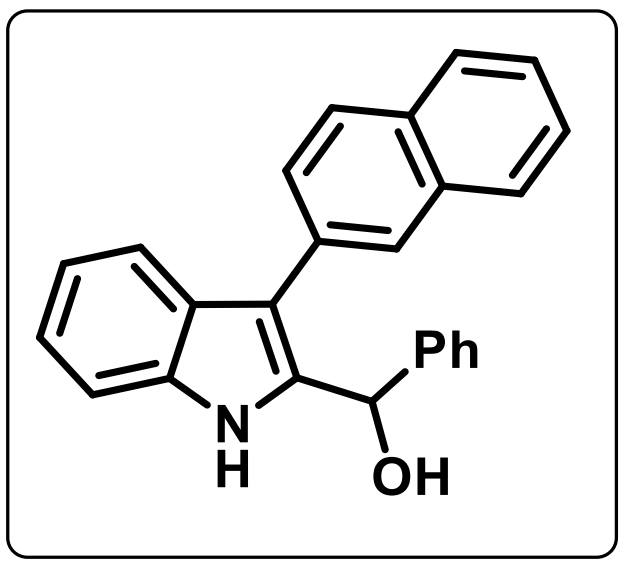

Following the general procedure (GP3), **2t** was isolated as a yellow solid (0.660 g, 63% yield). The physical data are matching with the reported value.

^{1}H NMR ((400 MHz, $CDCl_3$) δ (ppm)) δ 8.48 (s, 1H), 7.97 – 7.85 (m, 3H), 7.83 – 7.72 (m, 2H), 7.67 (dd, *J* = 8.4, 1.7 Hz, 1H), 7.57 – 7.46 (m, 2H), 7.41 – 7.36 (m, 2H), 7.36 – 7.28 (m, 4H), 7.28 – 7.22 (m, 2H), 7.17 (ddd, *J* = 8.1, 6.9, 1.2 Hz, 1H), 6.20 (s, 1H).

^{13}C {^{1}H} NMR ((101 MHz, $CDCl_3$) δ (ppm)) δ 142.02, 135.88, 135.72, 133.82, 132.31, 132.06, 128.83, 128.29, 128.27, 128.16, 128.10, 127.98, 127.86, 127.70, 126.63, 126.30, 125.81, 122.92, 120.43, 119.85, 115.98, 111.45, 68.68.

HRMS (ESI) Calcd. for $C_{25}H_{19}NO$ $[M-OH]^+$, 332.1434, found: 332.1442.

2.10. Synthesis of substrates 2u-2w: (General procedure-4)

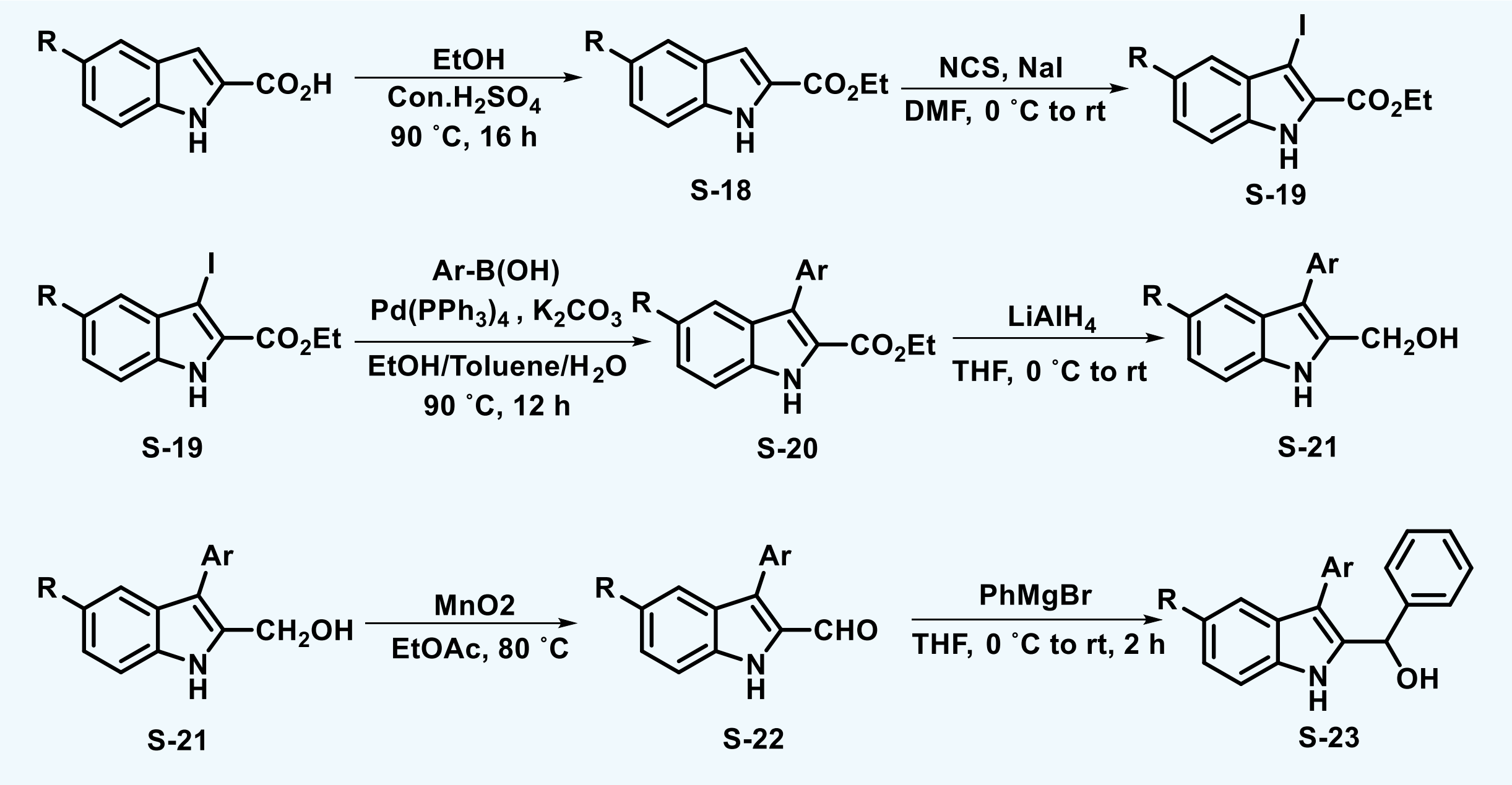

To the solution of indole-2-carboxylic acid (15.8 mmol, 1.0 equiv.) in EtOH (25 ml), was added con. H_2SO_4 (3 ml), and the mixture was stirred at 90 °C for 16 h. After completion of the reaction , the mixture was cooled to room temperature and the obtained solid was filtered, washed with water and dried under vacuum to give ethyl indole-2-carboxylate as a light brown solid .

A mixture of ethyl 1*H*-indole-2-carboxylate (15 mmol, 1.0 equiv.), N-chlorosuccinimide (1.2 equiv.), and NaI (1.2 equiv.) in anhydrous DMF (15 mL) was stirred at 25 °C for 1 h under N_2 in amber glassware (monitored by TLC). The reaction was quenched with saturated $Na_2S_2O_3$ (aq.) at 0 °C and stirred for 15 min. The precipitate was collected by filtration, washed sequentially with cold H_2O and petroleum ether, and dried in vacuo to afford ethyl 3-iodo-1*H*-indole-2-carboxylate.

A flame-dried three-neck round-bottom flask equipped with a condenser and magnetic stir bar was charged with $Pd(PPh_3)_4$ (5 mol%) under argon. Anhydrous EtOH/toluene/H_2O (3:1:1, v/v, 40 mL) was added, followed by ethyl 3-iodo-1*H*-indole-2-carboxylate (8 mmol, 1.0 equiv.), Ar-$B(OH)_2$ (3.0 equiv.), and K_2CO_3 (3.0 equiv.). Degasse the mixture and heat it to reflux under nitrogen protection for 12 h (monitored by TLC). After cooling to 25 °C, the mixture was diluted with DCM and filtered through a pad of Celite® (washed with DCM). The filtrate was washed with brine, dried over anhydrous Na_2SO_4, filtered, and concentrated under reduced pressure. Product **S-20** was purification by flash column chromatography.

A flame-dried, three-necked, round-bottom flask equipped with a magnetic stir bar was charged with **S-20** (8 mmol, 1.0 equiv.) dissolved in anhydrous THF (0.2 M) under N_2. A solution of $LiAlH_4$ (1.1 equiv.) in anhydrous THF (1.0 M) was added dropwise at 0 °C. The mixture was warmed to room temperature and stirred until TLC analysis indicated complete consumption of the starting material. The reaction

mixture was recooled to 0 °C and carefully quenched by dropwise addition of 80% aqueous MeOH. The mixture was diluted with EtOAc and washed sequentially with 10 wt % $NaHSO_4$ (aq.) solution and brine. The organic layer was dried over Na_2SO_4, filtered, and concentrated under reduced pressure. The crude product **S-21** was used directly in the next step without further purification.

To a solution of **S-21** (5 mmol, 1.0 equiv.) in EtOAc (0.5 M) was added MnO_2 (10.0 equiv.) at room temperature. The slurry was stirred at 80 °C until complete consumption of **S-21** (monitored by TLC). After cooling to room temperature, the mixture was filtered through a pad of Celite® (washed with EtOAc). The filtrate was concentrated in vacuo to afford **S-22**.

A flame-dried Schlenk flask was charged with PhMgBr (2.0 equiv) in anhydrous THF (1.0 M) under N_2. A solution of S-22 (3 mmol, 1.0 equiv.) in anhydrous THF (0.2 M) was added dropwise at 0 °C. The reaction mixture was warmed to room temperature and stirred for 2 h (monitored by TLC). After cooling to 0 °C, the reaction was carefully quenched by dropwise addition of saturated NH_4Cl (aq.). The aqueous layer was extracted with EtOAc. The combined organic extracts were washed with brine, dried over anhydrous Na_2SO_4, filtered, and concentrated under reduced pressure. Purification by flash column chromatography afforded substrates **2u-2w.**

(5-Methoxy-3-phenyl-1*H*-indol-2-yl)(phenyl)methanol (2u):

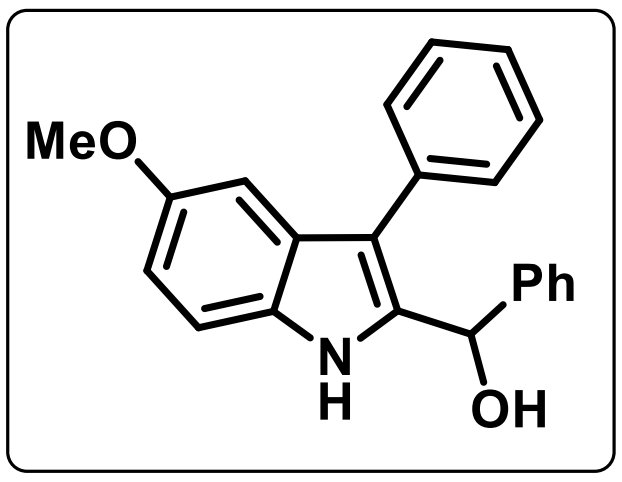

Following the general procedure (GP4), **2u** was isolated as a yellow solid (0.740 g, 75% yield).

^{1}H NMR ((400 MHz, $CDCl_3$) δ (ppm)) δ 8.23 (s, 1H), 7.53 – 7.39 (m, 4H), 7.37 – 7.28 (m, 6H), 7.17 (dd, *J* = 8.7, 0.6 Hz, 1H), 7.12 (dd, *J* = 2.6, 0.7 Hz, 1H), 6.86 (dd, *J* = 8.8, 2.4 Hz, 1H), 6.13 (s, 1H), 3.80 (s, 3H).

^{13}C {^{1}H} NMR ((101 MHz, $CDCl_3$) δ (ppm)) δ 154.67, 141.93, 136.31, 134.60, 130.77, 129.66, 128.83, 128.76, 128.01, 127.86, 126.59, 126.51, 116.03, 113.15, 112.10, 101.43, 68.45, 56.06.

HRMS (ESI) Calcd. for $C_{22}H_{19}NO_2$ $[M-OH]^+$, 312.1383, found: 312.1397.

(5-Fluoro-3-phenyl-1*H*-indol-2-yl)(phenyl)methanol (2v):

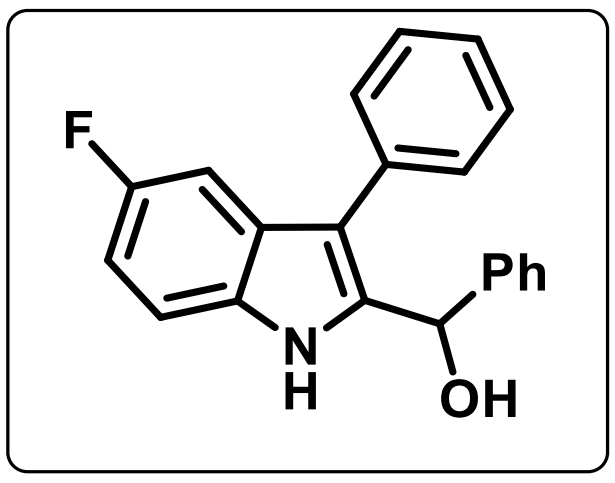

Following the general procedure (GP4), **2v** was isolated as a yellow solid (0.780 g, 82% yield).

^{1}H NMR ((400 MHz, $CDCl_3$) δ (ppm)) δ 8.48 (s, 1H), 7.44 (d, *J* = 5.4 Hz, 2H), 7.40 – 7.29 (m, 7H), 7.28 – 7.20 (m, 1H), 7.16 – 7.02 (m, 1H), 7.01 – 6.88 (m, 1H), 6.12 (d, *J* = 8.6 Hz, 1H), 3.10 (s, 1H).

^{13}C {^{1}H} NMR ((101 MHz, $CDCl_3$) δ (ppm)) δ 158.47 (d, *J* = 234.9 Hz), 141.64, 140.69, 137.12, 133.13 (d, *J* = 184.2 Hz), 129.57, 128.93, 128.23, 126.58, 126.29, 116.34 (d, *J* = 4.8 Hz), 112.61 (d, *J* = 9.5 Hz), 112.00 (d, *J* = 10.4 Hz), 111.26 (d, *J* = 26.2 Hz), 104.62, 103.41, 68.57.

^{19}F NMR ((376 MHz, $CDCl_3$) δ (ppm)) δ -123.02 (dd, *J* = 347.9, 4.3 Hz).

HRMS (ESI) Calcd. for $C_{21}H_{16}NOF$ $[M-OH]^+$, 300.1183, found: 300.1207

(5-Bromo-3-phenyl-1H-indol-2-yl)(phenyl)methanol (2w):

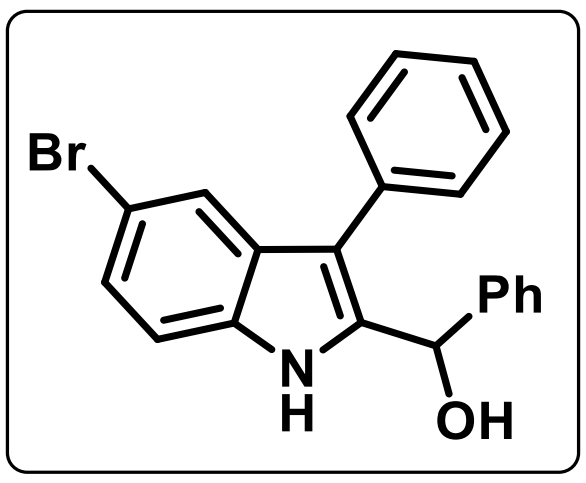

Following the general procedure (GP4), **2w** was isolated as a yellow solid (0.871 g, 77% yield).

^{1}H NMR (((400 MHz, $CDCl_3$) δ (ppm)) δ 8.62 (s, 1H), 7.44 (dddd, *J* = 7.4, 3.6, 1.8, 1.0 Hz, 4H), 7.39 – 7.28 (m, 4H), 7.24 – 7.14 (m, 4H), 6.14 (s, 1H).

^{13}C {^{1}H} NMR ((101 MHz, $CDCl_3$) δ (ppm)) δ 140.90, 140.00, 135.88, 130.50, 128.87, 128.86, 128.32, 128.30, 126.45, 126.24, 123.50, 121.07, 120.93, 120.62, 118.27, 111.62, 70.57.

HRMS (ESI) Calcd. for $C_{21}H_{16}NOBr$ $[M-OH]^+$, 360.0382, found: 360.0376.

2.11. (1-Methyl-1*H*-indol-3-yl)methanol (2x)

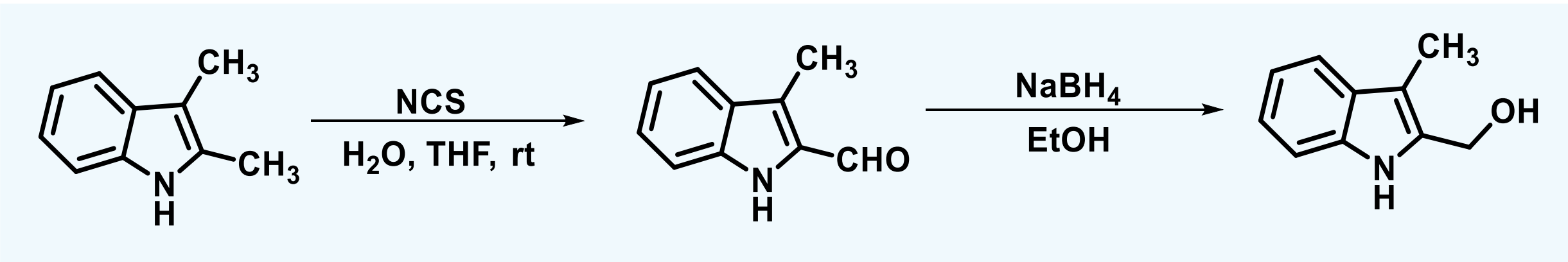

To the solution of 2,3-dimethylindole(7.0 mmol, 1.0 equiv.) in THF (35 mL) and H_2O (126.0 µL, 7.0 mmol, 1.0 equiv.) was added NCS (14.7 mmol, 1.96 g, 2.1 equiv.), and then stirred at room temperature. After the completion of the reaction, as indicated by TLC, the reaction was quenched with water. The resulting mixture was extracted with ethyl acetate and the combined organic layers were dried over Na_2SO_4 and evaporated under reduced pressure. The residue was purified by column chromatography (hexane/ethyl acetate = 50:1) on silica gel to afford desired product (858.0 mg, 77% yield) .

In a round bottom flask, 1-methyl-1*H*-indole-3-carbaldehyde (0.318 g, 2.0 mmol) was dissolved in 20 mL ethanol. The reaction mixture was cooled to 0 °C in an ice/water bath and $NaBH_4$ (0.189 g, 2.5 equiv) was added slowly. The reaction was stirred for 1 h at 0 °C. After completion, the reaction was quenched by adding H_2O and excess ethanol was evaporated in rota. Then, the aqueous layer was extracted with EtOAc and the combined organic layers were dried with Na_2SO_4, filtered, and concentrated in vacuum followed by the purification using silica gel flash column chromatography (hexane/ethyl acetate = 90:10 as eluent) to afford **2x** as yellow sticky liquid (0.316 g, 98% yield).

^{1}H NMR (399 MHz, DMSO-D_6) δ 10.68 (s, 1H), 7.38 (ddt, J = 7.8, 1.4, 0.7 Hz, 1H), 7.29 – 7.21 (m, 1H), 6.99 (ddd, J = 8.1, 7.0, 1.2 Hz, 1H), 6.90 (ddd, J = 7.9, 7.0, 1.1 Hz, 1H), 4.55 (d, J = 5.4 Hz, 2H), 2.17 (s, 3H).

^{13}C {^{1}H} NMR (101 MHz, DMSO-D_6) δ 135.91, 135.76, 129.02, 121.21, 118.54, 118.52, 111.41, 106.19, 55.19, 8.80.

HRMS (ESI) Calcd. for $C_{10}H_{11}NO$ $[M-OH]^+$, 144.0808, found: 144.0816.

2.12. (1-Methyl-1*H*-indol-3-yl)methanol (2y)

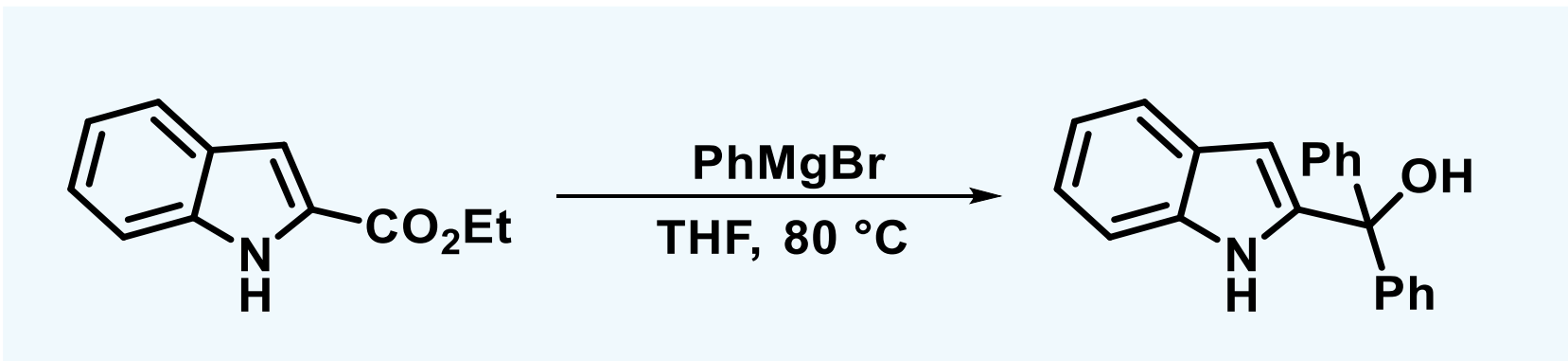

Under N_2 atmosphere, phenylmagnesium bromide (10 mL, 3 mol/mL) was added to a Schlenk bottle. Then, the solution of ethyl 1*H*-indole-2-carboxylate S1 (5 mmol) in anhydrous THF (1 mol/mL) was added dropwise to the Schlenk bottle at 0 °C. Subsequently, the reaction mixture was stirred at 80 °C overnight. After the completion of the reaction indicated by TLC, the reaction mixture was quenched by saturated ammonium chloride solution and extracted by ethyl acetate for three times. The combined organic layers were dried and concentrated under reduced pressure to give a residue. Finally, the residue was purified by flash column chromatography on silica gel to afford pure 2-indolylmethanols (Hexane/ EtOAc 90:10 as eluent) to afford **2y** as yellow sticky liquid (1.420 g, 95% yield).

^{1}H NMR (399 MHz, DMSO-D_6) δ 10.68 (s, 1H), 7.38 (ddt, *J* = 7.8, 1.4, 0.7 Hz, 1H), 7.29 – 7.21 (m, 1H), 6.99 (ddd, *J* = 8.1, 7.0, 1.2 Hz, 1H), 6.90 (ddd, *J* = 7.9, 7.0, 1.1 Hz, 1H), 5.05 – 4.98 (m, 1H), 4.55 (d, *J* = 5.4 Hz, 2H), 2.17 (s, 3H).

^{13}C {^{1}H} NMR (101 MHz, DMSO-D_6) δ 135.91, 135.76, 129.02, 121.21, 118.54, 118.52, 111.41, 106.19, 55.19, 8.80.

HRMS (ESI) Calcd. for $C_{21}H_{17}NO$ $[M+H]^+$, 300.1383, found: 300.1389.

3. Reaction Optimization:

3.1. Catalyst optimization:

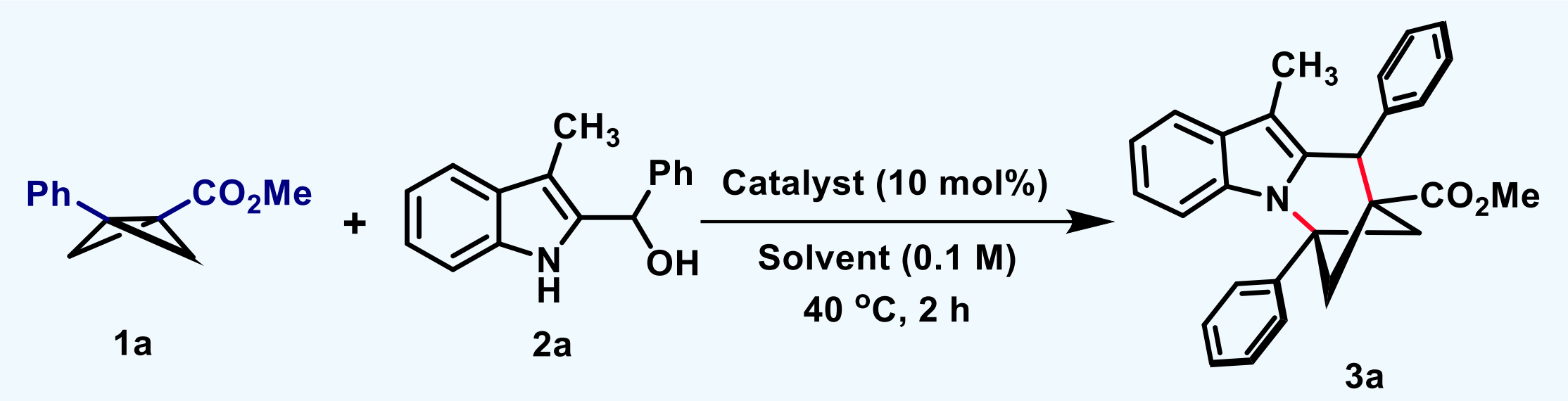

An oven-dried 10 mL Schlenk flask equipped with a stir bar was cooled under vacuum. (3-methyl-1*H*-indol-2-yl)(phenyl)methanol **2a** (23.7 mg, 0.1 mmol, 1.0 equiv.), and Catalyst (0.01 mmol, 0.10 equiv.) were loaded in the Schlenk flask. Methyl 3-phenylbicyclo[1.1.0]butane-1-carboxylate (BCB) **1a** (28.2 mg, 0.15 mmol, 1.5 equiv.) and Solvent (0.1 M) was added at the mentioned temperature. The reaction was stirred for the 2 h. then the solvent was evaporated, and the crude mixture was passed through a pad of silica gel and eluted with DCM (3x10 mL). The reaction mixture was concentrated under reduced pressure and then the yield of **3a** was determined by the ^1H NMR analysis of the crude reaction products using trichloroethylene (TCE) as the internal standard.

Table S1:

Entry	Catalyst	Solvent	Yield (%)[a]
1	$Sc(OTf)_3$	DCM	40
2	$Sn(OTf)_2$	DCM	41
3	AgOTf	DCM	34
4	$Yb(OTf)_3$	DCM	33
5	$Ni(OTf)_2$	DCM	32
6	$Co(OTf)_2$	DCM	56
7	$Mg(OTf)_2$	DCM	25
8	$Cu(OTf)_2$	DCM	58
9	$Zn(OTf)_2$	DCM	54
10	$Bi(OTf)_3$	DCM	33
11	*p*TSA	DCM	36
12	TMSOTf	DCM	<5
13	Pentafluoro phenol	DCM	39
14	Pentafluorophenyl boronic acid	DCM	0
15	No catalyst	HFIP	<5

Reaction conditions: 1a (0.15 mmol), 2a (0.1 mmol), catalyst (0.01 mmol), solvent (0.1 M, 1 mL) for 2 h. The ^{1}H *NMR* yield of the crude products was determined using 1,1,2-trichloroethylene as the internal standard

3.2. Solvent optimization:

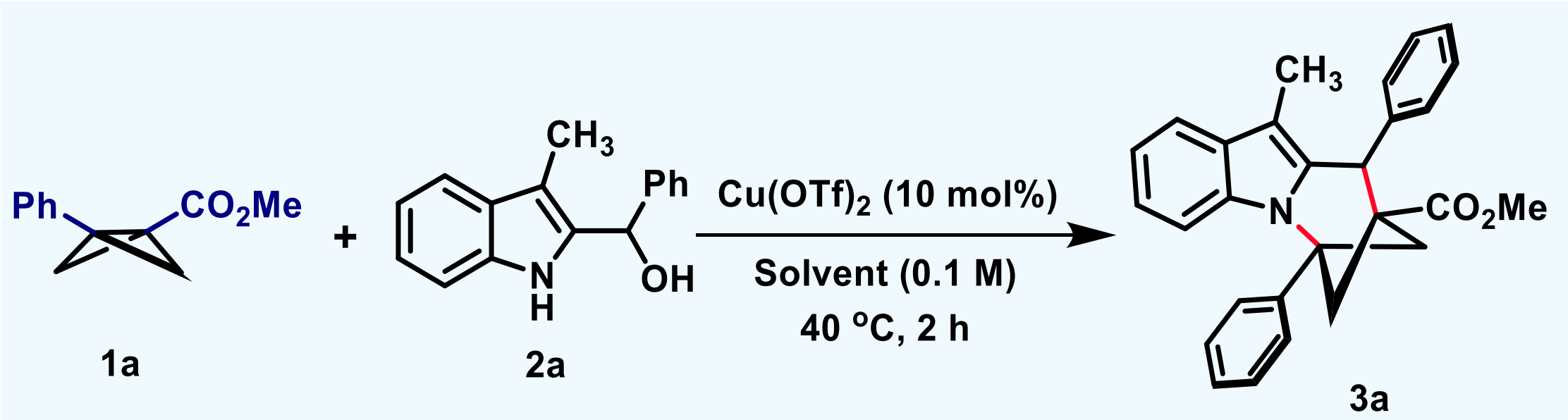

An oven-dried 10 mL Schlenk flask equipped with a stir bar was cooled under vacuum. (3-methyl-1*H*-indol-2-yl)(phenyl)methanol **2a** (23.7 mg, 0.1 mmol, 1.0 equiv.), and $Cu(OTf)_2$ (0.01 mmol, 0.10 equiv.) were loaded in the Schlenk flask. Methyl 3-phenylbicyclo[1.1.0]butane-1-carboxylate (BCB) **1a** (28.2 mg, 0.15 mmol, 1.5 equiv.) and Solvent (0.1 M) was added at the mentioned temperature. The reaction was stirred for the 2 h. then the solvent was evaporated, and the crude mixture was passed through a pad of silica gel and eluted with DCM (3x10 mL). The reaction mixture was concentrated under reduced pressure and then the yield of **3a** was determined by the ^{1}H NMR analysis of the crude reaction products using trichloroethylene (TCE) as the internal standard.

Table S2:

Entry	Catalyst	Solvent	Yield (%)
1	$Cu(OTf)_2$	DCM	58
2	$Cu(OTf)_2$	CH_3CN	64
3	$Cu(OTf)_2$	1,4-dioxane	56
4	$Cu(OTf)_2$	HFIP	<5
5	$Cu(OTf)_2$	DCE	49
6	$Cu(OTf)_2$	$CHCl_3$	68
7	$Cu(OTf)_2$	DCM + 1,4-dioxane (1:1)	41
8	$Cu(OTf)_2$	CH_3CN + 1,4-dioxane (1:1)	44
9	$Cu(OTf)_2$	DCM + HFIP (1:1)	36

Reaction conditions: 1a (0.15 mmol), 2a (0.1 mmol), $Cu(OTf)_2$ (0.01 mmol), solvent (0.1 M, 1 mL) for 2 h. The ^{1}H *NMR* yield of the crude products was determined using 1,1,2-trichloroethylene as the internal standard

3.3. Temperature optimization:

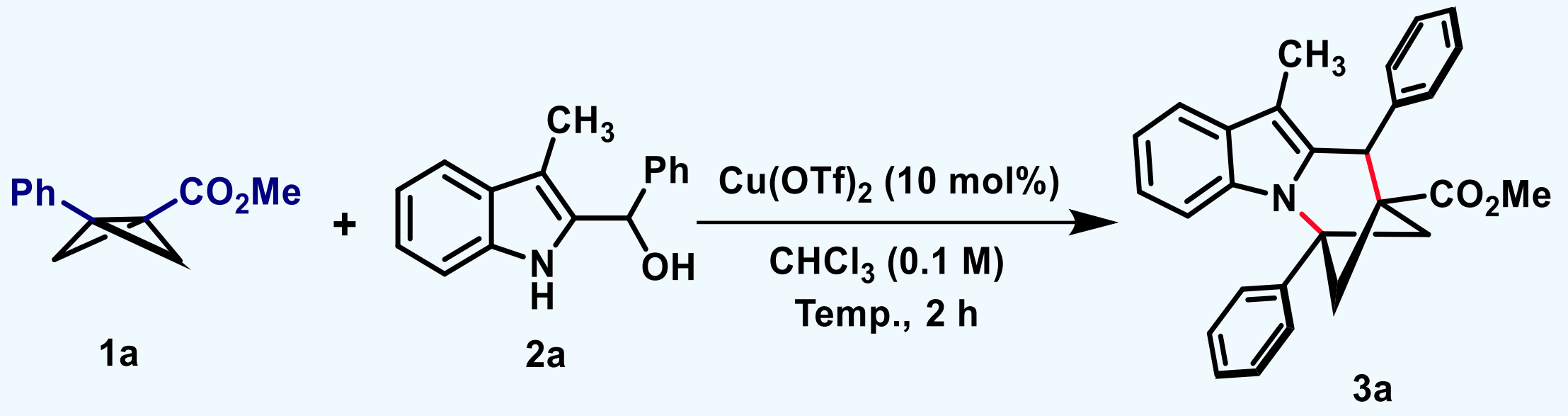

An oven-dried 10 mL Schlenk flask equipped with a stir bar was cooled under vacuum. (3-methyl-1*H*-indol-2-yl)(phenyl)methanol **2a** (23.7 mg, 0.1 mmol, 1.0 equiv.), and $Cu(OTf)_2$ (0.01 mmol, 0.10 equiv.) were loaded in the Schlenk flask. Methyl 3-phenylbicyclo[1.1.0]butane-1-carboxylate (BCB) **1a** (28.2 mg, 0.15 mmol, 1.5 equiv.) and $CHCl_3$ (0.1 M) was added at the mentioned temperature. The reaction was stirred for the 2 h. then the solvent was evaporated, and the crude mixture was passed through a pad of silica gel and eluted with DCM (3x10 mL). The reaction mixture was concentrated under reduced pressure and then the yield of **3a** was determined by the ^{1}H NMR analysis of the crude reaction products using trichloroethylene (TCE) as the internal standard.

Table S3:

Entry	Catalyst	Temperature (°C)	Yield (%)
1	$Cu(OTf)_2$	0 to 25	45
2	$Cu(OTf)_2$	40	68
3	$Cu(OTf)_2$	60	86
4	$Cu(OTf)_2$	80	84

Reaction conditions: 1a (0.15 mmol), 2a (0.1 mmol), $Cu(OTf)_2$ (0.01 mmol), $CHCl_3$ (0.1 M, 1 mL) for 2 h. The ^{1}*H NMR* yield of the crude products was determined using 1,1,2-trichloroethylene as the internal standard

3.4. Concentration optimization:

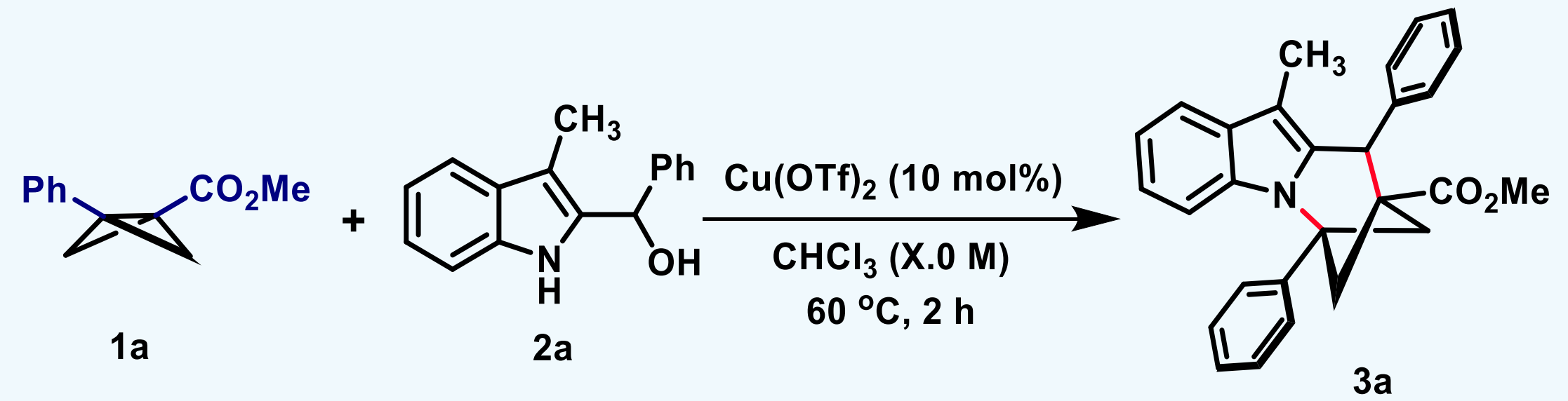

An oven-dried 10 mL Schlenk flask equipped with a stir bar was cooled under vacuum. (3-methyl-1*H*-indol-2-yl)(phenyl)methanol **2a** (23.7 mg, 0.1 mmol, 1.0 equiv.), and $Cu(OTf)_2$ (0.01 mmol, 0.10 equiv.)

were loaded in the Schlenk flask. Methyl 3-phenylbicyclo[1.1.0]butane-1-carboxylate (BCB) **1a** (28.2 mg, 0.15 mmol, 1.5 equiv.) and $CHCl_3$ (different concentrations) was added at 60 ºC. The reaction was stirred for the 2 h. then the solvent was evaporated, and the crude mixture was passed through a pad of silica gel and eluted with DCM (3x10 mL). The reaction mixture was concentrated under reduced pressure and then the yield of **3a** was determined by the ^{1}H NMR analysis of the crude reaction products using trichloroethylene (TCE) as the internal standard.

Table S4:

Entry	Solvent	Concentration (M)	Yield (%)
1	$CHCl_3$	0.05	84
2	$CHCl_3$	0.1	86
3	$CHCl_3$	0.2	64

Reaction conditions: 1a (0.15 mmol), 2a (0.1 mmol), $Cu(OTf)_2$ (0.01 mmol), $CHCl_3$ (0.X M) for 2 h. The ^{1}*H NMR* yield of the crude products was determined using 1,1,2-trichloroethylene as the internal standard

3.5. Equivalence optimization:

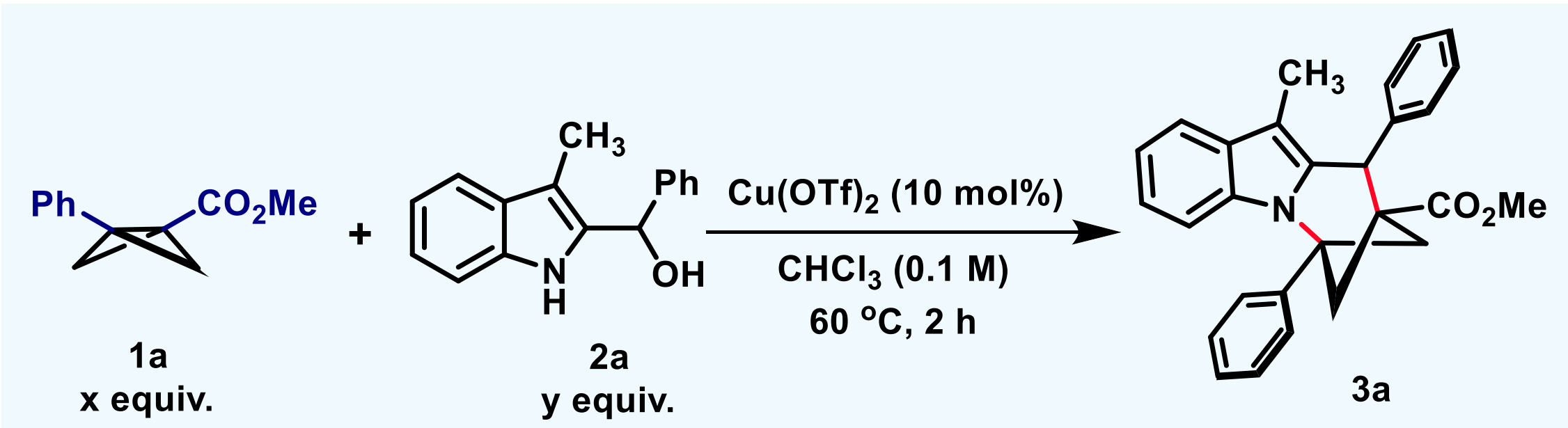

An oven-dried 10 mL Schlenk flask equipped with a stir bar was cooled under vacuum. (3-methyl-1*H*-indol-2-yl)(phenyl)methanol **2a** (y equiv.), and $Cu(OTf)_2$ (0.01 mmol, 0.10 equiv.) were loaded in the Schlenk flask. Methyl 3-phenylbicyclo[1.1.0]butane-1-carboxylate (BCB) **1a** (x equiv.) and $CHCl_3$ (0.1 M) was added at 60 ºC. The reaction was stirred for the 2 h. then the solvent was evaporated, and the crude mixture was passed through a pad of silica gel and eluted with DCM (3x10 mL). The reaction mixture was concentrated under reduced pressure and then the yield of **3a** was determined by the ^{1}H NMR analysis of the crude reaction products using trichloroethylene (TCE) as the internal standard.

Table S5:

Entry	BCB (equiv.)	Indolyl Methanol (equiv.)	Yield (%)
1	1	1	58
2	1.2	1	72
3	1.5	1	86
4	1	1.5	75

Reaction conditions: 1a (0.X mmol), 2a (0.Y mmol), $Cu(OTf)_2$ (0.01 mmol), $CHCl_3$ (0.1 M, 1 mL) for 2 h. The *^1H NMR* yield of the crude products was determined using 1,1,2-trichloroethylene as the internal standard

4. Sensitivity screen.

Recently, Glorius group has established a reaction-condition-based assignment to evaluate the sensitivity of a chemical reaction.[8] The sensitivity screen conducted in this study is based on a modification of the literature procedure.

Standard conditions:

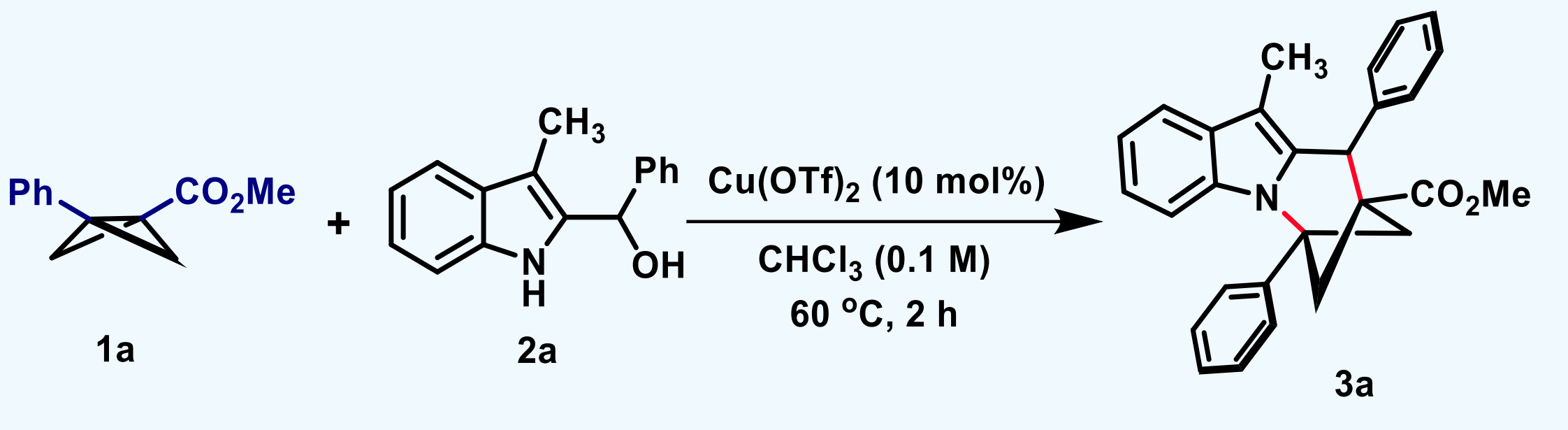

An oven-dried 10 mL Schlenk flask equipped with a stir bar was cooled under vacuum. (3-methyl-1*H*-indol-2-yl)(phenyl)methanol **2a** (23.7 mg, 0.1 mmol, 1.0 equiv.), and $Cu(OTf)_2$ (0.01 mmol, 0.10 equiv.) were loaded in the Schlenk flask. Methyl 3-phenylbicyclo[1.1.0]butane-1-carboxylate (BCB) **1a** (28.2 mg, 0.15 mmol, 1.5 equiv.) and $CHCl_3$ (0.1 M) was added at 60 ºC. The reaction was stirred for the 2 h. then the solvent was evaporated, and the crude mixture was passed through a pad of silica gel and eluted with DCM (3x10 mL). The reaction mixture was concentrated under reduced pressure and then the yield of **3a** was determined by the ^1H NMR analysis of the crude reaction products using trichloroethylene (TCE) as the internal standard.

Table S6:

Entry	Modification	Deviation form standard condition	Yield	Deviation
**1**	Standard		86%	0
**2**	Low Catalyst loading	(5mol%)	62%	-24
**3**	H_2O	+ 10μl H_2O	52%	-34
**4**	Air	+ 2ml air	45%	-41
**5**	Low *T*	40˚C	67%	-19
**6**	High *T*	80˚C	84%	-2
**7**	High *C*	0.2M	64%	-22
**8**	Low *C*	0.05M	84%	-2
**9**	Big Scale	Standard scale × 32	78%	-8

Reaction conditions: 1a (0.15 mmol), 2a (0.1 mmol), $Cu(OTf)_2$ (0.01 mmol), $CHCl_3$ (0.1 M, 1 mL) for 2 h. The *¹H NMR* yield of the crude products was determined using 1,1,2-trichloroethylene as the internal standard

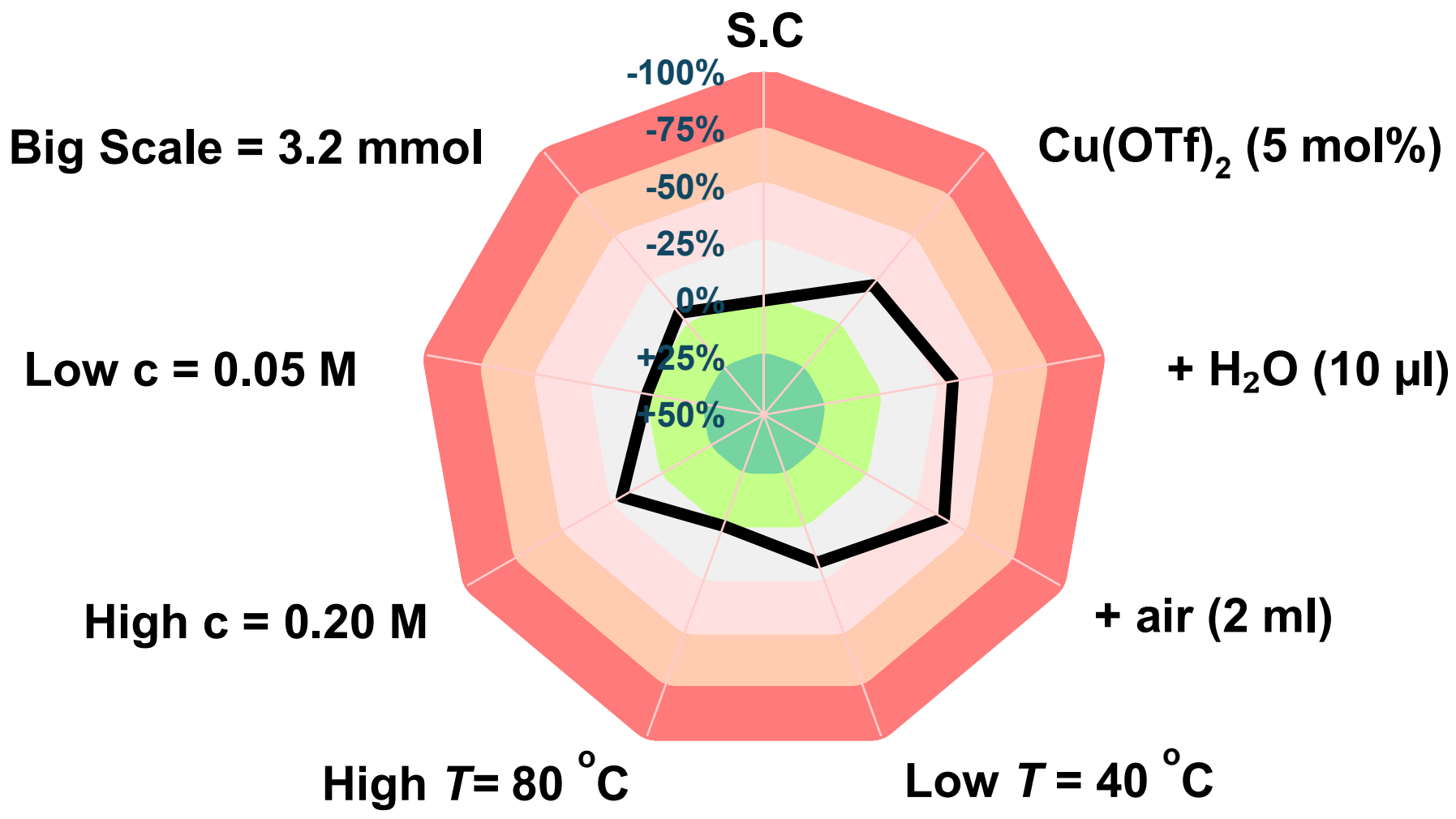

The sensitivity study indicates that the optimized condition is robust under most conditions. However, in the presence of H_2O and air, some deviation is observed.

5. General procedure (GP-5) for the formal [3+3]-annulation of bicyclo[1.1.0]butanes with indolyl methanol derivatives:

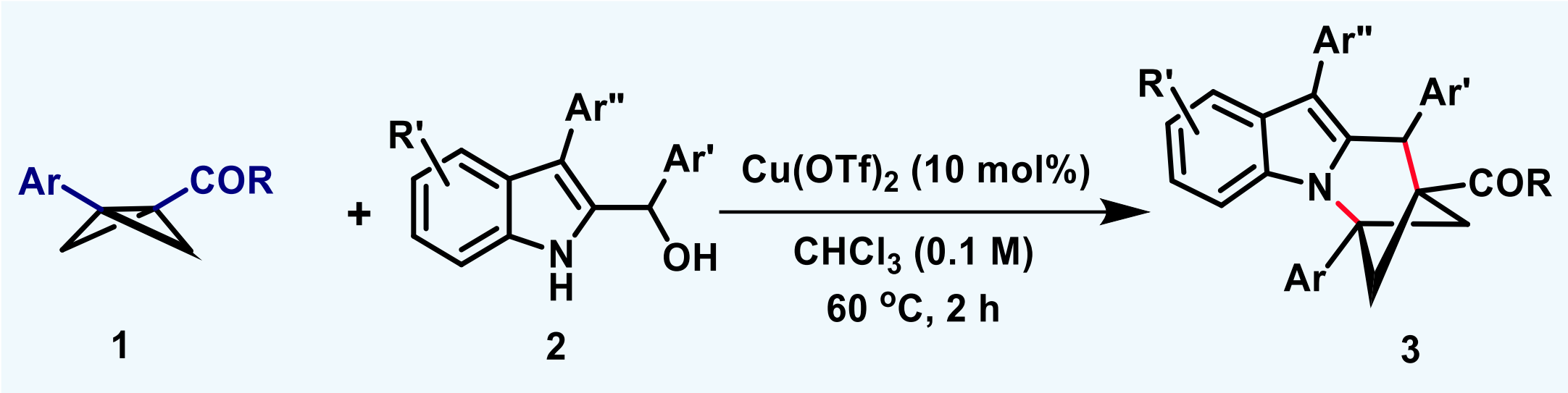

An oven-dried 10 mL Schlenk flask equipped with a stir bar was cooled under vacuum. Indolyl methanol derivative **2** (0.2 mmol, 1.0 equiv.), and $Cu(OTf)_2$ (0.02 mmol, 0.10 equiv.) were loaded in the Schlenk flask. BCB derivative **1** (0.3 mmol, 1.5 equiv.) and $CHCl_3$ (0.1 M) was added at 60 ºC. The reaction was stirred for the 2 h. then reaction mixture was diluted with 10 mL CH_2Cl_2. The crude residue was purified by column chromatography on silica gel.

Methyl-10-methyl-6,9-diphenyl-6,7-dihydro-6,8-methanopyrido[1,2-a]indole-8(9*H*)-carboxylate (3a):

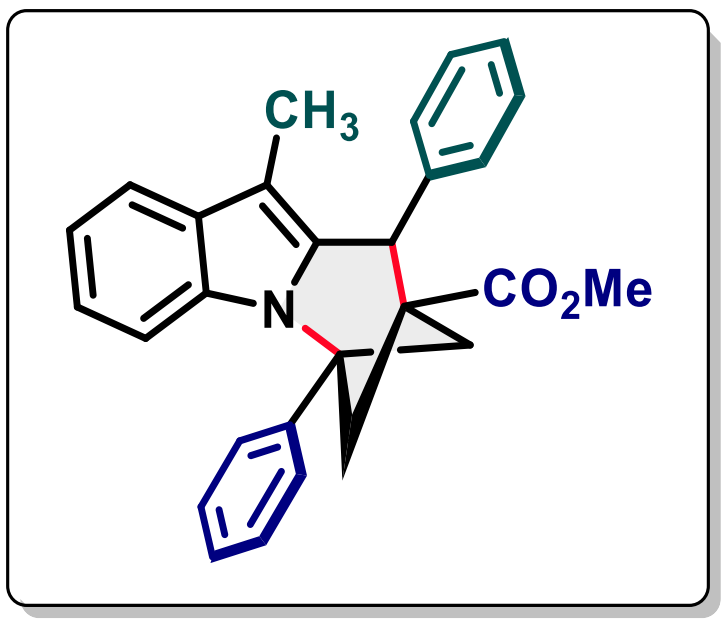

The reaction was performed according to the general procedure GP5 with methyl 3-phenylbicyclo[1.1.0]butane-1-carboxylate **1a** (56.4 mg, 0.3 mmol, 1.5 equiv.) and (3-methyl-1*H*-indol-2-yl)(phenyl)methanol **2a** (47.4 mg, 0.2 mmol, 1.0 equiv.) at 60 ºC for 2 h. After purification by flash column chromatography (hexane/ethyl acetate = 9.8/0.2), the desired compound **3a** was obtained as a White solid (67.6 mg, 83% yield).

Rf = 0.52 (hexane/ethyl acetate = 9/1).

M.P. = 186 – 189 ºC

^{1}H NMR ((400 MHz, $CDCl_3$) δ (ppm)) δ 7.66 – 7.40 (m, 3H), 7.39 – 7.22 (m, 3H), 6.96 (ddd, J = 7.9, 7.0, 1.0 Hz, 1H), 6.72 (ddd, J = 8.4, 7.0, 1.3 Hz, 1H), 5.34 (d, J = 8.6 Hz, 1H), 5.20 (s, 1H), 3.68 (s, 2H), 3.01 – 2.88 (m, 2H), 2.81 (dd, J = 9.6, 7.1 Hz, 1H), 1.89 (s, 1H).

^{13}C {^{1}H} NMR ((101 MHz, $CDCl_3$) δ (ppm)) δ 173.86, 140.22, 134.92, 133.34, 129.30, 129.13, 129.00, 128.93, 128.81, 128.54, 128.07, 128.02, 127.45, 120.85, 118.25, 111.33, 108.84, 62.32, 51.94, 48.36, 47.74, 47.32, 39.42, 8.71.

HRMS (ESI) Calcd. for $C_{28}H_{25}NO_2$ $[M+H]^+$, 408.1958, found: 408.1957.

FTIR (cm^{-1}) : 1100, 1214, 1331, 1451, 1741, 2924.

Methyl-10-methyl-6-phenyl-9-(*p*-tolyl)-6,7-dihydro-6,8-methanopyrido[1,2-a]indole-8(9*H*)-carboxylate (3b):

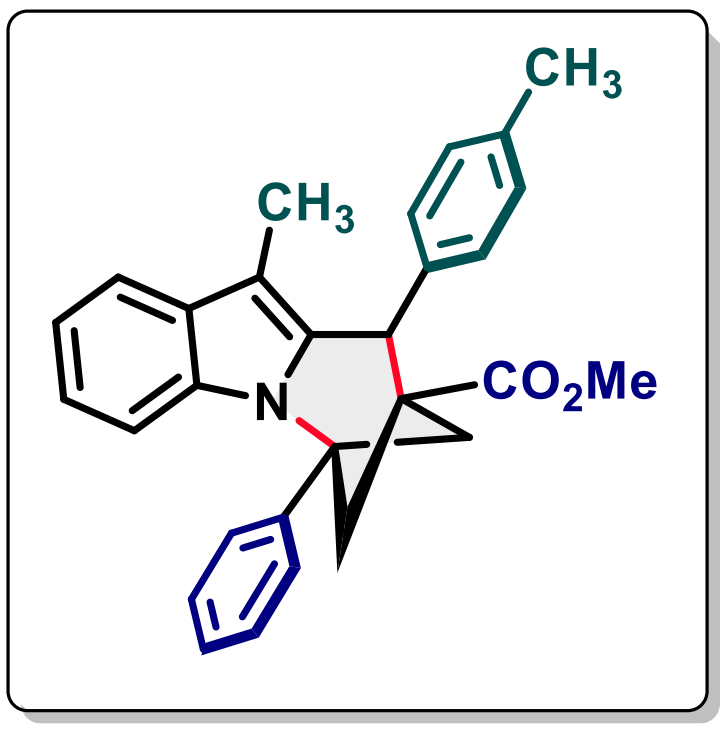

The reaction was performed according to the general procedure GP5 with methyl 3-phenylbicyclo[1.1.0]butane-1-carboxylate **1a** (56.4 mg, 0.3 mmol, 1.5 equiv.) and (3-methyl-1*H*-indol-2-yl)(p-tolyl)methanol **2b** (50.2 mg, 0.2 mmol, 1.00 equiv.) at 60 ºC for 2 h. After purification by flash column chromatography (hexane/ethyl acetate = 9.85/0.15), the desired compound **3b** was obtained as a white solid (64.9 mg, 77% yield).

Rf = 0.55 (hexane/ethyl acetate = 9/1).

M.P. = 128 – 130 ºC

^{1}H NMR ((400 MHz, $CDCl_3$) δ (ppm)) δ 7.64 – 7.35 (m, 7H), 7.11 (s, 4H), 6.93 (ddd, J = 7.9, 7.0, 0.9 Hz, 1H), 6.68 (ddd, J = 8.5, 7.0, 1.3 Hz, 1H), 5.30 (d, J = 8.6 Hz, 1H), 5.14 (s, 1H), 3.67 (s, 3H), 2.95 – 2.85 (m, 3H), 2.80 – 2.73 (m, 1H), 2.35 (s, 3H), 1.87 (d, J = 0.9 Hz, 3H).

^{13}C {^{1}H} NMR ((100 MHz, $CDCl_3$) δ (ppm)) δ 173.92, 140.26, 137.11, 136.93, 134.88, 133.51, 129.30, 129.21, 129.06, 128.95, 128.88, 128.60, 128.04, 128.00, 120.74, 118.20, 118.18, 111.29, 108.74, 62.29, 51.88, 48.31, 47.34, 47.22, 39.40, 21.22, 8.69.

HRMS (ESI) Calcd. for $C_{29}H_{27}NO_2$ $[M+H]^+$, 422.2115, found: 422.2118.

FTIR (cm^{-1}) : 1097, 1215, 1331, 1450, 1738, 2925.

Methyl-9-(4-(*tert*-butyl)phenyl)-10-methyl-6-phenyl-6,7-dihydro-6,8-methanopyrido-[1,2-a]indole-8(9*H*)-carboxylate (3c):

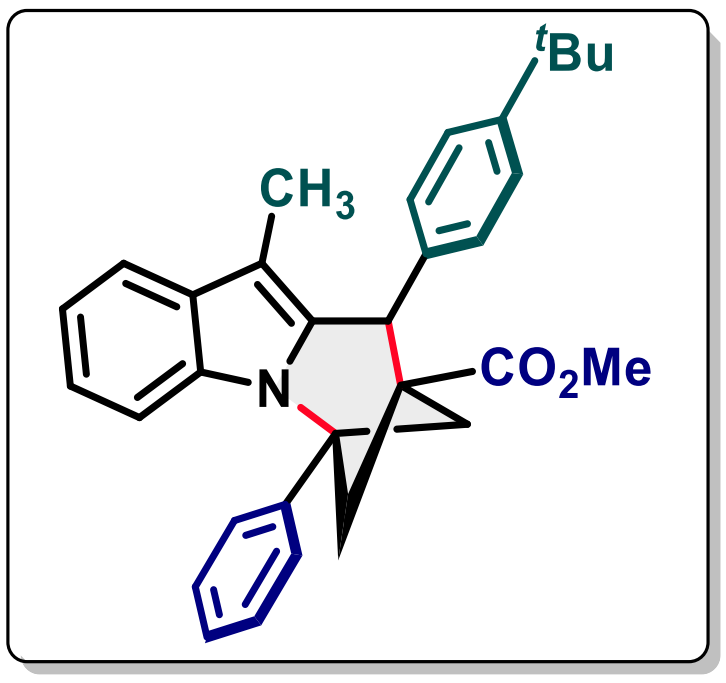

The reaction was performed according to the general procedure GP5 with methyl 3-phenylbicyclo[1.1.0]butane-1-carboxylate **1a** (56.4 mg, 0.3 mmol, 1.5 equiv.) and (4-(*tert*-butyl)phenyl)(3-methyl-1*H*-indol-2-yl)methanol **2c** (58.6 mg, 0.2 mmol, 1.0 equiv.) at 60 °C ˚C for 2 h. After purification by flash column chromatography (hexane/ethyl acetate = 9.85/0.15), the desired compound **3c** was obtained as a White solid (69.5 mg, 75% yield).

Rf = 0.6 (hexane/ethyl acetate = 9/1).

M.P. = 181 – 183 °C

^{1}H NMR ((400 MHz, $CDCl_3$) δ (ppm)) δ 7.60 – 7.44 (m, 4H), 7.44 – 7.36 (m, 2H), 7.31 – 7.22 (m, 2H), 7.13 – 7.07 (m, 2H), 6.90 (ddd, *J* = 8.0, 7.0, 0.9 Hz, 1H), 6.65 (ddd, *J* = 8.5, 7.1, 1.3 Hz, 1H), 5.31 – 5.23 (m, 1H), 5.11 (s, 1H), 3.64 (s, 3H), 2.94 – 2.81 (m, 3H), 2.74 (dd, *J* = 9.5, 7.1 Hz, 1H), 1.83 (d, *J* = 0.9 Hz, 3H), 1.30 (s, 9H).

^{13}C {^{1}H} NMR ((101 MHz, $CDCl_3$) δ (ppm)) δ 173.91, 150.04, 140.27, 136.93, 134.85, 133.60, 129.30, 129.03, 128.93, 128.84, 128.32, 128.01, 127.98, 125.25, 120.64, 118.18, 118.11, 111.24, 108.75, 62.28, 51.83, 48.26, 47.20, 39.44, 34.52, 31.46, 8.66.

HRMS (ESI) Calcd. for $C_{32}H_{33}NO_2$ $[M+H]^+$, 464.2584, found: 464.2576.

FTIR (cm^{-1}) : 1104, 1226, 1252, 1332, 1452, 1718, 2957.

Methyl-10-methyl-9-(naphthalen-2-yl)-6-phenyl-6,7-dihydro-6,8-methanopyrido-[1,2-a]indole-8(9*H*)-carboxylate (3d):

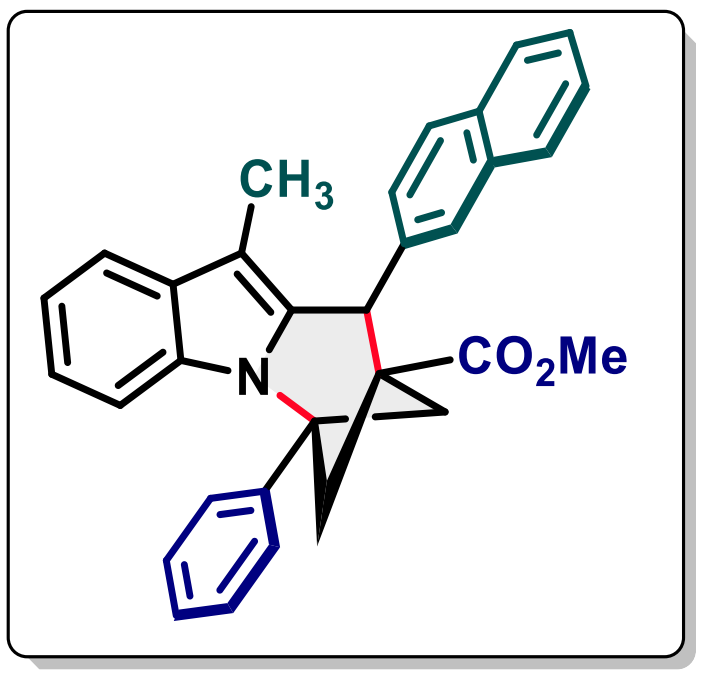

The reaction was performed according to the general procedure GP5 with methyl 3-phenylbicyclo[1.1.0]butane-1-carboxylate **1a** (56.4 mg, 0.3 mmol, 1.5 equiv.) and (3-methyl-1*H*-indol-2-yl)(naphthalen-2-yl)methanol **2d** (57.4 mg, 0.2 mmol, 1.00 equiv.) at 60 °C for 2 h. After purification by flash column chromatography (hexane/ethyl acetate = 9.75/0.25), the desired compound **3d** was obtained as a White solid (75.6 mg, 83% yield).

Rf = 0.46 (hexane/ethyl acetate = 9/1).

M.P. = 189 – 191 °C

^{1}H NMR ((400 MHz, $CDCl_3$) δ (ppm)) δ 7.89 – 7.75 (m, 3H), 7.74 – 7.69 (m, 1H), 7.68 – 7.61 (m, 1H), 7.59 – 7.41 (m, 8H), 7.40 – 7.32 (m, 2H), 6.96 (ddd, *J* = 8.0, 7.0, 0.9 Hz, 1H), 6.73 (ddd, *J* = 8.5, 7.0, 1.3 Hz, 1H), 5.37 – 5.33 (m, 2H), 3.63 (s, 3H), 3.11 – 2.80 (m, 5H), 1.85 (d, *J* = 0.9 Hz, 3H).

^{13}C {^{1}H} NMR ((101 MHz, $CDCl_3$) δ (ppm)) δ 173.85, 140.20, 137.76, 135.00, 133.60, 133.30, 132.85, 129.33, 129.13, 128.99, 128.92, 128.12, 128.10, 128.06, 128.03, 127.72, 126.96, 126.18, 125.96, 120.90, 118.36, 118.27, 111.37, 109.06, 62.39, 51.93, 48.44, 47.83, 47.39, 39.55, 8.73.

HRMS (ESI) Calcd. for $C_{32}H_{27}NO_2$ $[M+H]^+$, 458.2115, found: 458.2119.

FTIR (cm^{-1}) : 1101, 1208, 1247, 1329, 1450, 1729, 2936, 3048.

Methyl-9-(4-methoxyphenyl)-10-methyl-6-phenyl-6,7-dihydro-6,8-methanopyrido-[1,2-a]indole-8(9*H*)-carboxylate (3e):

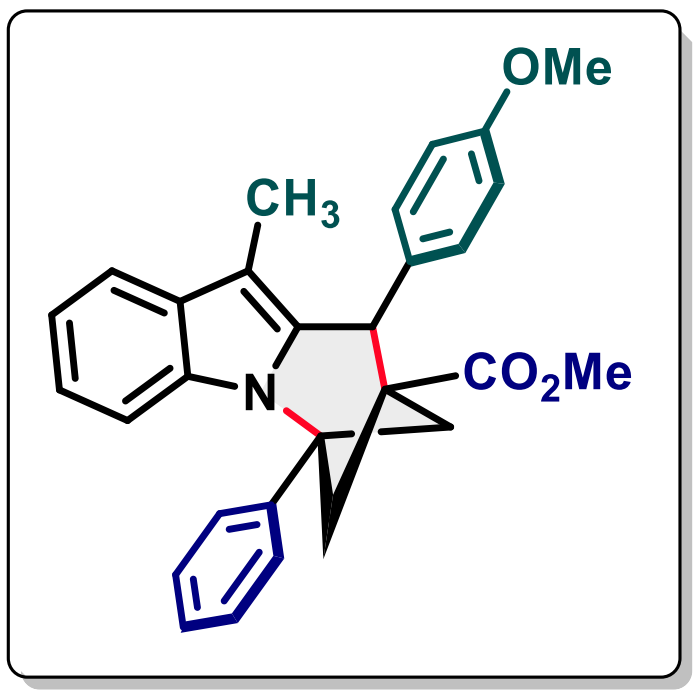

The reaction was performed according to the general procedure GP5 with methyl 3-phenylbicyclo[1.1.0]butane-1-carboxylate **1a** (56.4 mg, 0.3 mmol, 1.5 equiv.) and (4-methoxyphenyl)(3-methyl-1*H*-indol-2-yl)methanol **2e** (53.4 mg, 0.2 mmol, 1.00 equiv.) at 60 °C for 2 h. After purification by flash column chromatography (hexane/ethyl acetate = 9.75/0.25), the desired compound **3e** was obtained as a White solid (48.1 mg, 55% yield).

Rf = 0.37 (hexane/ethyl acetate = 9/1).

M.P. = 112 – 114 °C

^{1}H NMR ((400 MHz, $CDCl_3$) δ (ppm)) δ 7.62 – 7.44 (m, 4H), 7.40 (dddd, *J* = 9.4, 6.2, 1.8, 1.0 Hz, 2H), 7.16 – 7.08 (m, 2H), 6.90 (ddd, *J* = 7.8, 7.0, 0.9 Hz, 1H), 6.85 – 6.77 (m, 2H), 6.65 (ddd, *J* = 8.4, 7.0, 1.3 Hz, 1H), 5.26 (dt, *J* = 8.5, 0.8 Hz, 1H), 5.09 (s, 1H), 3.79 (s, 3H), 3.64 (s, 3H), 2.91 – 2.81 (m, 3H), 2.80 – 2.67 (m, 1H), 1.84 (d, *J* = 0.9 Hz, 3H).

^{13}C {^{1}H} NMR ((101 MHz, $CDCl_3$) δ (ppm)) δ 173.88, 158.81, 140.22, 134.82, 133.57, 132.29, 129.77, 129.04, 128.92, 128.01, 127.96, 120.70, 118.16, 113.79, 111.25, 108.71, 62.26, 55.27, 51.87, 48.31, 47.17, 46.92, 39.34, 8.61.

HRMS (ESI) Calcd. for $C_{29}H_{27}NO_3$ $[M+H]^+$, 438.2064, found: 438.2053.

FTIR (cm^{-1}) : 1029, 1101, 1178, 1205, 1250, 1329, 1452, 1509, 1608, 1732, 2928.

Methyl 9-(3-methoxyphenyl)-10-methyl-6-phenyl-6,7-dihydro-6,8-methanopyrido [1,2-a]indole-8(9*H*)-carboxylate (3f):

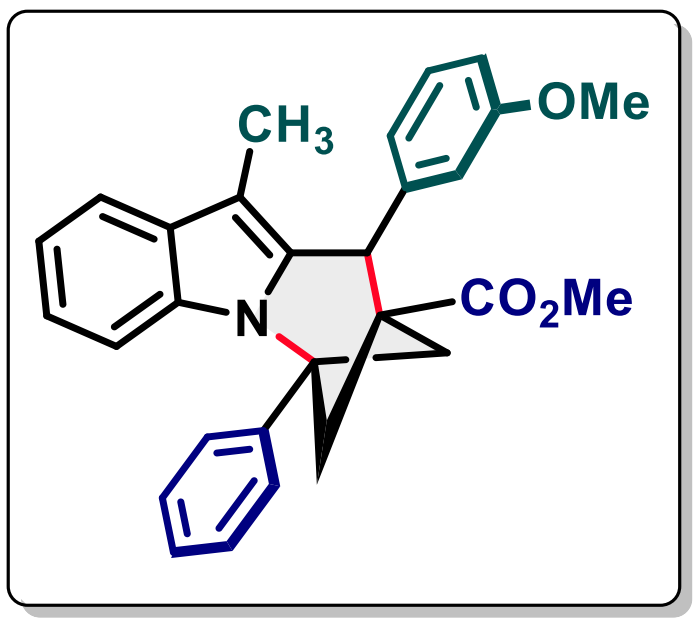

The reaction was performed according to the general procedure GP5 with methyl 3-phenylbicyclo[1.1.0]butane-1-carboxylate **1a** (56.4 mg, 0.3 mmol, 1.5 equiv.) and (3-methoxyphenyl)(3-methyl-1*H*-indol-2-yl)methanol **2f** (53.4 mg, 0.2 mmol, 1.00 equiv.) at 60 ºC for 2 h. After purification by flash column chromatography (hexane/ethyl acetate = 9.75/0.25), the desired compound **3f** was obtained as a White solid (62.1 mg, 71% yield).

Rf = 0.33 (hexane/ethyl acetate = 9/1).

M.P. = 117 – 119 ºC

^{1}H NMR ((400 MHz, $CDCl_3$) δ (ppm)) δ 7.65 – 7.34 (m, 6H), 7.20 (t, *J* = 8.1 Hz, 1H), 6.90 (ddd, *J* = 8.0, 7.0, 0.9 Hz, 1H), 6.84 – 6.74 (m, 3H), 6.66 (ddd, *J* = 8.5, 7.0, 1.3 Hz, 1H), 5.26 (d, *J* = 8.6 Hz, 1H), 5.11 (s, 1H), 3.75 (s, 3H), 3.65 (s, 3H), 2.95 – 2.82 (m, 3H), 2.78 – 2.68 (m, 2H), 1.86 (d, *J* = 0.9 Hz, 3H).

^{13}C {^{1}H} NMR ((101 MHz, $CDCl_3$) δ (ppm)) δ 173.83, 159.76, 141.74, 140.17, 134.90, 129.39, 129.25, 129.06, 128.93, 128.85, 127.98, 121.21, 120.75, 118.24, 118.13, 114.75, 112.43, 111.25, 108.95, 62.25, 55.32, 51.90, 48.28, 47.67, 47.27, 39.51, 8.66.

HRMS (ESI) Calcd. for $C_{29}H_{27}NO_3$ $[M+H]^+$, 438.2064, found: 438.2069.

FTIR (cm^{-1}) : 1045, 1097, 1217, 1269, 1332, 1450, 1583, 1735, 2924.

Methyl-9-(4-fluorophenyl)-10-methyl-6-phenyl-6,7-dihydro-6,8-methanopyrido[1,2-a]indole-8(9*H*)-carboxylate (3g):

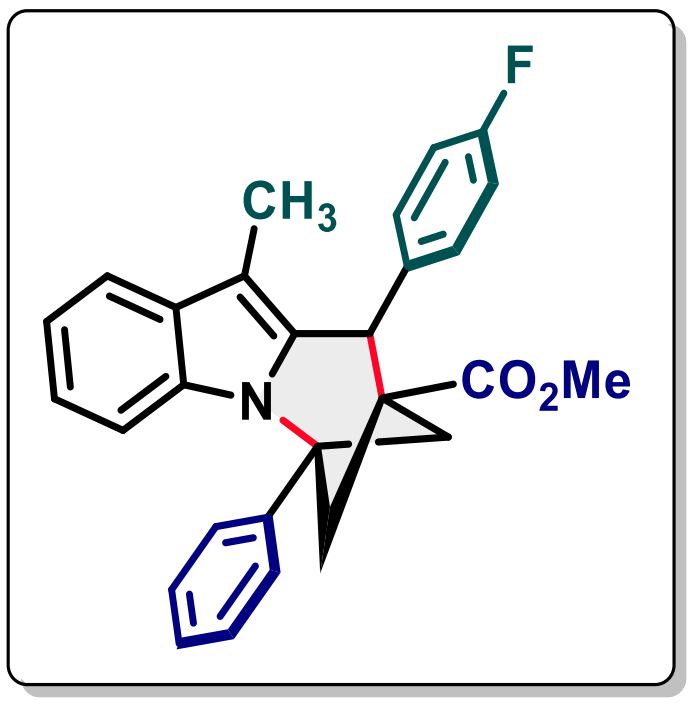

The reaction was performed according to the general procedure GP5 with methyl 3-phenylbicyclo[1.1.0]butane-1-carboxylate **1a** (56.4 mg, 0.3 mmol, 1.5 equiv.) and (4-fluorophenyl)(3-methyl-1*H*-indol-2-yl)methanol **2h** (50.2 mg, 0.2 mmol, 1.00 equiv.) at 60 ºC for 2 h. After purification by flash column chromatography (hexane/ethyl acetate = 9.8/0.2), the desired compound **3g** was obtained as a White solid (51.0 mg, 63% yield).

Rf = 0.44 (hexane/ethyl acetate = 9/1).

M.P. = 134 – 136 ºC

^{1}H NMR ((400 MHz, $CDCl_3$) δ (ppm)): δ 7.62 – 7.47 (m, 4H), 7.44 – 7.39 (m, 2H), 7.23 – 7.16 (m, 2H), 7.03 – 6.97 (m, 2H), 6.93 (ddd, *J* = 8.0, 7.0, 0.9 Hz, 1H), 6.68 (ddd, *J* = 8.5, 7.0, 1.3 Hz, 1H), 5.38 – 5.23 (m, 1H), 3.66 (s, 3H), 2.94 – 2.83 (m, 3H), 2.77 (dd, *J* = 9.7, 7.1 Hz, 1H), 1.85 (d, *J* = 0.9 Hz, 3H).

^{13}C {^{1}H} NMR ((101 MHz, $CDCl_3$) δ (ppm)): δ 173.68, 162.16 (d, *J* = 246.2 Hz), 140.05, 136.00, 134.89, 130.33 (d, *J* = 8.1 Hz), 129.20, 129.15, 128.99, 128.93, 127.98 (d, *J* = 8.2 Hz), 120.95, 118.29 (d, *J* = 3.0 Hz), 115.50, 115.28, 111.32, 108.84, 62.31, 51.97, 48.29, 47.25, 46.89, 39.28, 8.66.

^{19}FNMR (376 MHz, $CDCl_3$) δ -115.12.

HRMS (ESI) Calcd. for $C_{28}H_{24}FNO_2$ $[M+H]^+$, 426.1864, found: 426.1858.

FTIR (cm^{-1}) : 1018, 1066, 1109, 1177, 1326, 1451, 1728, 2987.

Methyl-9-(4-chlorophenyl)-10-methyl-6-phenyl-6,7-dihydro-6,8-methanopyrido[1,2-a]indole-8(9*H*)-carboxylate (3h):

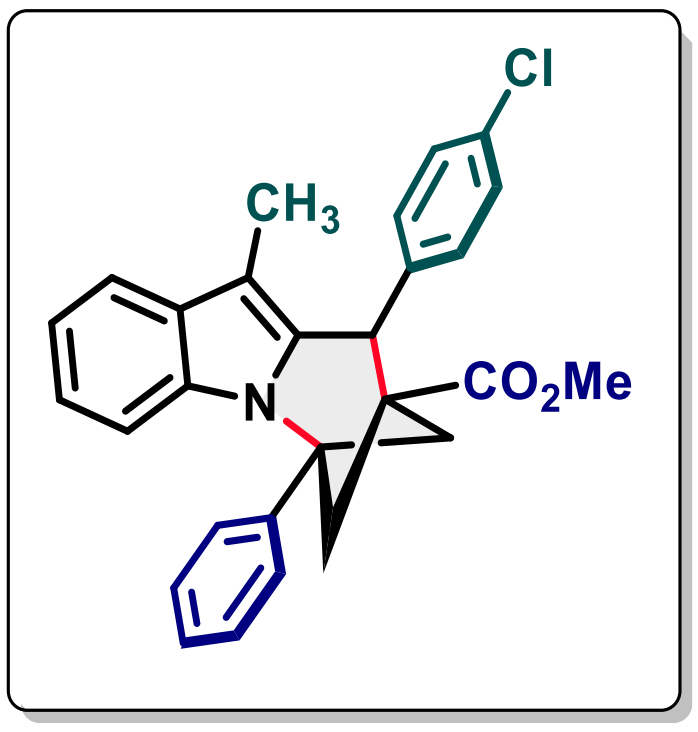

The reaction was performed according to the general procedure GP5 with methyl 3-phenylbicyclo[1.1.0]butane-1-carboxylate **1a** (56.4 mg, 0.3 mmol, 1.5 equiv.) and (4-chlorophenyl)(3-methyl-1*H*-indol-2-yl)methanol **2i** (54.2 mg, 0.2 mmol, 1.00 equiv.) at 60 °C for 2 h. After purification by flash column chromatography (hexane/ethyl acetate = 9.75/0.25), the desired compound **3h** was obtained as a White solid (67.9 mg, 77% yield).

Rf = 0.47 (hexane/ethyl acetate = 9/1).

M.P. = 129 – 131 °C

^{1}H NMR ((400 MHz, $CDCl_3$) δ (ppm)): δ 7.63 – 7.23 (m, 8H), 7.19 (dt, *J* = 8.5, 1.5 Hz, 2H), 6.94 (ddt, *J* = 8.0, 7.0, 1.1 Hz, 1H), 6.70 (ddt, *J* = 8.5, 7.0, 1.3 Hz, 1H), 5.31 (ddq, *J* = 8.6, 1.7, 1.0 Hz, 1H), 5.16 (s, 1H), 3.67 (d, *J* = 1.3 Hz, 3H), 2.97 – 2.81 (m, 2H), 2.80 – 2.64 (m, 2H), 1.87 (s, 3H).

^{13}C {^{1}H} NMR ((101 MHz, $CDCl_3$) δ (ppm)): δ 173.57, 140.02, 138.83, 134.95, 133.28, 132.77, 130.18, 129.18, 129.01, 128.94, 128.72, 128.01, 127.94, 121.03, 118.36, 118.32, 111.36, 108.93, 62.34, 52.01, 48.22, 47.33, 46.98, 39.32, 8.71.

HRMS (ESI) Calcd. for $C_{28}H_{24}ClNO_2$ $[M+H]^+$, 442.1568, found: 442.1571.

FTIR (cm^{-1}) : 1012, 1101, 1205, 1255, 1329, 1450, 1730, 2931.

Methyl-9-(3,4-dichlorophenyl)-10-methyl-6-phenyl-6,7-dihydro-6,8-methanopyrido-[1,2-a]indole-8(9*H*)-carboxylate (3i):

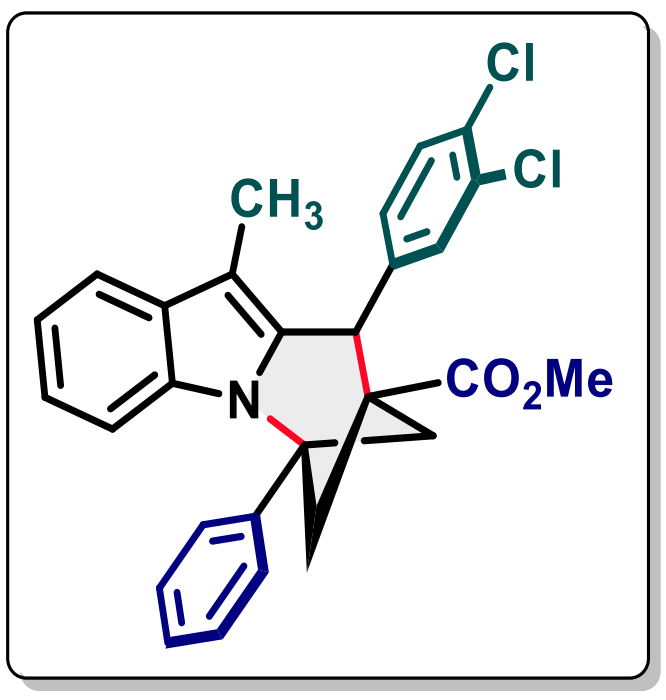

The reaction was performed according to the general procedure GP5 with methyl 3-phenylbicyclo[1.1.0]butane-1-carboxylate **1a** (56.4 mg, 0.3 mmol, 1.5 equiv.) and (3,4-dichlorophenyl)(3-methyl-1*H*-indol-2-yl)methanol **2j** (61.0 mg, 0.2 mmol, 1.00 equiv.) at 60 ºC for 2 h. After purification by flash column chromatography (hexane/ethyl acetate = 9.75/0.25), the desired compound **3i** was obtained as a White solid (40.1 mg, 43% yield).

Rf = 0.46 (hexane/ethyl acetate = 9/1).

M.P. = 188 – 190 ºC

^{1}H NMR ((400 MHz, $CDCl_3$) δ (ppm)): δ 7.60 – 7.45 (m, 4H), 7.43 (ddd, *J* = 7.9, 1.3, 0.7 Hz, 1H), 7.40 – 7.34 (m, 2H), 7.32 (d, *J* = 2.1 Hz, 1H), 7.06 (dd, *J* = 8.4, 2.1 Hz, 1H), 6.92 (ddd, *J* = 7.9, 7.0, 0.9 Hz, 1H), 6.68 (ddd, *J* = 8.5, 7.0, 1.3 Hz, 1H), 5.26 (dt, *J* = 8.6, 0.9 Hz, 1H), 5.10 (s, 1H), 3.67 (s, 3H), 2.96 – 2.86 (m, 2H), 2.82 – 2.68 (m, 2H), 1.86 (d, *J* = 0.9 Hz, 3H).

^{13}C {^{1}H} NMR ((101 MHz, $CDCl_3$) δ (ppm)): δ 173.36, 140.57, 139.79, 134.98, 132.73, 131.97, 131.56, 130.59, 130.45, 129.23, 129.08, 129.02, 128.94, 128.20, 127.92, 121.19, 118.42, 111.38, 109.15, 62.32, 52.10, 48.12, 47.27, 46.63, 39.29, 8.78.

HRMS (ESI) Calcd. for $C_{28}H_{23}Cl_2NO_2$ $[M+H]^+$, 476.1179, found: 476.1175.

FTIR (cm^{-1}) : 1104, 1214, 1245, 1332, 1452, 1738, 2855, 2923.

Methyl-10-methyl-6-phenyl-9-(4-(trifluoromethoxy)phenyl)-6,7-dihydro-6,8-methanopyrido [1,2-a]indole-8(9*H*)-carboxylate (3j):

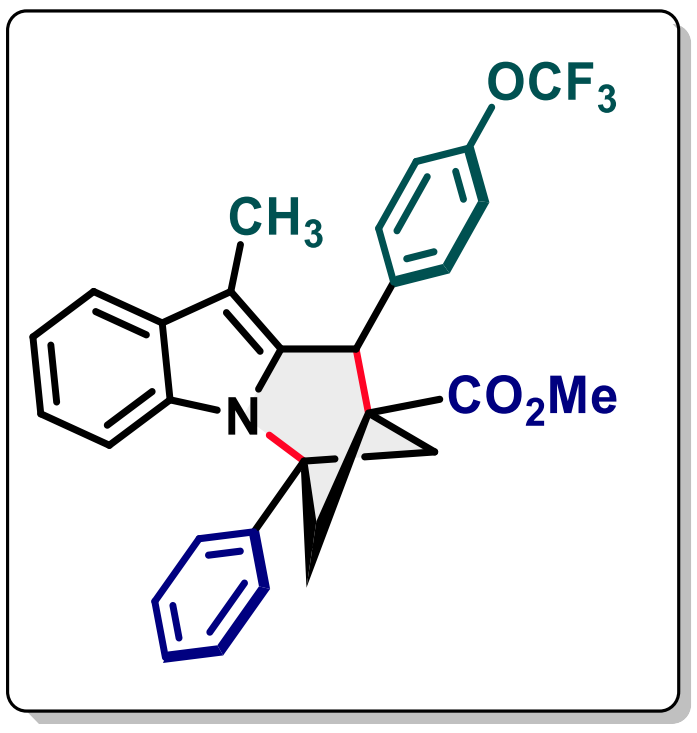

The reaction was performed according to the general procedure GP5 with methyl 3-phenylbicyclo[1.1.0]butane-1-carboxylate **1a** (56.4 mg, 0.3 mmol, 1.5 equiv.) and (3-methyl-1*H*-indol-2-yl)(4-(trifluoromethoxy)phenyl)methanol **2k** (64.2 mg, 0.2 mmol, 1.00 equiv.) at 60 ºC for 2 h. After purification by flash column chromatography (hexane/ethyl acetate = 9.75/0.25), the desired compound **3j** was obtained as a White solid (70.7 mg, 72% yield).

Rf = 0.48 (hexane/ethyl acetate = 9/1).

M.P. = 164 – 166 ºC

^{1}H NMR ((400 MHz, $CDCl_3$) δ (ppm)): δ 7.60 – 7.46 (m, 4H), 7.45 – 7.36 (m, 2H), 7.29 – 7.23 (m, 2H), 7.18 – 7.10 (m, 2H), 6.92 (ddd, *J* = 7.9, 7.0, 0.9 Hz, 1H), 6.68 (ddd, *J* = 8.5, 7.0, 1.3 Hz, 1H), 5.29 (dt, *J* = 8.6, 0.8 Hz, 1H), 5.17 (s, 1H), 3.65 (s, 3H), 2.96 – 2.81 (m, 3H), 2.76 (dd, *J* = 9.6, 7.6 Hz, 1H), 1.84 (d, *J* = 0.9 Hz, 3H).

^{13}C {^{1}H} NMR ((101 MHz, $CDCl_3$) δ (ppm)): δ 173.52, 148.54(q, 6Hz) , 139.98, 138.95, 134.95, 132.73, 130.18, 129.18, 129.16, 128.99, 128.93, 127.99, 121.04, 120.79, 118.35, 118.31, 111.35, 108.96, 62.34, 51.96, 48.23, 47.33, 46.90, 39.33, 8.65.

^{19}F NMR (376 MHz, $CDCl_3$) δ -57.66.

HRMS (ESI) Calcd. for $C_{29}H_{24}F_3NO_3$ $[M+H]^+$, 492.1781, found: 492.1786.

FTIR (cm^{-1}) : 1102, 1172, 1207, 1253, 1328, 1452, 1725, 2995.

Methyl-10-methyl-6-phenyl-9-(4-(trifluoromethyl)phenyl)-6,7-dihydro-6,8-methanopyrido[1,2-a]indole-8(9*H*)-carboxylate (3k):

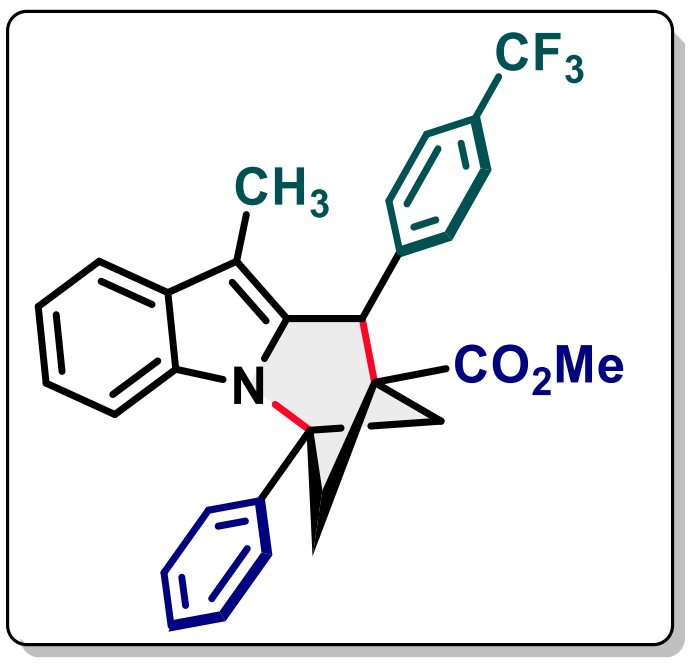

The reaction was performed according to the general procedure GP5 with methyl 3-phenylbicyclo[1.1.0]butane-1-carboxylate **1a** (56.4 mg, 0.3 mmol, 1.5 equiv.) and (3-methyl-1*H*-indol-2-yl)(4-(trifluoromethyl)phenyl)methanol **2l** (61.0 mg, 0.2 mmol, 1.00 equiv.) at 60 ºC for 2 h. After purification by flash column chromatography (hexane/ethyl acetate = 9.75/0.25), the desired compound **3k** was obtained as a White solid (58.9 mg, 62% yield).

Rf = 0.45 (hexane/ethyl acetate = 9/1).

M.P. = 135 – 137 ºC

^{1}H NMR ((400 MHz, $CDCl_3$) δ (ppm)): δ 7.58 – 7.51 (m, 5H), 7.51 – 7.46 (m, 1H), 7.45 – 7.37 (m, 2H), 7.36 – 7.31 (m, 2H), 6.92 (ddd, *J* = 8.0, 7.0, 0.9 Hz, 1H), 6.68 (ddd, *J* = 8.4, 7.0, 1.3 Hz, 1H), 5.28 (dt, *J* = 8.5, 0.8 Hz, 1H), 5.21 (s, 1H), 3.65 (s, 3H), 2.95 – 2.88 (m, 2H), 2.87 – 2.81 (m, 1H), 2.77 (dd, *J* = 9.5, 7.7 Hz, 1H), 1.82 (d, *J* = 0.9 Hz, 3H).

^{13}C {^{1}H} NMR ((101 MHz, $CDCl_3$) δ (ppm)): δ 173.40, 144.32, 139.85, 134.96, 132.34, 129.20, 129.17, 129.10, 129.00, 128.93, 127.95, 127.87, 125.43 (q, *J* = 7.3, 3.7 Hz), 121.11, 118.39, 118.35, 111.35, 109.03, 62.34, 52.05, 48.17, 47.38, 47.23, 39.28, 8.69.

^{19}F NMR (376 MHz, $CDCl_3$) δ -115.12.

HRMS (ESI) Calcd. for $C_{29}H_{24}F_3NO_2$ $[M+H]^+$, 476.1832, found: 476.1833.

FTIR (cm^{-1}) : 1018, 1066, 1109, 1177, 1326, 1451, 1728, 2987.

Methyl-10-methyl-6-phenyl-9-(thiophen-2-yl)-6,7-dihydro-6,8-methanopyrido[1,2-a]indole-8(9*H*)-carboxylate (3l):

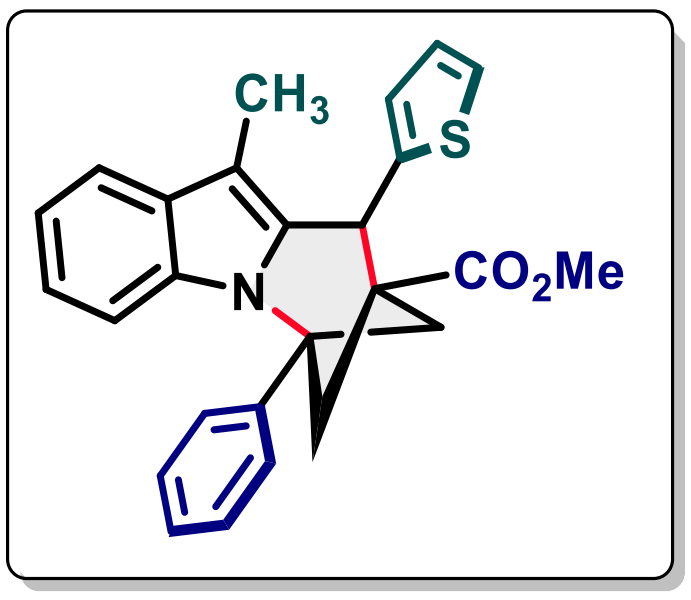

The reaction was performed according to the general procedure GP5 with methyl 3-phenylbicyclo[1.1.0]butane-1-carboxylate **1a** (56.4 mg, 0.3 mmol, 1.5 equiv.) and (3-methyl-1*H*-indol-2-yl)(thiophen-2-yl)methanol **2m** (48.6 mg, 0.2 mmol, 1.00 equiv.) at 60 ºC for 2 h. After purification by flash column chromatography (hexane/ethyl acetate = 9.8/0.2), the desired compound **3l** was obtained as a White solid (57.8 mg, 70% yield).

Rf = 0.49 (hexane/ethyl acetate = 9/1).

M.P. = 166 – 168 ºC

^{1}H NMR ((400 MHz, $CDCl_3$) δ (ppm)): δ 7.62 – 7.43 (m, 5H), 7.39 (ddd, *J* = 7.2, 2.1, 1.0 Hz, 1H), 7.21 (dd, *J* = 5.1, 1.3 Hz, 1H), 7.01 – 6.85 (m, 3H), 6.68 (ddd, *J* = 8.5, 7.0, 1.3 Hz, 1H), 5.46 (d, *J* = 1.2 Hz, 1H), 5.28 (d, *J* = 8.6 Hz, 1H), 3.74 (s, 3H), 3.04 – 2.88 (m, 3H), 2.72 (dd, *J* = 9.8, 7.8 Hz, 1H), 1.99 (d, *J* = 0.9 Hz, 3H).

^{13}C {^{1}H} NMR ((101 MHz, $CDCl_3$) δ (ppm)): δ 173.39, 143.32, 140.09, 134.90, 133.03, 129.21, 129.13, 128.99, 128.87, 128.04, 127.92, 126.57, 126.56, 124.77, 121.05, 118.42, 118.26, 111.32, 109.64, 62.56, 52.12, 48.39, 46.71, 42.98, 40.11, 8.70.

HRMS (ESI) Calcd. for $C_{26}H_{23}NO_2S$ $[M+H]^+$, 414.1522, found: 414.1525.

FTIR (cm^{-1}) : 1100, 1217, 1244, 1331, 1451, 1740, 2946.

Methyl-6,9,10-triphenyl-6,7-dihydro-6,8-methanopyrido[1,2-a]indole-8(9*H*)-carboxylate (3m):

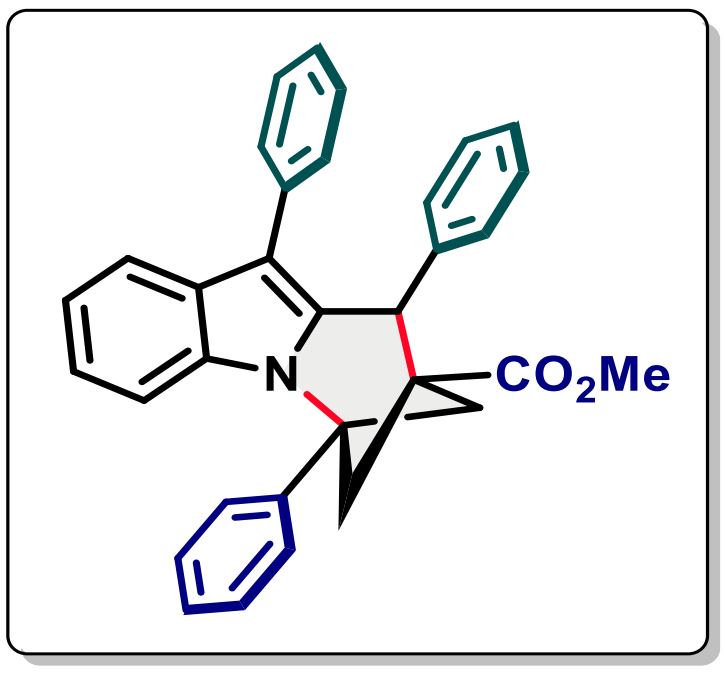

The reaction was performed according to the general procedure GP5 with methyl 3-phenylbicyclo[1.1.0]butane-1-carboxylate **1a** (56.4 mg, 0.3 mmol, 1.5 equiv.) and phenyl(3-phenyl-1*H*-indol-2-yl)methanol **2p** (59.8 mg, 0.2 mmol, 1.00 equiv.) at 60 ºC for 2 h. After purification by flash column chromatography (hexane/ethyl acetate = 9.7/0.3), the desired compound **3m** was obtained as a White solid (61.9 mg, 66% yield).

Rf = 0.41 (hexane/ethyl acetate = 9/1).

M.P. = 190 – 192 ºC

^{1}**H NMR** ((400 MHz, $CDCl_3$) δ (ppm)): δ 7.68 – 7.43 (m, 6H), 7.28 – 7.19 (m, 2H), 7.19 – 7.11 (m, 2H), 7.12 – 7.01 (m, 6H), 6.94 (ddd, *J* = 8.0, 7.0, 1.0 Hz, 1H), 6.73 (ddd, *J* = 8.4, 7.0, 1.4 Hz, 1H), 5.39 (s, 1H), 5.38 – 5.35 (m, 1H), 3.62 (s, 3H), 3.01 (dd, *J* = 10.5, 7.4 Hz, 1H), 2.96 – 2.85 (m, 3H).

^{13}**C {^{1}H} NMR** ((101 MHz, $CDCl_3$) δ (ppm)): δ 173.63, 140.21, 140.08, 135.34, 134.53, 134.11, 129.41, 129.27, 129.05, 128.99, 128.86, 128.25, 128.18, 128.04, 127.99, 127.10, 125.84, 121.36, 119.34, 119.30, 115.91, 111.80, 62.92, 51.93, 48.36, 48.06, 47.33, 39.20.

HRMS (ESI) Calcd. for $C_{33}H_{27}NO_2$ $[M+H]^+$, 470.2115, found: 470.2113.

FTIR (cm^{-1}) : 1105, 1214, 1329, 1448, 1734, 2947.

Methyl-10-(4-methoxyphenyl)-6,9-diphenyl-6,7-dihydro-6,8-methanopyrido[1,2-a]indole-8(9*H*)-carboxylate (3n):

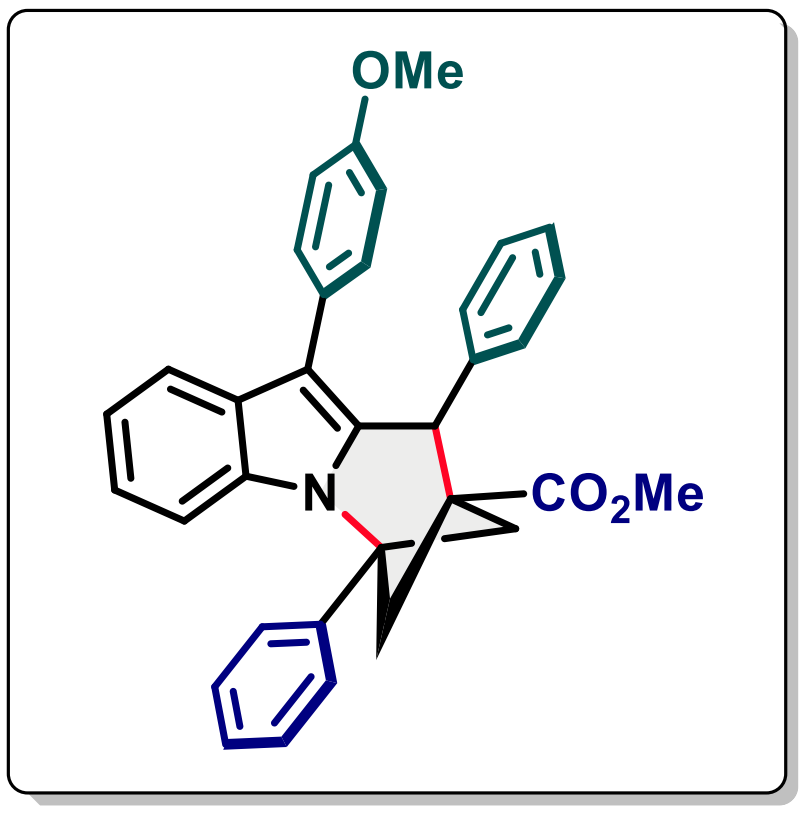

The reaction was performed according to the general procedure GP5 with methyl 3-phenylbicyclo[1.1.0]butane-1-carboxylate **1a** (56.4 mg, 0.3 mmol, 1.5 equiv.) and (3-(4-methoxyphenyl)-1*H*-indol-2-yl)(phenyl)methanol **2q** (65.8 mg, 0.2 mmol, 1.00 equiv.) at 60 ºC for 2 h. After purification by flash column chromatography (hexane/ethyl acetate = 9.2/0.8), the desired compound **3n** was obtained as a White solid (53.9 mg, 54% yield).

Rf = 0.28 (hexane/ethyl acetate = 9/1).

M.P. = 191 – 193 ºC

^{1}H NMR ((400 MHz, $CDCl_3$) δ (ppm)): δ 7.72 – 7.61 (m, 1H), 7.57 – 7.44 (m, 5H), 7.13 – 7.08 (m, 7H), 6.93 (ddd, J = 7.9, 7.0, 0.9 Hz, 1H), 6.74 – 6.69 (m, 3H), 5.42 – 5.29 (m, 2H), 3.74 (s, 3H), 3.62 (s, 3H), 3.02 (dd, J = 10.6, 7.7 Hz, 1H), 2.97 – 2.81 (m, 3H).

^{13}C {^{1}H} NMR ((101 MHz, $CDCl_3$) δ (ppm)): δ 173.67, 157.73, 140.48, 140.17, 135.32, 133.89, 130.50, 129.23, 129.03, 128.96, 128.85, 128.56, 128.18, 128.05, 128.01, 127.14, 126.89, 121.27, 119.31, 119.16, 115.57, 113.50, 111.78, 62.91, 55.27, 51.91, 48.34, 47.96, 47.29, 39.19.

HRMS (ESI) Calcd. for $C_{34}H_{29}NO_3$ $[M+H]^+$, 500.2220, found: 500.2223.

FTIR (cm^{-1}) : 1031, 1104, 1212, 1241, 1332, 1449, 1505, 1731, 2948.

Methyl-10-(4-fluorophenyl)-6,9-diphenyl-6,7-dihydro-6,8-methanopyrido[1,2-a]indole-8(9*H*)-carboxylate (3o):

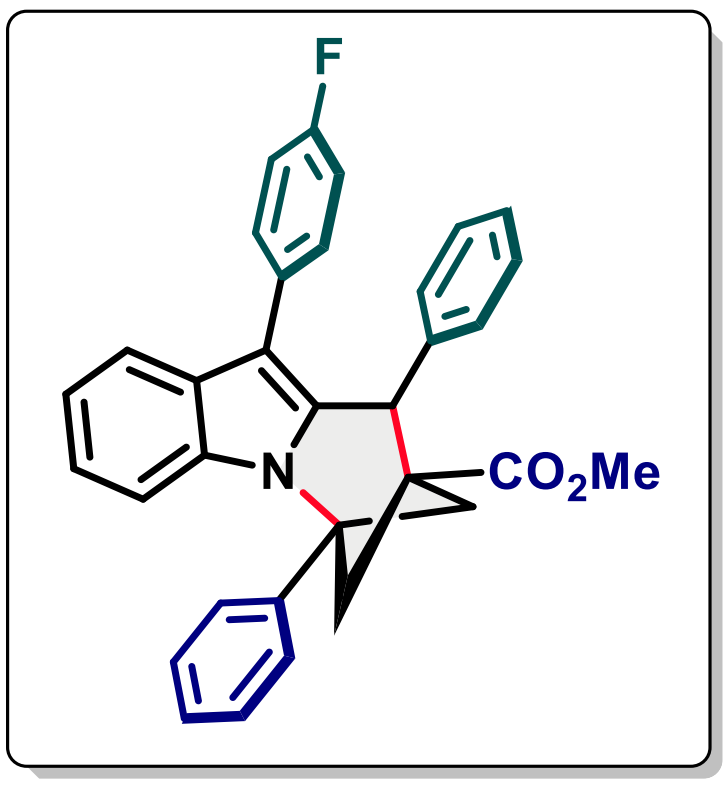

The reaction was performed according to the general procedure GP5 with methyl 3-phenylbicyclo[1.1.0]butane-1-carboxylate **1a** (56.4 mg, 0.3 mmol, 1.5 equiv.) and (3-(4-fluorophenyl)-1*H*-indol-2-yl)(phenyl)methanol **2r** (63.4 mg, 0.2 mmol, 1.00 equiv.) at 60 ºC for 2 h. After purification by flash column chromatography (hexane/ethyl acetate = 9.7/0.3), the desired compound **3o** was obtained as a White solid (58.4 mg, 60% yield).

Rf = 0.39 (hexane/ethyl acetate = 9/1).

M.P. = 195 – 197 ºC

^{1}H NMR ((400 MHz, $CDCl_3$) δ (ppm)): δ 7.69 – 7.61 (m, 1H), 7.61 – 7.43 (m, 5H), 7.20 – 7.04 (m, 7H), 6.95 (ddd, *J* = 8.0, 7.0, 1.0 Hz, 1H), 6.90 – 6.80 (m, 2H), 6.74 (ddd, *J* = 8.5, 7.0, 1.3 Hz, 1H), 5.39 (dt, *J* = 8.6, 0.9 Hz, 1H), 5.34 (s, 1H), 3.62 (s, 3H), 3.06 – 2.85 (m, 4H).

^{13}C {^{1}H} NMR ((101 MHz, $CDCl_3$) δ (ppm)): δ 173.51, 161.22 (d, *J* = 244.4 Hz), 140.04 (d, *J* = 5.2 Hz), 135.25, 134.21, 130.94 (d, *J* = 7.8 Hz), 129.30, 129.04 (d, *J* = 5.2 Hz), 128.86, 128.28, 128.16, 128.08, 128.03, 127.25, 121.46, 119.39, 119.03, 114.99, 114.89, 114.78, 111.84, 62.94, 51.92, 48.37, 48.07, 47.40, 39.24.

^{19}F NMR ((376 MHz, $CDCl_3$) δ (ppm)): δ -116.74.

HRMS (ESI) Calcd. for $C_{33}H_{26}FNO_2$ $[M+H]^+$, 488.2020, found: 488.2028.

FTIR (cm^{-1}) : 1104, 1212, 1329, 1449, 1500, 1723, 2953.

Methyl-10-(4-chlorophenyl)-6,9-diphenyl-6,7-dihydro-6,8-methanopyrido[1,2-a]indole-8(9*H*)-carboxylate (3p):

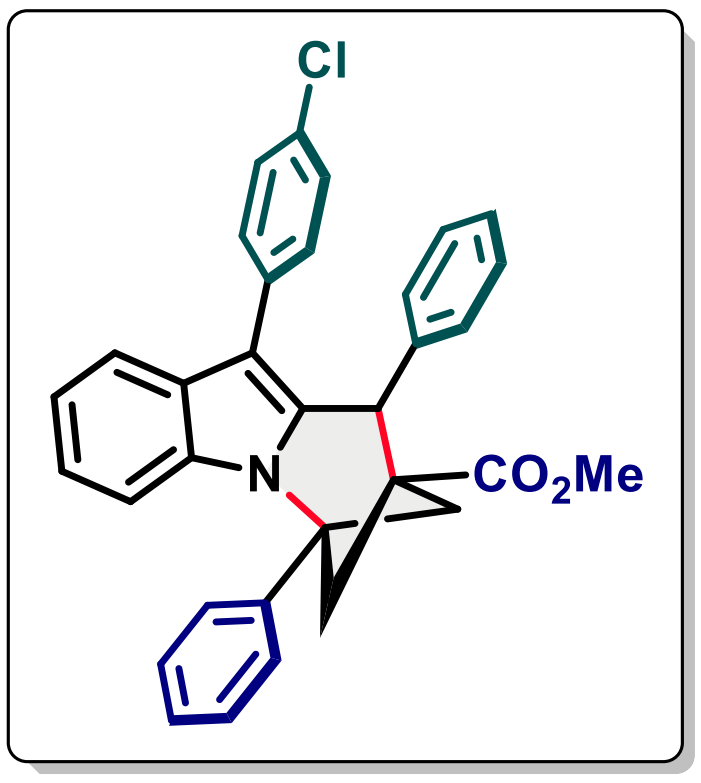

The reaction was performed according to the general procedure GP5 with methyl 3-phenylbicyclo[1.1.0]butane-1-carboxylate **1a** (56.4 mg, 0.3 mmol, 1.5 equiv.) and (3-(4-chlorophenyl)-1*H*-indol-2-yl)(phenyl)methanol **2s** (66.6 mg, 0.2 mmol, 1.00 equiv.) at 60 ºC for 2 h. After purification by flash column chromatography (hexane/ethyl acetate = 9.4/0.6), the desired compound **3p** was obtained as a White solid (67.4 mg, 67% yield).

Rf = 0.39 (hexane/ethyl acetate = 9/1).

M.P. = 205 – 207 ºC

^{1}H NMR ((400 MHz, $CDCl_3$) δ (ppm)): δ 7.70 – 7.62 (m, 1H), 7.62 – 7.36 (m, 5H), 7.13 (dd, *J* = 7.1, 2.1 Hz, 7H), 7.10 – 7.03 (m, 2H), 6.97 (ddd, *J* = 8.0, 7.0, 0.9 Hz, 1H), 6.75 (ddd, *J* = 8.5, 7.0, 1.3 Hz, 1H), 5.42 – 5.31 (m, 2H), 3.63 (s, 3H), 3.14 – 2.56 (m, 4H).

^{13}C {^{1}H} NMR ((101 MHz, $CDCl_3$) δ (ppm)): δ 173.50, 140.07, 139.95, 135.35, 134.38, 133.11, 131.54, 130.65, 129.35, 129.10, 129.06, 128.85, 128.21, 128.17, 128.04, 127.34, 121.57, 119.53, 119.03, 114.65, 111.94, 63.02, 51.98, 48.36, 48.06, 47.35, 39.18.

HRMS (ESI) Calcd. for $C_{33}H_{26}ClNO_2$ $[M+H]^+$, 504.1725, found: 504.1721.

FTIR (cm^{-1}) : 1102, 1210, 1328, 1448, 1724, 2924.

methyl 10-(naphthalen-2-yl)-6,9-diphenyl-6,7-dihydro-6,8-methanopyrido[1,2-a]indole-8(9*H*)-carboxylate (3q):

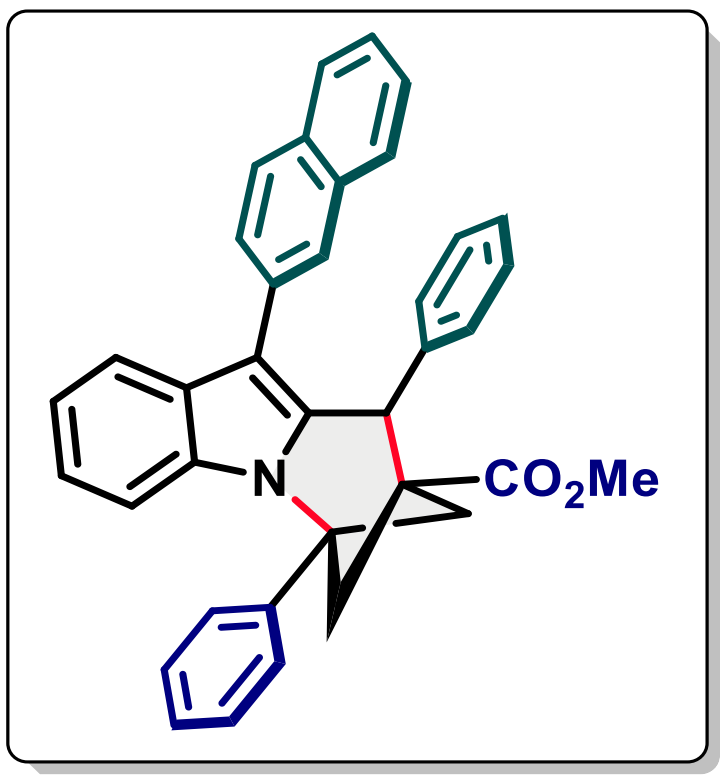

The reaction was performed according to the general procedure GP5 with methyl 3-phenylbicyclo[1.1.0]butane-1-carboxylate **1a** (56.4 mg, 0.3 mmol, 1.5 equiv.) and (3-(naphthalen-2-yl)-1*H*-indol-2-yl)(phenyl)methanol **2t** (69.8 mg, 0.2 mmol, 1.00 equiv.) at 60 °C for 2 h. After purification by flash column chromatography (hexane/ethyl acetate = 9.6/0.4), the desired compound **3q** was obtained as a White solid (59.2 mg, 57% yield).
Rf = 0.37 (hexane/ethyl acetate = 9/1).
M.P. = 214 – 216 °C

^{1}H NMR ((400 MHz, $CDCl_3$) δ (ppm)): δ 7.77 – 7.69 (m, 1H), 7.69 – 7.59 (m, 3H), 7.59 – 7.48 (m, 5H), 7.48 – 7.40 (m, 2H), 7.40 – 7.31 (m, 2H), 7.15 – 7.05 (m, 5H), 6.96 (ddd, *J* = 8.0, 7.0, 1.0 Hz, 1H), 6.75 (ddd, *J* = 8.4, 7.0, 1.3 Hz, 1H), 5.39 (d, *J* = 8.7 Hz, 2H), 3.61 (s, 3H), 3.09 (dd, *J* = 10.7, 7.8 Hz, 1H), 3.00 – 2.82 (m, 3H).

^{13}C {^{1}H} NMR ((101 MHz, $CDCl_3$) δ (ppm)): δ 173.57, 140.85, 140.13, 135.59, 134.67, 133.44, 132.09, 131.82, 129.27, 129.06, 128.98, 128.90, 128.55, 128.20, 128.13, 128.07, 128.02, 127.97, 127.84, 127.54, 127.37, 127.15, 125.69, 125.28, 121.47, 119.48, 119.40, 115.84, 112.01, 63.13, 51.91, 48.35, 48.09, 47.27, 39.11.
HRMS (ESI) Calcd. for $C_{37}H_{29}NO_2$ $[M+H]^+$, 520.2271, found: 520.2275.
FTIR (cm^{-1}) : 1104, 1211, 1330, 1448, 1602, 1727, 2925.

Methyl-2-methoxy-6,9,10-triphenyl-6,7-dihydro-6,8-methanopyrido[1,2-a]indole-8(9*H*)-carboxylatecarboxylate (3r):

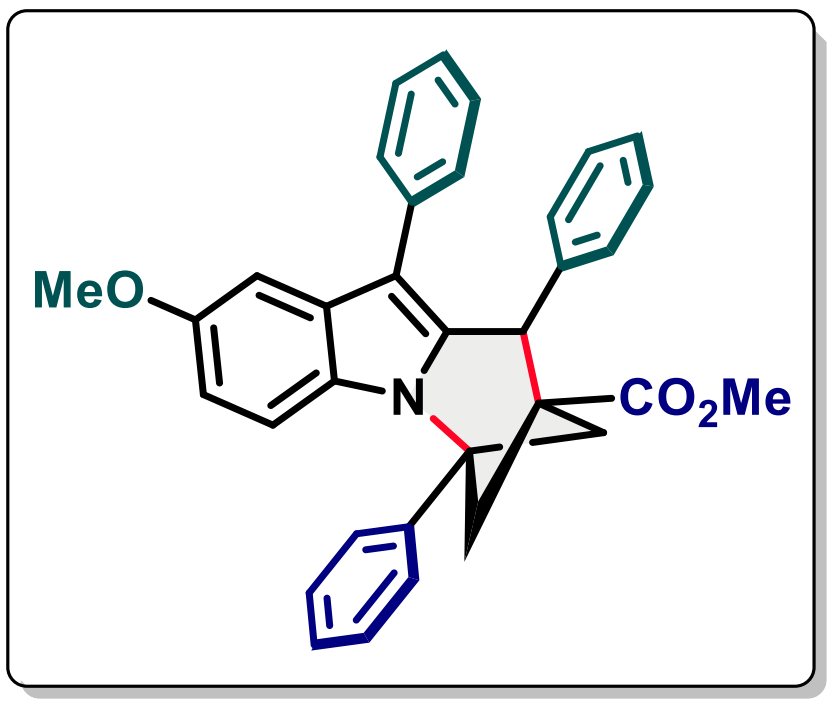

The reaction was performed according to the general procedure GP5 with methyl 3-phenylbicyclo[1.1.0]butane-1-carboxylate **1a** (56.4 mg, 0.3 mmol, 1.5 equiv.) and (5-methoxy-3-phenyl-1*H*-indol-2-yl)(phenyl)methanol **2u** (65.8 mg, 0.2 mmol, 1.00 equiv.) at 60 ºC for 2 h. After purification by flash column chromatography (hexane/ethyl acetate = 9.3/0.7), the desired compound **3r** was obtained as a White solid (69.9 mg, 70% yield).

Rf = 0.33 (hexane/ethyl acetate = 9/1).

M.P. = 181 – 183 ºC

^{1}H NMR ((400 MHz, $CDCl_3$) δ (ppm)): δ 7.69 – 7.48 (m, 4H), 7.47 – 7.41 (m, 1H), 7.23 – 7.13 (m, 4H), 7.12 – 7.04 (m, 6H), 7.03 (d, *J* = 2.6 Hz, 1H), 6.40 (dd, *J* = 9.2, 2.6 Hz, 1H), 5.36 (s, 1H), 5.24 (d, *J* = 9.2 Hz, 1H), 3.71 (s, 3H), 3.61 (s, 3H), 3.01 (dd, *J* = 10.6, 7.6 Hz, 1H), 2.96 – 2.82 (m, 3H).

^{13}C {^{1}H} NMR ((101 MHz, $CDCl_3$) δ (ppm)): δ 173.63, 153.87, 140.31, 140.00, 134.91, 134.68, 130.65, 129.32, 129.29, 129.06, 129.00, 128.87, 128.68, 128.13, 128.09, 128.01, 127.99, 127.10, 125.80, 115.55, 112.63, 111.43, 100.94, 62.86, 55.91, 51.93, 48.37, 48.08, 47.22, 39.14.

HRMS (ESI) Calcd. for $C_{30}H_{29}NO_3$ $[M+H]^+$, 500.2220, found: 500.2226.

FTIR (cm^{-1}) : 1034, 1106, 1215, 1233, 1324, 1437, 1475, 1736, 2943.

Methyl-2-fluoro-6,9,10-triphenyl-6,7-dihydro-6,8-methanopyrido[1,2-a]indole-8(9*H*)-carboxylatecarboxylate (3s):

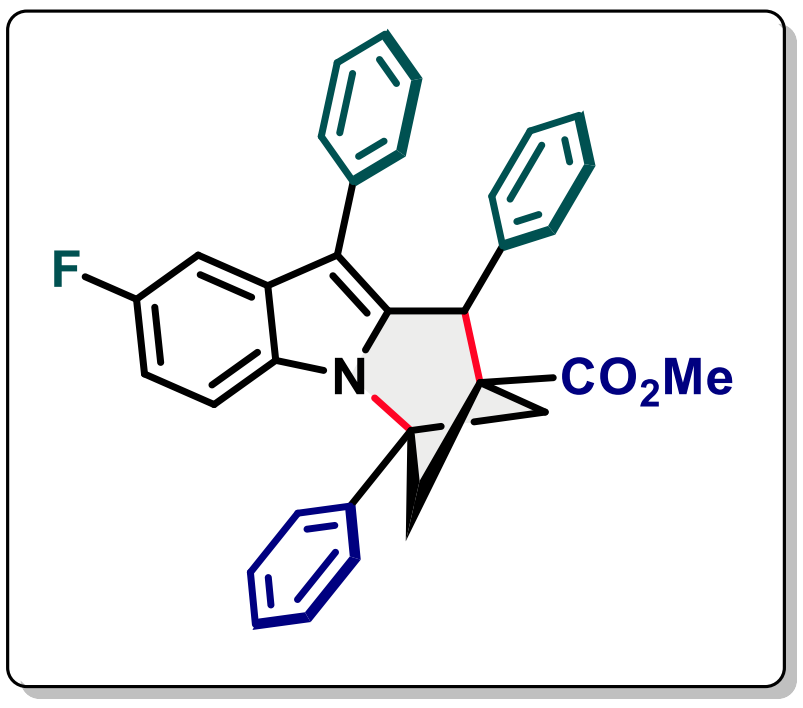

The reaction was performed according to the general procedure GP5 with methyl 3-phenylbicyclo[1.1.0]butane-1-carboxylate **1a** (56.4 mg, 0.3 mmol, 1.5 equiv.) and (5-fluoro-3-phenyl-1*H*-indol-2-yl)(phenyl)methanol **2v** (63.4 mg, 0.2 mmol, 1.00 equiv.) at 60 ºC for 2 h. After purification by flash column chromatography (hexane/ethyl acetate = 9.5/0.5), the desired compound **3s** was obtained as a White solid (49.7 mg, 51% yield).

Rf = 0.34 (hexane/ethyl acetate = 9/1).

M.P. = 167 – 169 ºC

^{1}H NMR ((400 MHz, $CDCl_3$) δ (ppm)): δ 7.67 – 7.47 (m, 4H), 7.46 – 7.41 (m, 1H), 7.20 – 7.11 (m, 5H), 7.11 – 7.01 (m, 6H), 6.46 (td, *J* = 9.2, 2.7 Hz, 1H), 5.36 (s, 1H), 5.22 (ddd, *J* = 9.3, 4.5, 0.5 Hz, 1H), 3.61 (s, 3H), 3.05 – 2.79 (m, 4H).

^{13}C {^{1}H} NMR ((101 MHz, $CDCl_3$) δ (ppm)): δ 157.69 (d, *J* = 235.3 Hz), 139.99, 139.77, 135.80, 134.09, 131.95, 129.39, 129.19, 129.10, 128.83, 128.73 (d, *J* = 9.6 Hz), 128.09, 128.04, 127.97, 127.17, 126.02, 115.89 (d, *J* = 4.8 Hz), 112.47 (d, *J* = 9.1 Hz), 109.56 (d, *J* = 25.5 Hz), 103.94 (d, *J* = 23.4 Hz), 63.04, 51.91, 48.30, 48.13, 47.24, 39.10.

HRMS (ESI) Calcd. for $C_{33}H_{26}FNO_2$ $[M+H]^+$, 488.2020, found: 488.2020.

FTIR (cm^{-1}) : 1107, 1215, 1327, 1440, 1471, 1739, 2924.

Methyl-2-bromo-6,9,10-triphenyl-6,7-dihydro-6,8-methanopyrido[1,2-a]indole-8(9*H*)-carboxylate (3t):

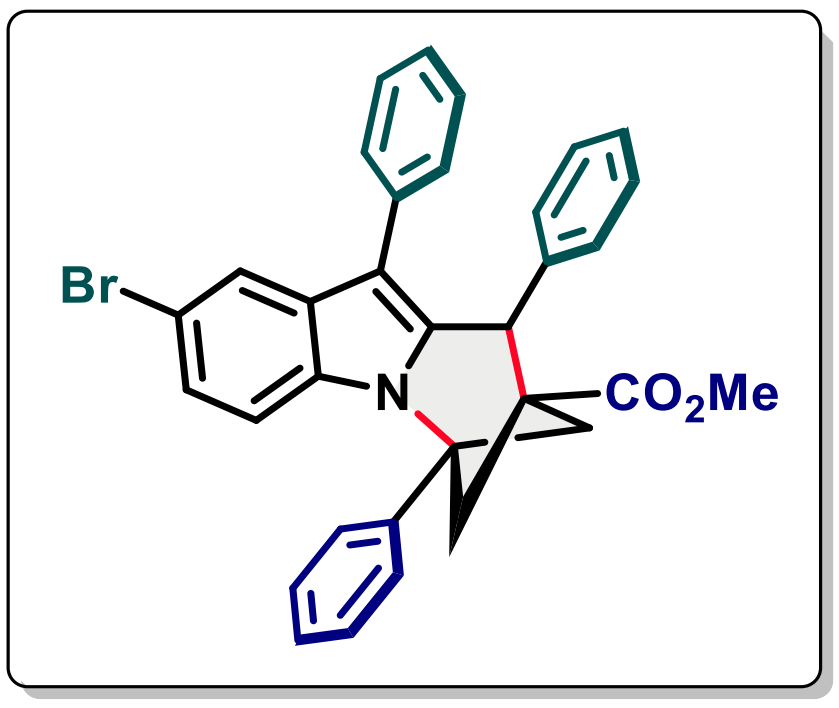

The reaction was performed according to the general procedure GP5 with methyl 3-phenylbicyclo[1.1.0]butane-1-carboxylate **1a** (56.4 mg, 0.3 mmol, 1.5 equiv.) and (5-bromo-3-phenyl-1*H*-indol-2-yl)(phenyl)methanol **2a** (75.4 mg, 0.2 mmol, 1.00 equiv.) at 60 ºC for 2 h. After purification by flash column chromatography (hexane/ethyl acetate = 9.4/0.6), the desired compound **3t** was obtained as a White solid (58.0 mg, 53% yield).

Rf = 0.34 (hexane/ethyl acetate = 9/1).

M.P. = 176 – 178 ºC

^{1}H NMR ((400 MHz, $CDCl_3$) δ (ppm)): δ 7.65 – 7.46 (m, 5H), 7.46 – 7.38 (m, 1H), 7.14 (d, J = 4.4 Hz, 5H), 7.11 – 6.98 (m, 7H), 6.78 (dd, J = 9.1, 2.1 Hz, 1H), 5.34 (s, 1H), 5.16 (d, J = 9.1 Hz, 1H), 3.60 (s, 3H), 2.99 – 2.86 (m, 4H).

^{13}C {^{1}H} NMR ((101 MHz, $CDCl_3$) δ (ppm)): δ 173.34, 139.89, 139.60, 135.33, 133.94, 133.79, 130.04, 129.47, 129.34, 129.14, 129.08, 128.81, 128.14, 128.06, 127.96, 127.23, 126.18, 124.15, 121.65, 115.55, 113.17, 112.85, 63.12, 51.94, 48.27, 48.02, 47.22, 39.01.

HRMS (ESI) Calcd. for $C_{33}H_{26}BrNO_2$ $[M+H]^+$, 448.1220, found: 448.1228.

FTIR (cm^{-1}) : 1106, 1214, 1330, 1447, 1737, 2924.

Ethyl-10-methyl-6,9-diphenyl-6,7-dihydro-6,8-methanopyrido[1,2-a]indole-8(9*H*)-carboxylate (3u):

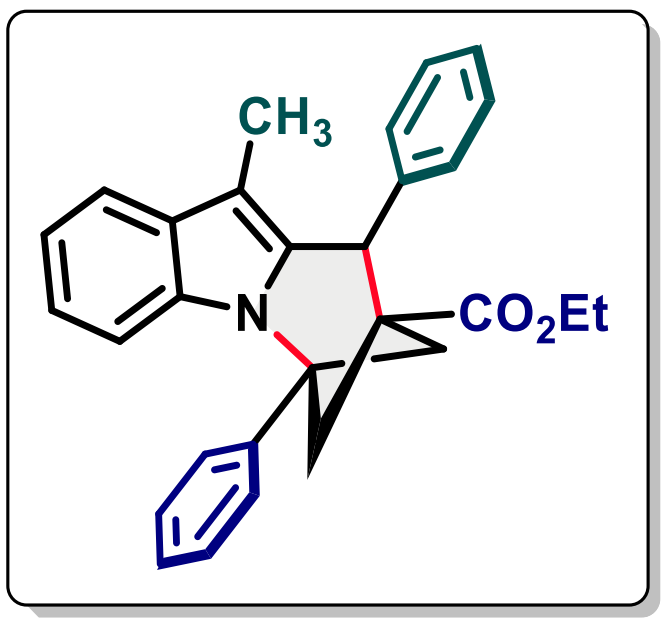

The reaction was performed according to the general procedure GP5 with ethyl 3-phenylbicyclo[1.1.0]butane-1-carboxylate **1b** (28.2 mg, 0.3 mmol, 1.5 equiv.) and (3-methyl-1*H*-indol-2-yl)(phenyl)methanol **2a** (47.4 mg, 0.2 mmol, 1.00 equiv.) at 60 °C for 2 h. After purification by flash column chromatography (hexane/ethyl acetate = 9.8/0.2), the desired compound **3u** was obtained as a White solid (61.5 mg, 73% yield).

Rf = 0.54 (hexane/ethyl acetate = 9/1).

M.P. = 170 – 172 °C

^{1}H NMR ((400 MHz, $CDCl_3$) δ (ppm)): δ 7.64 – 7.46 (m, 4H), 7.43 (dddd, J = 7.8, 7.1, 1.8, 0.9 Hz, 2H), 7.37 – 7.19 (m, 5H), 6.92 (ddd, J = 7.9, 7.0, 0.9 Hz, 1H), 6.68 (ddd, J = 8.4, 7.0, 1.3 Hz, 1H), 5.29 (dt, J = 8.6, 0.8 Hz, 1H), 5.15 (s, 1H), 4.10 (qd, J = 7.1, 2.1 Hz, 2H), 2.97 – 2.83 (m, 3H), 2.77 (dd, J = 9.7, 7.4 Hz, 1H), 1.85 (d, J = 0.9 Hz, 3H), 1.18 (t, J = 7.2 Hz, 3H).

^{13}C {^{1}H} NMR ((101 MHz, $CDCl_3$) δ (ppm)): δ 173.45, 140.22, 134.86, 133.45, 129.28, 129.07, 128.94, 128.91, 128.88, 128.41, 128.06, 128.01, 127.34, 120.75, 118.19, 111.28, 108.78, 62.22, 60.89, 48.26, 47.71, 47.35, 39.42, 14.23, 8.64.

HRMS (ESI) Calcd. for $C_{29}H_{23}NO_2$ $[M+H]^+$, 422.2115, found: 422.2116.

FTIR (cm^{-1}) : 1024, 1103, 1205, 1327, 1450, 1718, 2982.

Isopropyl-10-methyl-6,9-diphenyl-6,7-dihydro-6,8-methanopyrido[1,2-a]indole-8(9*H*)-carboxylate (3v):

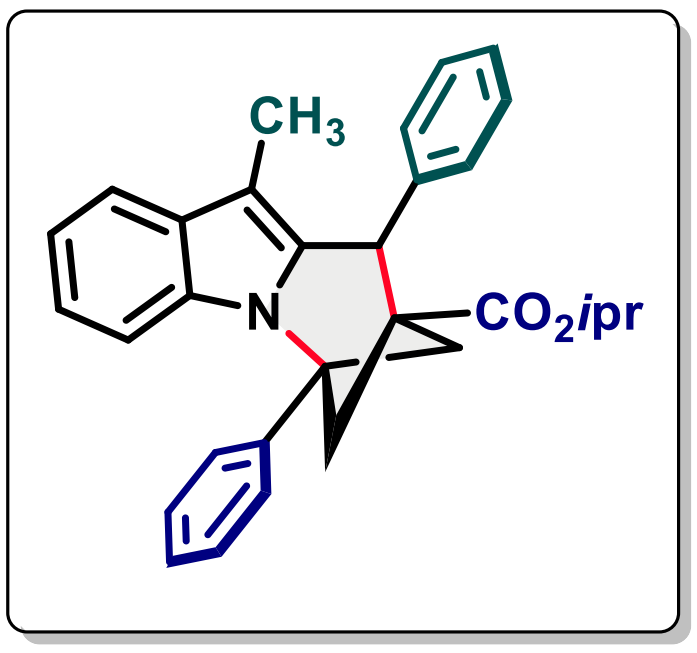

The reaction was performed according to the general procedure GP5 with isopropyl 3-phenylbicyclo[1.1.0]butane-1-carboxylate **1c** (64.8 mg, 0.3 mmol, 1.5 equiv.) and (3-methyl-1*H*-indol-2-yl)(phenyl)methanol **2a** (47.4 mg, 0.2 mmol, 1.00 equiv.) at 60 ºC for 2 h. After purification by flash column chromatography (hexane/ethyl acetate = 9.9/0.1), the desired compound **3v** was obtained as a White solid (69.6 mg, 80% yield).

Rf = 0.63 (hexane/ethyl acetate = 9/1).

M.P. = 155 – 157 ºC

^{1}H NMR ((400 MHz, $CDCl_3$) δ (ppm)): δ 7.65 – 7.49 (m, 4H), 7.48 – 7.41 (m, 2H), 7.34 – 7.27 (m, 5H), 6.94 (ddd, *J* = 7.9, 7.0, 0.9 Hz, 1H), 6.70 (ddd, *J* = 8.4, 7.0, 1.3 Hz, 1H), 5.32 (dt, *J* = 8.6, 0.8 Hz, 1H), 5.17 (s, 1H), 5.00 (p, *J* = 6.3 Hz, 1H), 2.99 – 2.86 (m, 3H), 2.79 (dd, *J* = 9.6, 7.6 Hz, 1H), 1.87 (d, *J* = 0.9 Hz, 3H), 1.27 (d, *J* = 6.3 Hz, 3H), 1.06 (d, *J* = 6.2 Hz, 3H).

^{13}C {^{1}H} NMR ((101 MHz, $CDCl_3$) δ (ppm)): δ 173.03, 140.39, 140.27, 134.87, 133.59, 129.32, 129.07, 128.92, 128.41, 128.12, 128.06, 127.34, 120.76, 118.24, 118.18, 111.29, 108.77, 68.40, 62.16, 48.31, 47.76, 47.45, 39.52, 21.92, 8.67.

HRMS (ESI) Calcd. for $C_{30}H_{29}NO_2$ $[M+H]^+$, 436.2271, found: 436.2274.

FTIR (cm^{-1}) : 1102, 1181, 1217, 1247, 1331, 1450, 1722, 2982.

***tert*-butyl-10-methyl-6,9-diphenyl-6,7-dihydro-6,8-methanopyrido[1,2-a]indole-8(9*H*)-carboxylate (3w):**

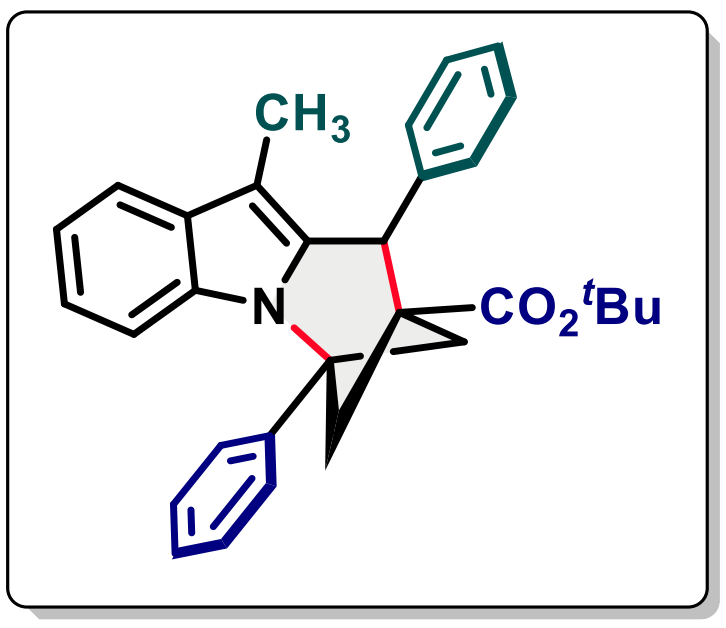

The reaction was performed according to the general procedure GP5 with *tert*-butyl 3-phenylbicyclo[1.1.0]butane-1-carboxylate **1d** (69.0 mg, 0.3 mmol, 1.5 equiv.) and (3-methyl-1*H*-indol-2-yl)(phenyl)methanol **2a** (47.4 mg, 0.2 mmol, 1.00 equiv.) at 60 °C for 2 h. After purification by flash column chromatography (hexane/ethyl acetate = 9.9/0.1), the desired compound **3w** was obtained as a White solid (53.0 mg, 59% yield).

Rf = 0.73 (hexane/ethyl acetate = 9/1).

M.P. = 168 – 170 °C

^{1}H NMR ((400 MHz, $CDCl_3$) δ (ppm)): δ 7.58 (dtdd, *J* = 5.9, 4.1, 2.0, 1.0 Hz, 1H), 7.56 – 7.51 (m, 2H), 7.51 – 7.45 (m, 1H), 7.42 (ddd, *J* = 8.0, 1.4, 0.8 Hz, 2H), 7.29 – 7.26 (m, 5H), 6.90 (ddd, *J* = 7.9, 7.0, 0.9 Hz, 1H), 6.66 (ddd, *J* = 8.5, 7.0, 1.3 Hz, 1H), 5.28 (dt, *J* = 8.6, 0.8 Hz, 1H), 5.10 (s, 1H), 2.92 – 2.77 (m, 3H), 2.77 – 2.70 (m, 1H), 1.82 (d, *J* = 0.9 Hz, 3H), 1.37 (s, 9H).

^{13}C {^{1}H} NMR ((101 MHz, $CDCl_3$) δ (ppm)): δ 172.62, 140.46, 140.33, 134.78, 133.80, 129.28, 129.20, 129.01, 128.91, 128.85, 128.26, 128.07, 128.05, 127.24, 120.63, 118.15, 111.23, 108.65, 81.25, 61.97, 48.70, 47.61, 39.50, 28.03, 8.60.

HRMS (ESI) Calcd. for $C_{31}H_{31}NO_2$ $[M+H]^+$, 450.2428, found: 450.2424.

FTIR (cm^{-1}) : 1103, 1160, 1251, 1329, 1449, 1730, 2927.

Phenyl-10-methyl-6,9-diphenyl-6,7-dihydro-6,8-methanopyrido[1,2-a]indole-8(9*H*)-carboxylate (3x):

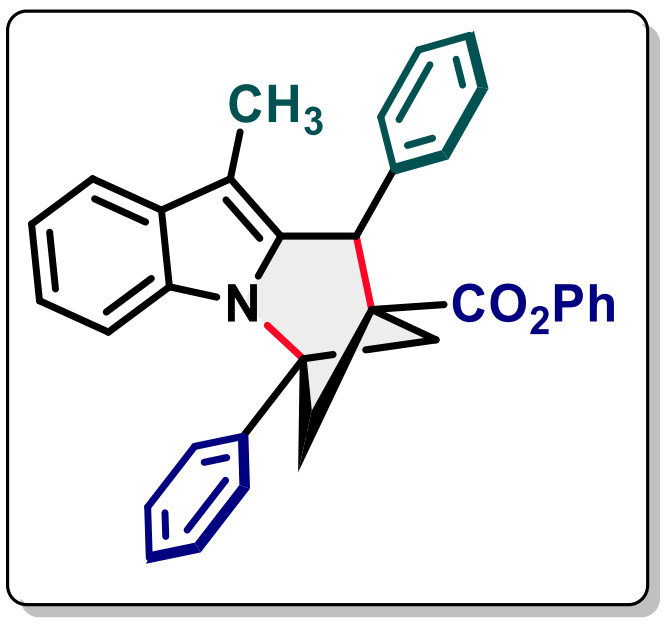

The reaction was performed according to the general procedure GP5 with phenyl 3-phenylbicyclo[1.1.0]butane-1-carboxylate **1e** (75.0 mg, 0.3 mmol, 1.5 equiv.) and (3-methyl-1*H*-indol-2-yl)(phenyl)methanol **2a** (47.4 mg, 0.2 mmol, 1.00 equiv.) at 60 °C for 2 h. After purification by flash column chromatography (hexane/ethyl acetate = 9.7/0.3), the desired compound **3x** was obtained as a White solid (71.3 mg, 76% yield).

Rf = 0.60 (hexane/ethyl acetate = 9/1).

M.P. = 176 – 178 °C

^{1}H NMR ((400 MHz, $CDCl_3$) δ (ppm)): δ 7.67 – 7.59 (m, 1H), 7.59 – 7.50 (m, 3H), 7.49 – 7.43 (m, 2H), 7.39 – 7.29 (m, 8H), 7.25 – 7.18 (m, 1H), 6.98 – 6.85 (m, 3H), 6.70 (ddd, *J* = 8.5, 7.0, 1.3 Hz, 1H), 5.43 – 5.24 (m, 2H), 3.17 – 3.03 (m, 3H), 2.92 (ddd, *J* = 9.7, 6.9, 0.9 Hz, 1H), 1.86 (d, *J* = 0.9 Hz, 3H).

^{13}C {^{1}H} NMR ((101 MHz, $CDCl_3$) δ (ppm)): δ 171.91, 150.43, 140.08, 134.89, 133.11, 129.57, 129.29, 129.22, 129.13, 129.04, 128.99, 128.67, 128.45, 128.08, 128.01, 127.67, 126.11, 121.46, 120.91, 118.28, 111.29, 109.01, 62.27, 48.49, 47.91, 47.64, 39.70, 8.68.

HRMS (ESI) Calcd. for $C_{33}H_{27}NO_2$ $[M+H]^+$, 470.2115, found: 470.2108.

FTIR (cm^{-1}) : 1082, 1185, 1329, 1448, 1489, 1750, 2924.

Benzyl-10-methyl-6,9-diphenyl-6,7-dihydro-6,8-methanopyrido[1,2-a]indole-8(9*H*)-carboxylate (3y):

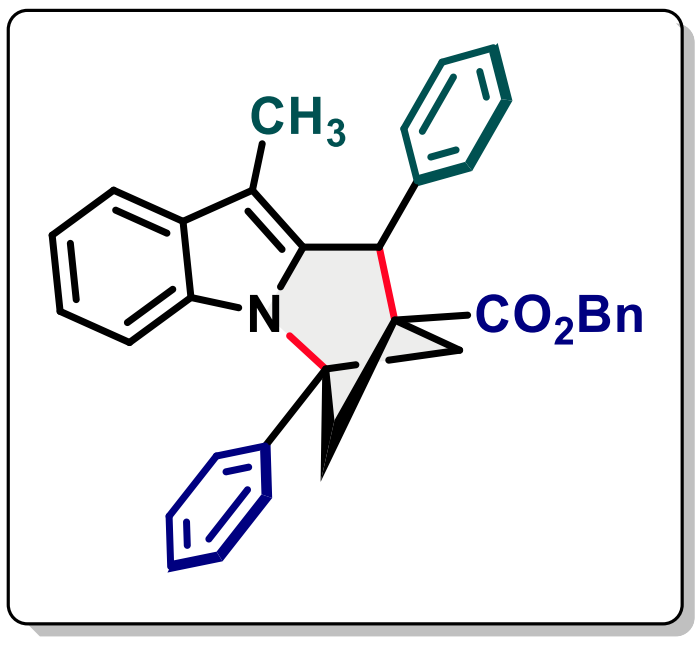

The reaction was performed according to the general procedure GP5 with benzyl 3-phenylbicyclo[1.1.0]butane-1-carboxylate **1f** (79.2 mg, 0.3 mmol, 1.5 equiv.) and (3-methyl-1*H*-indol-2-yl)(phenyl)methanol **2a** (47.4 mg, 0.2 mmol, 1.00 equiv.) at 60 ºC for 2 h. After purification by flash column chromatography (hexane/ethyl acetate = 9.7/0.3), the desired compound **3y** was obtained as a White solid (70.5 mg, 73% yield).

Rf = 0.58 (hexane/ethyl acetate = 9/1).
M.P. = 172 – 174 ºC
^{1}H NMR ((400 MHz, $CDCl_3$) δ (ppm)): δ 7.58 – 7.47 (m, 4H), 7.45 – 7.39 (m, 2H), 7.38 – 7.31 (m, 3H), 7.27 – 7.20 (m, 5H), 7.19 – 7.13 (m, 2H), 6.92 (ddd, *J* = 7.9, 7.0, 0.9 Hz, 1H), 6.68 (ddd, *J* = 8.4, 7.0, 1.3 Hz, 1H), 5.29 (dt, *J* = 8.6, 0.8 Hz, 1H), 5.23 – 4.89 (m, 3H), 2.99 – 2.83 (m, 3H), 1.83 (d, *J* = 0.9 Hz, 3H).

^{13}C {^{1}H} NMR ((101 MHz, $CDCl_3$) δ (ppm)): δ 173.25, 140.18, 140.08, 135.55, 134.87, 133.31, 129.27, 129.09, 128.95, 128.86, 128.68, 128.57, 128.49, 128.47, 128.05, 128.01, 127.36, 120.78, 118.21 (d, *J* = 4.3 Hz), 111.28, 108.81, 66.68, 62.26, 48.36, 47.76, 47.32, 39.47, 8.64.

HRMS (ESI) Calcd. for $C_{34}H_{29}NO_2$ $[M+H]^+$, 484.2271, found: 484.2267.

FTIR (cm^{-1}) : 1009, 1105, 1181, 1207, 1242, 1331, 1451, 1742, 2942.

4-Methoxyphenyl-10-methyl-6,9-diphenyl-6,7-dihydro-6,8-methanopyrido[1,2-a]indole-8(9*H*)-carboxylate (3z):

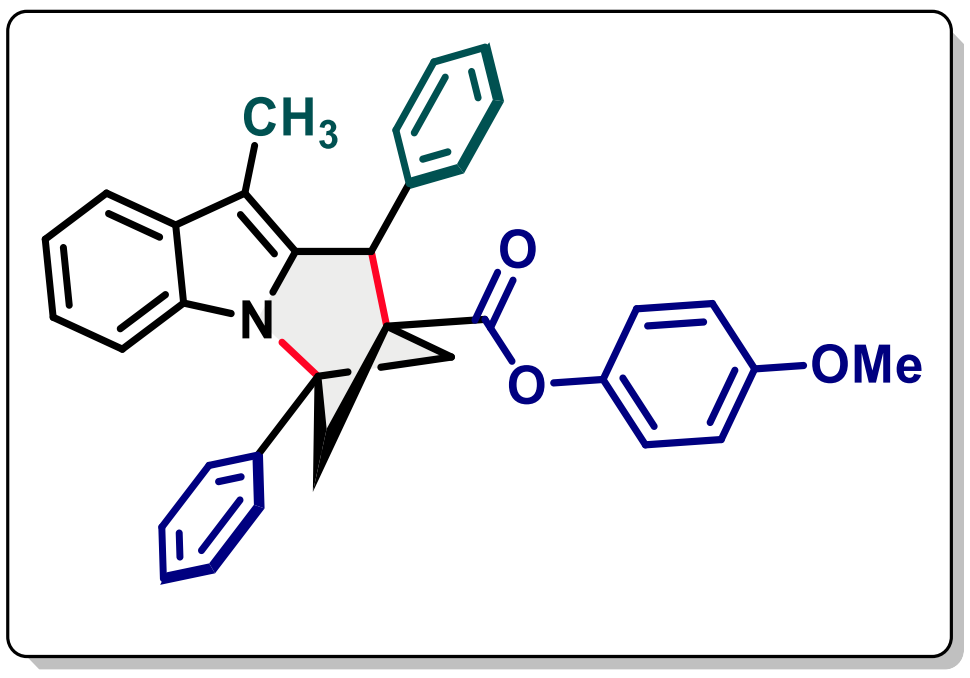

The reaction was performed according to the general procedure GP5 with 4-methoxyphenyl 3-phenylbicyclo[1.1.0]butane-1-carboxylate **1g** (84.0 mg, 0.3 mmol, 1.5 equiv.) and (3-methyl-1*H*-indol-2-yl)(phenyl)methanol **2a** (47.4 mg, 0.2 mmol, 1.00 equiv.) at 60 ºC for 2 h. After purification by flash column chromatography (hexane/ethyl acetate = 9.6/0.4), the desired compound **3z** was obtained as a White solid (77.9 mg, 78% yield).

Rf = 0.42 (hexane/ethyl acetate = 9/1).
M.P. = 171 – 173 ºC
^{1}H NMR ((400 MHz, $CDCl_3$) δ (ppm)): δ 7.69 – 7.61 (m, 1H), 7.60 – 7.51 (m, 3H), 7.50 – 7.44 (m, 2H), 7.41 – 7.32 (m, 5H), 6.96 (ddd, *J* = 7.9, 7.0, 0.9 Hz, 1H), 6.91 – 6.79 (m, 4H), 6.72 (ddd, *J* = 8.4, 7.0, 1.3 Hz, 1H), 5.35 (dt, *J* = 8.6, 0.9 Hz, 1H), 5.31 (d, *J* = 1.1 Hz, 1H), 3.81 (s, 3H), 3.14 – 3.02 (m, 3H), 2.93 (ddd, *J* = 9.6, 6.9, 0.8 Hz, 1H), 1.89 (d, *J* = 0.9 Hz, 3H).

^{13}C {^{1}H} NMR ((101 MHz, $CDCl_3$) δ (ppm)): δ 172.28, 157.48, 143.91, 140.16, 140.14, 134.94, 133.17, 129.33, 129.22, 129.16, 129.05, 128.99, 128.67, 128.11, 128.04, 127.66, 122.22, 120.93, 118.31, 118.30, 114.59, 111.32, 109.00, 62.30, 55.70, 48.49, 47.96, 47.66, 39.74, 8.70.

HRMS (ESI) Calcd. for $C_{34}H_{29}NO_3$ $[M+H]^+$, 500.2220, found: 500.2217.

FTIR (cm^{-1}) : 1027, 1086, 1193, 1246, 1330, 1450, 1502, 1741, 2934.

***N*-methoxy-*N*,10-dimethyl-6,9-diphenyl-6,7-dihydro-6,8-methanopyrido[1,2-a]indole-8(9*H*)-carboxamide (3aa):**

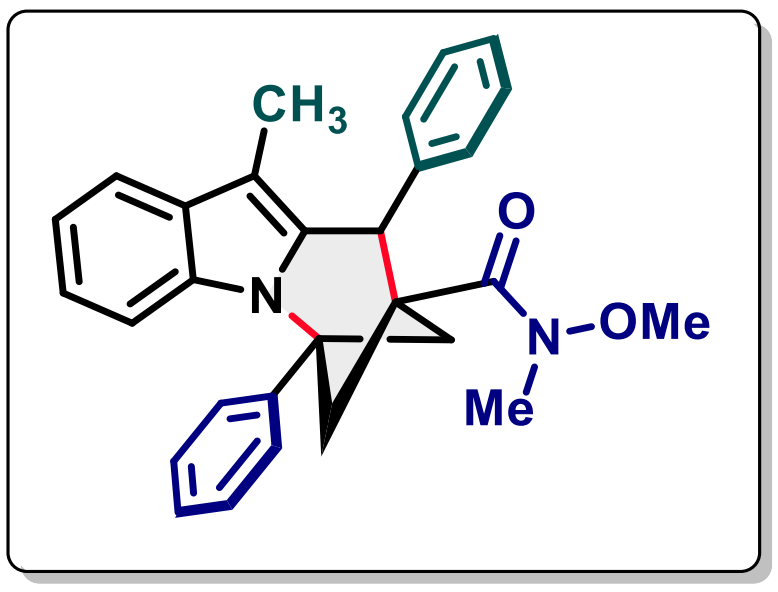

The reaction was performed according to the general procedure GP5 with *N*-methoxy-*N*-methyl-3-phenylbicyclo[1.1.0]butane-1-carboxamide **1r** (65.1 mg, 0.3 mmol, 1.5 equiv.) and (3-methyl-1*H*-indol-2-yl)(phenyl)methanol **2a** (47.4 mg, 0.2 mmol, 1.00 equiv.) at 60 ºC for 2 h. After purification by flash column chromatography (hexane/ethyl acetate = 8.5/1.5), the desired compound **3aa** was obtained as a White solid (39.3 mg, 45% yield).

Rf = 0.50 (hexane/ethyl acetate = 7/3).
M.P. = 202 – 204 ºC
^{1}H NMR ((400 MHz, $CDCl_3$) δ (ppm)): δ 7.61 – 7.47 (m, 4H), 7.49 – 7.39 (m, 3H), 7.36 – 7.23 (m, 3H), 7.19 (d, *J* = 7.3 Hz, 2H), 6.91 (ddd, *J* = 7.9, 7.0, 0.9 Hz, 1H), 6.67 (ddd, *J* = 8.5, 7.0, 1.3 Hz, 1H), 5.29 (dt, *J* = 8.6, 0.9 Hz, 2H), 3.52 (s, 3H), 3.18 (dt, *J* = 10.2, 4.9 Hz, 3H), 3.03 – 2.91 (m, 2H), 2.91 – 2.81 (m, 2H), 1.83 (d, *J* = 0.9 Hz, 3H).

^{13}C {^{1}H} NMR ((101 MHz, $CDCl_3$) δ (ppm)): δ 174.38, 140.43, 134.74, 133.50, 129.62, 129.22, 128.96, 128.80, 128.49, 128.08, 120.63, 118.13, 118.09, 115.47, 111.26, 108.35, 62.12, 61.17, 49.21, 47.55, 40.70, 22.47, 14.21, 8.78.

HRMS (ESI) Calcd. for $C_{29}H_{28}N_2O_2$ $[M+H]^+$, 437.2224, found: 437.2206.
FTIR (cm^{-1}) : 1176, 1328, 1375, 1449, 1659, 2932.
Methyl-10-methyl-6-(naphthalen-2-yl)-9-phenyl-6,7-dihydro-6,8-methanopyrido[1,2-a]indole-8(9*H*)-carboxylate (3ab):

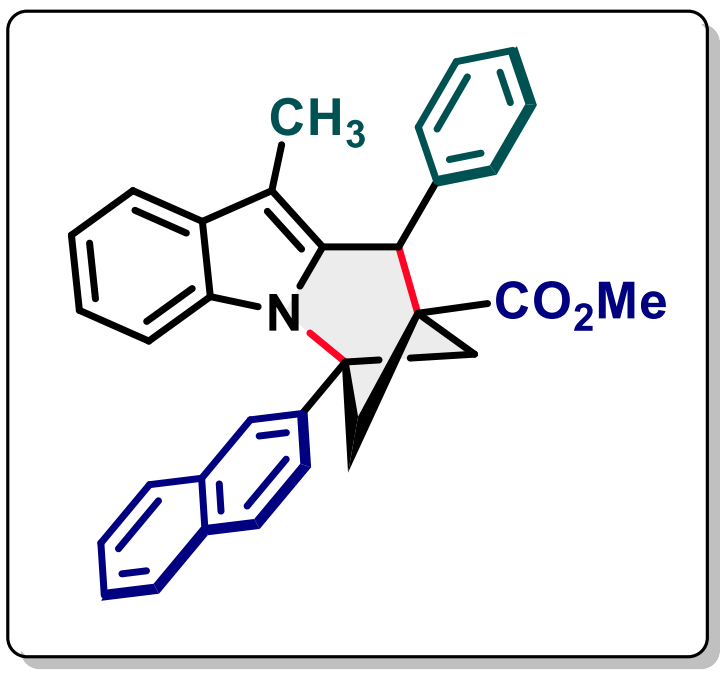

The reaction was performed according to the general procedure GP5 with methyl 3-(naphthalen-2-yl)bicyclo[1.1.0]butane-1-carboxylate **1h** (71.4 mg, 0.3 mmol, 1.5 equiv.) and (3-methyl-1*H*-indol-2-yl)(phenyl)methanol **2a** (47.4 mg, 0.2 mmol, 1.00 equiv.) at 60 ºC for 2 h. After purification by flash column chromatography (hexane/ethyl acetate = 9.7/0.3), the desired compound **3ab** was obtained as a White solid (47.5 mg, 52% yield).

Rf = 0.41 (hexane/ethyl acetate = 9/1).
M.P. = 156 – 158 ºC
^{1}H NMR ((400 MHz, $CDCl_3$) δ (ppm)): δ 8.10 – 7.88 (m, 4H), 7.74 – 7.53 (m, 3H), 7.47 (ddd, *J* = 7.9, 1.3, 0.8 Hz, 1H), 7.43 – 7.22 (m, 5H), 6.92 (dddd, *J* = 7.9, 7.0, 1.6, 0.9 Hz, 1H), 6.59 (dddd, *J* = 8.5,

6.9, 5.3, 1.3 Hz, 1H), 5.38 (ddt, J = 8.6, 1.7, 0.9 Hz, 1H), 5.25 (s, 1H), 3.69 (d, J = 2.8 Hz, 3H), 3.14 – 3.00 (m, 2H), 2.97 – 2.87 (m, 2H), 1.91 (dd, J = 5.7, 0.9 Hz, 3H).

^{13}C {^{1}H} NMR ((101 MHz, $CDCl_3$) δ (ppm)): δ 173.84, 140.29, 140.20, 137.62, 135.13, 134.97, 133.45, 133.38, 133.22, 133.17, 129.32, 128.14, 127.47, 127.17, 127.09, 126.97, 126.93, 125.74, 125.63, 120.90, 118.25, 111.46, 108.94, 62.46, 51.94, 48.48, 47.74, 47.21, 40.05, 39.36, 8.76, 8.69.

HRMS (ESI) Calcd. for $C_{32}H_{27}NO_2$ $[M+H]^+$, 458.2115, found: 458.2114.

FTIR (cm^{-1}) : 1021, 1097, 1209, 1247, 1326, 1450, 1731, 2365, 2922.

Methyl-6-(4-(*tert*-butyl)phenyl)-10-methyl-9-phenyl-6,7-dihydro-6,8-methanopyrido[1,2-a]indol--8(9*H*)-carboxylate (3ac):

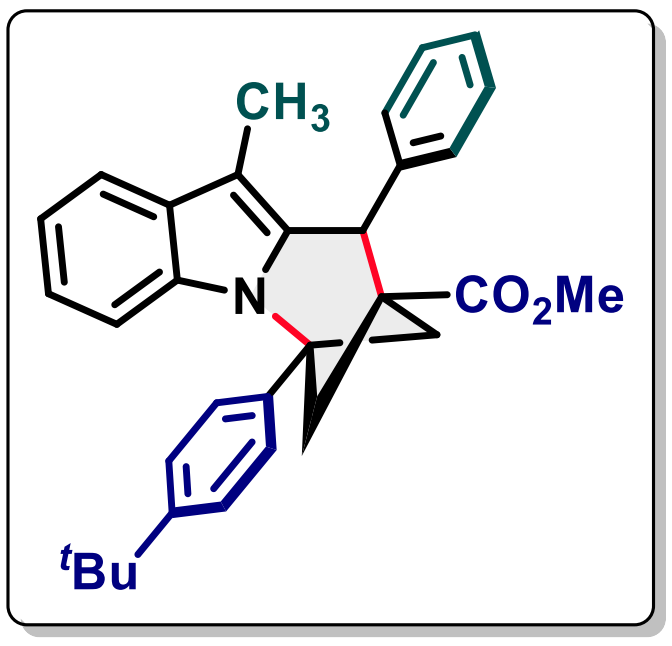

The reaction was performed according to the general procedure GP5 with methyl 3-(4-(*tert*-butyl)phenyl)bicyclo[1.1.0]butane-1-carboxylate **1i** (73.2 mg, 0.3 mmol, 1.5 equiv.) and (3-methyl-1*H*-indol-2-yl)(phenyl)methanol **2a** (47.4 mg, 0.2 mmol, 1.00 equiv.) at 60 °C for 2 h. After purification by flash column chromatography (hexane/ethyl acetate = 9.85/0.15), the desired compound **3ac** was obtained as a White solid (55.6 mg, 60% yield).

Rf = 0.57 (hexane/ethyl acetate = 9/1).

M.P. = 167 – 169 °C

^{1}H NMR ((400 MHz, $CDCl_3$) δ (ppm)): δ 7.60 – 7.47 (m, 3H), 7.47 – 7.39 (m, 1H), 7.36 – 7.27 (m, 4H), 7.27 – 7.21 (m, 2H), 6.92 (ddd, J = 7.9, 7.0, 0.9 Hz, 1H), 6.67 (ddd, J = 8.5, 7.0, 1.3 Hz, 1H), 5.29 (dt, J = 8.6, 0.8 Hz, 1H), 5.16 (s, 1H), 3.65 (s, 3H), 2.98 – 2.84 (m, 3H), 2.77 (ddd, J = 9.7, 6.6, 1.1 Hz, 1H), 1.86 (d, J = 0.9 Hz, 3H), 1.45 (s, 9H).

^{13}C {^{1}H} NMR ((101 MHz, $CDCl_3$) δ (ppm)): δ 173.87, 152.44, 140.26, 137.20, 134.88, 133.34, 129.22, 128.81, 128.47, 127.71, 127.64, 127.37, 125.77, 125.74, 120.70, 118.15 (d, J = 3.0 Hz), 111.42, 108.69, 62.05, 51.86, 48.37, 47.73, 47.30, 39.34, 34.97, 31.58, 8.67.

HRMS (ESI) Calcd. for $C_{32}H_{33}NO_2$ $[M+H]^+$, 464.2584, found: 464.2583.

FTIR (cm^{-1}) : 1022, 1094, 1213, 1330, 1451, 1742, 2950.

Methyl-6-([1,1'-biphenyl]-4-yl)-10-methyl-9-phenyl-6,7-dihydro-6,8-methanopyrido[1,2-a]indole-8(9*H*)-carboxylate (3ad):

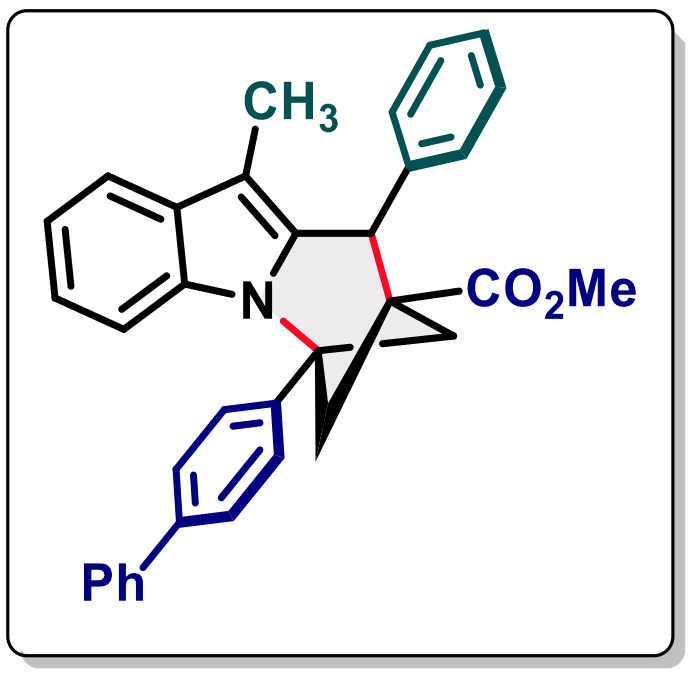

The reaction was performed according to the general procedure GP5 with methyl 3-([1,1'-biphenyl]-4-yl)bicyclo[1.1.0]butane-1-carboxylate **1j** (79.2 mg, 0.3 mmol, 1.5 equiv.) and (3-methyl-1*H*-indol-2-yl)(phenyl)methanol **2a** (47.4 mg, 0.2 mmol, 1.00 equiv.) at 60 °C for 2 h. After purification by flash column chromatography (hexane/ethyl acetate = 9.7/0.3), the desired compound **3ad** was obtained as a White solid (65.7 mg, 68% yield).

Rf = 0.40 (hexane/ethyl acetate = 9/1).
M.P. = 186 – 188 °C
^{1}H NMR ((400 MHz, $CDCl_3$) δ (ppm)): δ 7.84 – 7.72 (m, 4H), 7.66 (ddd, *J* = 8.0, 2.1, 0.6 Hz, 1H), 7.57 – 7.39 (m, 5H), 7.37 – 7.29 (m, 3H), 7.28 – 7.21 (m, 2H), 6.94 (ddd, *J* = 7.9, 7.0, 0.9 Hz, 1H), 6.71 (ddd, *J* = 8.4, 7.0, 1.3 Hz, 1H), 5.52 (dt, *J* = 8.6, 0.9 Hz, 1H), 5.19 (s, 1H), 3.67 (s, 3H), 3.00 – 2.88 (m, 3H), 2.81 (dd, *J* = 9.6, 7.0 Hz, 1H), 1.88 (d, *J* = 0.9 Hz, 3H).

^{13}C {^{1}H} NMR ((101 MHz, $CDCl_3$) δ (ppm)): δ 173.80, 141.84, 140.44, 140.19, 139.17, 134.94, 133.32, 129.32, 129.09, 128.79, 128.51, 128.46, 127.90, 127.57, 127.49, 127.43, 127.33, 120.90, 118.28, 111.38, 108.88, 62.11, 51.91, 48.38, 47.73, 47.42, 39.52, 8.68.

HRMS (ESI) Calcd. for $C_{34}H_{29}NO_2$ $[M+H]^+$, 484.2271, found: 484.2269.

FTIR (cm^{-1}) : 1102, 1210, 1328, 1448, 1724, 2924.

Methyl 6-(3-methoxyphenyl)-10-methyl-9-phenyl-6,7-dihydro-6,8-methanopyrido [1,2-a]indole-8(9*H*)-carboxylate (3ae):

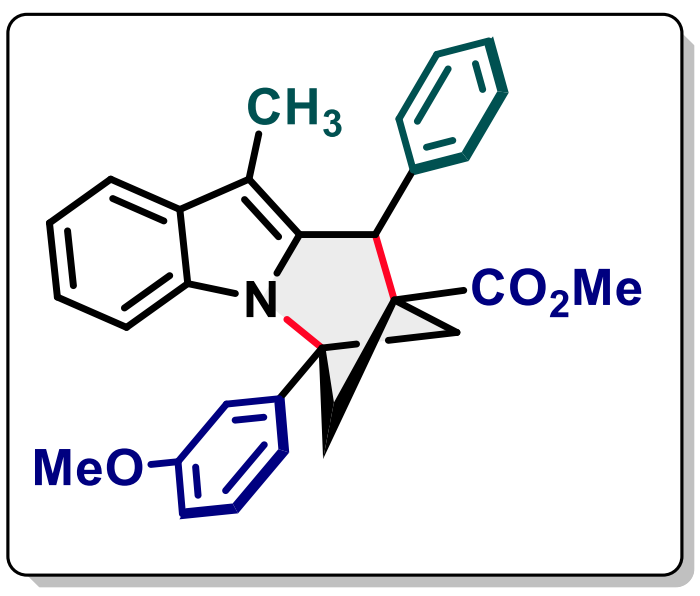

The reaction was performed according to the general procedure GP5 with methyl 3-(3-methoxyphenyl)bicyclo[1.1.0]butane-1-carboxylate **1k** (65.4 mg, 0.3 mmol, 1.5 equiv.) and (3-methyl-1*H*-indol-2-yl)(phenyl)methanol **2a** (47.4 mg, 0.2 mmol, 1.00 equiv.) at 60 ºC for 2 h. After purification by flash column chromatography (hexane/ethyl acetate = 9.2/0.8), the desired compound **3ae** was obtained as a colourless liquid (79.6 mg, 91% yield).

Rf = 0.31 (hexane/ethyl acetate = 9/1).

^{1}H NMR ((400 MHz, $CDCl_3$) δ (ppm)): δ 7.50 – 7.35 (m, 2H), 7.35 – 7.14 (m, 6H), 7.13 – 6.98 (m, 2H), 6.94 (tt, *J* = 6.6, 1.0 Hz, 1H), 6.73 (ddt, *J* = 8.5, 7.1, 1.4 Hz, 1H), 5.48 – 5.40 (m, 1H), 5.16 (s, 1H), 3.84 (dd, *J* = 17.5, 0.9 Hz, 3H), 3.65 (dd, *J* = 1.5, 0.9 Hz, 3H), 2.96 – 2.83 (m, 3H), 2.80 – 2.70 (m, 1H), 1.85 (d, *J* = 0.9 Hz, 3H).

^{13}C {^{1}H} NMR ((101 MHz, $CDCl_3$) δ (ppm)): δ 173.80, 160.02, 141.63, 140.17, 134.82, 133.24, 130.04, 129.97, 129.21, 128.77, 128.49, 127.40, 120.91, 120.42, 118.22, 114.83, 113.47, 113.18, 111.44, 108.84, 62.27, 55.52, 51.89, 48.20, 47.67, 47.37, 47.24, 39.38, 8.65.

HRMS (ESI) Calcd. for $C_{29}H_{27}NO_3$ $[M+H]^+$, 438.2064, found: 438.2059.

FTIR (cm^{-1}) : 1044, 1104, 1176, 1204, 1240, 1331, 1451, 1602, 1733, 2947.

Methyl-6-(4-fluorophenyl)-10-methyl-9-phenyl-6,7-dihydro-6,8-methanopyrido[1,2-a]indole-8(9*H*)-carboxylate (3af):

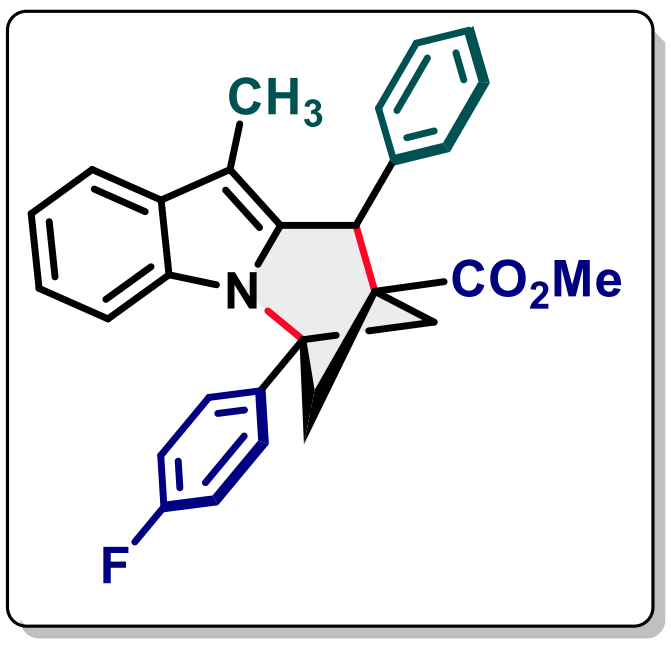

The reaction was performed according to the general procedure GP5 with methyl 3-(4-fluorophenyl)bicyclo[1.1.0]butane-1-carboxylate **1l** (61.8 mg, 0.3 mmol, 1.5 equiv.) and (3-methyl-1*H*-indol-2-yl)(phenyl)methanol **2a** (47.4 mg, 0.2 mmol, 1.0 equiv.) at 60 ºC for 2 h. After purification by flash column chromatography (hexane/ethyl acetate = 9.7/0.3), the desired compound **3af** was obtained as a White solid (68.9 mg, 81% yield).

Rf = 0.41 (hexane/ethyl acetate = 9/1).

M.P. = 180 – 182 ºC

^{1}H NMR ((400 MHz, $CDCl_3$) δ (ppm)): δ 7.61 – 7.52 (m, 1H), 7.47 (ddd, *J* = 7.9, 1.3, 0.7 Hz, 1H), 7.39 (ddd, *J* = 8.5, 5.2, 2.3 Hz, 1H), 7.36 – 7.27 (m, 3H), 6.96 (ddd, *J* = 8.0, 7.0, 0.9 Hz, 1H), 6.75 (ddd, *J* =

8.5, 7.0, 1.3 Hz, 1H), 5.38 (dt, J = 8.6, 0.9 Hz, 1H), 5.18 (s, 1H), 3.67 (s, 3H), 2.95 – 2.85 (m, 3H), 2.77 (dd, J = 9.6, 7.4 Hz, 1H), 1.87 (d, J = 0.9 Hz, 3H).

^{13}C {^{1}H} NMR ((101 MHz, $CDCl_3$) δ (ppm)): δ 173.70, 163.12 (d, J = 248.6 Hz), 140.07, 136.28 (d, J = 3.4 Hz), 134.84, 133.28, 129.92, 129.77, 128.75, 128.54, 127.48, 120.96, 118.38 (d, J = 4.8 Hz), 116.12 (d, J = 7.9 Hz), 115.94 , 111.08, 109.00, 61.76, 51.94, 48.25, 47.68, 47.36, 39.52, 8.67.

^{19}F NMR (376 MHz, $CDCl_3$**)** δ -111.83.

HRMS (ESI) Calcd. for $C_{28}H_{24}FNO_2$ $[M+H]^+$, 426.1864, found: 426.1856.

FTIR (cm^{-1}): 1105, 1214, 1329, 1448, 1738, 2953.

Methyl-6-(4-chlorophenyl)-10-methyl-9-phenyl-6,7-dihydro-6,8-methanopyrido[1,2-a]indole-8(9*H*)-carboxylate (3ag):

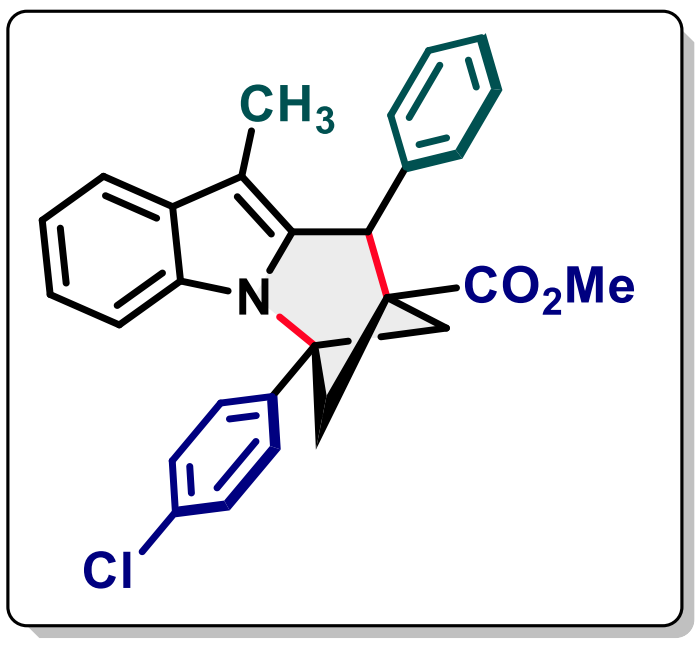

The reaction was performed according to the general procedure GP5 with methyl 3-(4-chlorophenyl)bicyclo[1.1.0]butane-1-carboxylate **1m** (66.6 mg, 0.3 mmol, 1.5 equiv.) and (3-methyl-1*H*-indol-2-yl)(phenyl)methanol **2a** (47.4 mg, 0.2 mmol, 1.00 equiv.) at 60 ºC for 2 h. After purification by flash column chromatography (hexane/ethyl acetate = 9.7/0.3), the desired compound **3ag** was obtained as a White solid (67.9 mg, 77% yield).

Rf = 0.41 (hexane/ethyl acetate = 9/1).

M.P. = 207 – 209 ºC

^{1}H NMR ((400 MHz, $CDCl_3$) δ (ppm)): δ 7.55 – 7.41 (m, 4H), 7.36 – 7.25 (m, 4H), 7.23 – 7.13 (m, 2H), 6.94 (ddd, J = 7.9, 7.0, 0.9 Hz, 1H), 6.74 (ddd, J = 8.5, 7.0, 1.3 Hz, 1H), 5.42 (dt, J = 8.6, 0.8 Hz, 1H), 5.14 (s, 1H), 3.64 (s, 3H), 2.93 – 2.78 (m, 3H), 2.74 (dd, J = 9.6, 7.6 Hz, 1H), 1.84 (d, J = 0.9 Hz, 3H).

^{13}C {^{1}H} NMR ((101 MHz, $CDCl_3$) δ (ppm)): δ 173.61, 139.99, 138.77, 135.05, 134.80, 133.20, 129.46, 129.41, 129.34, 129.24, 129.16, 128.71, 128.51, 127.46, 121.00, 118.39, 111.07, 109.06, 61.75, 51.93, 48.24, 47.62, 47.30, 39.48, 8.61.

HRMS (ESI) Calcd. for $C_{28}H_{24}ClNO_2$ $[M+H]^+$, 442.1568, found: 442.1569.

FTIR (cm^{-1}) : 1011, 1083, 1221, 1328, 1449, 1743, 2943.

Methyl-6-(4-chlorophenyl)-10-methyl-9-phenyl-6,7-dihydro-6,8-methanopyrido[1,2-a]indole-8(9*H*)-carboxylate (3ai):

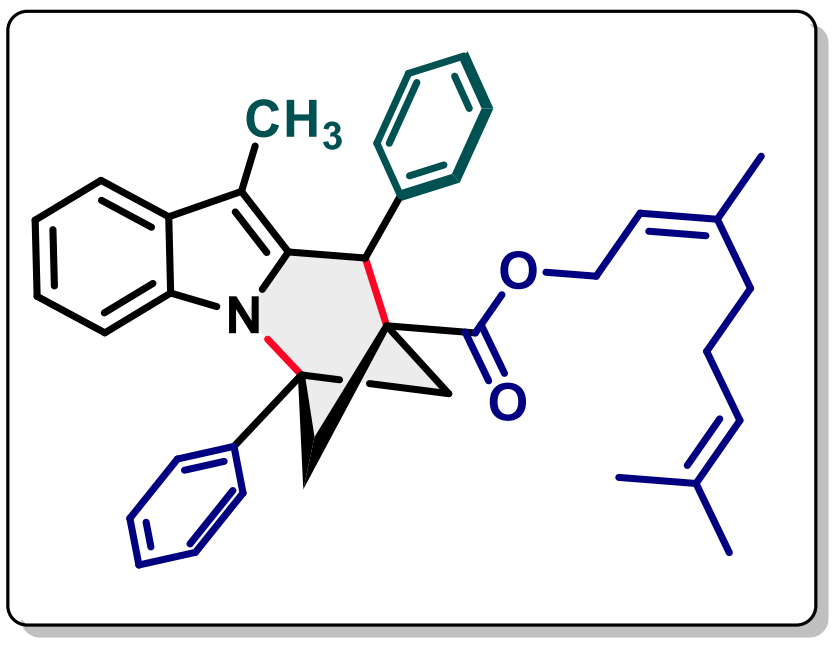

The reaction was performed according to the general procedure GP5 with (*Z*)-3,7-dimethylocta-2,6-dien-1-yl 3-phenylbicyclo[1.1.0]butane-1-carboxylate **1o** (93.0 mg, 0.3 mmol, 1.5 equiv.) and (3-methyl-1*H*-indol-2-yl)(phenyl)methanol **2a** (47.4 mg, 0.2 mmol, 1.00 equiv.) at 60 ºC for 2 h. After purification by flash column chromatography (hexane/ethyl acetate = 9.9/0.1), the desired compound **3ah** was obtained as a colourless liquid (67.7 mg, 64% yield).

Rf = 0.75 (hexane/ethyl acetate = 9/1).

M.P. = 98 – 100 ºC

^{1}H NMR ((400 MHz, $CDCl_3$) δ (ppm)): δ 7.62 – 7.44 (m, 4fH), 7.41 (tt, *J* = 7.5, 1.0 Hz, 2H), 7.32 – 7.19 (m, 5H), 6.91 (ddd, *J* = 7.9, 7.0, 0.9 Hz, 1H), 6.66 (ddd, *J* = 8.5, 7.0, 1.3 Hz, 1H), 5.31 – 5.20 (m, 2H), 5.15 (s, 1H), 5.11 – 5.03 (m, 1H), 4.59 (ddd, *J* = 12.3, 7.3, 1.1 Hz, 1H), 4.49 (ddd, *J* = 12.3, 7.3, 1.1 Hz, 1H), 2.95 – 2.82 (m, 3H), 2.75 (dd, *J* = 9.6, 7.0 Hz, 1H), 2.16 – 2.03 (m, 4H), 1.83 (d, *J* = 0.9 Hz, 3H), 1.76 (q, *J* = 1.2 Hz, 3H), 1.67 (d, *J* = 1.3 Hz, 3H), 1.59 (d, *J* = 1.4 Hz, 3H).

^{13}C {^{1}H} NMR ((101 MHz, $CDCl_3$) δ (ppm)): δ 173.35, 142.89, 140.15, 134.85, 133.47, 129.27, 129.03, 128.94, 128.85, 128.39, 128.04, 128.00, 127.30, 123.65, 120.72, 119.00, 118.16 (d, *J* = 5.2 Hz), 111.27, 108.74, 62.25, 61.49, 48.26, 47.64, 47.32, 39.39, 32.24, 26.76, 25.80, 23.62, 17.80, 8.61, 1.12.

HRMS (ESI) Calcd. for $C_{28}H_{24}ClNO_2$ $[M+H]^+$, 442.1568, found: 442.1569.

FTIR (cm^{-1}) : 1103, 1204, 1330, 1452, 1725, 2928, 2952.

Benzo[d][1,3]dioxol-5-yl-10-methyl-6,9-diphenyl-6,7-dihydro-6,8-methanopyrido[1,2-a]indole-8(9*H*)-carboxylate (3aj):

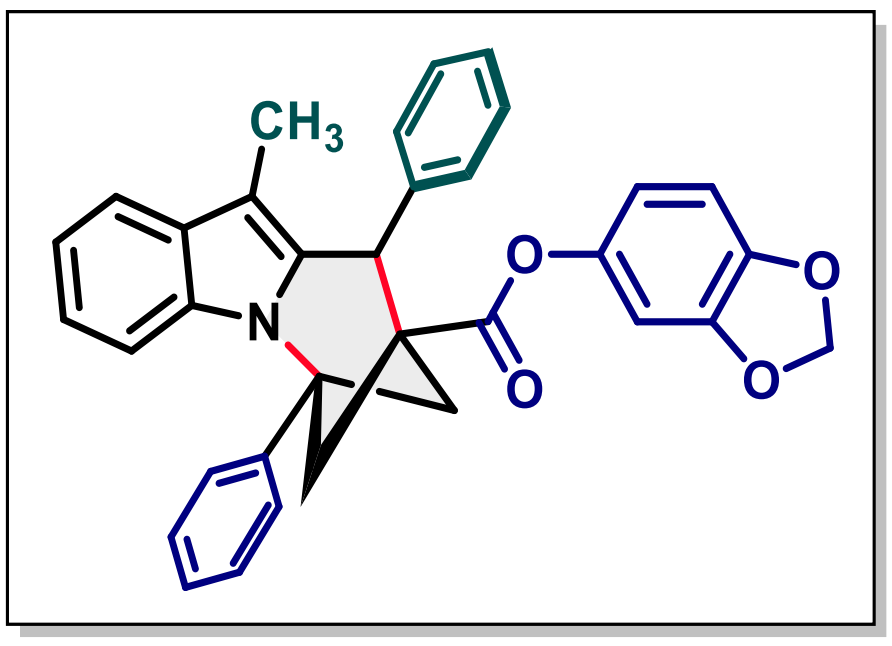

The reaction was performed according to the general procedure GP5 with benzo[d][1,3]dioxol-5-yl-3-phenylbicyclo[1.1.0]butane-1-carboxylate **1p** (88.2 mg, 0.3 mmol, 1.5 equiv.) and (3-methyl-1*H*-indol-2-yl)(phenyl)methanol **2a** (47.4 mg, 0.2 mmol, 1.00 equiv.) at 60 ºC for 2 h. After purification by flash column chromatography (hexane/ethyl acetate = 9.8/0.2), the desired compound **3ai** was obtained as a White solid (78.0 mg, 76% yield).

Rf = 0.36 (hexane/ethyl acetate = 9/1)

M.P. = 180 – 182 ºC

^{1}H NMR ((400 MHz, $CDCl_3$) δ (ppm)): δ 7.66 – 7.50 (m, 5H), 7.49 – 7.44 (m, 2H), 7.40 – 7.34 (m, 6H), 6.95 (ddd, *J* = 7.9, 7.0, 0.9 Hz, 1H), 6.80 – 6.67 (m, 2H), 6.39 (d, *J* = 2.3 Hz, 1H), 6.34 (dd, *J* = 8.4, 2.4 Hz, 1H), 6.04 – 5.93 (m, 2H), 5.33 (d, *J* = 8.6 Hz, 1H), 5.28 (s, 1H), 3.11 – 3.03 (m, 3H), 2.92 (dd, *J* = 9.6, 7.1 Hz, 1H), 1.87 (d, *J* = 0.9 Hz, 3H).

^{13}C {^{1}H} NMR ((101 MHz, $CDCl_3$) δ (ppm)): δ 172.21, 148.10, 145.59, 144.70, 140.10, 134.93, 133.06, 129.32, 129.22, 129.13, 129.04, 128.70, 128.46, 128.08, 127.72, 126.12, 120.94, 118.31, 113.84, 111.30, 109.03, 108.08, 103.62, 101.85, 62.28, 48.48, 47.97, 47.65, 39.75, 35.80, 8.67.

HRMS (ESI) Calcd. for $C_{34}H_{27}NO_4$ $[M+H]^+$, 514.2013, found: 514.2014.

FTIR (cm^{-1}) : 1036, 1121, 1174, 1246, 1483, 1750, 2924.

(1R,2R,5R)-2-isopropyl-5-methylcyclohexyl-10-methyl-6,9-diphenyl-6,7-dihydro-6,8-methano pyrido[1,2-a]indole-8(9*H*)-carboxylate (3ak):

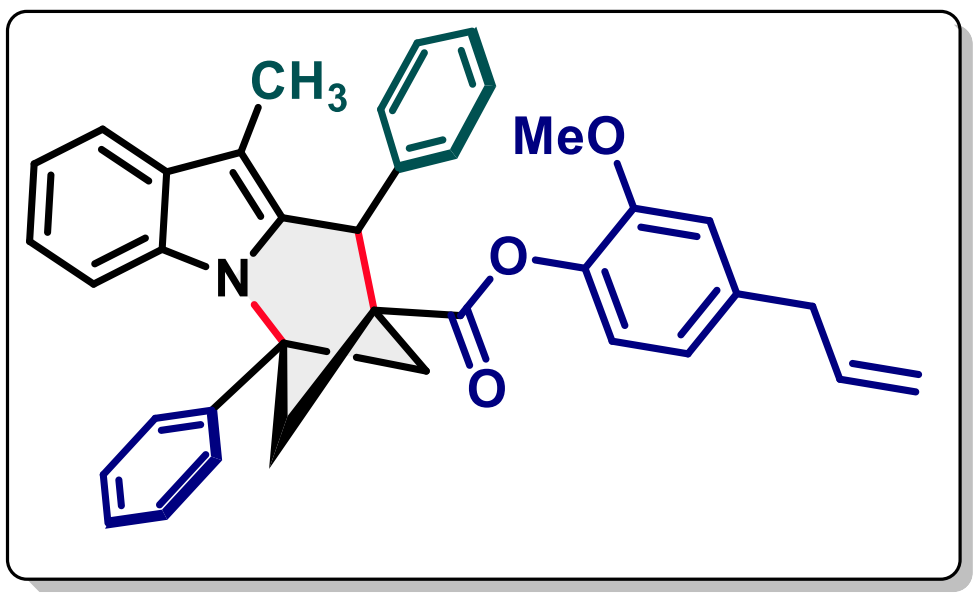

The reaction was performed according to the general procedure GP5 with 4-allyl-2-methoxyphenyl 3-phenylbicyclo[1.1.0]butane-1-carboxylate **1q** (96.0 mg, 0.3 mmol, 1.5 equiv.) and (3-methyl-1*H*-indol-2-yl)(phenyl)methanol **2a** (47.4 mg, 0.2 mmol, 1.00 equiv.) at 60 ºC for 2 h. After purification by flash column chromatography (hexane/ethyl acetate = 9.7/0.3), the desired compound **3aj** was obtained as a White solid (78.7 mg, 73% yield).

Rf = 0.45 (hexane/ethyl acetate = 9/1).

M.P. = 146 – 148 ºC

^{1}H NMR ((400 MHz, $CDCl_3$) δ (ppm)): δ 7.65 – 7.59 (m, 1H), 7.59 – 7.49 (m, 3H), 7.46 (ddt, *J* = 9.9, 7.9, 1.2 Hz, 2H), 7.42 – 7.37 (m, 2H), 7.37 – 7.28 (m, 3H), 6.93 (ddd, *J* = 7.9, 7.0, 0.9 Hz, 1H), 6.79 – 6.64 (m, 4H), 5.95 (ddt, *J* = 17.0, 10.2, 6.7 Hz, 1H), 5.34 – 5.26 (m, 2H), 5.15 – 5.05 (m, 2H), 3.75 (s, 3H), 3.37 (dt, *J* = 6.8, 1.5 Hz, 2H), 3.16 – 2.99 (m, 3H), 2.90 (ddd, *J* = 9.7, 6.6, 1.2 Hz, 1H), 1.86 (d, *J* = 0.9 Hz, 3H).

^{13}C {^{1}H} NMR ((101 MHz, $CDCl_3$) δ (ppm)): δ 203.03, 140.22, 139.98, 135.40, 134.97, 133.48, 133.28, 132.42, 130.39, 129.78, 129.27, 129.08, 128.95, 128.90, 128.87, 128.70, 128.57, 128.06, 127.87, 127.53, 126.88, 124.51, 120.90, 118.30, 111.39, 108.81, 62.47, 53.55, 49.63, 48.87, 41.58, 35.58, 29.83, 8.80.

HRMS (ESI) Calcd. for $C_{37}H_{32}NO_3$ $[M+H]^+$, 540.2533, found: 540.2539.

FTIR (cm^{-1}) : 1028, 1082, 1187, 1329, 1451, 1506, 1604, 1749, 2924.

(3,5-dimethyl-1*H*-pyrazol-1-yl)(10-methyl-6,9-diphenyl-6,7-dihydro-6,8-methanopyrido[1,2-a]indol-8(9*H*)-yl)methanone (3al):

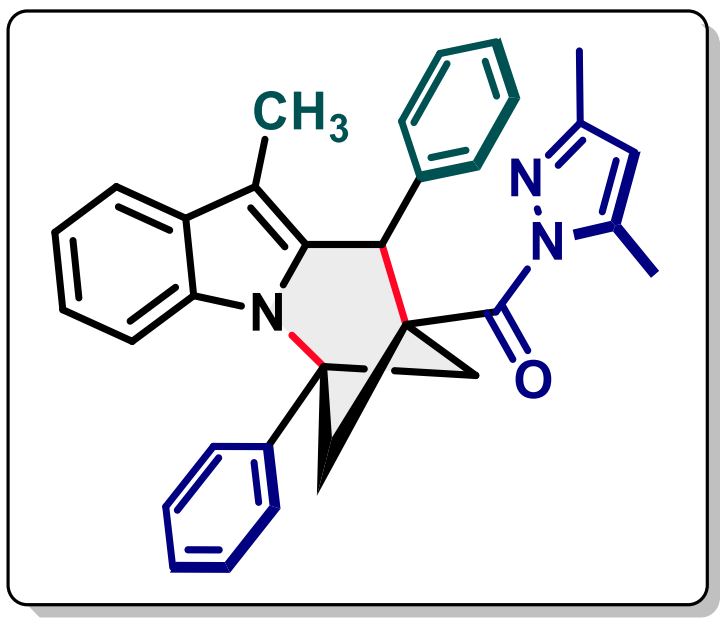

The reaction was performed according to the general procedure GP5 with (3,5-dimethyl-1*H*-pyrazol-1-yl)(3-phenylbicyclo[1.1.0]butan-1-yl)methanone **1u** (75.6 mg, 0.3 mmol, 1.5 equiv.) and (3-methyl-1*H*-indol-2-yl)(phenyl)methanol **2a** (47.4 mg, 0.2 mmol, 1.00 equiv.) at 60 ºC for 2 h. After purification by flash column chromatography (hexane/ethyl acetate = 9.8/0.2), the desired compound **3ak** was obtained as a White solid (69.7 mg, 74% yield).

Rf = 0.50 (hexane/ethyl acetate = 9/1).

M.P. = 214 – 216 ºC

^{1}H NMR ((400 MHz, $CDCl_3$) δ (ppm)): δ 7.66 – 7.60 (m, 1H), 7.58 – 7.48 (m, 4H), 7.46 (dt, *J* = 7.8, 1.1 Hz, 1H), 7.24 (dd, *J* = 5.0, 1.9 Hz, 3H), 6.98 – 6.85 (m, 3H), 6.70 (ddd, *J* = 8.4, 7.0, 1.3 Hz, 1H), 6.04 (d, *J* = 1.1 Hz, 1H), 5.74 (s, 1H), 5.34 (dt, *J* = 8.6, 0.8 Hz, 1H), 3.30 (dd, *J* = 10.3, 7.7 Hz, 1H), 3.16 (d, *J* = 10.3 Hz, 1H), 3.01 (dd, *J* = 10.6, 7.8 Hz, 1H), 2.90 (dd, *J* = 10.6, 1.0 Hz, 1H), 2.43 (d, *J* = 1.1 Hz, 3H), 2.32 (s, 3H), 1.85 (d, *J* = 0.9 Hz, 3H).

^{13}C {^{1}H} NMR ((101 MHz, $CDCl_3$) δ (ppm)): δ 173.42, 152.62, 144.33, 140.51, 140.13, 134.82, 133.52, 128.95, 128.93, 128.81, 128.46, 128.33, 128.17, 127.39, 120.64, 118.10, 118.08, 111.28, 110.84, 108.13, 62.34, 51.55, 48.96, 48.31, 40.33, 14.28, 8.85.

HRMS (ESI) Calcd. for $C_{32}H_{29}N_3O$ $[M+H]^+$, 472.2383, found: 472.2388.

FTIR (cm^{-1}) : 1343, 1376, 1450, 1715, 2926, 3029.

(10-Methyl-6,9-diphenyl-6,7-dihydro-6,8-methanopyrido[1,2-a]indol-8(9*H*)-yl)(naphthalen-2-yl)methanone (3am):

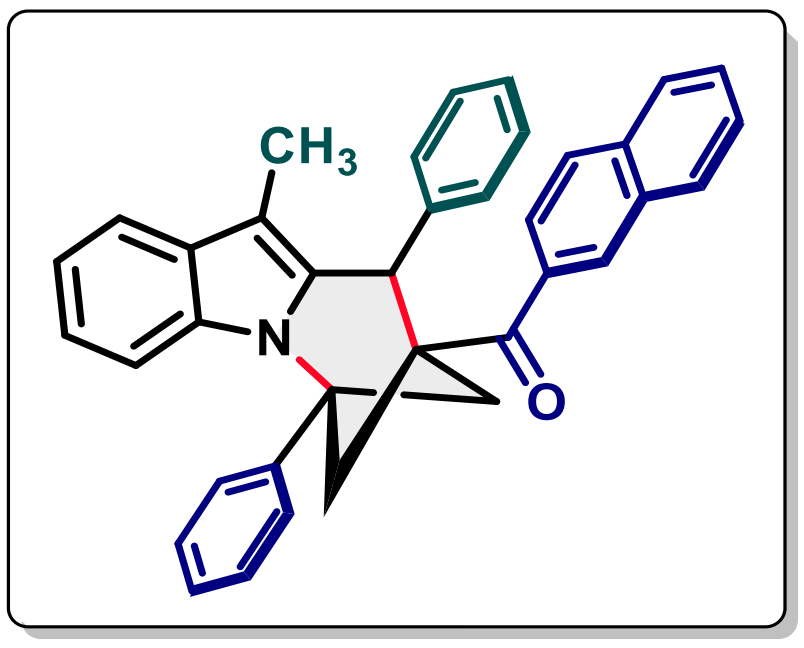

The reaction was performed according to the general procedure GP5 with naphthalen-2-yl(3-phenylbicyclo[1.1.0]butan-1-yl)methanone **1s** (85.2 mg, 0.3 mmol, 1.5 equiv.) and (3-methyl-1*H*-indol-2-yl)(phenyl)methanol **2a** (47.4 mg, 0.2 mmol, 1.00 equiv.) at 60 ºC for 2 h. After purification by flash column chromatography (hexane/ethyl acetate = 9.5/0.5), the desired compound **3al** was obtained as a colourless liquid (71.4 mg, 71% yield).

Rf = 0.38 (hexane/ethyl acetate = 9/1).

^{1}H NMR ((400 MHz, $CDCl_3$) δ (ppm)): δ 7.95 (d, *J* = 1.7 Hz, 1H), 7.91 – 7.75 (m, 4H), 7.69 (dd, *J* = 8.6, 1.8 Hz, 1H), 7.65 – 7.43 (m, 9H), 7.22 – 7.13 (m, 3H), 7.10 – 7.05 (m, 2H), 6.97 (ddd, *J* = 8.0, 7.0, 0.9 Hz, 1H), 6.73 (ddd, *J* = 8.5, 7.0, 1.3 Hz, 1H), 5.49 (s, 1H), 5.37 (d, *J* = 8.6 Hz, 1H), 3.24 – 3.12 (m, 3H), 2.99 – 2.91 (m, 1H), 1.90 (d, *J* = 0.9 Hz, 3H).

^{13}C {^{1}H} NMR ((101 MHz, $CDCl_3$) δ (ppm)): δ 203.03, 140.22, 139.98, 135.40, 134.97, 133.28, 132.42, 130.39, 129.78, 129.27, 129.08, 128.95, 128.89, 128.87, 128.70, 128.57, 128.55, 128.06, 128.04, 127.87, 127.53, 126.88, 120.90, 118.30, 111.39, 108.81, 62.47, 53.55, 49.63, 48.87, 41.58, 8.80.

HRMS (ESI) Calcd. for $C_{37}H_{29}NO$ $[M+H]^+$, 504.2322, found: 504.2313

FTIR (cm^{-1}) : 1067, 1105, 1245, 1330, 1450, 1666, 2983.

(3-methoxyphenyl)(10-methyl-6,9-diphenyl-6,7-dihydro-6,8-methanopyrido[1,2-a]indol-8(9*H*)-yl)methanone (3an):

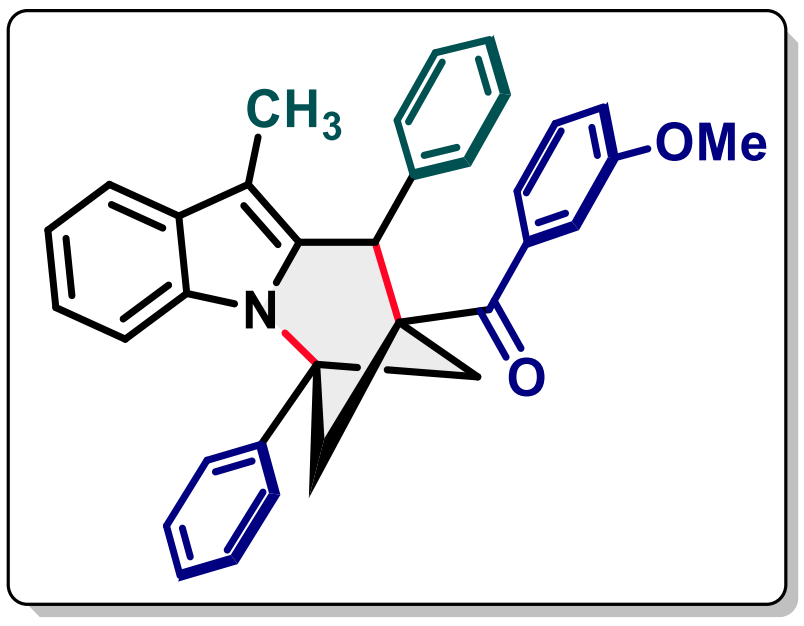

The reaction was performed according to the general procedure GP5 with (3-methoxyphenyl)(3-phenylbicyclo[1.1.0]butan-1-yl)methanone **1t** (79.2 mg, 0.3 mmol, 1.5 equiv.) and (3-methyl-1*H*-indol-2-yl)(phenyl)methanol **2a** (47.4 mg, 0.2 mmol, 1.00 equiv.) at 60 ºC for 2 h. After purification by flash column chromatography (hexane/ethyl acetate = 9.2/0.8), the desired compound **3am** was obtained as a colourless liquid (73.4 mg, 76% yield).

Rf = 0.29 (hexane/ethyl acetate = 9/1).

^{1}H NMR ((400 MHz, $CDCl_3$) δ (ppm)): δ 7.57 – 7.41 (m, 7H), 7.36 – 7.31 (m, 1H), 7.27 (d, *J* = 8.2 Hz, 1H), 7.19 (td, *J* = 4.9, 2.9 Hz, 2H), 7.15 – 7.10 (m, 1H), 7.04 (tt, *J* = 5.5, 3.0 Hz, 3H), 6.98 – 6.88 (m, 1H), 6.74 – 6.64 (m, 1H), 5.39 (s, 1H), 5.32 (t, *J* = 6.7 Hz, 1H), 3.75 (s, 3H), 3.01 (s, 3H), 2.77 (d, *J* = 9.0 Hz, 1H), 1.85 (s, 3H).

^{13}C {^{1}H} NMR ((101 MHz, $CDCl_3$) δ (ppm)): δ 202.83, 159.78, 140.19, 139.91, 137.24, 134.90, 129.59, 129.24, 129.06, 128.93, 128.87, 128.81, 128.53, 128.02, 127.44, 121.19, 120.83, 119.57, 118.25, 118.23, 112.91, 111.33, 108.74, 62.38, 55.51, 53.37, 49.41, 48.75, 41.42, 8.73.

HRMS (ESI) Calcd. for $C_{32}H_{29}N_3O$ $[M+H]^+$, 472.2383, found: 472.2388.

FTIR (cm^{-1}) : 1108, 1265, 1457, 1672, 2933.

6. Scale up synthesis of Methyl 10-methyl-6,9-diphenyl-6,7-dihydro-6,8-methanopyrido[1,2-a]indole-8(9*H*)-carboxylate(3a):

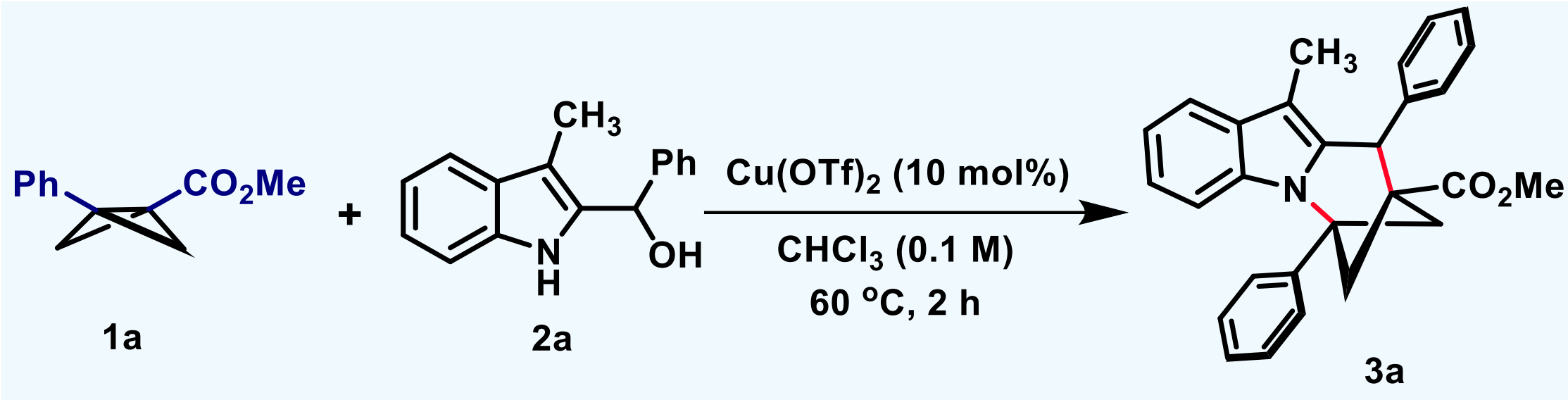

An oven-dried 250 mL Schlenk flask equipped with a stir bar was cooled under vacuum. (3-methyl-1*H*-indol-2-yl)(phenyl)methanol **2a** (3.2 mmol, 1.0 equiv.), and $Cu(OTf)_2$ (0.32 mmol, 0.10 equiv.) were loaded in the Schlenk flask. Methyl 3-phenylbicyclo[1.1.0]butane-1-carboxylate (BCB) **1a** (4.8 mmol, 1.5 equiv.) and $CHCl_3$ (0.1 M) was added at 60 ºC. The reaction was stirred for the 2 h. reaction mixture was diluted with 30 mL CH_2Cl_2. The crude residue was purified by column chromatography (hexane/ethyl acetate = 9.8/0.2), the desired compound **3a** was obtained as a White solid (1.01 g, 78% yield). on silica gel.

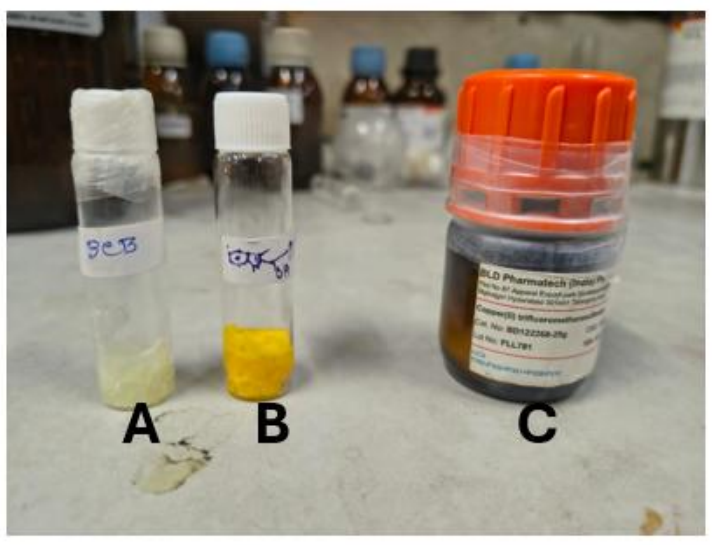

A :- Bicyclobutane,
B :- 2-indolylmethanol
C :- $Cu(OTf)_2$

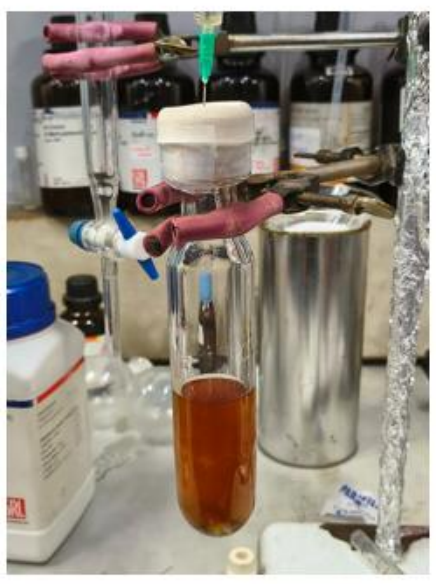

After Addition of all the reagent and solvent under inert condition

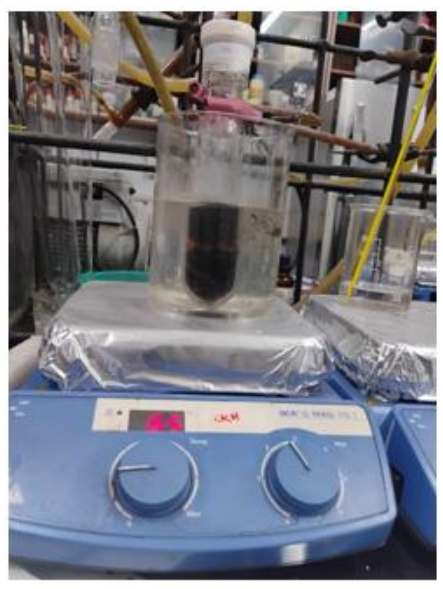

After Reaction

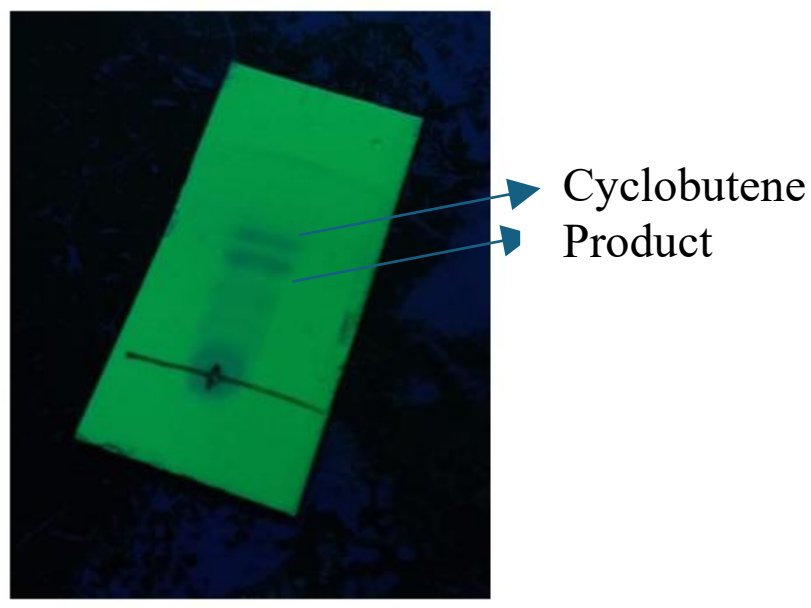

TLC after Reaction

7. Synthetic Applications

7.1. Synthesis of (10-methyl-6,9-diphenyl-6,7-dihydro-6,8-methanopyrido[1,2-a]indol-8(9*H*)-yl)diphenylmethanol (4):

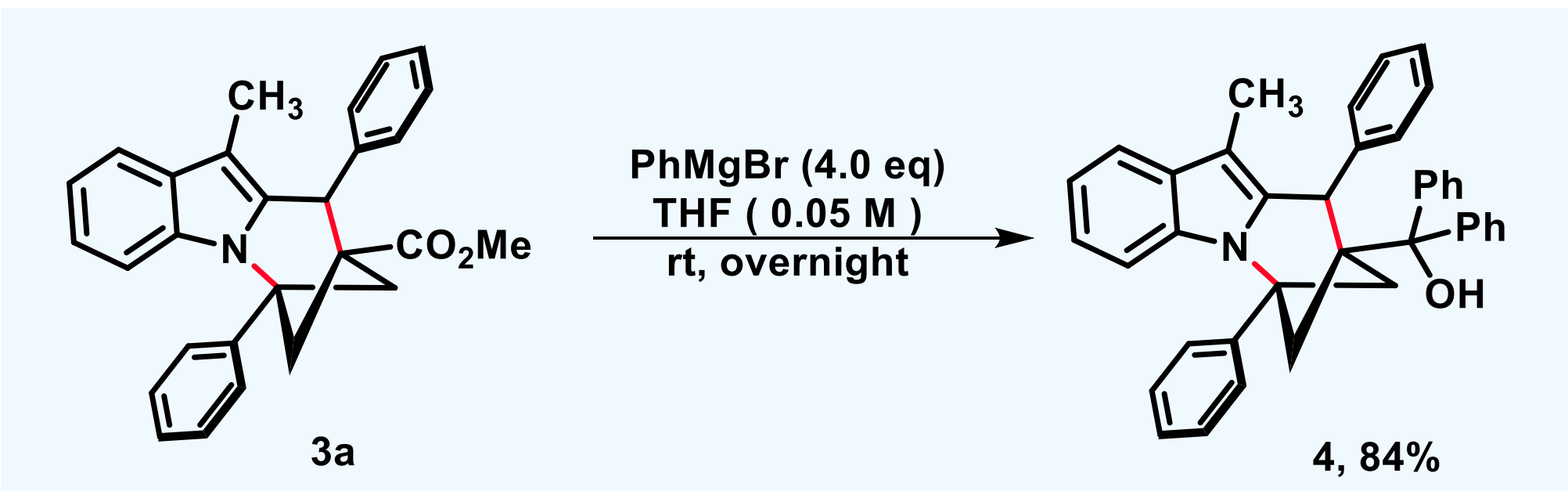

3a (20.3 mg, 0.05 mmol, 1.0 equiv.) was dissolved in anhydrous tetrahydrofuran (2.0 mL) under nitrogen atmosphere. Phenyl magnesium bromide (0.067 mL, 3.0 M solution in THF, 0.200 mmol, 4.0

equiv.) was added dropwise at room temperature. The reaction was allowed to stir overnight at room temperature. Ammonium chloride solution (2 mL) was added and the phases were separated. The aqueous phase was extracted with ethyl acetate (3 x 5 ml), and the combined organic phases were dried over sodium sulfate, filtered, and concentrated under reduced pressure. Purification by column chromatography on silica gel (hexane/ethyl acetate, = 9.2/0.8) gave **4** as a colorless solid (22.3 mg, 84%).

Rf = 0.29 (hexane/ethyl acetate = 9/1).

M.P. = 244 – 246 ºC

^{1}H NMR ((400 MHz, $CDCl_3$) δ (ppm)): δ 7.54 (d, *J* = 7.6 Hz, 2H), 7.50 – 7.30 (m, 11H), 7.29 – 7.22 (m, 3H), 7.17 (ddd, *J* = 7.9, 7.0, 1.2 Hz, 3H), 7.12 – 7.03 (m, 1H), 6.85 (ddd, *J* = 7.9, 7.0, 0.9 Hz, 1H), 6.61 (ddd, *J* = 8.4, 7.0, 1.3 Hz, 1H), 6.19 (s, 1H), 5.31 (s, 1H), 5.08 (dt, *J* = 8.6, 0.9 Hz, 1H), 3.13 (dd, *J* = 10.6, 7.5 Hz, 1H), 2.86 – 2.72 (m, 2H), 2.66 (dd, *J* = 10.6, 1.2 Hz, 1H), 1.73 (s, 1H), 1.57 (d, *J* = 0.9 Hz, 3H).

^{13}C {^{1}H} NMR ((101 MHz, $CDCl_3$) δ (ppm)): δ 146.07, 142.56, 141.61, 140.85, 136.08, 134.19, 129.39, 128.74, 128.69, 128.26, 127.90, 127.75, 127.46, 127.43, 127.36, 126.90, 120.42, 117.98, 117.85, 111.23, 107.86, 81.87, 62.40, 50.55, 48.51, 47.86, 38.41, 8.24.

HRMS (ESI) Calcd. for $C_{39}H_{33}NO$ $[M+H]^+$, 532.2635, found: 532.2639.

FTIR (cm^{-1}) : 1027, 1332, 1450, 2926, 3058, 3544.

7.2. Synthesis of (10-methyl-6,9-diphenyl-6,7-dihydro-6,8-methanopyrido[1,2-a]indol-8(9*H*)-yl)methanol (5):

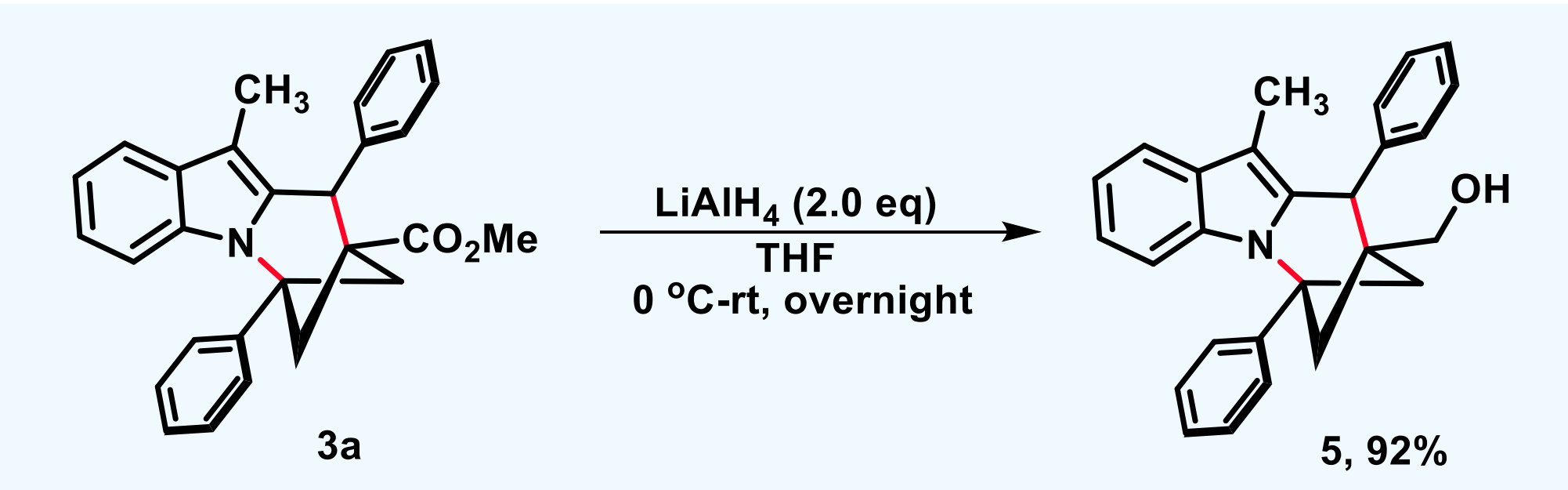

3a (20.4 mg, 0.05 mmol, 1.0 equiv.) was dissolved in anhydrous tetrahydrofuran (0.25 mL) under nitrogen atmosphere. Lithium aluminum hydride (2.5 mg, 0.100 mmol, 2.0 equiv.) was added at 0 ºC. The reaction was allowed to stir overnight at room temperature. Ammonium chloride solution (2 mL) was added and the phases were separated. The aqueous phase was extracted with ethyl acetate (3 x 5 ml), and the combined organic phases were dried over sodium sulfate, filtered, and concentrated under reduced pressure. Purification by column chromatography on silica gel (hexane/ethyl acetate, = 8.5/1.5) gave **5** as a yellow solid (17.4 mg, 92%).

Rf = 0.73 (hexane/ethyl acetate = 7/3).

M.P. = 136 – 138 °C

^{1}H NMR ((400 MHz, $CDCl_3$) δ (ppm)): δ 7.58 – 7.46 (m, 5H), 7.42 (dt, *J* = 7.9, 1.1 Hz, 1H), 7.38 – 7.25 (m, 6H), 6.90 (ddd, *J* = 7.9, 7.0, 0.9 Hz, 1H), 6.66 (ddd, *J* = 8.4, 7.0, 1.2 Hz, 1H), 5.29 (dd, *J* = 8.6, 1.0 Hz, 1H), 4.83 (s, 1H), 3.59 (d, *J* = 9.6 Hz, 1H), 3.48 (d, *J* = 11.1 Hz, 1H), 2.80 – 2.67 (m, 2H), 2.51 (dd, *J* = 9.5, 8.1 Hz, 1H), 2.30 (dd, *J* = 10.2, 1.1 Hz, 1H), 1.81 (d, *J* = 0.9 Hz, 3H).

^{13}C {^{1}H} NMR ((101 MHz, $CDCl_3$) δ (ppm)): δ 141.12, 140.84, 135.10, 134.61, 129.44, 129.41, 128.84, 128.81, 128.43, 128.20, 128.03, 126.99, 126.25, 120.45, 118.06, 117.93, 111.26, 108.27, 66.63, 63.06, 47.15, 45.51, 44.33, 38.67, 8.62.

HRMS (ESI) Calcd. for $C_{27}H_{25}NO$ $[M+H]^+$, 380.2009, found: 380.2008.

FTIR (cm^{-1}) : 1027, 1329, 1450, 1627, 3423.

7.3. Synthesis of 8-(bromomethyl)-10-methyl-6,9-diphenyl-6,7,8,9-tetrahydro-6,8-methanopyrido[1,2-a]indole (6):

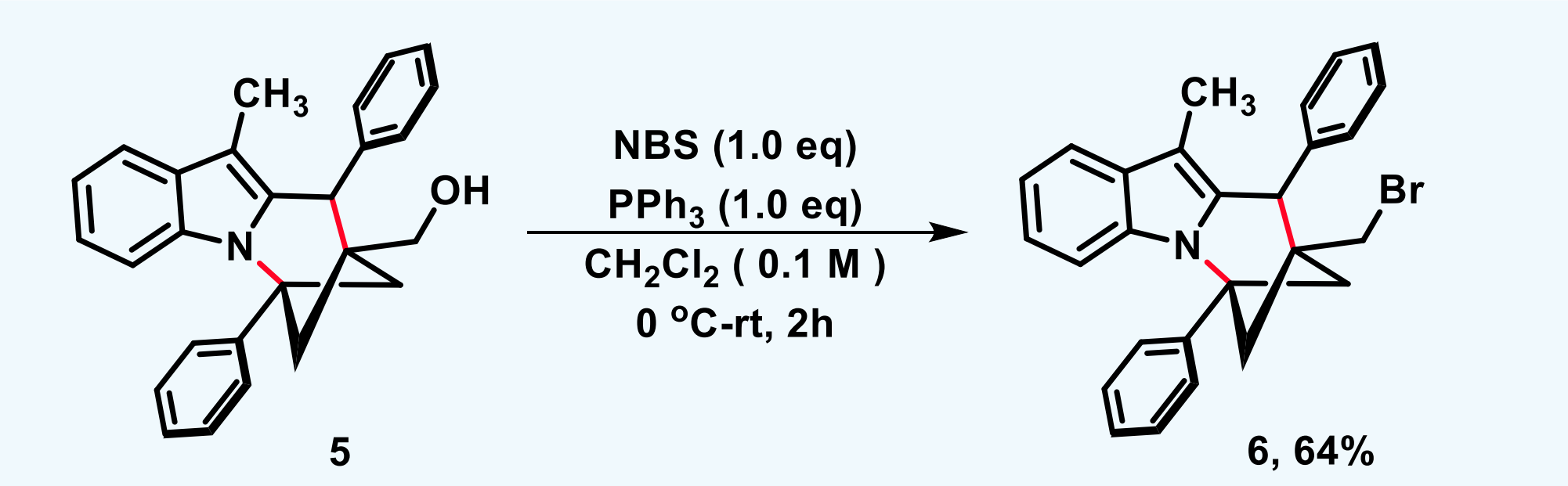

To a stirred solution of **5** (18.9 mg, 0.05 mmol) and PPh_3 (13 mg, 1.0 equiv) in 0.5 mL of CH_2Cl_2 was added NBS (9 mg, 1.0 equiv) slowly at 0 °C. The mixture was stirred for 2 hours at 25 °C. After the reaction was completed, the reaction mixture was quenched with water and extracted with CH_2Cl_2. The organic phase was dried over Na_2SO_4 and concentrated under reduced pressure and purified by flash column chromatography (hexane/ethyl acetate, = 9.9/0.1) of the crude reaction mixture afforded 8-bromomethyl-10-methyl-6,9-diphenyl-6,7,8,9-tetrahydro-6,8-methanopyrido[1,2-a]indole **6** as a white solid (14.1 mg, 64% yield).

Rf = 0.86 (hexane/ethyl acetate = 9/1).

M.P. = 180 – 182 °C

^{1}H NMR ((400 MHz, $CDCl_3$) δ (ppm)): δ 7.61 – 7.47 (m, 4H), 7.47 – 7.43 (m, 1H), 7.41 (dt, *J* = 7.9, 1.0 Hz, 1H), 7.39 – 7.27 (m, 6H), 6.90 (ddd, *J* = 7.9, 7.0, 0.9 Hz, 1H), 6.65 (ddd, *J* = 8.4, 7.0, 1.3 Hz, 1H), 5.31 – 5.24 (m, 1H), 4.93 (s, 1H), 3.50 – 3.31 (m, 2H), 2.91 (dd, *J* = 10.2, 7.8 Hz, 1H), 2.70 – 2.55 (m, 2H), 2.28 (dd, *J* = 10.2, 1.3 Hz, 1H), 1.81 (d, *J* = 0.8 Hz, 3H).

^{13}C {^1H} NMR ((101 MHz, $CDCl_3$) δ (ppm)): δ 140.50, 140.17, 134.61, 134.52, 129.31, 129.01, 128.93, 128.87, 128.52, 128.15, 127.98, 127.28, 120.59, 118.14, 118.06, 111.19, 108.58, 61.57, 47.51, 46.71, 43.52, 42.08, 40.82, 8.57.

HRMS (ESI) Calcd. for $C_{27}H_{24}BrN$ $[M+H]^+$, 442.1165, found: 442.1163.

FTIR (cm^{-1}) : 1024, 1073, 1233, 1327, 1448, 2925.

7.4. Synthesis of methyl 10-methyl-2,6,9-triphenyl-6,7-dihydro-6,8-methanopyrido[1,2-a]indole-8(9*H*)-carboxylate (7):

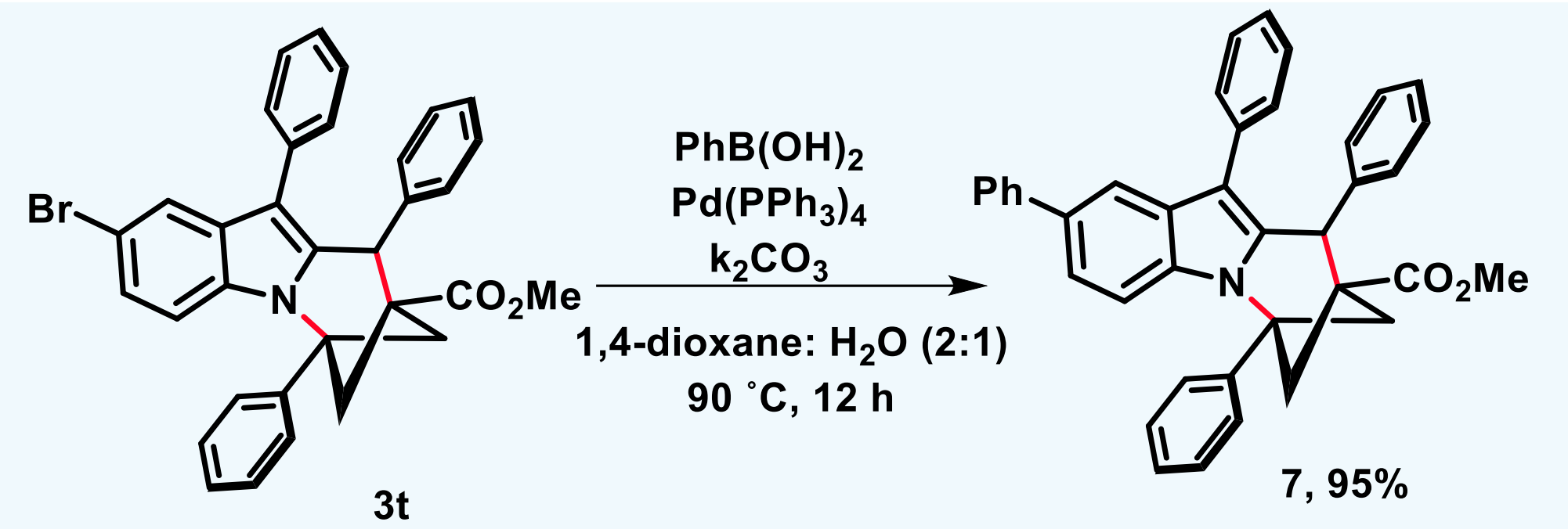

3t (24.3 mg, 0.05 mmol, 1.0 equiv) was added substituted phenylboronic acid (12.0 mg, 2.0 equiv), $Pd(PPh_3)_4$ (3.0 mg, 0.05 equiv) and K_2CO_3 (27.0 mg, 4.0 equiv). The mixture was placed under vacuum then backfilled with nitrogen for three cycles. To this mixture was added degassed 1,4-dioxane (0.6 ml) and water (0.3 ml). The reaction was refluxed for 12 hours at 90 °C. The crude mixture was quenched with saturated ammonium chloride solution, diluted with EtOAc, and washed with H_2O and brine. The organic layer was collected, and the aqueous layer was extracted three times with ethyl acetate. The organic layers were combined, dried with Na_2SO_4, and concentrated in vacuo to yield crude products. Purification through flash column chromatography (hexane/ethyl acetate, = 9.1/0.9) afforded the desired product methyl-10-methyl-2,6,9-triphenyl-6,7-dihydro-6,8-methanopyrido[1,2-a]indole-8(9*H*)-carboxylate **7** as white solid (22.9 mg, 95% yield).

Rf = 0.26 (hexane/ethyl acetate = 9/1).

M.P. = 181 – 183 ºC

^1H NMR ((400 MHz, $CDCl_3$) δ (ppm)): δ 7.80 (dd, *J* = 1.9, 0.6 Hz, 1H), 7.71 – 7.65 (m, 1H), 7.63 – 7.49 (m, 6H), 7.41 – 7.31 (m, 2H), 7.27 – 7.25 (m, 3H), 7.22 – 7.16 (m, 2H), 7.15 – 7.08 (m, 6H), 7.10 – 6.98 (m, 1H), 5.48 – 5.38 (m, 2H), 3.64 (s, 3H), 3.06 (dd, *J* = 10.6, 7.4 Hz, 1H), 3.01 – 2.88 (m, 3H).

^{13}C {^1H} NMR ((101 MHz, $CDCl_3$) δ (ppm)): δ 173.62, 142.39, 140.15, 139.96, 134.88, 134.82, 134.41, 132.90, 129.50, 129.39, 129.14, 129.09, 128.88, 128.63, 128.21, 128.12, 128.07, 127.38, 127.18, 126.34, 125.99, 121.21, 117.73, 116.36, 115.45, 112.00, 63.03, 51.98, 48.38, 48.11, 47.27, 39.15.a

HRMS (ESI) Calcd. for $C_{39}H_{31}NO_2$ $[M+H]^+$, 546.2428, found: 546.2429.

FTIR (cm^{-1}) : 1104, 1213, 1331, 1451, 1733, 2924, 3048.

7.5. Synthesis of 10-methyl-6,9-diphenyl-6,7-dihydro-6,8-methanopyrido[1,2-a]indole-8(9*H*)-carbaldehyde (8):

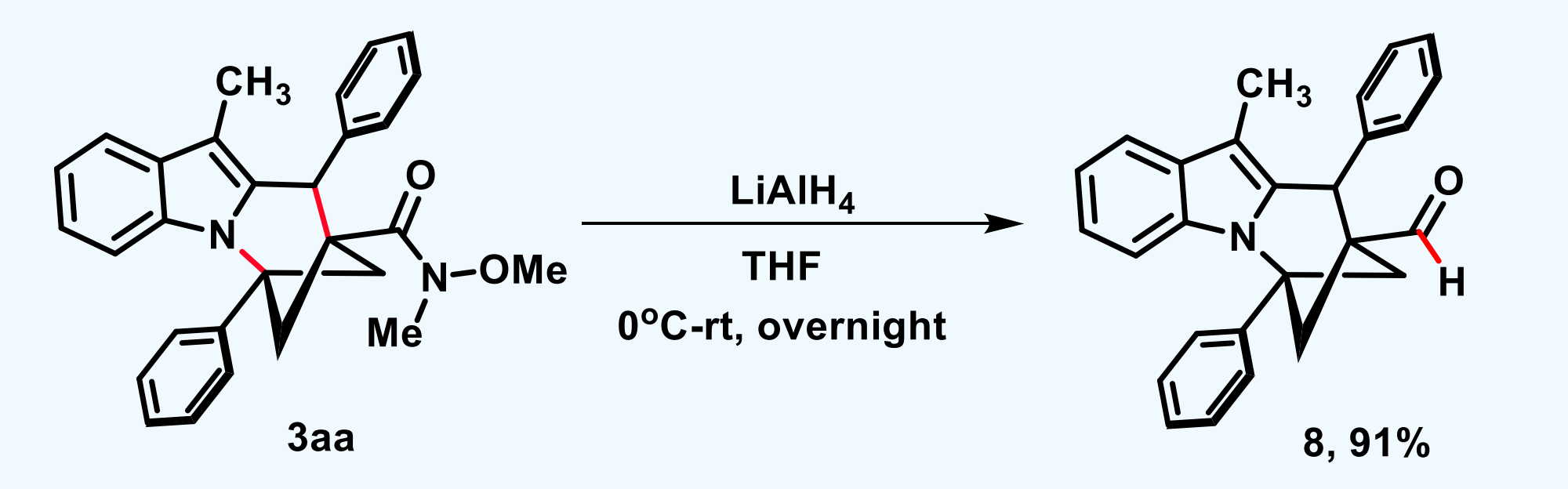

$LiAlH_4$ (7.6 mg, 0.2 mmol) was added to the solution of **3aa** (21.8 mg, 0.050 mmol, 1.0 equiv.) in dry THF (1.0 mL) at 0 °C. The reaction was allowed to stir at 0 °C for 24 h. The reaction was quenched by addition of 2 mL water and the aqueous layer was extracted with ethyl acetate (3 x 5 mL). Purification by column chromatography on silica gel (hexane/ethyl acetate, = 9.2/0.8) gave **8** as a colorless gummy liquid (17.2 mg, 91%).

Rf = 0.31 (pentane/ethyl acetate = 9/1).

M.P. = 62 – 64 °C

^{1}H NMR ((400 MHz, $CDCl_3$) δ (ppm)): δ 9.77 (s, 1H), 7.62 – 7.47 (m, 4H), 7.45 – 7.40 (m, 2H), 7.35 – 7.25 (m, 5H), 6.93 (ddd, *J* = 7.9, 7.0, 0.9 Hz, 1H), 6.69 (ddd, *J* = 8.5, 7.0, 1.3 Hz, 1H), 5.29 (dt, *J* = 8.6, 0.9 Hz, 1H), 5.22 (s, 1H), 2.91 – 2.75 (m, 3H), 2.70 – 2.61 (m, 1H), 1.86 (d, *J* = 0.9 Hz, 3H).

^{13}C {^{1}H} NMR ((101 MHz, $CDCl_3$) δ (ppm)): δ 201.48, 140.07, 139.82, 134.94, 133.18, 129.27, 129.22, 129.12, 129.01, 128.96, 128.74, 127.93, 127.86, 127.52, 121.00, 118.34, 118.30, 111.35, 109.17, 62.74, 52.43, 46.14, 45.10, 37.85, 8.65.

HRMS (ESI) Calcd. for $C_{27}H_{23}NO$ $[M+H]^+$, 378.1852, found: 378.1848.

FTIR (cm^{-1}) : 1329, 1450, 1716, 2853, 2924.

7.6. Synthesis of 10-methyl-6,9-diphenyl-6,7-dihydro-6,8-methanopyrido[1,2-a]indole-8(9*H*)-carbaldehyde (9):

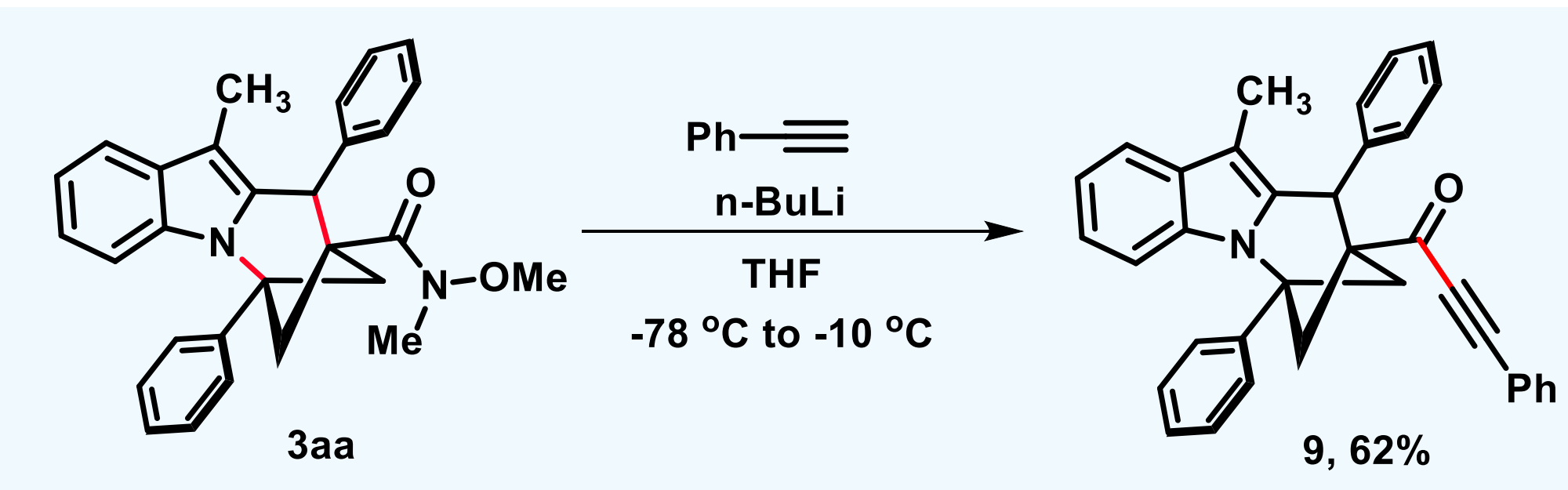

nBuLi (1.6 M solution in hexane, 0.08 mL, 0.125 mmol) was added to the solution of phenylacetelyne (13.5 μL, 0.125 mmol) in dry THF (0.6 mL) at -78 °C. The reaction was allowed to stir at the same temperature for 30 mins. **3aa** (21.8 mg, 0.050 mmol, 1.0 equiv.) in 0.2 mL THF was added dropwise at -78 °C. After stirring for 10 minutes at -78 °C, the reaction was allowed to stir for 1 h at -10 °C. The reaction was then quenched by addition of 2 mL aq NH_4Cl and the aqueous layer was extracted with ethyl acetate (3 x 5 mL). Purification by column chromatography on silica gel (hexane/ethyl acetate, = 9.6/0.4) gave **9** as a colorless solid (14.8 mg, 62%).

Rf = 0.48 (pentane/ethyl acetate = 9/1).

M.P. = 172– 174 °C

^{1}H NMR ((400 MHz, $CDCl_3$) δ (ppm)): δ 7.65 – 7.59 (m, 1H), 7.55 (ddt, *J* = 7.1, 3.1, 1.3 Hz, 4H), 7.52 – 7.34 (m, 7H), 7.29 (d, *J* = 4.3 Hz, 4H), 6.93 (t, *J* = 7.5 Hz, 1H), 6.69 (td, *J* = 7.6, 1.3 Hz, 1H), 5.36 (s, 1H), 5.29 (d, *J* = 8.6 Hz, 1H), 3.18 – 2.65 (m, 4H), 1.87 (s, 3H).

^{13}C {^{1}H} NMR ((101 MHz, $CDCl_3$) δ (ppm)): δ 188.56, 140.06, 139.59, 134.89, 133.34, 133.32, 131.15, 129.24, 129.16, 129.04, 129.00, 128.91, 128.79, 128.55, 128.01, 127.48, 120.90, 119.73, 118.28, 118.26, 111.32, 108.97, 94.85, 85.97, 61.90, 54.85, 47.59, 47.20, 38.79, 8.67.

HRMS (ESI) Calcd. for $C_{35}H_{27}NO$ $[M+H]^+$, 478.2165, found: 478.2174.

FTIR (cm^{-1}) : 1044, 1166, 1329, 1449, 1658, 2187, 2924.

8. Mechanistic experiments:

8.1. Intermediate detemination

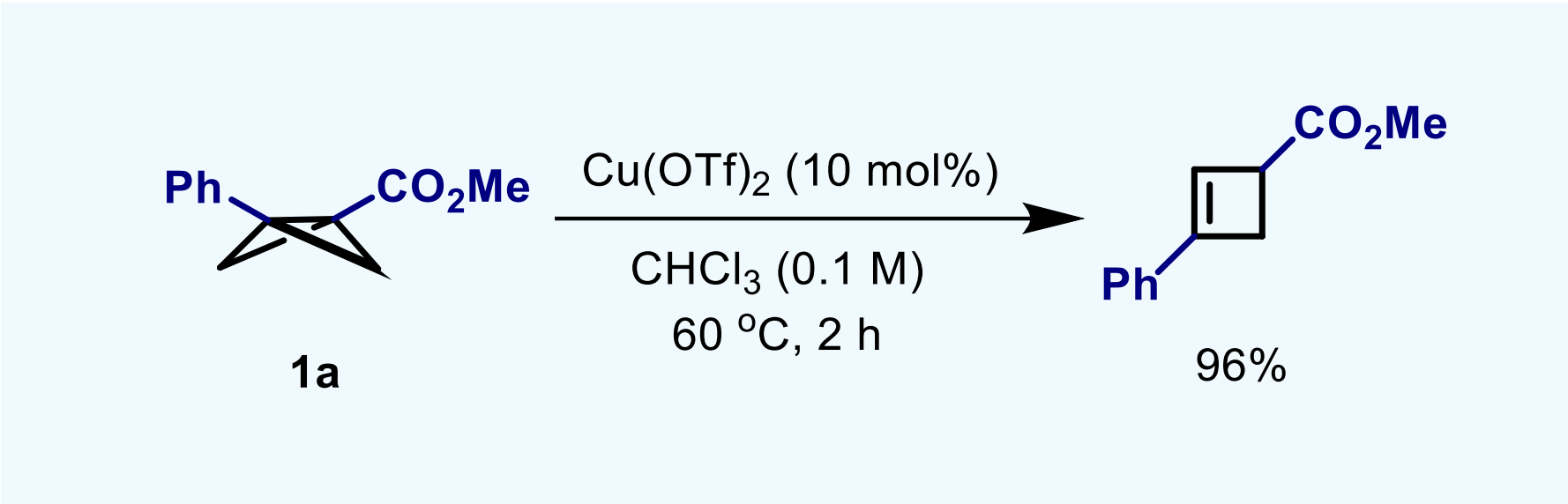

An oven-dried 10 mL Schlenk flask equipped with a stir bar was cooled under vacuum. BCB **1a** (0.15 mmol, 1.5 equiv.), and Catalyst (0.01 mmol, 0.10 equiv.) were loaded in the Schlenk flask. Solvent was added at the mentioned temperature. The reaction was stirred for the 2 h. then the solvent was evaporated, and the crude mixture was passed through a pad of silica gel and eluted with EtOAc (3x10 mL). The reaction mixture was concentrated under reduced pressure and then the yield of cyclobutene was determined by the ^{1}H NMR analysis of the crude reaction products using trichloroethylene (TCE) as the internal standard. And the crude residue was purified by column chromatography on silica gel (hexane/ethyl acetate = 98/2) to give cyclobutene as pale-yellow oil. The analytical data is matching with the reported value.

^{1}H-NMR of 1a

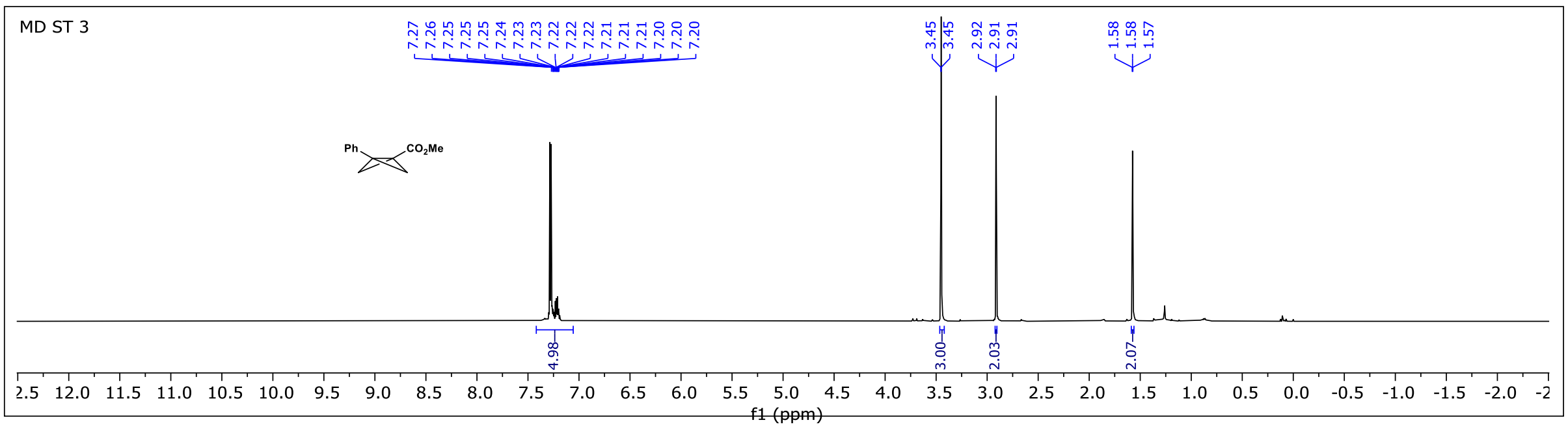

^{1}H-NMR of Cyclobutene

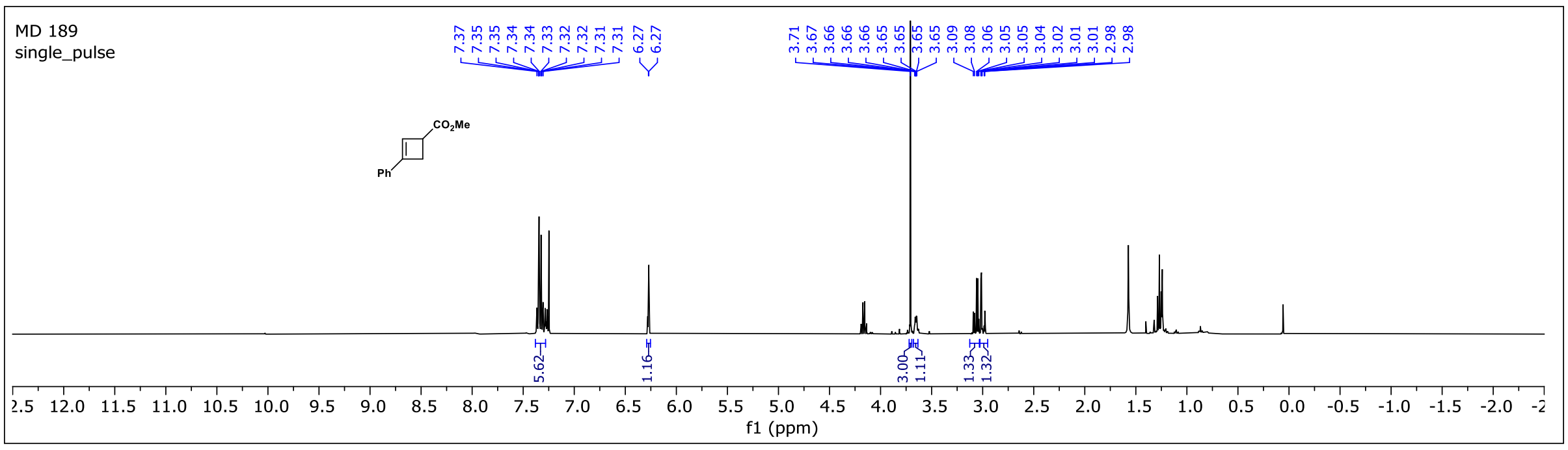

^{1}H-NMR of the Crude Reaction Mixture

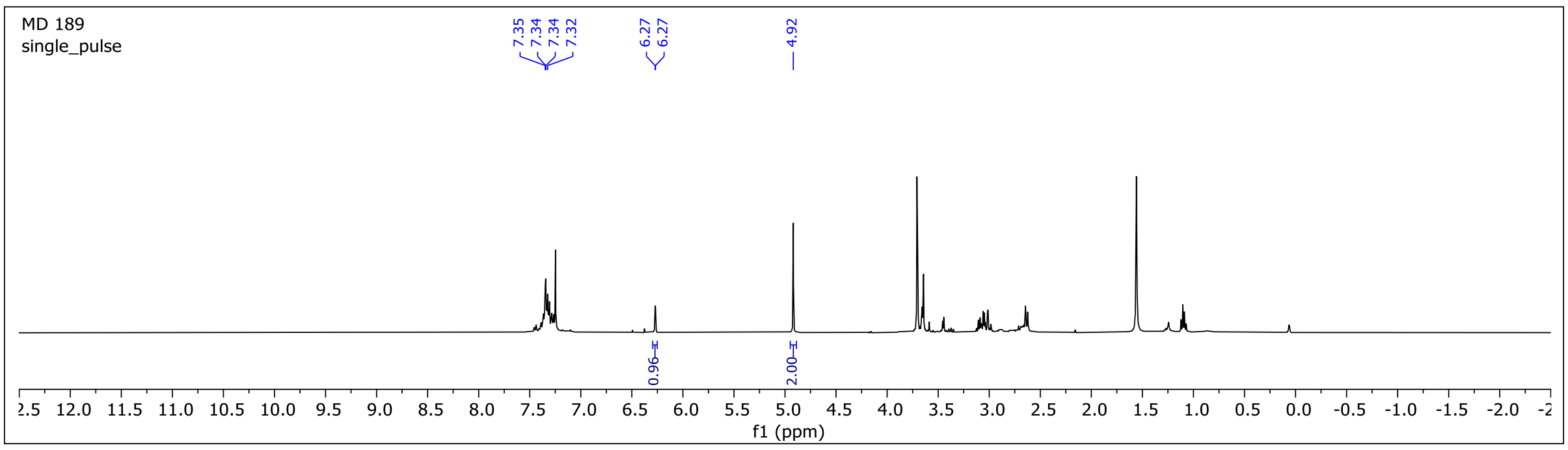

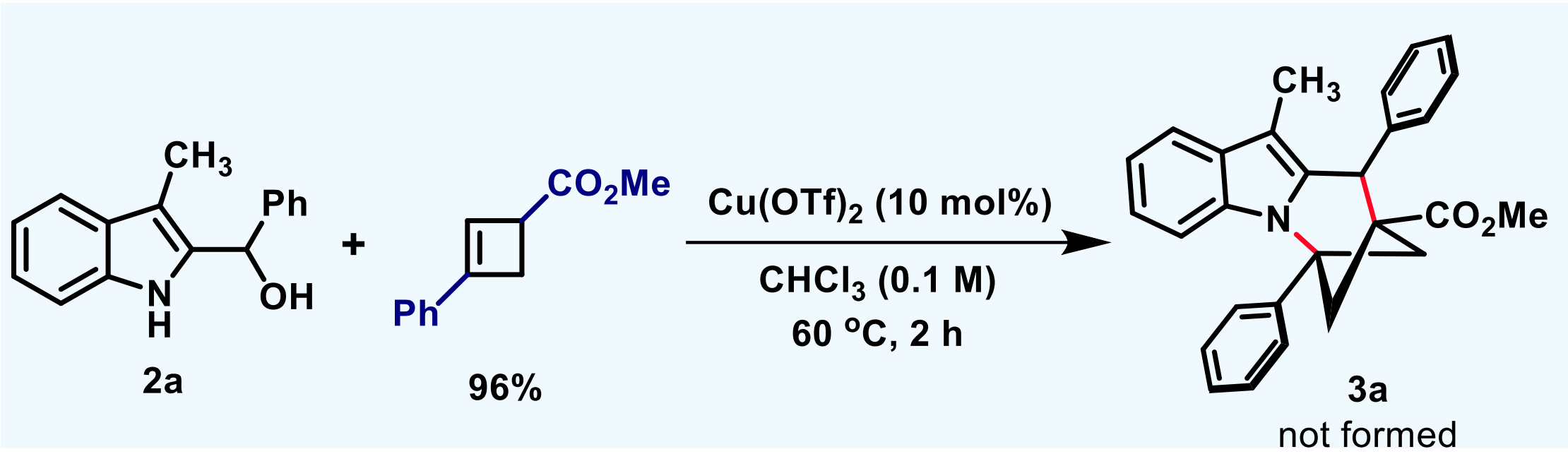

An oven-dried 10 mL Schlenk flask equipped with a stir bar was cooled under vacuum. Cyclobutene (14.1 mg, 0.075 mmol, 1.0 equiv.) and indolyl methanol derivative **2a** (11.8 mg, 0.05 mmol, 1.5 equiv.) were loaded in the Schlenk flask. The suspension was dissolved with $CHCl_3$ (1.0 mL, 0.05M) at the mentioned temperature for 2 h. **3a** product was not formed.

In conclusion, cyclobutene is not a viable intermediate; instead, it inhibits the reaction by forming as a side product. Therefore, it is essential to add BCB only after combining indolylmethanols, Cu(OTf)₂, and the solvent. Direct contact of BCB with Cu(OTf)₂ also leads to cyclobutene formation.

8.2. 3-position unprotected indolyl alcohol

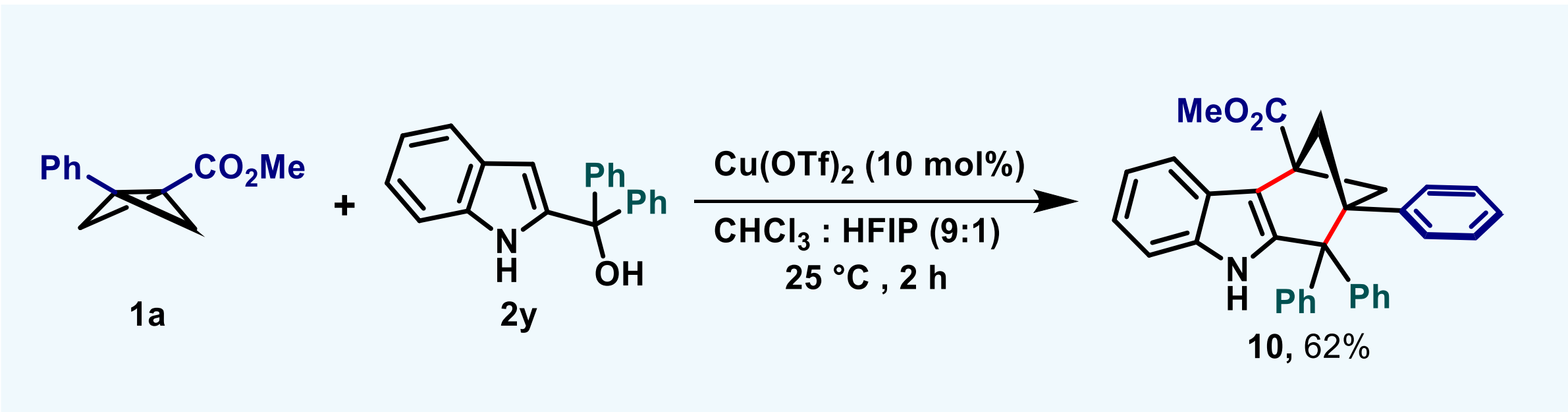

According to the general procedure GP5 an oven-dried 10 mL Schlenk flask equipped with a stir bar was cooled under vacuum. (1H-indol-2-yl)diphenylmethanol **2y** (59.8 mg, 0.2 mmol, 1.0 equiv.), and $Cu(OTf)_2$ (7.2 mg, 0.02 mmol, 0.10 equiv.) were loaded in the Schlenk flask. BCB **1a** (56.4 mg, 0.3 mmol, 1.5 equiv.), Solvent $CHCl_3$ (1.8 mL) and HFIP (0.2 ml) was added at the mentioned temperature. The reaction was stirred for the 2h. reaction mixture was diluted with 10 mL CH_2Cl_2. The crude residue

was purified by column chromatography on silica gel (hexane/ethyl acetate = 9.5:0.5) gave **10** as a colorless semi-solid (58.2 mg, 62%).

Rf = 0.43 (hexane/ethyl acetate = 9/1).

M.P. = 163 – 165 °C

^{1}H NMR ((400 MHz, $CDCl_3$) δ (ppm)): 7.51 – 7.45 (m, 3H), 7.42 – 7.37 (m, 1H), 7.35 – 7.32 (m, 2H), 7.27 (d, J = 1.8 Hz, 1H), 7.26 – 7.21 (m, 5H), 7.04 (ddd, J = 8.2, 6.4, 1.9 Hz, 1H), 6.95 – 6.87 (m, 2H), 4.95 (s, 1H), 3.61 (s, 3H), 2.85 – 2.68 (m, 4H).

^{13}C {^{1}H} NMR ((101 MHz, $CDCl_3$) δ (ppm)): 174.8, 146.8, 140.9, 140.8, 134.9, 129.1, 128.8, 128.2, 127.5, 127.1, 127.0, 126.4, 120.6, 119.9, 118.7, 111.1, 106.4, 51.5, 50.6, 47.4, 46.9, 42.6, 38.0.

HRMS (ESI) Calcd. for $C_{33}H_{27}NO_2$ $[M+H]^+$, 470.2115, found: 470.2119.

FTIR (cm^{-1}) : 1114, 1210, 1328, 1448, 1724, 2924.

In conclusion, protection at the C3 position of 2-indolylmethanols is essential; otherwise, undesired products form due to the higher reactivity of the indole C3 position compared to the N1 position.

8.3. Reaction with unsubstituted BCBs

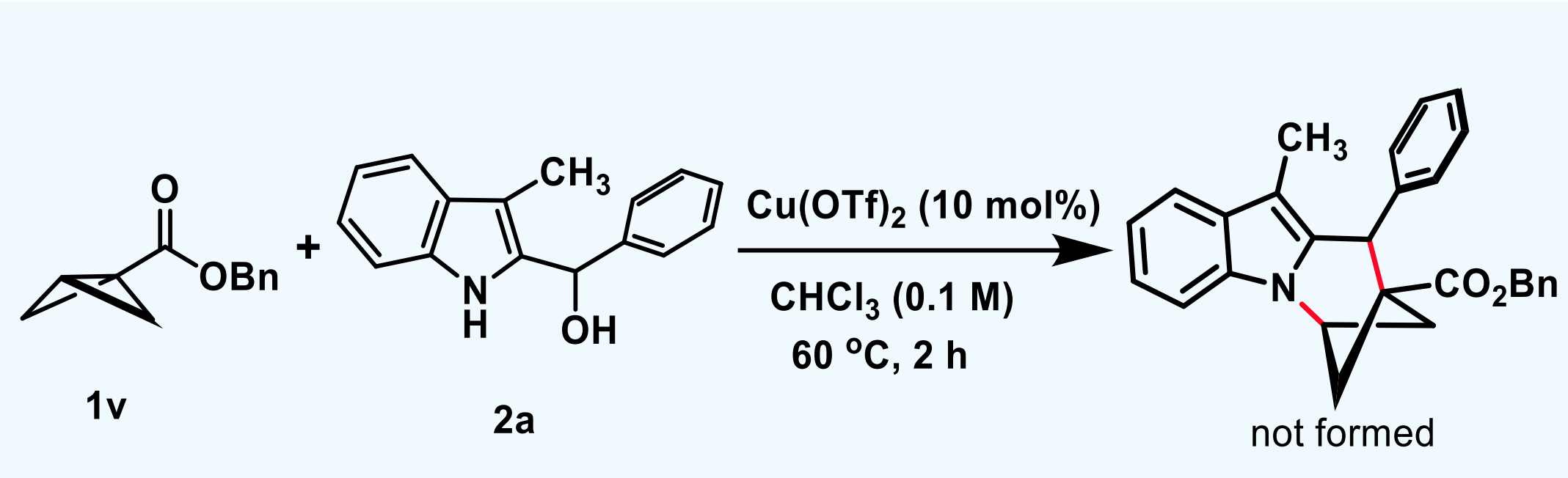

An oven-dried 10 mL Schlenk flask equipped with a stir bar was cooled under vacuum. (3-methyl-1*H*-indol-2-yl)(phenyl)methanol **2a** (47.4 mg, 0.2 mmol, 1.0 equiv.), and $Cu(OTf)_2$ (7.2 mg, 0.02 mmol, 0.10 equiv.) were loaded in the Schlenk flask. benzyl bicyclo[1.1.0]butane-1-carboxylate (BCB) **1v** (56.4 mg, 0.3 mmol, 1.5 equiv.) and $CHCl_3$ (0.1 M) was added at 60 ºC. The reaction was stirred for the 2 h. reaction mixture was diluted with 30 mL CH_2Cl_2. The crude residue was analyzed using ^{1}H NMR spectroscopy.

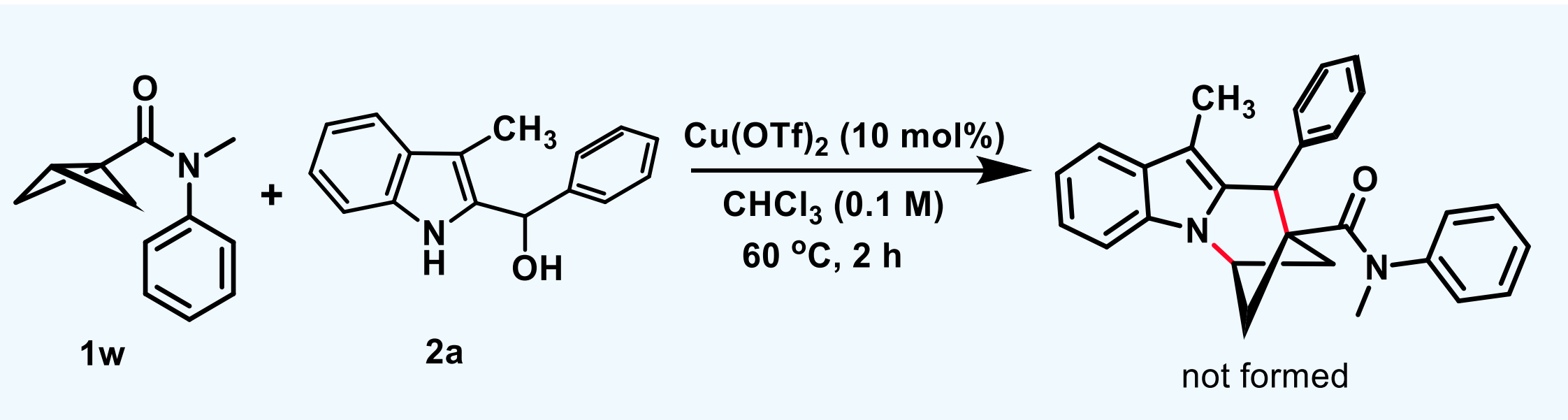

An oven-dried 10 mL Schlenk flask equipped with a stir bar was cooled under vacuum. (3-methyl-1H-indol-2-yl)(phenyl)methanol **2a** (47.4 mg, 0.2 mmol, 1.0 equiv.), and $Cu(OTf)_2$ (7.2 mg, 0.02 mmol, 0.10 equiv.) were loaded in the Schlenk flask. N-methyl-N-phenylbicyclo[1.1.0]butane-1-carboxamide (BCB) **1w** (56.1 mg, 0.3 mmol, 1.5 equiv.) and $CHCl_3$ (0.1 M) was added at 60 °C. The reaction was stirred for the 2 h. reaction mixture was diluted with 30 mL CH_2Cl_2. The crude residue was analyzed using ^1H NMR spectroscopy.

These experiments highlight the importance of the aryl moiety for the formation of the desired product. The lack of product formation with monosubstituted BCBs is likely due to the instability of the carbocation generated upon Lewis acid activation, which is required for ester enolate formation. In contrast, when an aryl group is present at the 3-position of the BCB, the resulting carbocation is stabilized through resonance with the aryl moiety, thereby facilitating the formation of the aza-bicyclo[3.1.1]heptane product in the case of disubstituted keto/ester BCBs.

8.4. Reaction with unsubstituted 2-indolylalcohol

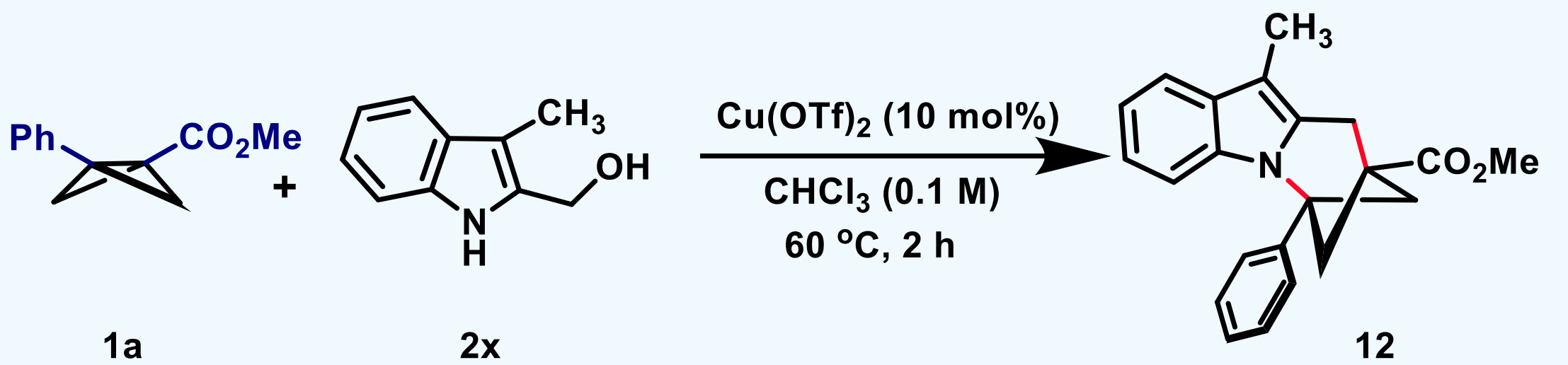

1a 2x 12 not formed

An oven-dried 10 mL Schlenk flask equipped with a stir bar was cooled under vacuum. (3-methyl-1H-indol-2-yl)(phenyl)methanol **2x** (32.2 mg, 0.2 mmol, 1.0 equiv.), and $Cu(OTf)_2$ (7.2 mg, 0.02 mmol, 0.10 equiv.) were loaded in the Schlenk flask. (BCB) **1a** (56.4 mg, 0.3 mmol, 1.5 equiv.) and $CHCl_3$ (0.1 M) was added at 60 °C. The reaction was stirred for the 2 h. reaction mixture was diluted with 30 mL CH_2Cl_2. The crude residue was analyzed using ^1H NMR spectroscopy.

This experiment indicates that stabilization of the cationic indolenium species, generated via Lewis acid activation of the indolyl methanol, is crucial for product formation. In the absence of substitution, the reaction does not proceed, likely due to insufficient stabilization of the indolenium intermediate. In contrast, the presence of an aryl substituent provides resonance stabilization to the indolenium ion, thereby enabling the formation of the tetracyclic indole product.

8.5. Hammett Studies

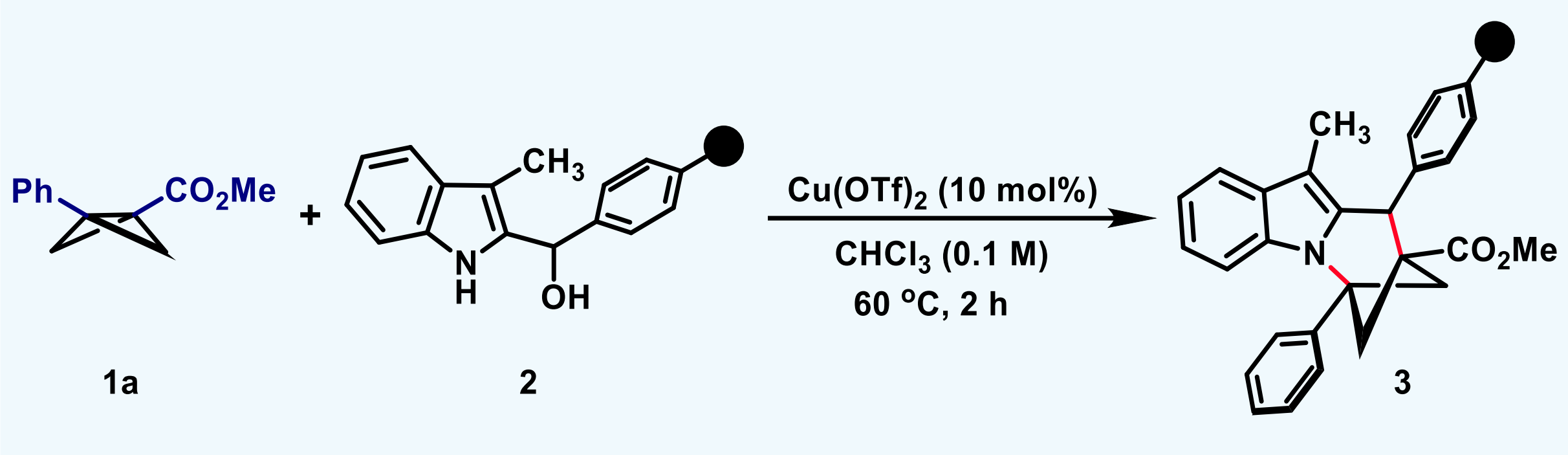

Following the general procedure GP5 for the cycloaddition reaction, the reaction of BCB **1a** (1.5 eq.) and p-substituted aryl of 2-indolylmethanol (1.0 eq.) were performed in presence of $Cu(OTf)_2$ for 5 minutes. Upon completion of the reaction the crude product was analyzed by ^{1}H NMR spectroscopy using trichloroethylene (TCE) as internal standard. The k_X/k_H was estimated based on the product formed in each case.

Substituents	Log(k_X/k_H)	σ
-^{t}Bu	0.18	-0.2
-CH_3	0.15	-0.17
-H	0	0
-F	-0.08	0.06
-CF_3	-0.39	0.54

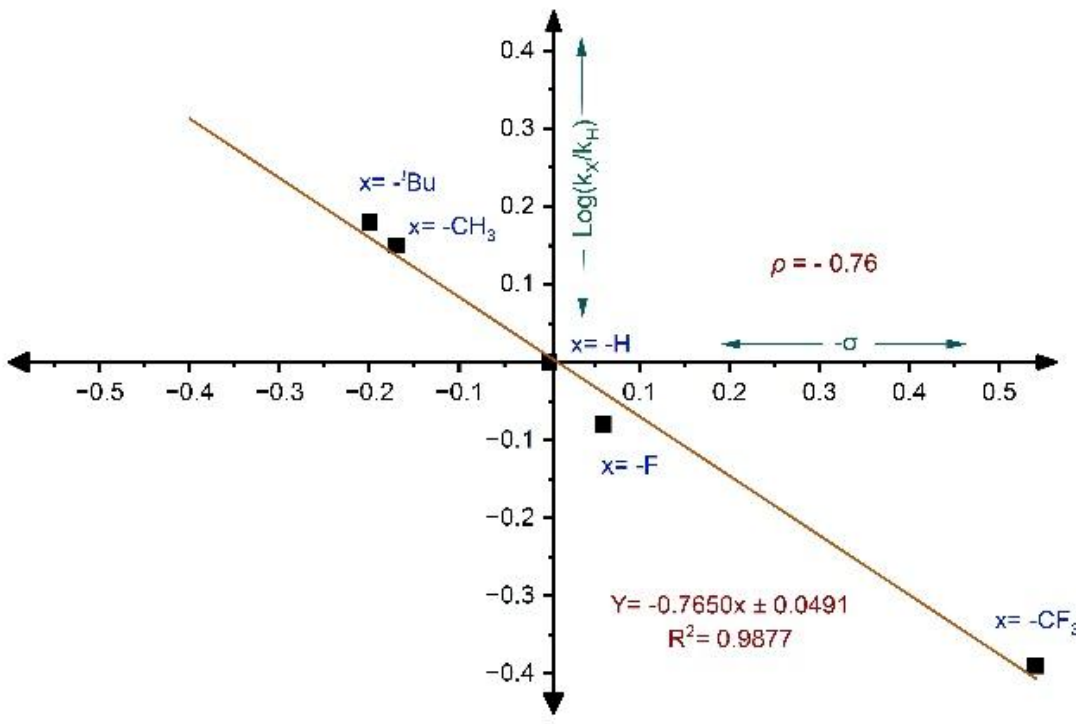

Hammett Plot of log(k_X/k_H) vs. σ

A negative linear correlation was observed when plotting log(k_X/k_H) against σ, indicating a linear free-energy relationship (ρ = - 0.76). The reaction is likely faster when electron-donating groups are present at the 4-position of aryl moiety attached to of 2-indolylmethanol.

8.6. Reaction with electronically poor BCB

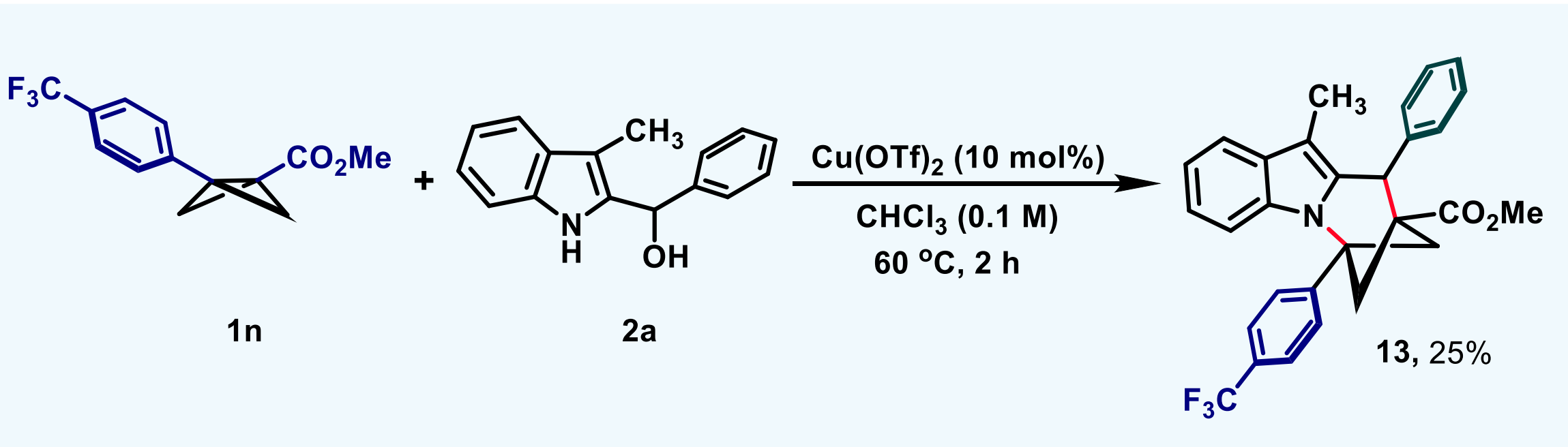

According to the general procedure GP5 an oven-dried 10 mL Schlenk flask equipped with a stir bar was cooled under vacuum. (1*H*-indol-2-yl)diphenylmethanol **2a** (47.4 mg, 0.2 mmol, 1.0 equiv.), and

$Cu(OTf)_2$ (7.2 mg, 0.02 mmol, 0.10 equiv.) were loaded in the Schlenk flask. Methyl 3-(4-(trifluoromethyl)phenyl)bicyclo[1.1.0]butane-1-carboxylate (BCB) **1n** (76.8 mg, 0.3 mmol, 1.5 equiv.), and $CHCl_3$ (0.1 M) was added at the mentioned temperature. The reaction was stirred for the 2h. reaction mixture was diluted with 10 mL CH_2Cl_2. The crude residue was purified by column chromatography on silica gel (hexane/ethyl acetate = 9.6:0.4) gave **13** as a white solid (23.7 mg, 25%).

Rf = 0.35 (hexane/ethyl acetate = 9/1).

M.P. = 192 – 194 °C

^{1}H NMR ((400 MHz, $CDCl_3$) δ (ppm)): δ 7.87 – 7.64 (m, 3H), 7.57 – 7.49 (m, 1H), 7.44 (dt, *J* = 7.9, 1.1 Hz, 1H), 7.33 – 7.26 (m, 3H), 7.20 (dd, *J* = 7.6, 2.0 Hz, 2H), 6.93 (ddd, *J* = 7.9, 7.0, 0.9 Hz, 1H), 6.69 (ddd, *J* = 8.5, 7.0, 1.3 Hz, 1H), 5.29 (dt, *J* = 8.5, 0.8 Hz, 1H), 5.15 (s, 1H), 3.64 (s, 3H), 2.77 (dd, *J* = 9.6, 7.6 Hz, 4H), 1.84 (d, *J* = 0.9 Hz, 3H).

^{13}C {^{1}H} NMR ((101 MHz, $CDCl_3$) δ (ppm)): δ 173.48, 143.62, 139.88, 137.44, 134.79, 133.18, 131.15, 129.40, 128.70, 128.54, 128.47, 127.50, 125.93 (q, *J* = 13 Hz), 121.07, 118.48, 118.38, 110.80, 109.26, 61.87, 51.95, 48.30, 47.60, 47.30, 39.55, 8.59.

^{19}F NMR ((376 MHz, $CDCl_3$) δ (ppm)) δ -62.35.

HRMS (ESI) Calcd. for $C_{29}H_{24}F_3NO_2$ $[M+H]^+$, 476.1832, found: 476.1846.

FTIR (cm^{-1}) : 1018, 1066, 1117, 1164, 1224, 1324, 1450, 1741, 2919.

8.7. Cation trapping experiment

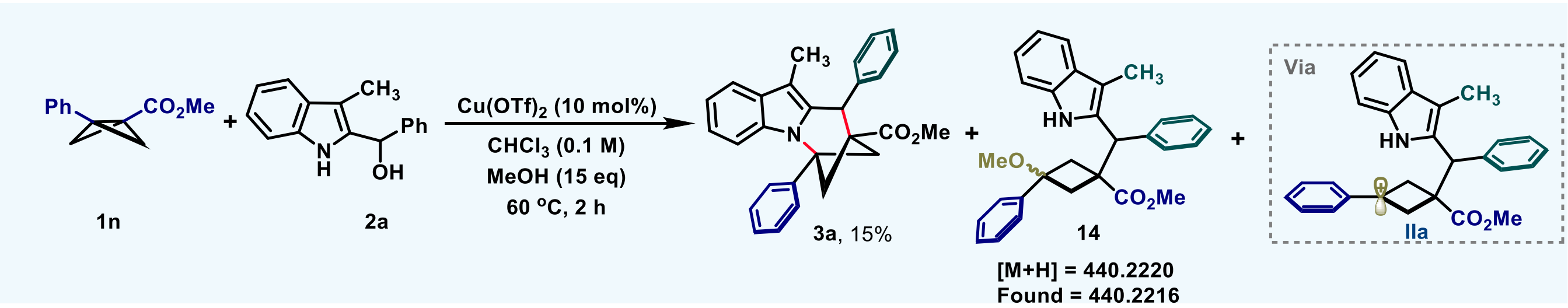

According to the general procedure GP5 an oven-dried 10 mL Schlenk flask equipped with a stir bar was cooled under vacuum. (1H-indol-2-yl)diphenylmethanol **2a** (47.4 mg, 0.2 mmol, 1.0 equiv.), and $Cu(OTf)_2$ (7.2 mg, 0.02 mmol, 0.10 equiv.) were loaded in the Schlenk flask. methyl 3-phenylbicyclo[1.1.0]butane-1-carboxylate (BCB) **1a** (56.4 mg, 0.3 mmol, 1.5 equiv.), $CHCl_3$ (0.1 M) and MeOH (3 mmol, 15 equiv.) was added at the mentioned temperature. The reaction was stirred for the 2h. reaction mixture was diluted with 10 mL CH_2Cl_2. The crude residue was analyzed using ^{1}H NMR spectroscopy.

In conclusion, the formation of the MeOH-trapped intermediate ***IIa*** *sheds light on the reaction mechanism, indicating that it proceeds through a discrete, reactive intermediate that can be trapped by methanol.*

8.8. Checking Hidden Brønsted acid catalysis

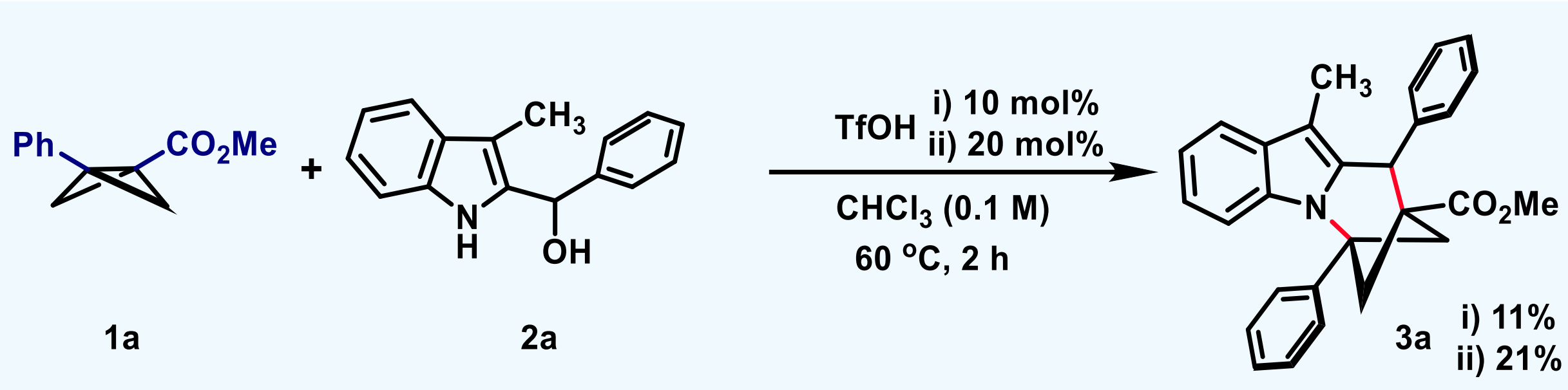

According to the general procedure GP5 an oven-dried 10 mL Schlenk flask equipped with a stir bar was cooled under vacuum. (3-methyl-1*H*-indol-2-yl)(phenyl)methanol **2a** (47.4 mg, 0.2 mmol, 1.0 equiv.), and TfOH (0.02 mmol, 0.10 equiv./ 0.04 mmol, 0.10 equiv.) were loaded in the Schlenk flask. methyl 3-phenylbicyclo[1.1.0]butane-1-carboxylate (BCB) **1a** (56.4 mg, 0.3 mmol, 1.5 equiv.), and $CHCl_3$ (0.1 M) was added at the mentioned temperature. The reaction was stirred for the 2h. reaction mixture was diluted with 10 mL CH_2Cl_2. The crude residue was analyzed using ^{1}H NMR spectroscopy.

In conclusion, the Brønsted acid does not catalyse the reaction.

8.9. Reaction without Catalyst

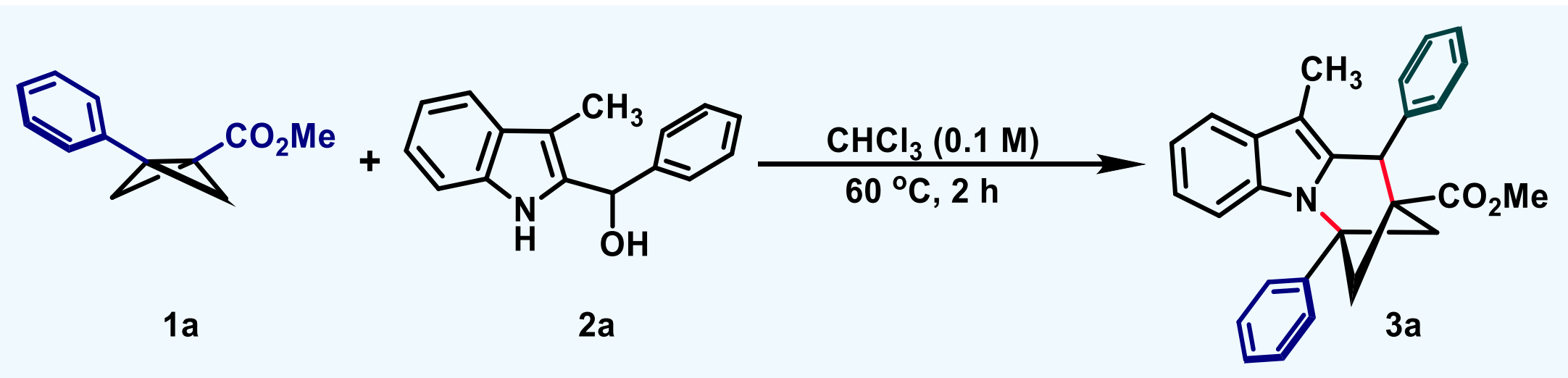

According to the general procedure GP5 an oven-dried 10 mL Schlenk flask equipped with a stir bar was cooled under vacuum. (3-methyl-1*H*-indol-2-yl)(phenyl)methanol **2a** (47.4 mg, 0.2 mmol, 1.0 equiv.) was loaded in the Schlenk flask. methyl 3-phenylbicyclo[1.1.0]butane-1-carboxylate (BCB) **1a** (56.4 mg, 0.3 mmol, 1.5 equiv.), and $CHCl_3$ (0.1 M) was added at the mentioned temperature. The reaction was stirred for the 2h. reaction mixture was diluted with 10 mL CH_2Cl_2. The crude residue was analyzed using ^{1}H NMR spectroscopy.

Since the reaction occurs at elevated temperature, we initially considered that heat alone might activate it; however, our results show that the reaction requires Lewis acid activation.

8.10. Intermediate trapping via HRMS

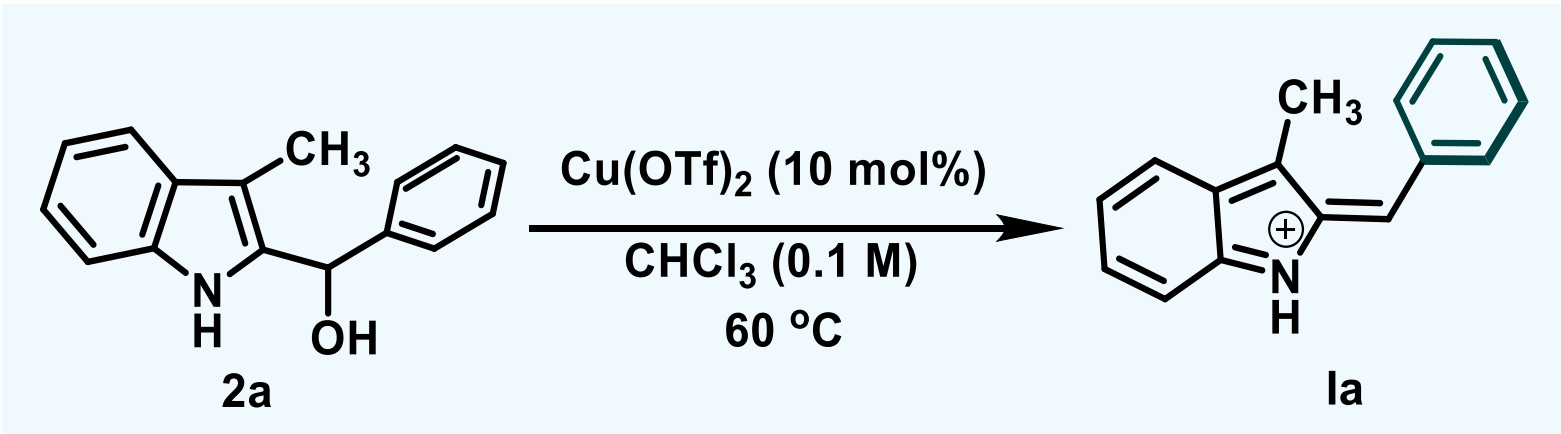

According to the general procedure GP5 an oven-dried 10 mL Schlenk flask equipped with a stir bar was cooled under vacuum. (3-methyl-1*H*-indol-2-yl)(phenyl)methanol **2a** (47.4 mg, 0.2 mmol, 1.0 equiv.) was loaded in the Schlenk flask. $CHCl_3$ (0.1 M) was added at the mentioned temperature. The reaction was stirred for the 10 min. The crude residue was analyzed using HRMS.

HRMS (ESI) for $C_{16}H_{14}N$ [Calcd.] , 220.1121, found: 220.1131.

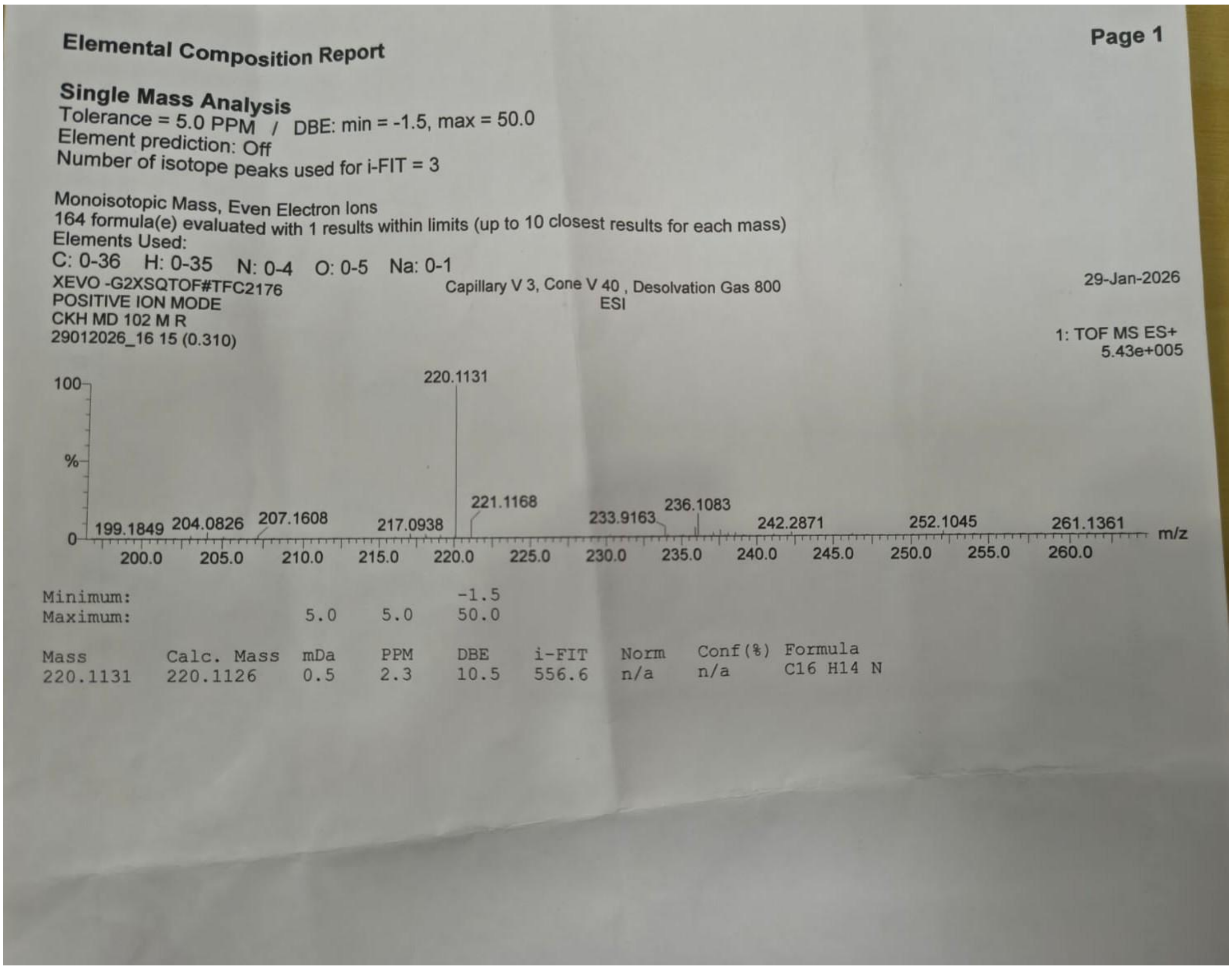

Elemental Composition Report

Page 1

Single Mass Analysis
Tolerance = 5.0 PPM / DBE: min = -1.5, max = 50.0
Element prediction: Off
Number of isotope peaks used for i-FIT = 3

Monoisotopic Mass, Even Electron Ions
164 formula(e) evaluated with 1 results within limits (up to 10 closest results for each mass)
Elements Used:
C: 0-36 H: 0-35 N: 0-4 O: 0-5 Na: 0-1
XEVO -G2XSQTOF#TFC2176
POSITIVE ION MODE
CKH MD 102 M R
29012026_16 15 (0.310)

Capillary V 3, Cone V 40 , Desolvation Gas 800
ESI

29-Jan-2026

1: TOF MS ES+
5.43e+005

Minimum:			-1.5
Maximum:	5.0	5.0	50.0

Mass	Calc. Mass	mDa	PPM	DBE	i-FIT	Norm	Conf(%)	Formula
220.1131	220.1126	0.5	2.3	10.5	556.6	n/a	n/a	C16 H14 N

9. DFT Calculations

All calculations were conducted using a DFT method as implemented in the Gaussian 16 suite of programs.[9] For geometry optimization and frequency analysis, the PBE0 functional[10] in addition to including the Grimme dispersion corrections including Becke-Johnson damping (D3BJ)[11] was employed. Geometry optimizations utilized the def2-SVP[12] split-valence plus single polarization basis set for non-metals. No symmetry constraints were imposed during geometry optimizations. Single-point energy refinements were performed using the hybrid-meta-GGA M06 functional[13] with the def2-TZVP[12] basis set. Solvation energies were calculated implicitly for all intermediates and transition states using a self-consistent reaction field (SCRF) approach in chloroform solvent (ε = 4.71) and the SMD continuum solvation model.[14] Free energies (ΔG) reported throughout the article, unless stated otherwise, were determined at the M06(SMD, DCM)/def2-TZVP//PB0-D3(BJ)/def2-SVP level. The ΔG values were obtained by adding the in-solvent electronic energy (ΔE) at M06(SMD, DCM)/def2-TZVP to the free energy corrections computed at PBE0-D3(BJ)/def2-SVP in the gas phase.

Results

To gain deeper insight into the reaction mechanism and the formation of an exclusive product, we have carried out quantum chemical calculations using density functional theory (DFT). The reaction starts with BCB carboxylate, **1a** reacts with methylene carbon of protonated indolyl methanol (**A**), generating carbocation intermediate B, which is highly exergonic intermediate species **IIa** (-44.1 kcal/mol). Next, **IIa** undergoes cyclization go gives spiro-intermediate **IIIab** (-57.0 kcal/mol). The reaction will complete by enlarging the spiro-intermediate **IIIab** undergoes selective tertiary or secondary migration of group to generate desired products. The transition state barrier for **TS$_1$** (transfer of tertiary group), is observed 6.8 kcal/mol lower in energy barrier as compared to transfer of secondary group having transition state **TS$_2$**. Finally, the deprotonation gives the highly stable desired product **3a** and **3a'**.

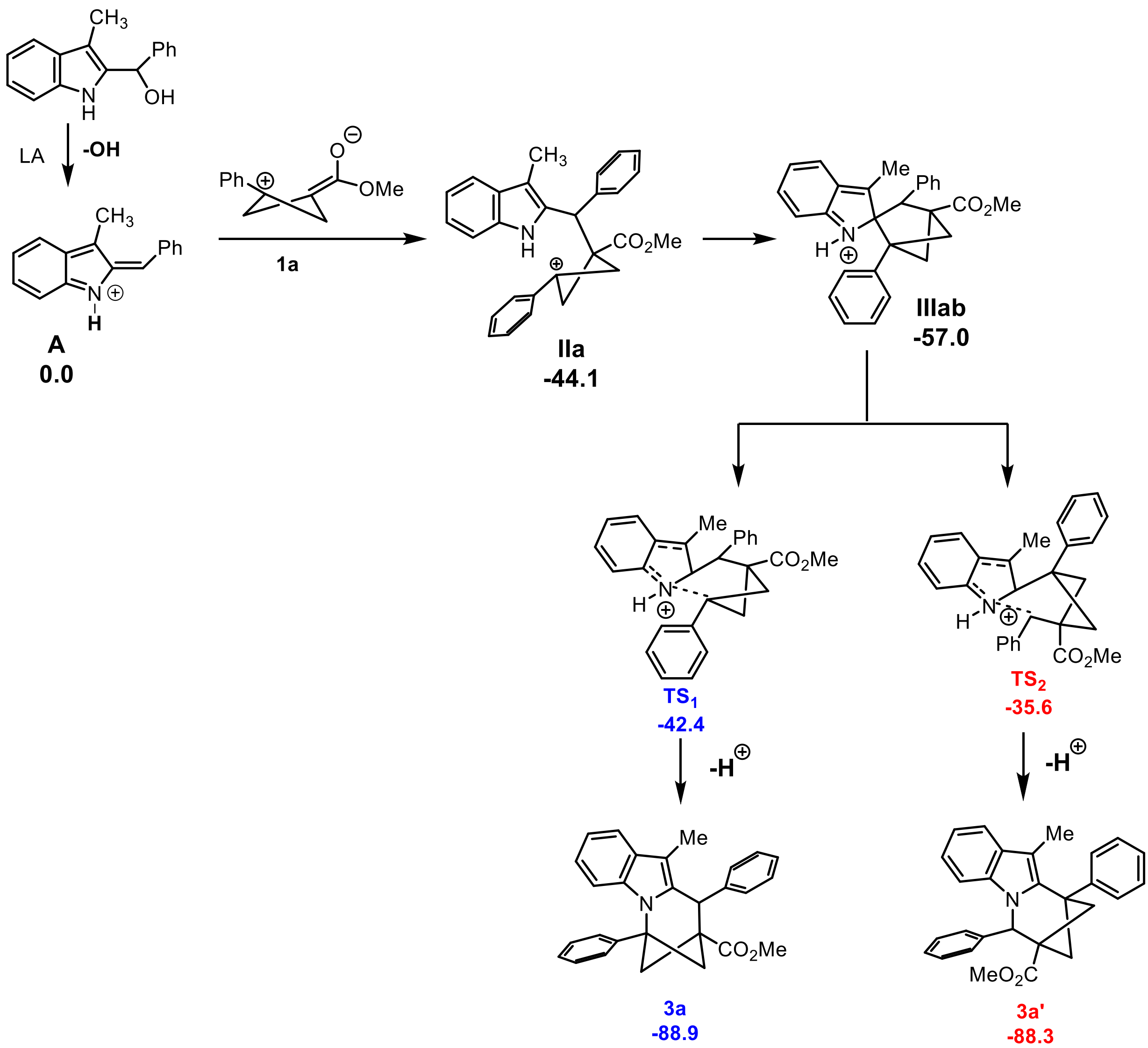
CH3
Ph
OH
N
H
LA
-OH
CH3
Ph
N
H
A
0.0
Ph
O
OMe
1a
CH3
CO2Me
IIa
-44.1
Me
Ph
CO2Me
H
N
IIIab
-57.0
Me
Ph
CO2Me
N
H
TS1
-42.4
Me
N
H
Ph
CO2Me
TS2
-35.6
-H
-H
Me
N
CO2Me
3a
-88.9
Me
N
MeO2C
3a'
-88.3

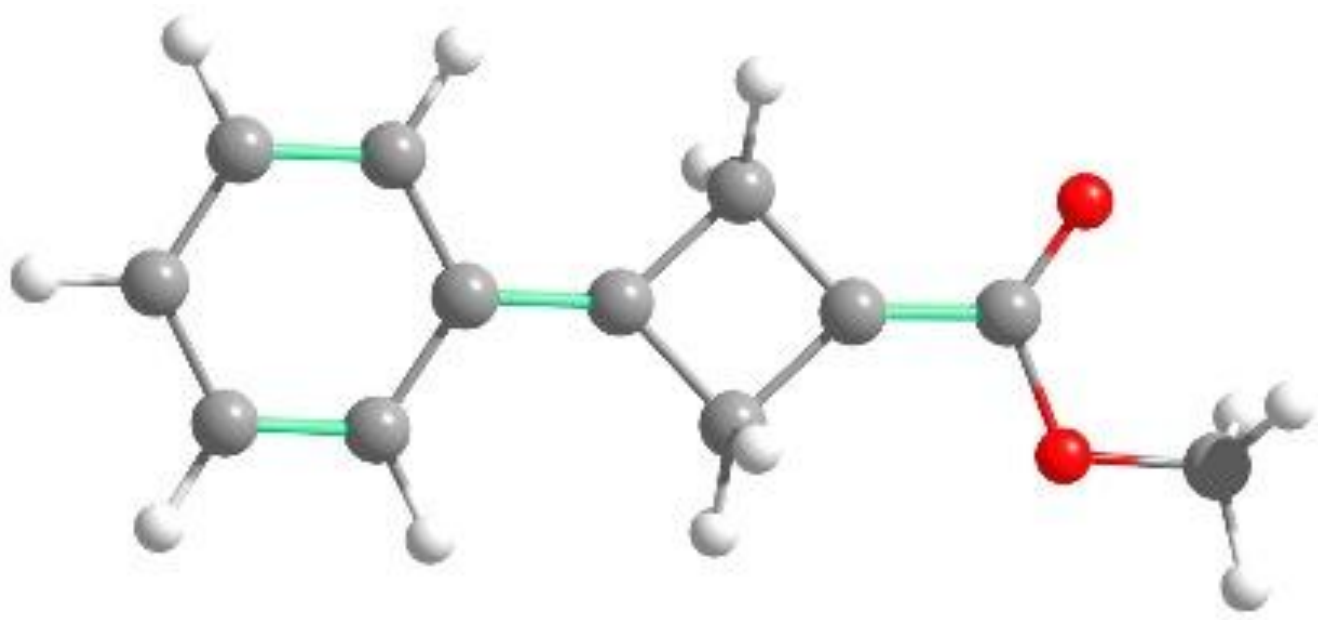

1a

E_e^S = -614.6317136

C	0.300958000	-0.483678000	3.077616000
C	0.735877000	-2.557457000	3.189731000
C	1.527865000	-1.304934000	3.257204000
H	2.369876000	-1.093793000	3.930922000
H	1.889337000	-1.236383000	2.197634000
C	-0.486501000	-1.741871000	3.012548000
H	-1.337195000	-1.961731000	2.352794000
H	-0.853141000	-1.712511000	4.073451000
C	0.010874000	0.897650000	2.995751000
C	1.017644000	1.870682000	3.217528000
C	-1.296298000	1.353185000	2.690109000
C	0.728758000	3.223321000	3.128935000
C	-1.576773000	2.708657000	2.619940000
C	-0.568582000	3.653531000	2.834128000
H	2.032095000	1.544084000	3.458647000
H	-2.088268000	0.622082000	2.510941000
H	1.520529000	3.957005000	3.298320000
H	-2.592228000	3.038711000	2.388040000
H	-0.792910000	4.720479000	2.771513000
C	0.976530000	-3.952279000	3.280231000
O	0.131104000	-4.828653000	3.224975000
O	2.300573000	-4.225556000	3.443866000
C	2.621092000	-5.594340000	3.534502000
H	2.130797000	-6.065667000	4.400970000
H	2.304277000	-6.142565000	2.633229000
H	3.711460000	-5.652973000	3.644506000

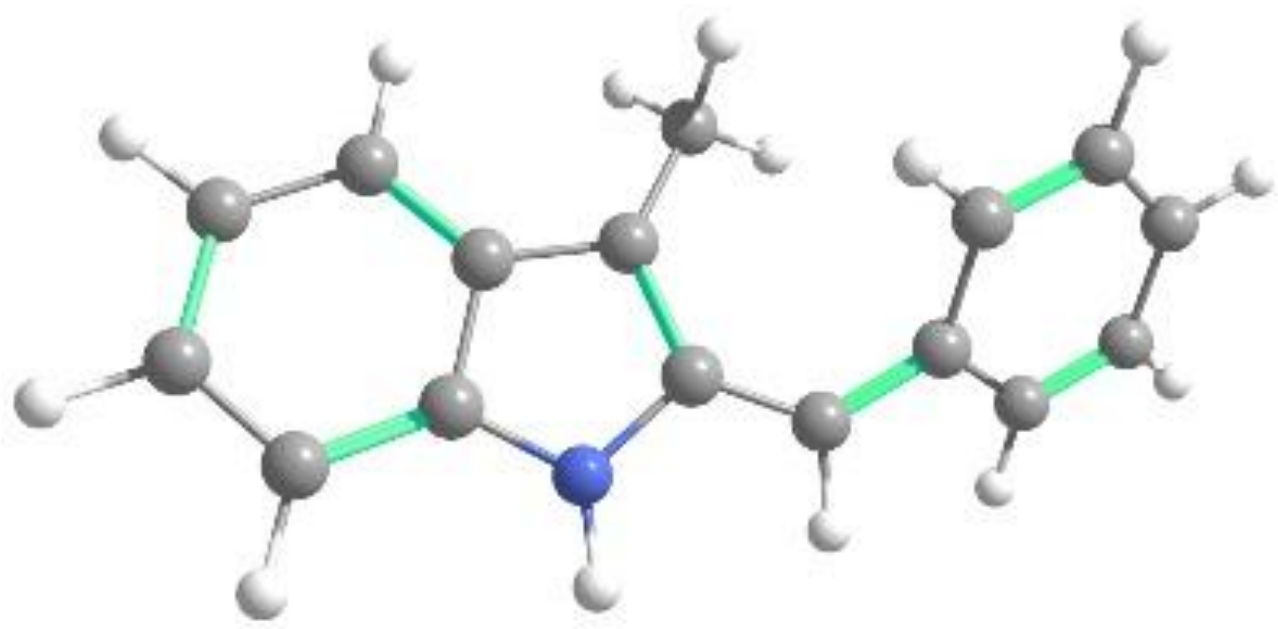

A

E_e^S = -672.4437961

C	0.349110000	0.331609000	-1.881844000
C	0.277989000	-0.497850000	-0.730121000
C	-0.115335000	0.067122000	0.527707000

C	-0.403827000	1.432843000	0.663654000
C	-0.311180000	2.205150000	-0.477269000
C	0.060136000	1.665218000	-1.742125000
C	0.524263000	-1.864407000	-0.537494000
C	0.268344000	-2.127729000	0.859118000
N	-0.146775000	-0.927723000	1.439466000
H	0.640458000	-0.083546000	-2.848451000
H	-0.697415000	1.864603000	1.621647000
H	-0.535520000	3.272537000	-0.411375000
H	0.113779000	2.331714000	-2.604596000
C	0.230743000	-3.296090000	1.593901000
H	-0.327819000	-3.229414000	2.536302000
C	0.837004000	-2.825219000	-1.624041000
H	1.919348000	-2.861376000	-1.828540000
H	0.514826000	-3.846404000	-1.381767000
H	0.344977000	-2.511603000	-2.555244000
C	0.810616000	-4.576932000	1.328285000
C	1.984832000	-4.741926000	0.558159000
C	0.219371000	-5.715468000	1.926191000
C	2.514087000	-6.005732000	0.354207000
C	0.737875000	-6.978958000	1.694126000
C	1.883006000	-7.125650000	0.905545000
H	2.508765000	-3.863607000	0.180445000
H	-0.666978000	-5.594146000	2.553872000
H	3.433997000	-6.125768000	-0.221428000
H	0.262256000	-7.855534000	2.138035000
H	2.302913000	-8.120451000	0.739317000

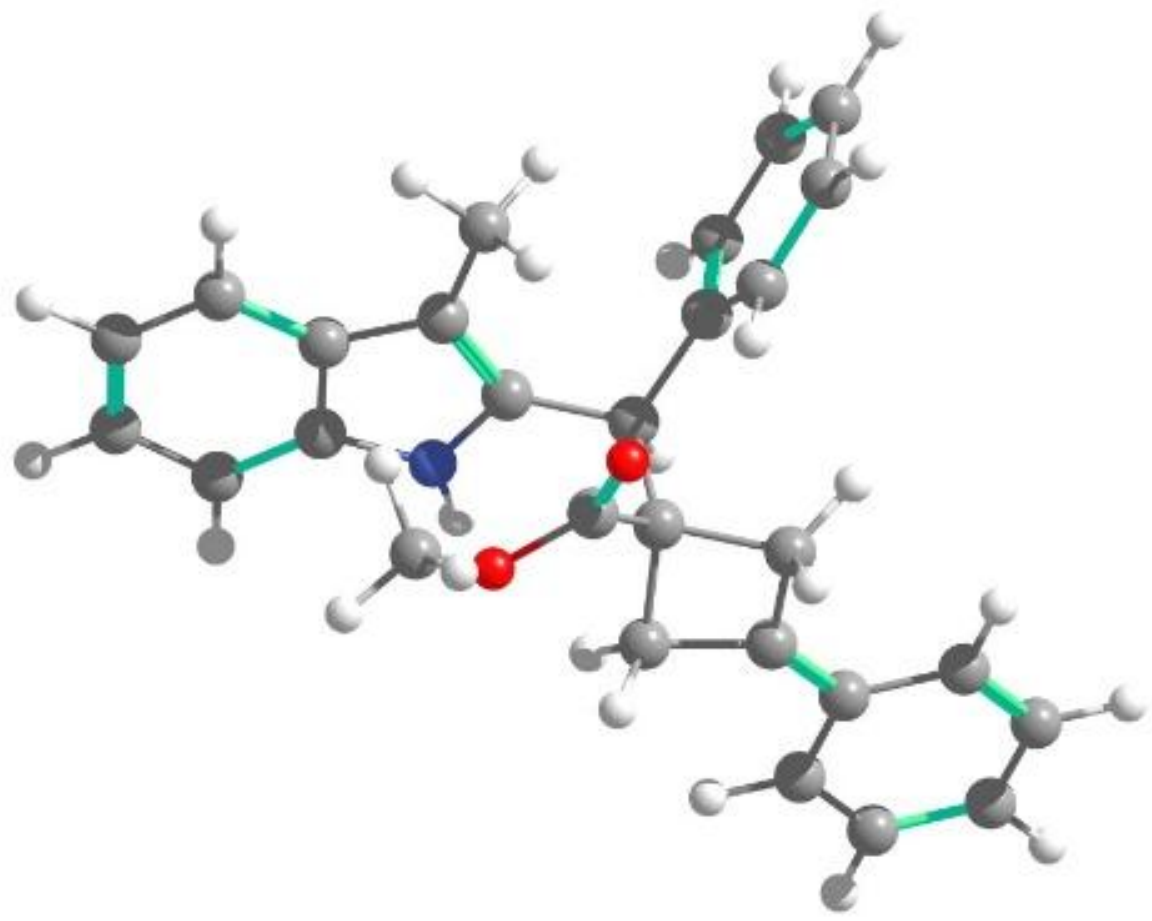

IIa

E_e^S = -1287.176493

C	1.597829000	-3.177349000	-0.909017000
C	1.449852000	-0.155159000	-0.706695000
H	-0.184956000	-3.073596000	0.286038000
C	3.184515000	1.306647000	-1.736075000
H	2.502287000	1.411784000	-2.584717000

C	2.439897000	-2.428110000	0.048316000
C	0.352425000	-2.493607000	-0.485314000
C	2.516193000	-5.772908000	-4.063567000
C	1.261299000	-1.418663000	0.185817000
H	-0.385112000	-2.202398000	-1.246703000
H	2.599085000	-2.972286000	0.996891000
H	3.418455000	-2.064551000	-0.301538000
C	0.850497000	-1.055719000	1.603339000
O	1.623465000	-0.802460000	2.490007000
O	-0.467647000	-1.062908000	1.724988000
C	-1.005436000	-0.577482000	2.953855000
H	-2.080843000	-0.778799000	2.915440000
H	-0.539109000	-1.086186000	3.808000000
H	-0.828695000	0.504831000	3.028971000
H	1.350274000	-0.524968000	-1.746183000
C	2.813130000	0.510443000	-0.645322000
C	3.691790000	0.398527000	0.437214000
H	3.407902000	-0.176022000	1.321121000
C	4.917193000	1.065905000	0.421796000
H	5.588826000	0.974305000	1.278348000
C	5.276099000	1.857557000	-0.665542000
H	6.233025000	2.383971000	-0.671238000
C	4.402428000	1.978603000	-1.747084000
H	4.672739000	2.598840000	-2.604560000
H	4.579978000	-5.591210000	-3.435609000
C	3.548706000	-5.285406000	-3.250225000
C	3.253642000	-4.417330000	-2.217318000
H	4.049179000	-4.029804000	-1.577443000
C	1.906649000	-4.023039000	-1.980402000
H	2.755050000	-6.458444000	-4.880602000
C	0.874856000	-4.526508000	-2.821634000
H	-0.160354000	-4.228012000	-2.642863000
C	1.182534000	-5.396045000	-3.849289000
H	0.394556000	-5.789566000	-4.494022000
C	-1.851958000	3.324293000	1.037358000
C	-1.137186000	2.310060000	0.376068000
C	-3.197299000	3.494621000	0.746166000
H	-1.357069000	3.971679000	1.764839000
C	-1.814505000	1.492283000	-0.562966000
C	0.232225000	1.875331000	0.430114000
C	-3.163808000	1.665548000	-0.868979000
N	-0.895888000	0.590016000	-1.044429000
C	-3.844510000	2.676349000	-0.198101000
H	-3.671757000	1.034329000	-1.601373000
H	-4.903743000	2.837976000	-0.409480000
H	-3.765634000	4.277876000	1.251659000
C	0.332559000	0.811972000	-0.461774000
C	1.286100000	2.486448000	1.293315000
H	0.870922000	3.336341000	1.851174000
H	1.694773000	1.776313000	2.027589000
H	2.133234000	2.859659000	0.699264000

H	-1.058518000	-0.024368000	-1.828573000

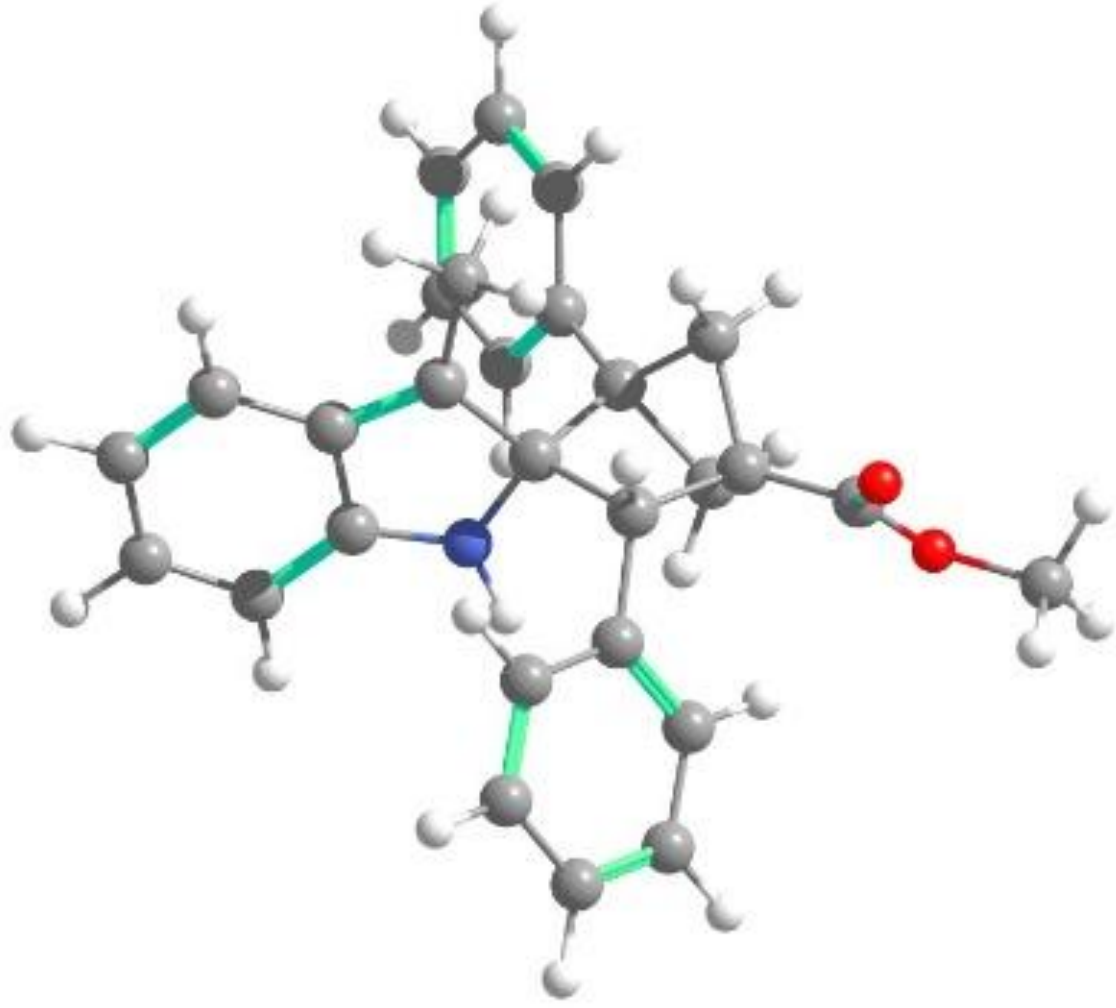

IIIab

E_e^S = -1287.202606

C	-1.967774000	-0.717577000	-1.024526000
C	-1.147607000	-1.215452000	0.033391000
C	0.280446000	-1.315547000	-0.150623000
C	0.885349000	-0.950503000	-1.372598000
C	0.052880000	-0.483012000	-2.360991000
C	-1.364896000	-0.359269000	-2.196362000
C	-1.438809000	-1.677547000	1.301888000
C	-0.161097000	-2.053189000	1.975704000
N	0.827837000	-1.780760000	0.964497000
H	-3.047491000	-0.627720000	-0.893688000
H	1.962494000	-1.035547000	-1.521575000
H	0.488010000	-0.189899000	-3.319936000
H	-1.958182000	0.025788000	-3.027303000
C	-0.065659000	-3.527674000	2.569212000
H	-1.077760000	-3.954743000	2.628430000
C	-2.776405000	-1.747530000	1.916864000
H	-2.962683000	-2.730243000	2.375768000
H	-2.854244000	-0.996266000	2.721060000
H	-3.568370000	-1.543305000	1.186439000
C	0.789613000	-4.447081000	1.742175000
C	2.083157000	-4.820755000	2.122496000
C	0.278536000	-4.923060000	0.527416000
C	2.848845000	-5.649069000	1.299991000
C	1.043940000	-5.743423000	-0.294734000
C	2.335609000	-6.106678000	0.089415000
H	2.498540000	-4.490629000	3.077098000
H	-0.738265000	-4.652997000	0.226227000
H	3.852135000	-5.943694000	1.615743000
H	0.627975000	-6.112038000	-1.235035000
H	2.935976000	-6.756607000	-0.550781000
C	0.152567000	-1.248474000	3.309014000
C	0.398749000	-3.169102000	3.990774000

C	1.406487000	-2.023408000	3.795851000
H	1.765760000	-1.619231000	4.750700000
H	2.261575000	-2.165083000	3.119955000
C	-0.617167000	-2.070615000	4.364421000
H	-1.682422000	-2.298502000	4.238170000
H	-0.438864000	-1.665366000	5.369713000
C	0.076844000	0.236565000	3.211054000
C	1.071290000	0.960917000	2.541186000
C	-1.000723000	0.930726000	3.773679000
C	0.978680000	2.344695000	2.419865000
C	-1.094382000	2.315759000	3.652152000
C	-0.107800000	3.024598000	2.969719000
H	1.936085000	0.440597000	2.123311000
H	-1.765597000	0.387315000	4.335032000
H	1.764615000	2.898534000	1.901615000
H	-1.936977000	2.844789000	4.102758000
H	-0.177499000	4.110656000	2.878283000
C	0.564314000	-4.344280000	4.910476000
O	-0.290201000	-5.176073000	5.058725000
O	1.738317000	-4.337166000	5.533723000
C	1.971163000	-5.413106000	6.439743000
H	1.212204000	-5.416718000	7.234339000
H	1.930604000	-6.374963000	5.909462000
H	2.967975000	-5.246723000	6.861466000
H	1.790527000	-2.084029000	1.045899000

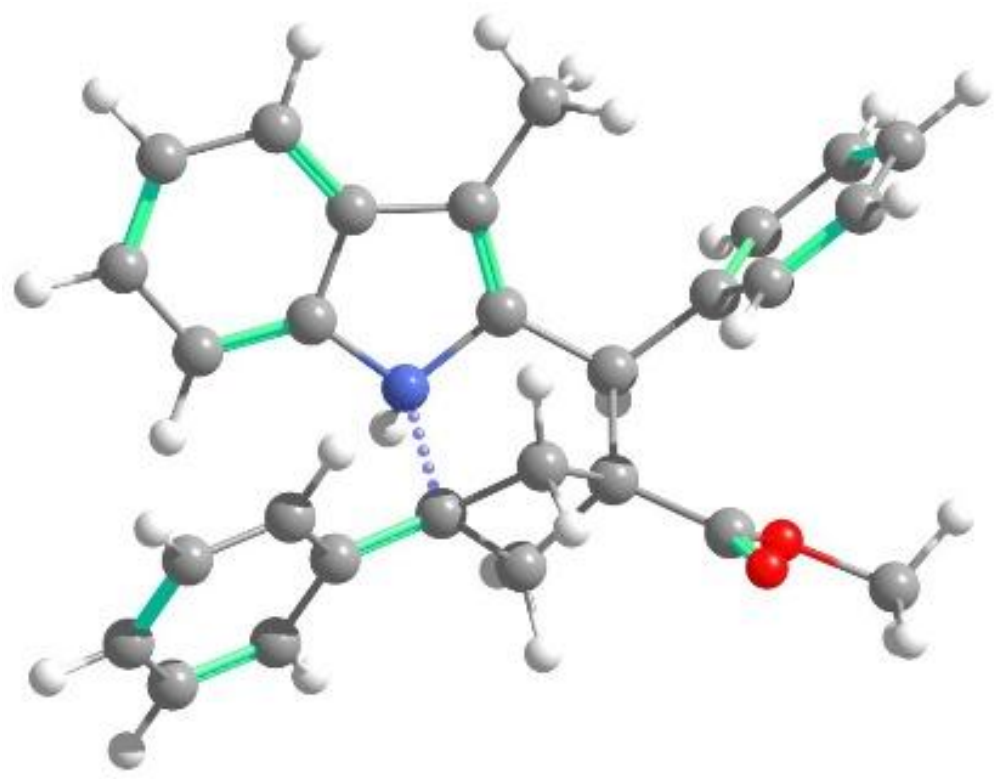

TS1

E_e^S = -1287.182302

C	1.183514000	2.846174000	1.384053000
C	-0.292378000	1.415815000	-0.765886000
H	-0.249429000	4.405896000	1.038470000
C	-1.843872000	0.252325000	-2.359652000
H	-1.026322000	-0.435973000	-2.591520000
C	1.631479000	2.958822000	-0.054874000
C	-0.194989000	3.306636000	0.983531000
C	3.381158000	3.686006000	4.903610000
C	0.135714000	2.875698000	-0.464969000
H	-1.068980000	2.877351000	1.492104000

H	2.031832000	3.971338000	-0.229380000
H	2.345010000	2.234147000	-0.478613000
C	-0.232694000	3.861490000	-1.550983000
O	-0.709862000	4.943690000	-1.337929000
O	0.103313000	3.385085000	-2.741309000
C	-0.200587000	4.185251000	-3.881399000
H	0.624568000	4.062095000	-4.592694000
H	-1.136376000	3.821814000	-4.328627000
H	-0.312099000	5.238689000	-3.594842000
H	0.467976000	1.000599000	-1.450944000
C	-1.632729000	1.293817000	-1.453054000
C	-2.681965000	2.178047000	-1.179264000
H	-2.545699000	3.008278000	-0.482501000
C	-3.921720000	2.014447000	-1.796211000
H	-4.730493000	2.715317000	-1.577927000
C	-4.128279000	0.962910000	-2.687454000
H	-5.100107000	0.834773000	-3.168938000
C	-3.084546000	0.083017000	-2.970511000
H	-3.234734000	-0.735961000	-3.677487000
H	5.148418000	3.461842000	3.678214000
C	4.057224000	3.435534000	3.706327000
C	3.337222000	3.168599000	2.548225000
H	3.864579000	2.988678000	1.607695000
C	1.933043000	3.146308000	2.582118000
H	3.949150000	3.903665000	5.811007000
C	1.262608000	3.393113000	3.790150000
H	0.170878000	3.372283000	3.818507000
C	1.987187000	3.669317000	4.944916000
H	1.464145000	3.870268000	5.881873000
C	-0.997719000	-0.857433000	3.761412000
C	-0.550666000	-0.201275000	2.620812000
C	-0.140556000	-0.924215000	4.865402000
H	-1.983920000	-1.324172000	3.797685000
C	0.722254000	0.378193000	2.612136000
C	-1.153288000	0.017557000	1.300033000
C	1.593169000	0.307250000	3.684360000
N	0.906031000	1.034713000	1.361514000
C	1.134272000	-0.360247000	4.827046000
H	2.592495000	0.743948000	3.651986000
H	1.787145000	-0.438520000	5.698359000
H	-0.471446000	-1.438396000	5.770107000
C	-0.244030000	0.697649000	0.551977000
C	-2.509444000	-0.486926000	0.946297000
H	-2.973112000	-0.983078000	1.808233000
H	-3.175042000	0.318301000	0.608904000
H	-2.457146000	-1.214089000	0.123869000
H	1.784687000	0.754552000	0.919953000

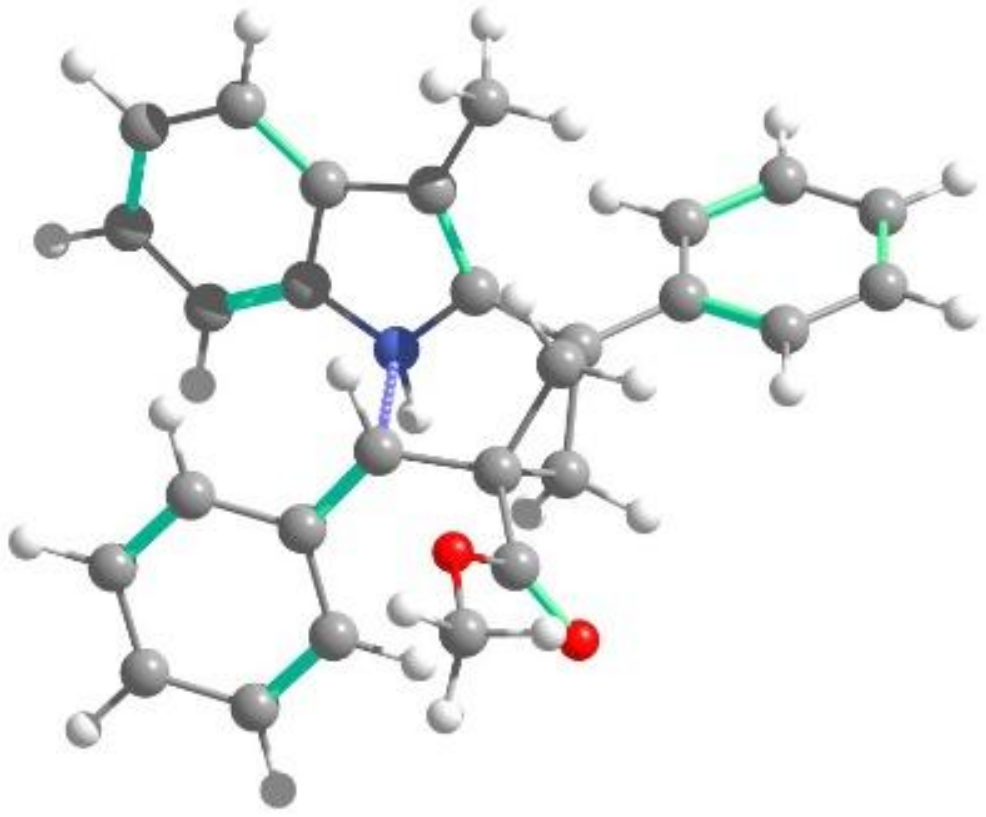

…..**TS2**

E_e^S = -1287.165388

C	-0.176642000	-1.366927000	-0.008521000
C	-0.030225000	1.411961000	-0.797525000
H	0.812362000	-1.706715000	-1.942419000
C	0.991728000	3.536659000	-0.237354000
H	0.194454000	3.690326000	0.493259000
C	-1.307958000	-0.761049000	-0.902354000
C	0.790886000	-0.948760000	-1.148605000
C	-0.233063000	-5.574753000	0.975807000
C	-0.232907000	0.145944000	-1.543765000
H	1.827156000	-0.656153000	-0.925549000
H	-1.677841000	-1.490269000	-1.635090000
H	-2.155951000	-0.274470000	-0.402759000
C	-0.410699000	0.402780000	-3.037484000
O	0.167792000	-0.195821000	-3.899304000
O	-1.316486000	1.350964000	-3.229804000
C	-1.633820000	1.669044000	-4.586272000
H	-2.368295000	2.479665000	-4.542568000
H	-0.731356000	1.991011000	-5.123923000
H	-2.059041000	0.792267000	-5.093658000
H	-0.813428000	1.706764000	-0.090312000
C	0.991107000	2.354463000	-1.026287000
C	2.025169000	2.153459000	-1.982446000
H	2.035613000	1.253106000	-2.601119000
C	3.010654000	3.107823000	-2.137002000
H	3.802158000	2.963658000	-2.874937000
C	2.995210000	4.268610000	-1.345698000
H	3.780061000	5.017575000	-1.478238000
C	1.991166000	4.482730000	-0.398317000
H	1.990872000	5.391208000	0.206689000
H	-0.880715000	-5.868693000	-1.064166000
C	-0.591455000	-5.143086000	-0.300844000
C	-0.581700000	-3.785809000	-0.608178000
H	-0.859735000	-3.474701000	-1.618650000
C	-0.218770000	-2.835105000	0.354627000
H	-0.241716000	-6.639653000	1.217844000
C	0.140718000	-3.279785000	1.631011000

H	0.422526000	-2.554291000	2.397415000
C	0.134952000	-4.639178000	1.939108000
H	0.417964000	-4.966850000	2.941894000
C	-0.803296000	1.751116000	3.983468000
C	-0.362750000	0.984519000	2.895613000
C	0.025410000	2.754407000	4.470067000
H	-1.775184000	1.561041000	4.443928000
C	0.910219000	1.265470000	2.328957000
C	-0.943420000	-0.100490000	2.143254000
C	1.752903000	2.272596000	2.820628000
N	1.073446000	0.423881000	1.268105000
C	1.288998000	3.010595000	3.899026000
H	2.736109000	2.457858000	2.382932000
H	1.915457000	3.799315000	4.320685000
H	-0.302093000	3.355884000	5.320693000
C	-0.037676000	-0.431616000	1.162982000
C	-2.277510000	-0.717291000	2.387661000
H	-3.094497000	0.008811000	2.244644000
H	-2.457419000	-1.577092000	1.729138000
H	-2.351640000	-1.081541000	3.424366000
H	1.968464000	0.233387000	0.836652000

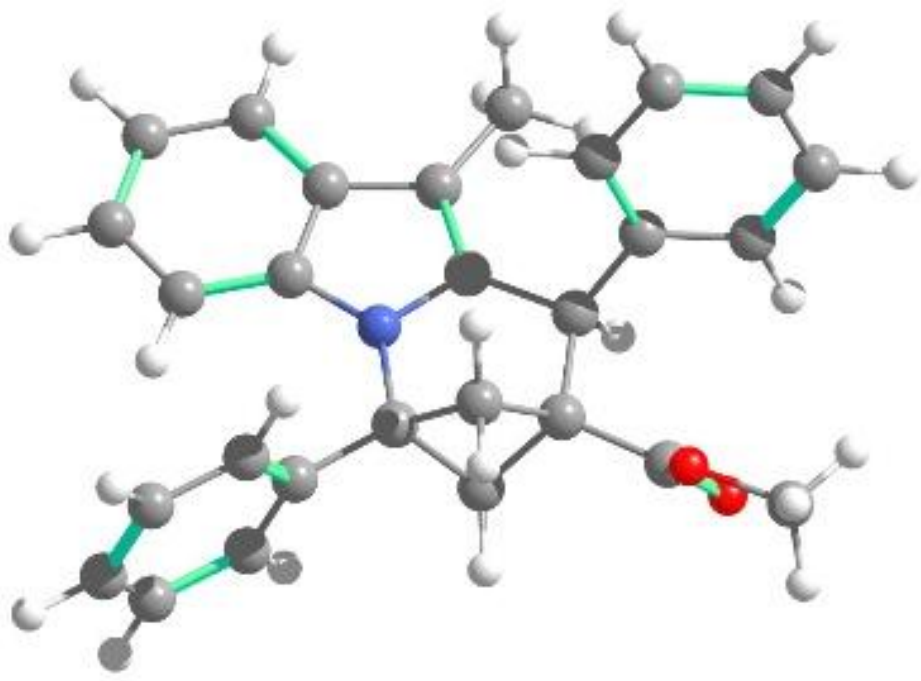

3a

E_e^S = -1286.789054

C	-0.437840000	-0.159235000	-2.135101000
C	-0.300445000	-0.752237000	-0.870596000
C	-0.147887000	0.066181000	0.287663000
C	-0.134108000	1.465448000	0.175965000
C	-0.273598000	2.020030000	-1.089584000
C	-0.424727000	1.222653000	-2.238123000
C	-0.273590000	-2.123726000	-0.450621000
C	-0.104391000	-2.099563000	0.912335000
N	-0.026081000	-0.789183000	1.365211000
H	-0.552224000	-0.785411000	-3.023610000
H	-0.015499000	2.114400000	1.040265000
H	-0.263442000	3.108135000	-1.189243000
H	-0.530592000	1.699132000	-3.215498000
C	0.024832000	-3.223696000	1.900464000
H	-0.909843000	-3.810733000	1.908028000

C	-0.381307000	-3.322476000	-1.328875000
H	0.498829000	-3.423143000	-1.986273000
H	-0.456044000	-4.249469000	-0.743681000
H	-1.267291000	-3.261651000	-1.981337000
C	1.166562000	-4.175461000	1.596227000
C	2.332771000	-3.745757000	0.954317000
C	1.076702000	-5.514411000	1.997096000
C	3.390973000	-4.626021000	0.737299000
C	2.133050000	-6.396296000	1.778414000
C	3.296735000	-5.953550000	1.151417000
H	2.398605000	-2.714885000	0.597446000
H	0.164885000	-5.865767000	2.487007000
H	4.293125000	-4.272723000	0.231941000
H	2.041880000	-7.439117000	2.092377000
H	4.124090000	-6.644983000	0.974897000
C	0.088157000	-0.564746000	2.806640000
C	0.155253000	-2.614593000	3.319262000
C	1.208931000	-1.493992000	3.335616000
H	1.492622000	-1.232152000	4.363204000
H	2.107927000	-1.617009000	2.716592000
C	-0.922343000	-1.519594000	3.492848000
H	-1.892728000	-1.673887000	3.000228000
H	-1.065923000	-1.255957000	4.549869000
C	0.095872000	0.881296000	3.198364000
C	1.302316000	1.576606000	3.310709000
C	-1.105335000	1.554672000	3.435176000
C	1.307715000	2.931961000	3.632774000
C	-1.102410000	2.909470000	3.758011000
C	0.104554000	3.601992000	3.852314000
H	2.244453000	1.054796000	3.126432000
H	-2.052015000	1.016246000	3.346323000
H	2.255815000	3.469213000	3.710143000
H	-2.047216000	3.429213000	3.933116000
H	0.107660000	4.665424000	4.102759000
C	0.183887000	-3.707532000	4.350321000
O	-0.734762000	-4.460117000	4.556153000
O	1.344497000	-3.759612000	5.002186000
C	1.484072000	-4.815216000	5.937590000
H	0.683057000	-4.781169000	6.689998000
H	1.441908000	-5.787933000	5.425492000
H	2.463090000	-4.680155000	6.411061000

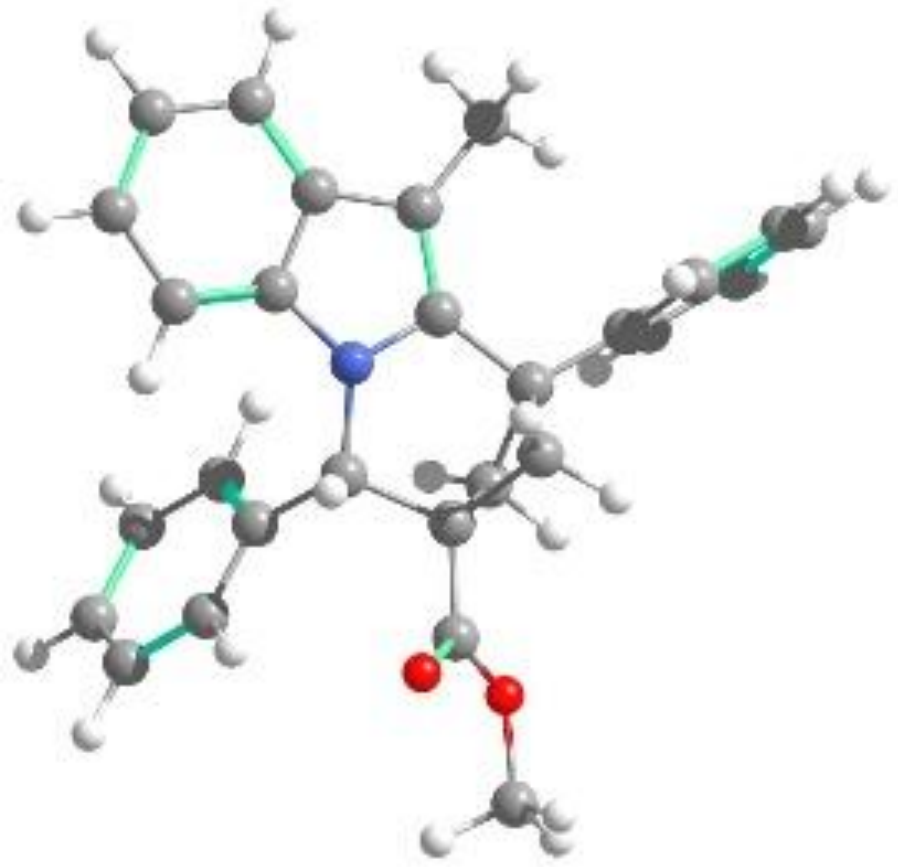

3a'

E_e^S = -1286.788514

C	-0.752464000	0.856776000	1.294040000
C	-0.218327000	-0.414057000	1.551536000
C	1.112461000	-0.694322000	1.149547000
C	1.917781000	0.256020000	0.517245000
C	1.360171000	1.506329000	0.275293000
C	0.039952000	1.804381000	0.658082000
C	-0.749916000	-1.600518000	2.179295000
C	0.258625000	-2.538116000	2.127067000
N	1.380759000	-1.991570000	1.511394000
H	-1.777406000	1.097052000	1.587619000
H	2.946422000	0.029649000	0.230993000
H	1.961590000	2.272622000	-0.219462000
H	-0.365004000	2.797885000	0.451730000
C	2.640183000	-2.686707000	1.366959000
H	2.909555000	-2.746948000	0.298279000
C	-2.137589000	-1.699897000	2.723031000
H	-2.879098000	-1.850127000	1.920295000
H	-2.255850000	-2.522729000	3.437100000
H	-2.409450000	-0.766130000	3.239936000
C	3.767499000	-1.996603000	2.112518000
C	3.514429000	-1.230119000	3.254727000
C	5.087045000	-2.132324000	1.666733000
C	4.561646000	-0.631249000	3.951360000
C	6.134028000	-1.533008000	2.364196000
C	5.875232000	-0.784246000	3.511081000
H	2.483984000	-1.079099000	3.585040000
H	5.290211000	-2.717009000	0.766840000
H	4.347006000	-0.030965000	4.838648000
H	7.159034000	-1.645490000	2.002546000
H	6.695647000	-0.309369000	4.054409000
C	0.401054000	-3.986017000	2.502069000
C	2.421296000	-4.134728000	1.853170000
C	1.757179000	-4.159549000	3.240485000
H	1.838655000	-5.157364000	3.690101000
H	2.058669000	-3.394096000	3.968353000
C	1.083744000	-4.675727000	1.285390000

H	0.796294000	-4.357263000	0.273061000
H	1.030392000	-5.770280000	1.374103000
C	-0.831770000	-4.612164000	3.079157000
C	-1.000865000	-4.715280000	4.462340000
C	-1.845747000	-5.078610000	2.236559000
C	-2.169103000	-5.258187000	4.995079000
C	-3.012455000	-5.623761000	2.766117000
C	-3.179392000	-5.711104000	4.148187000
H	-0.214347000	-4.353851000	5.129455000
H	-1.723241000	-4.998904000	1.153253000
H	-2.290989000	-5.327689000	6.078733000
H	-3.798614000	-5.980713000	2.096410000
H	-4.095313000	-6.136727000	4.564745000
C	3.625491000	-4.984976000	1.552032000
O	4.234842000	-4.948178000	0.512159000
O	3.914610000	-5.827314000	2.542110000
C	5.013423000	-6.694763000	2.316509000
H	4.838824000	-7.324062000	1.431663000
H	5.934785000	-6.116589000	2.155046000
H	5.105297000	-7.314903000	3.215288000

10. X-ray Data of 3a

Single crystal of **3a** (recrystallized from $CDCl_3$/n-hexane at 25 °C) was mounted and the diffraction data was collected at 150 K on a Bruker APEX-II CCD diffractometer using SMART/SAINT software. Intensity data were collected using MoKα radiation (λ=0.71073 A°). The single crystal was affixed to a Hampton Research cryoloop using Paratone-N oil. Data collection and reduction was performed using Bruker APEX2 and Bruker SAINT, respectively. The structure was solved by direct methods using the SHELX-97 and refined by full-matrix leastsquares on F2. Empirical absorption corrections were applied with SADABS. All Nonhydrogen atoms were refined anisotropically and hydrogen atoms were included in geometric positions. Structure was drawn using Olex-2 and Mercury-3. CCDC 2542004 (**3a**) contains the supplementary crystallographic data for this paper. This data can be obtained free of charge from Data Centre via www.ccdc.cam.ac.uk/data_request/cif. The crystallographic refinement parameters are given below:

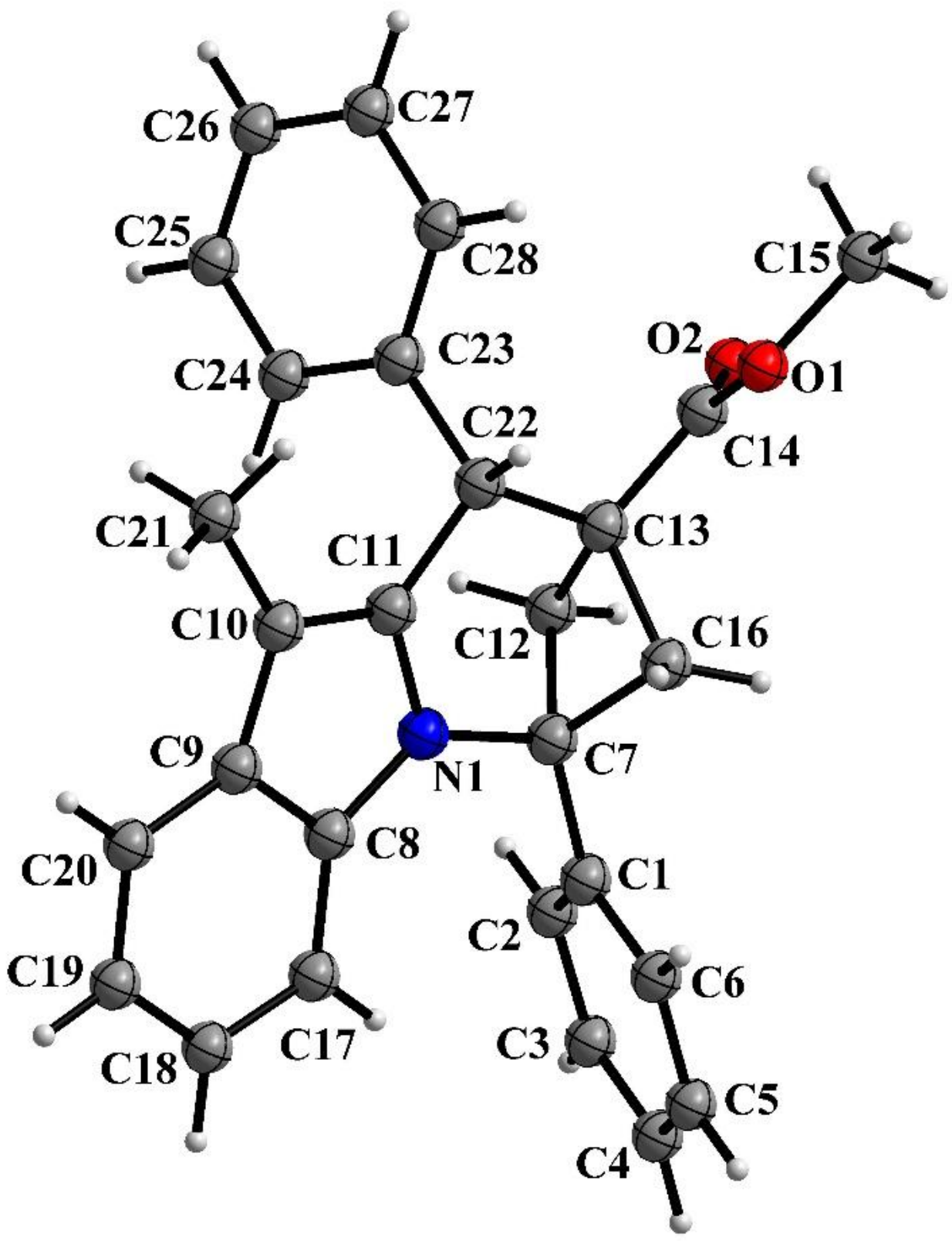

Figure S1. Crystal Structure of 3a

Table 1 Crystal data and structure refinement for 102md_0m_a.

Identification code	102md_0m_a
Empirical formula	$C_{28}H_{25}NO_2$
Formula weight	407.49
Temperature/K	270.00
Crystal system	monoclinic
Space group	$P2_1/n$
a/Å	11.4462(17)
b/Å	10.7222(14)
c/Å	17.927(3)
α/°	90
β/°	98.324(6)
γ/°	90
Volume/Å^3	2177.0(5)
Z	4
ρ_{calc}g/cm^3	1.243
μ/mm^{-1}	0.078
F(000)	864.0
Crystal size/mm^3	0.18 × 0.15 × 0.11
Radiation	MoKα (λ = 0.71073)
2Θ range for data collection/°	4.438 to 56.59
Index ranges	$-15 \le h \le 15$, $-14 \le k \le 14$, $-23 \le l \le 23$
Reflections collected	47928
Independent reflections	5399 [R_{int} = 0.0815, R_{sigma} = 0.0441]
Data/restraints/parameters	5399/0/282
Goodness-of-fit on F^2	1.033
Final R indexes [I>=2σ (I)]	R_1 = 0.0552, wR_2 = 0.1333
Final R indexes [all data]	R_1 = 0.0900, wR_2 = 0.1582
Largest diff. peak/hole / e Å^{-3}	0.31/-0.22

11. References:

1. Dasgupta, A., Bhattacharjee, S., Tong, Z., Guin, A., McNamee, R. E., Christensen, K. E., Biju, A. T. & Anderson, E. A. Stereoselective Alder–Ene reactions of bicyclo[1.1.0]butanes: Facile synthesis of cyclopropyl- and aryl-substituted cyclobutenes. *J. Am. Chem. Soc.* **146**, 1196–1203 (2023).
2. Dutta, S., Daniliuc, C. G., Mück-Lichtenfeld, C. & Studer, A. Enantioselective synthesis of tetrahydro-1H-1,3-methanocarbazoles by formal (3+3)-cycloaddition using bicyclo[1.1.0]butanes. *J. Am. Chem. Soc.* **147**, 4249–4257 (2025).
3. Schwartz, B. D., Smyth, A. P., Nashar, P. E., Gardiner, M. G. & Malins, L. R. Investigating bicyclobutane–triazolinedione cycloadditions as a tool for peptide modification. *Org. Lett.* **24**, 1268–1273 (2022).
4. Singha, T., Bapat, N. A., Mishra, S. K. & Hari, D. P. Photoredox-catalyzed strain-release-driven synthesis of functionalized spirocyclobutyl oxindoles. *Org. Lett.* **26**, 6396–6401 (2024).
5. Zhang, F., Luo, Y., Liu, X., Liu, Y. & Xu, J. NCS-mediated direct C(sp^3)–H oxygenation of 2-methylindoles using water as the oxygen source. *J. Org. Chem.* **89**, 14586–14590 (2024).
6. Bera, K. & Schneider, C. Brønsted acid catalyzed [3+2]-cycloaddition of 2-vinylindoles with in situ generated 2-methide-2H-indoles: Highly enantioselective synthesis of pyrrolo[1,2-a]indoles. *Chem. Eur. J.* **22**, 7074–7078 (2016).
7. Li, Q., Han, P., Wang, T. & Wang, Y. Q. Enantioselective synthesis of spiro-bis-N-heterocyclic lactams via formal (3+2) cycloaddition of 3-methylene isoindolinones with C3-substituted 2-indolylmethanols. *Org. Lett.* **27**, 13318–13324 (2025).
8. Pitzer, L., Schäfers, F. & Glorius, F. Rapid assessment of the reaction-condition-based sensitivity of chemical transformations. *Angew. Chem. Int. Ed.* **58**, 8572–8576 (2019).
9. Frisch, M. J. *et al.* Gaussian 16, Revision C.02 (Gaussian, Inc., Wallingford, CT, 2019).
10. Adamo, C. & Barone, V. Toward reliable density functional methods without adjustable parameters: The PBE0 model. *J. Chem. Phys.* **110**, 6158–6170 (1999).
11. a) Grimme, S., Ehrlich, S. & Goerigk, L. Effect of the damping function in dispersion-corrected density functional theory. *J. Comput. Chem.* **32**, 1456–1465 (2011). b) Grimme, S., Antony, J., Ehrlich, S. & Krieg, H. A consistent and accurate *ab initio* parametrization of density functional dispersion correction (DFT-D) for the 94 elements H–Pu. *J. Chem. Phys.* **132**, 154104 (2010).
12. Weigend, F. & Ahlrichs, R. Balanced basis sets of split valence, triple zeta valence and quadruple zeta valence quality for H to Rn: Design and assessment of accuracy. *Phys. Chem. Chem. Phys.* **7**, 3297–3305 (2005).
13. Zhao, Y. & Truhlar, D. G. The M06 suite of density functionals for main group thermochemistry, thermochemical kinetics, non-covalent interactions, excited states, and

transition elements: Two new functionals and systematic testing of four M06-class functionals and 12 other functionals. *Theor. Chem. Acc.* **120**, 215–241 (2008).

14. Marenich, A. V., Cramer, C. J. & Truhlar, D. G. Universal solvation model based on solute electron density and on a continuum model of the solvent defined by the bulk dielectric constant and atomic surface tensions. *J. Phys. Chem. B* **113**, 6378–6396 (2009).

^{1}H, ^{13}C{^{1}H} and ^{19}F{^{1}H}-NMRs

Methyl-3-phenylbicyclo[1.1.0]butane-1-carboxylate (1a):

^{1}H NMR (400 MHz, $CDCl_3$)

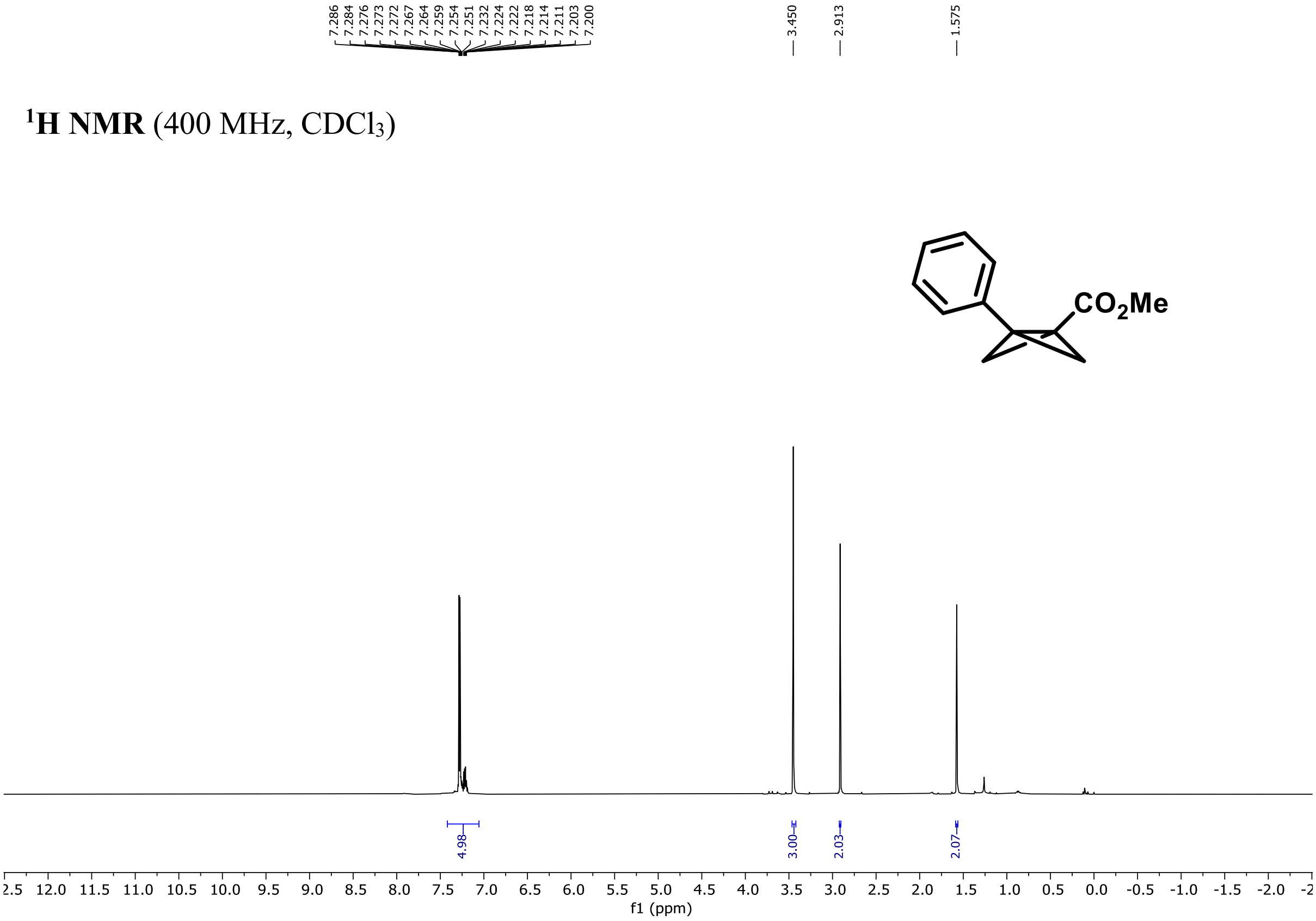

^{13}C {^{1}H} NMR (101 MHz, $CDCl_3$)

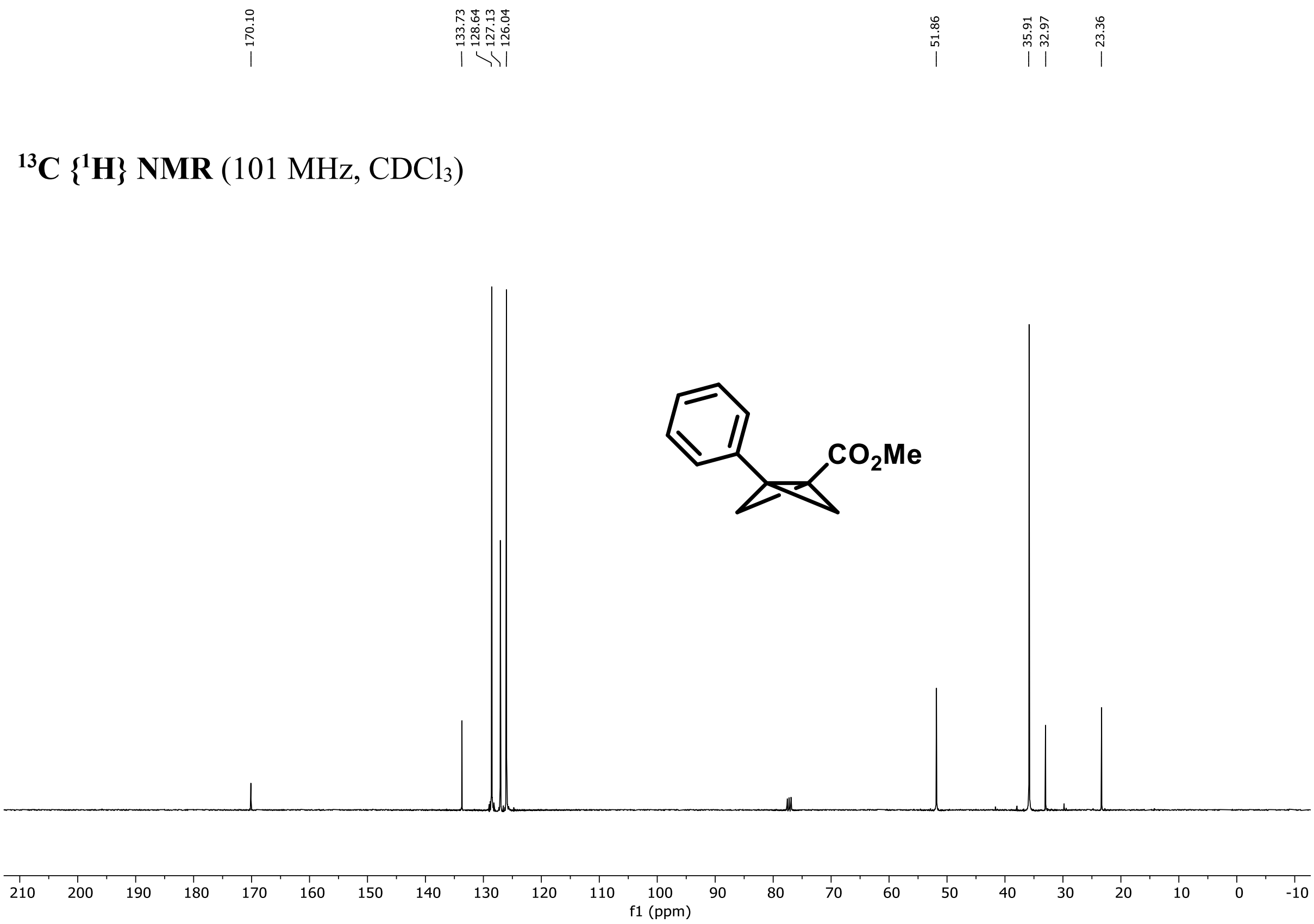

Ethyl 3-phenylbicyclo[1.1.0]butane-1-carboxylate (1b):

^{1}H NMR (400 MHz, $CDCl_3$)

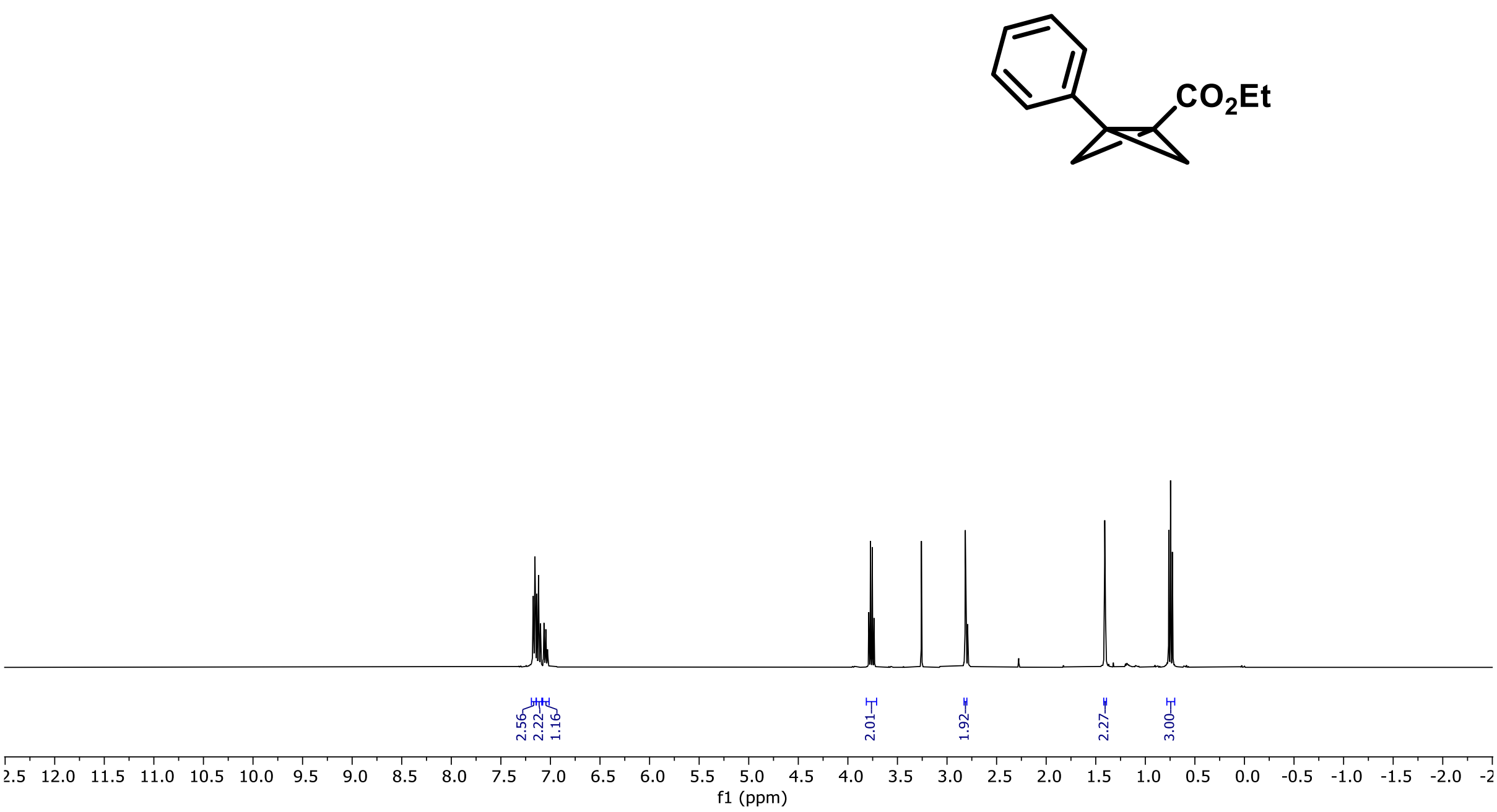

^{13}C {^{1}H} NMR (101 MHz, $CDCl_3$)

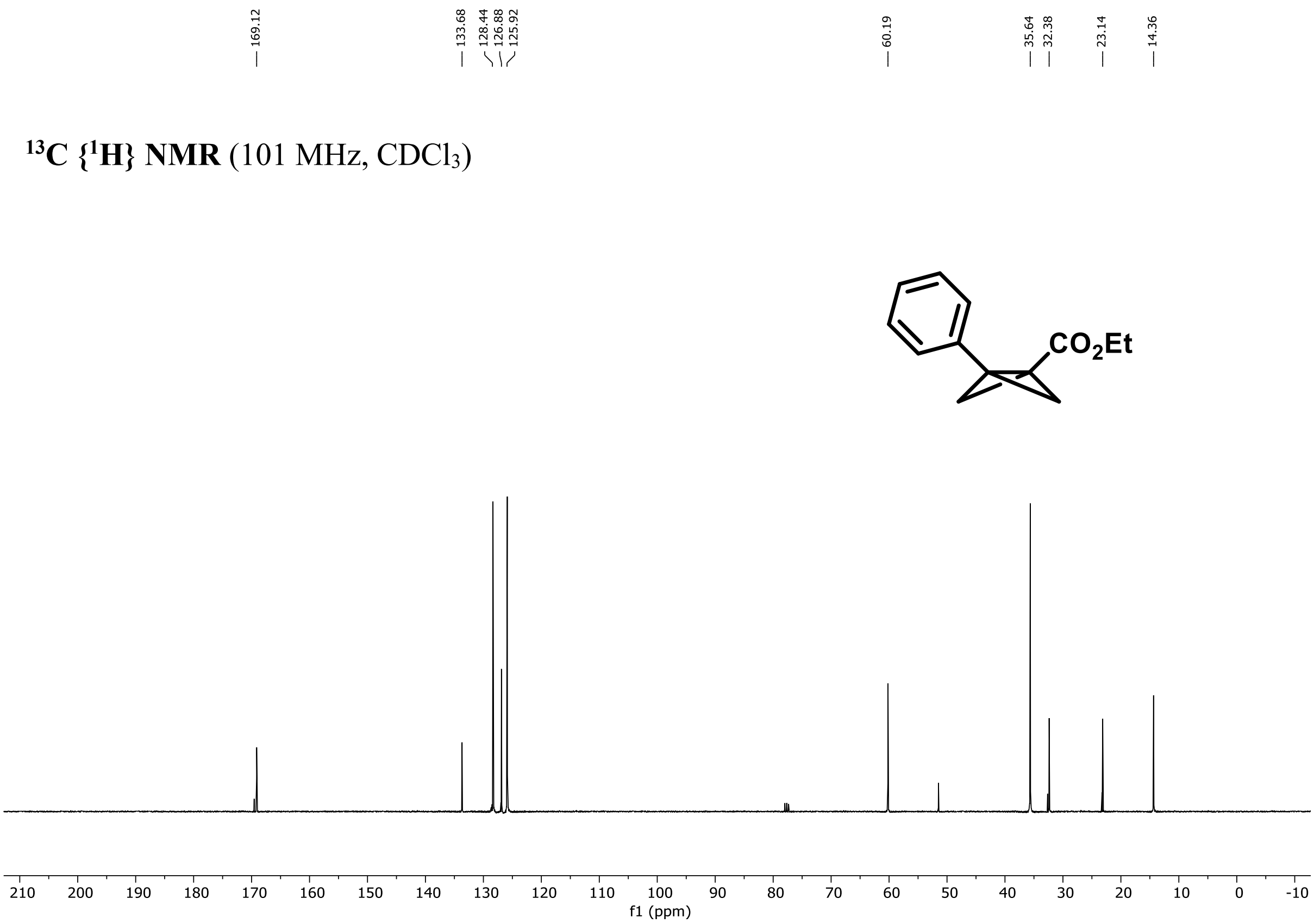

Isopropyl 3-phenylbicyclo[1.1.0]butane-1-carboxylate (1c):

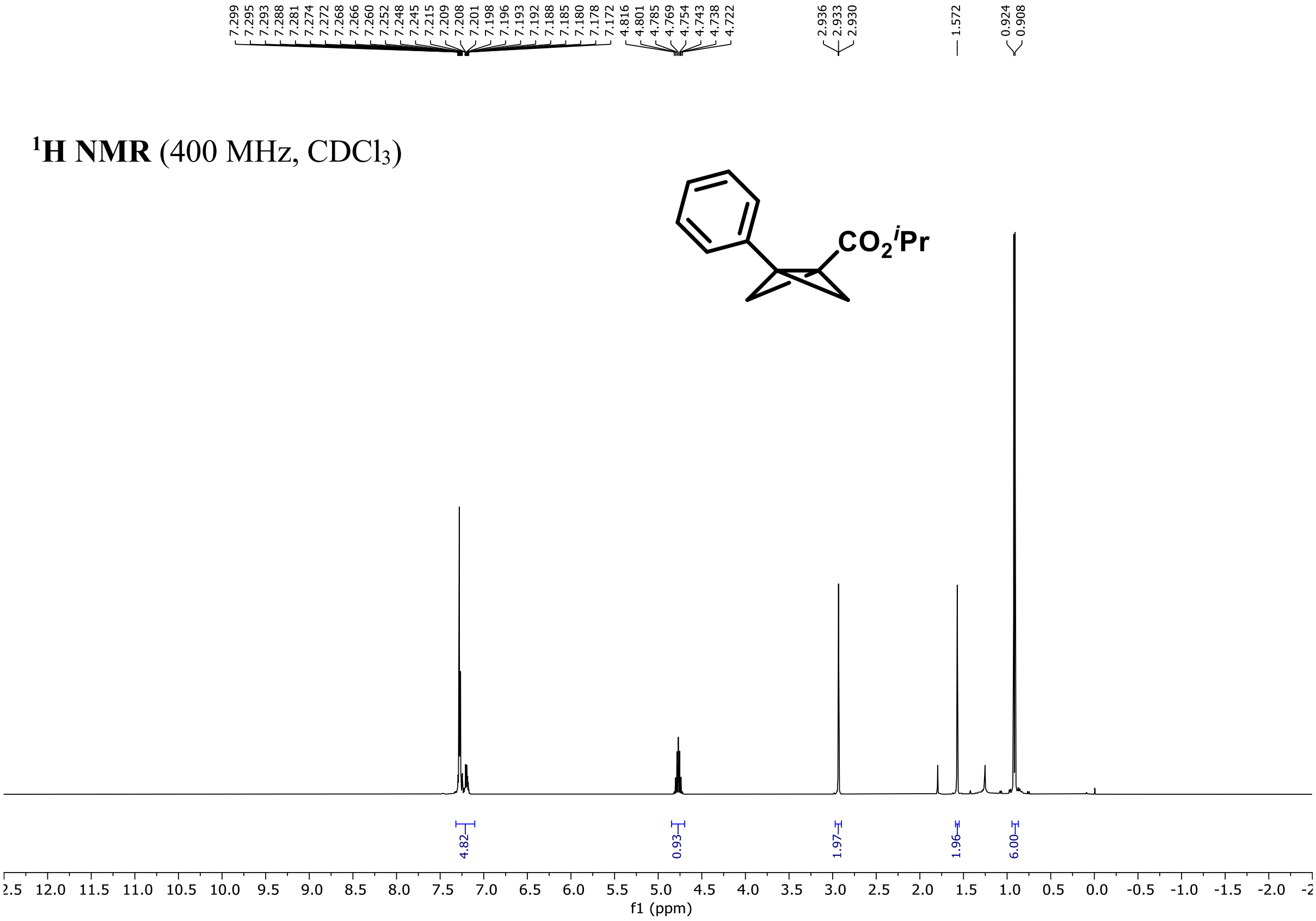

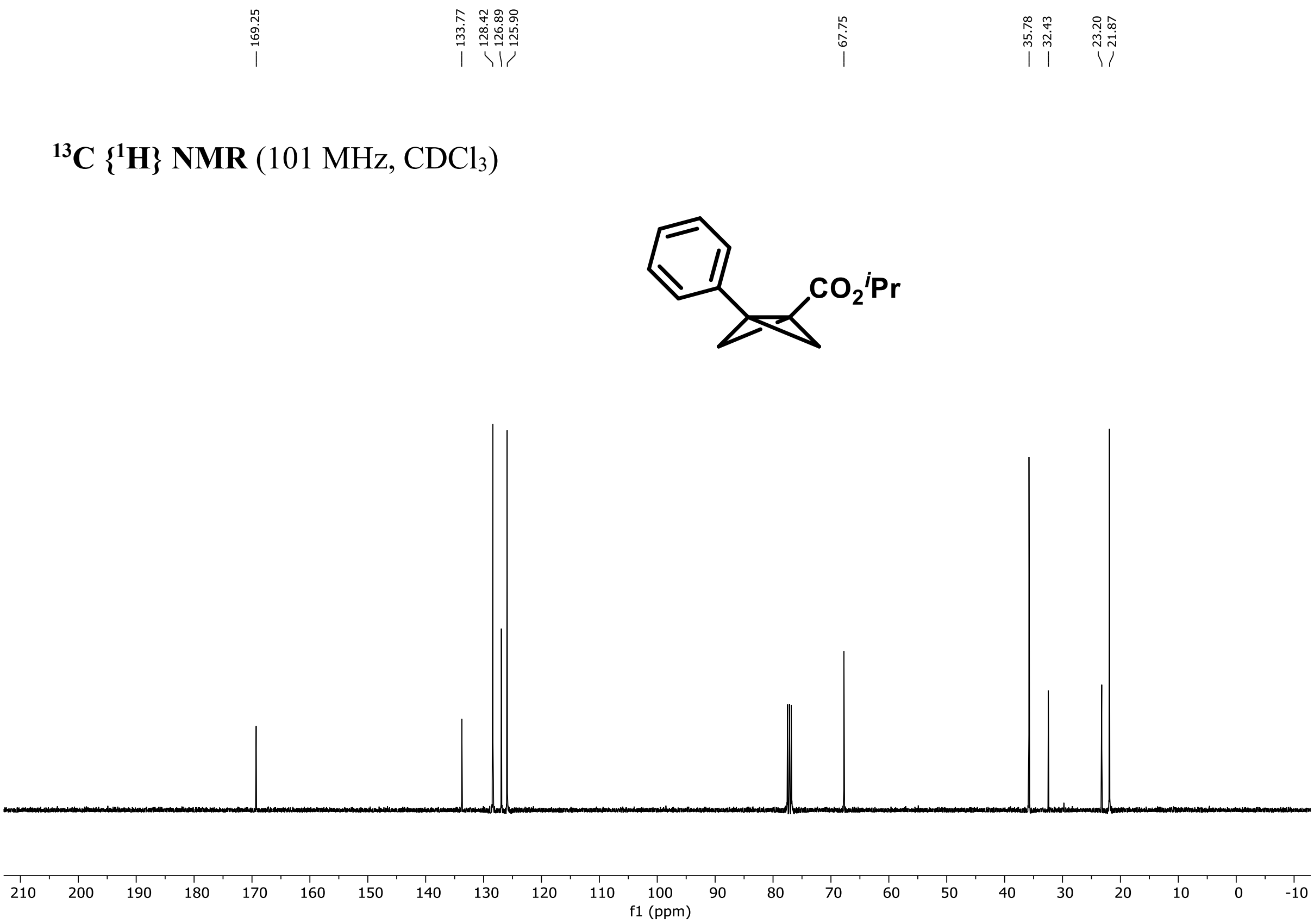

***tert*-butyl 3-phenylbicyclo[1.1.0]butane-1-carboxylate (1d):**

^{1}H NMR (400 MHz, $CDCl_3$)

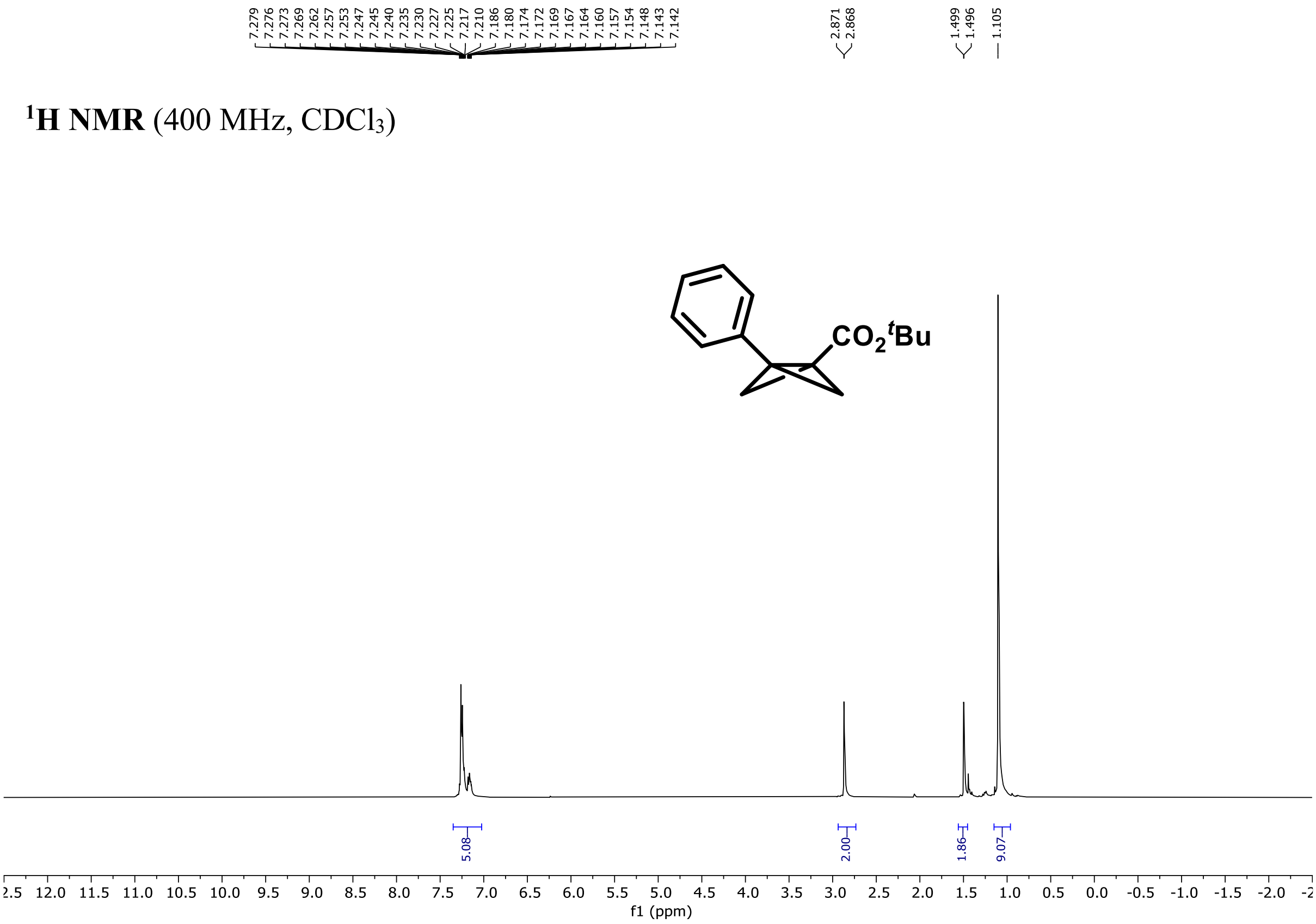

^{13}C {^{1}H} NMR (101 MHz, $CDCl_3$)

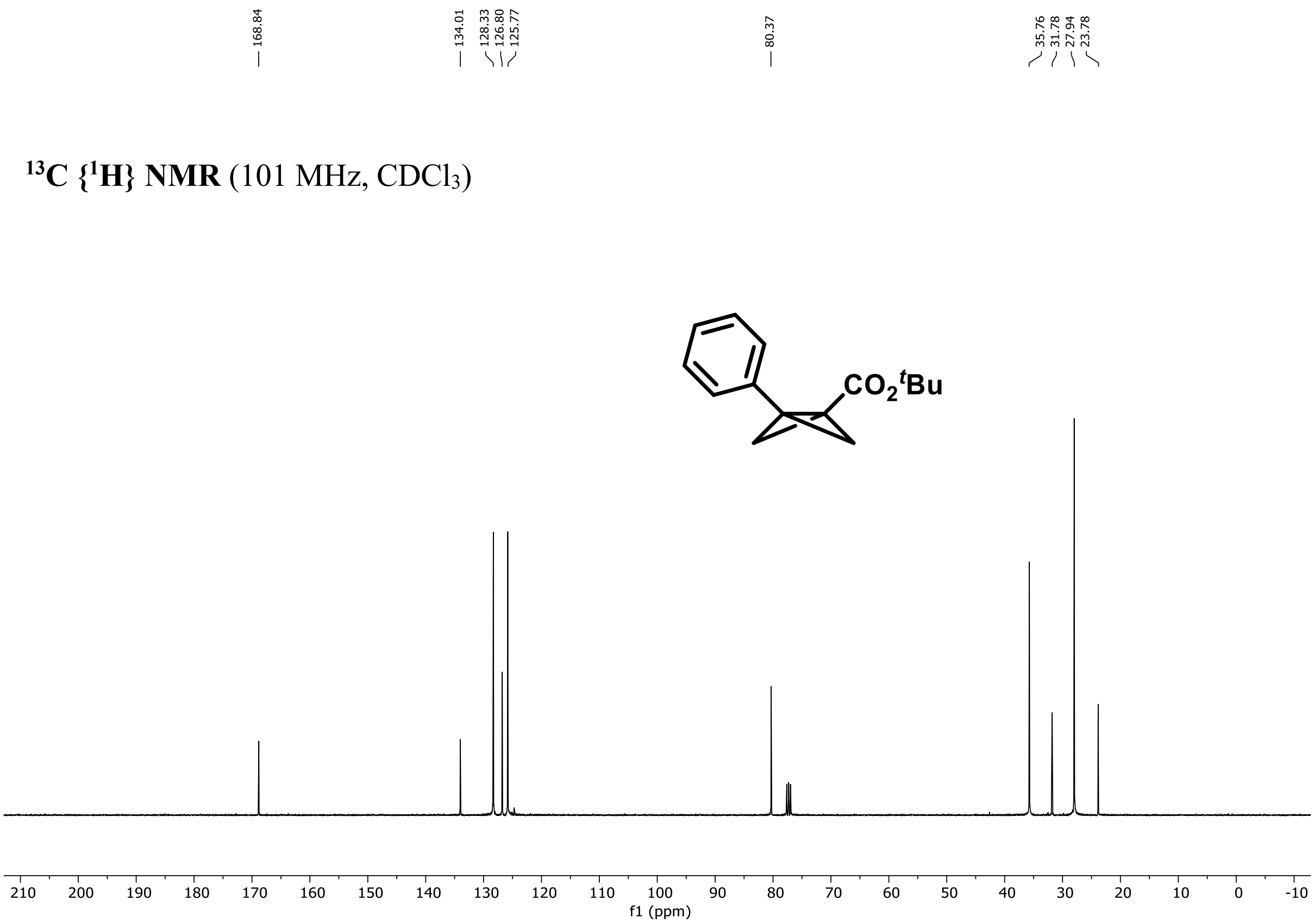

Phenyl 3-phenylbicyclo[1.1.0]butane-1-carboxylate (1e):

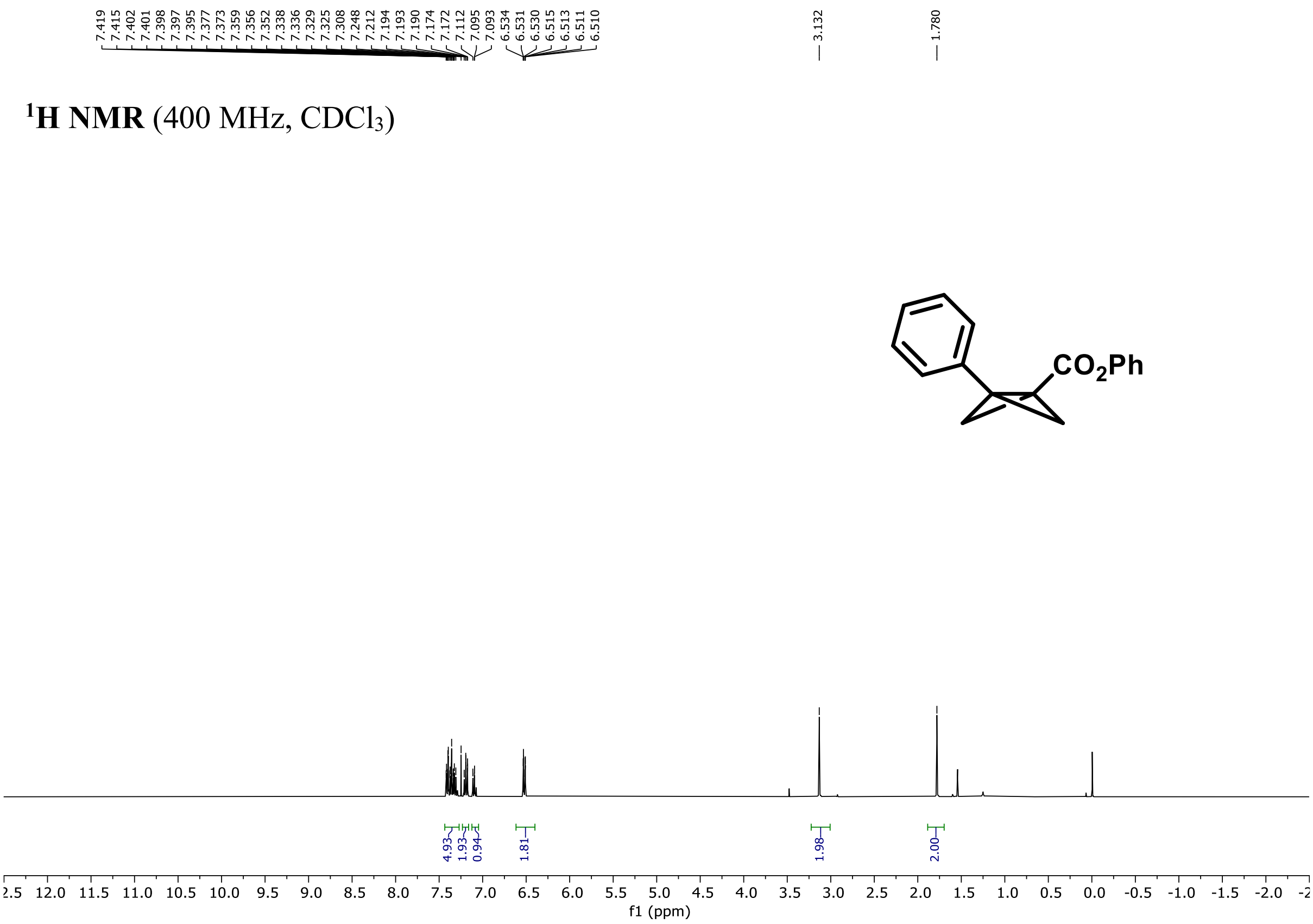

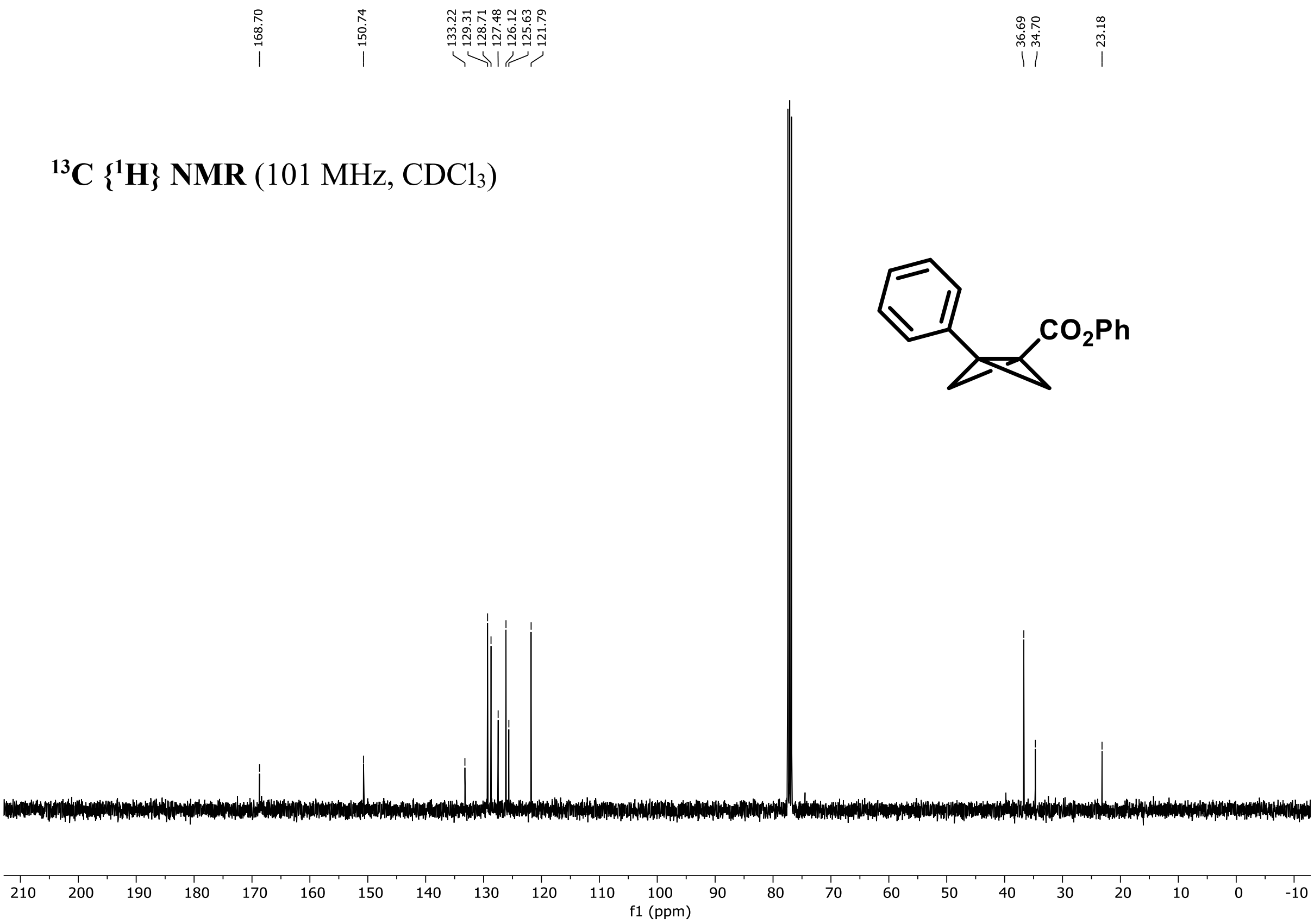

Benzyl 3-phenylbicyclo[1.1.0]butane-1-carboxylate (1f):

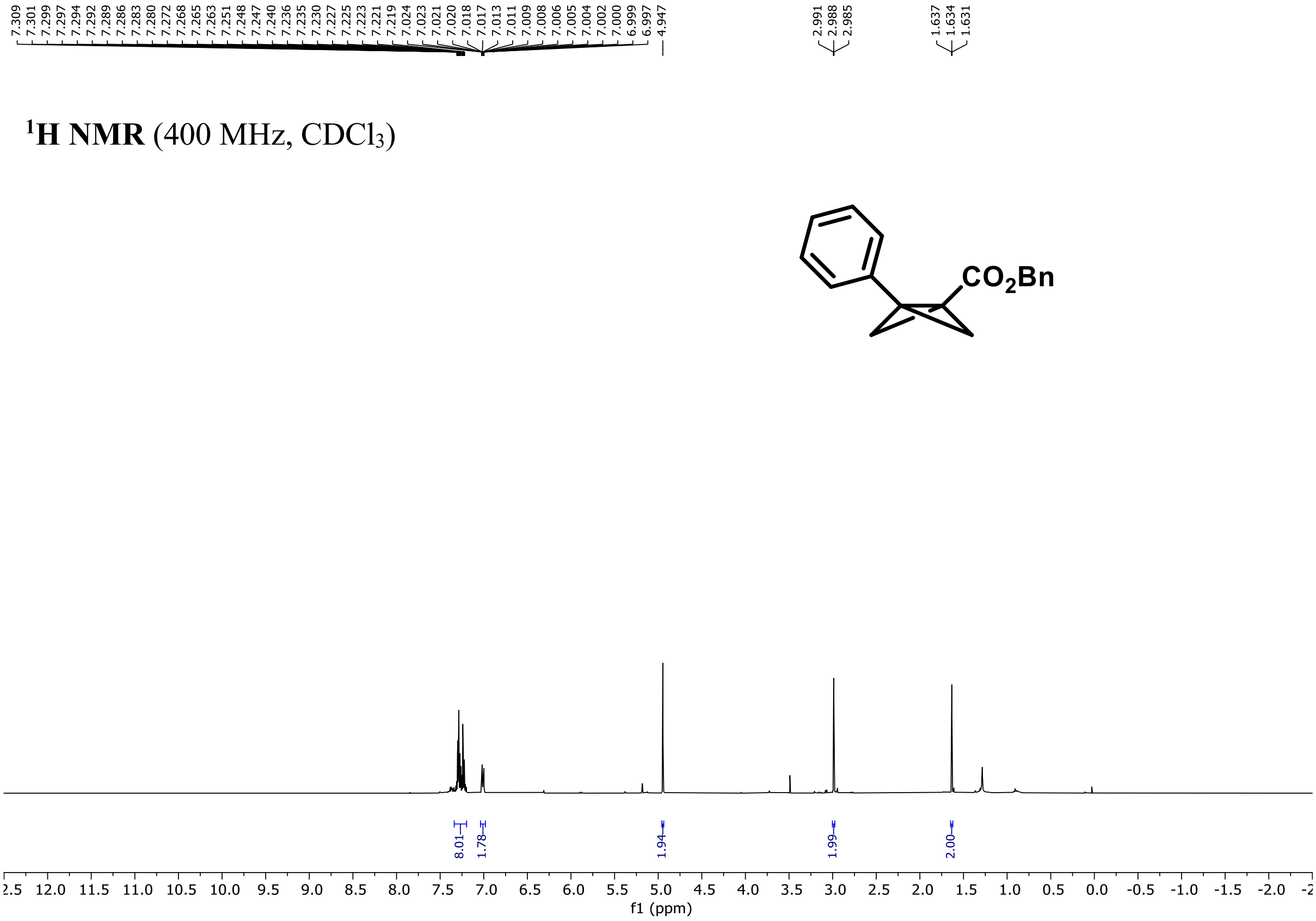

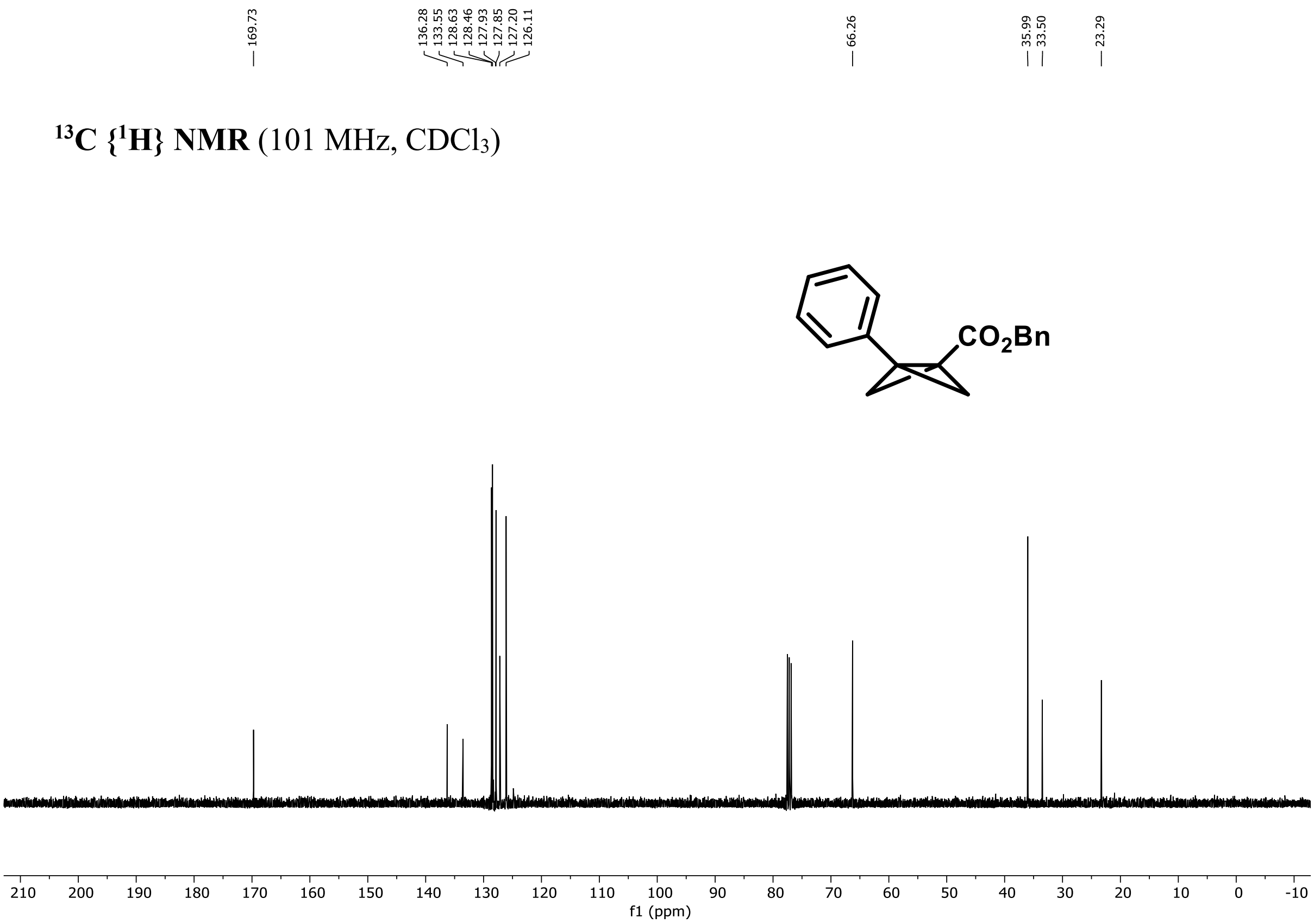

4-Methoxyphenyl 3-phenylbicyclo[1.1.0]butane-1-carboxylate (1g):

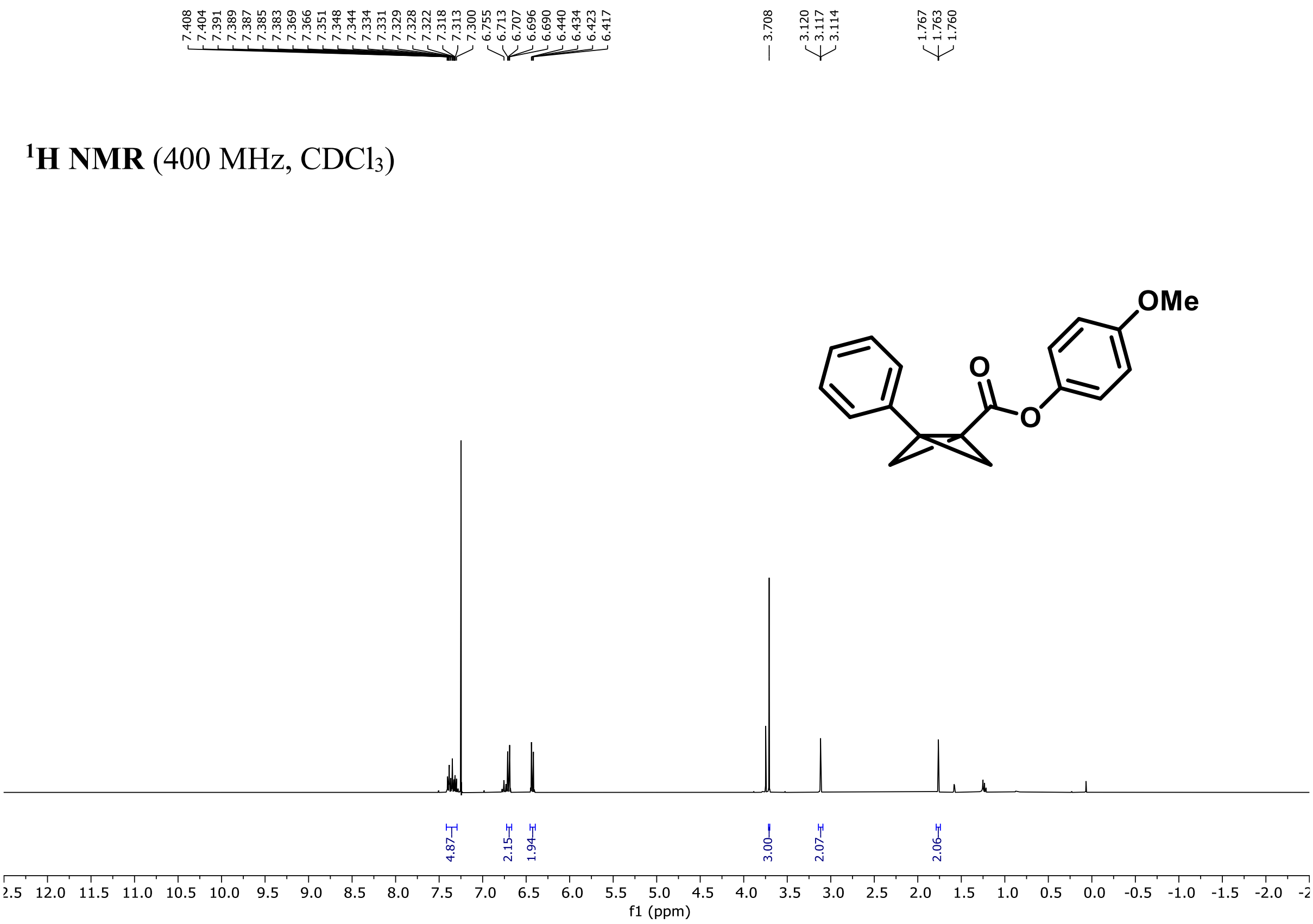

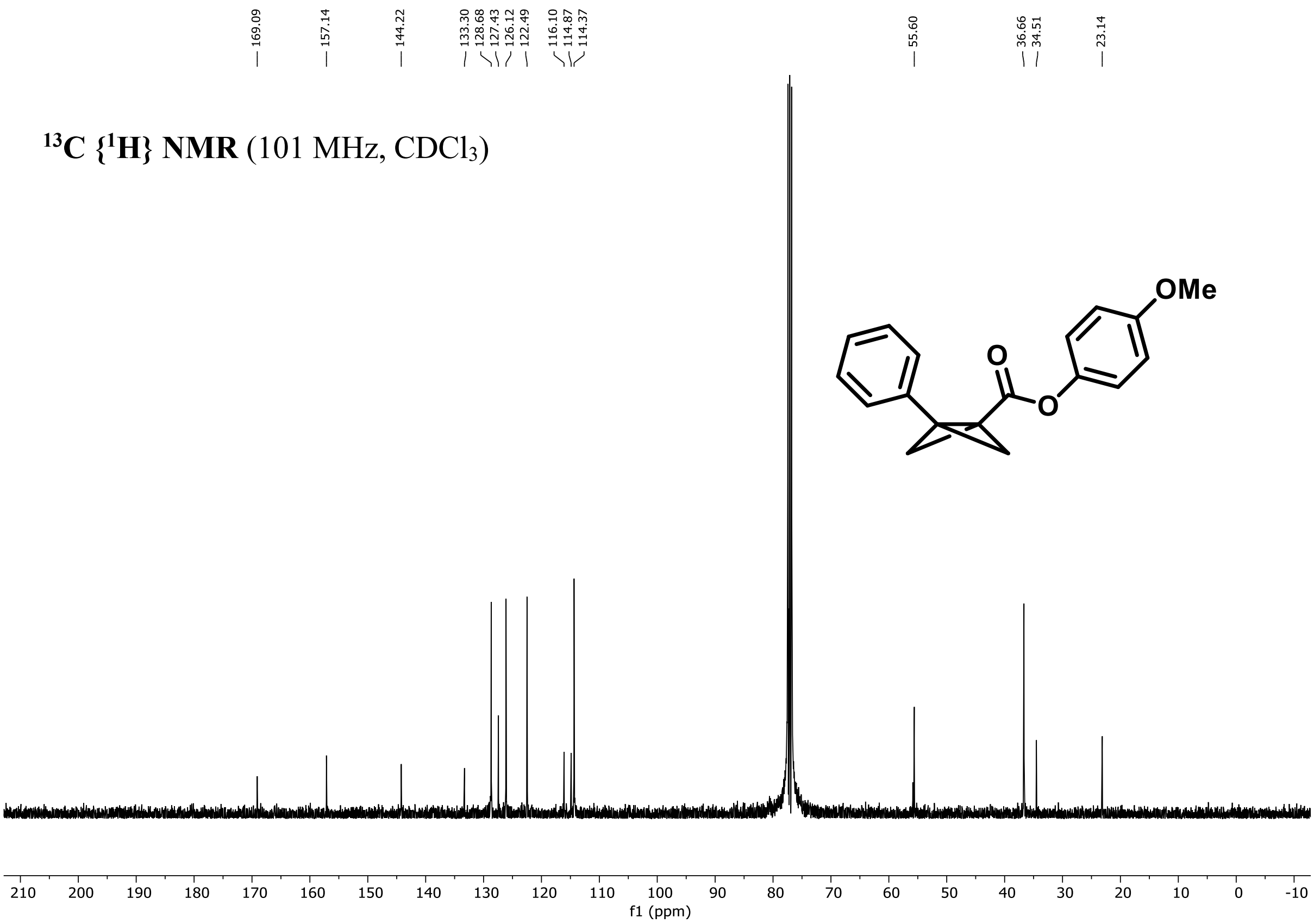

Methyl 3-(naphthalen-2-yl)bicyclo[1.1.0]butane-1-carboxylate (1h):

^{1}H NMR (400 MHz, $CDCl_3$)

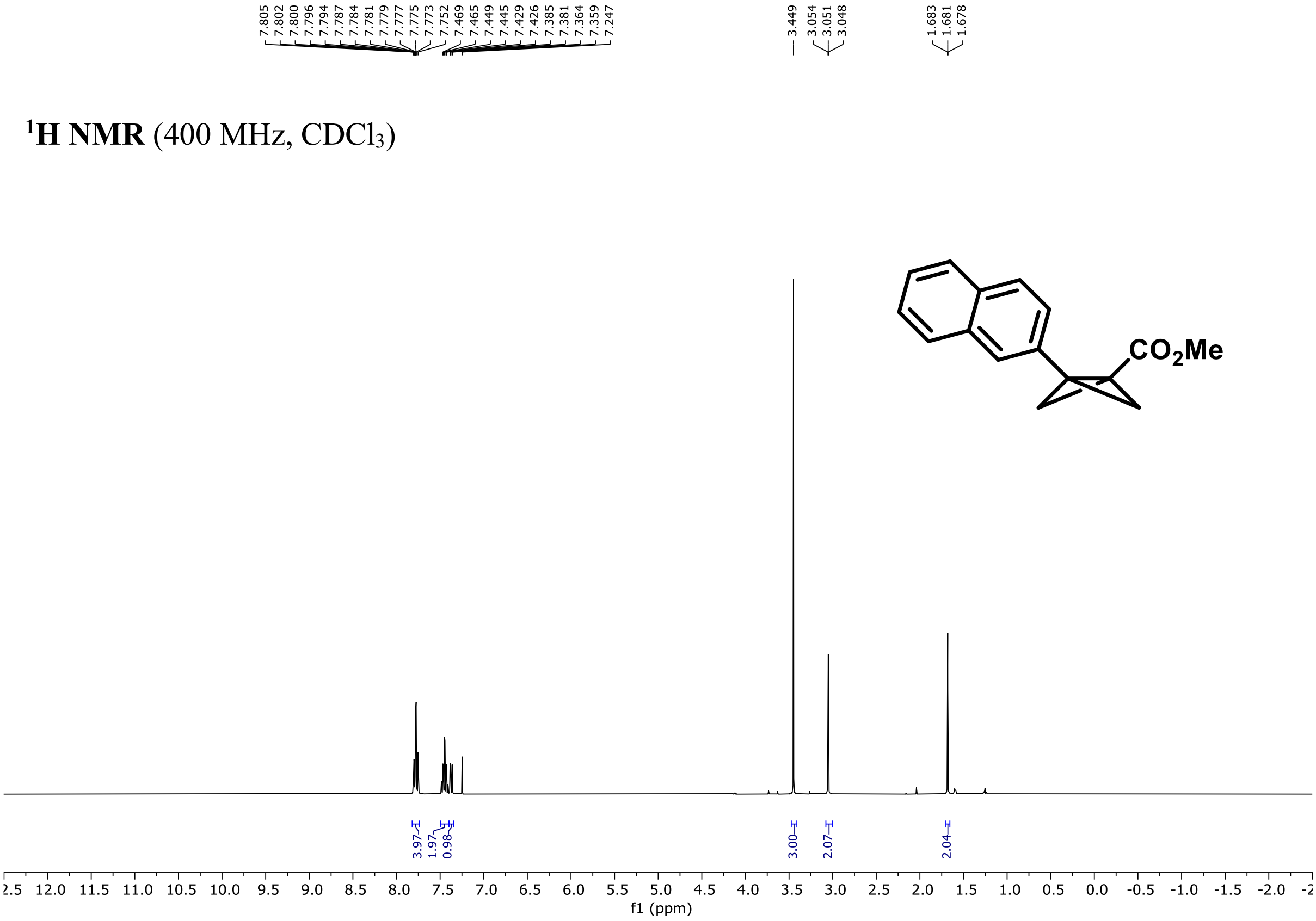

^{13}C {^{1}H} NMR (101 MHz, $CDCl_3$)

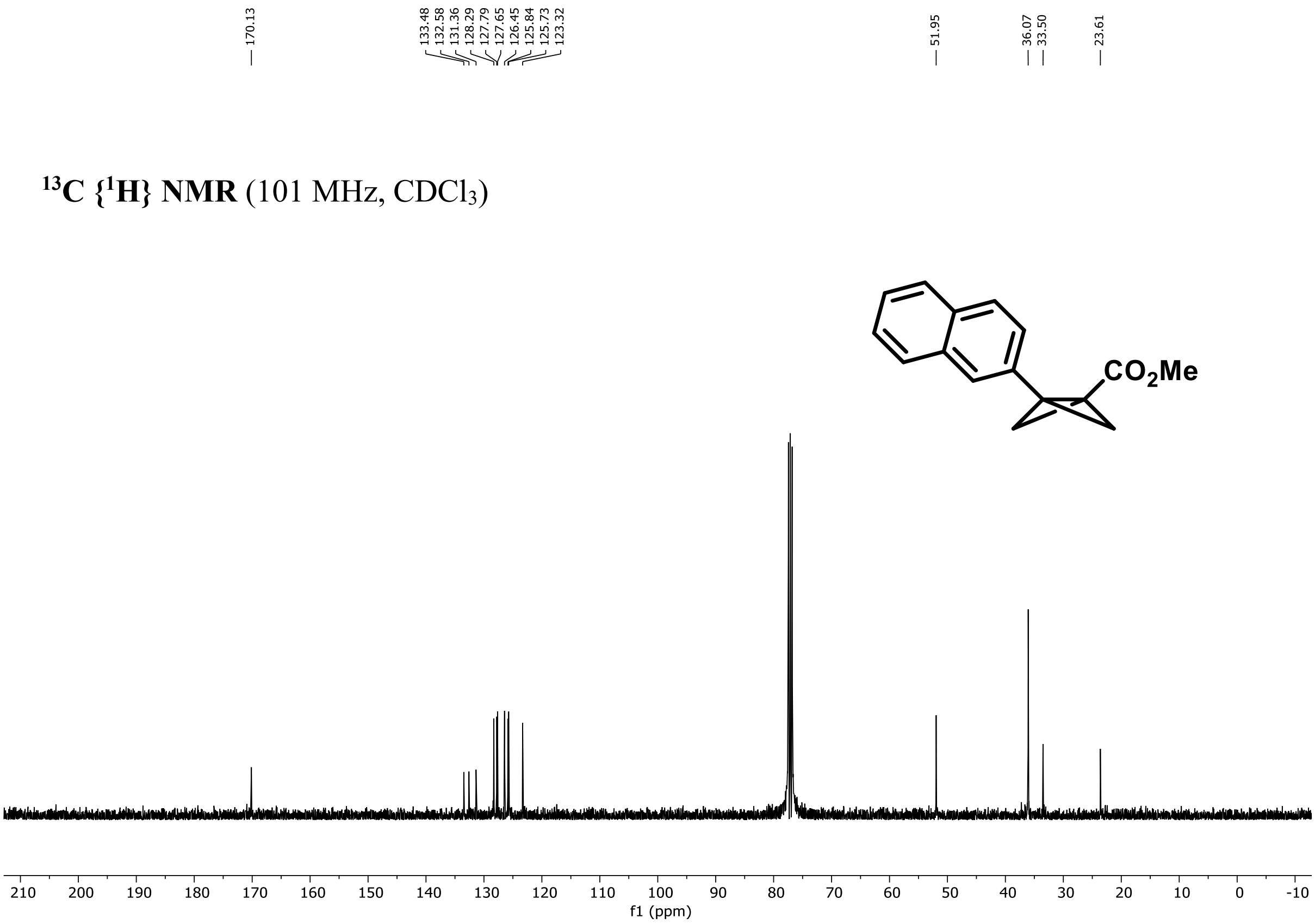

Methyl 3-(4-(*tert*-butyl)phenyl)bicyclo[1.1.0]butane-1-carboxylate (1i):

^{1}H NMR (400 MHz, $CDCl_3$)

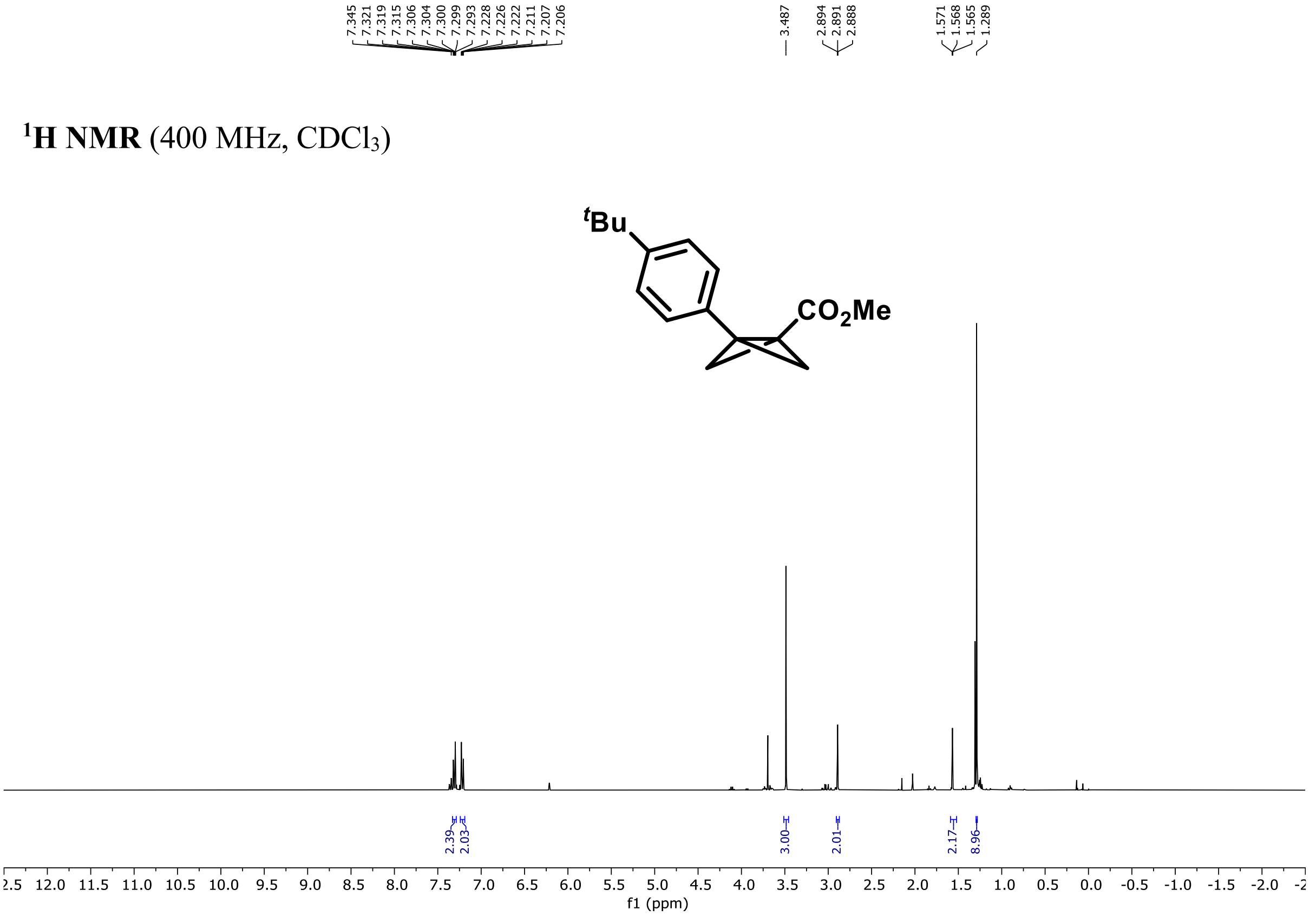

^{13}C {^{1}H} NMR (101 MHz, $CDCl_3$)

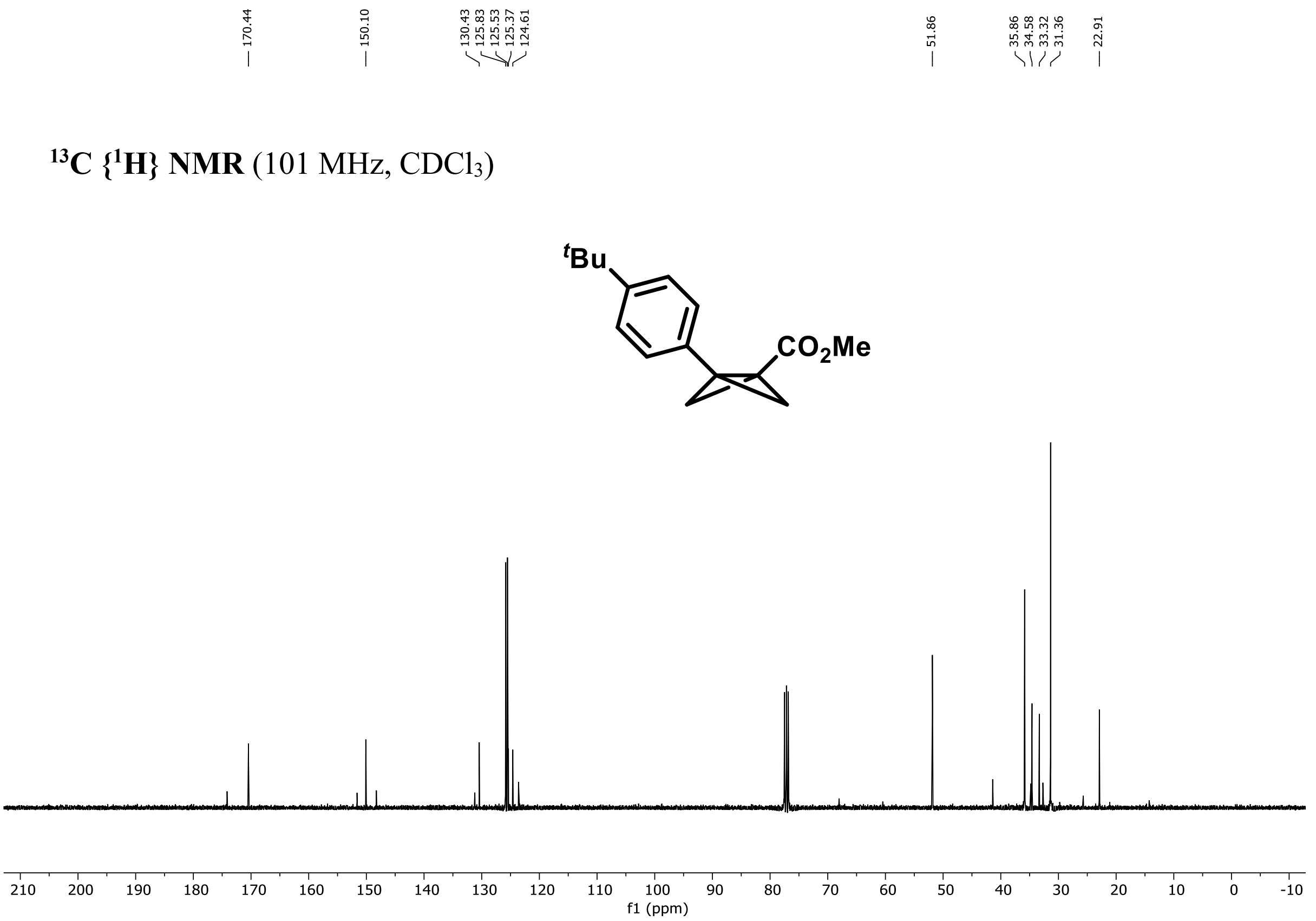

Methyl 3-([1,1'-biphenyl]-4-yl)bicyclo[1.1.0]butane-1-carboxylate (1j):

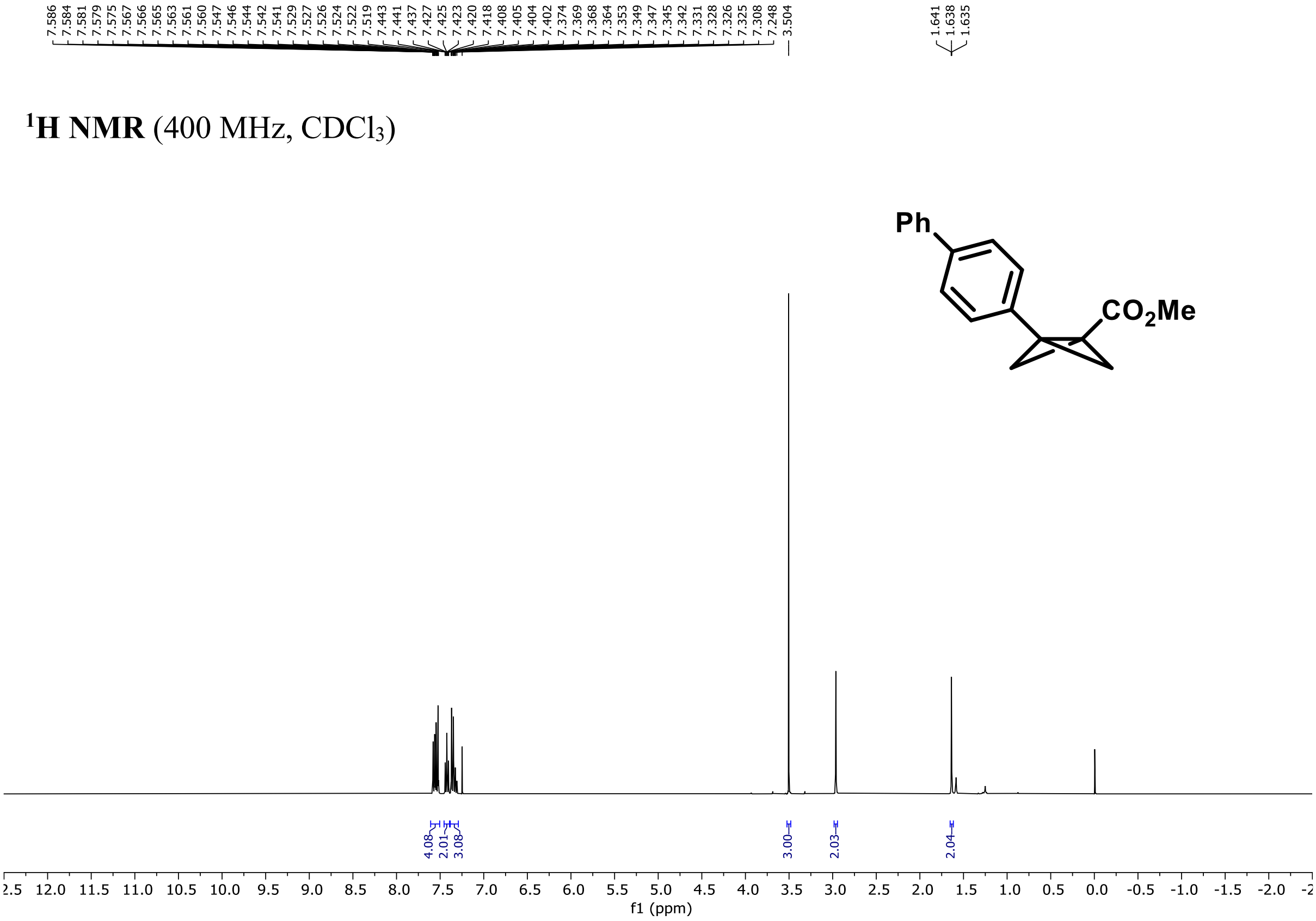

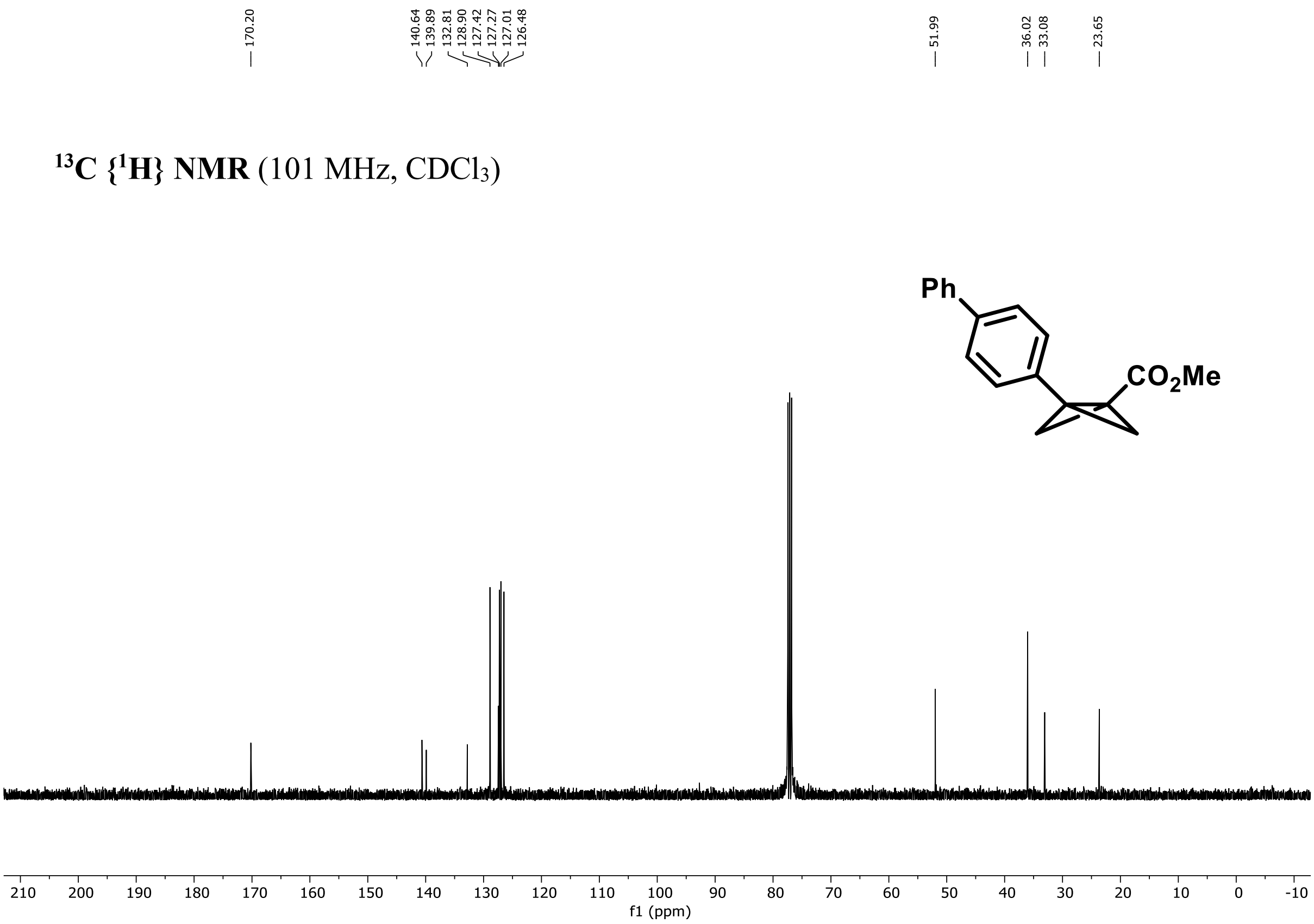

Methyl 3-(3-methoxyphenyl)bicyclo[1.1.0]butane-1-carboxylate (1k):

^{1}H NMR (400 MHz, $CDCl_3$)

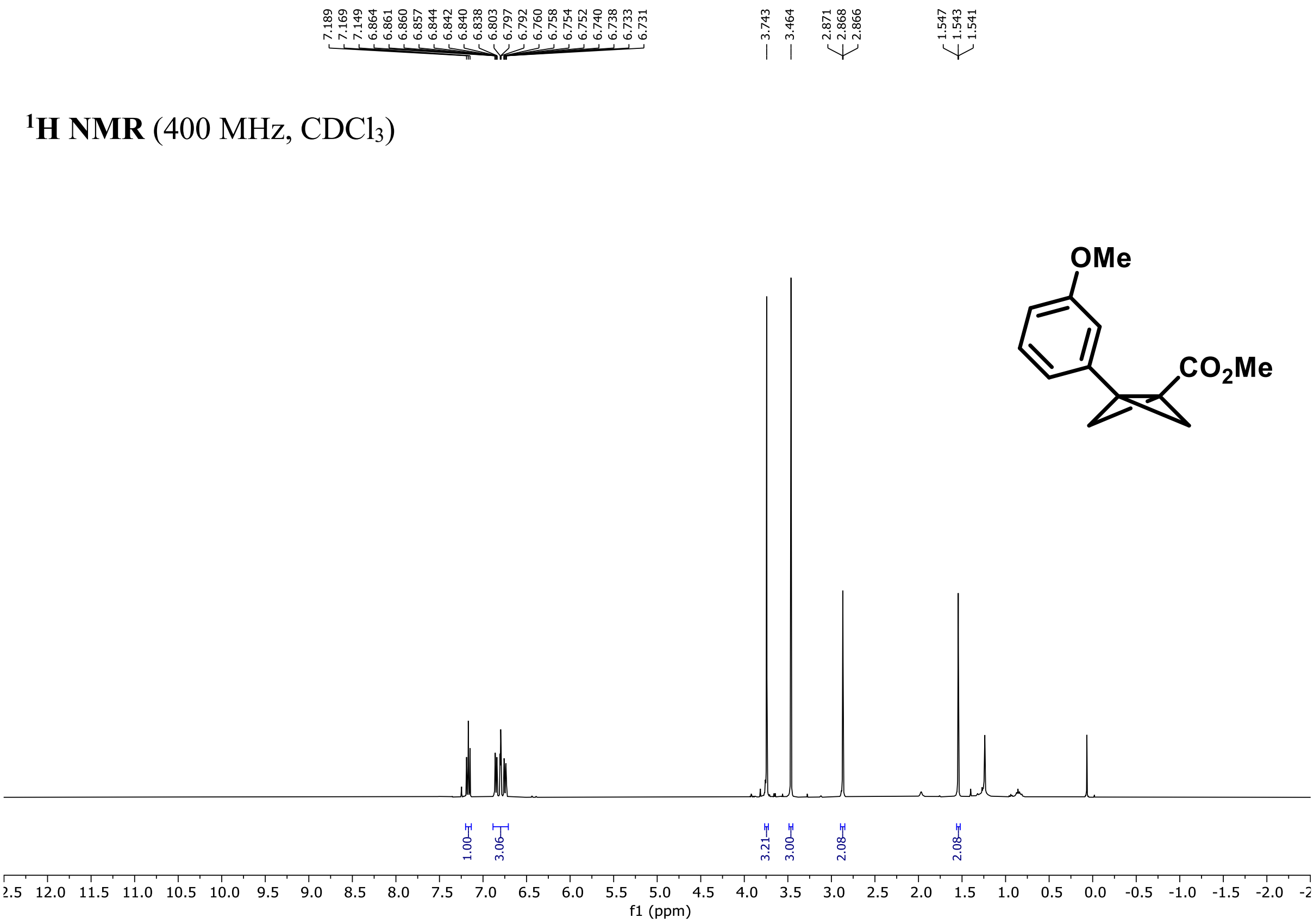

^{13}C {^{1}H} NMR (101 MHz, $CDCl_3$)

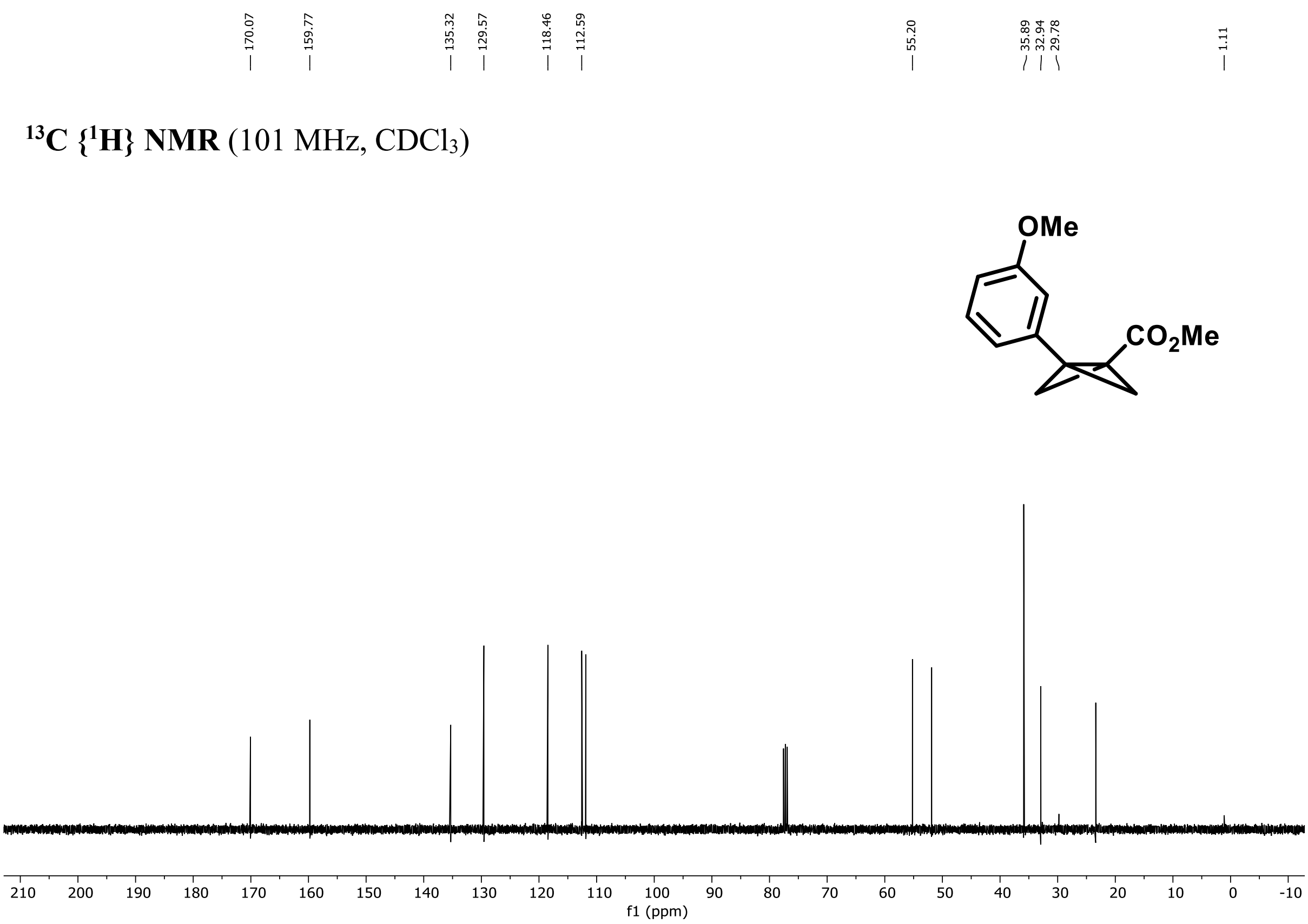

Methyl 3-(4-fluorophenyl)bicyclo[1.1.0]butane-1-carboxylate (1l):

^{1}H NMR (400 MHz, $CDCl_3$)

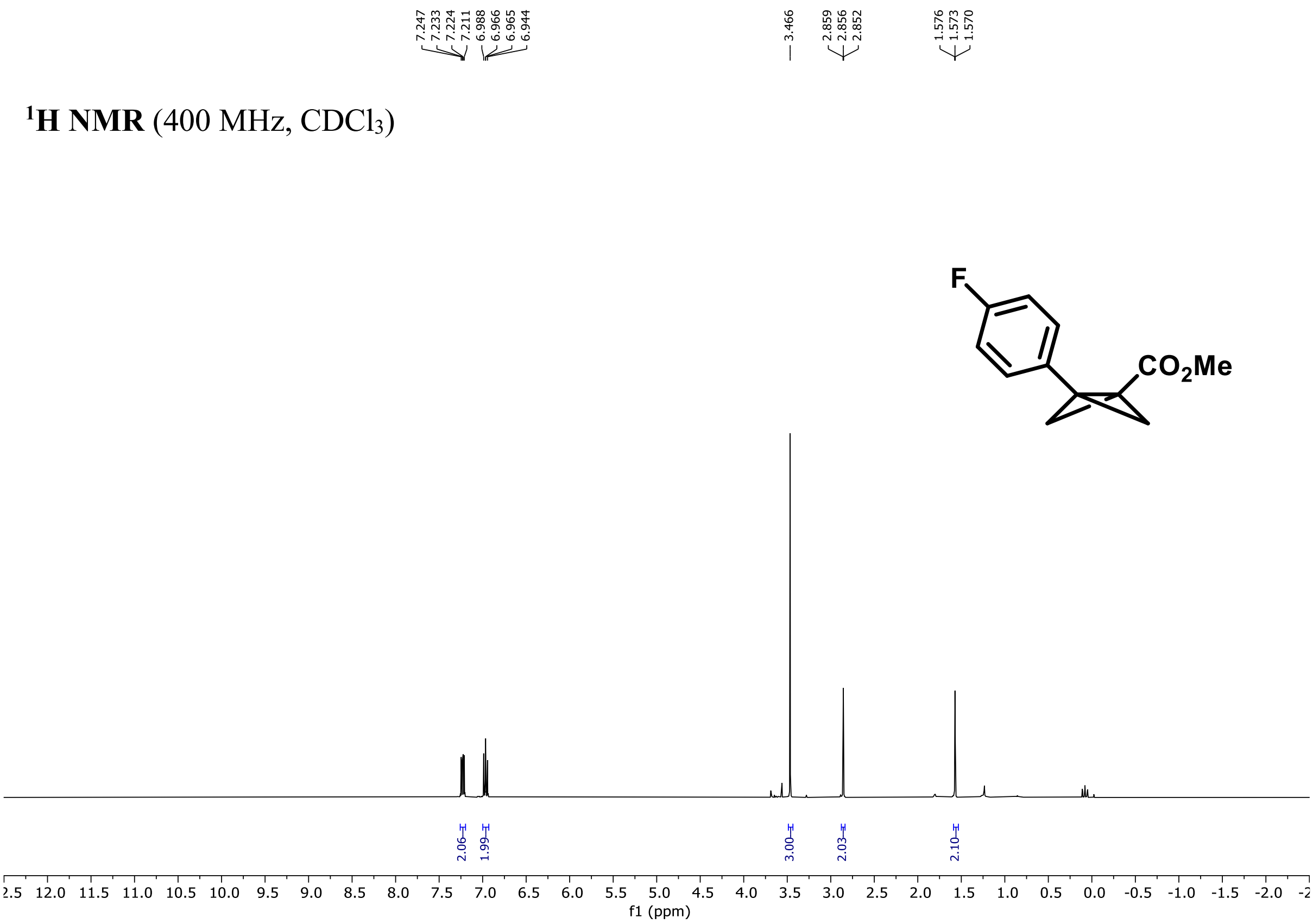

^{13}C {^{1}H} NMR (101 MHz, $CDCl_3$)

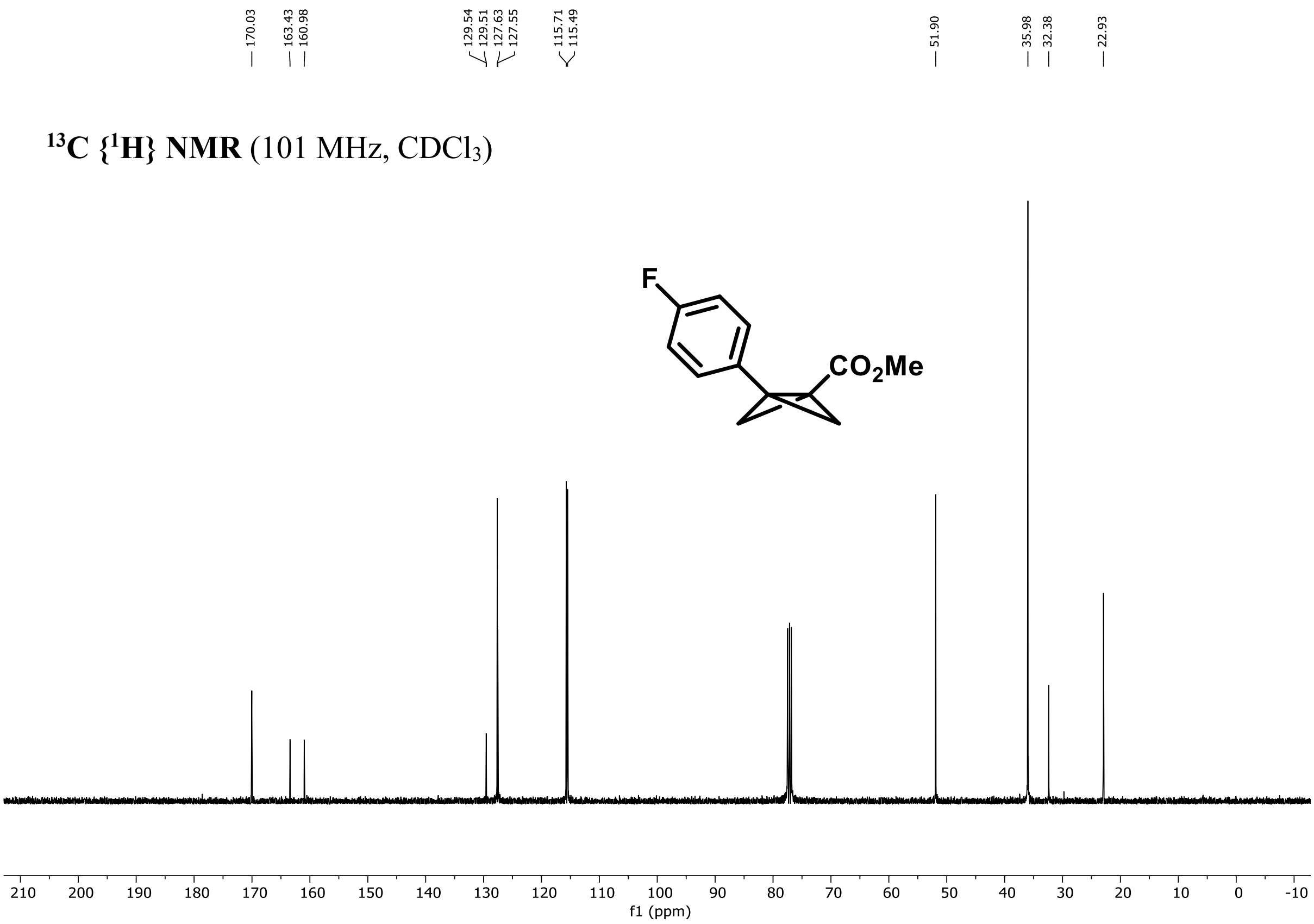

^{19}F NMR (376 MHz, $CDCl_3$)

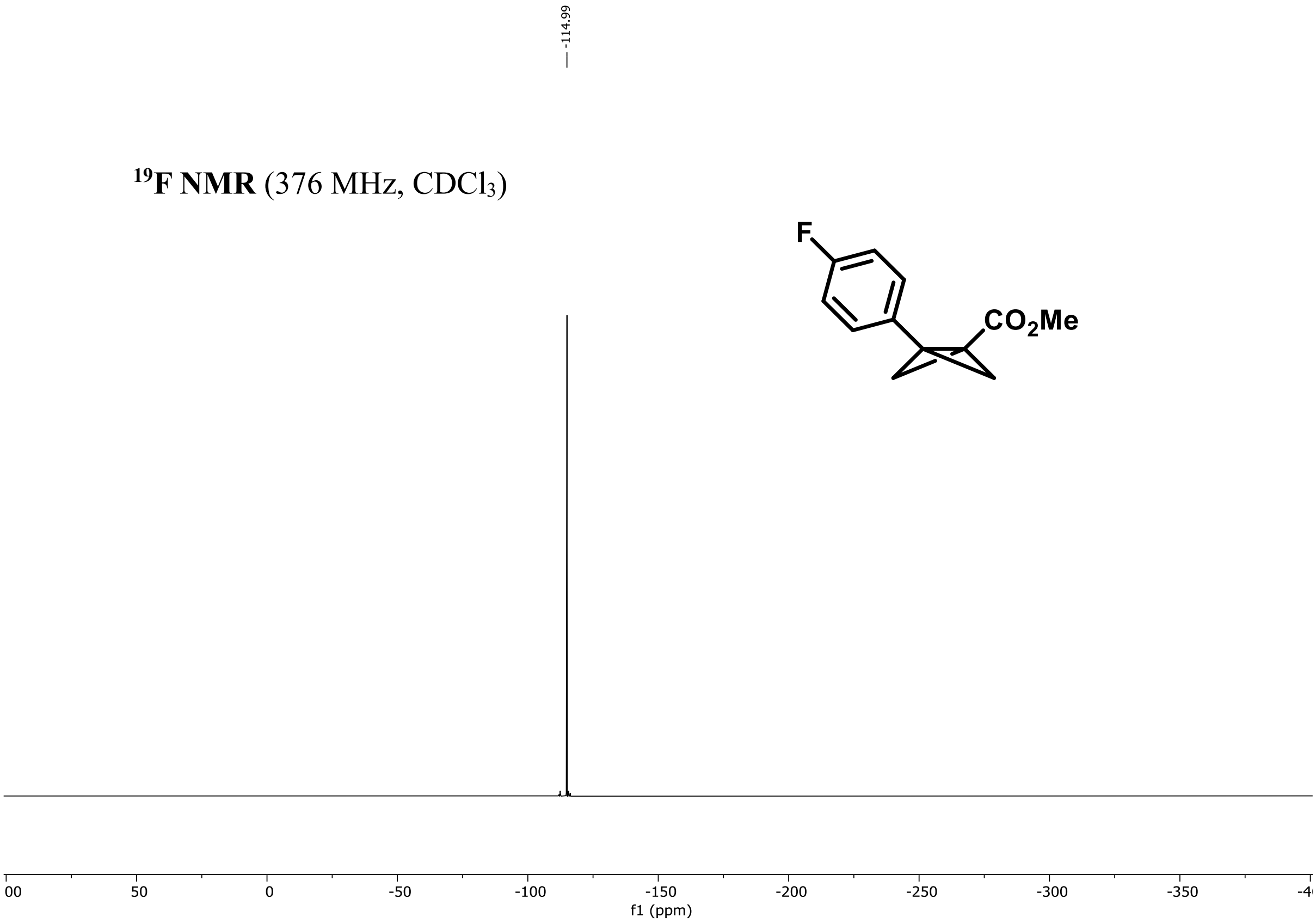

Methyl 3-(4-chlorophenyl)bicyclo[1.1.0]butane-1-carboxylate (1m):

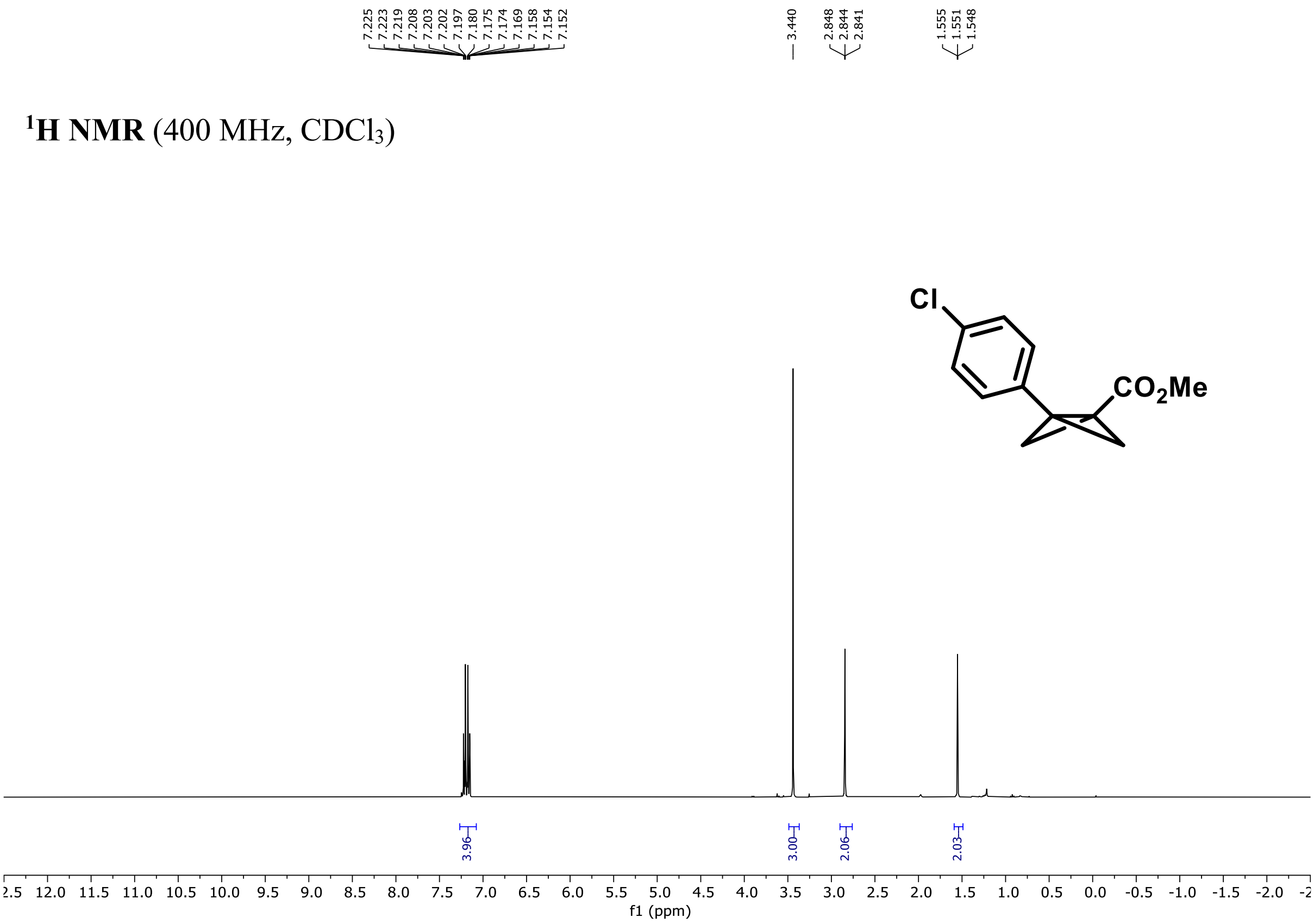

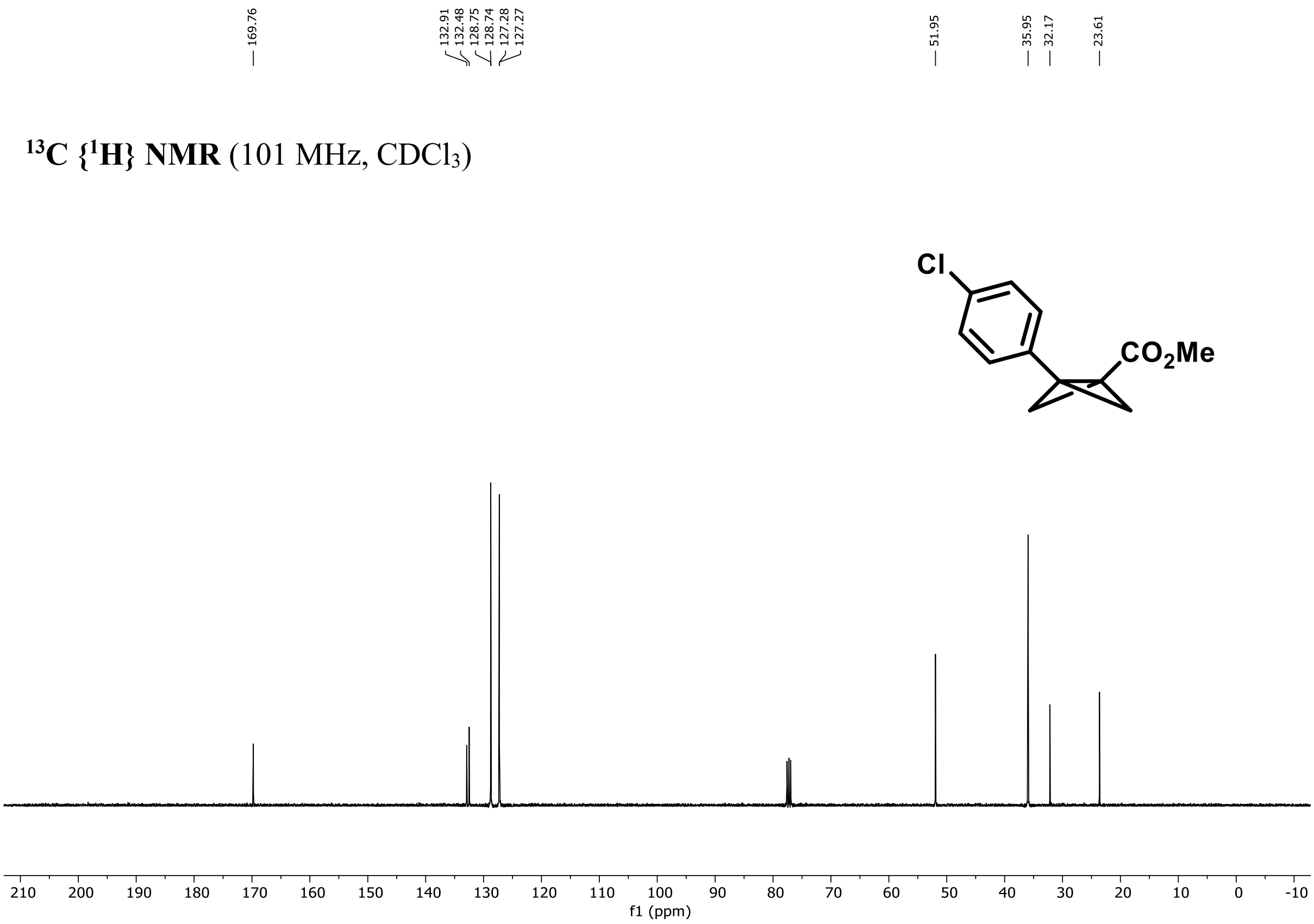

Methyl 3-(4-(trifluoromethyl)phenyl)bicyclo[1.1.0]butane-1-carboxylate (1n):

[1]**H NMR** (400 MHz, $CDCl_3$)

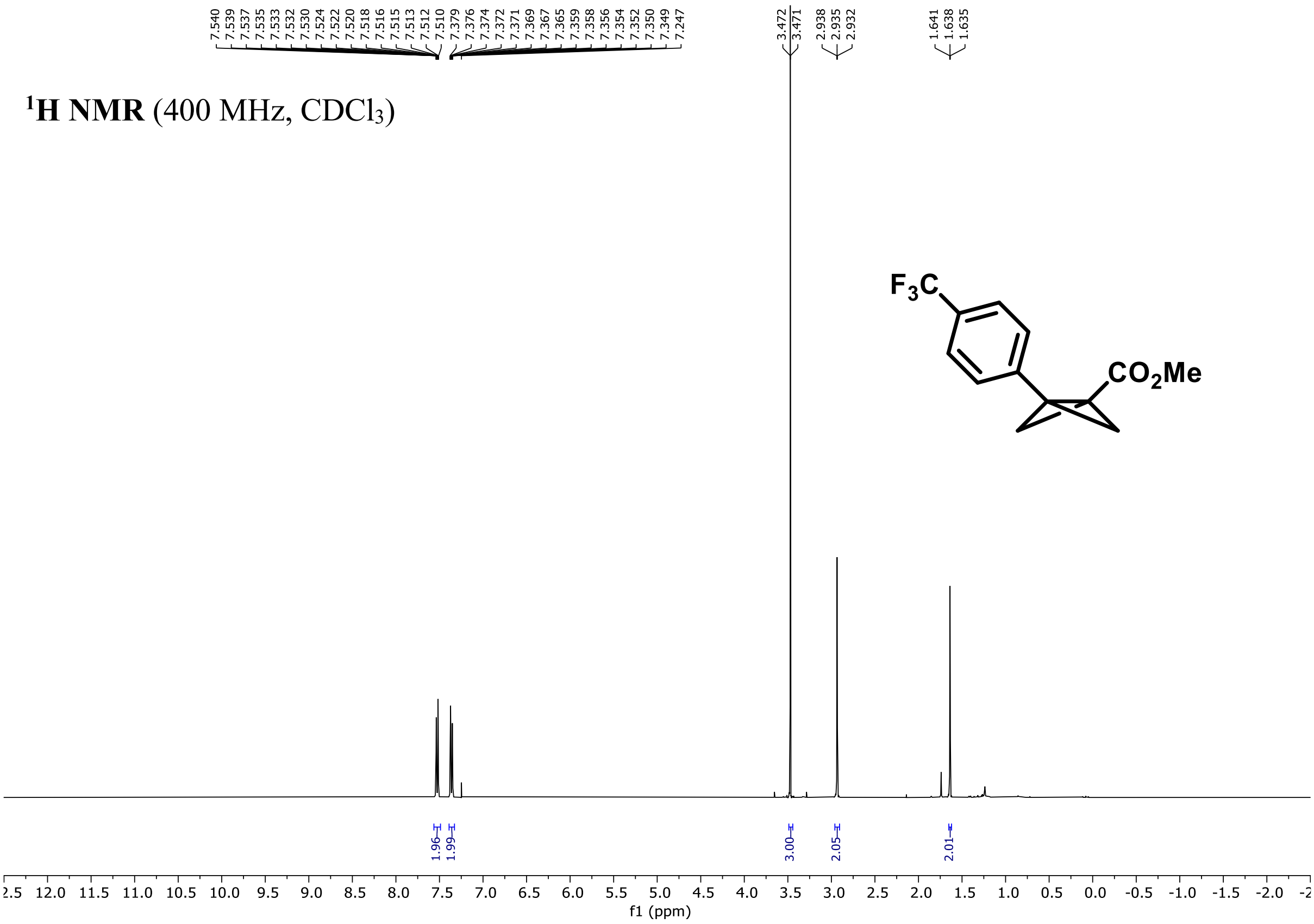

[13]**C {**[1]**H} NMR** (101 MHz, $CDCl_3$)

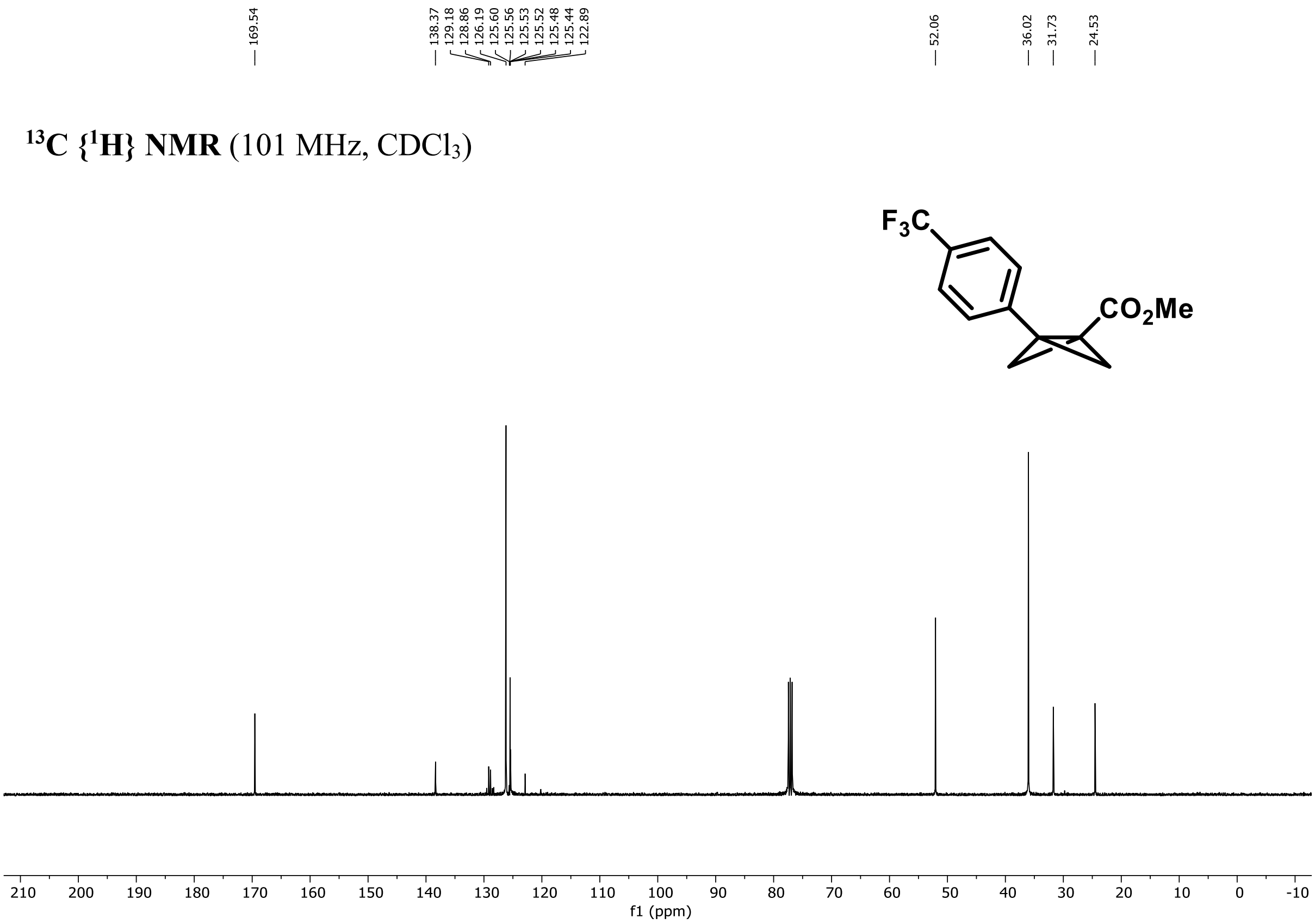

^{19}F NMR (376 MHz, $CDCl_3$)

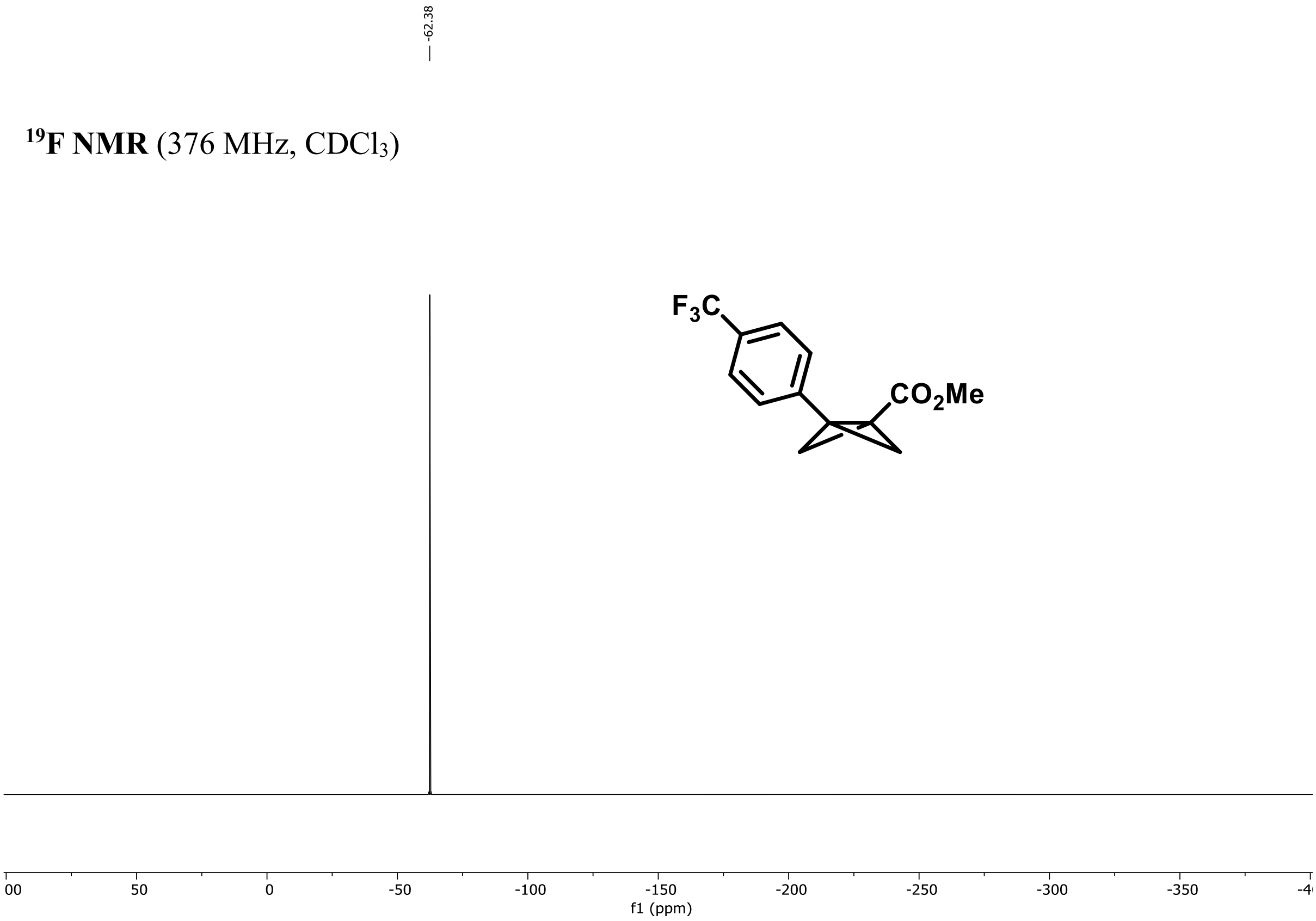

(*Z*)-3,7-dimethylocta-2,6-dien-1-yl 3-phenylbicyclo[1.1.0]butane-1-carboxylate (1o):

^{1}H NMR (400 MHz, $CDCl_3$)

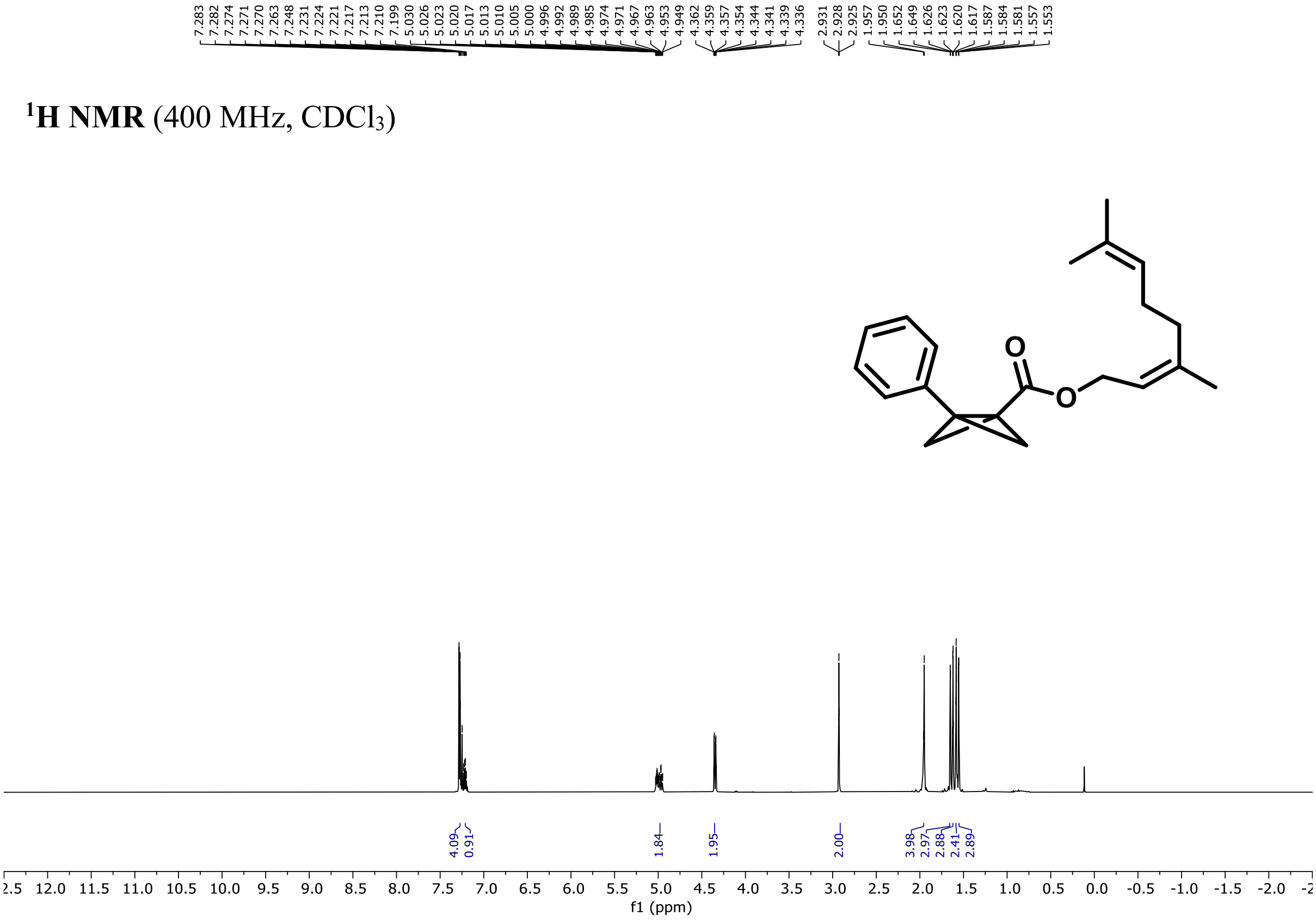

^{13}C {^{1}H} NMR (101 MHz, $CDCl_3$)

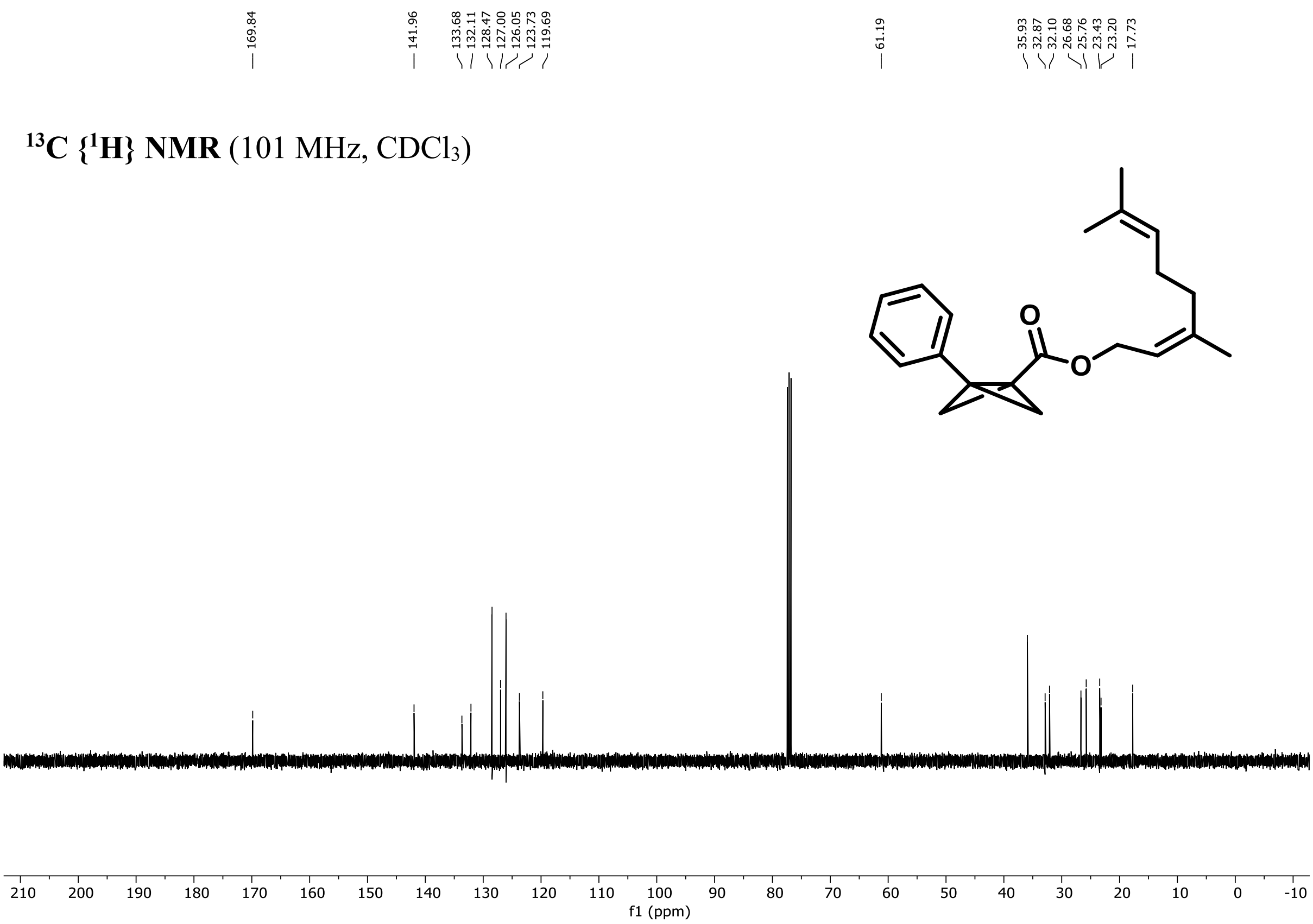

Benzo[d][1,3]dioxol-5-yl-3-phenylbicyclo[1.1.0]butane-1-carboxylate (1p):

[1]**H NMR** (400 MHz, $CDCl_3$)

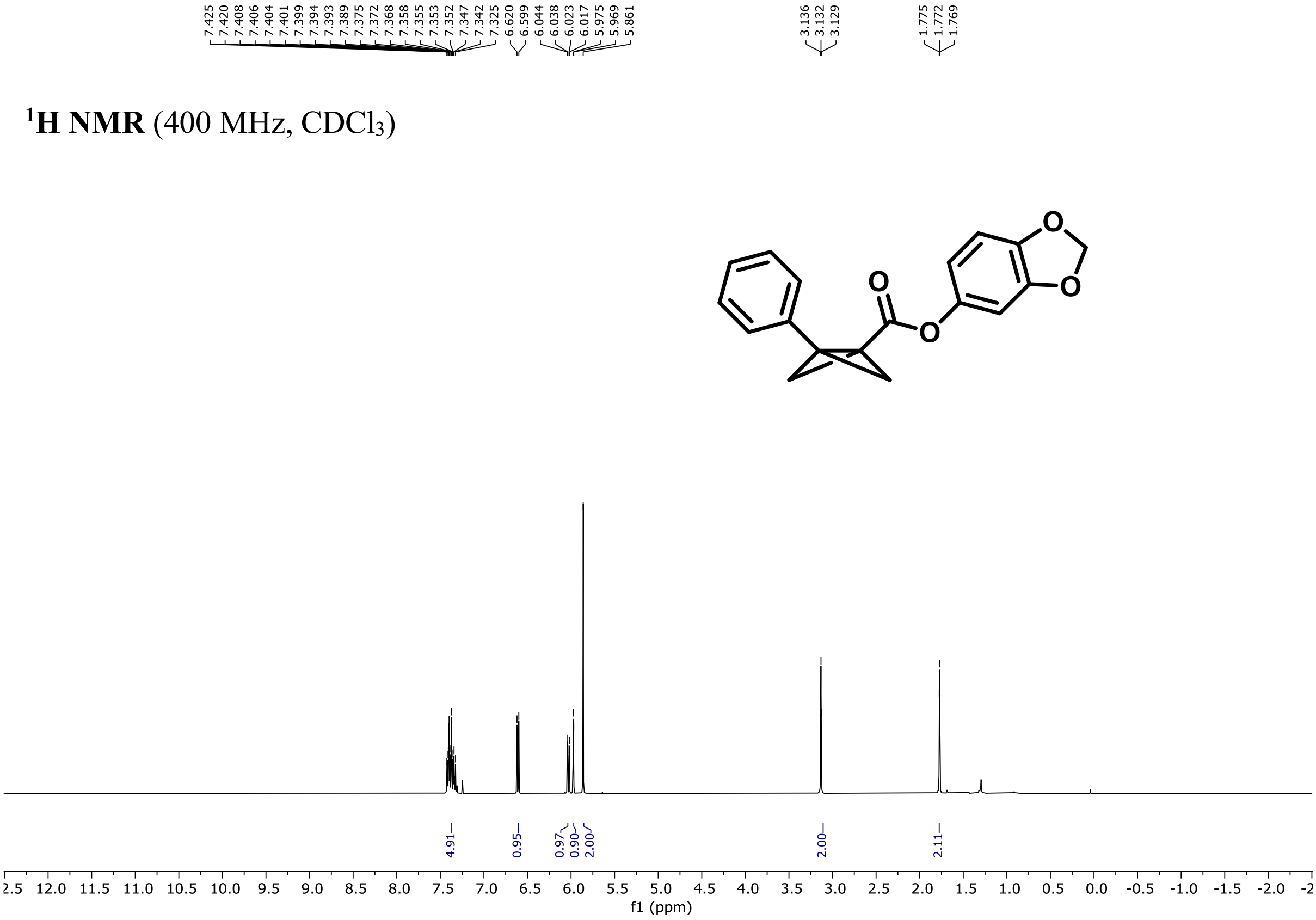

[13]**C {^{1}H} NMR** (101 MHz, $CDCl_3$)

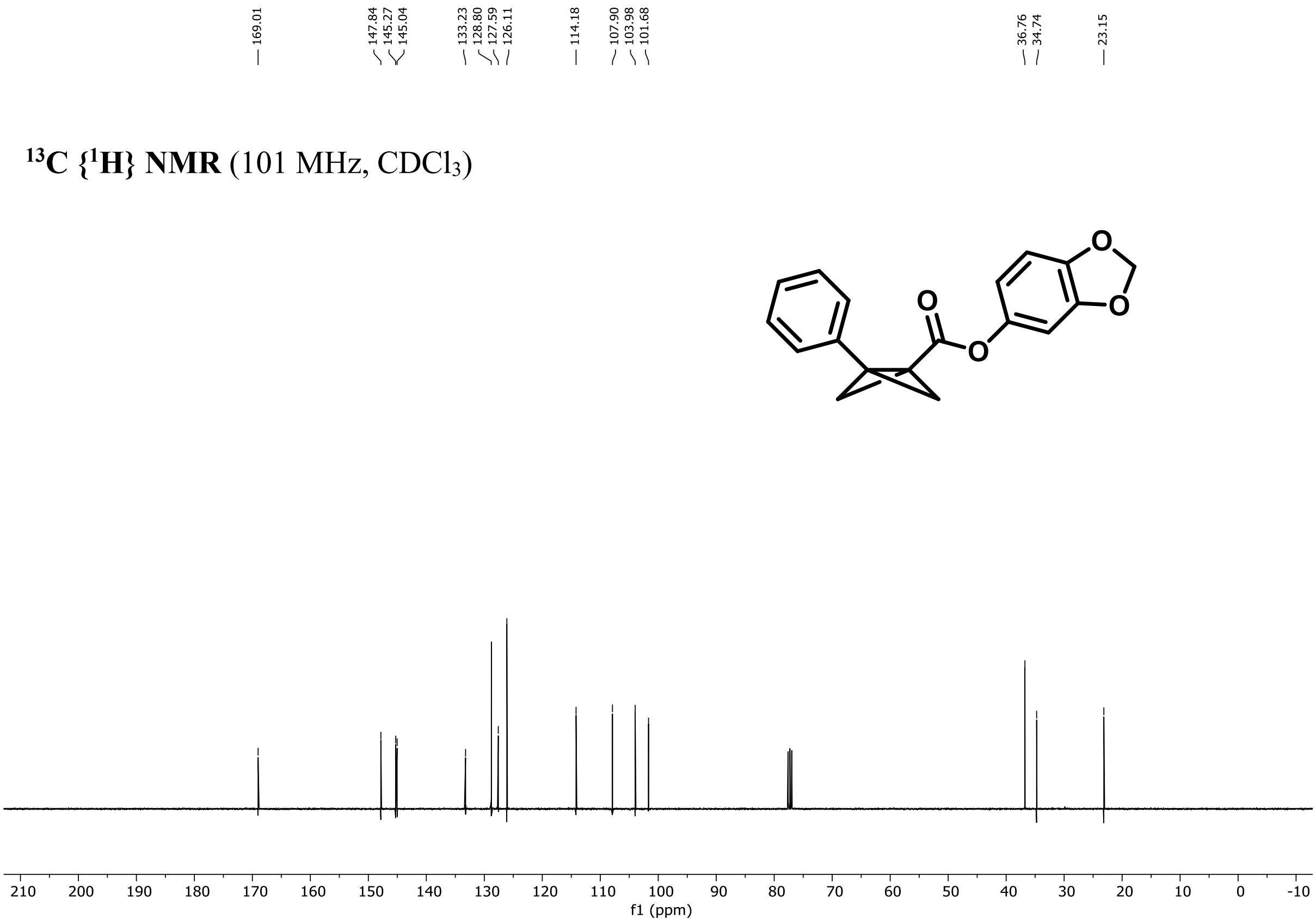

4-Allyl-2-methoxyphenyl 3-phenylbicyclo[1.1.0]butane-1-carboxylate (1q):

^{1}H NMR (400 MHz, $CDCl_3$)

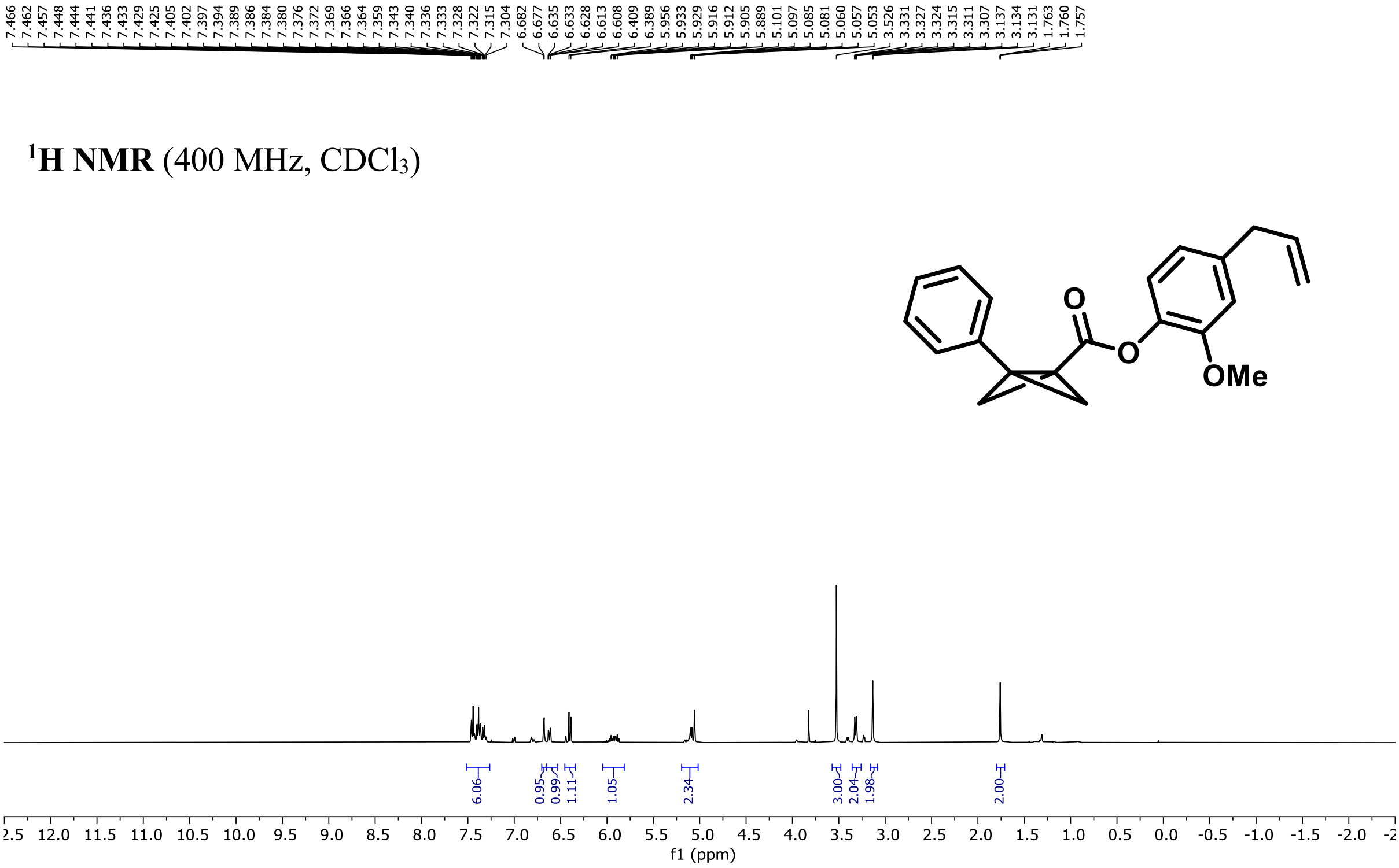

^{13}C {^{1}H} NMR (101 MHz, $CDCl_3$)

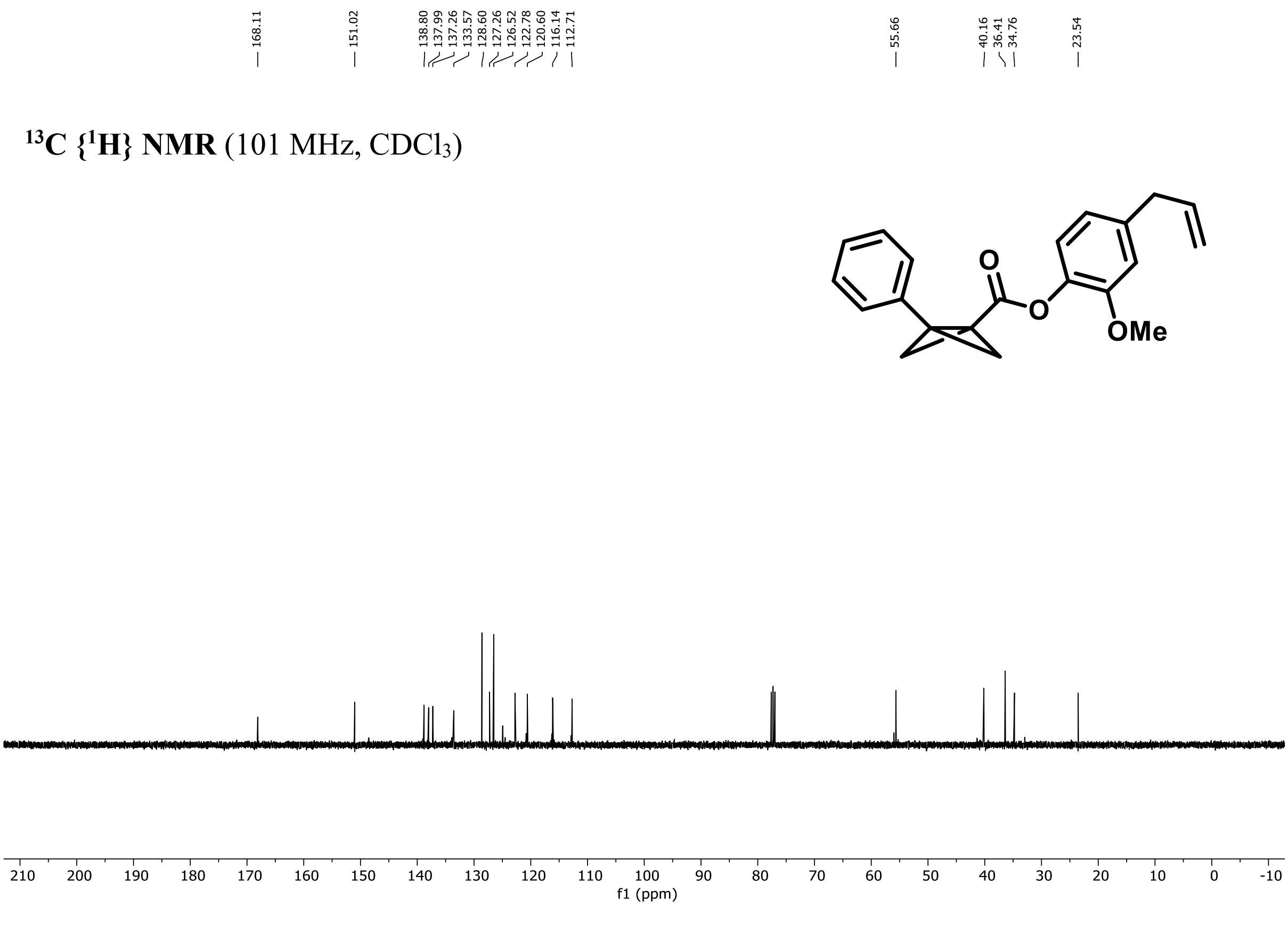

N-Methoxy-N-methyl-3-phenylbicyclo[1.1.0]butane-1-carboxamide (1r):

^{1}H NMR (400 MHz, $CDCl_3$)

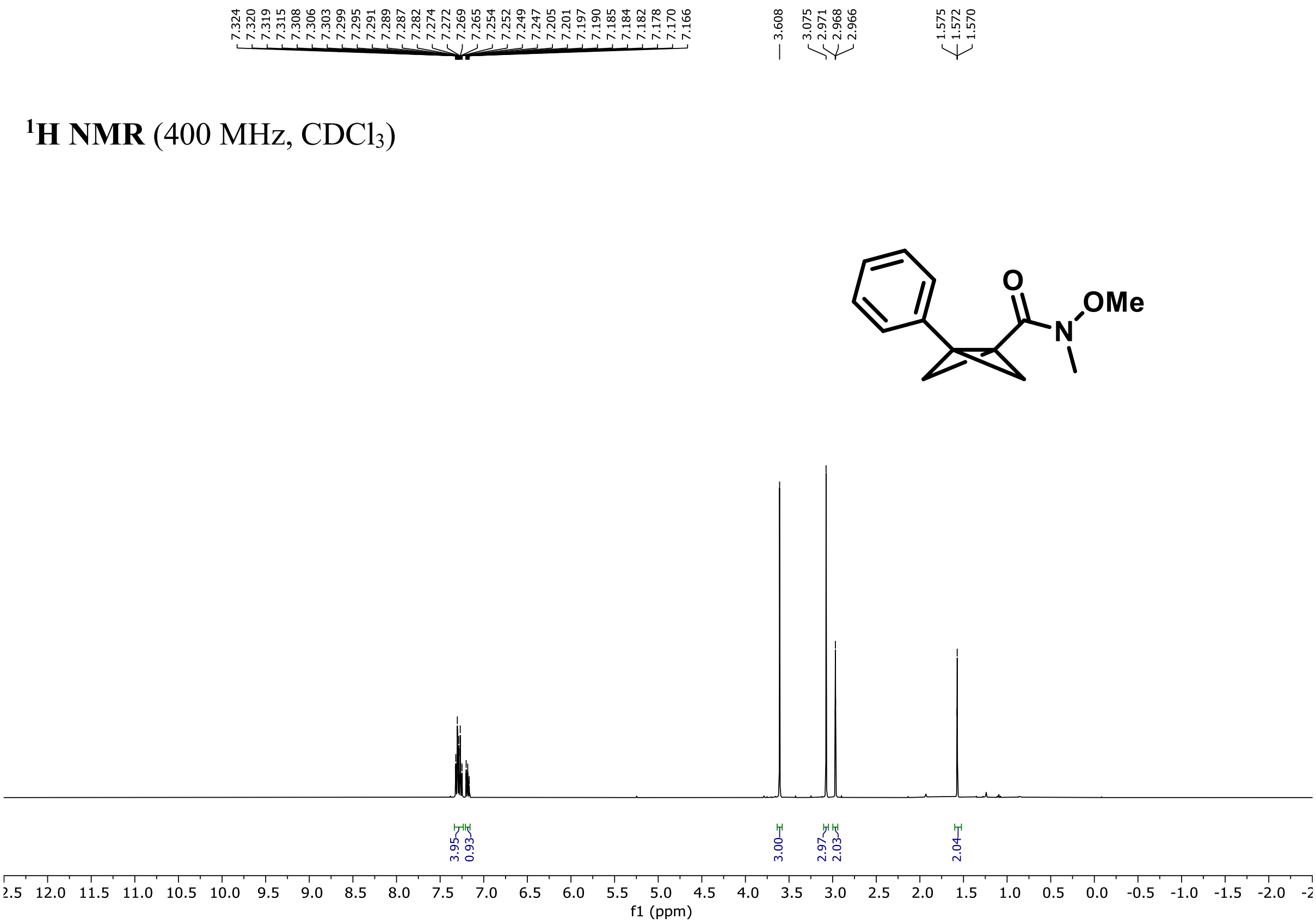

^{13}C {^{1}H} NMR (101 MHz, $CDCl_3$)

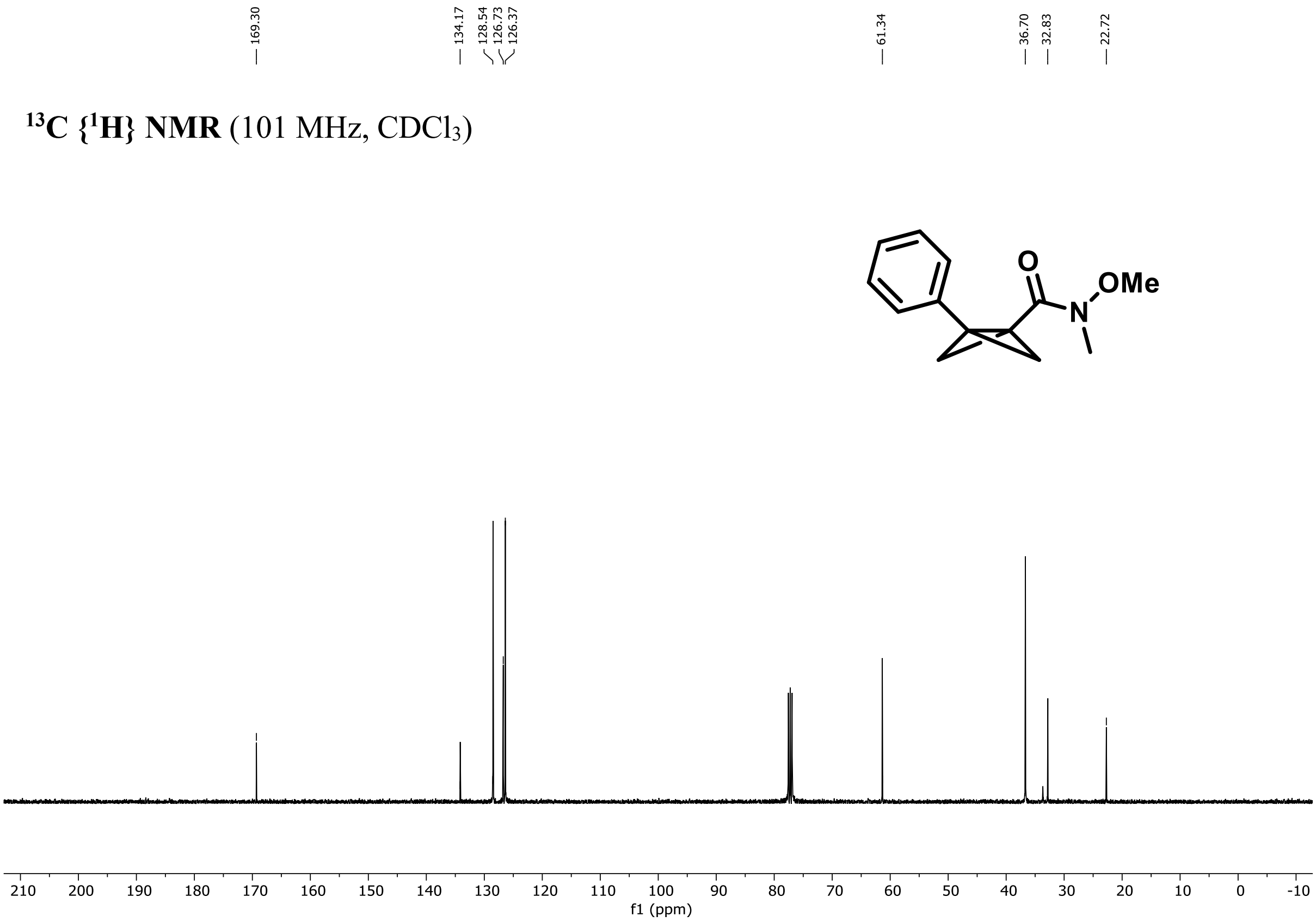

Naphthalen-2-yl(3-phenylbicyclo[1.1.0]butan-1-yl)methanone (1s):

8.136, 8.135, 8.133, 8.131, 8.129, 8.127, 7.893, 7.891, 7.888, 7.873, 7.871, 7.869, 7.867, 7.838, 7.835, 7.832, 7.817, 7.814, 7.813, 7.774, 7.772, 7.770, 7.769, 7.752, 7.751, 7.749, 7.748, 7.574, 7.571, 7.570, 7.567, 7.556, 7.553, 7.550, 7.546, 7.537, 7.534, 7.532, 7.530, 7.514, 7.511, 7.248, 7.210, 7.205, 7.199, 7.198, 7.196, 7.192, 7.148, 7.145, 7.142, 7.140, 7.137, 7.135, 7.129, 7.123, 3.238, 3.235, 3.232, 1.977, 1.974, 1.971

^{1}H NMR (400 MHz, $CDCl_3$)

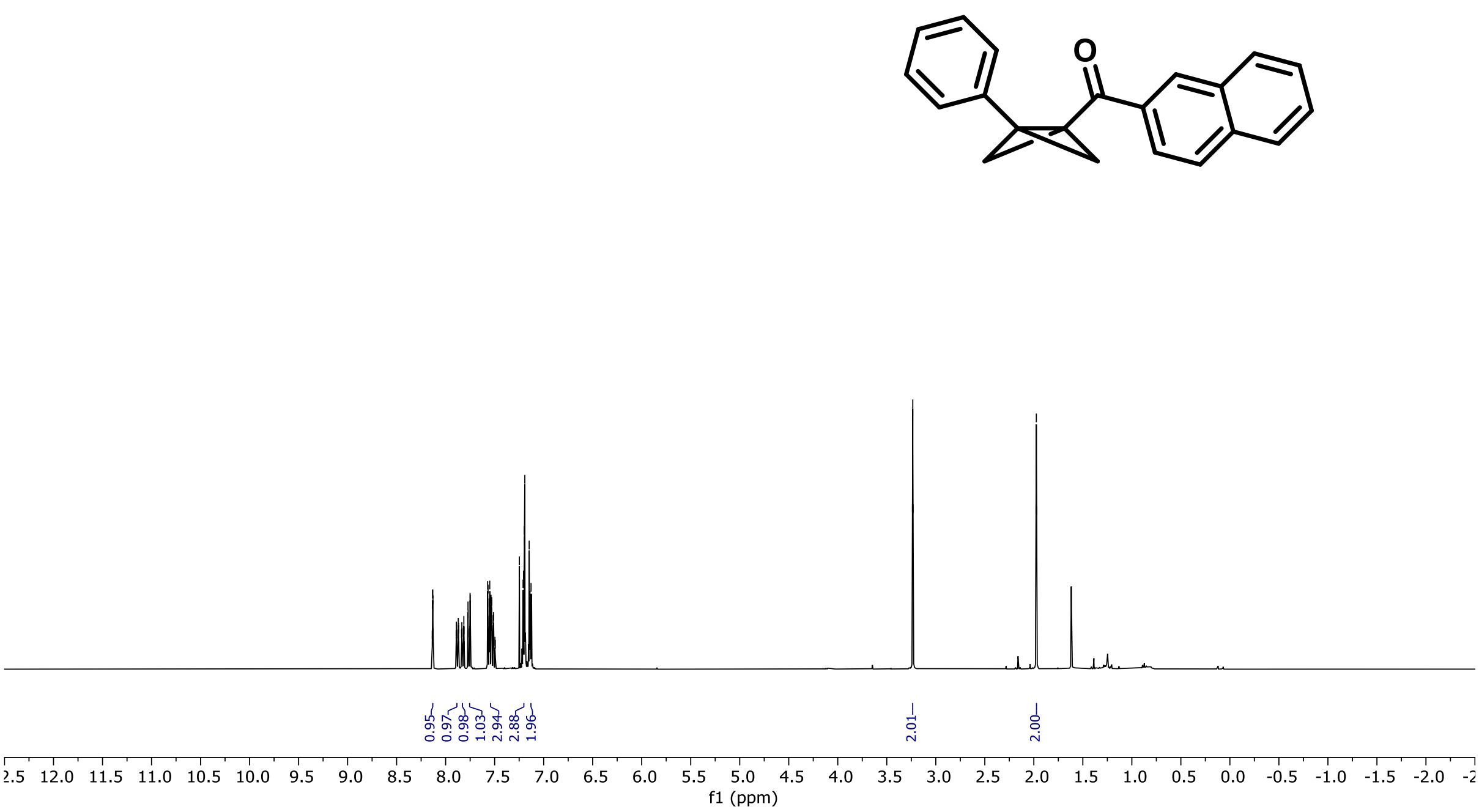

196.528, 135.729, 135.065, 132.890, 132.287, 129.871, 129.363, 128.510, 128.049, 128.019, 127.855, 127.343, 126.688, 126.154, 124.535, 38.897, 37.953, 31.579

^{13}C {^{1}H} NMR (101 MHz, $CDCl_3$)

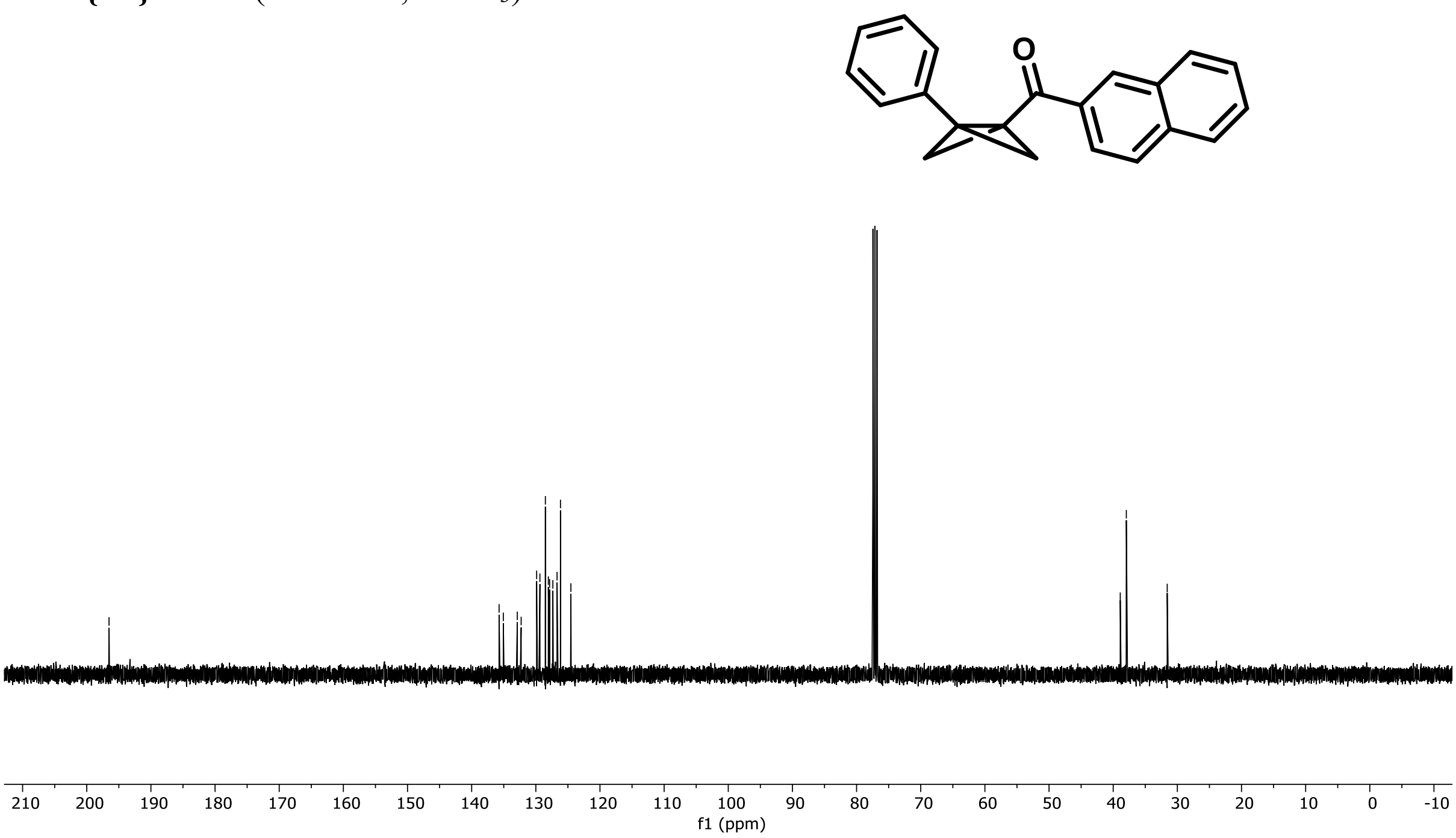

(3-Methoxyphenyl)(3-phenylbicyclo[1.1.0]butan-1-yl)methanone (1t):

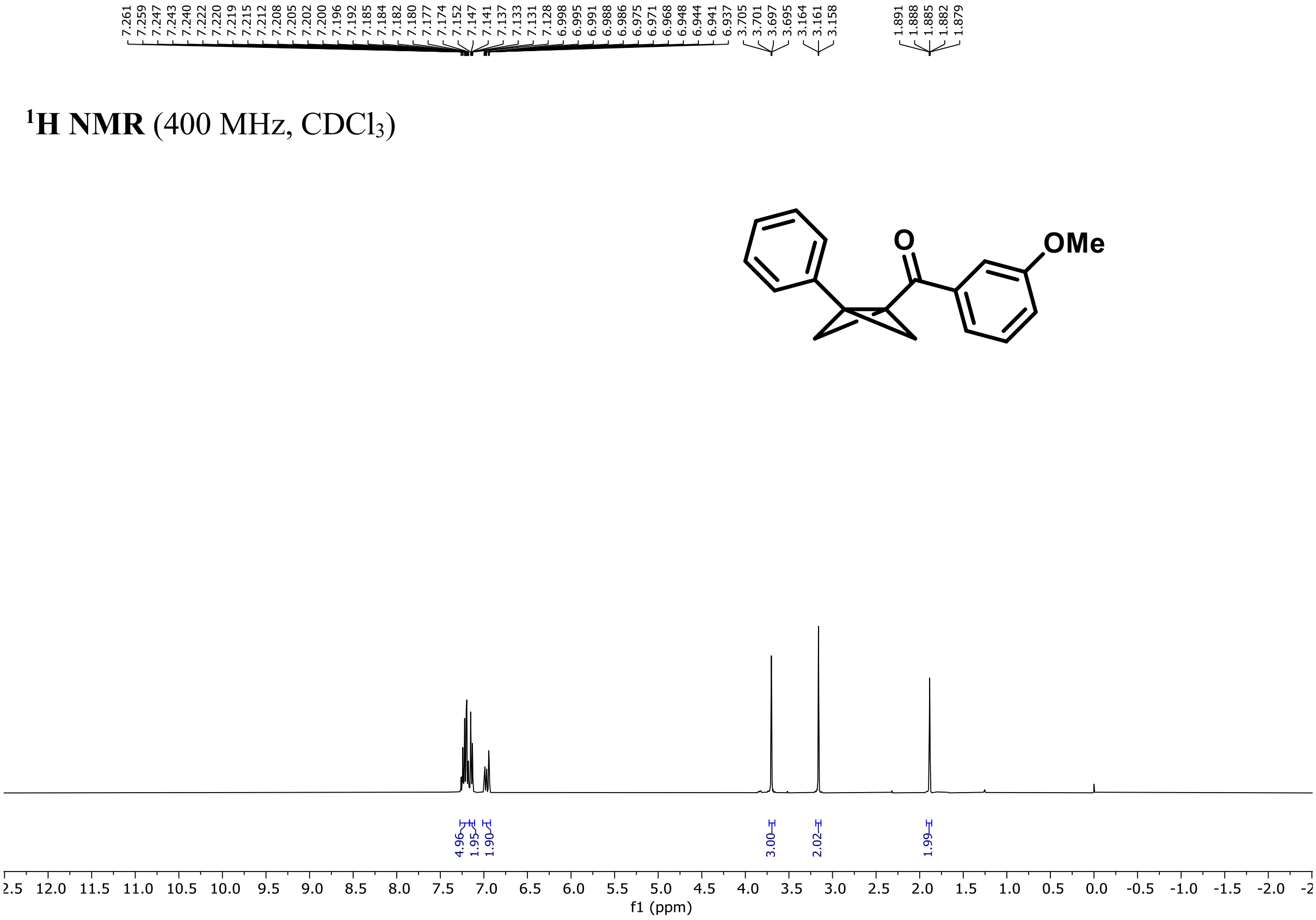

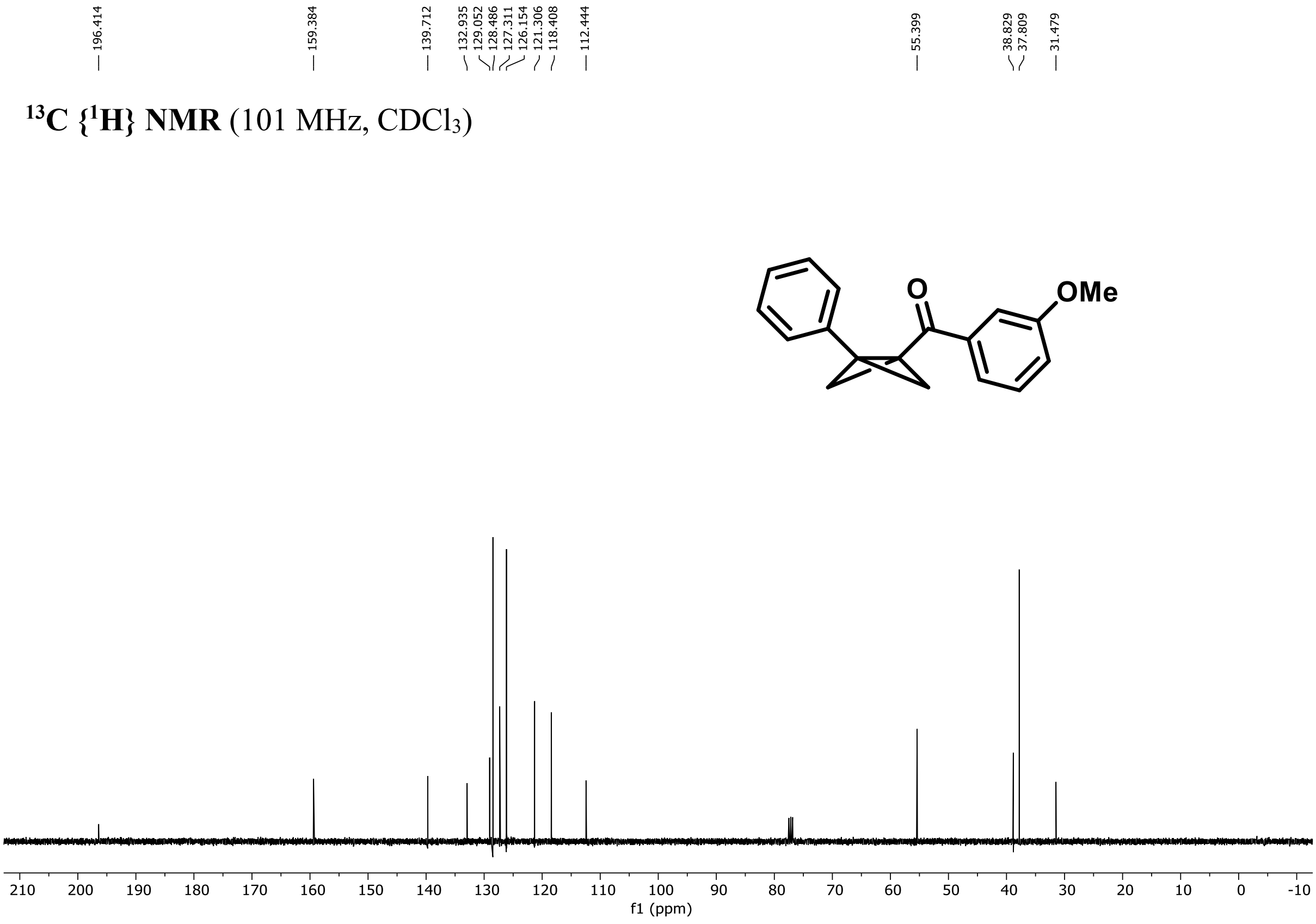

(3,5-dimethyl-1*H*-pyrazol-1-yl)(3-phenylbicyclo[1.1.0]butan-1-yl)methanone (1u):

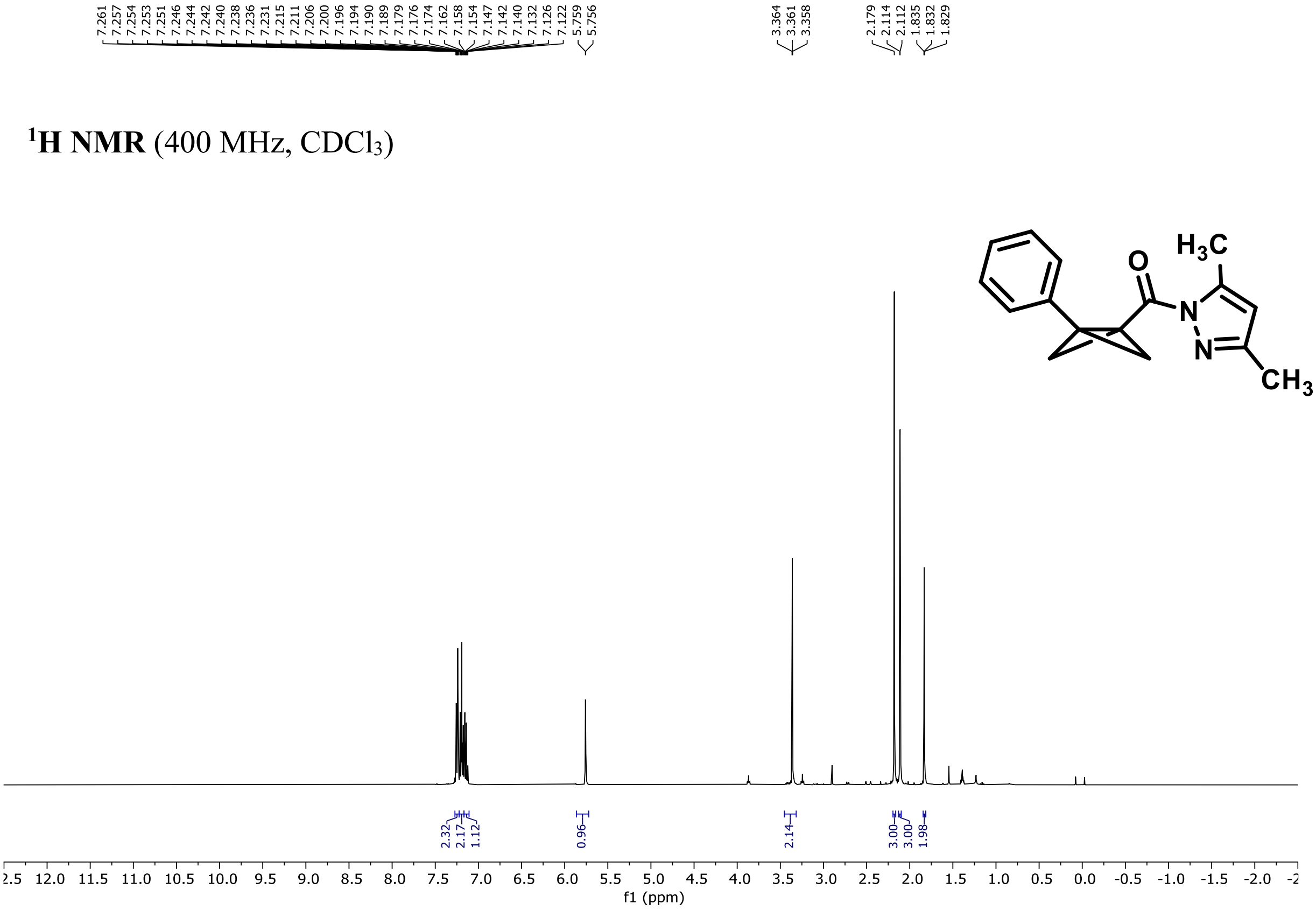

^{1}H NMR (400 MHz, $CDCl_3$)

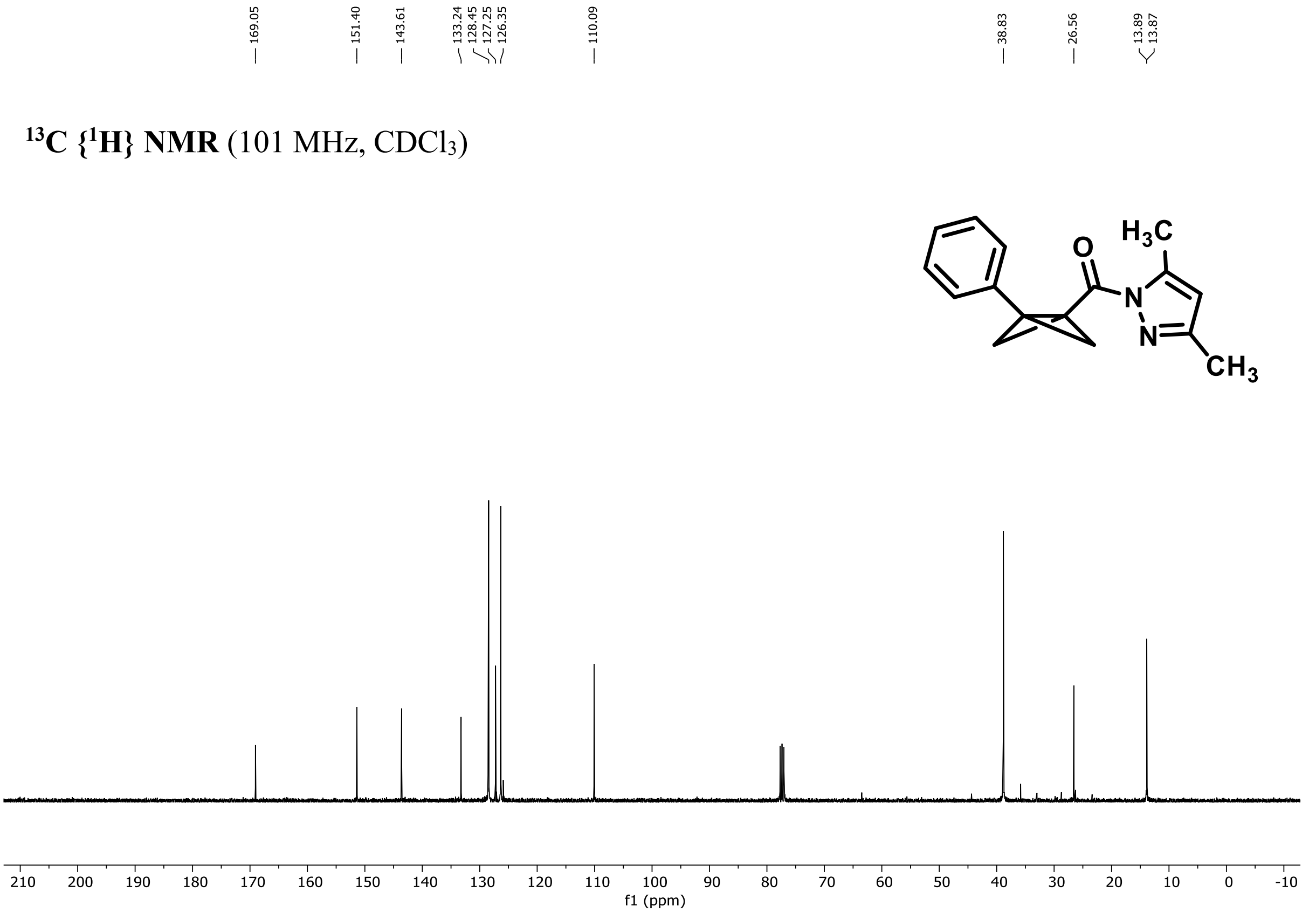

^{13}C {^{1}H} NMR (101 MHz, $CDCl_3$)

Benzyl-bicyclo[1.1.0]butane-1-carboxylate (1v):

^{1}H NMR (400 MHz, $CDCl_3$)

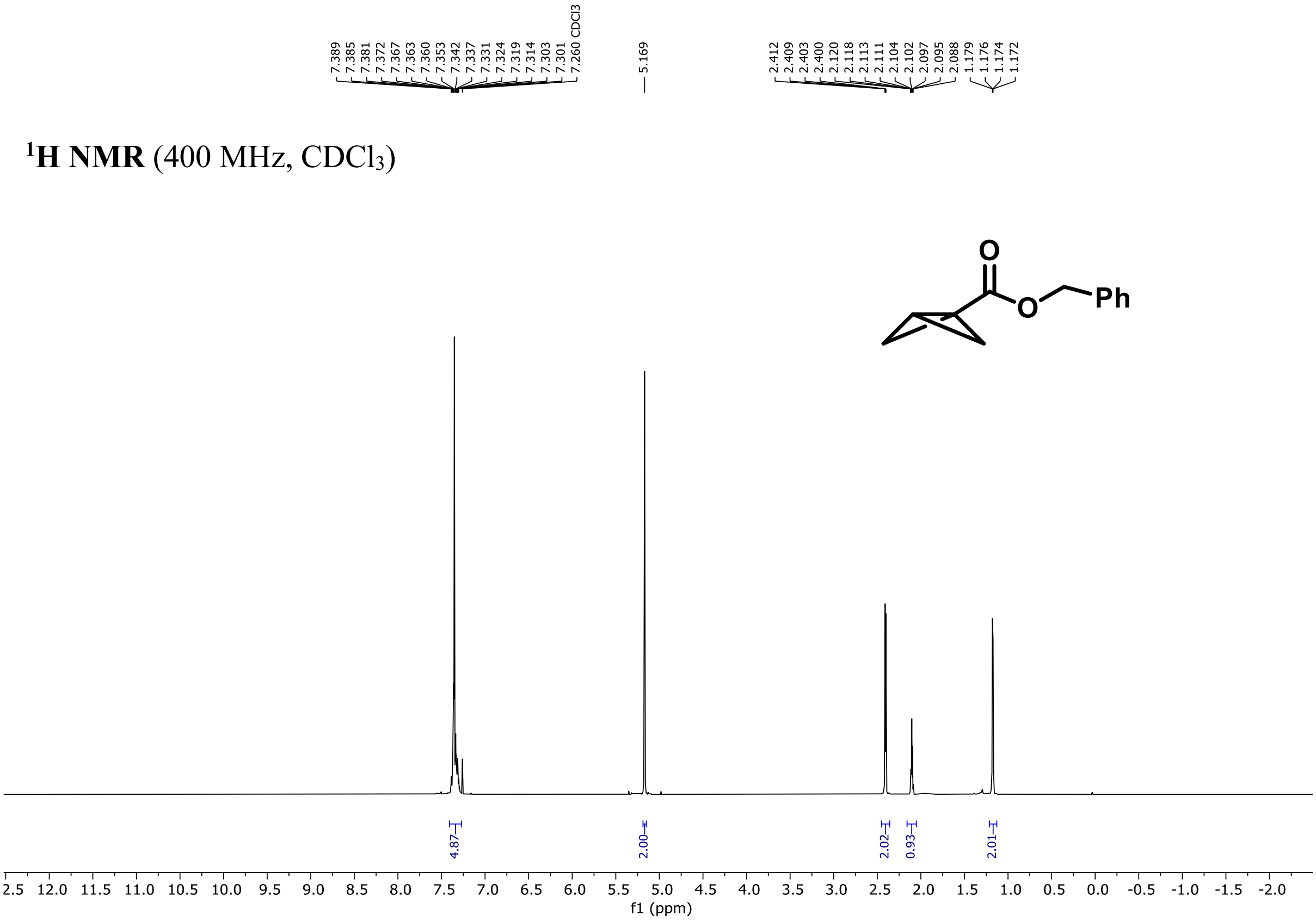

^{13}C {^{1}H} NMR (101 MHz, $CDCl_3$)

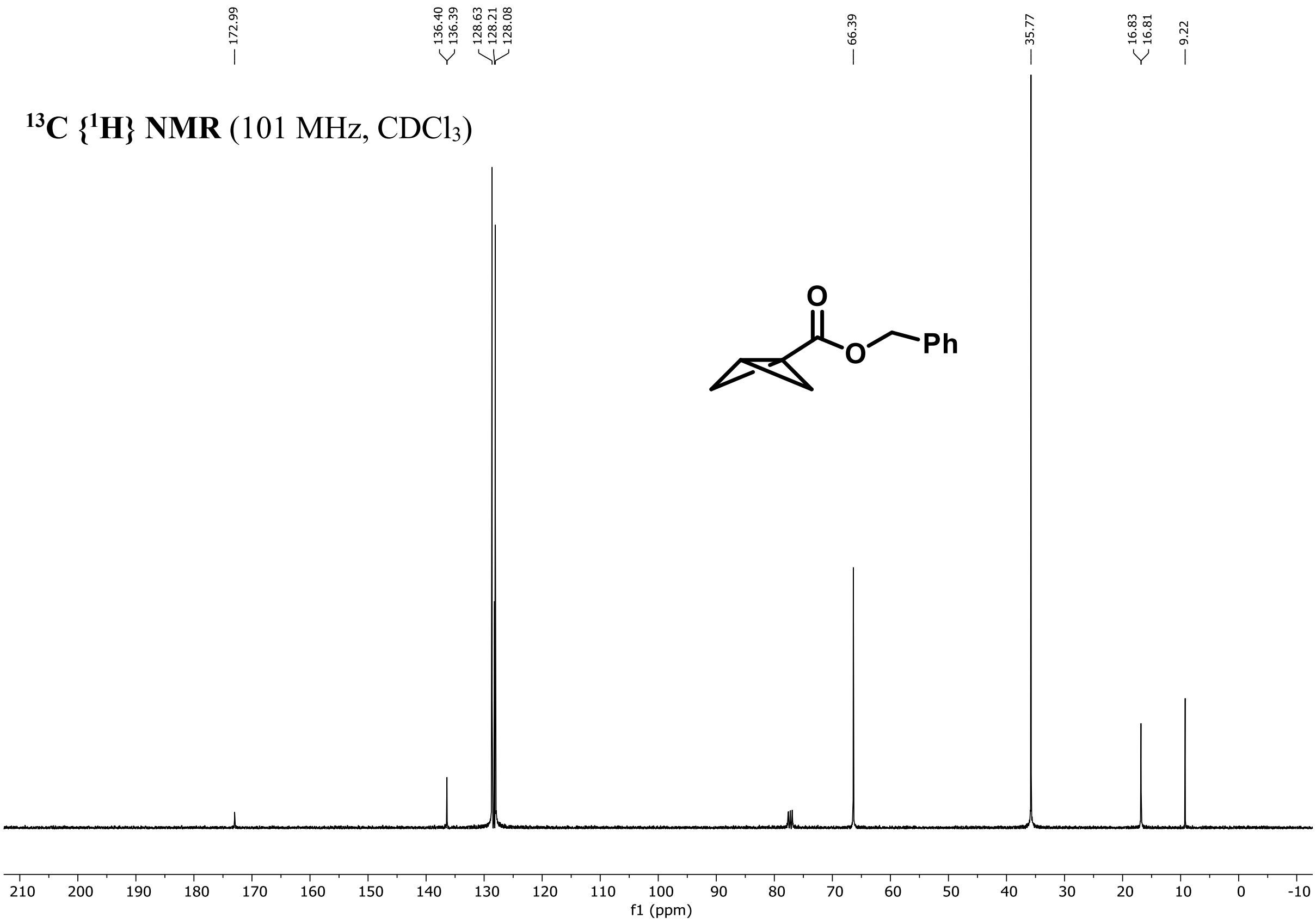

***N*-Methyl-*N*-phenylbicyclo[1.1.0]butane-1-carboxamide (1w):**

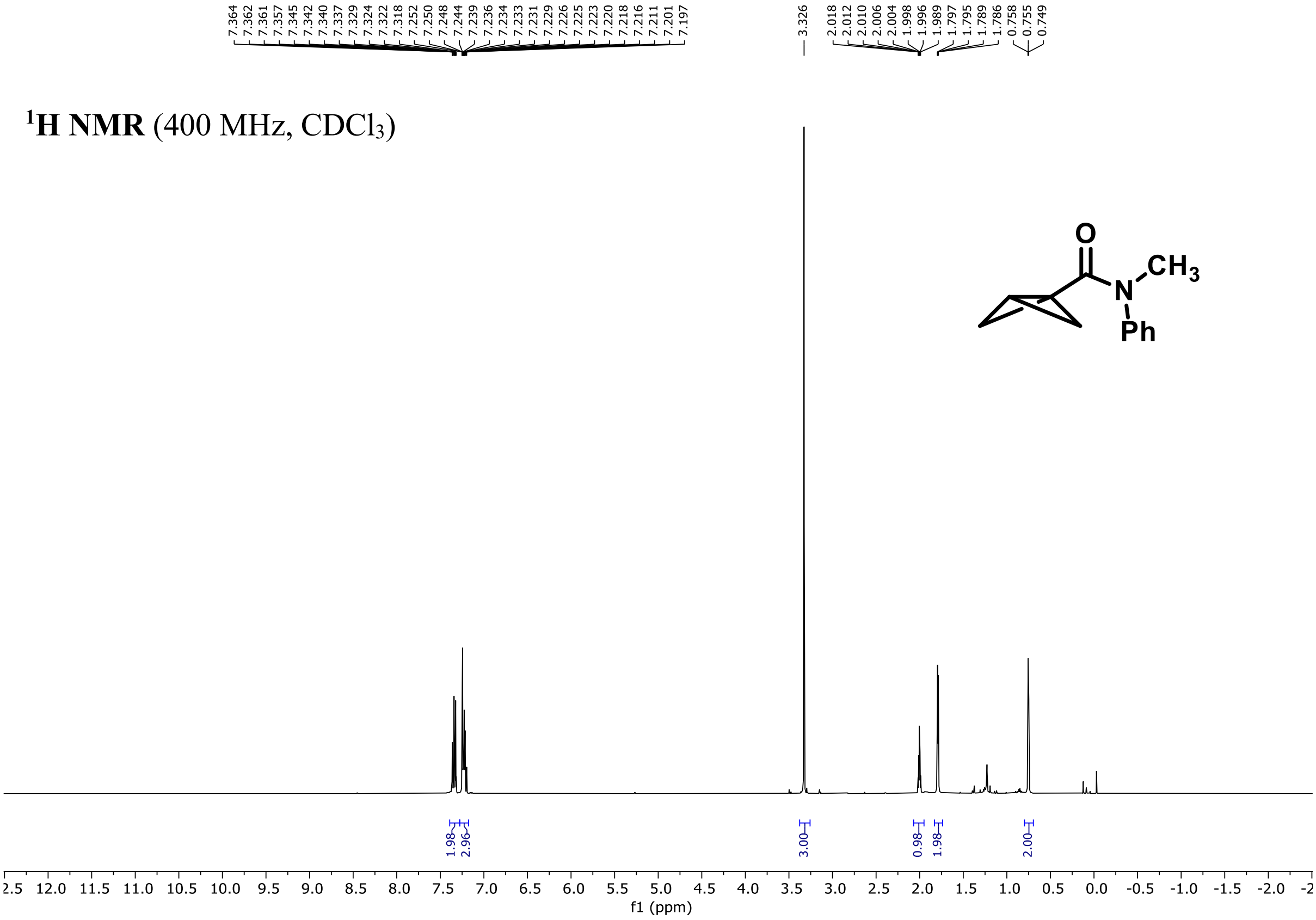

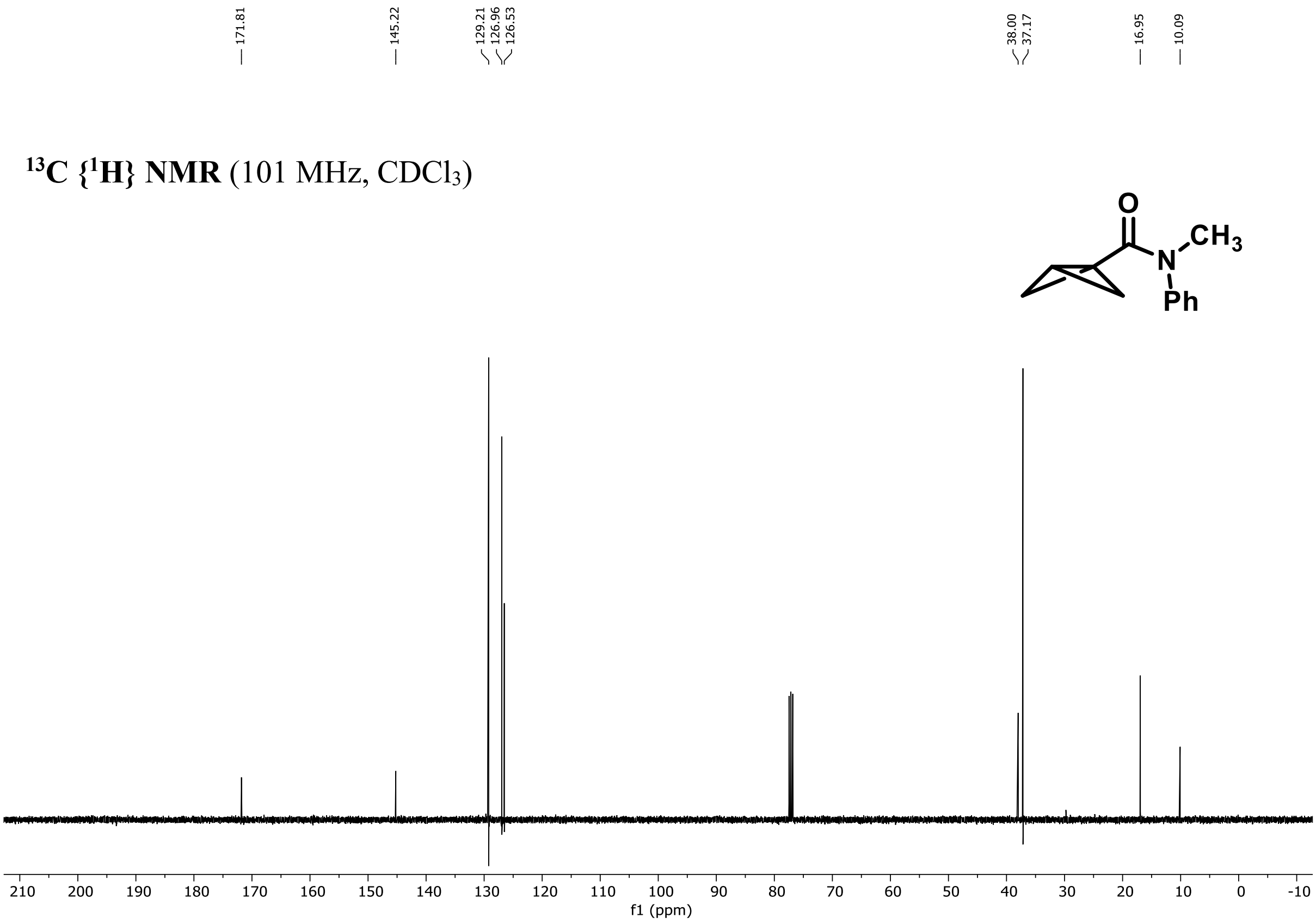

Benzyl-bicyclo[1.1.0]butane-1-carboxylate (1x):

^{1}H NMR (400 MHz, $CDCl_3$)

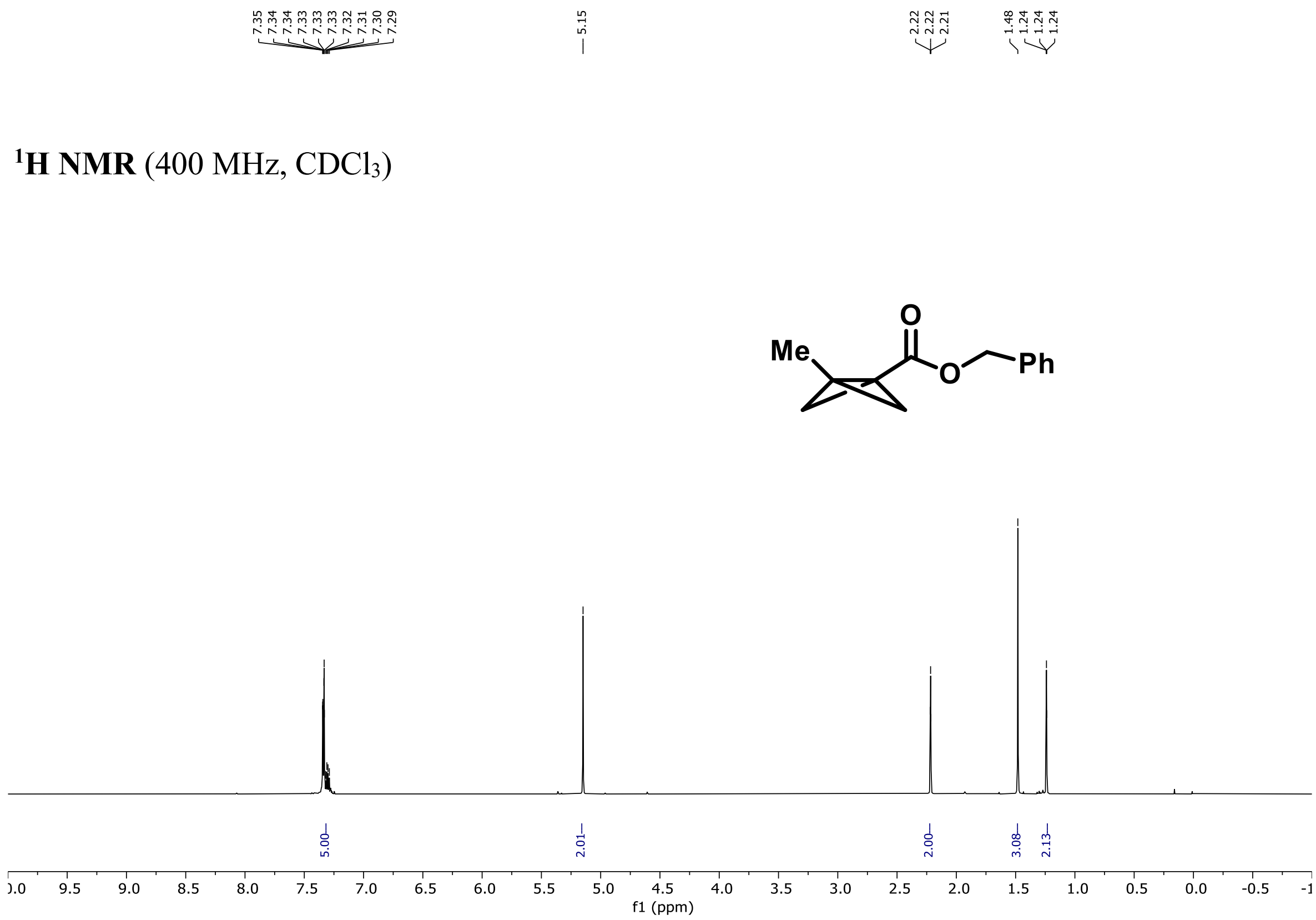

^{13}C {^{1}H} NMR (101 MHz, $CDCl_3$)

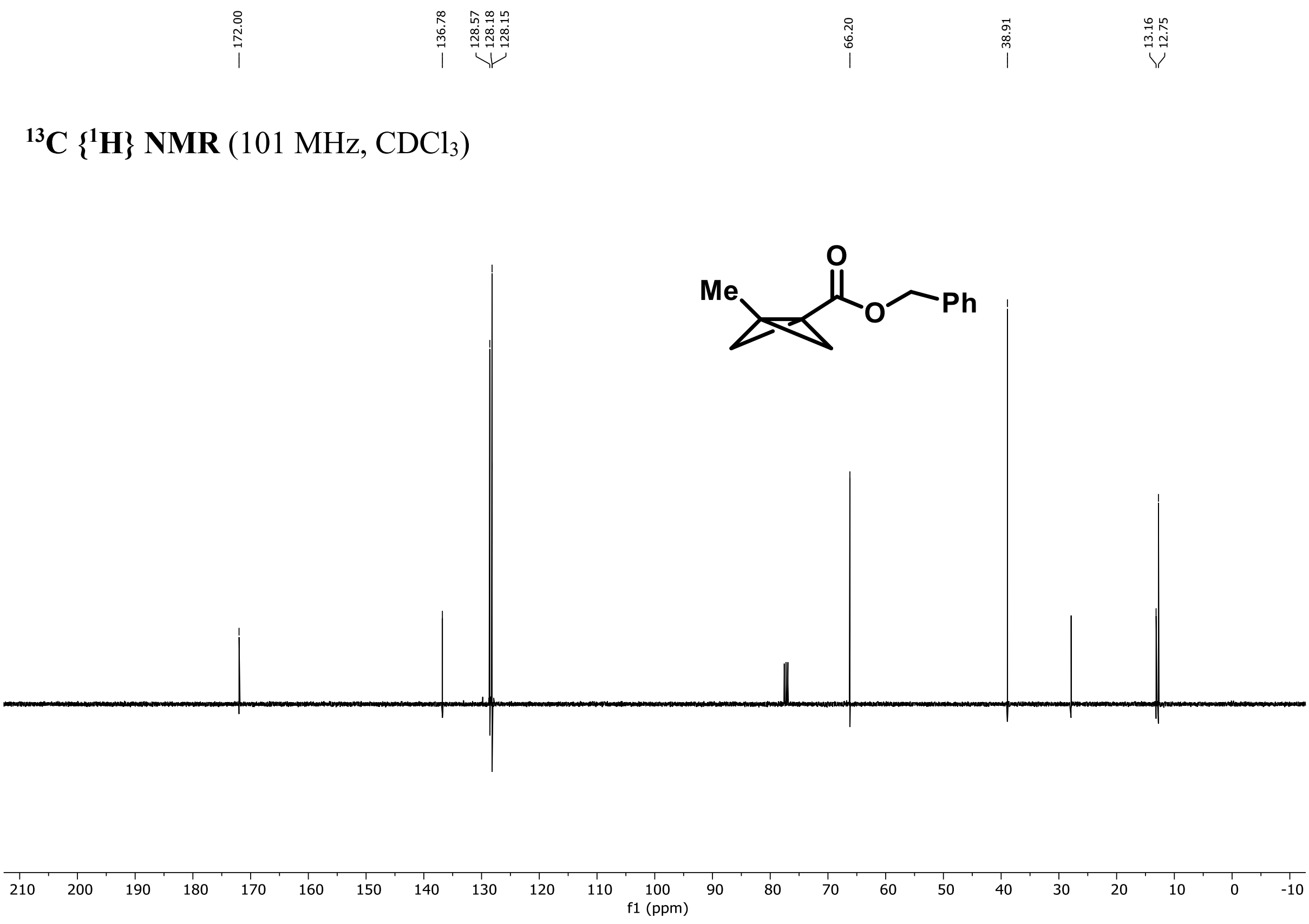

(3-Methyl-1*H*-indol-2-yl)(phenyl)methanol (2a):

^{1}H NMR (400 MHz, $CDCl_3$)

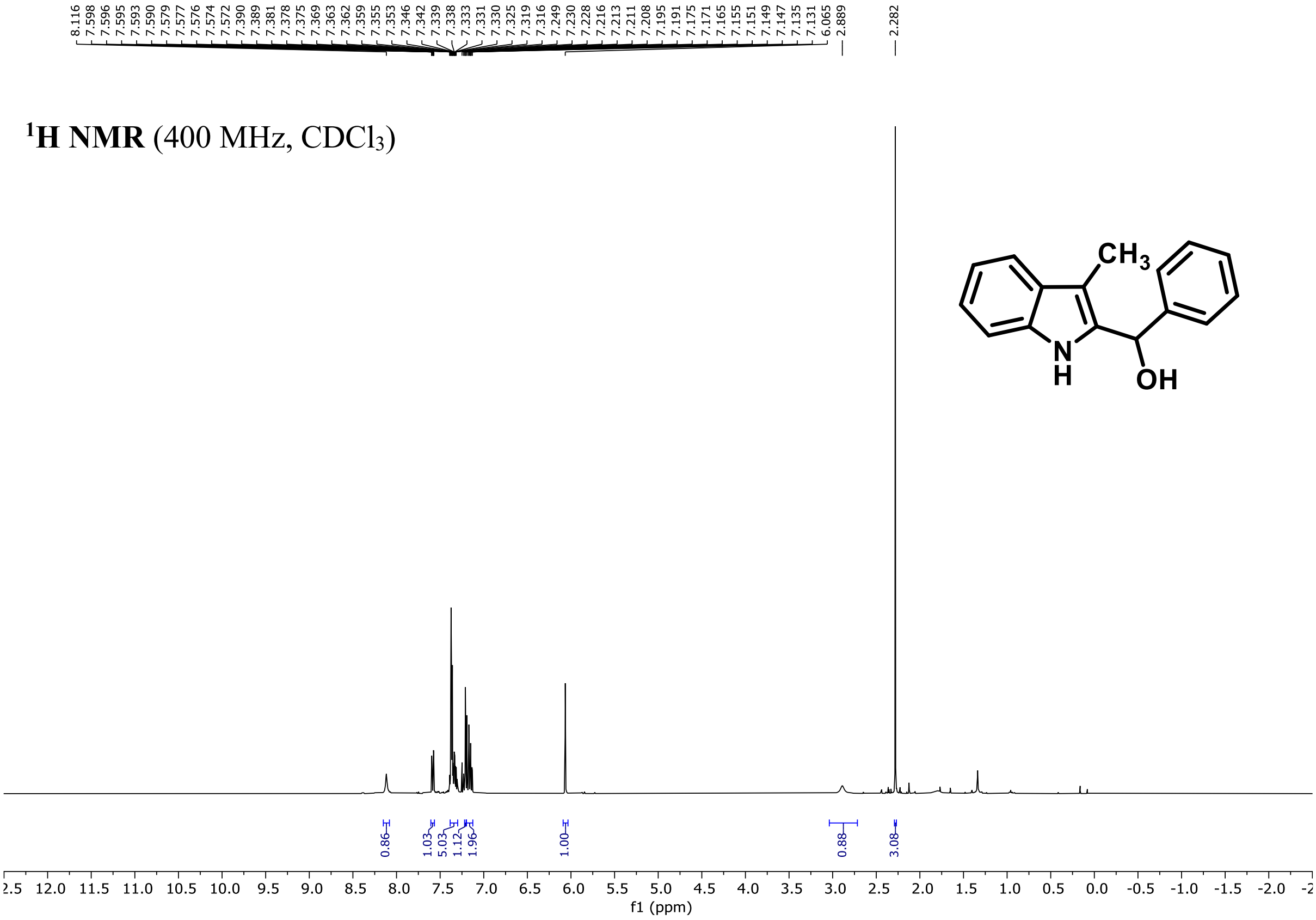

^{13}C {^{1}H} NMR (101 MHz, $CDCl_3$)

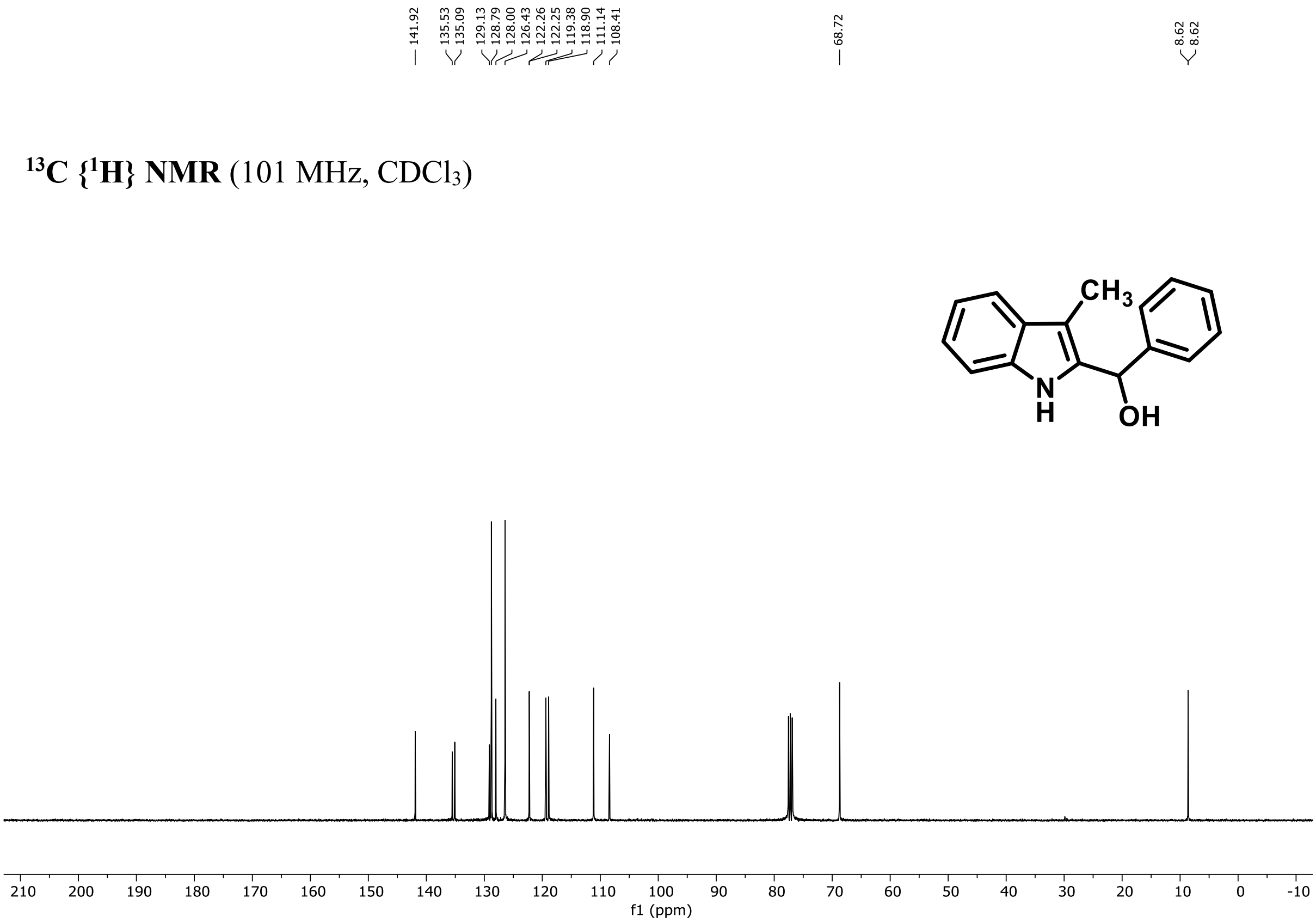

(3-Methyl-1*H*-indol-2-yl)(*p*-tolyl)methanol (2b):

^{1}H NMR (400 MHz, $CDCl_3$)

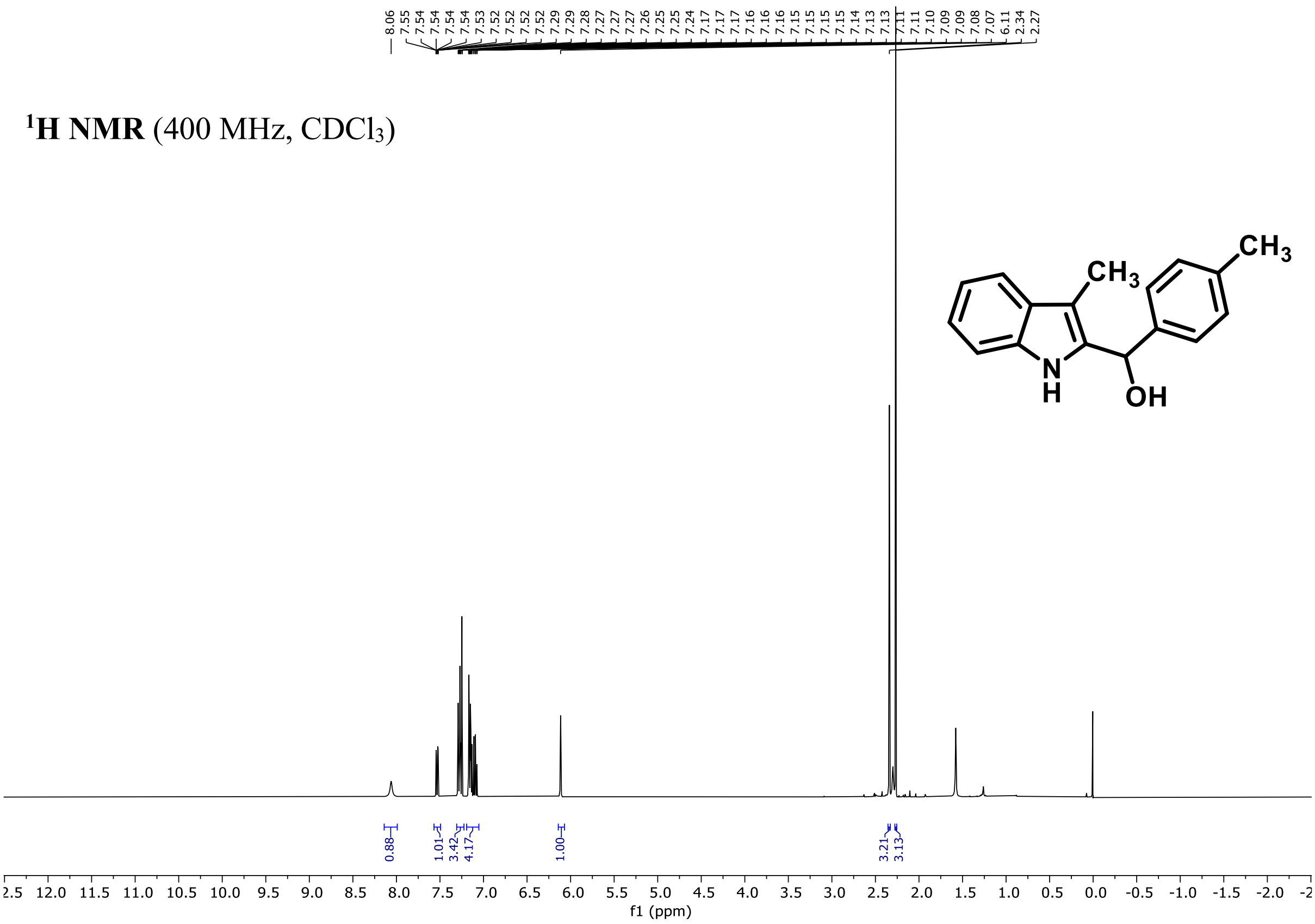

^{13}C {^{1}H} NMR (101 MHz, $CDCl_3$)

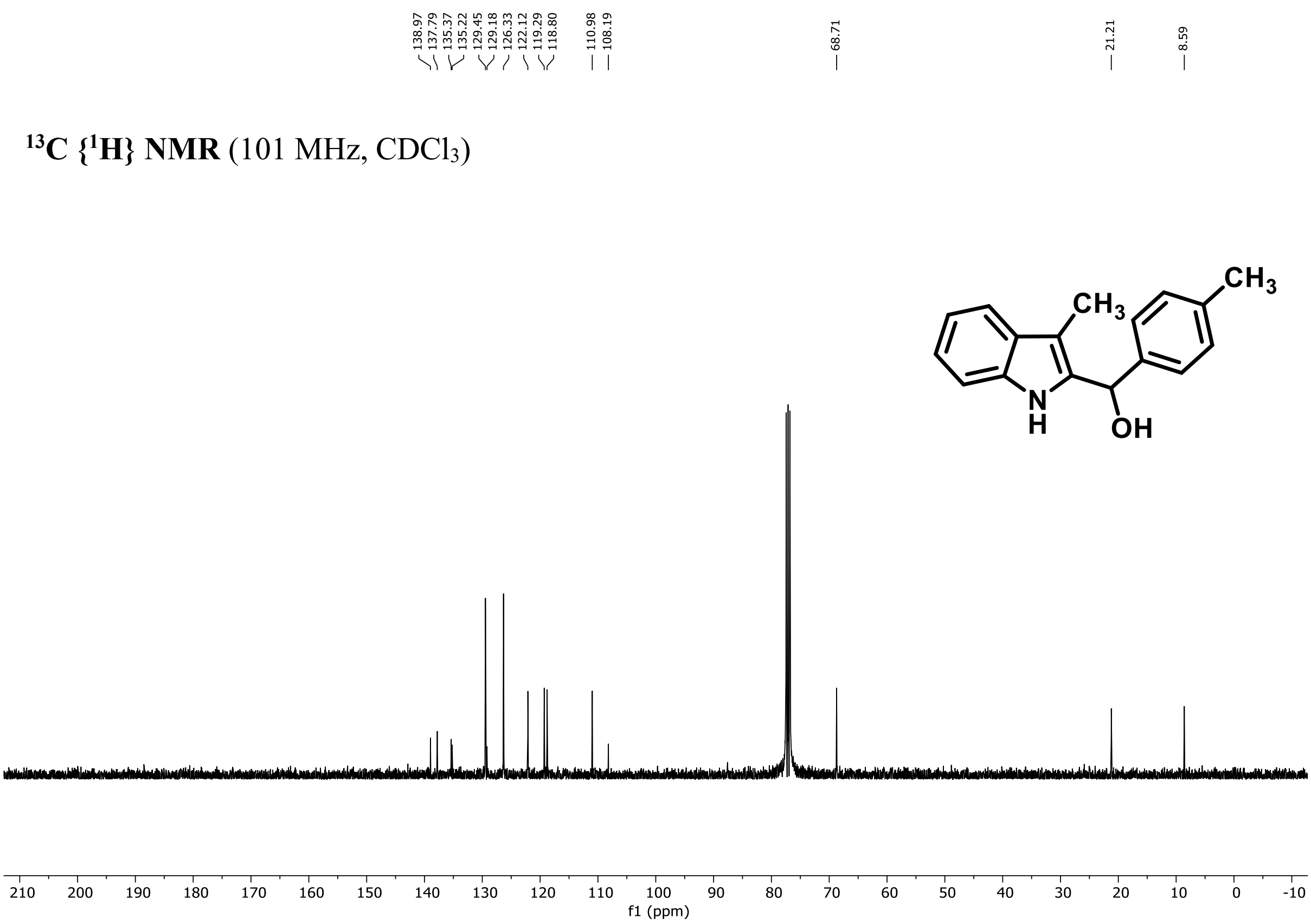

(4-(*tert*-butyl)phenyl)(3-methyl-1*H*-indol-2-yl)methanol (2c):

^{1}H NMR (400 MHz, $CDCl_3$)

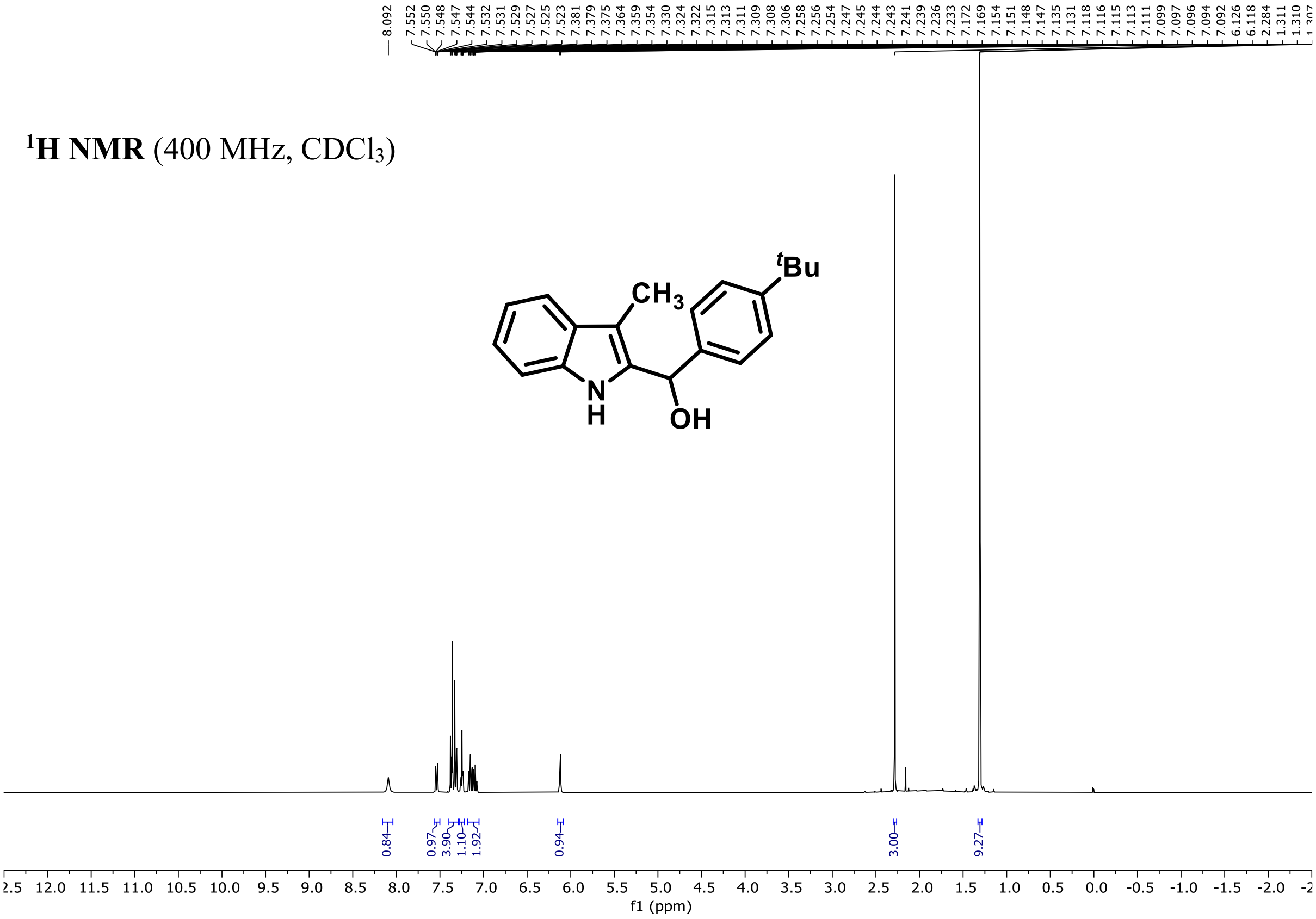

^{13}C {^{1}H} NMR (101 MHz, $CDCl_3$)

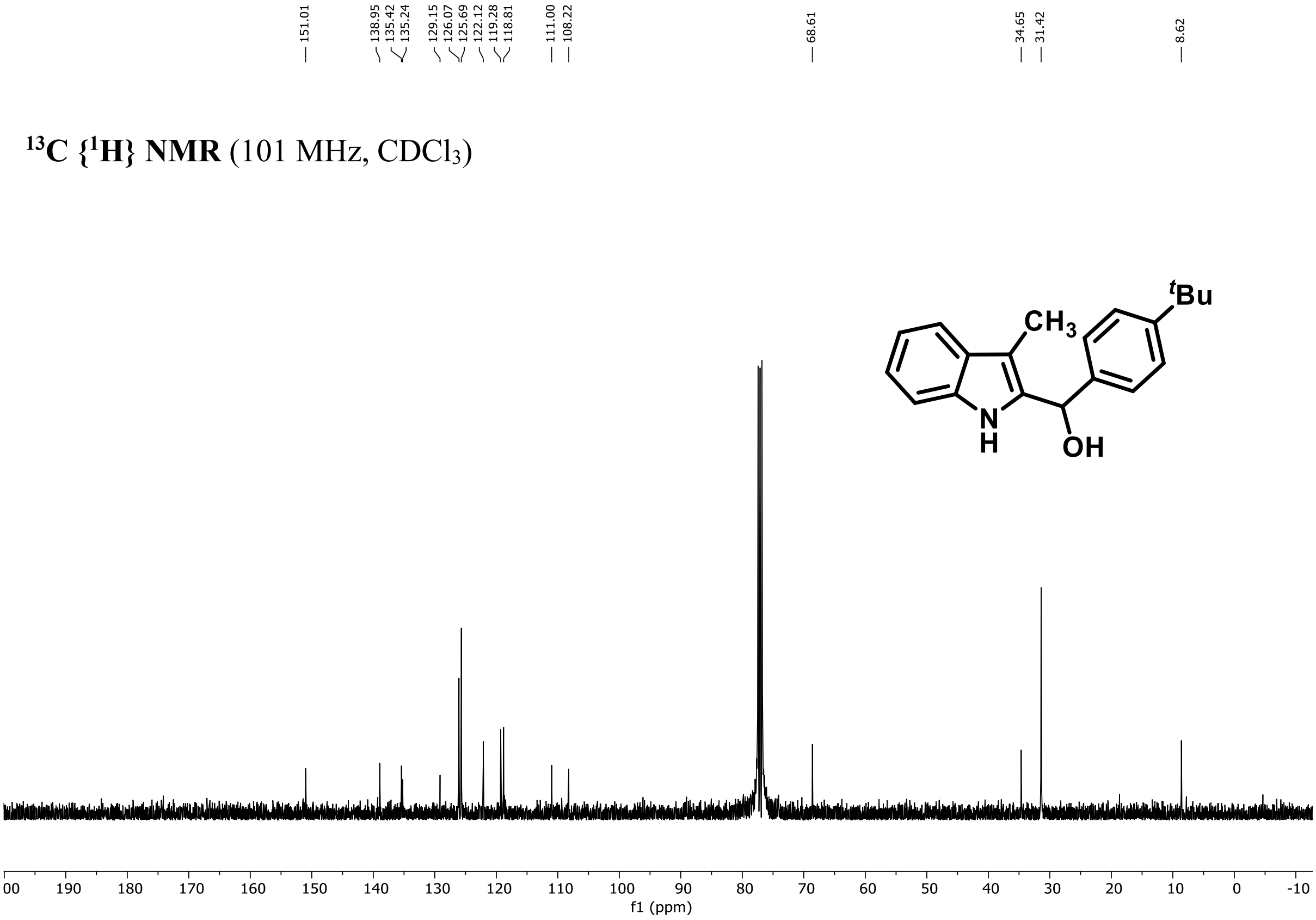

(3-Methyl-1*H*-indol-2-yl)(naphthalen-2-yl)methanol (2d):

^{1}H NMR (400 MHz, DMSO-d6)

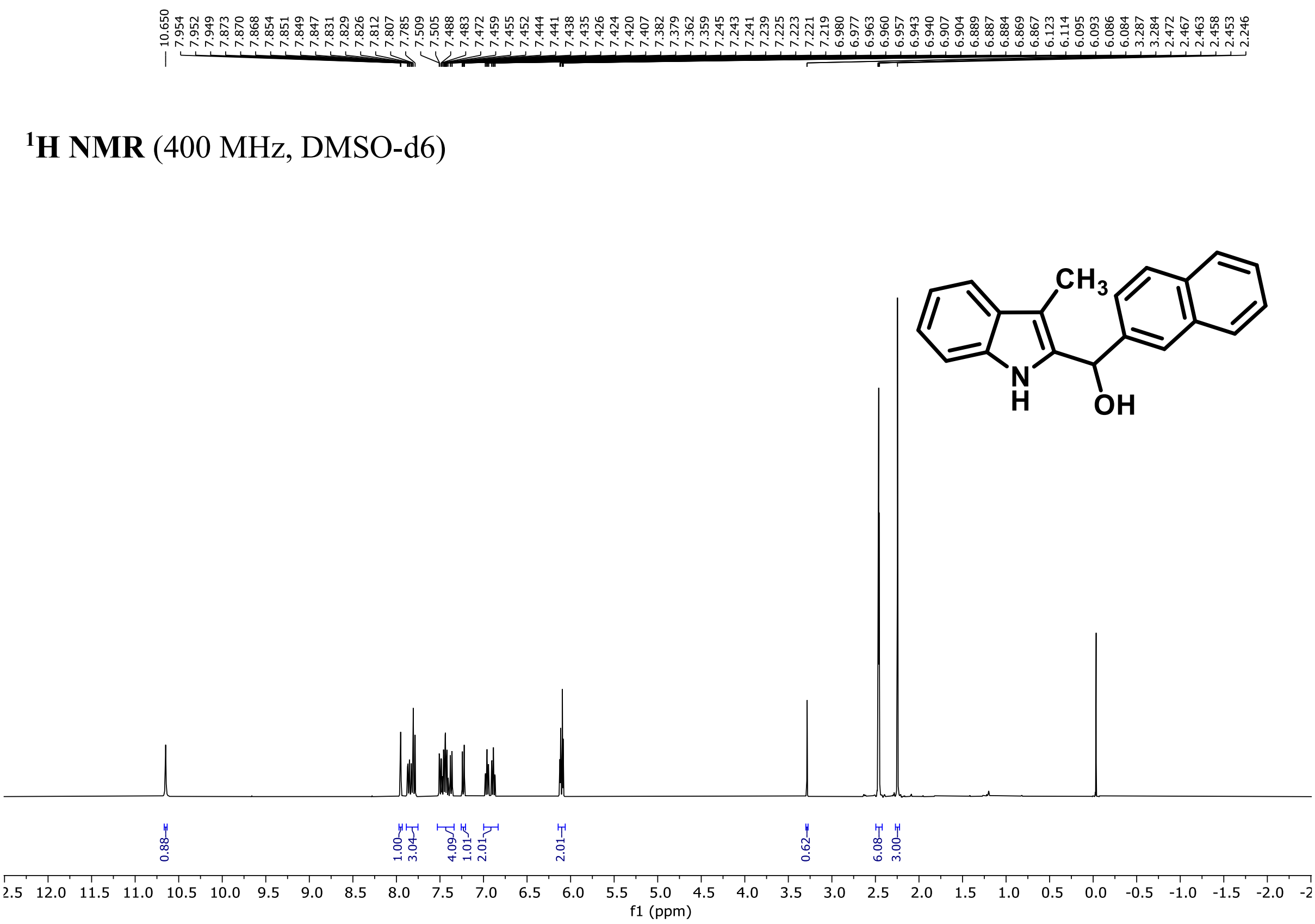

^{13}C {^{1}H}NMR (101 MHz, DMSO-d6)

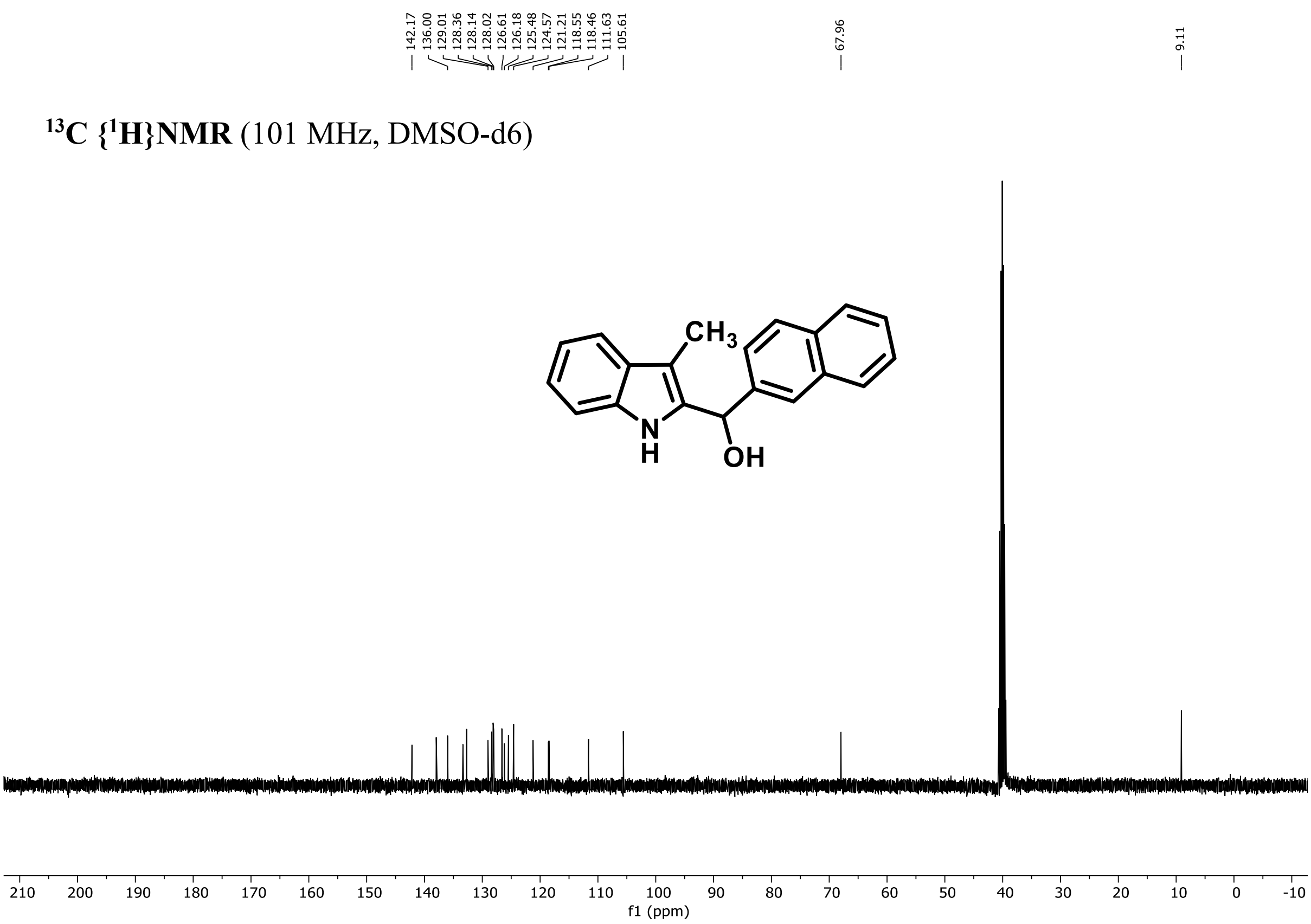

(4-Methoxyphenyl)(3-methyl-1*H*-indol-2-yl)methanol (2e):

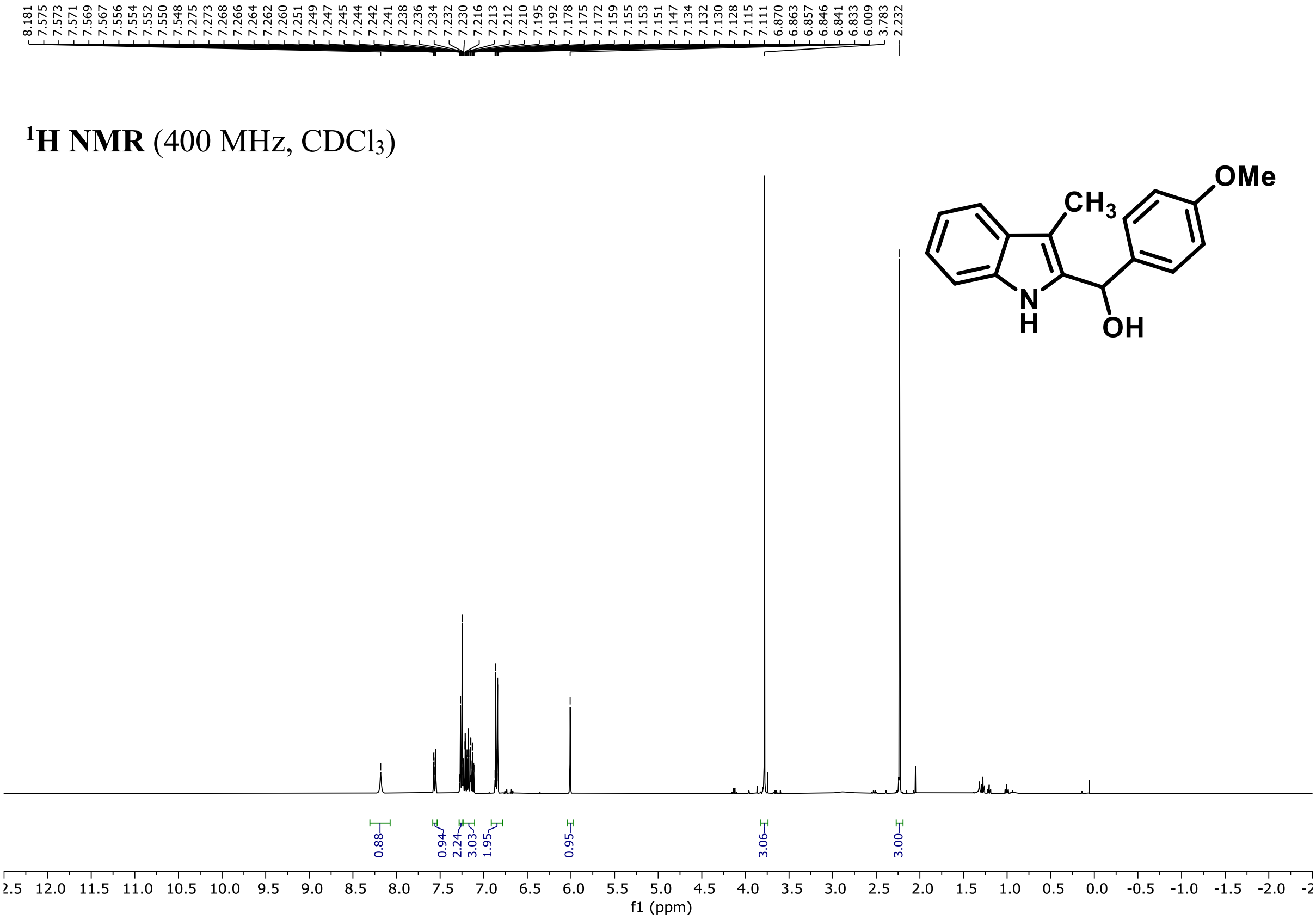

^{1}H NMR (400 MHz, $CDCl_3$)

^{13}C {^{1}H} NMR (101 MHz, $CDCl_3$)

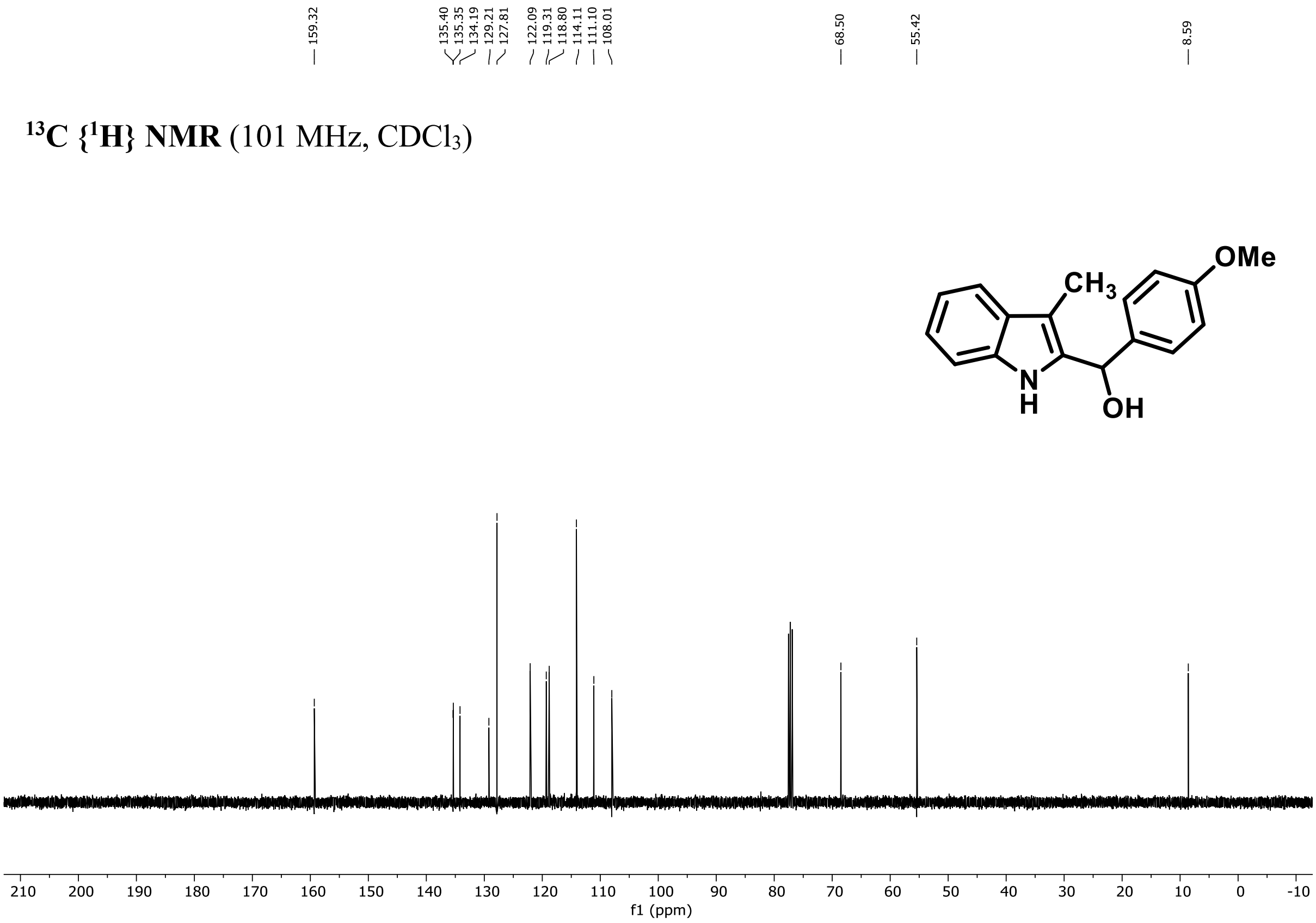

(3-Methoxyphenyl)(3-methyl-1*H*-indol-2-yl)methanol (2f):

^{1}H NMR (400 MHz, $CDCl_3$)

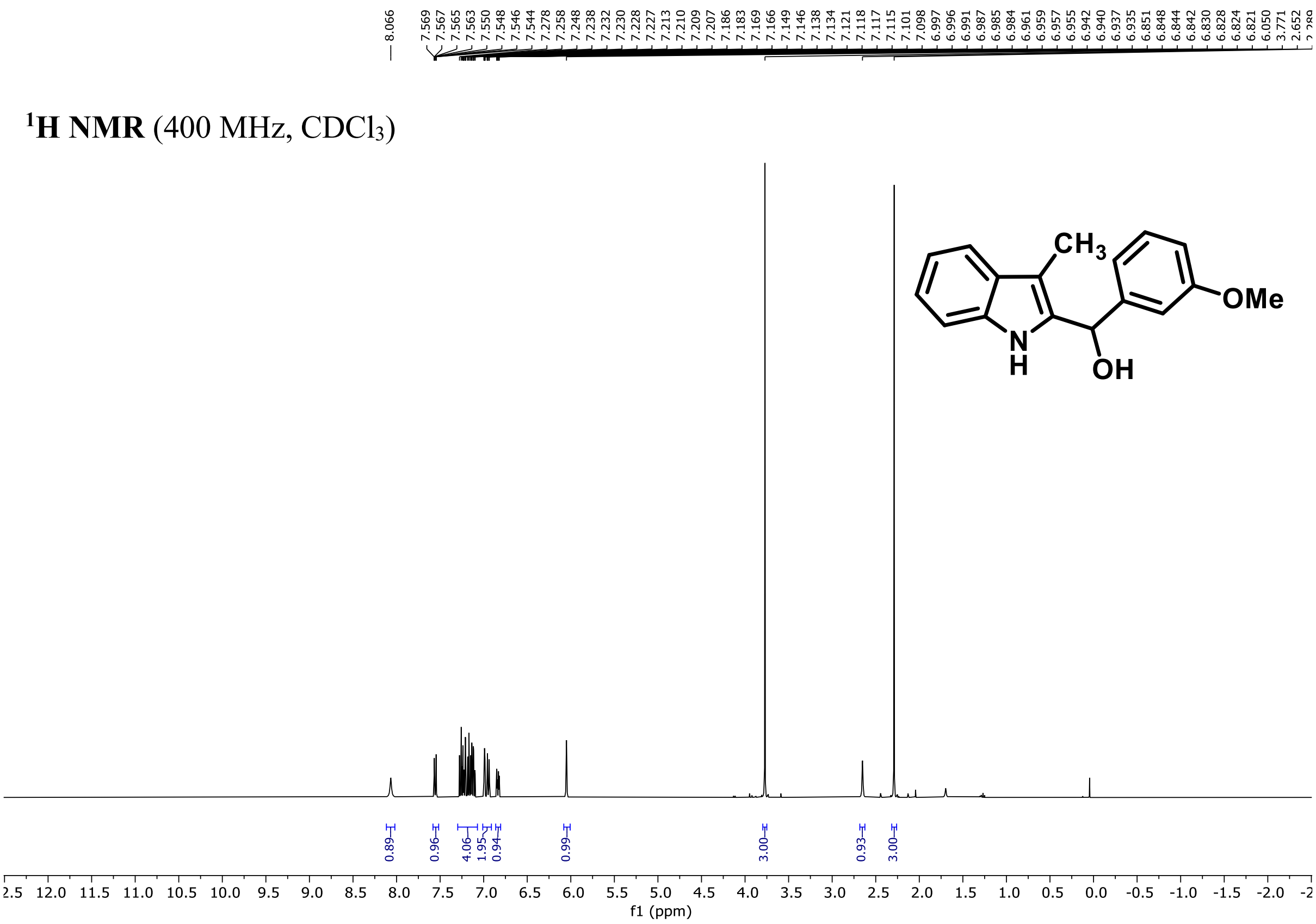

^{13}C {^{1}H} NMR (101 MHz, $CDCl_3$)

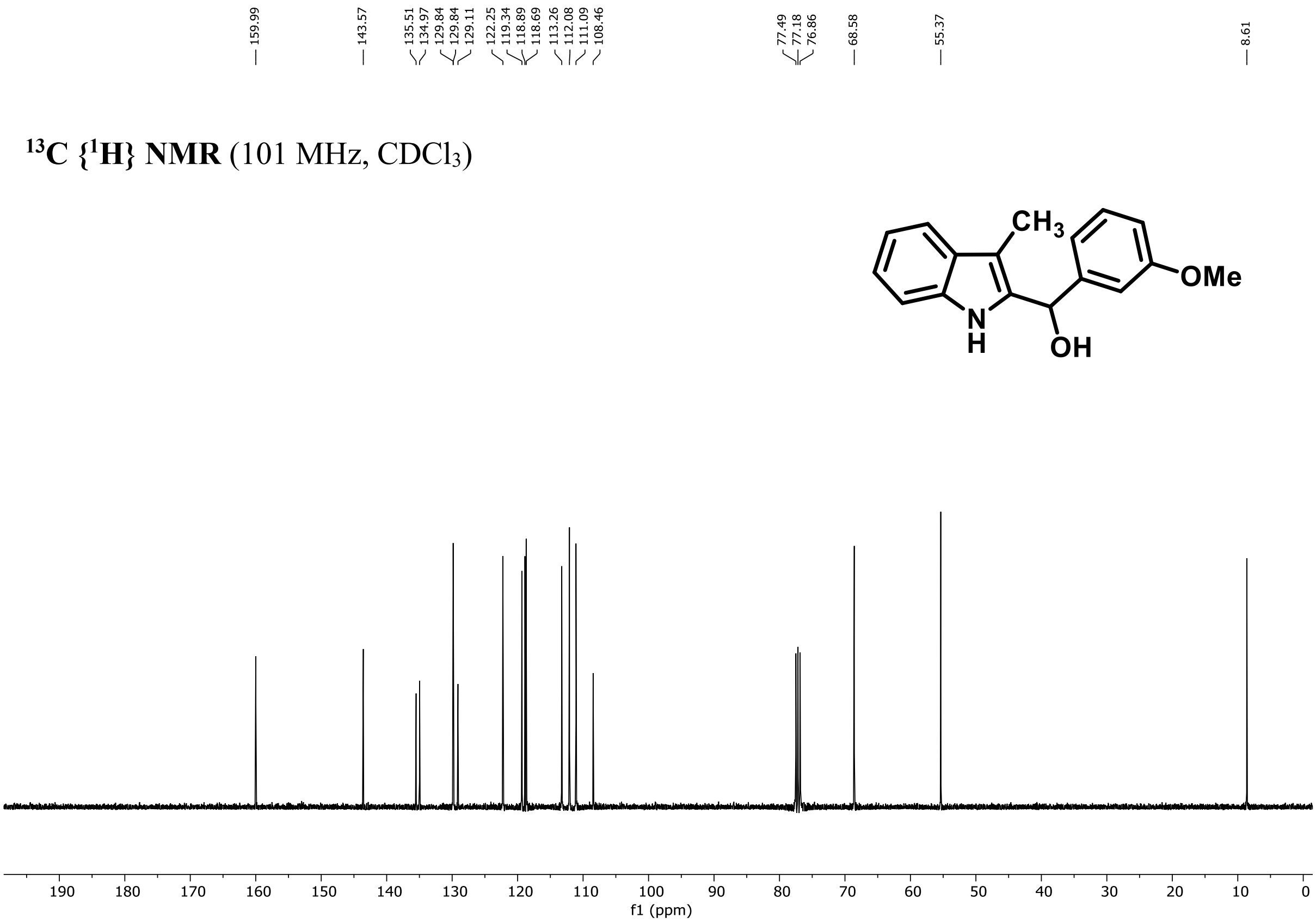

(2-Methoxyphenyl)(3-methyl-1*H*-indol-2-yl)methanol (2g):

^{1}H NMR (400 MHz, $CDCl_3$)

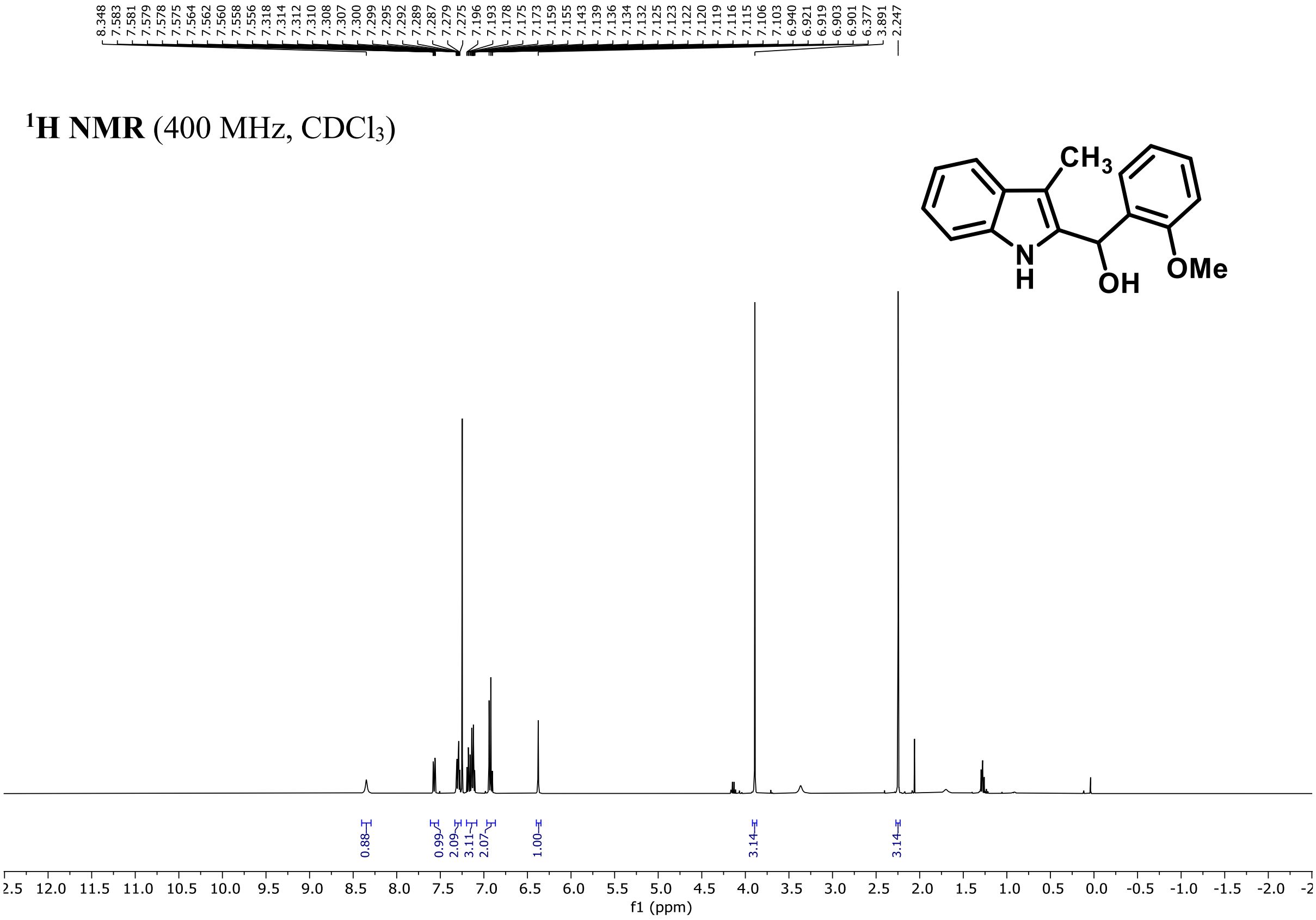

^{13}C {^{1}H} NMR (101 MHz, $CDCl_3$)

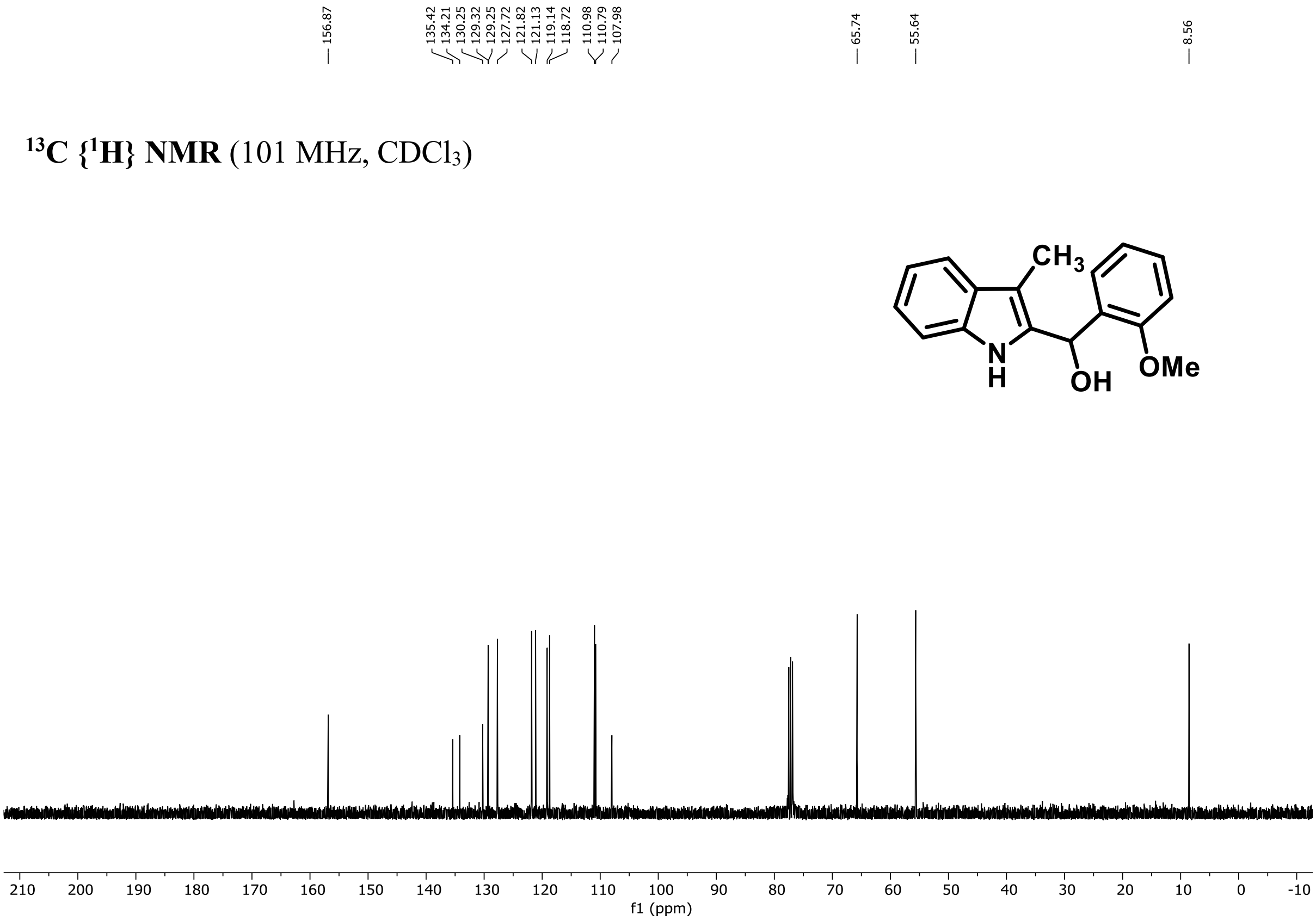

(4-Fluorophenyl)(3-methyl-1*H*-indol-2-yl)methanol (2h):

^{1}H NMR (400 MHz, $CDCl_3$)

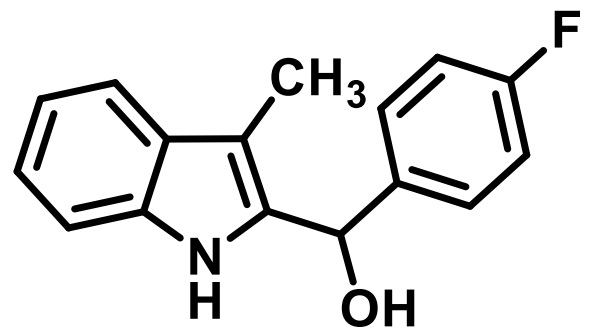

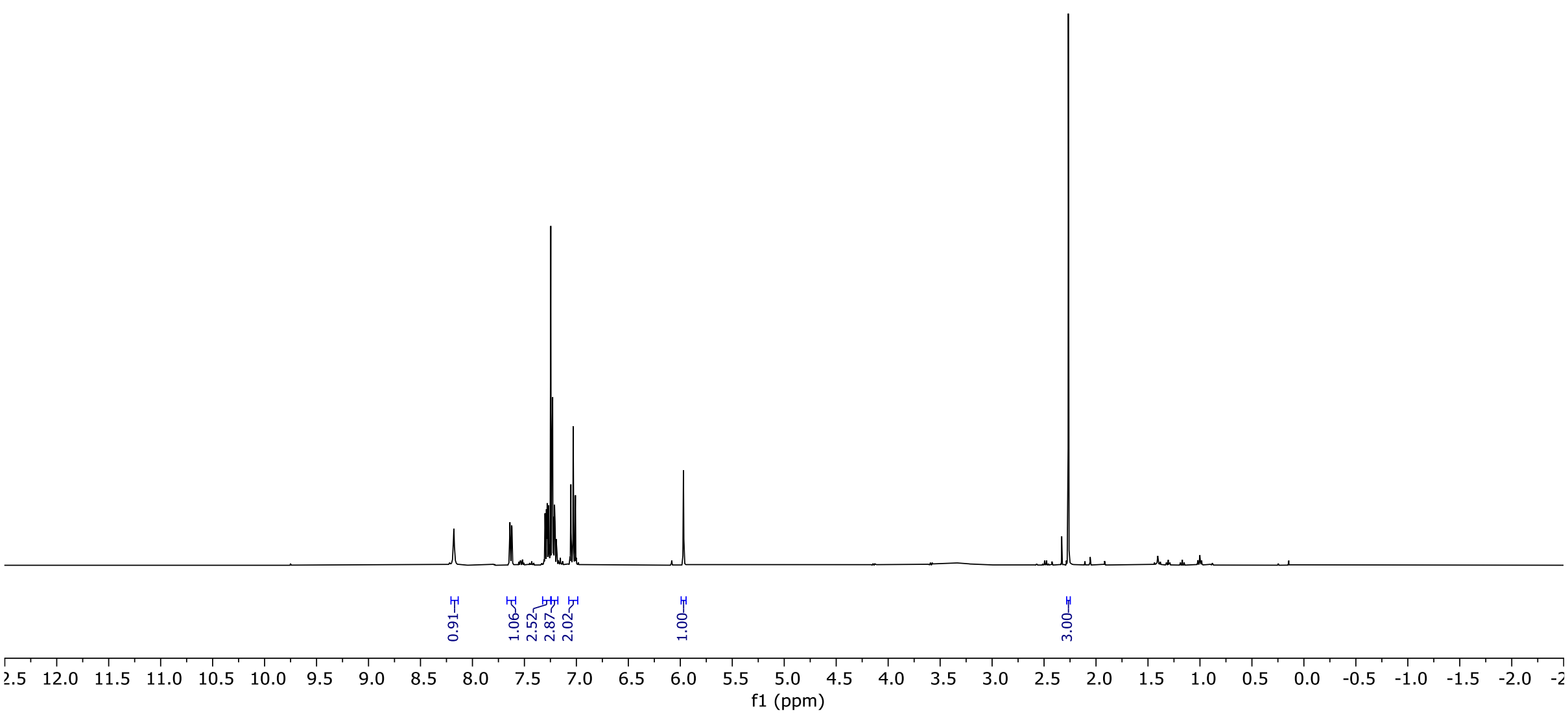

\

^{13}C {^{1}H} NMR (101 MHz, $CDCl_3$)

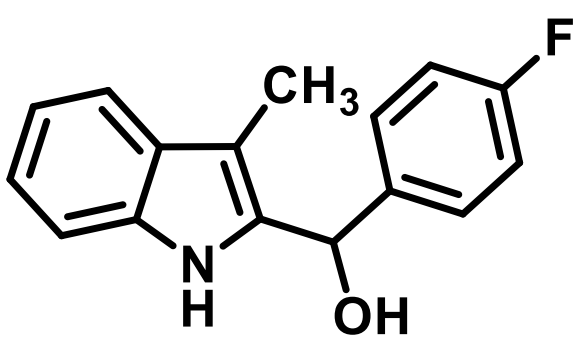

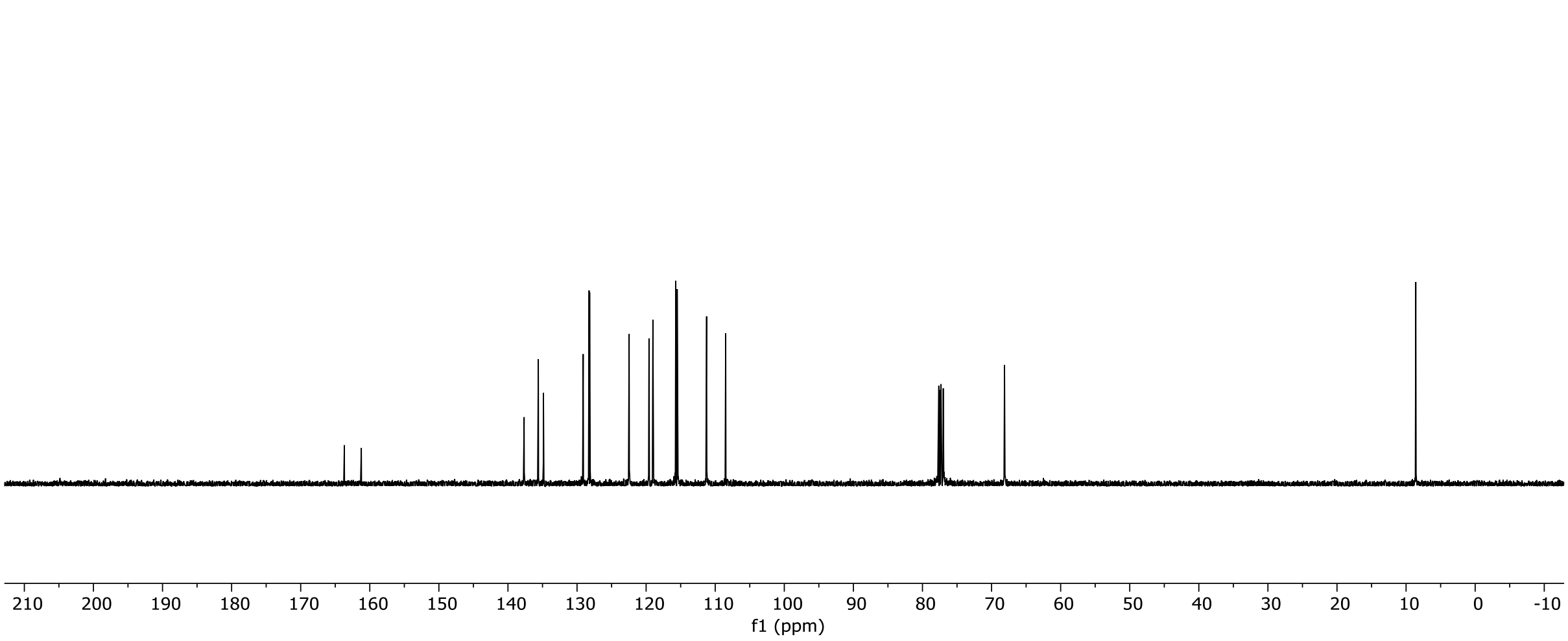

^{19}F NMR (376 MHz, $CDCl_3$)

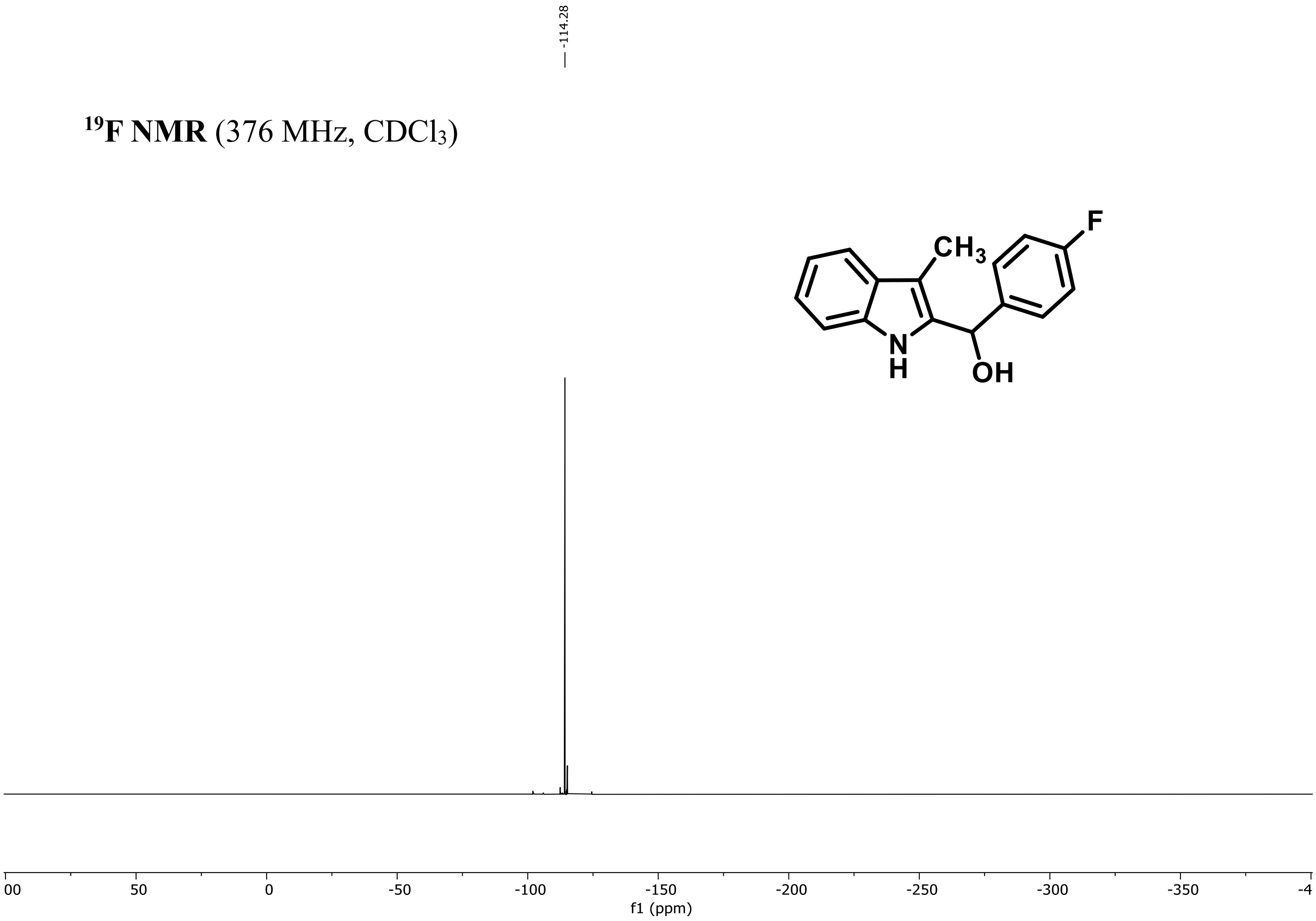

(4-Chlorophenyl)(3-methyl-1*H*-indol-2-yl)methanol (2i):

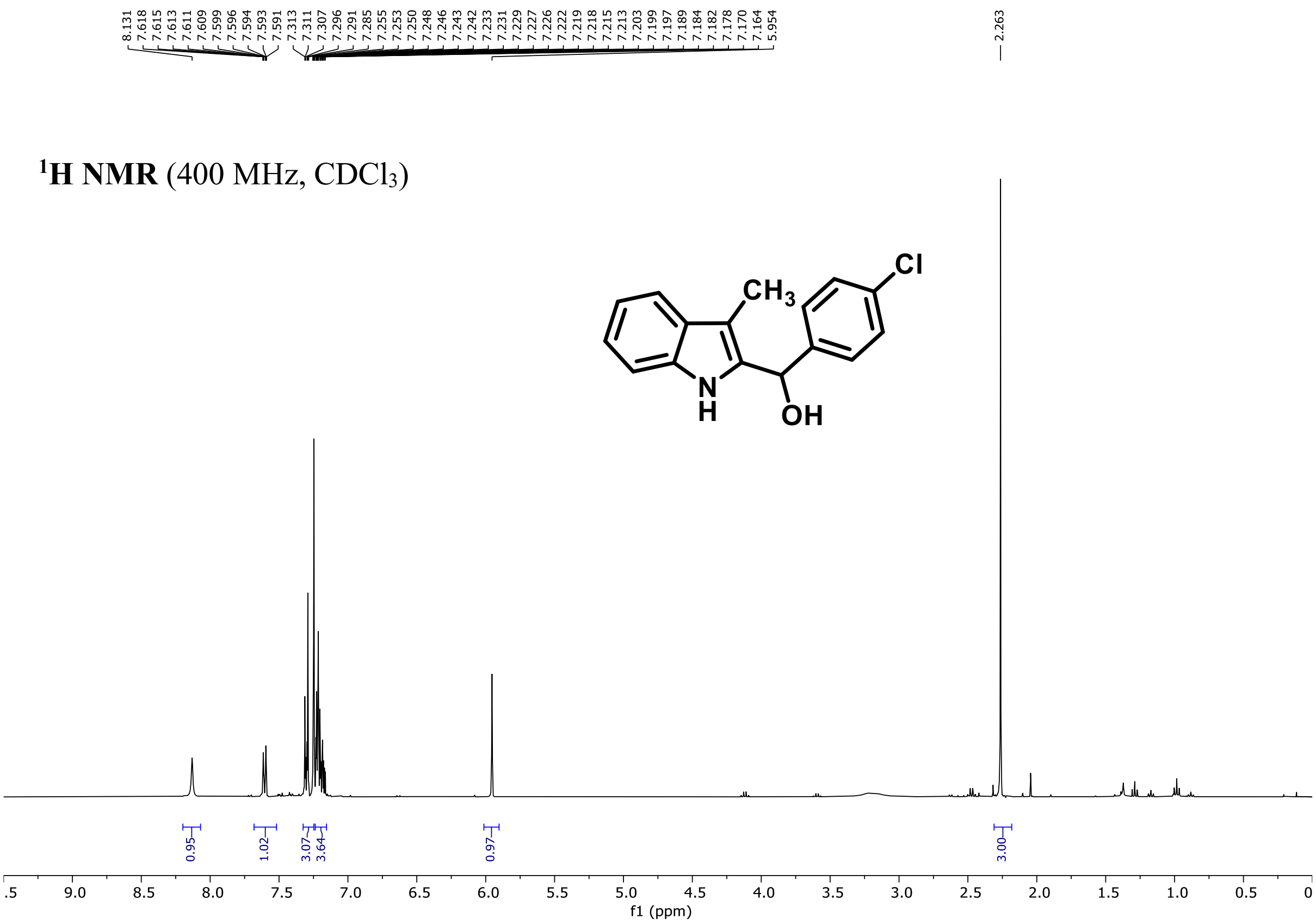

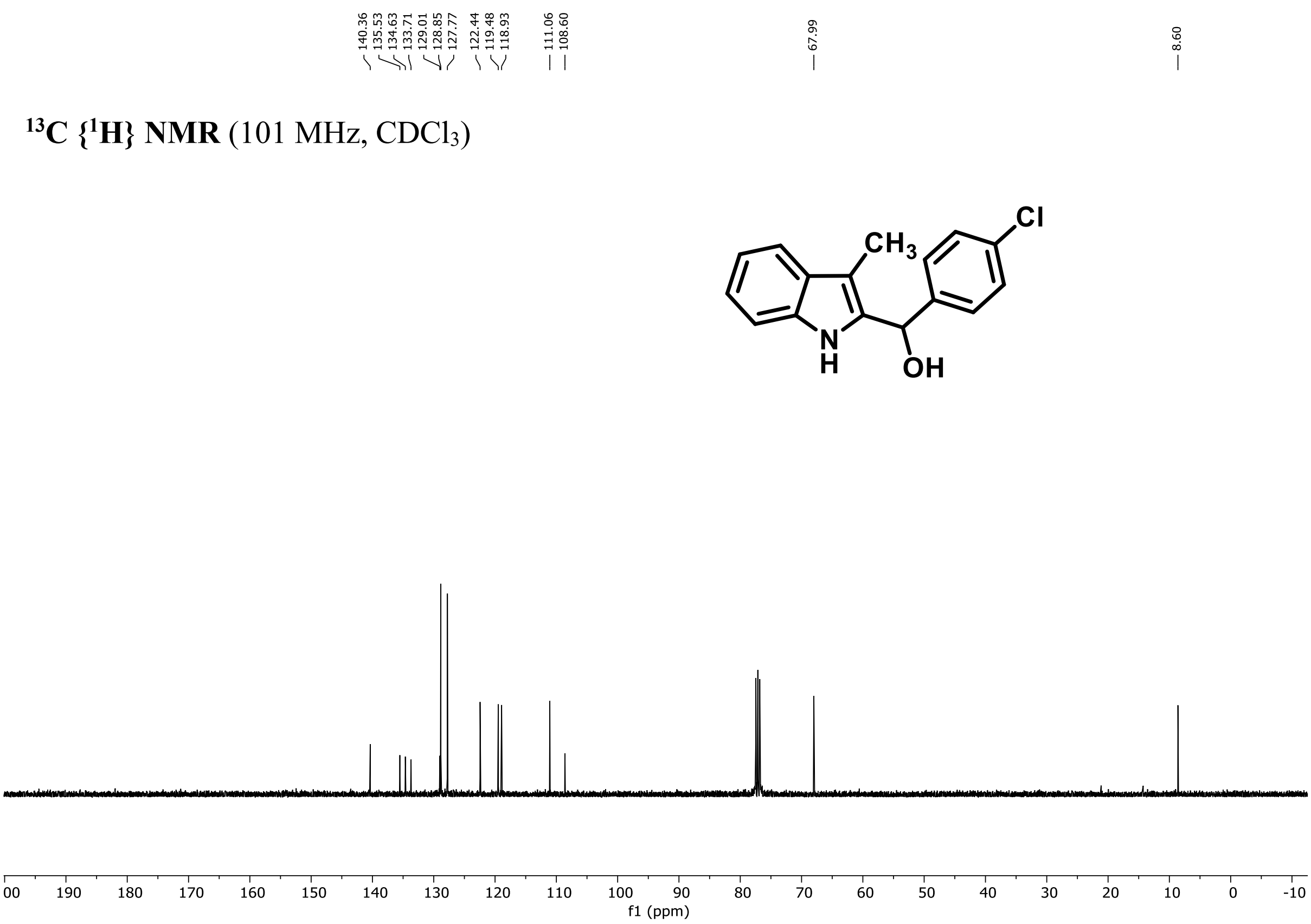

(3,4-dichlorophenyl)(3-methyl-1*H*-indol-2-yl)methanol (2j):

^{1}H NMR (400 MHz, $CDCl_3$)

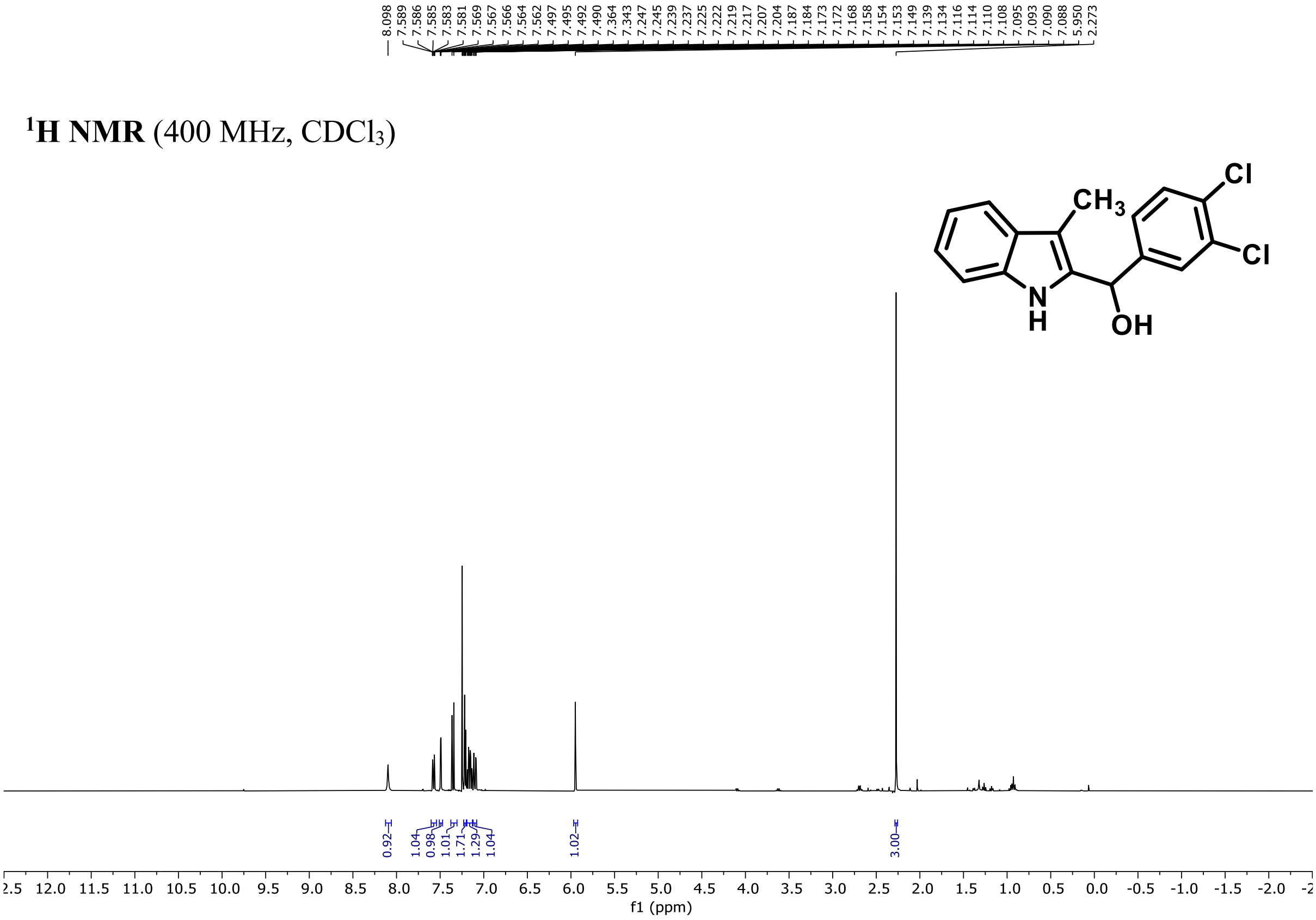

^{13}C {^{1}H} NMR (101 MHz, $CDCl_3$)

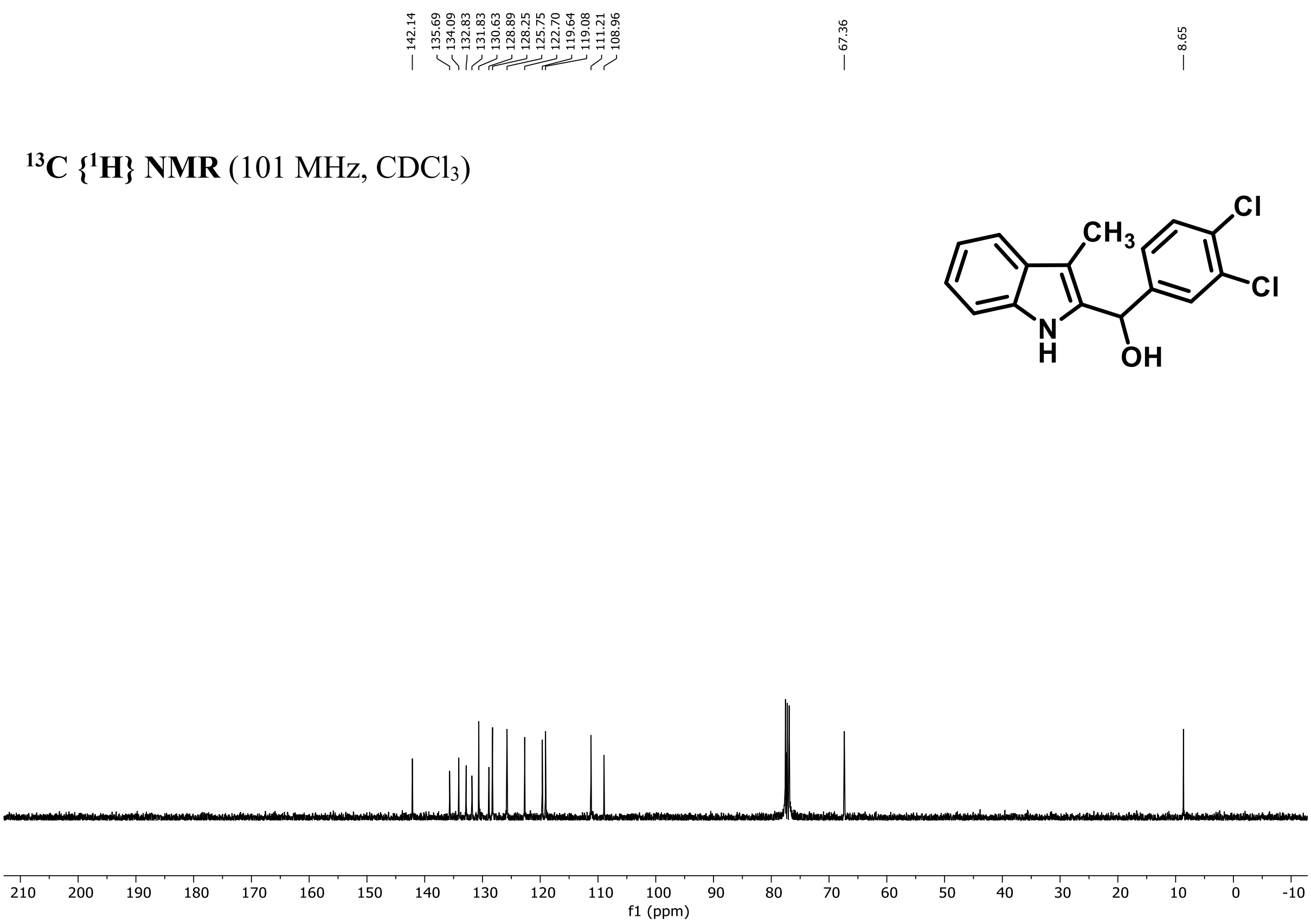

(3-Methyl-1*H*-indol-2-yl)(4-(trifluoromethoxy)phenyl)methanol (2k):

^{1}H NMR (400 MHz, $CDCl_3$)

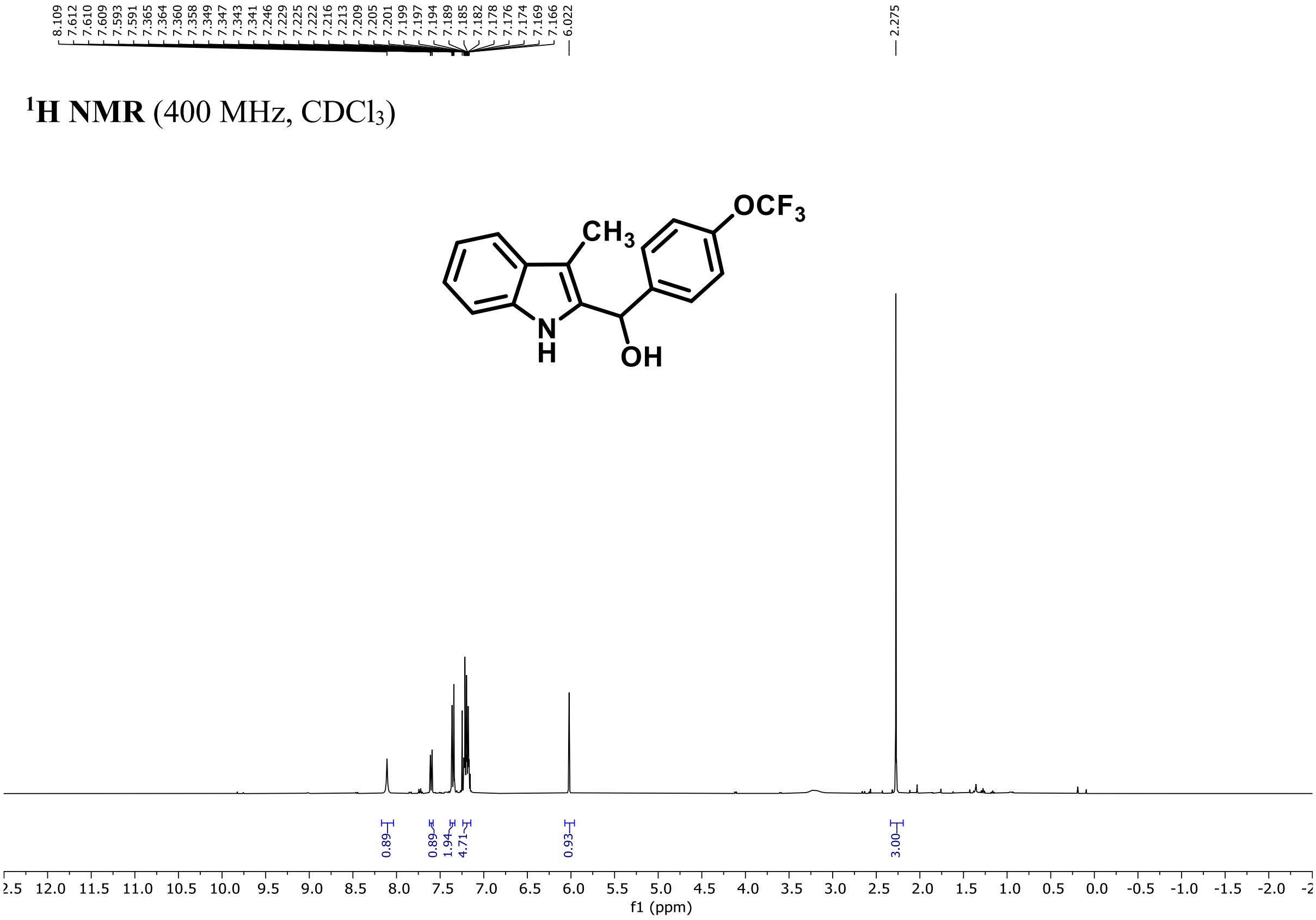

^{13}C {^{1}H} NMR (101 MHz, $CDCl_3$)

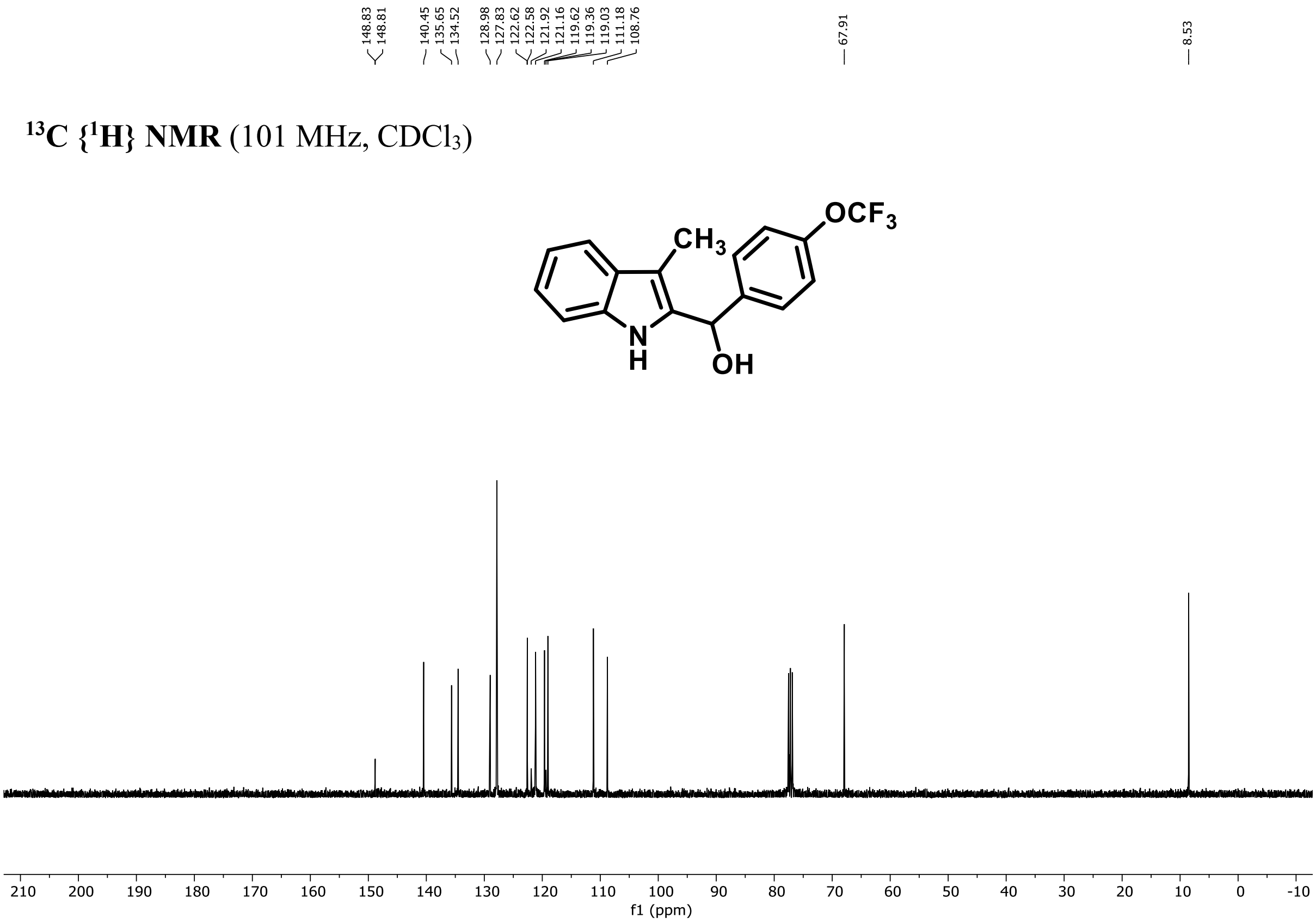

^{19}F NMR (376 MHz, $CDCl_3$)

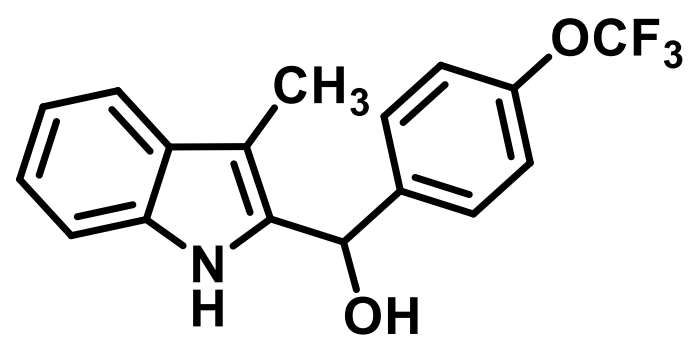

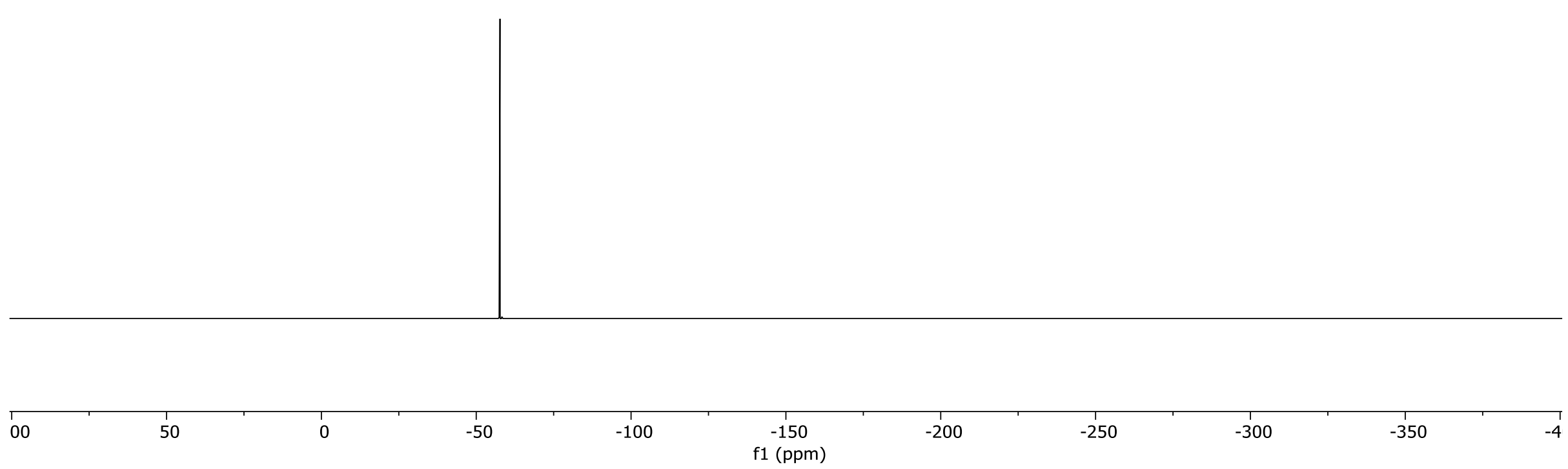

(3-Methyl-1*H*-indol-2-yl)(4-(trifluoromethyl)phenyl)methanol (2l):

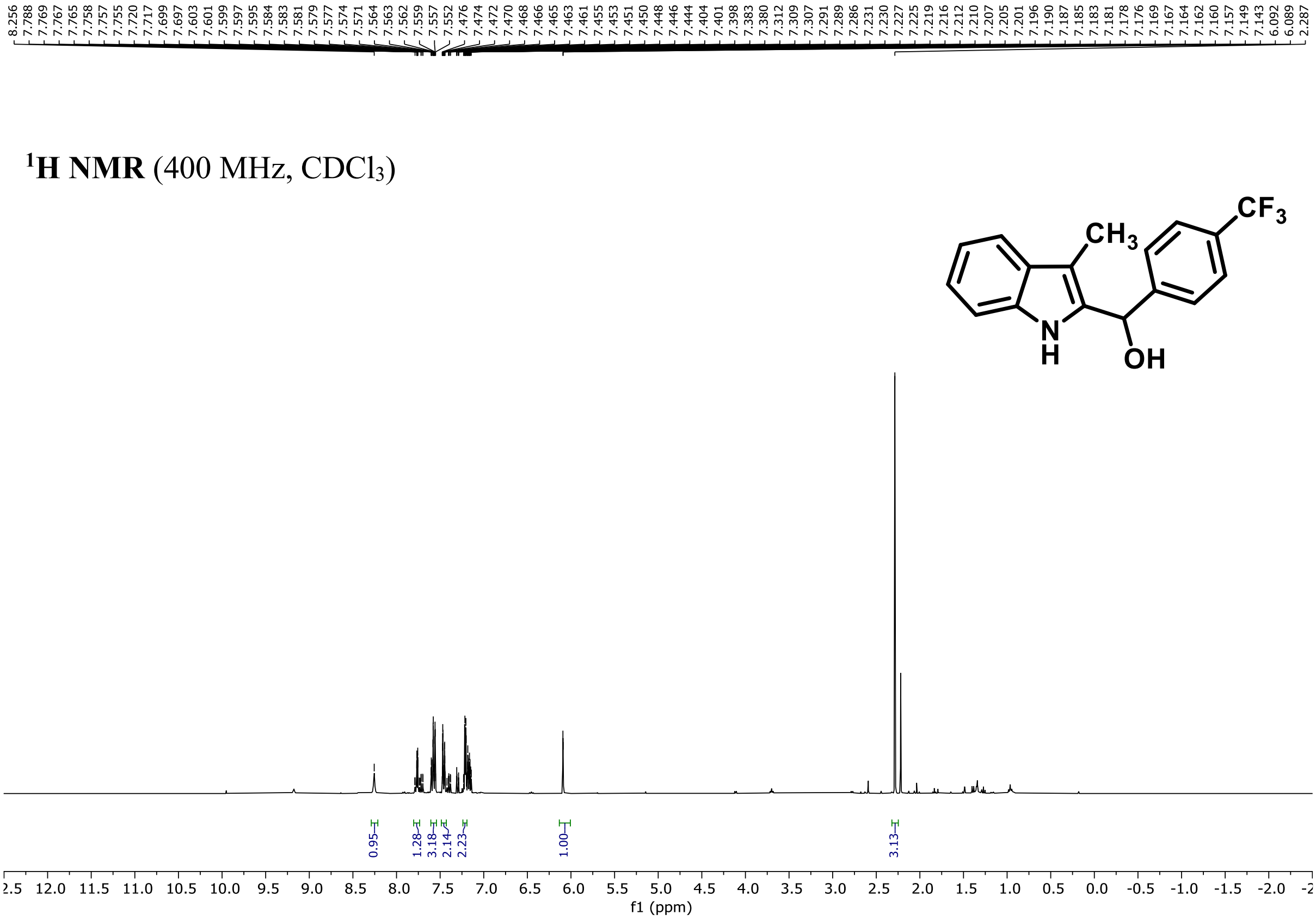

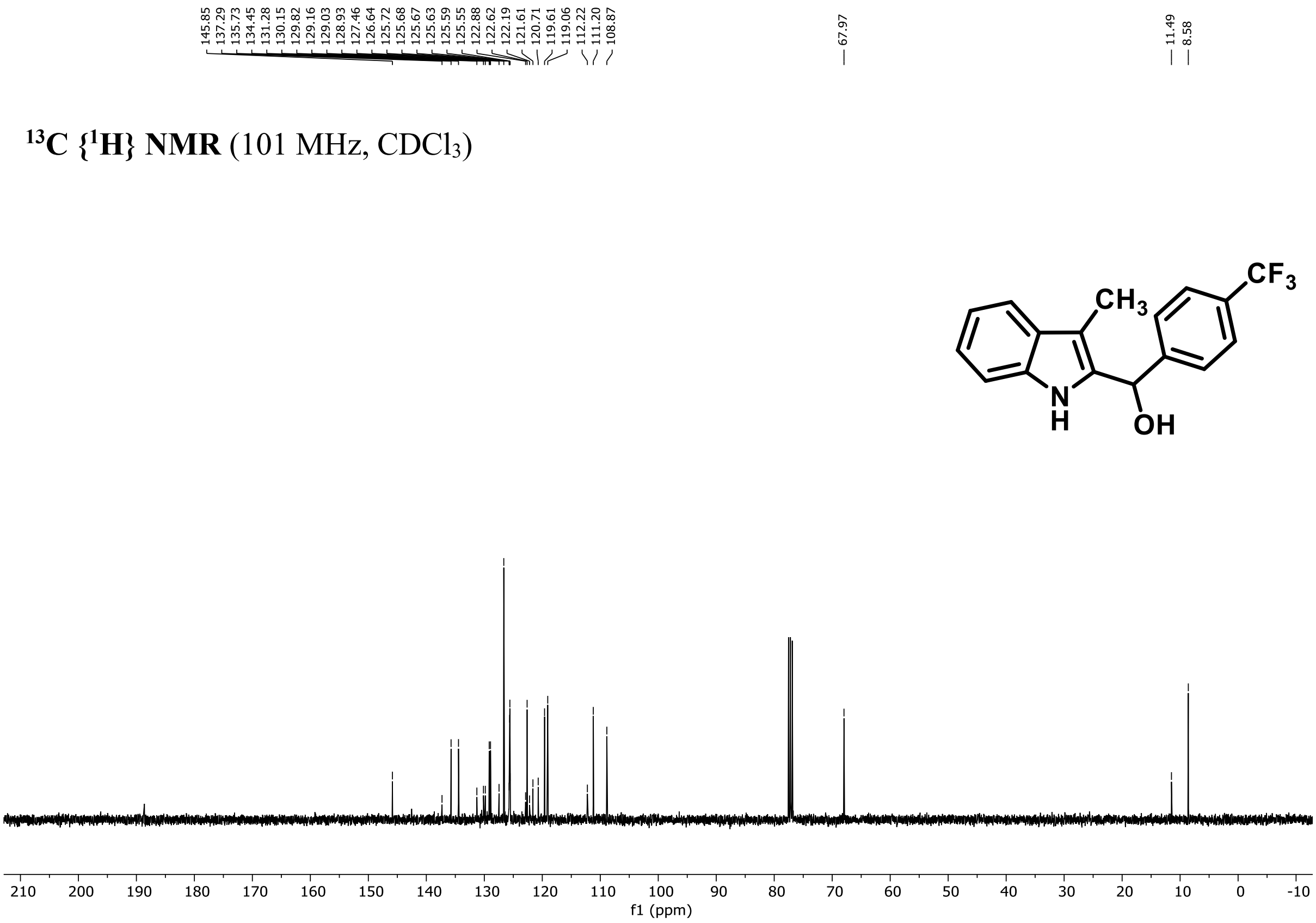

19F NMR (376 MHz, $CDCl_3$)

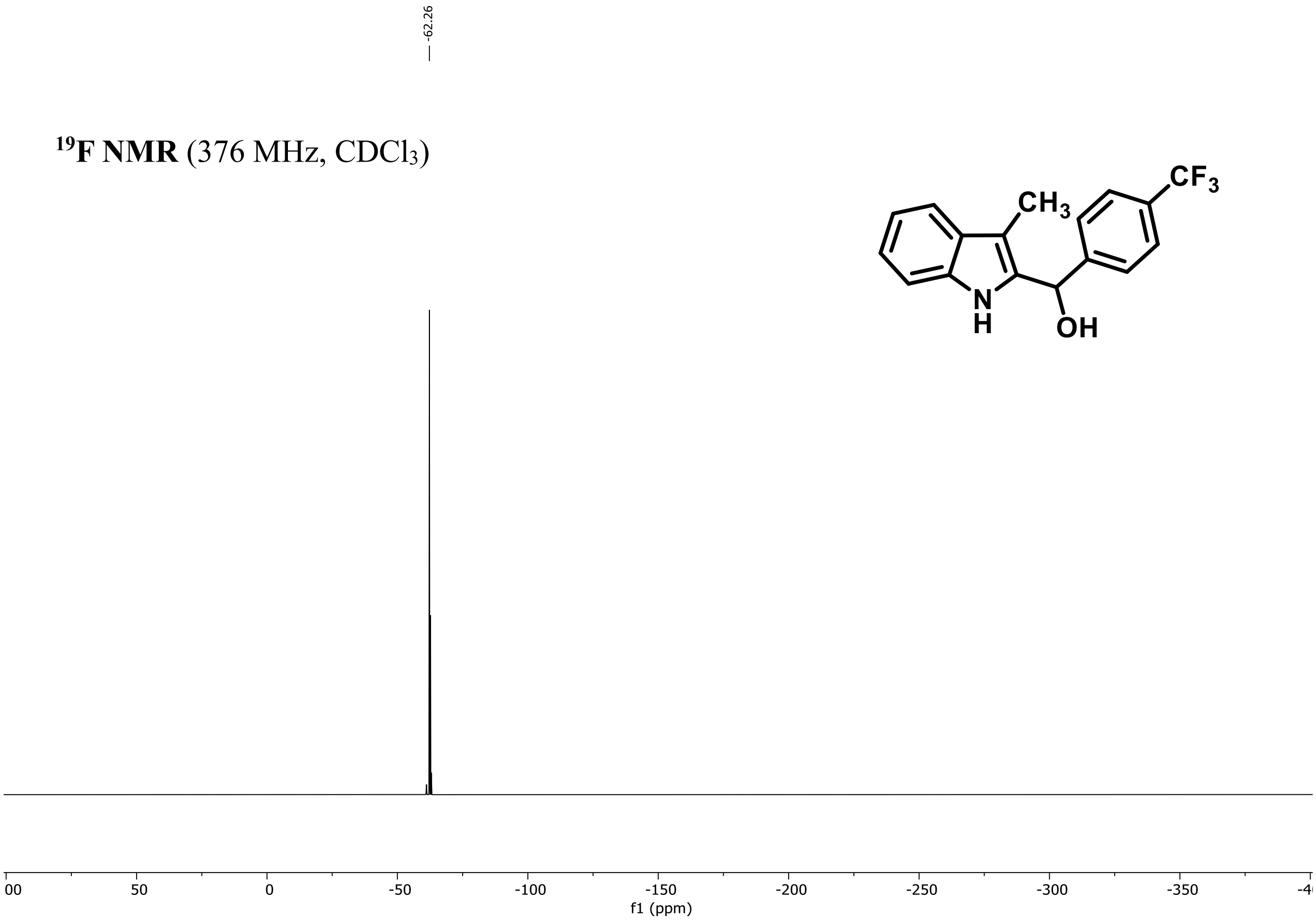

(3-Methyl-1*H*-indol-2-yl)(thiophen-2-yl)methanol (2m):

^{1}H NMR (400 MHz, $CDCl_3$)

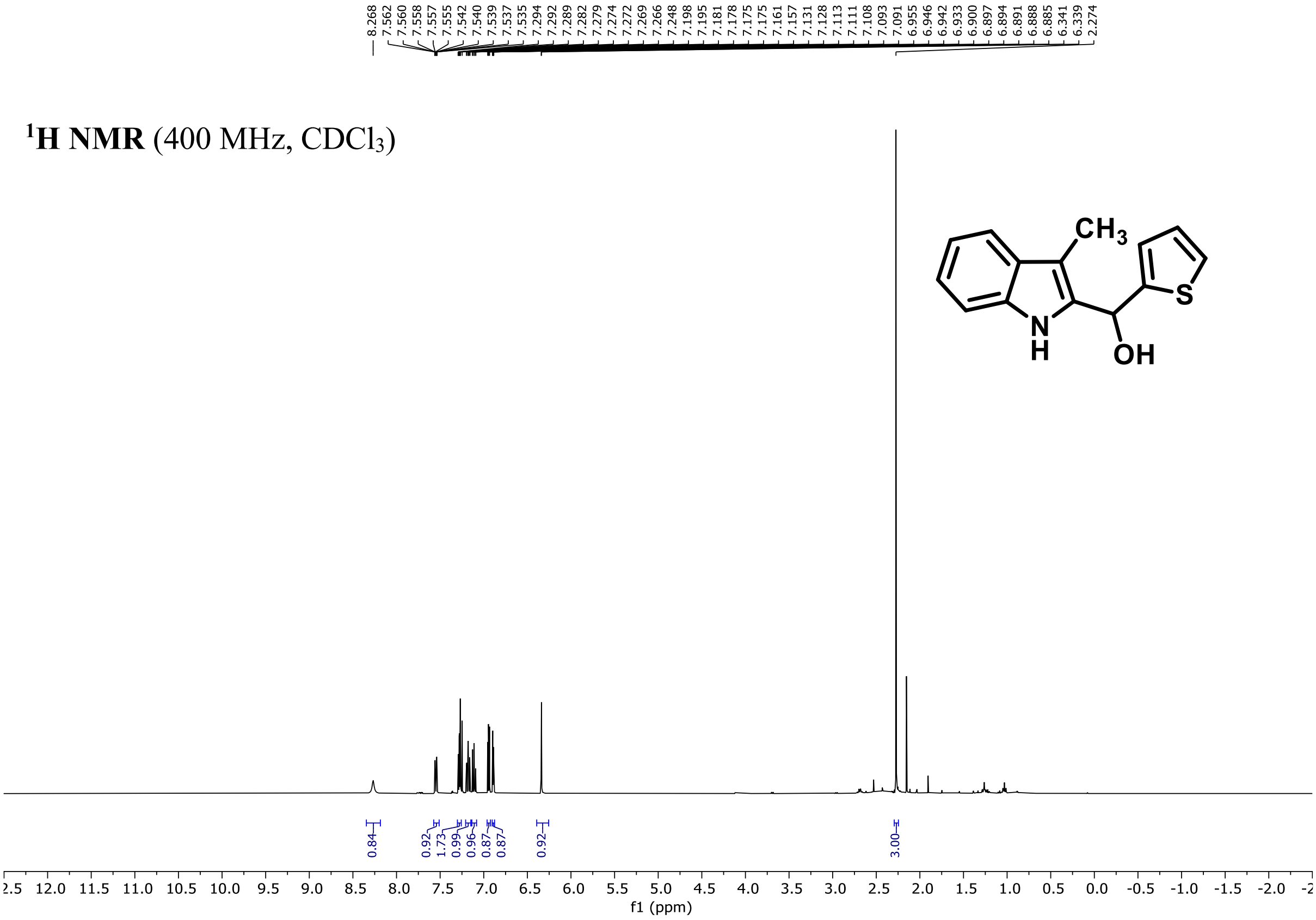

^{13}C {^{1}H} NMR (101 MHz, $CDCl_3$)

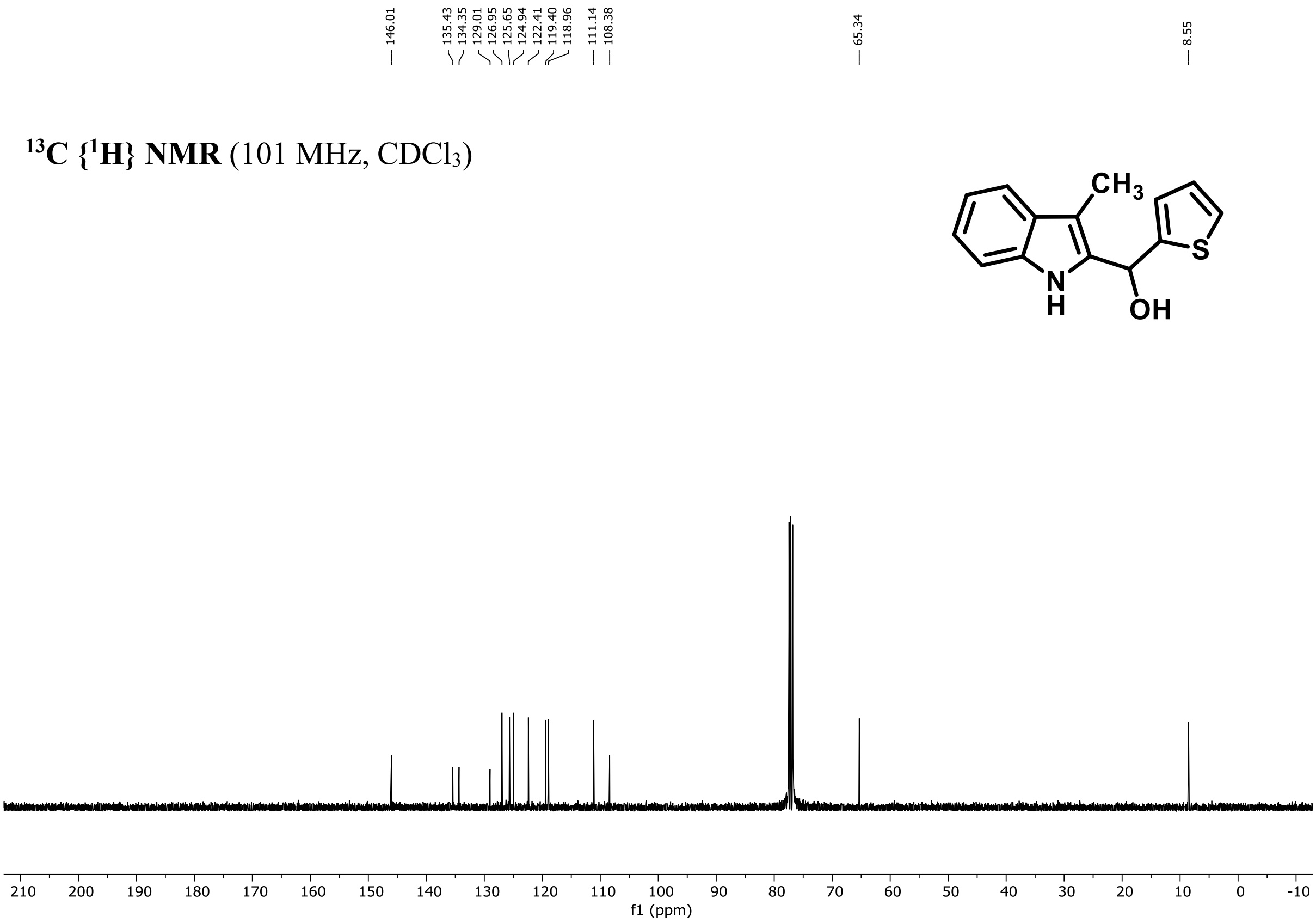

1-(3-Methyl-1*H*-indol-2-yl)ethan-1-ol (2n):

^{1}H NMR (400 MHz, $CDCl_3$)

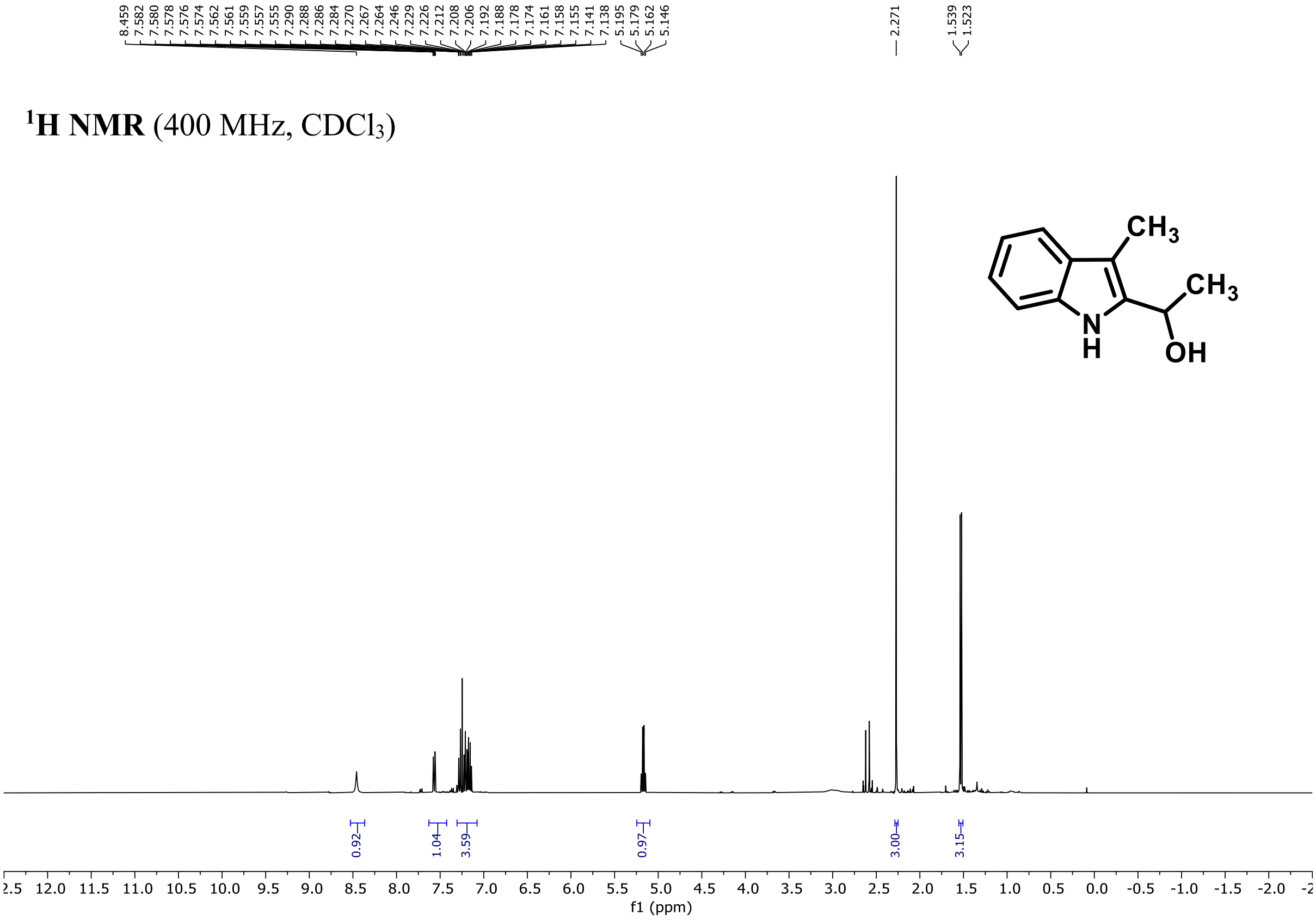

^{13}C {^{1}H} NMR (101 MHz, $CDCl_3$)

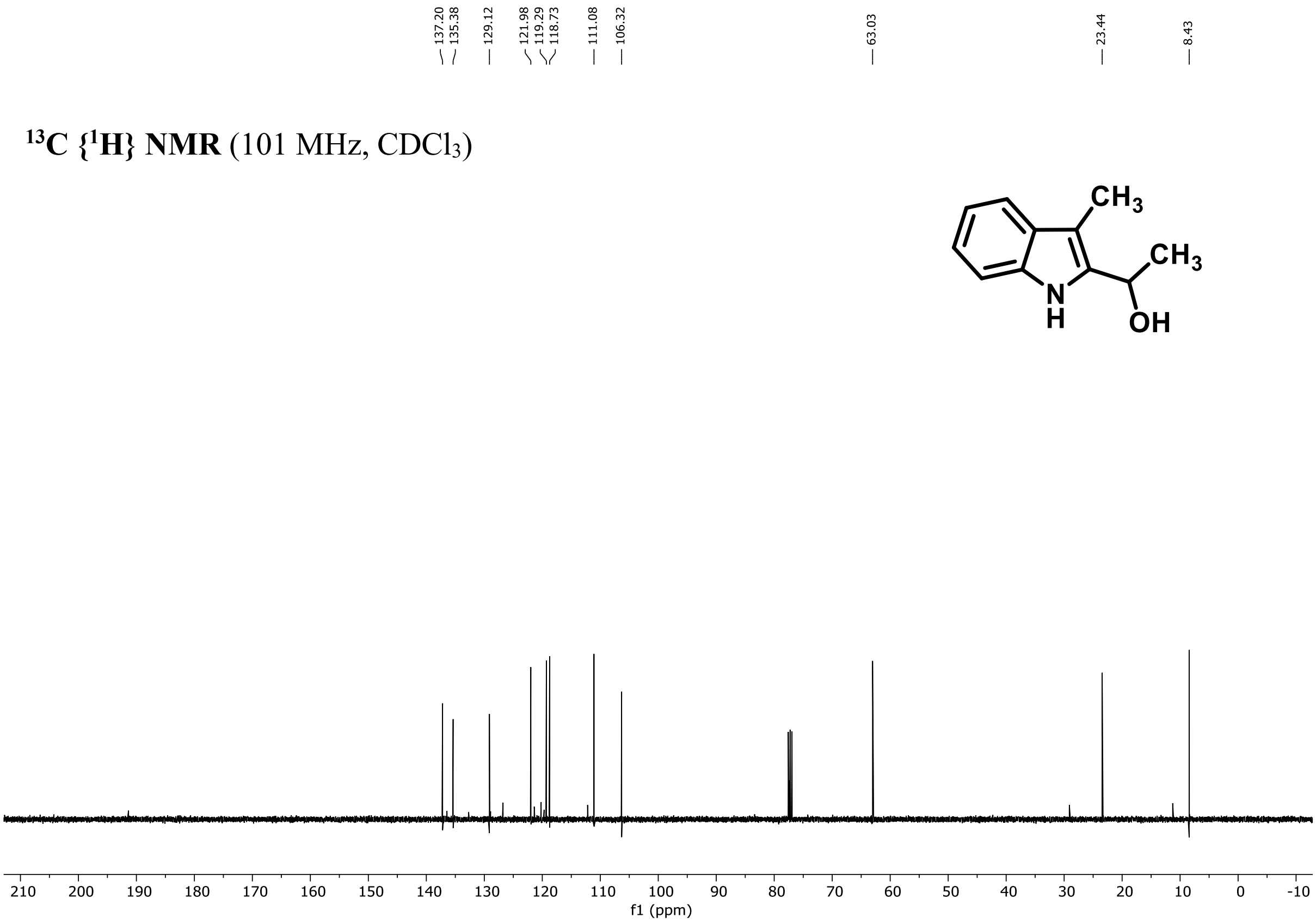

1-(3-Methyl-1*H*-indol-2-yl)but-3-en-1-ol (2o):

^{1}H NMR (400 MHz, $CDCl_3$)

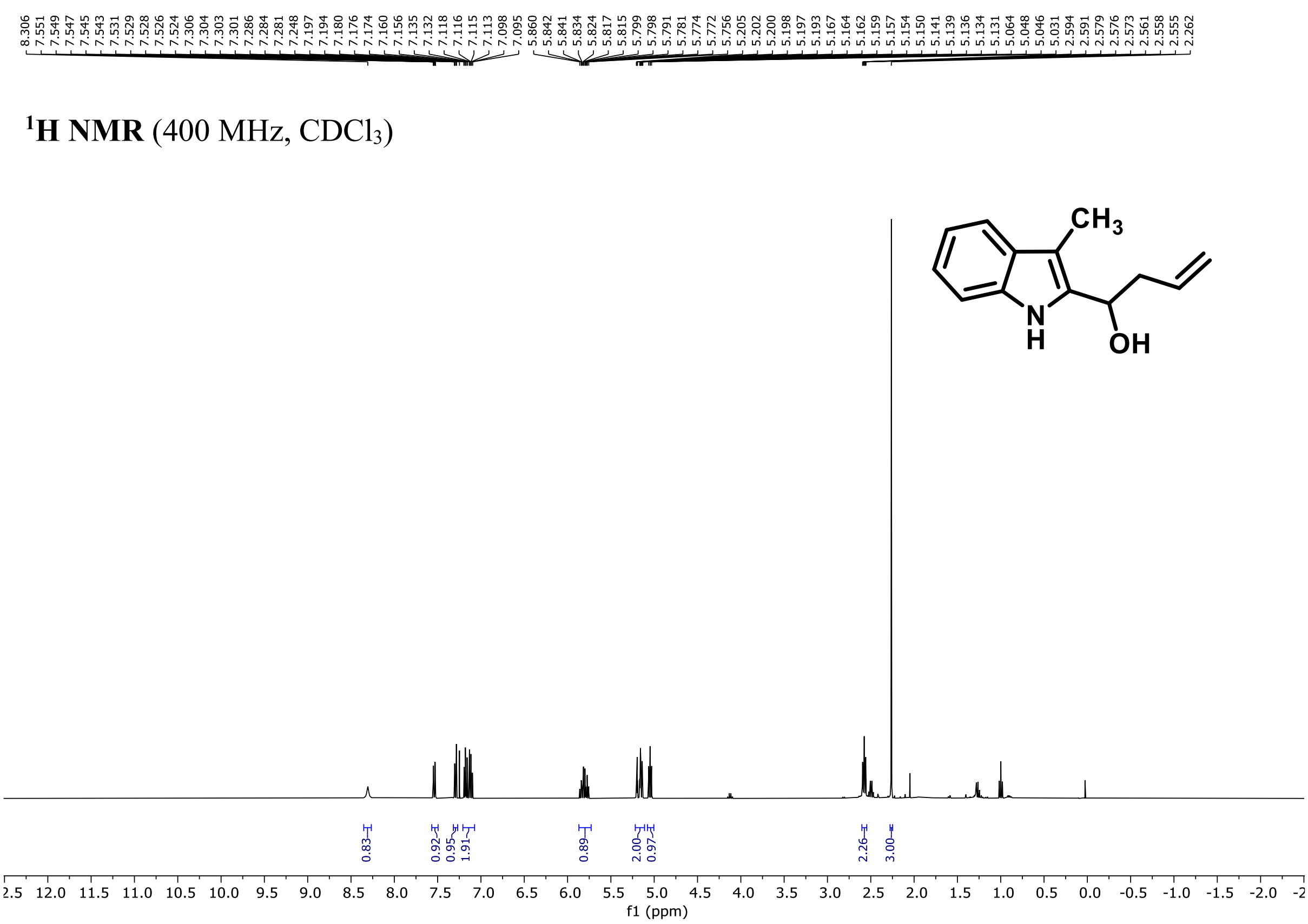

^{13}C {^{1}H} NMR (101 MHz, $CDCl_3$)

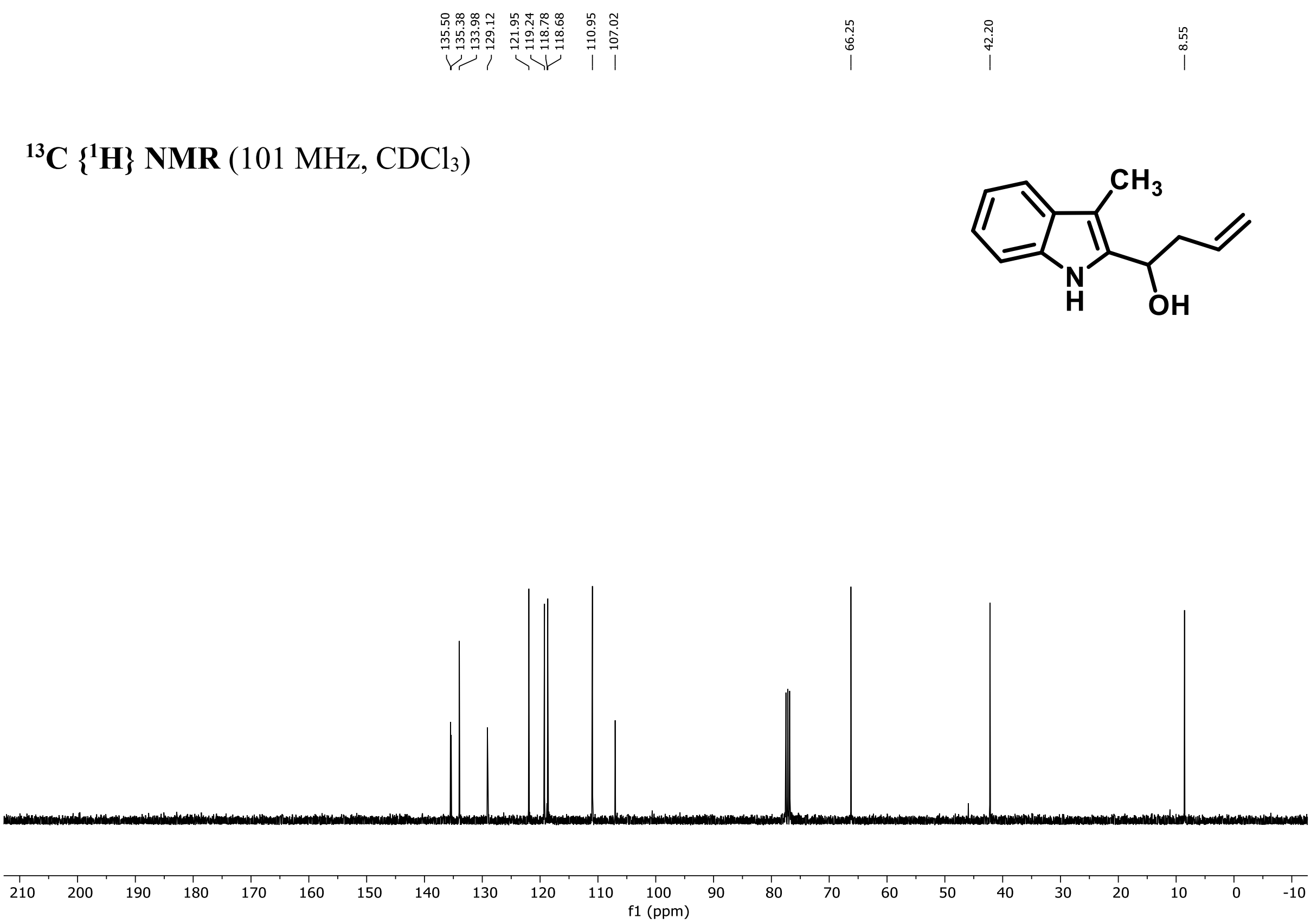

Phenyl(3-phenyl-1*H*-indol-2-yl)methanol (2p):

^{1}H NMR (400 MHz, $CDCl_3$)

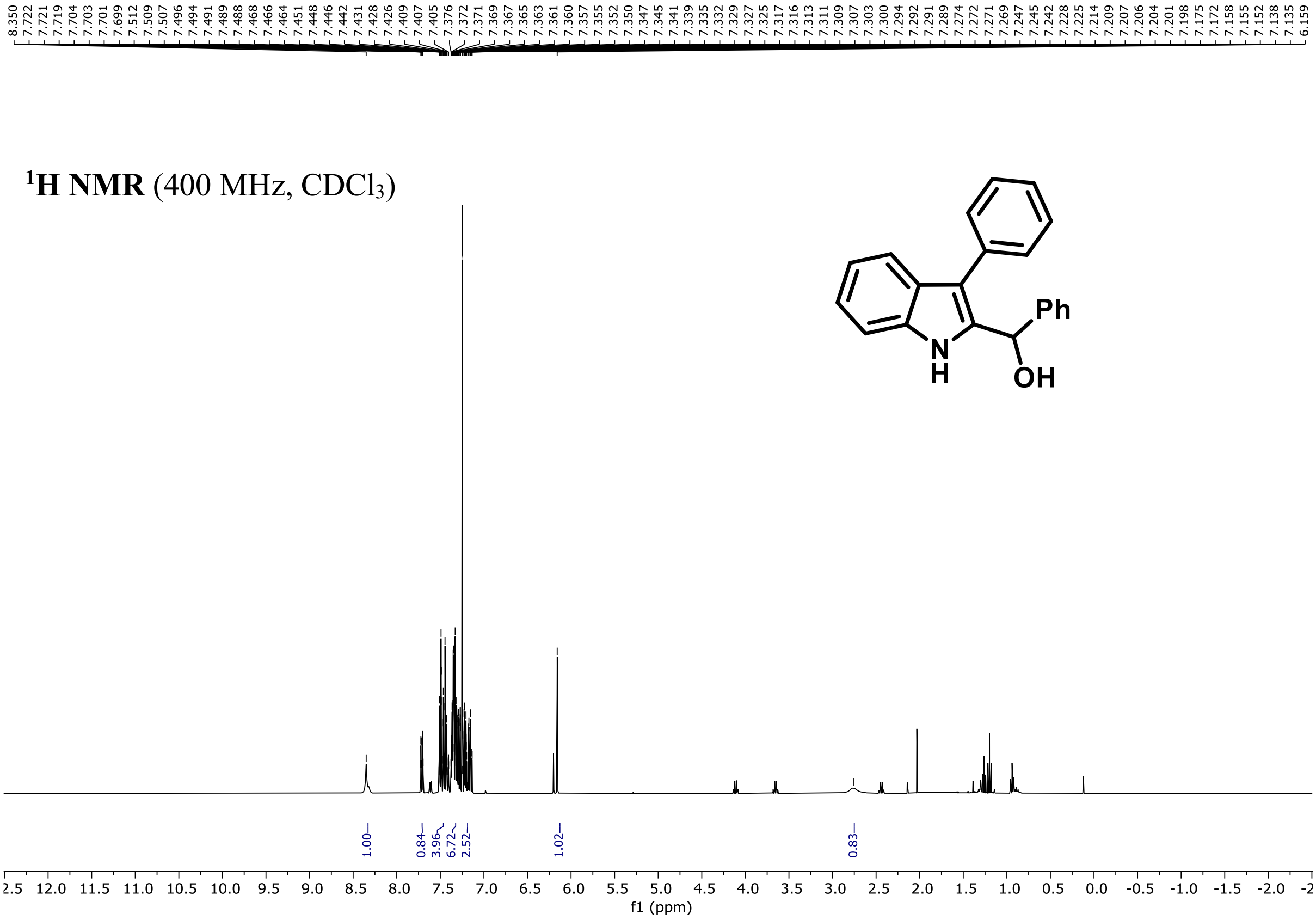

^{13}C {^{1}H} NMR (101 MHz, $CDCl_3$)

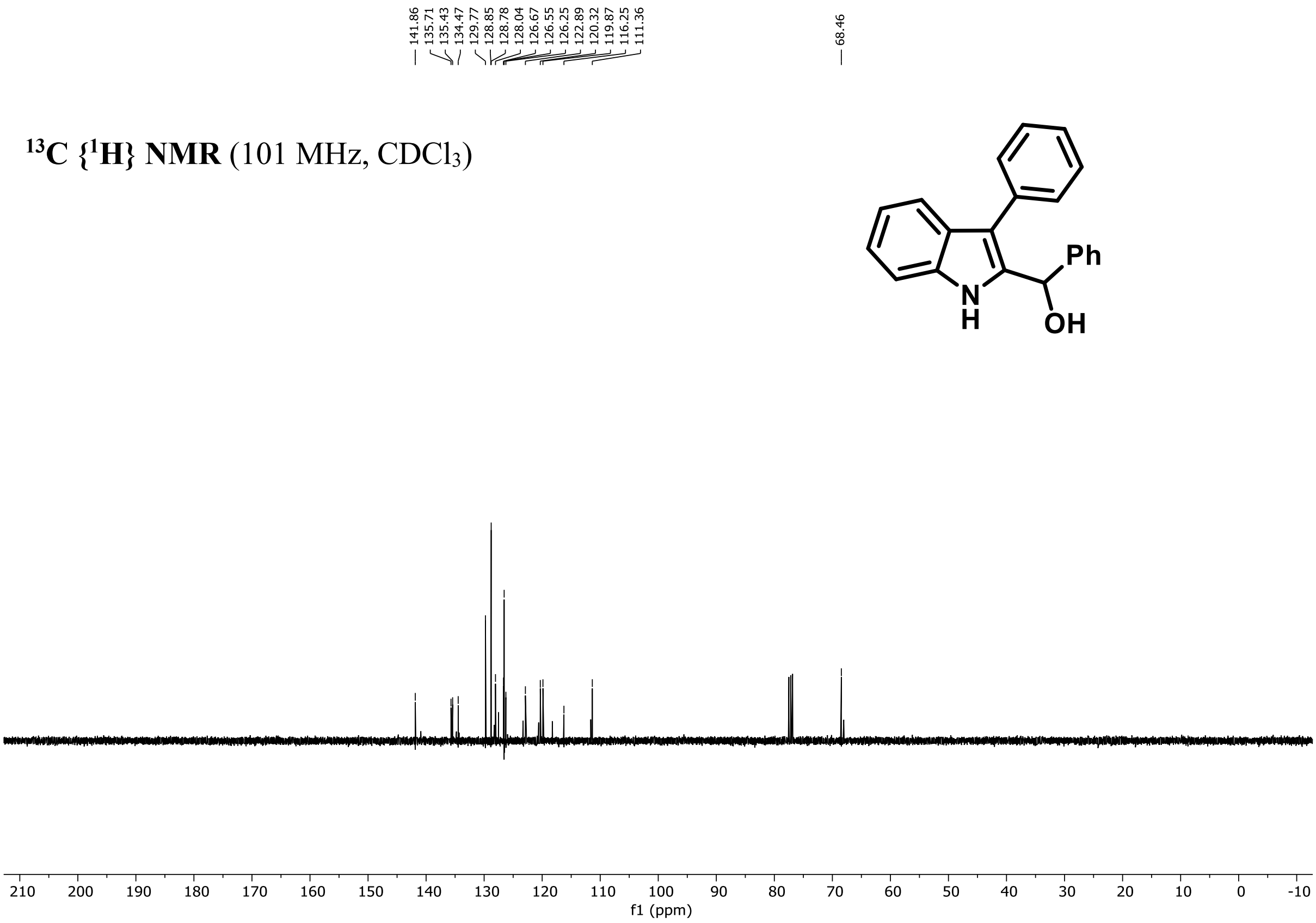

(3-(4-Methoxyphenyl)-1*H*-indol-2-yl)(phenyl)methanol (2q):

^{1}H NMR (400 MHz, $CDCl_3$)

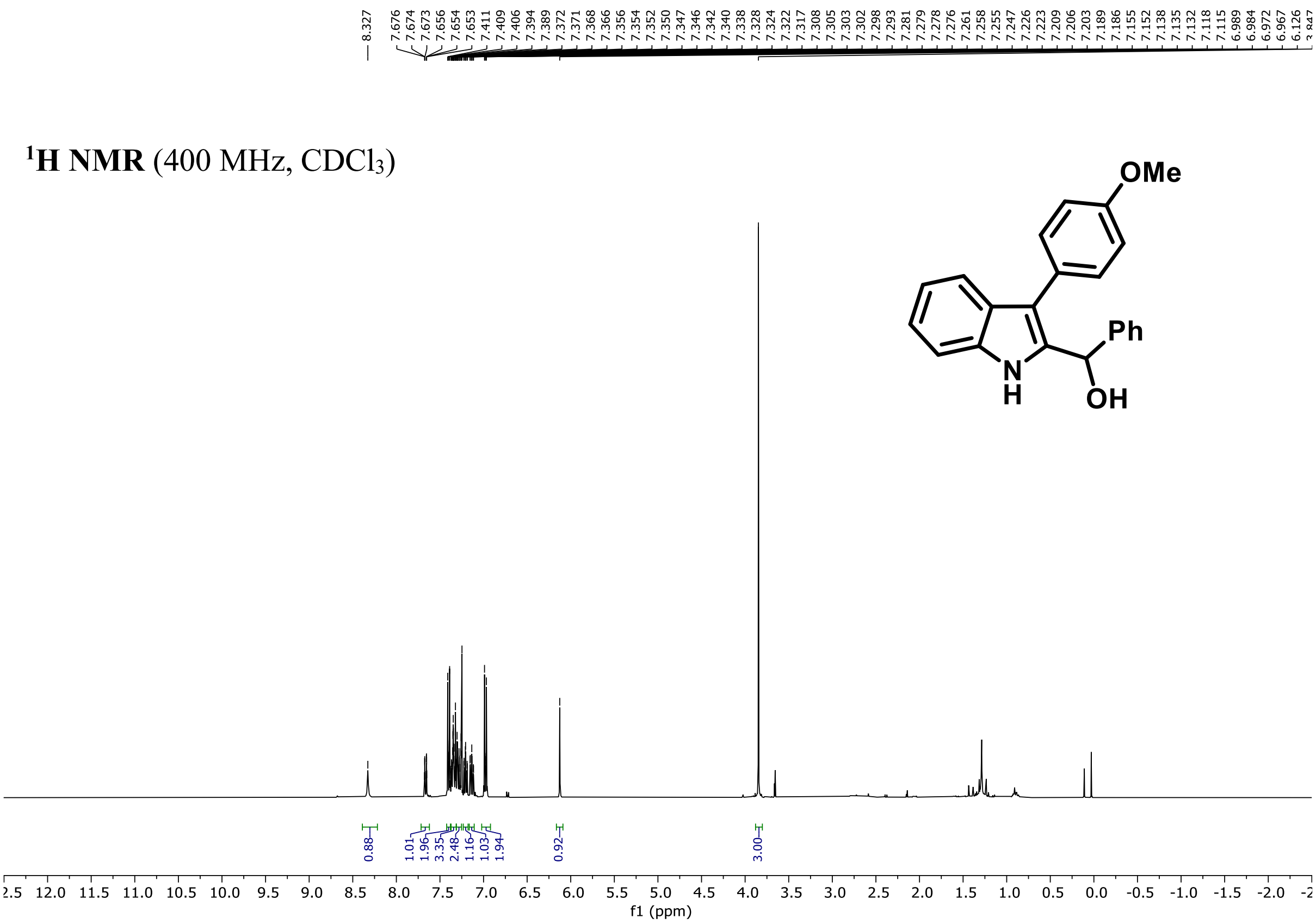

^{13}C {^{1}H} NMR (101 MHz, $CDCl_3$)

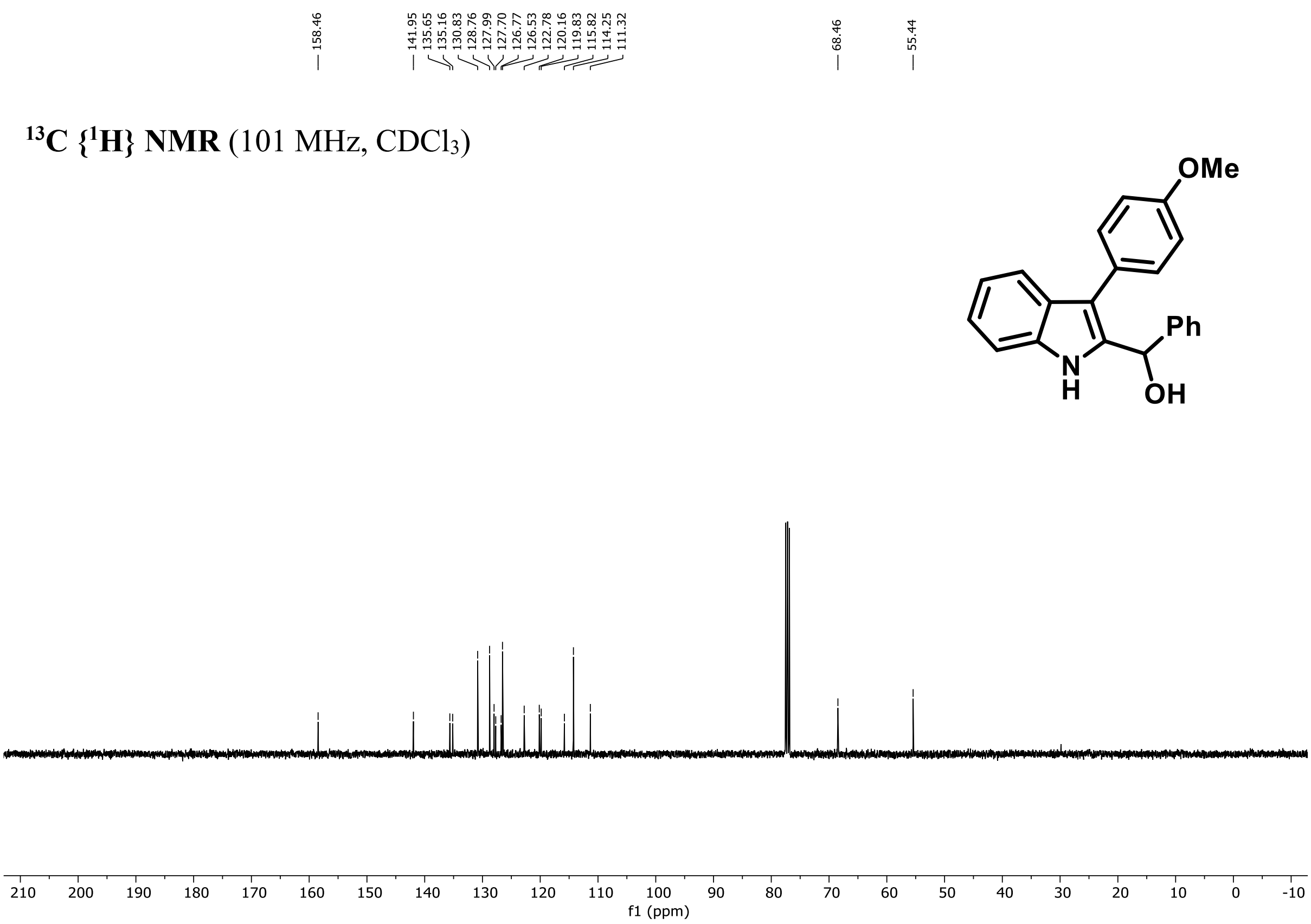

(3-(4-Fluorophenyl)-1*H*-indol-2-yl)(phenyl)methanol (2r):

^{1}H NMR (400 MHz, $CDCl_3$)

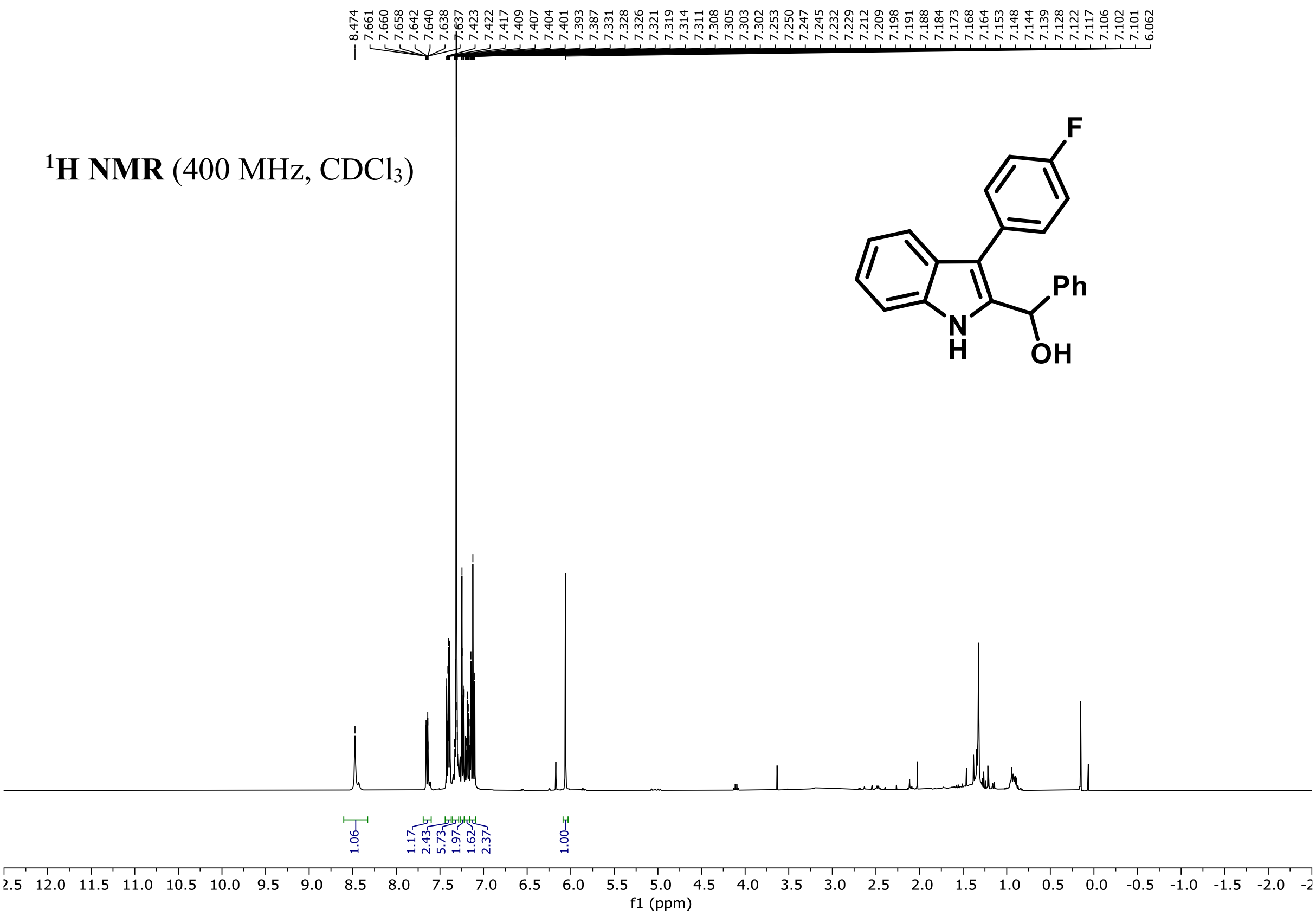

^{13}C {^{1}H} NMR (101 MHz, $CDCl_3$)

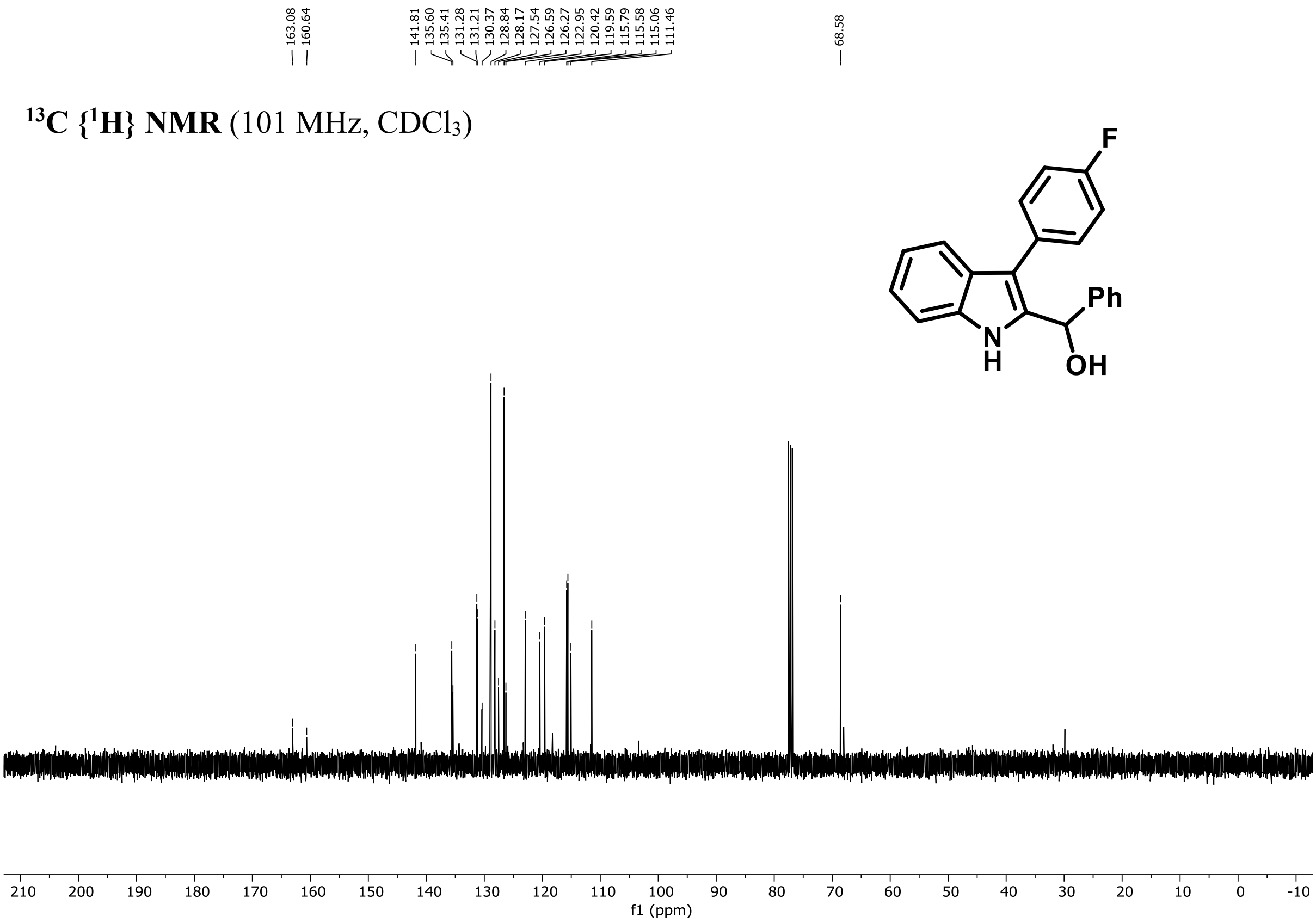

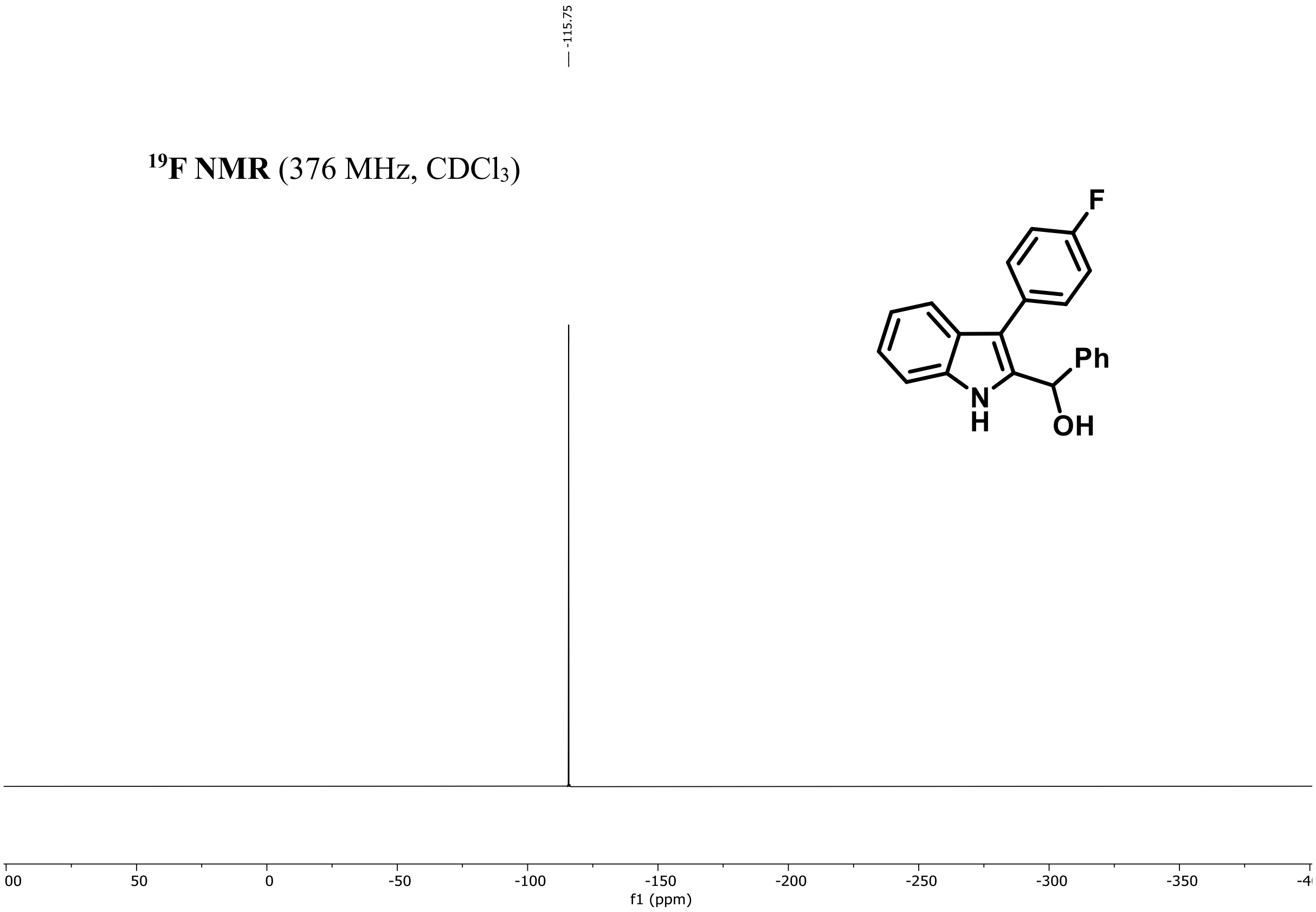

^{19}F NMR (376 MHz, $CDCl_3$)

(3-(4-Chlorophenyl)-1*H*-indol-2-yl)(phenyl)methanol (2s):

^{1}H NMR (400 MHz, $CDCl_3$)

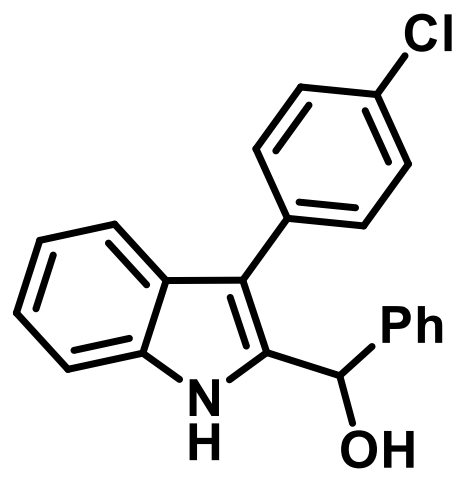

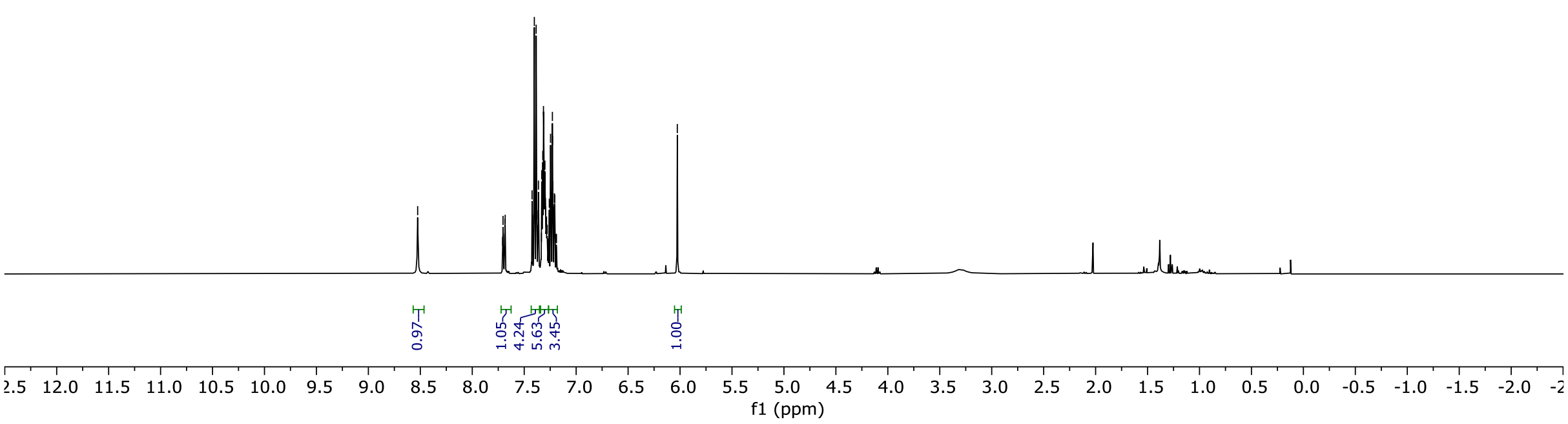

^{13}C {^{1}H} NMR (101 MHz, $CDCl_3$)

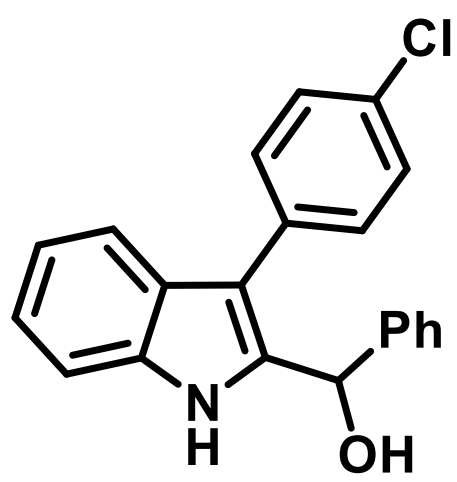

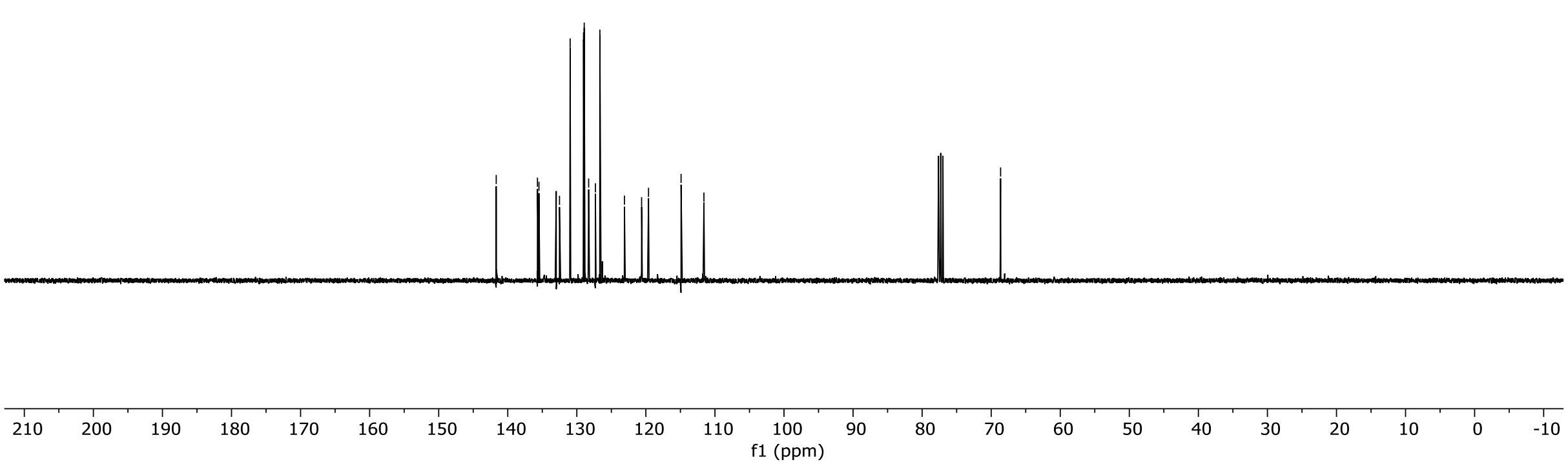

(3-(Naphthalen-2-yl)-1*H*-indol-2-yl)(phenyl)methanol (2t):

^{1}H NMR (400 MHz, $CDCl_3$)

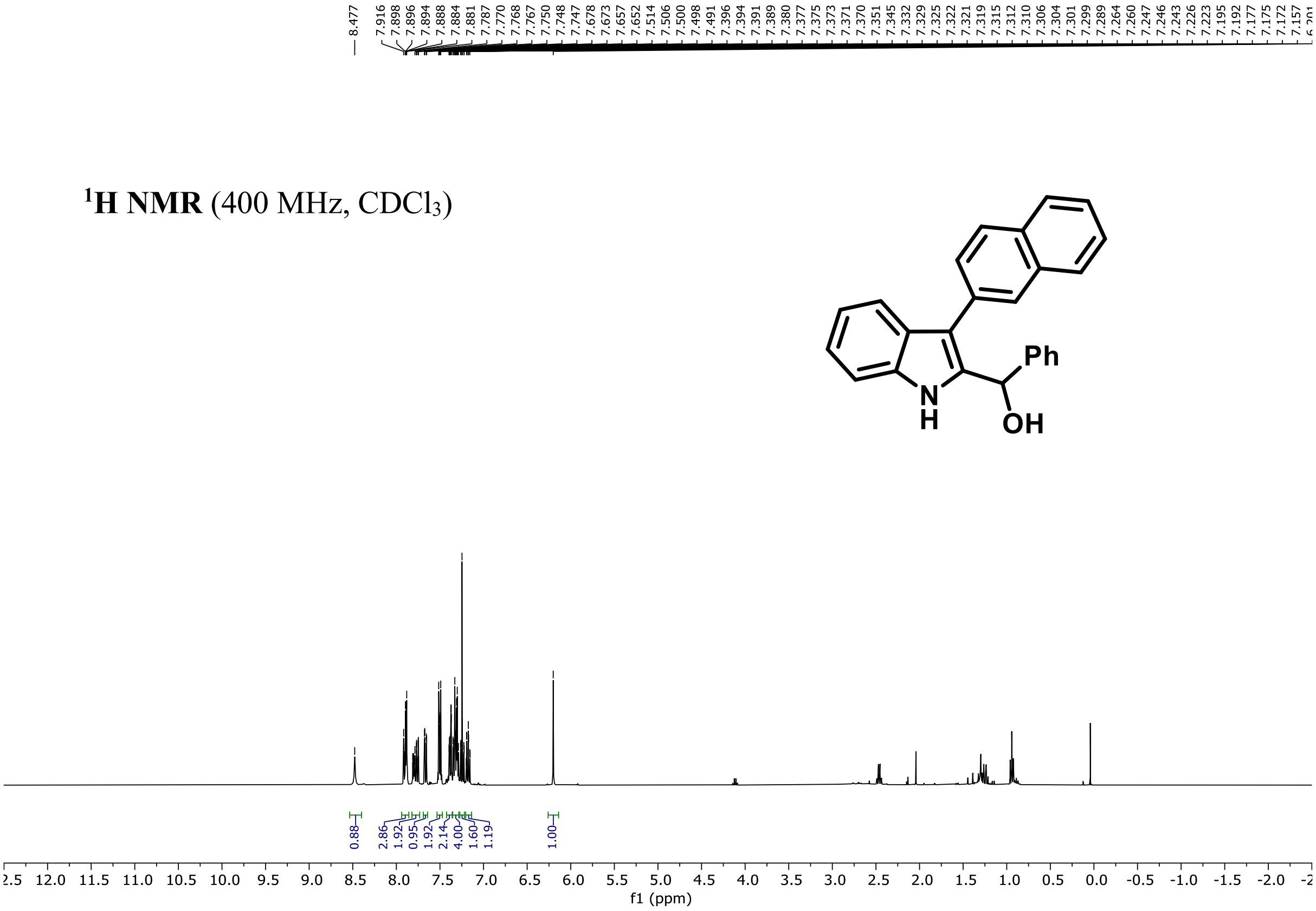

^{13}C {^{1}H} NMR (101 MHz, $CDCl_3$)

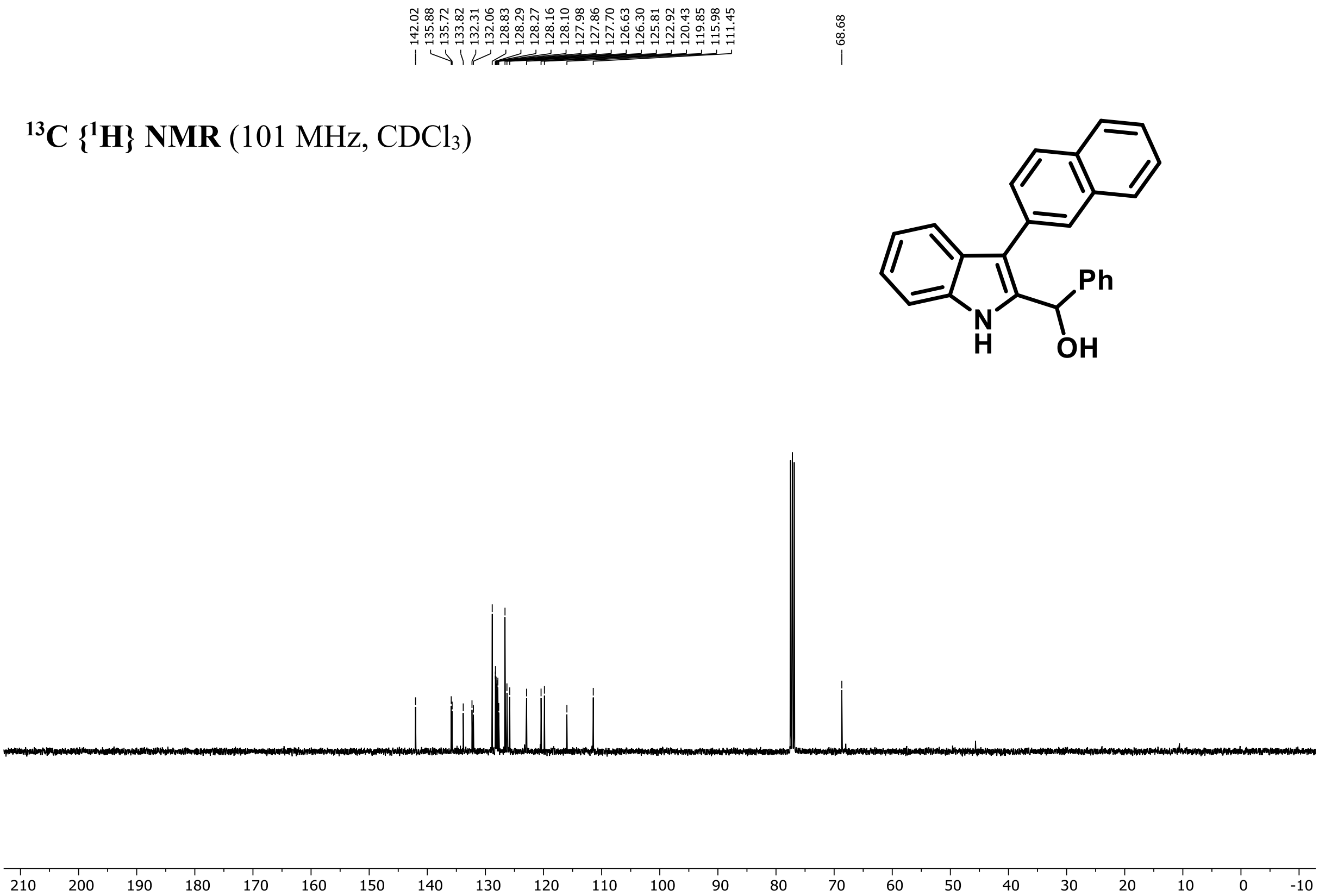

(5-Methoxy-3-phenyl-1*H*-indol-2-yl)(phenyl)methanol (2u):

^{1}H NMR (400 MHz, $CDCl_3$)

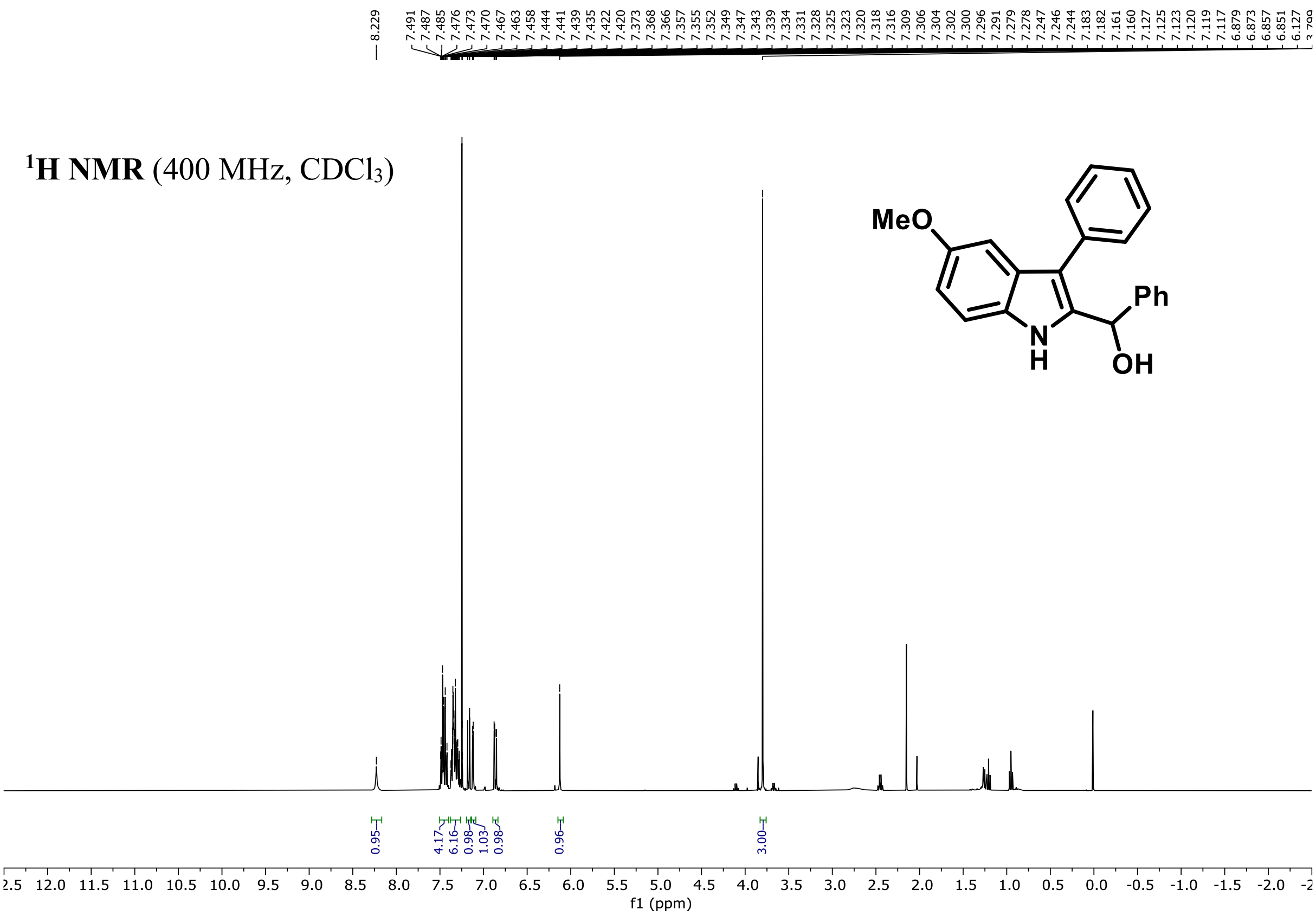

^{13}C {^{1}H} NMR (101 MHz, $CDCl_3$)

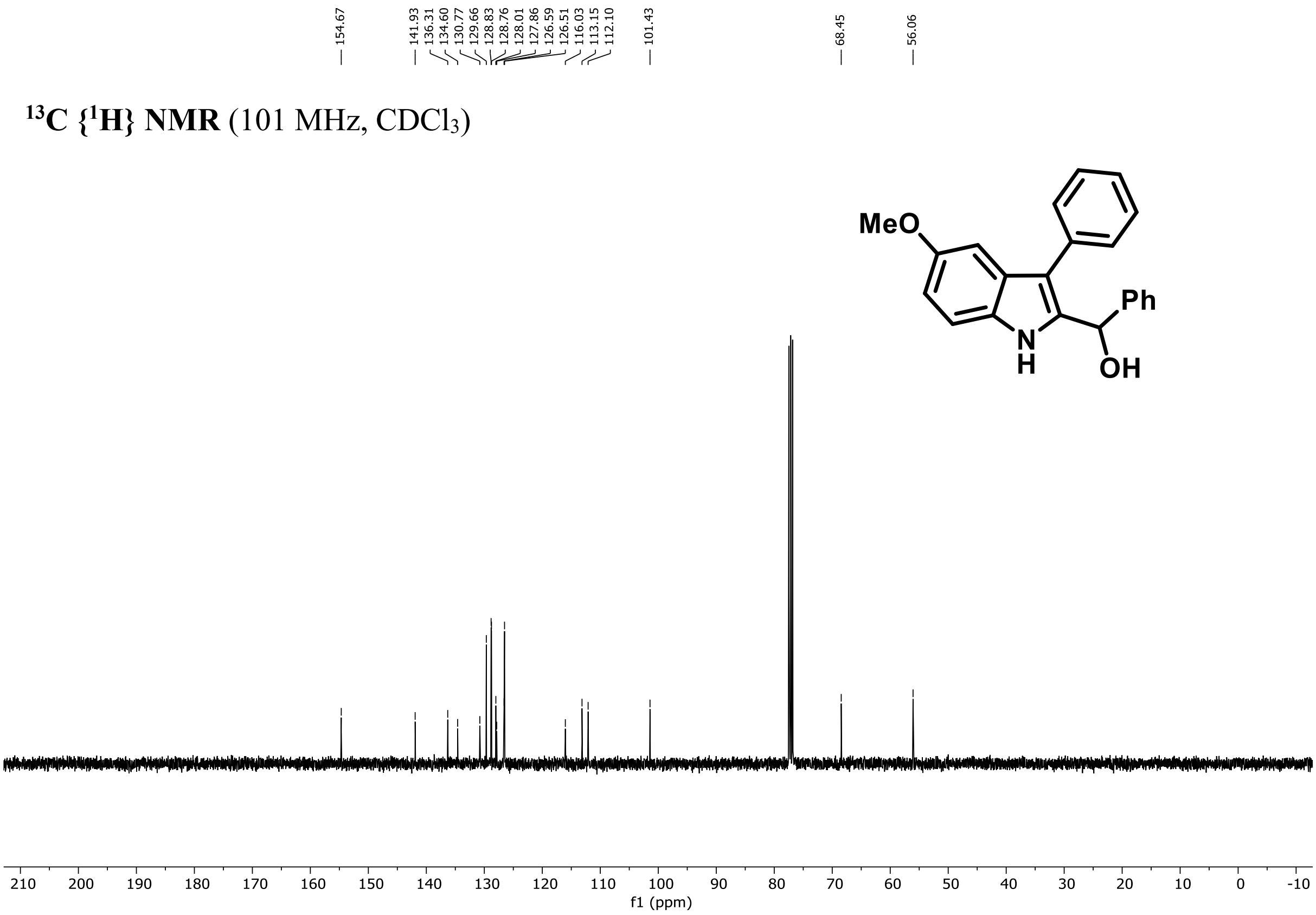

(5-Fluoro-3-phenyl-1*H*-indol-2-yl)(phenyl)methanol (2v):

^{1}H NMR (400 MHz, $CDCl_3$)

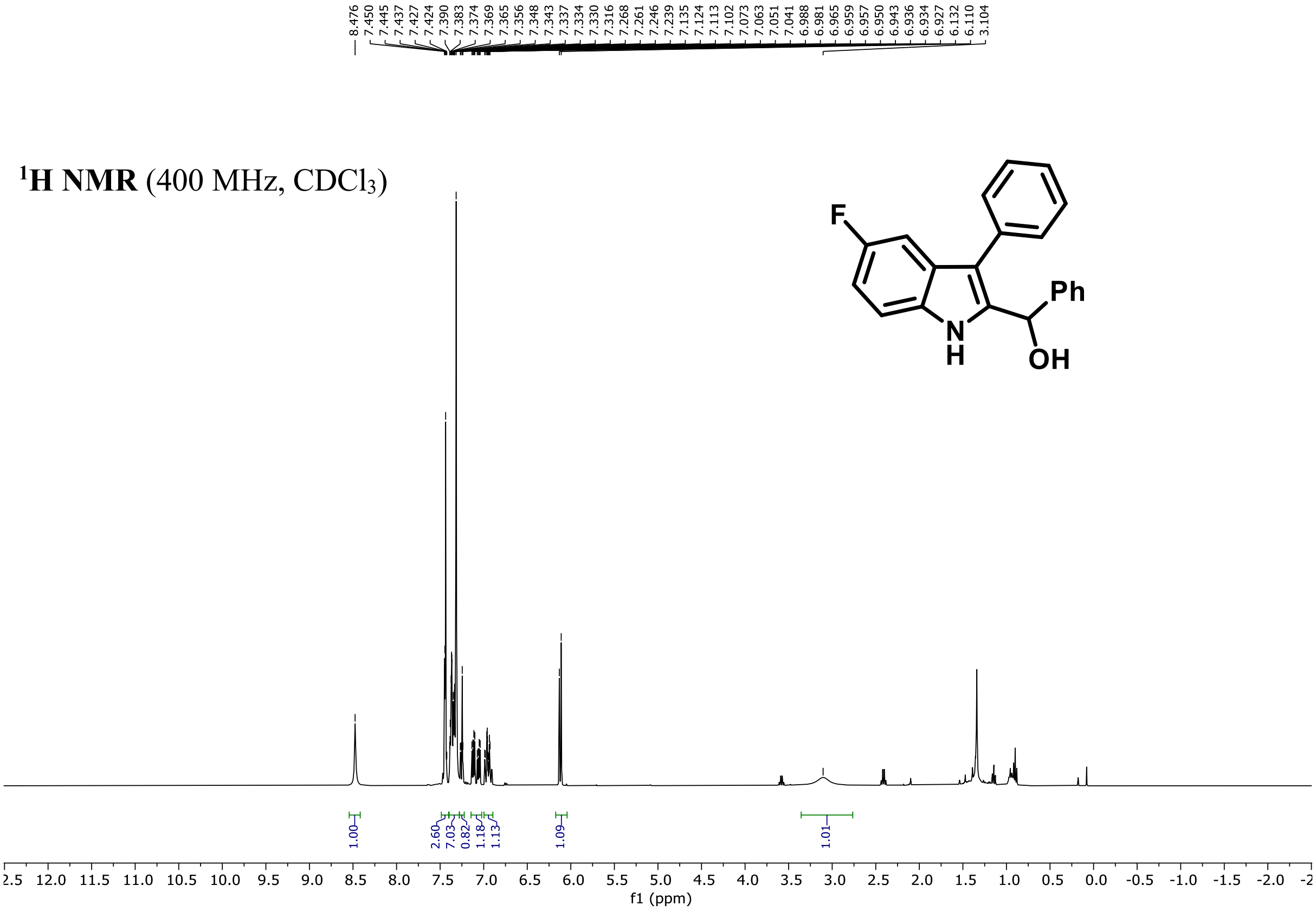

^{13}C {^{1}H} NMR (101 MHz, $CDCl_3$)

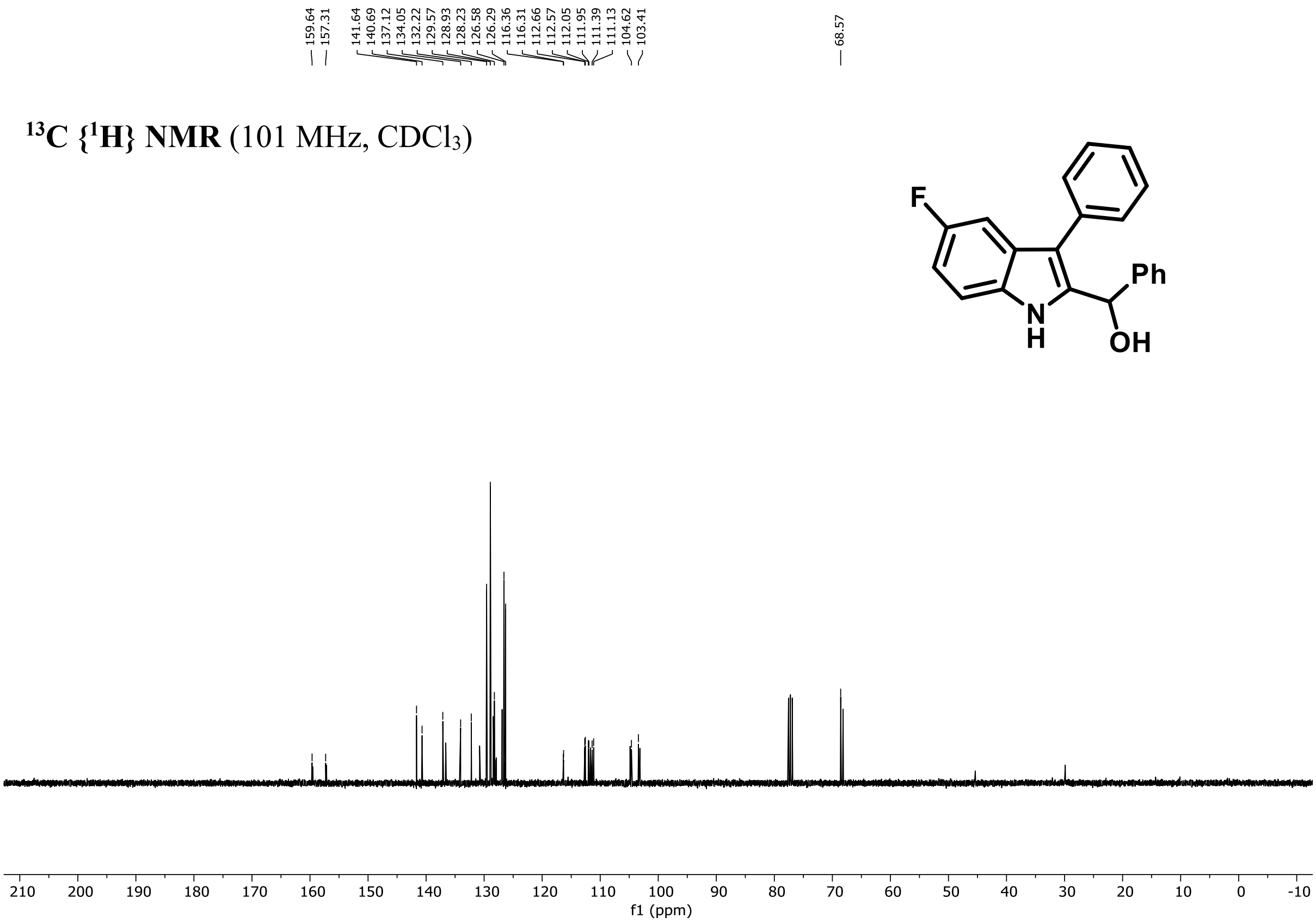

^{19}F NMR (376 MHz, $CDCl_3$)

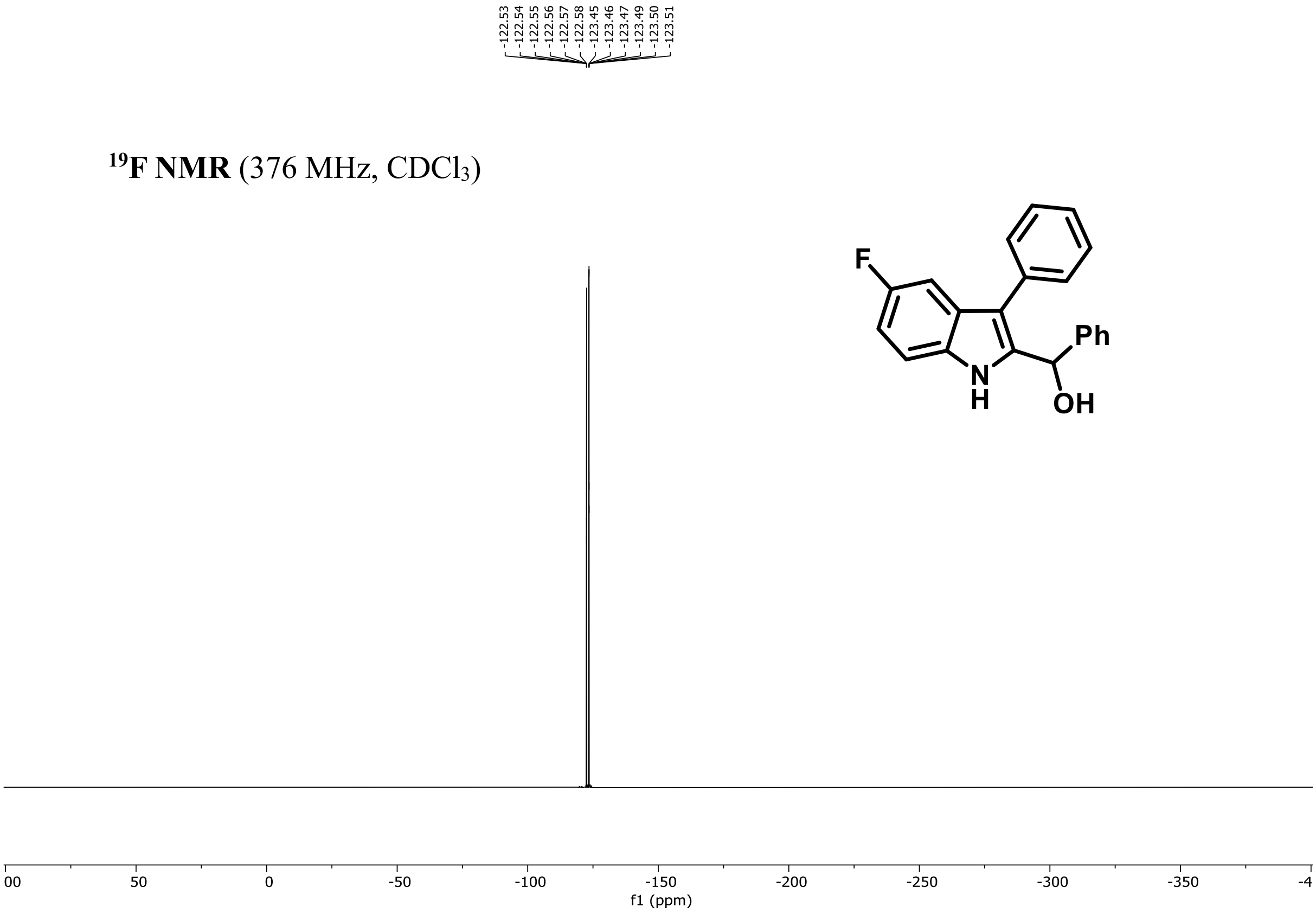

(5-Bromo-3-phenyl-1*H*-indol-2-yl)(phenyl)methanol (2w):

^{1}H NMR (400 MHz, $CDCl_3$)

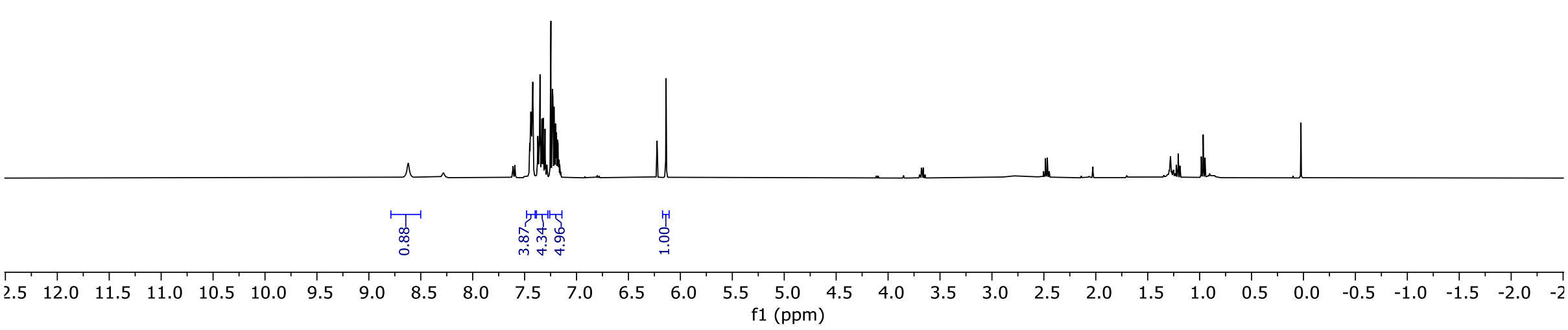

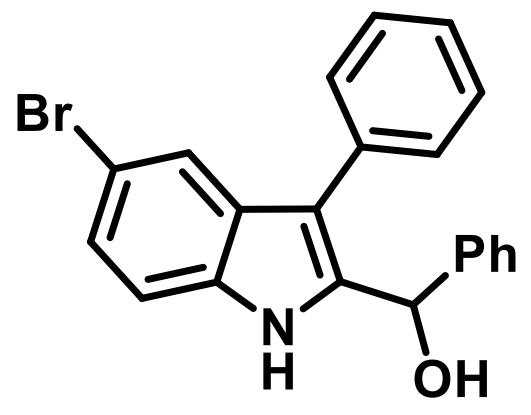

^{13}C {^{1}H} NMR (101 MHz, $CDCl_3$)

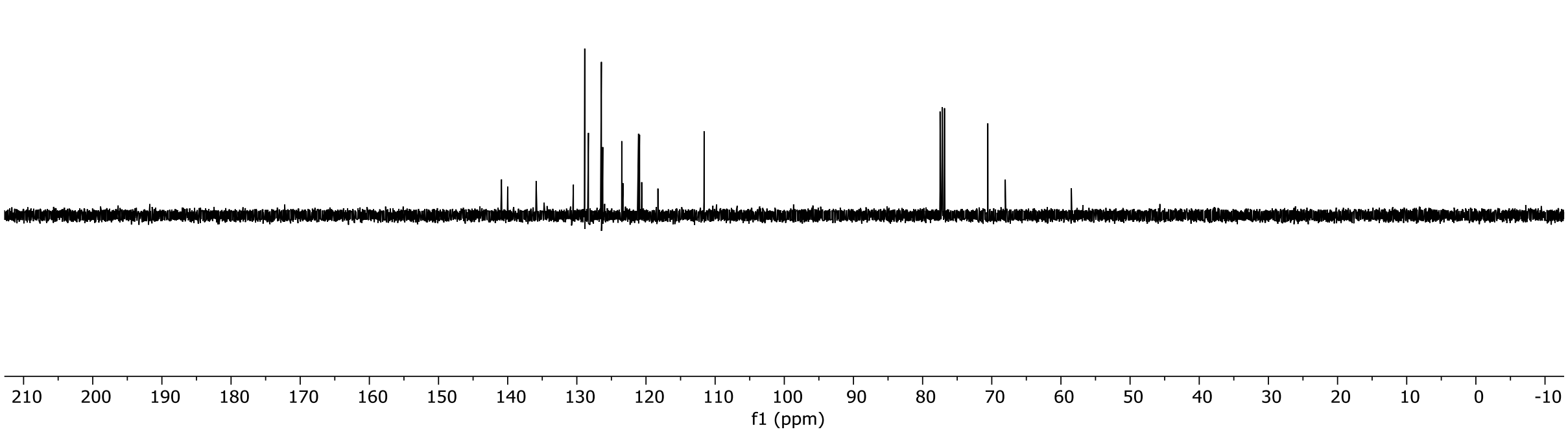

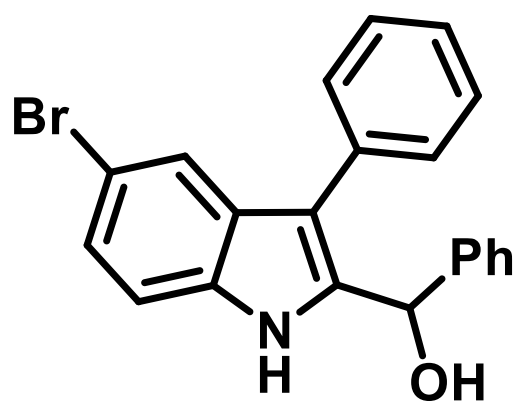

(3-Methyl-1*H*-indol-2-yl)methanol (2x):

^{1}H NMR (400 MHz, DMSO-d6)

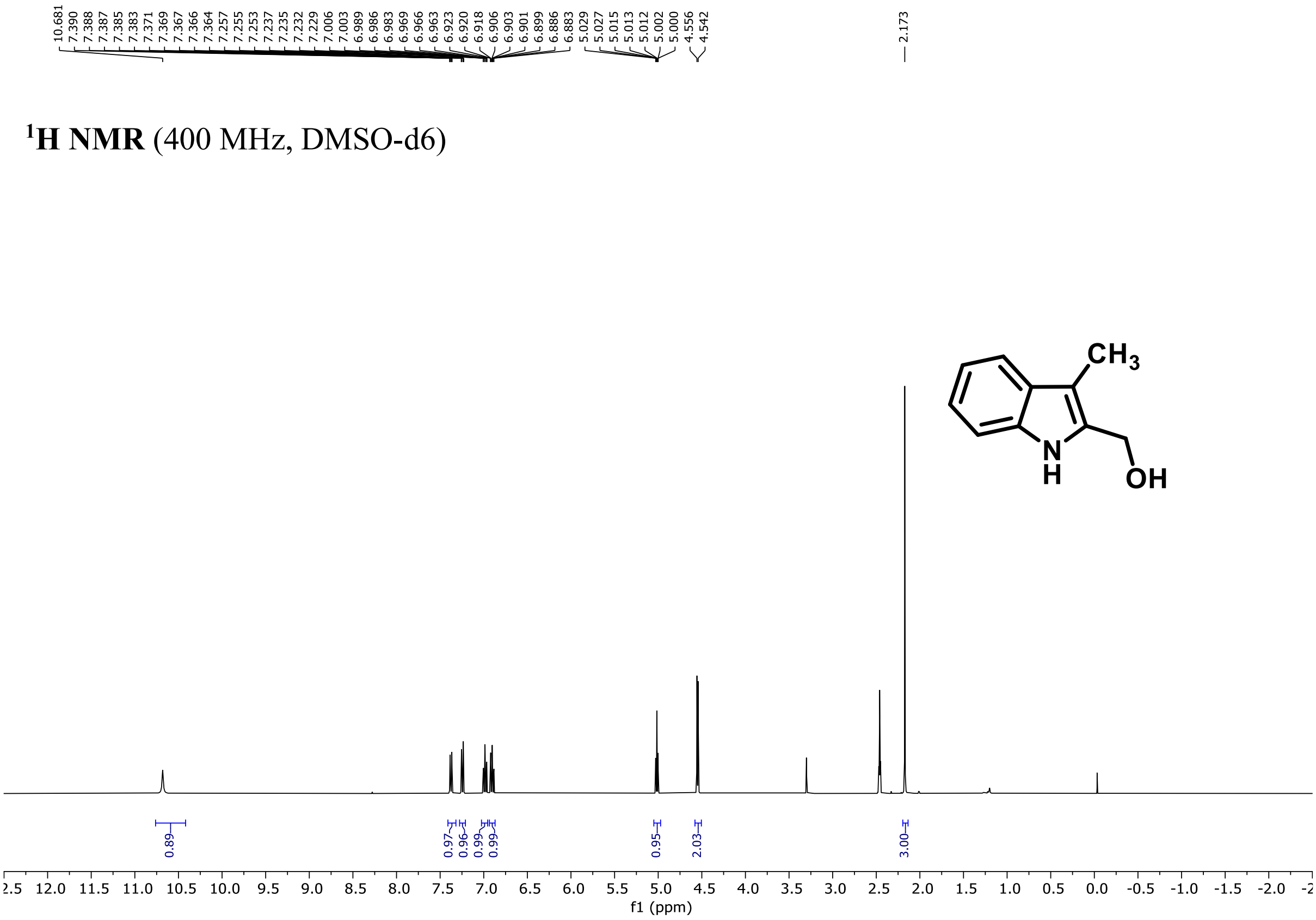

^{13}C {^{1}H}NMR (101 MHz, DMSO-d6)

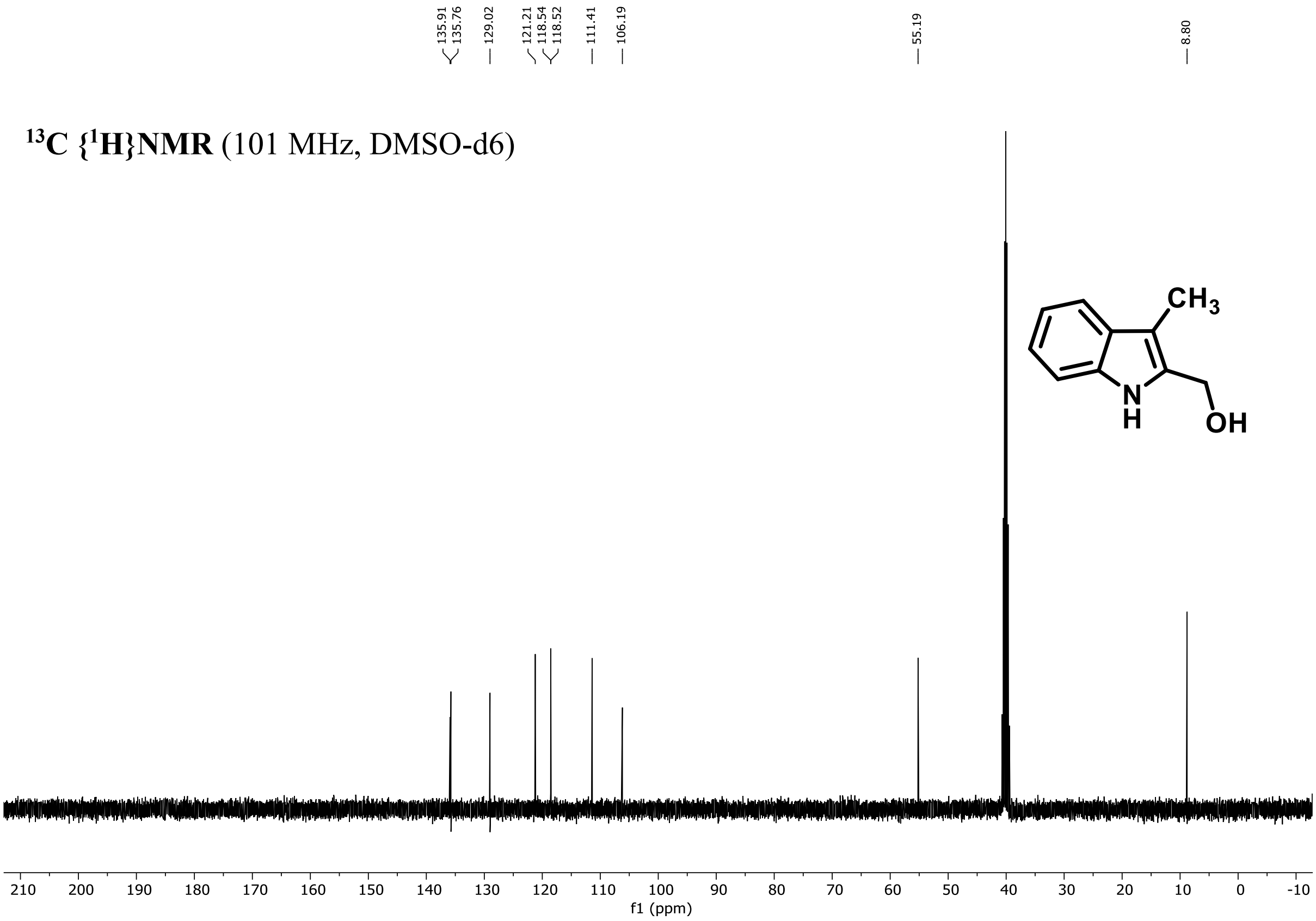

(1*H*-Indol-2-yl)diphenylmethanol (2y):

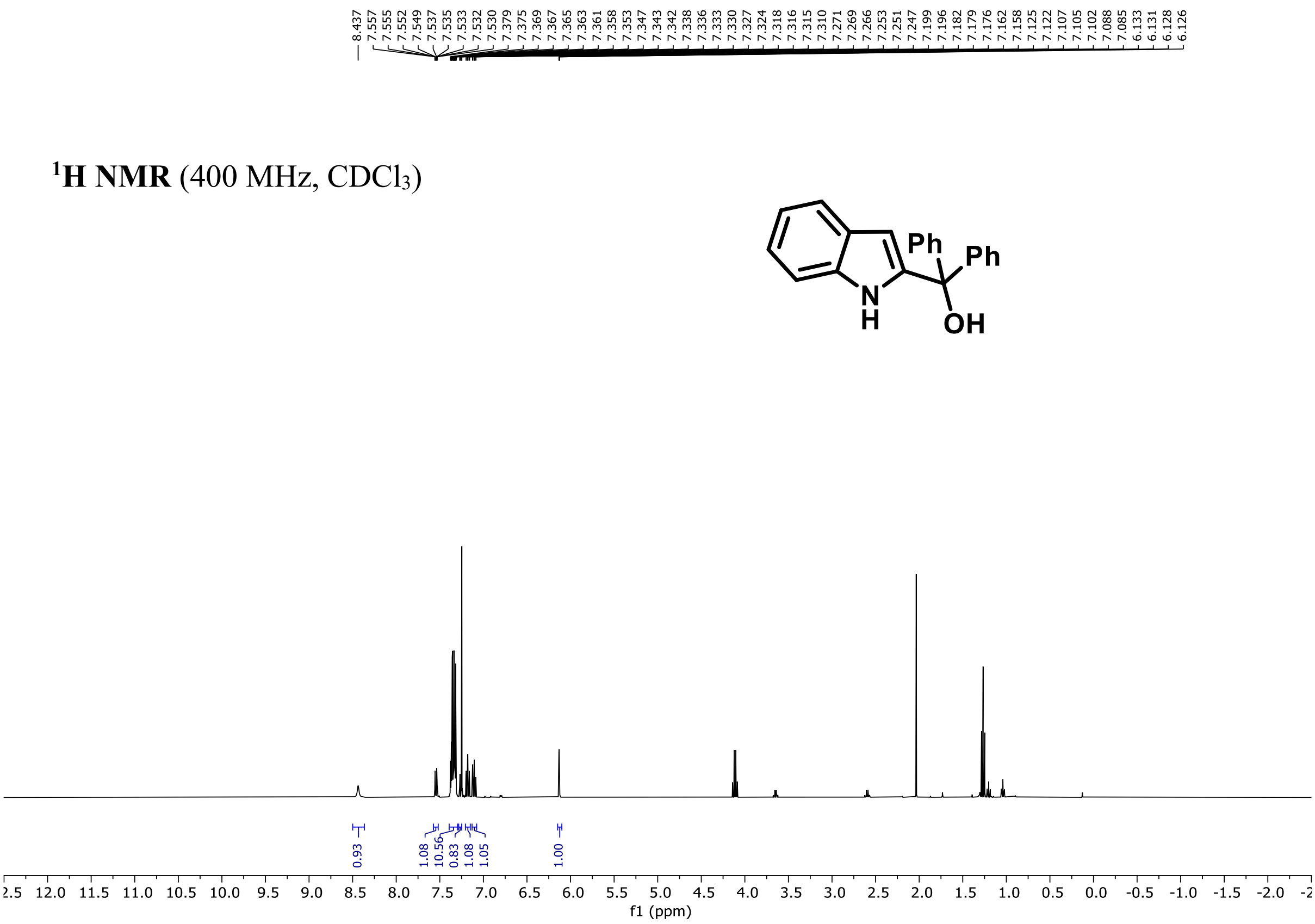

^{1}H NMR (400 MHz, $CDCl_3$)

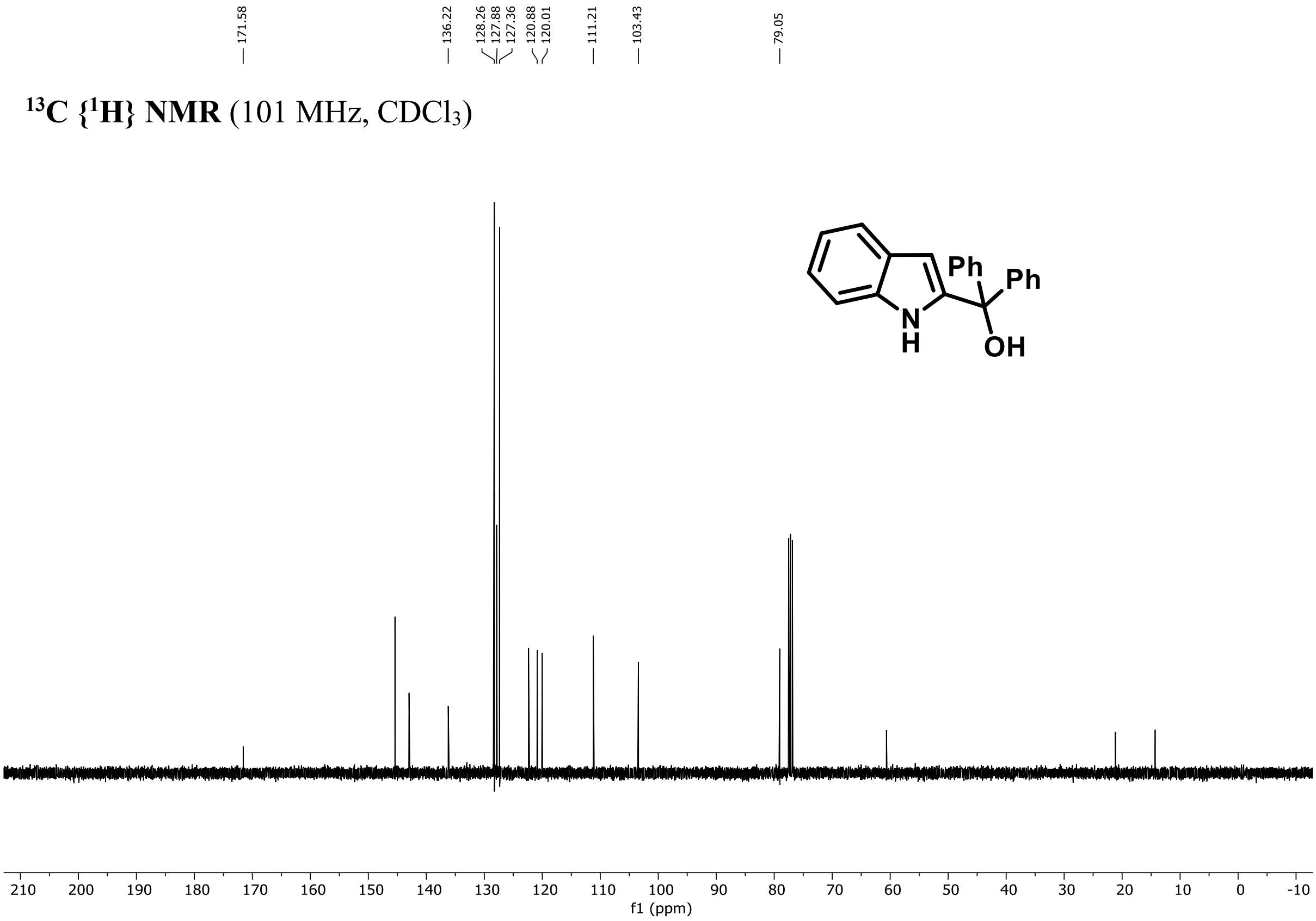

^{13}C {^{1}H} NMR (101 MHz, $CDCl_3$)

Methyl-10-methyl-6,9-diphenyl-6,7-dihydro-6,8-methanopyrido[1,2-a]indole-8(9*H*)-carboxylate (3a):

^{1}H NMR (400 MHz, $CDCl_3$)

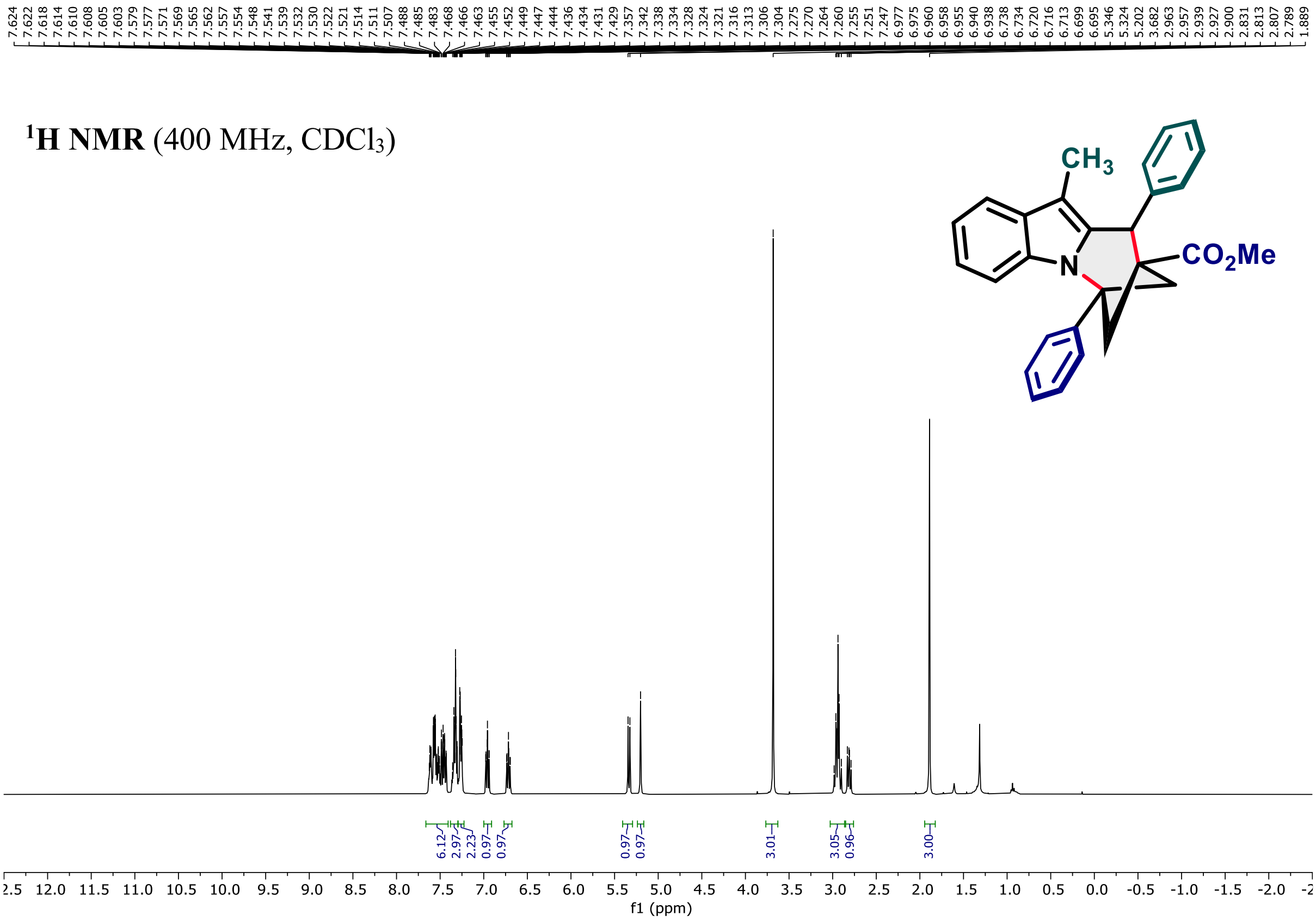

^{13}C {^{1}H} NMR (101 MHz, $CDCl_3$)

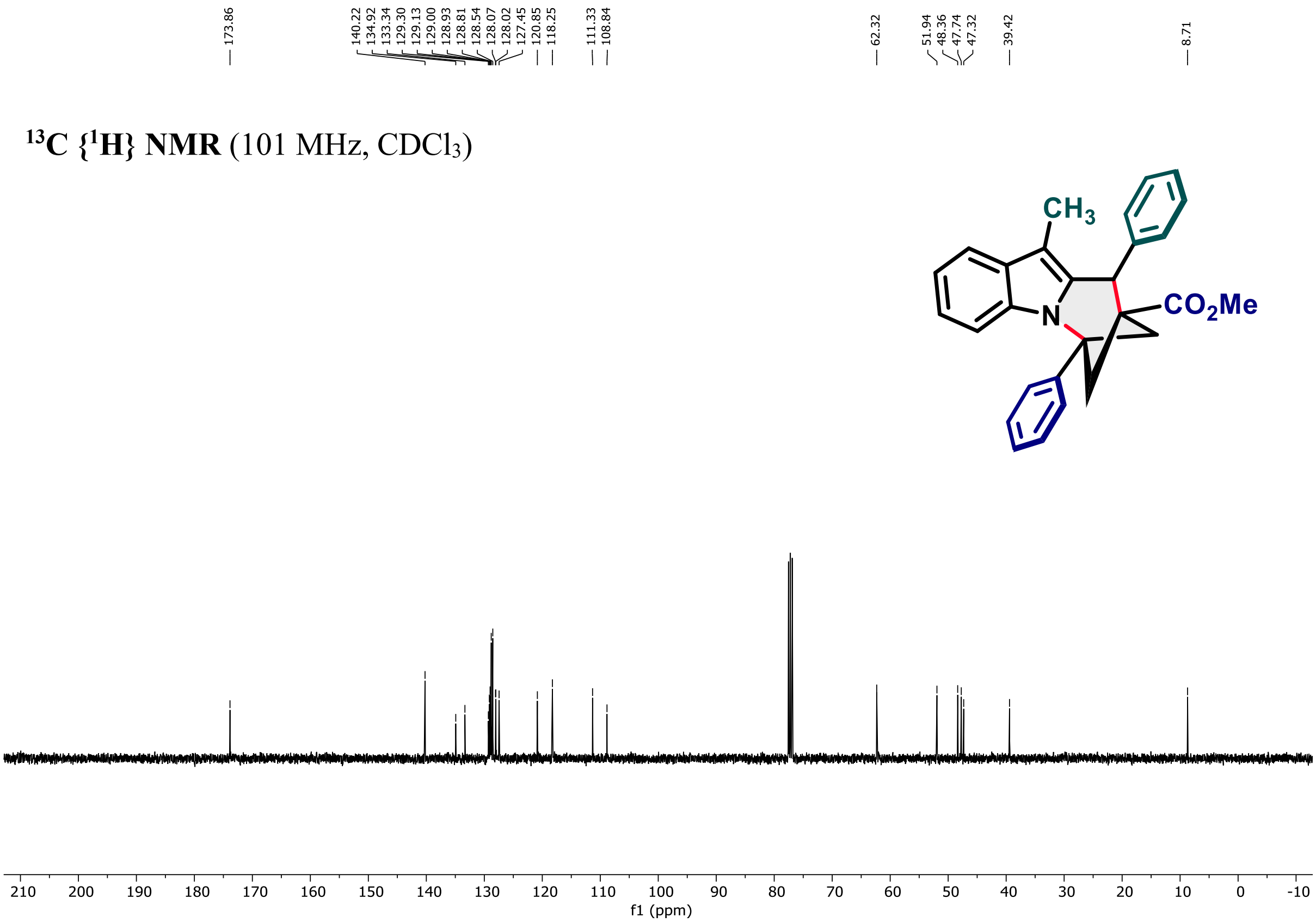

Methyl-10-methyl-6-phenyl-9-(*p*-tolyl)-6,7-dihydro-6,8-methanopyrido[1,2-a]indole-8(9*H*)-carboxylate (3b):

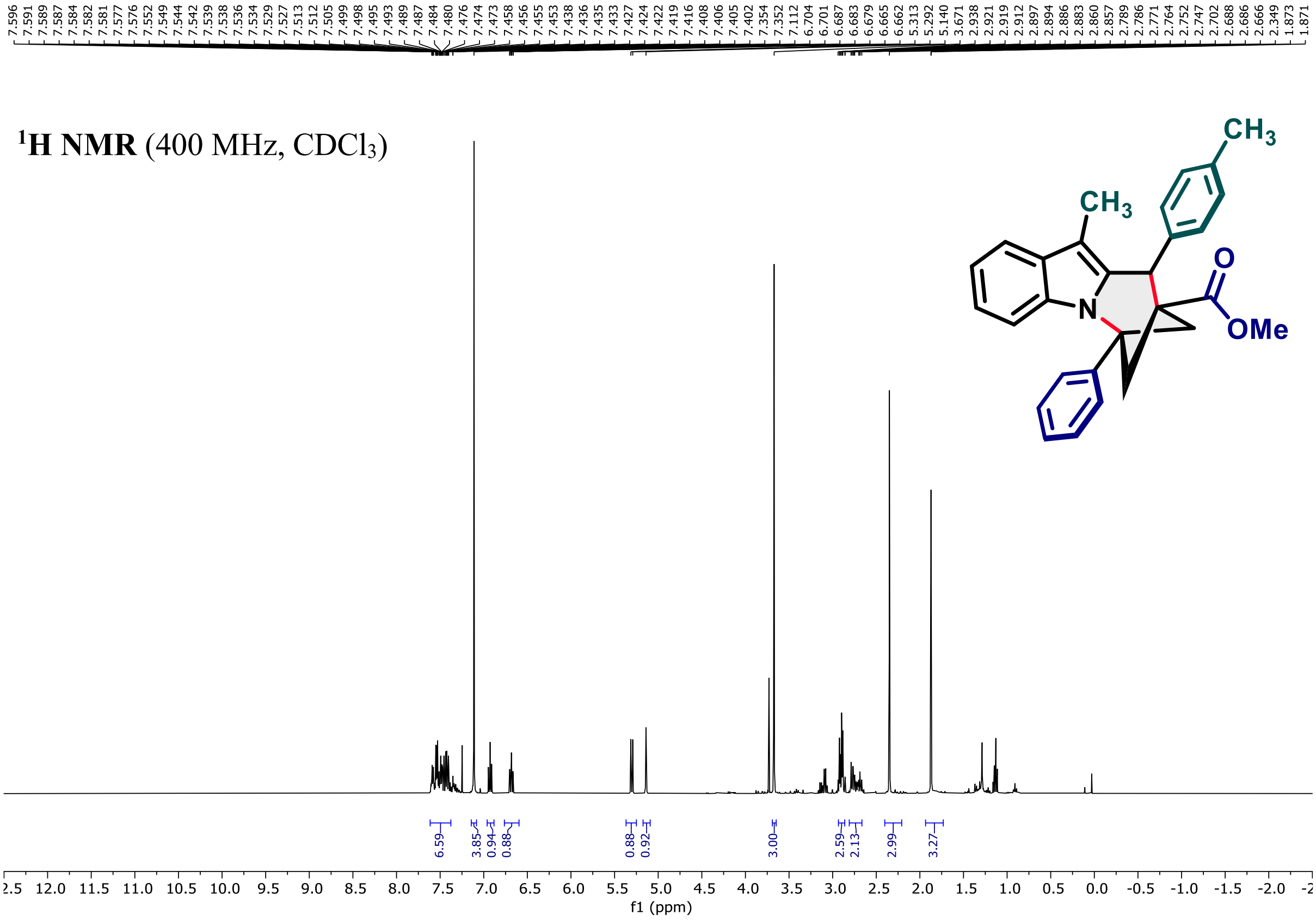

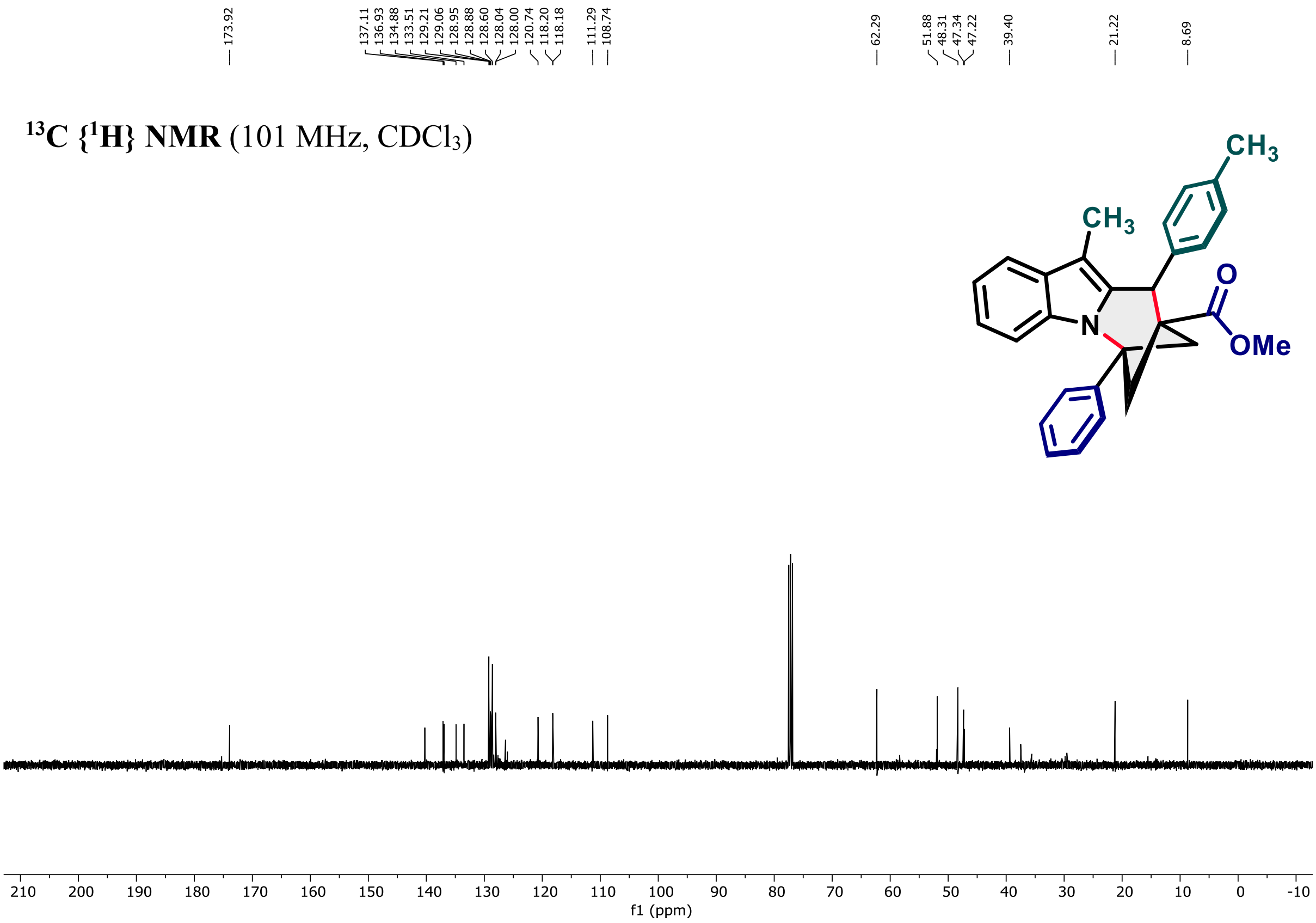

Methyl-9-(4-(*tert*-butyl)phenyl)-10-methyl-6-phenyl-6,7-dihydro-6,8-methanopyrido [1,2-a]indole-8(9*H*)-carboxylate (3c):

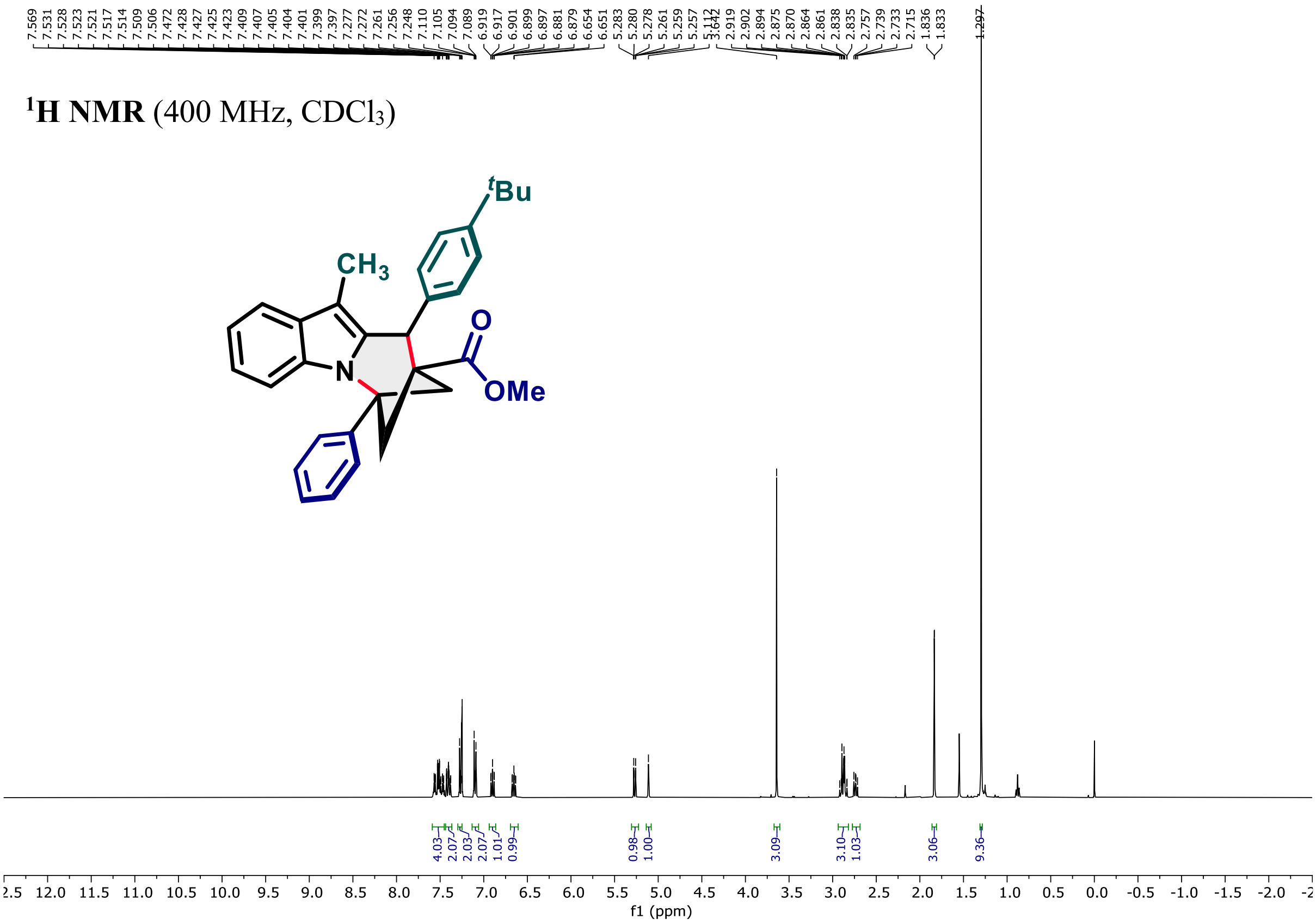

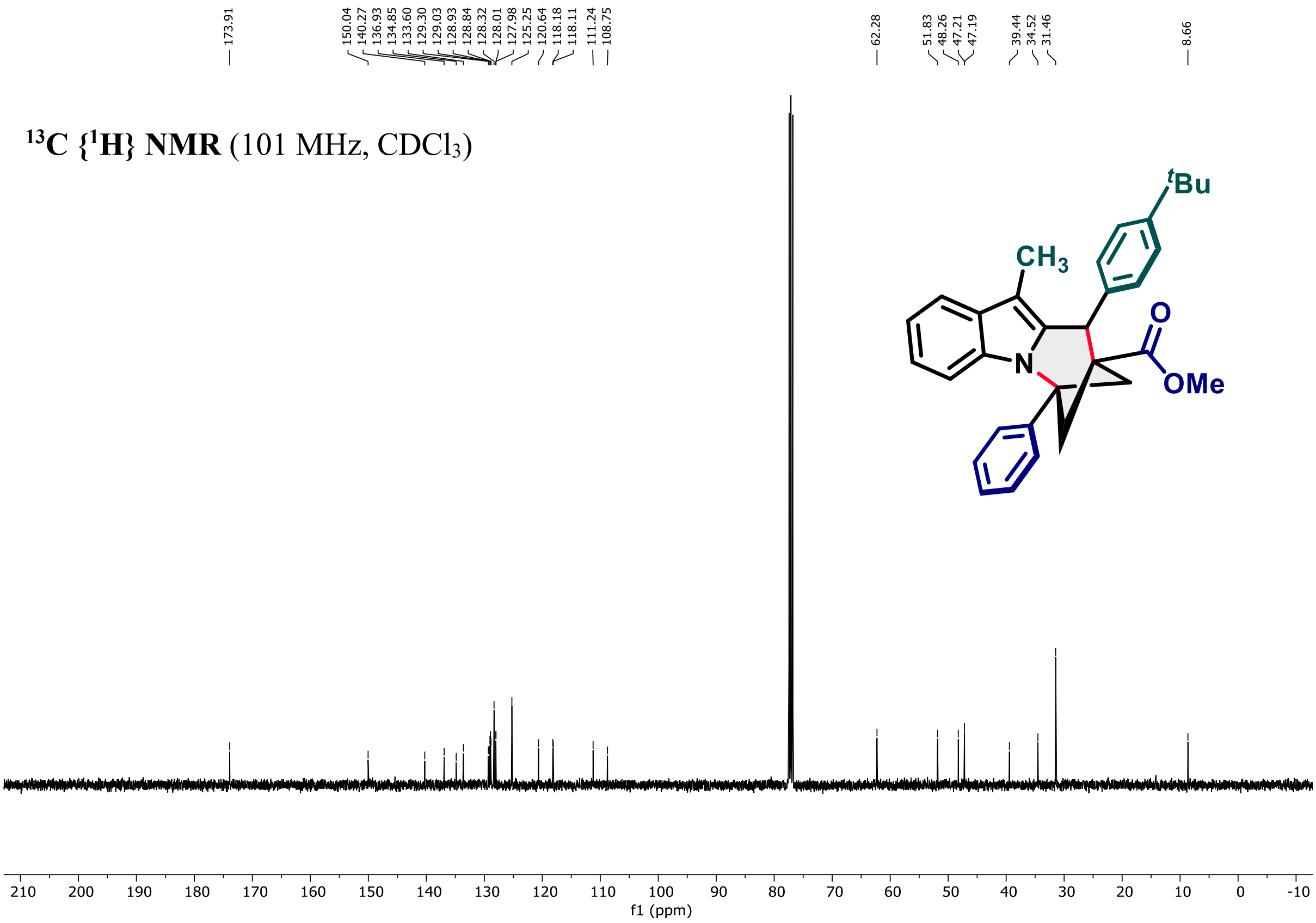

Methyl-10-methyl-9-(naphthalen-2-yl)-6-phenyl-6,7-dihydro-6,8-methanopyrido [1,2-a]indole-8(9*H*)-carboxylate (3d):

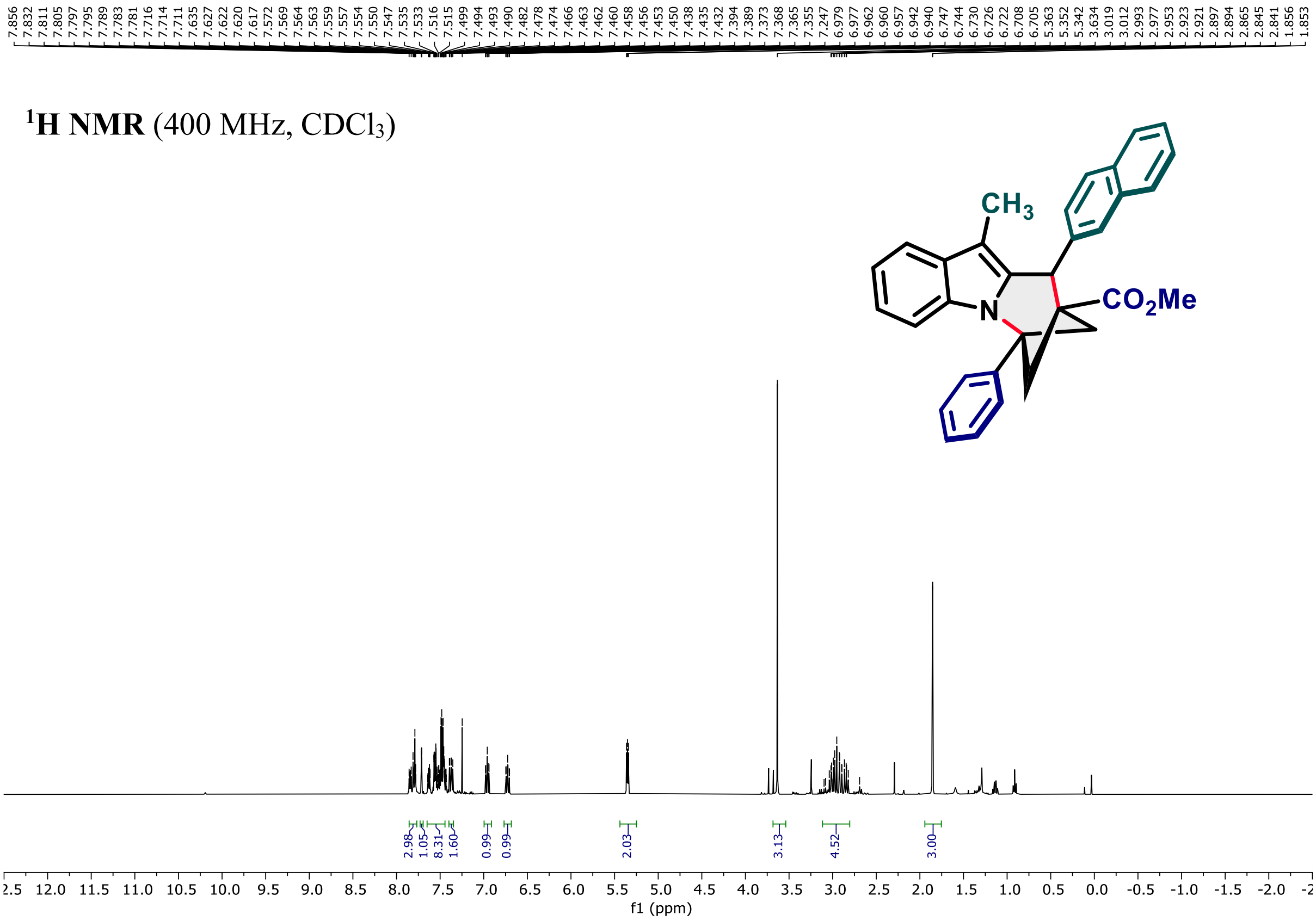

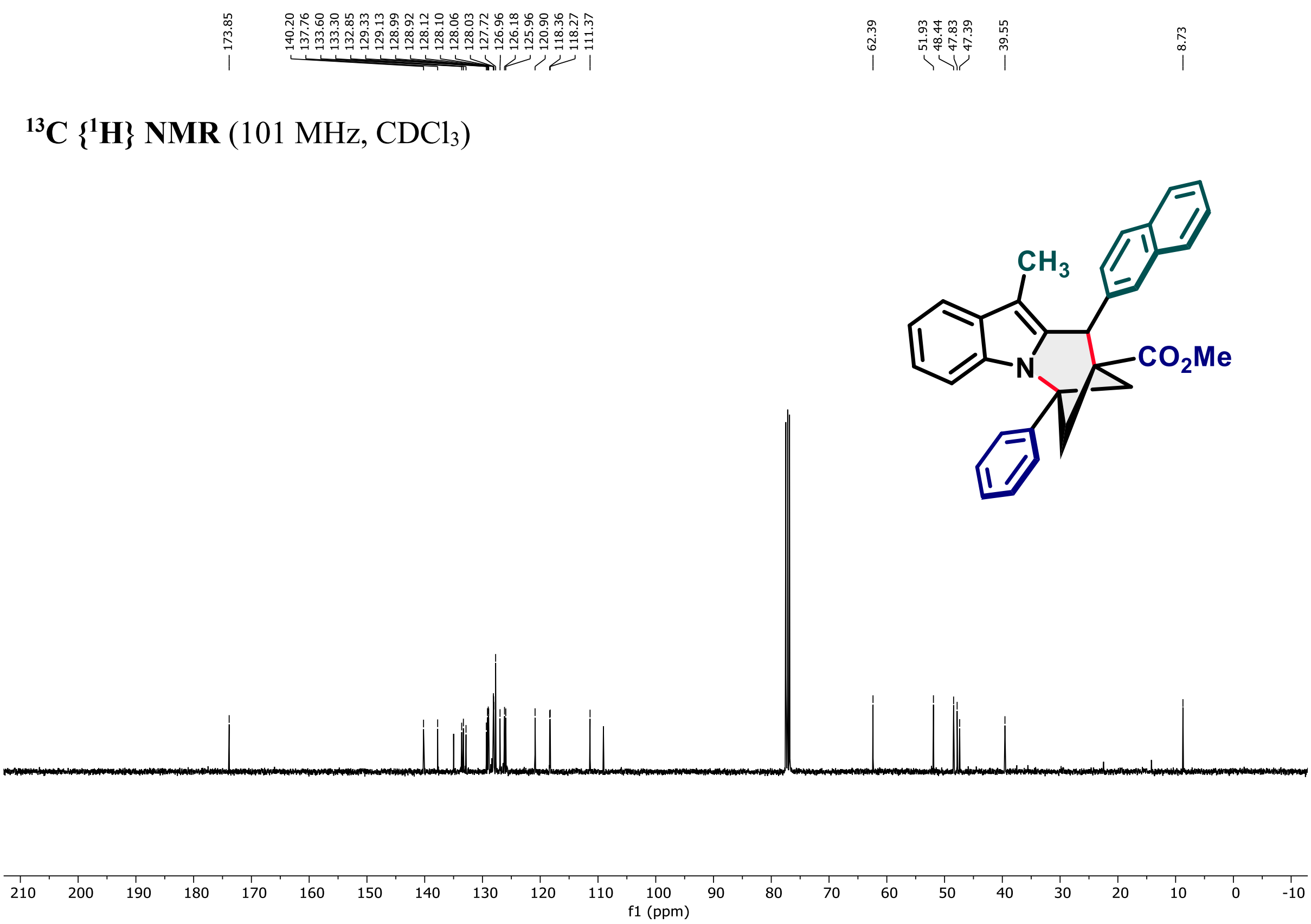

Methyl 9-(4-methoxyphenyl)-10-methyl-6-phenyl-6,7-dihydro-6,8-methanopyrido [1,2-a]indole-8(9*H*)-carboxylate (3e):

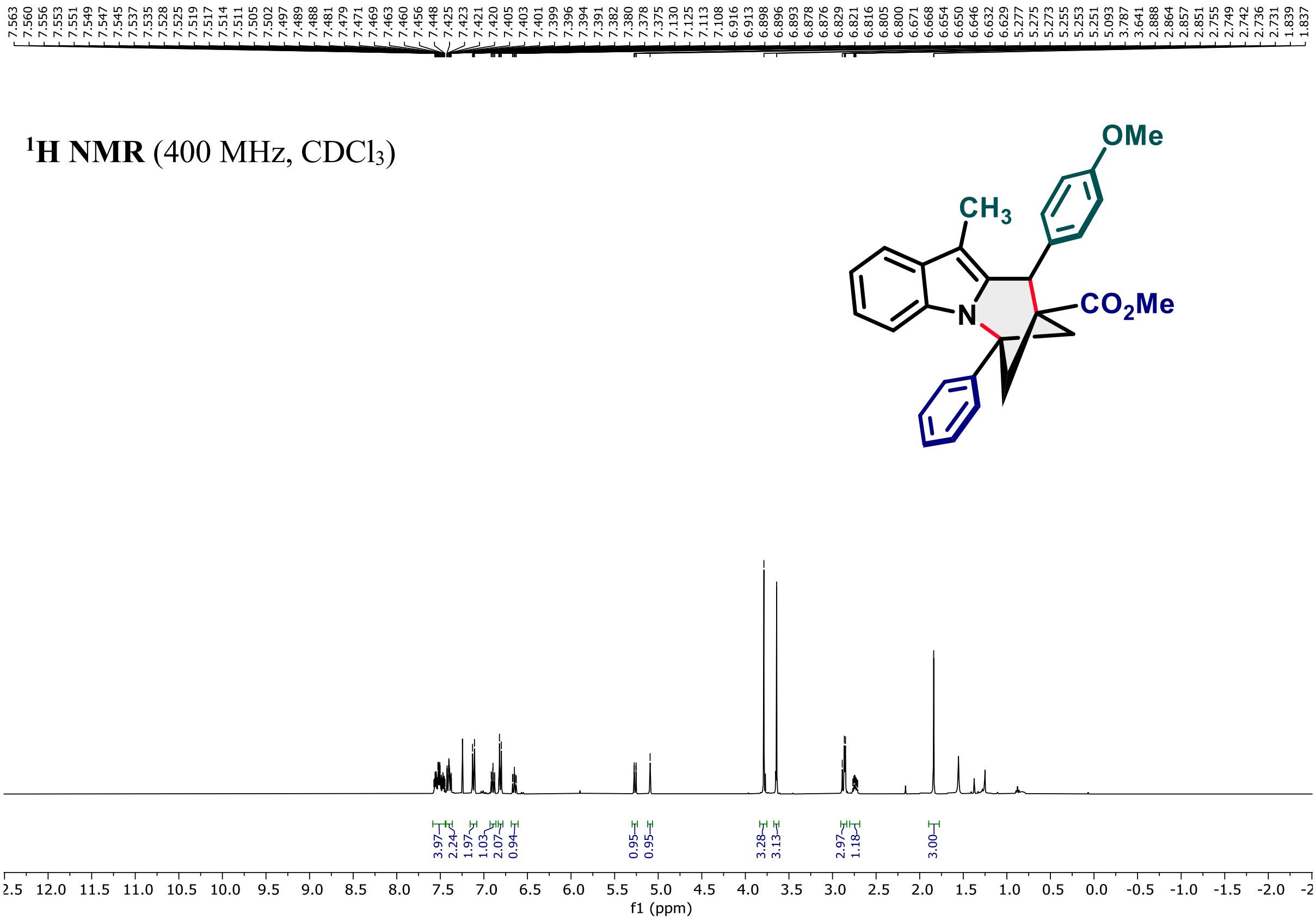

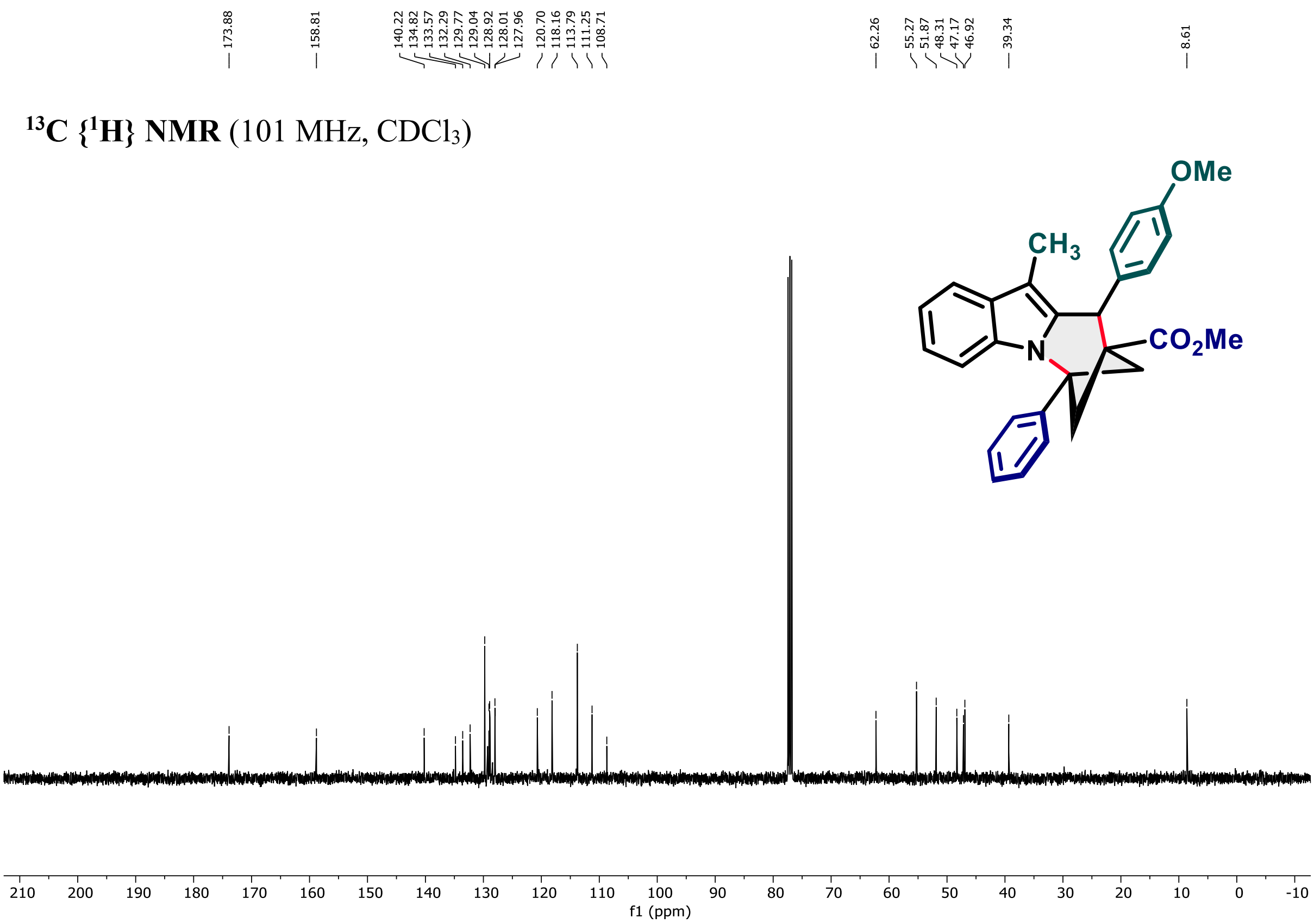

Methyl 9-(3-methoxyphenyl)-10-methyl-6-phenyl-6,7-dihydro-6,8-methanopyrido [1,2-a]indole-8(9*H*)-carboxylate (3f):

^{1}H NMR (400 MHz, $CDCl_3$)

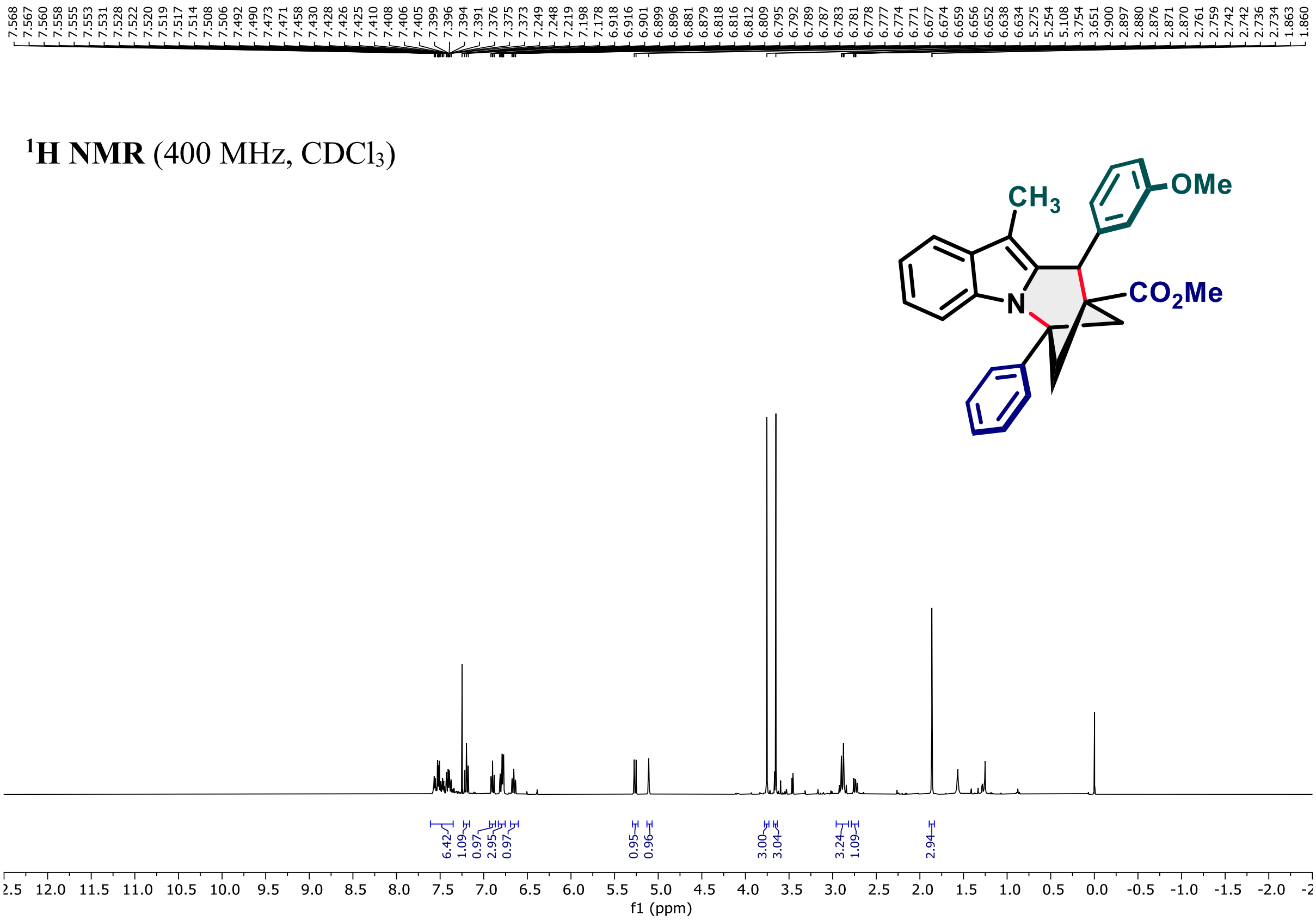

^{13}C {^{1}H} NMR (101 MHz, $CDCl_3$)

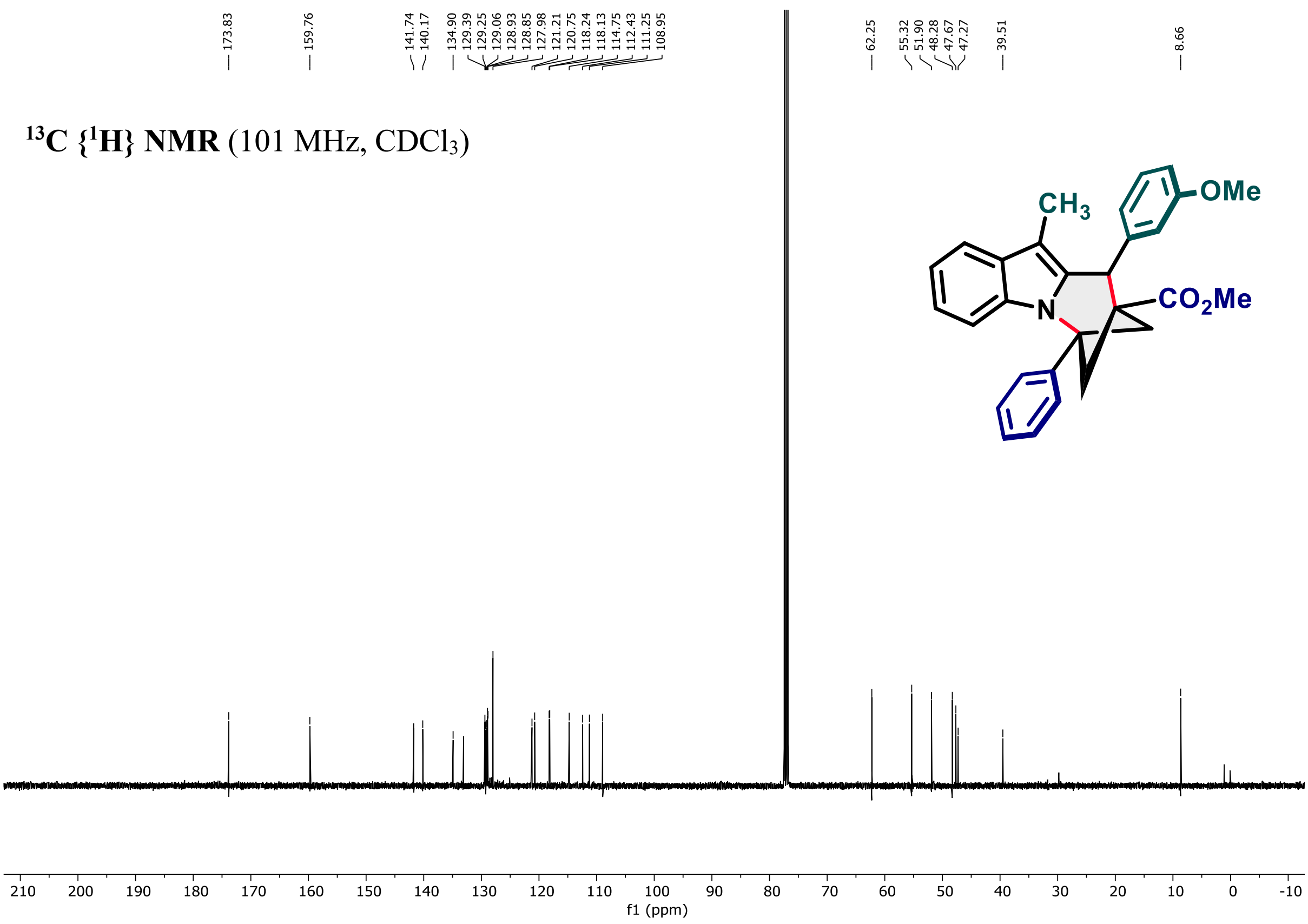

Methyl-9-(4-fluorophenyl)-10-methyl-6-phenyl-6,7-dihydro-6,8-methanopyrido[1,2-a]indole-8(9*H*)-carboxylate (3g):

[1]**H NMR** (400 MHz, $CDCl_3$)

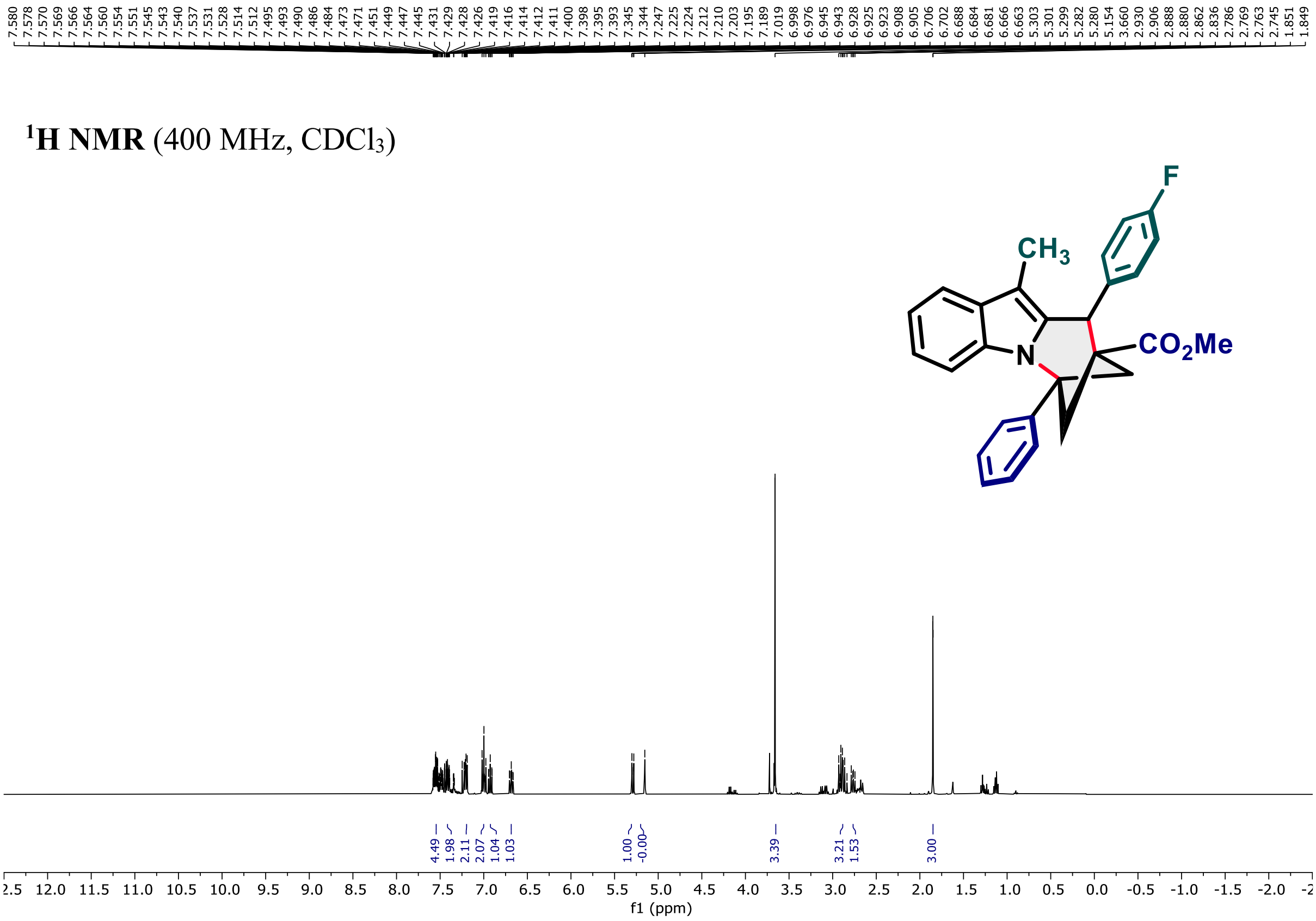

[13]**C {^{1}H} NMR** (101 MHz, $CDCl_3$)

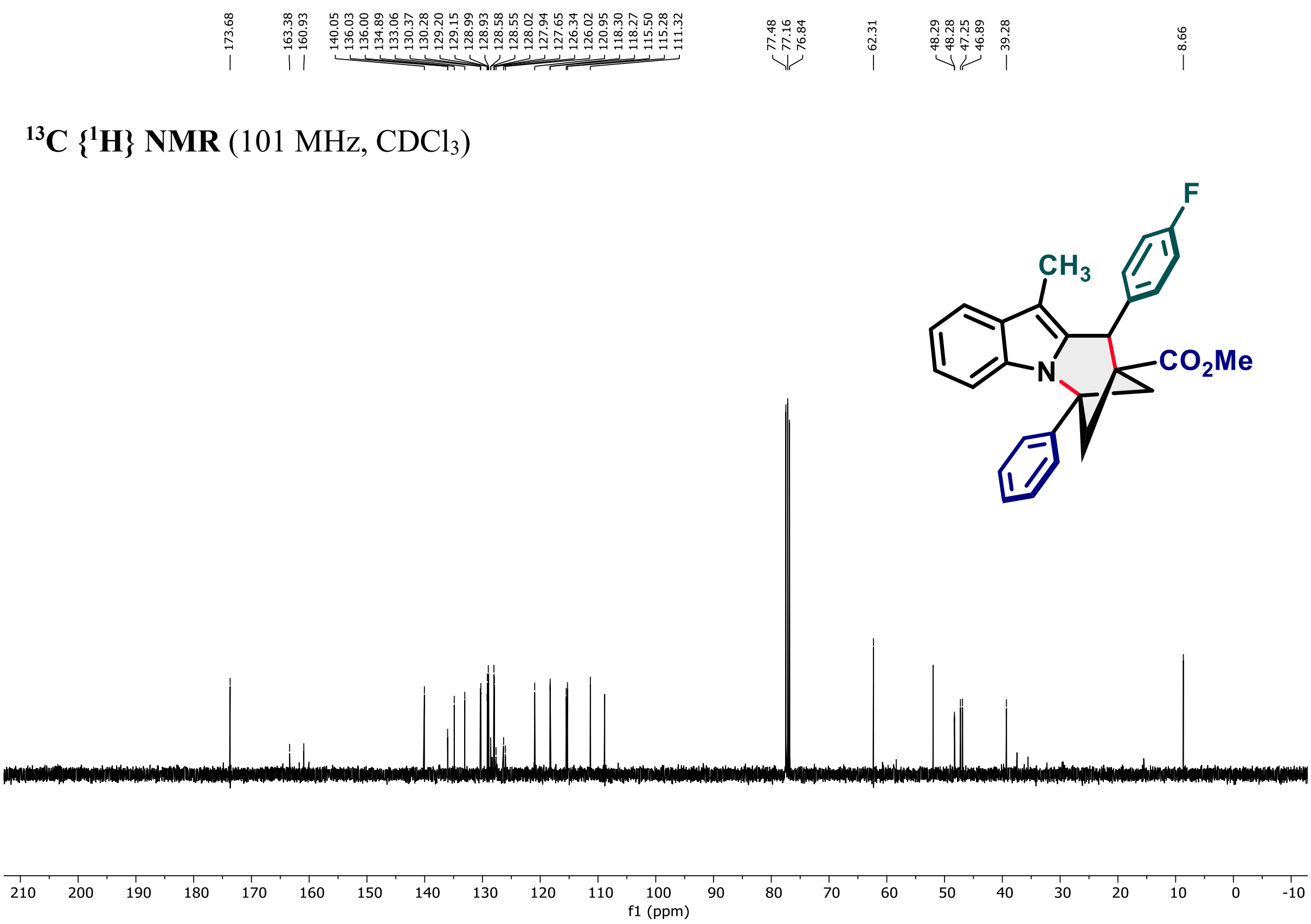

^{19}F NMR (376 MHz, $CDCl_3$)

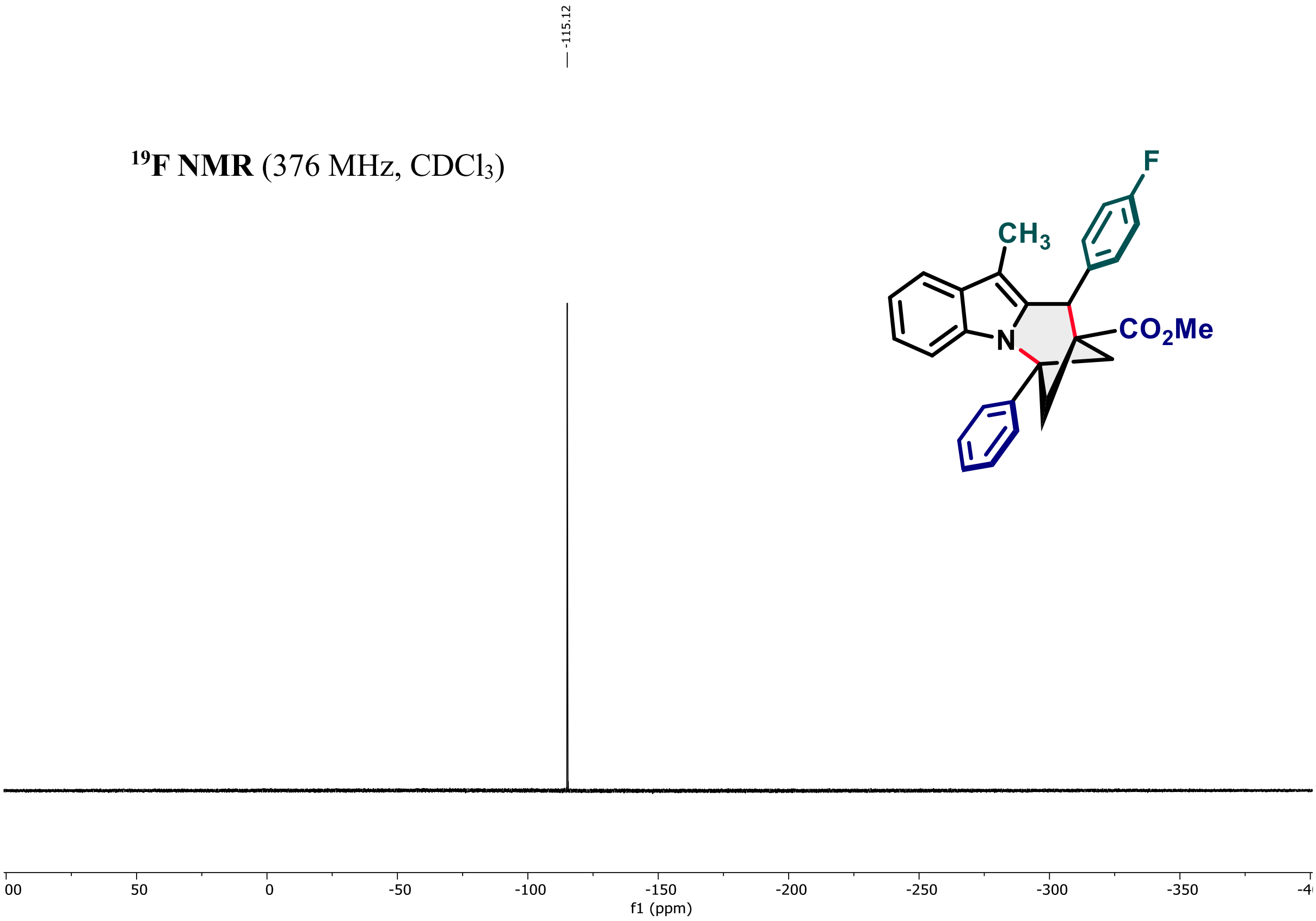

Methyl-9-(4-chlorophenyl)-10-methyl-6-phenyl-6,7-dihydro-6,8-methanopyrido[1,2-a]indole-8(9*H*)-carboxylate (3h):

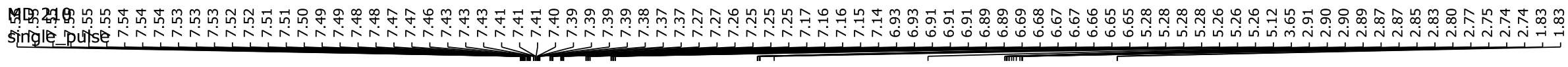

^{1}H NMR (400 MHz, $CDCl_3$)

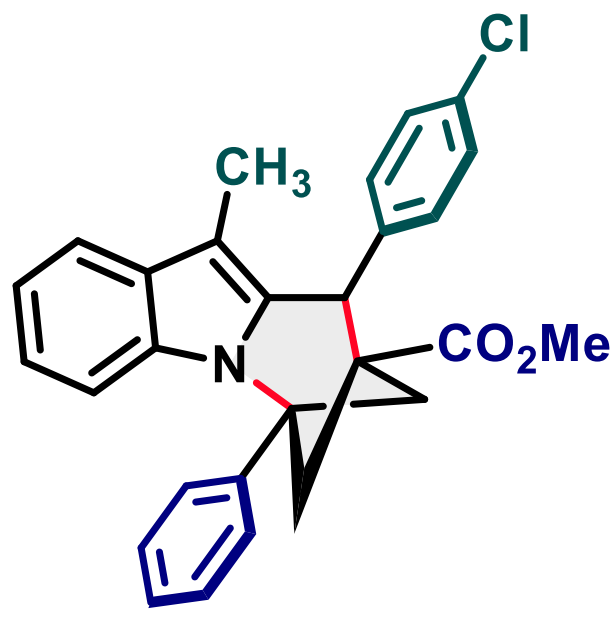

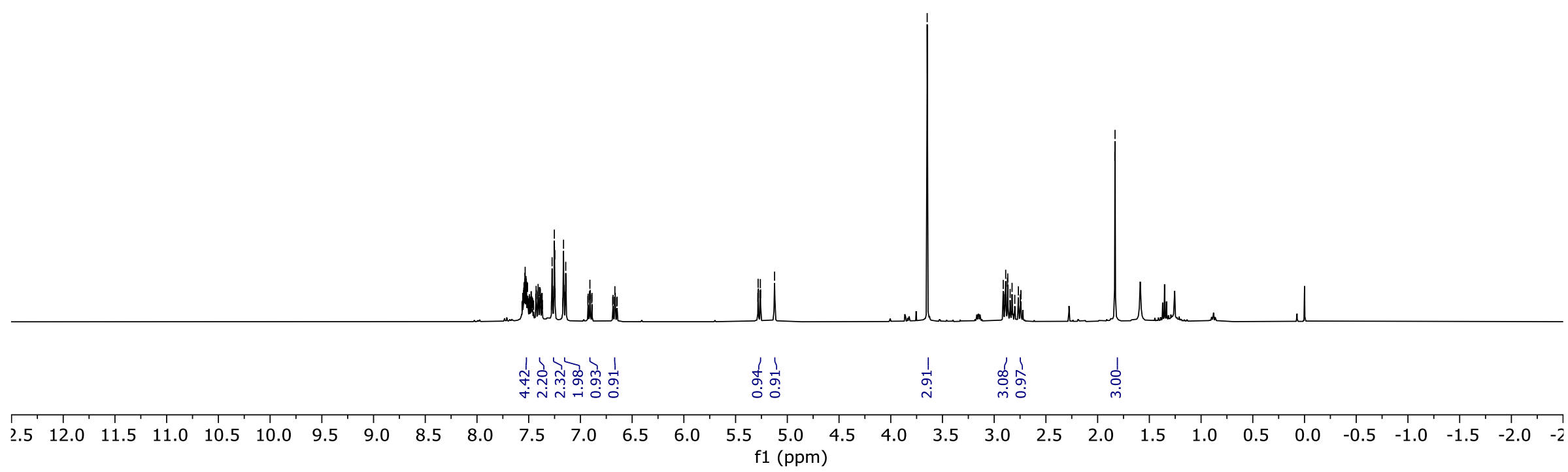

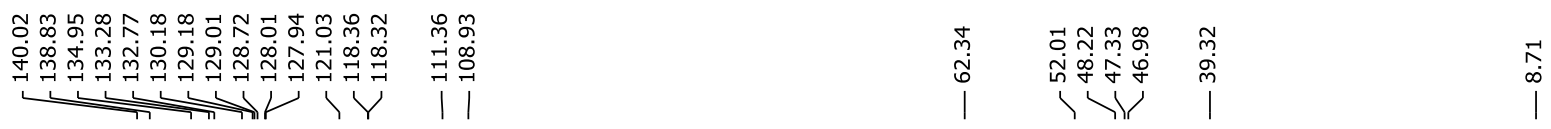

^{13}C {^{1}H} NMR (101 MHz, $CDCl_3$)

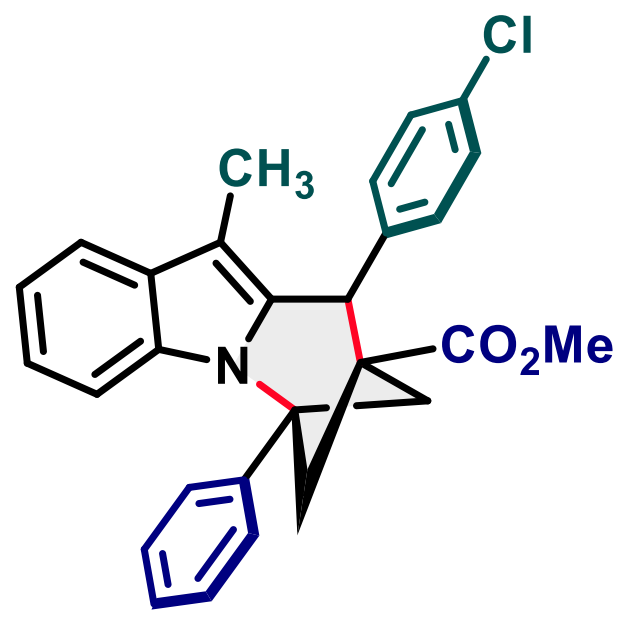

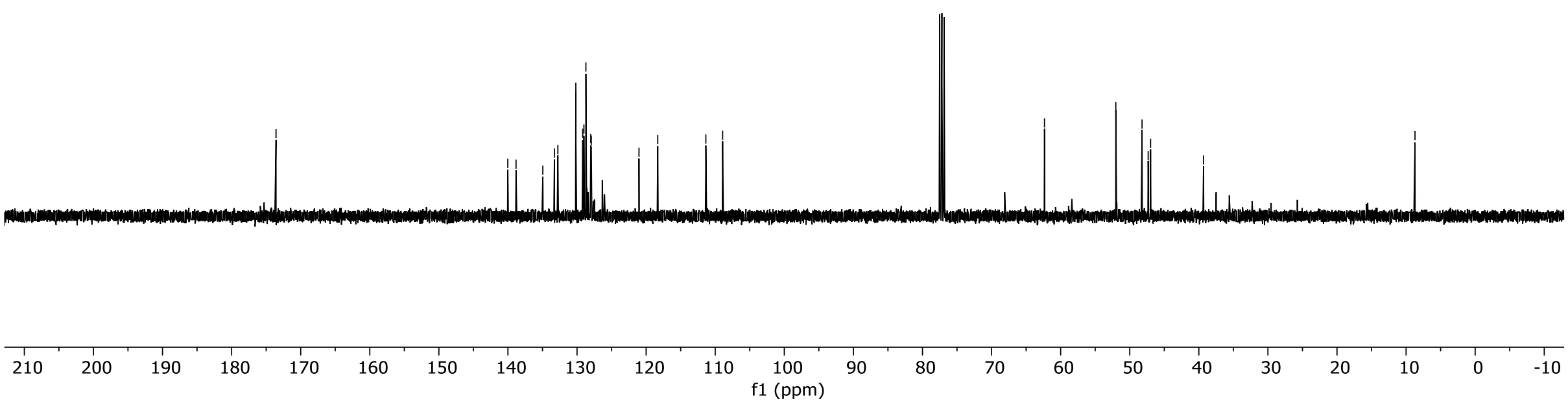

Methyl 9-(3,4-dichlorophenyl)-10-methyl-6-phenyl-6,7-dihydro-6,8-methanopyrido [1,2-a]indole-8(9*H*)-carboxylate (3i):

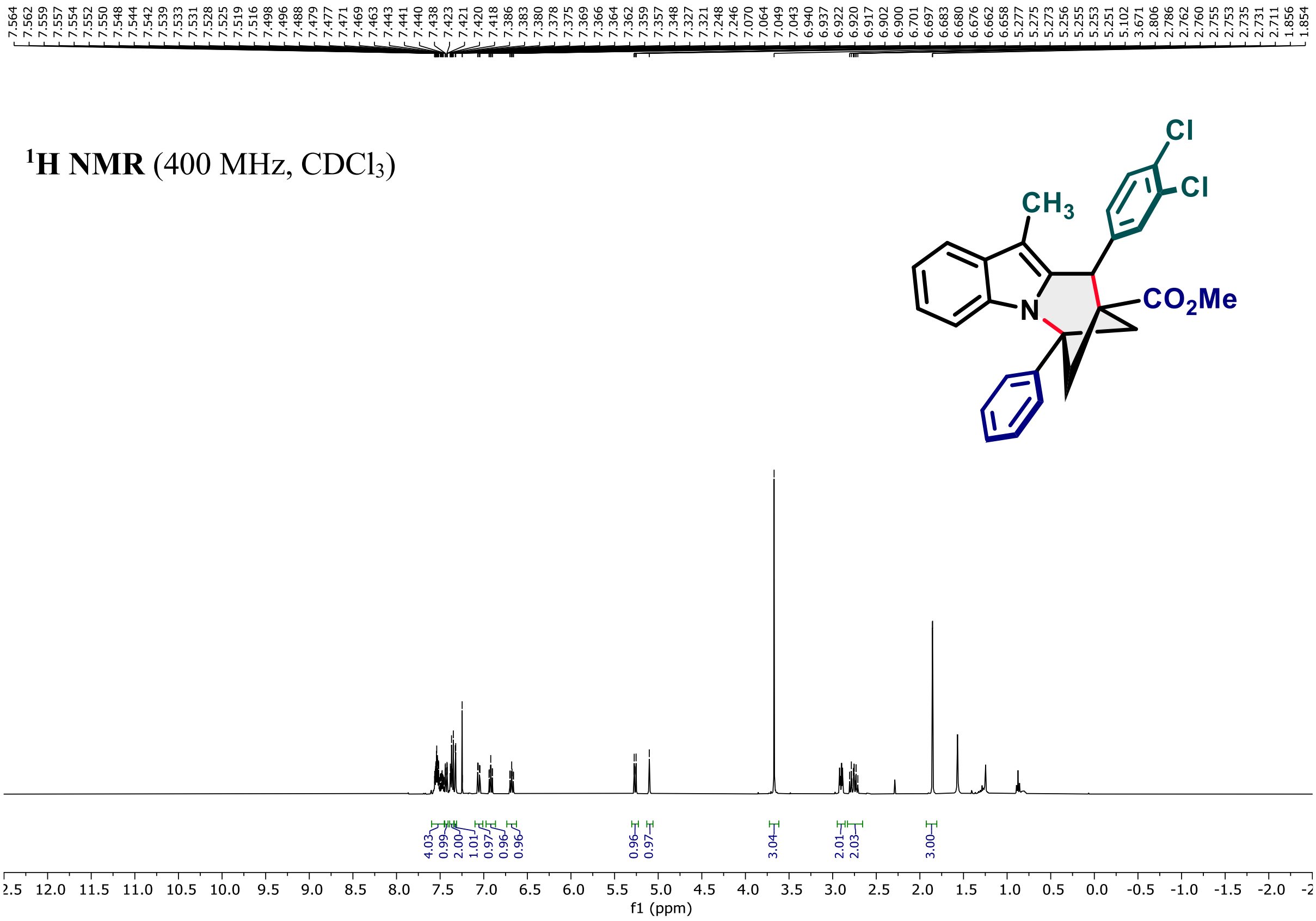

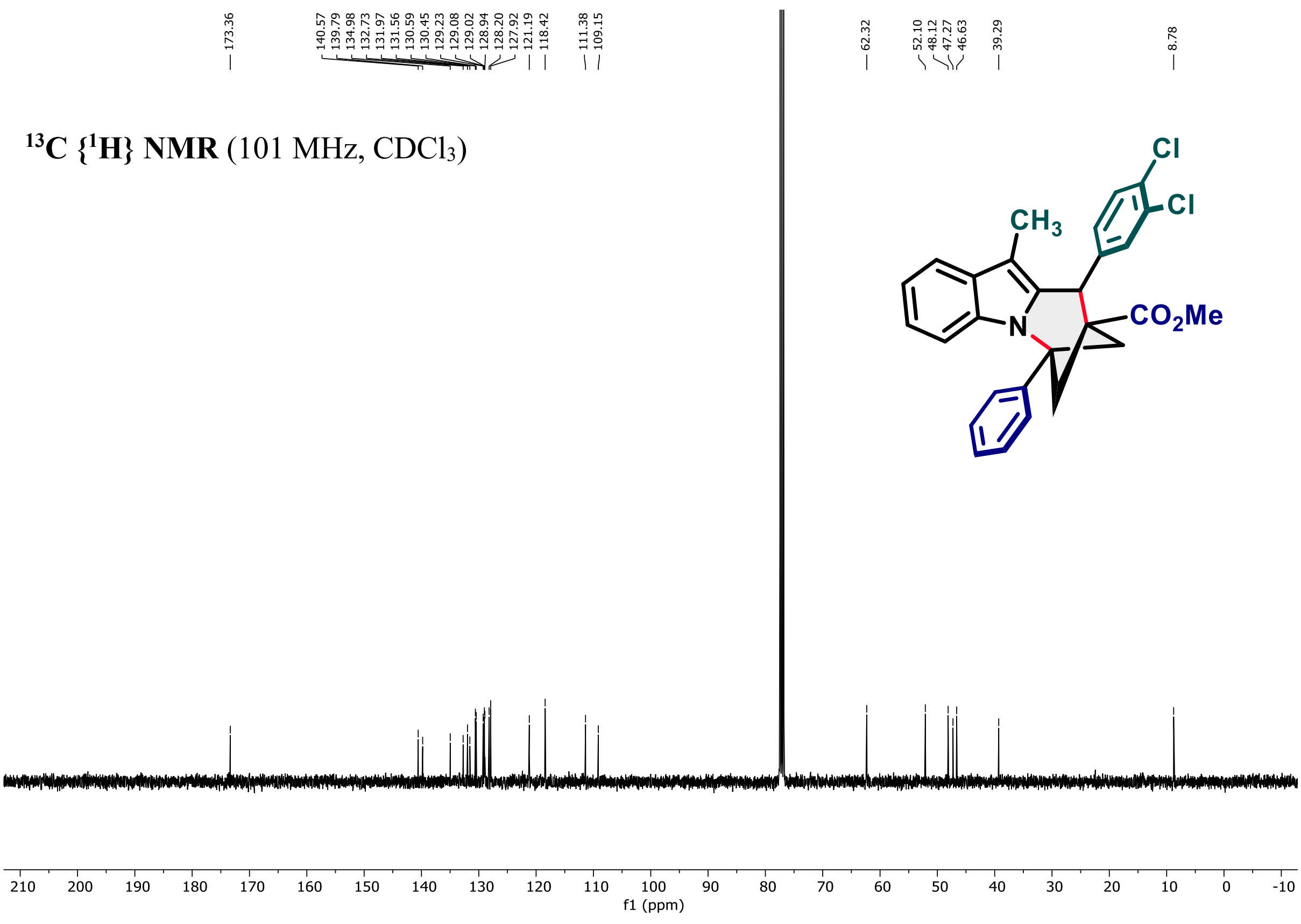

Methyl-10-methyl-6-phenyl-9-(4-(trifluoromethoxy)phenyl)-6,7-dihydro-6,8-methanopyrido[1,2-a]indole-8(9*H*)-carboxylate (3j):

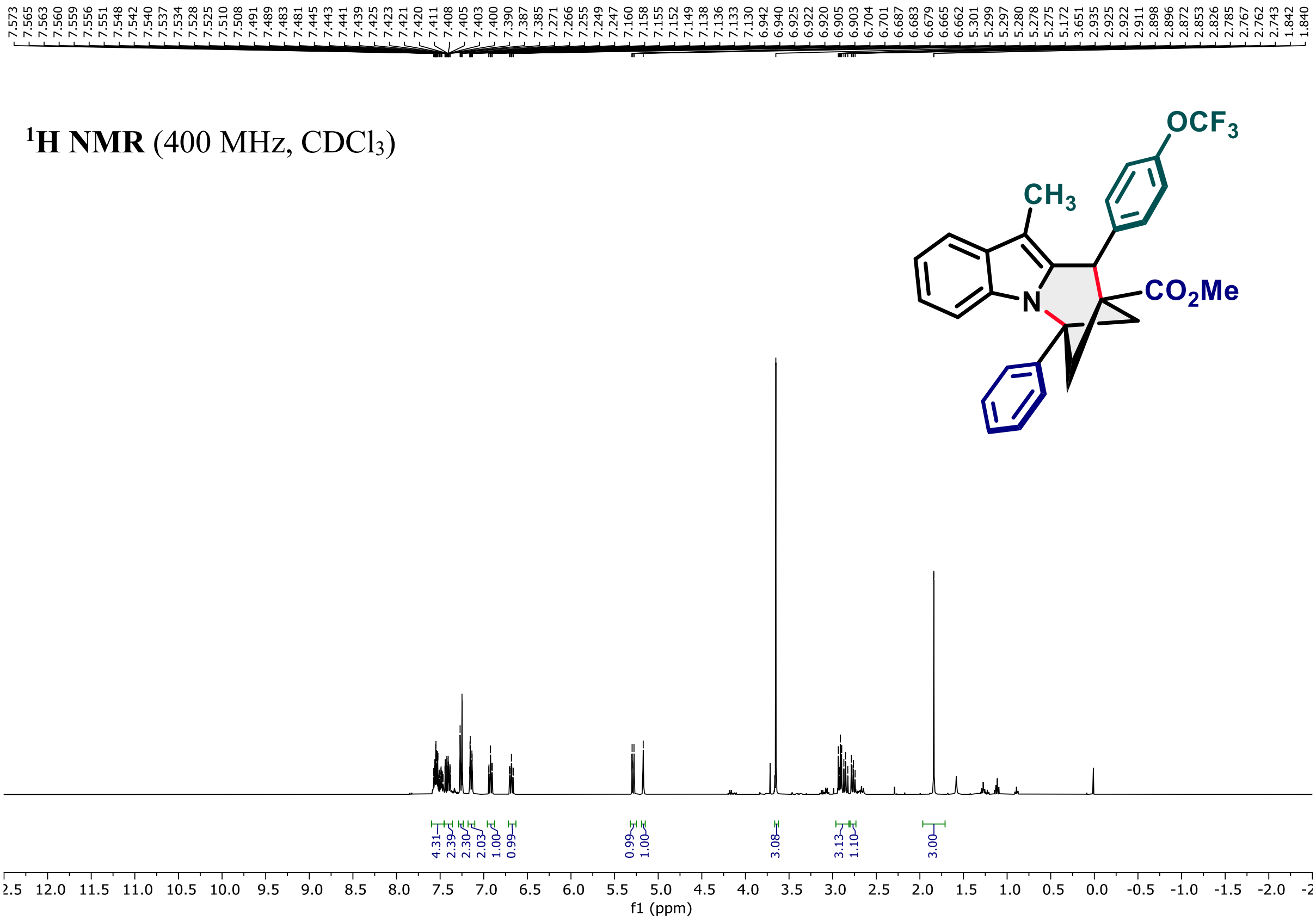

^{1}H NMR (400 MHz, $CDCl_3$)

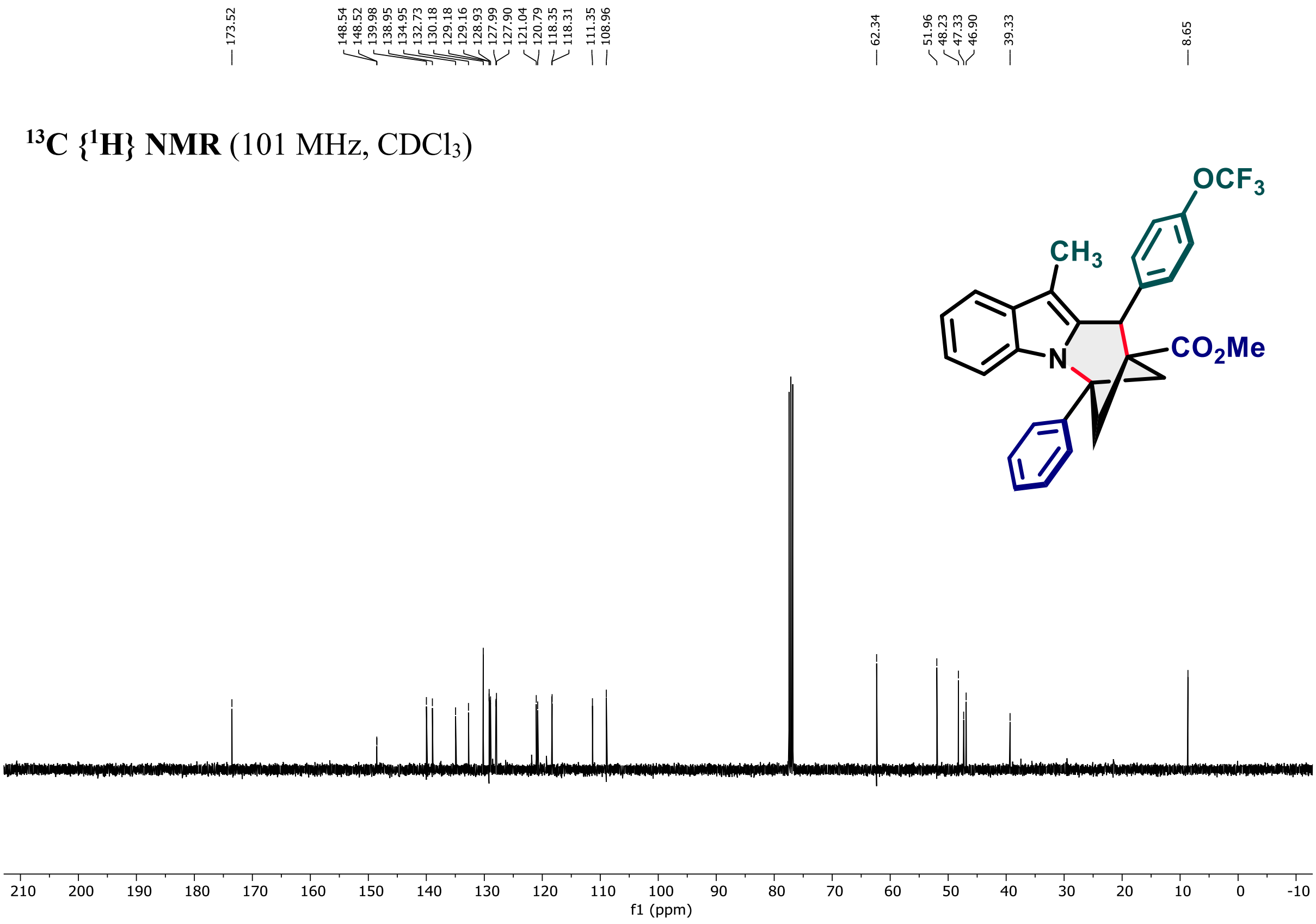

^{13}C {^{1}H} NMR (101 MHz, $CDCl_3$)

[19]**F NMR** (376 MHz, $CDCl_3$)

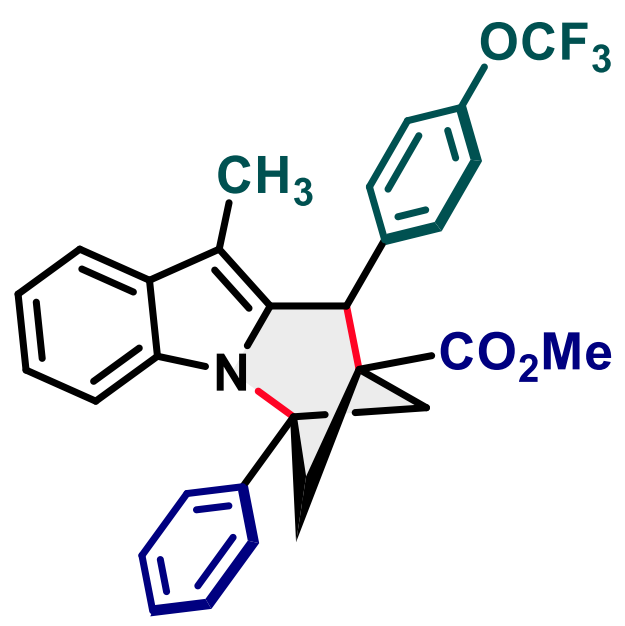

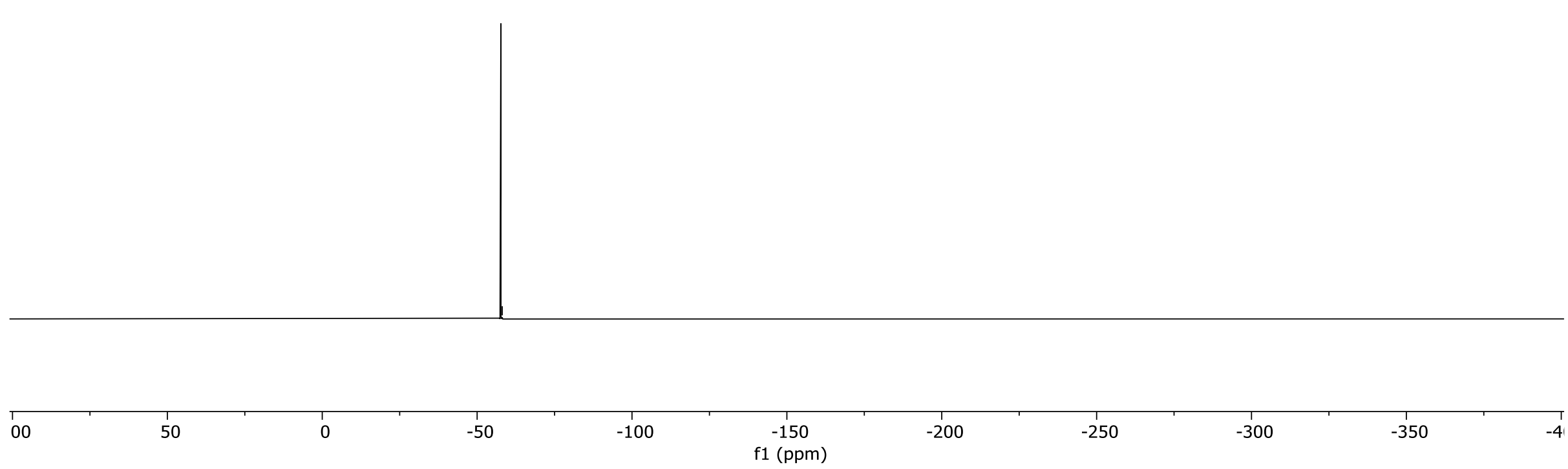

Methyl-10-methyl-6-phenyl-9-(4-(trifluoromethyl)phenyl)-6,7-dihydro-6,8-methano-pyrido[1,2-a]indole-8(9*H*)-carboxylate (3k):

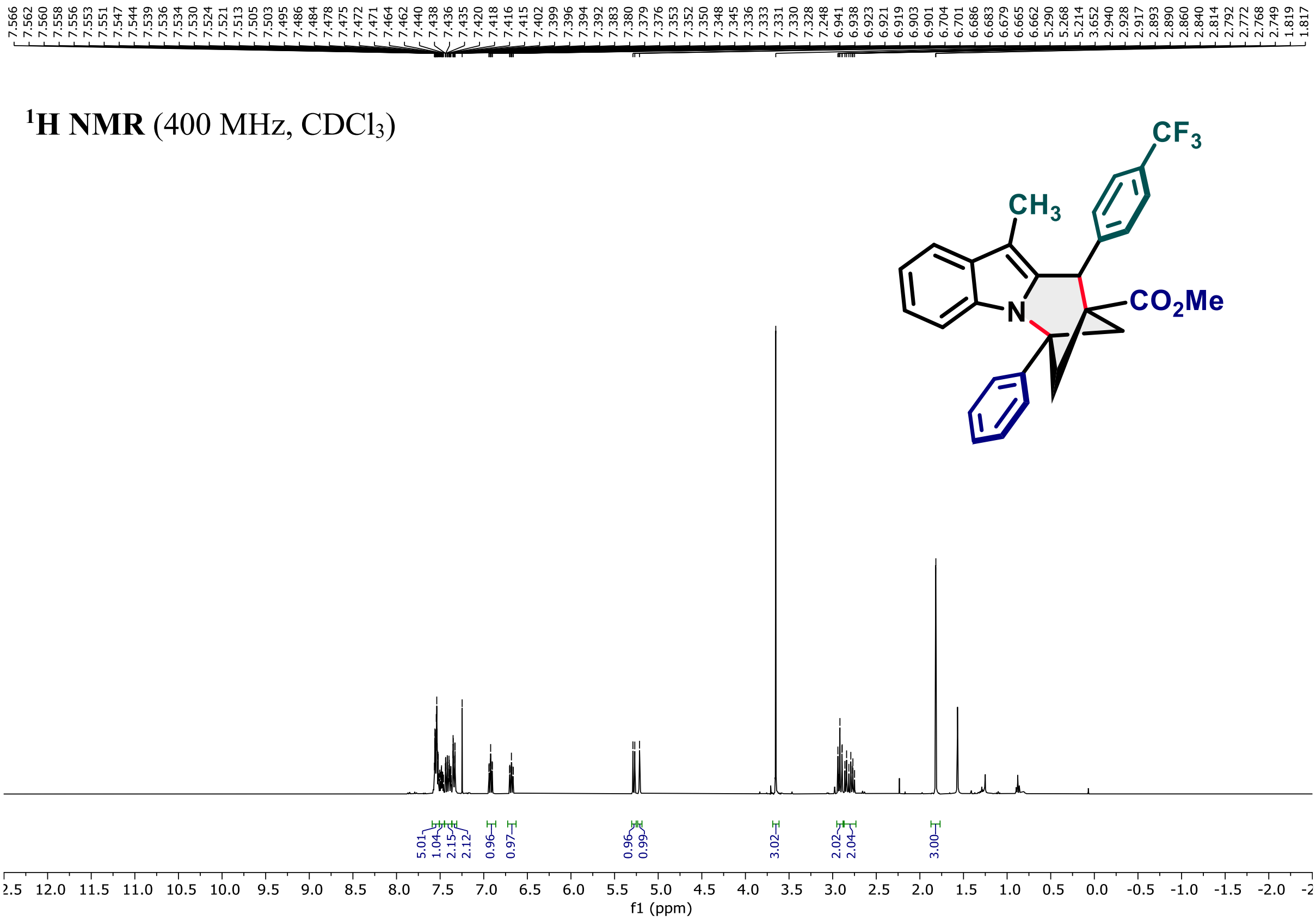

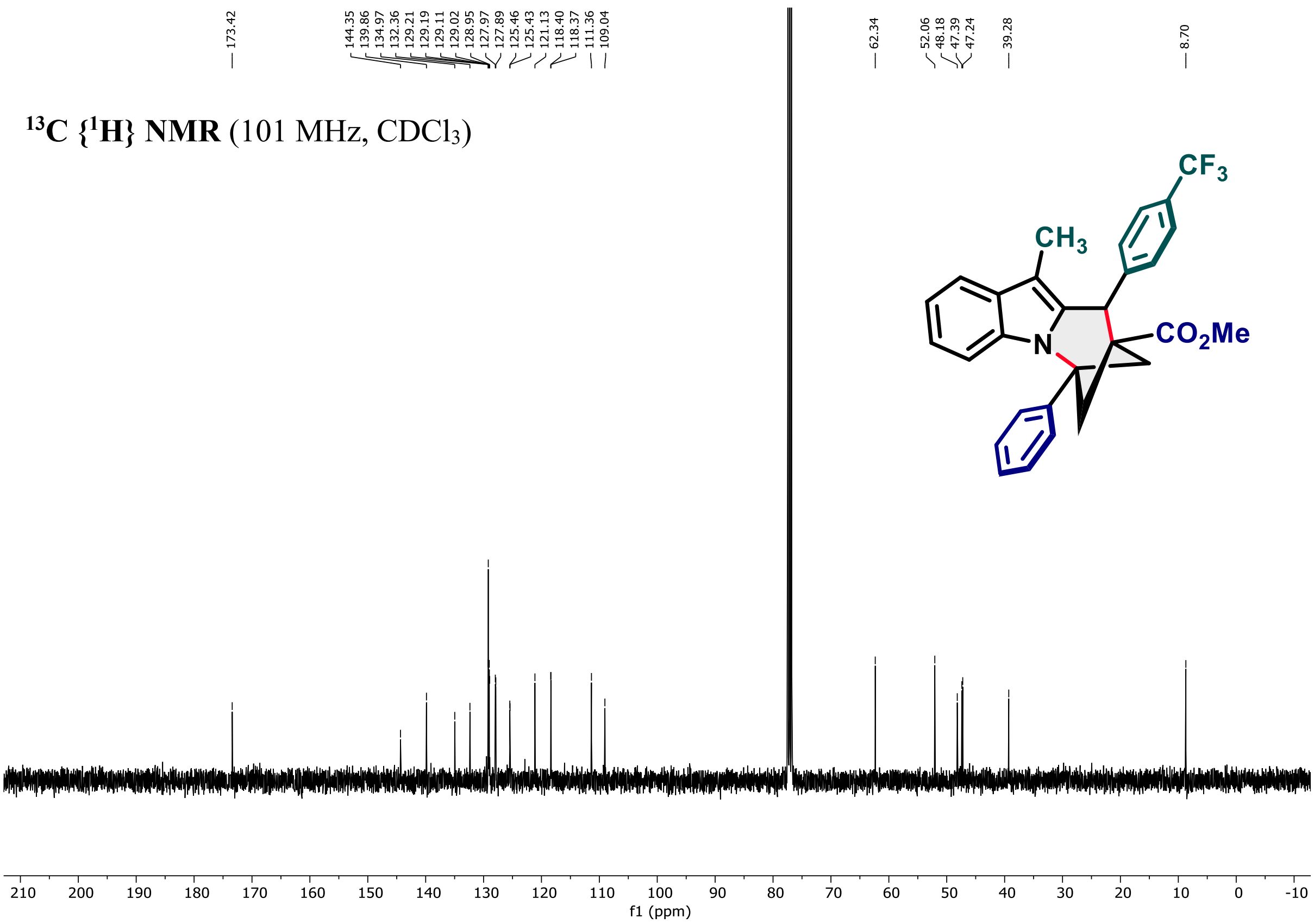

^{19}F NMR (376 MHz, $CDCl_3$)

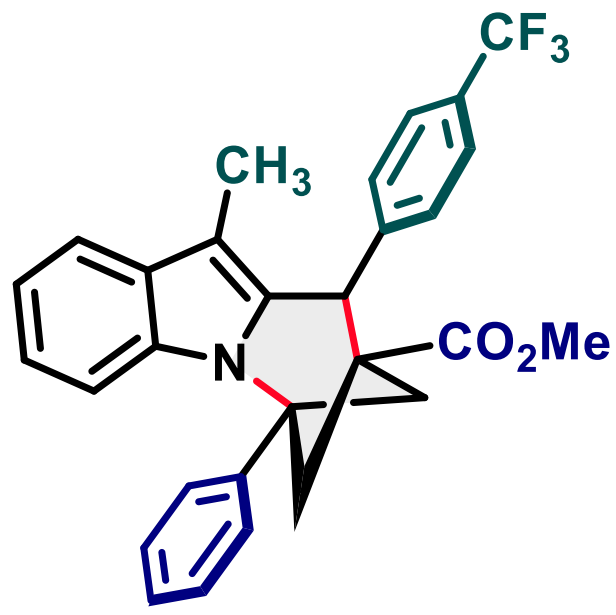

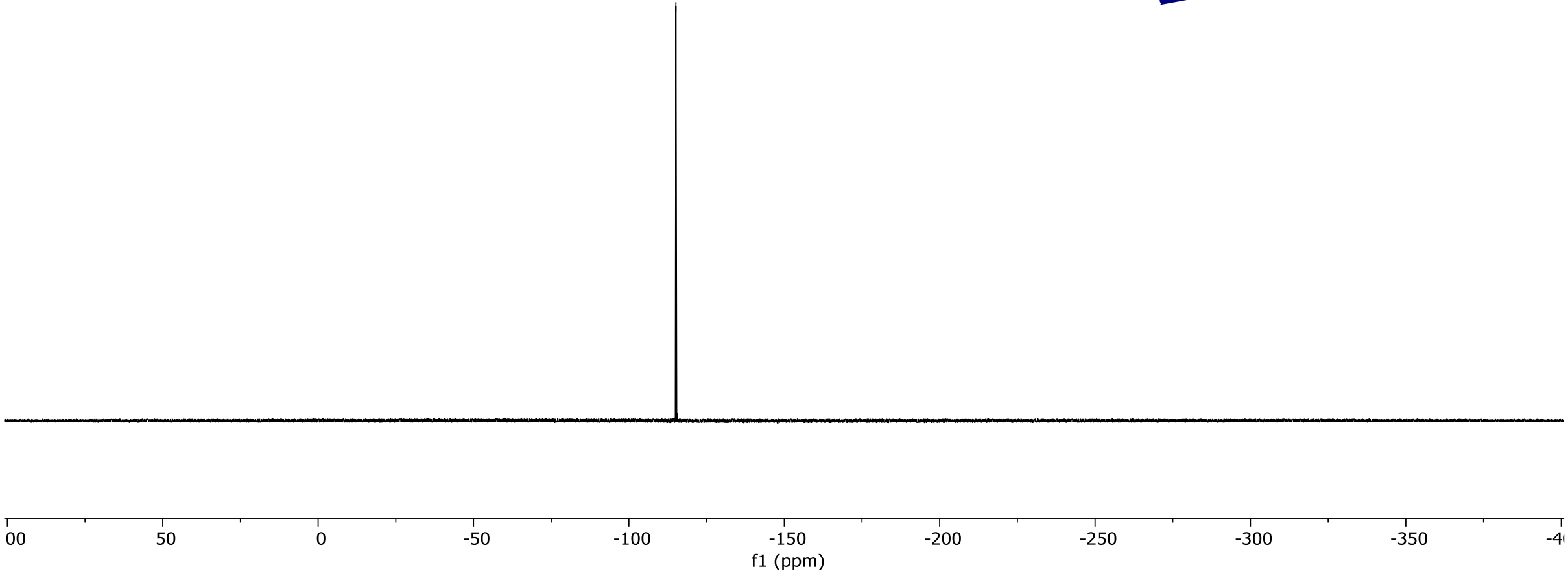

Methyl-10-methyl-6-phenyl-9-(thiophen-2-yl)-6,7-dihydro-6,8-methanopyrido[1,2-a]indole-8(9*H*)-carboxylate (3l):

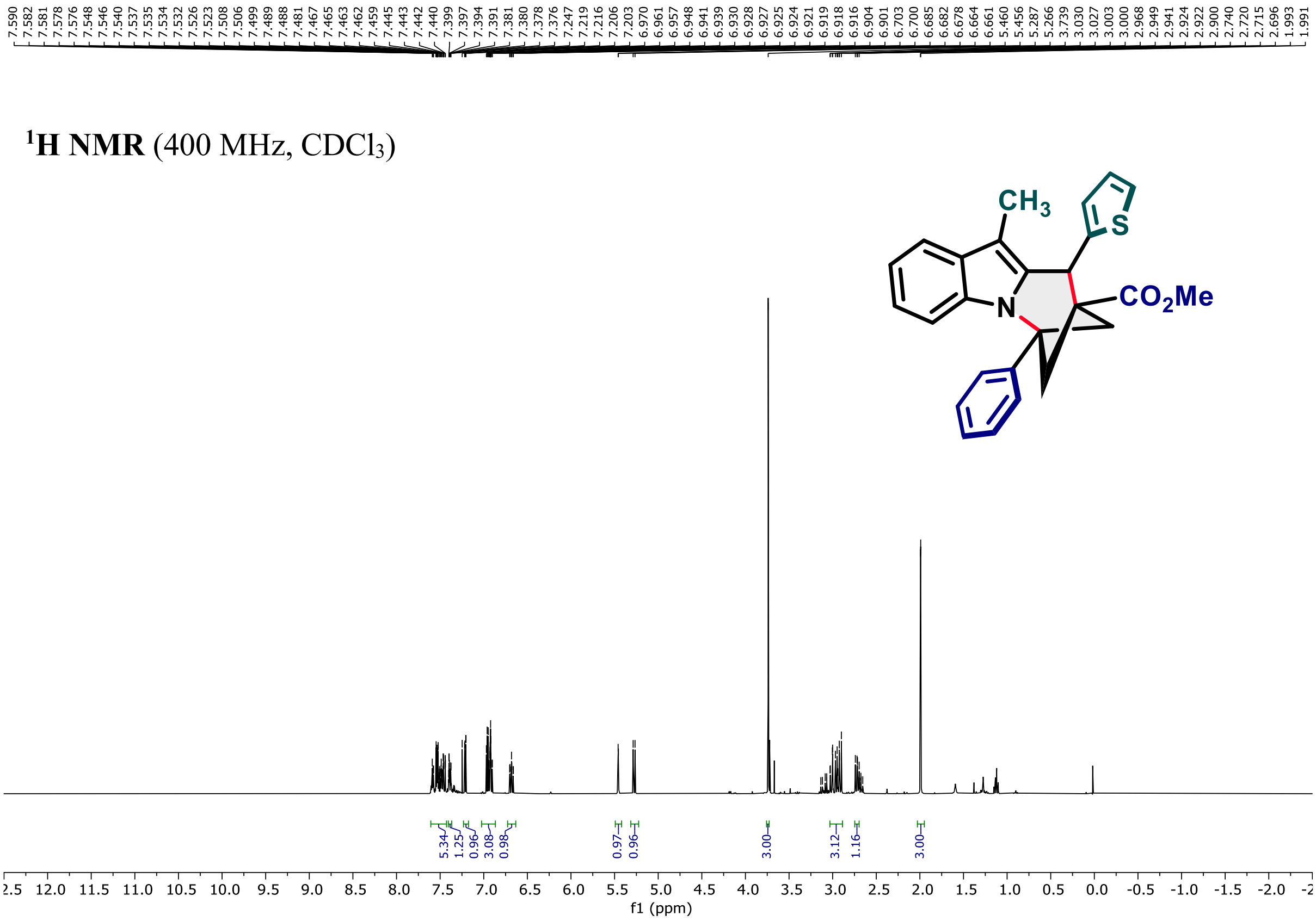

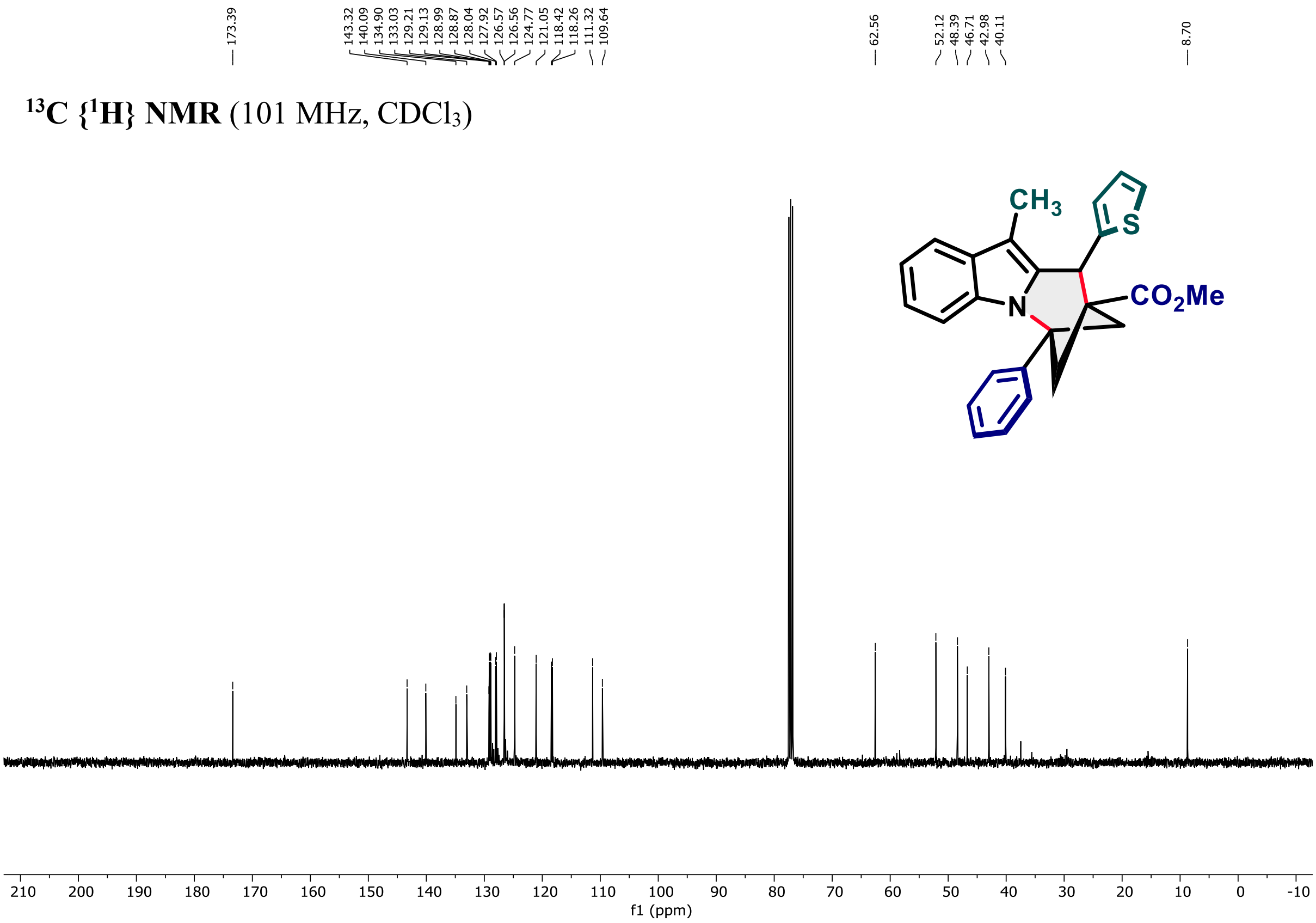

Methyl-6,9,10-triphenyl-6,7-dihydro-6,8-methanopyrido[1,2-a]indole-8(9*H*)-carboxylate (3m):

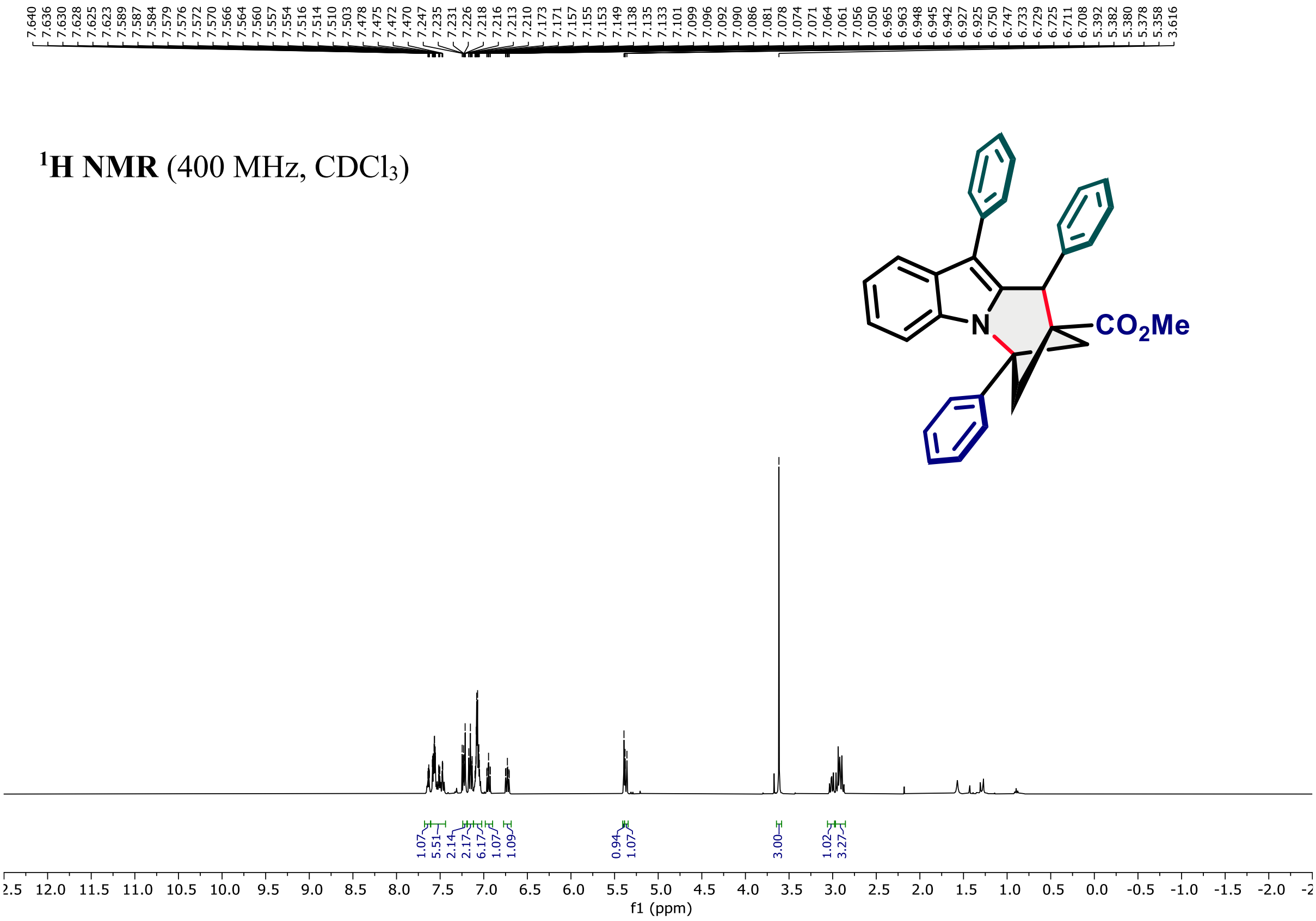

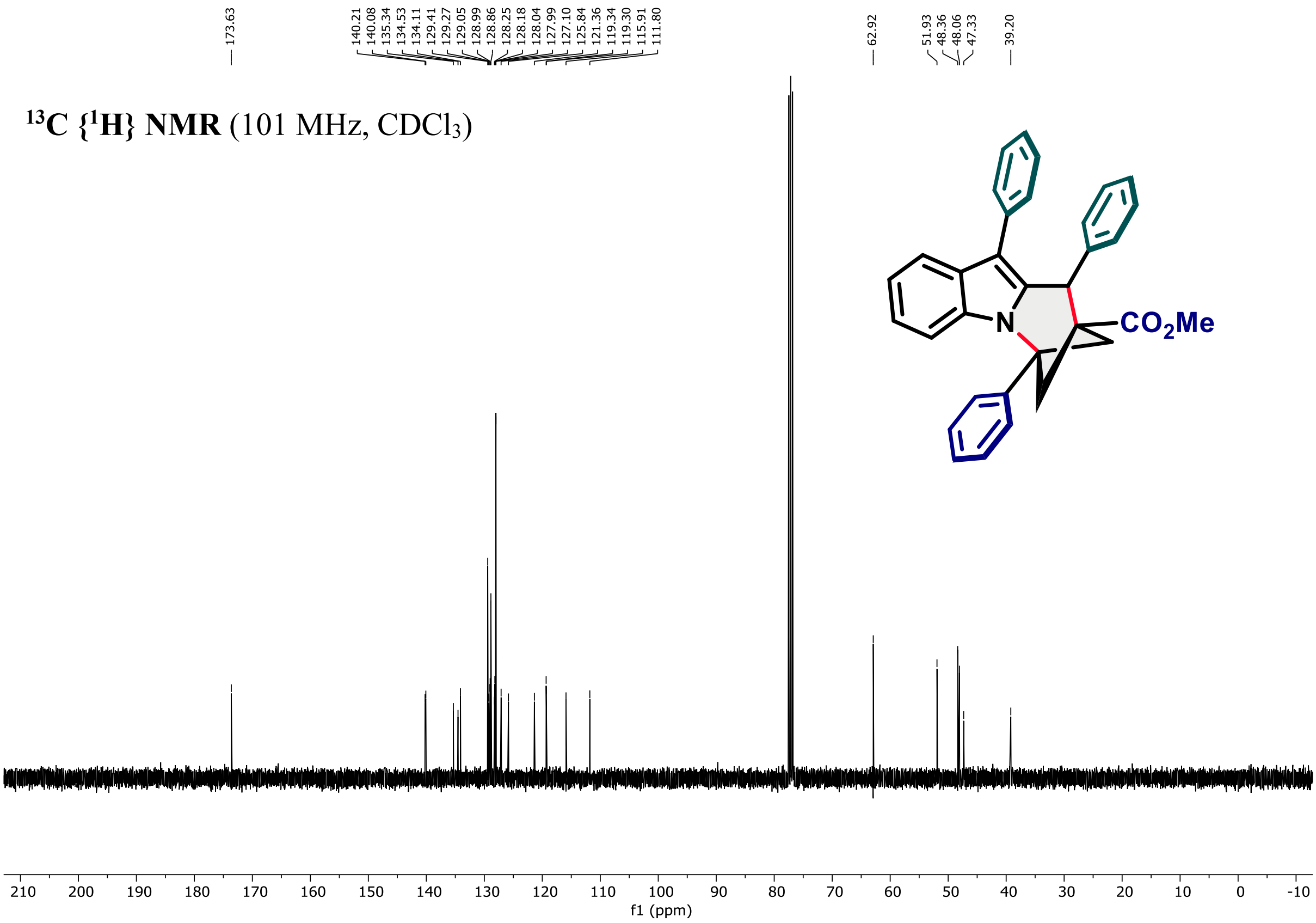

Methyl-10-(4-methoxyphenyl)-6,9-diphenyl-6,7-dihydro-6,8-methanopyrido[1,2-a]indole-8(9*H*)-carboxylate (3n):

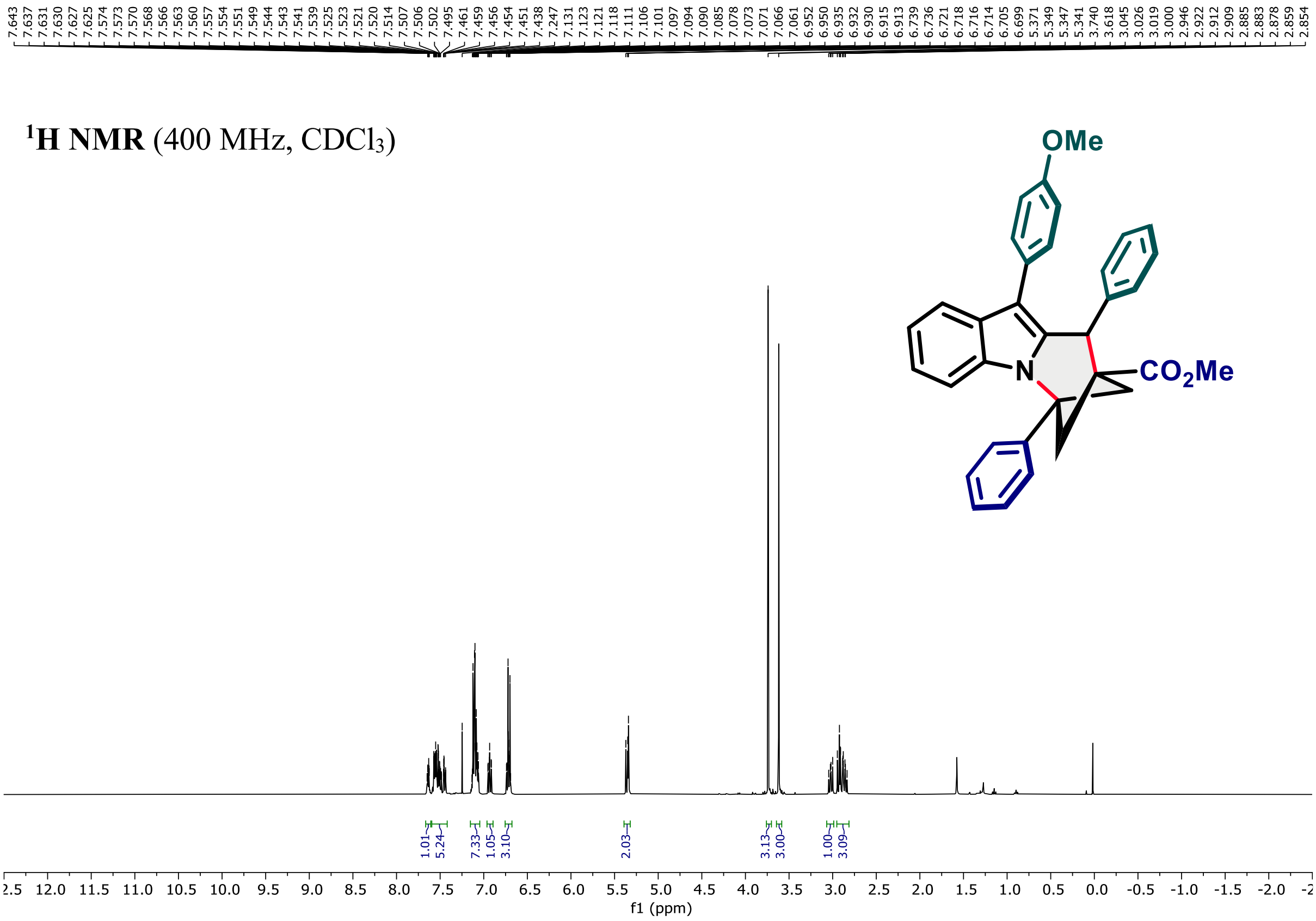

^{1}H NMR (400 MHz, $CDCl_3$)

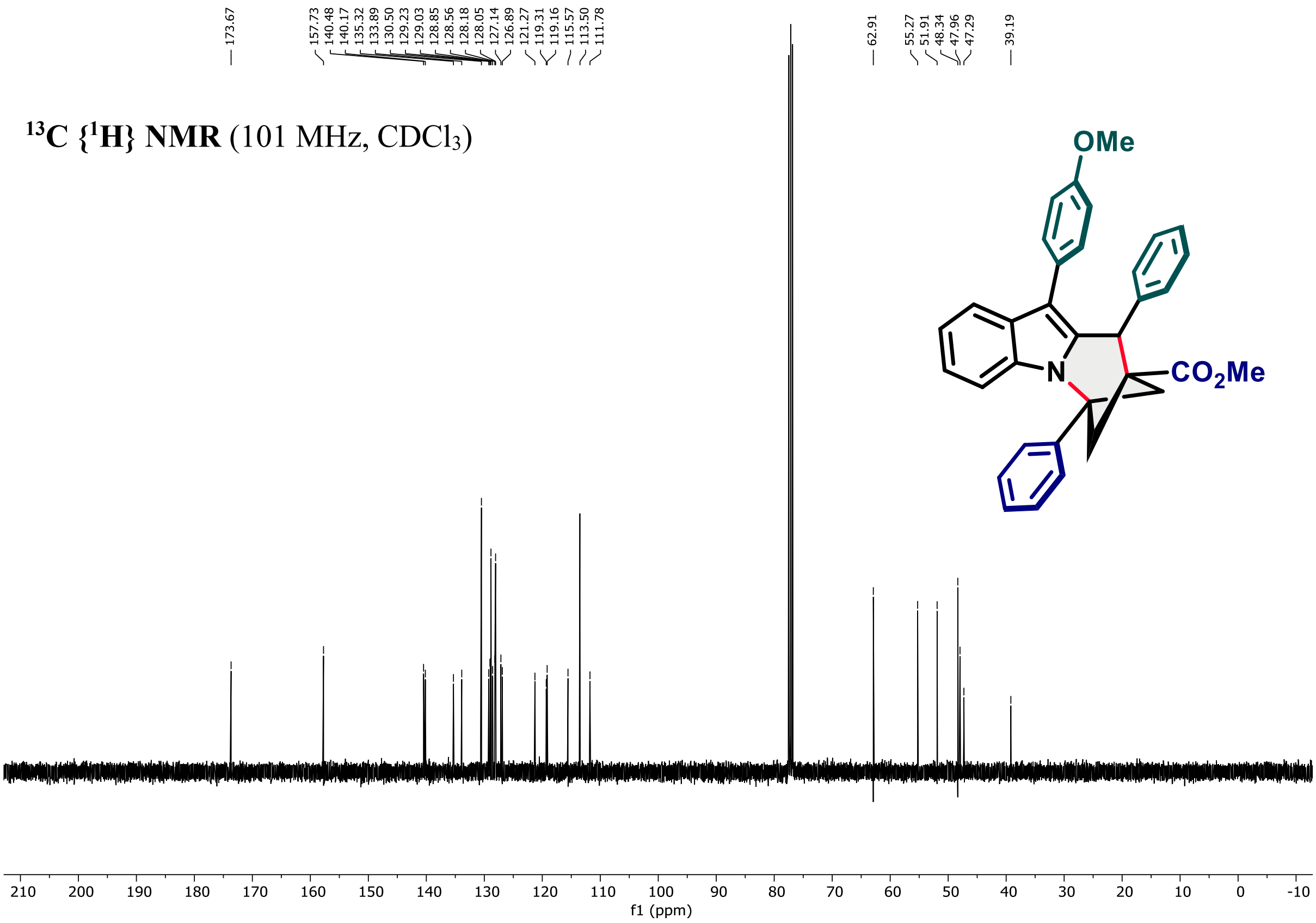

^{13}C {^{1}H} NMR (101 MHz, $CDCl_3$)

Methyl-10-(4-fluorophenyl)-6,9-diphenyl-6,7-dihydro-6,8-methanopyrido[1,2-a]indole-8(9*H*)-carboxylate (3o):

^{1}H NMR (400 MHz, $CDCl_3$)

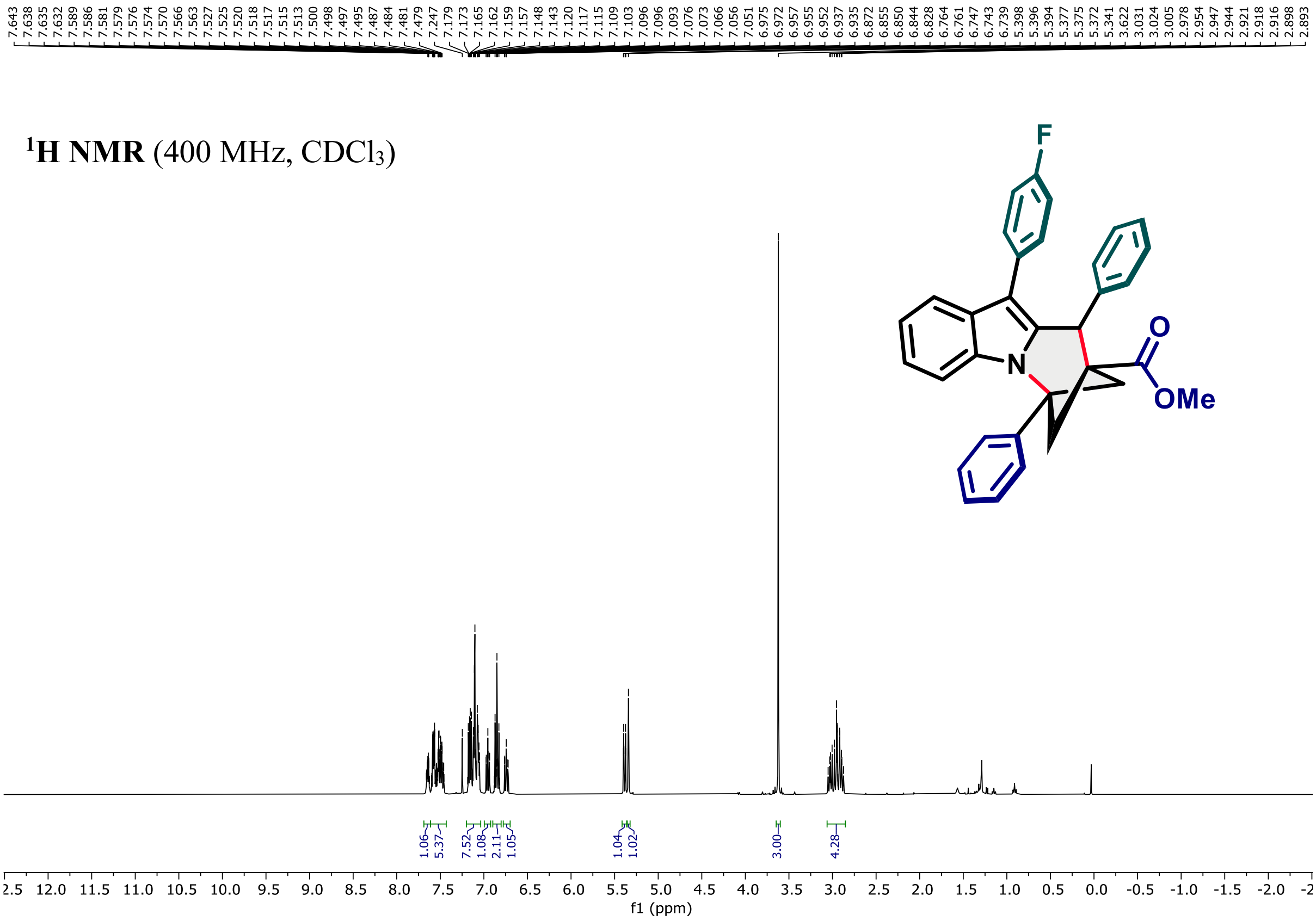

^{13}C {^{1}H} NMR (101 MHz, $CDCl_3$)

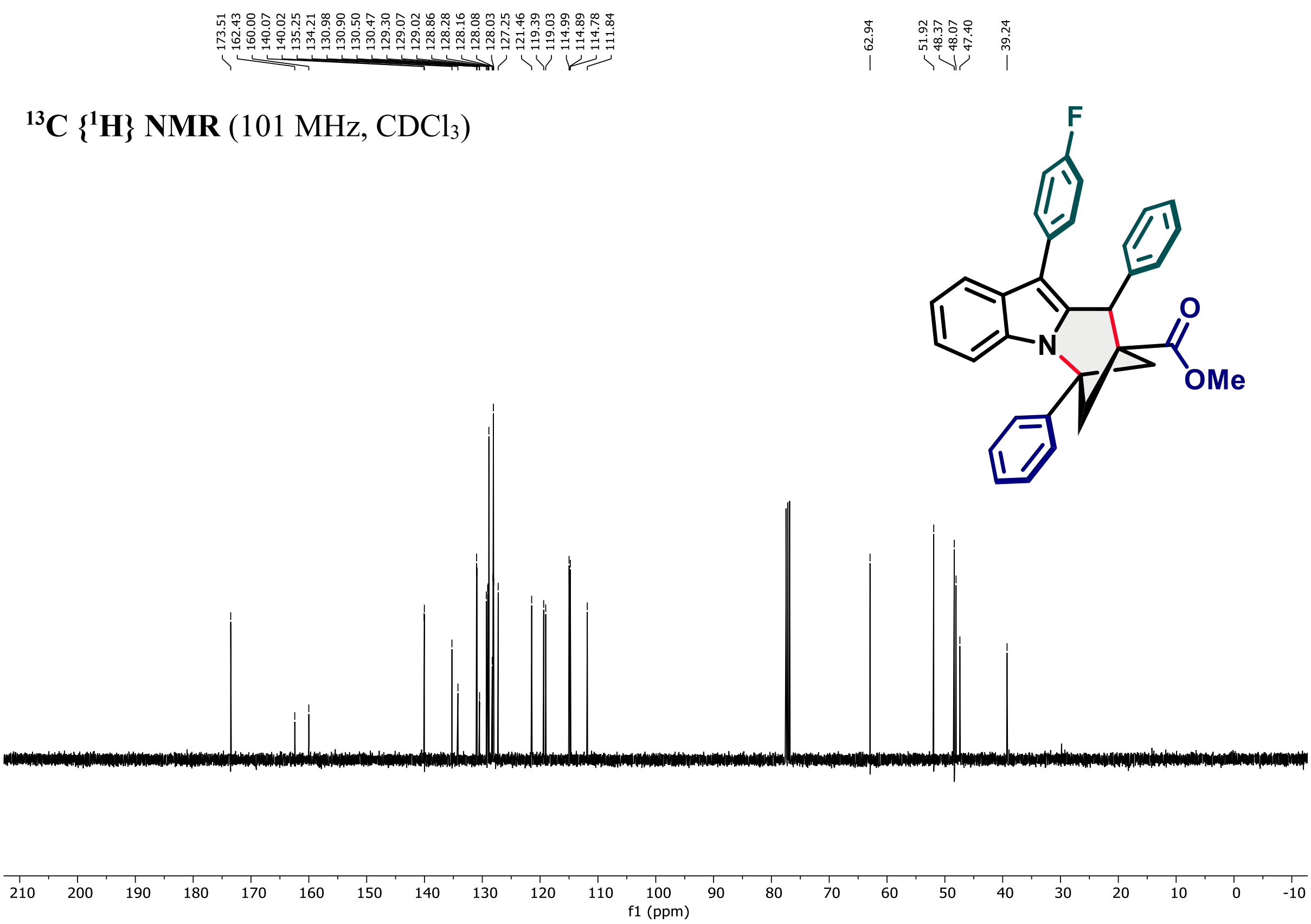

^{19}F NMR (376 MHz, $CDCl_3$)

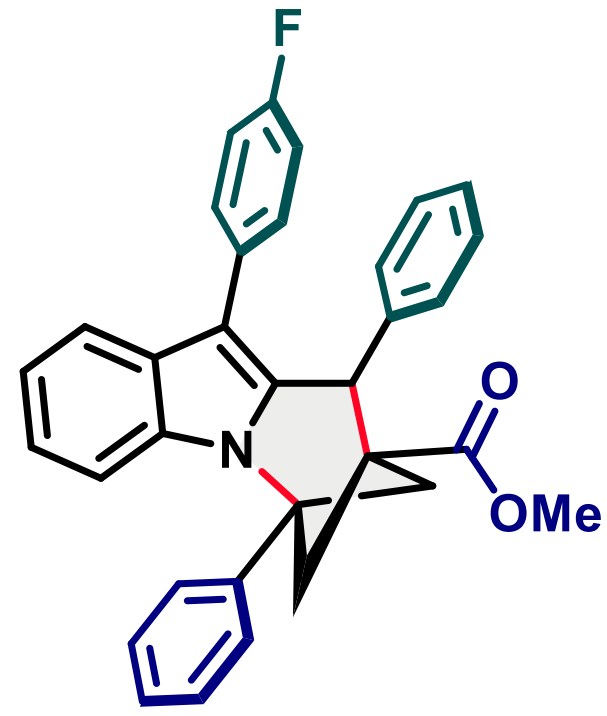

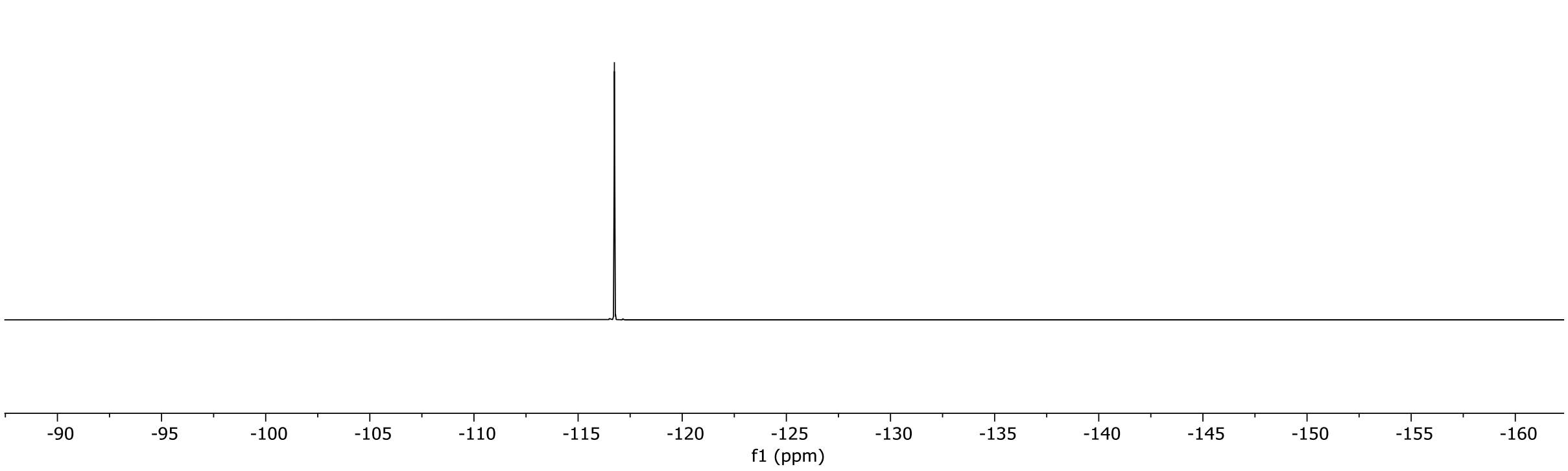

Methyl-10-(4-chlorophenyl)-6,9-diphenyl-6,7-dihydro-6,8-methanopyrido[1,2-a]indole-8(9*H*)-carboxylate (3p):

^{1}H NMR (400 MHz, $CDCl_3$)

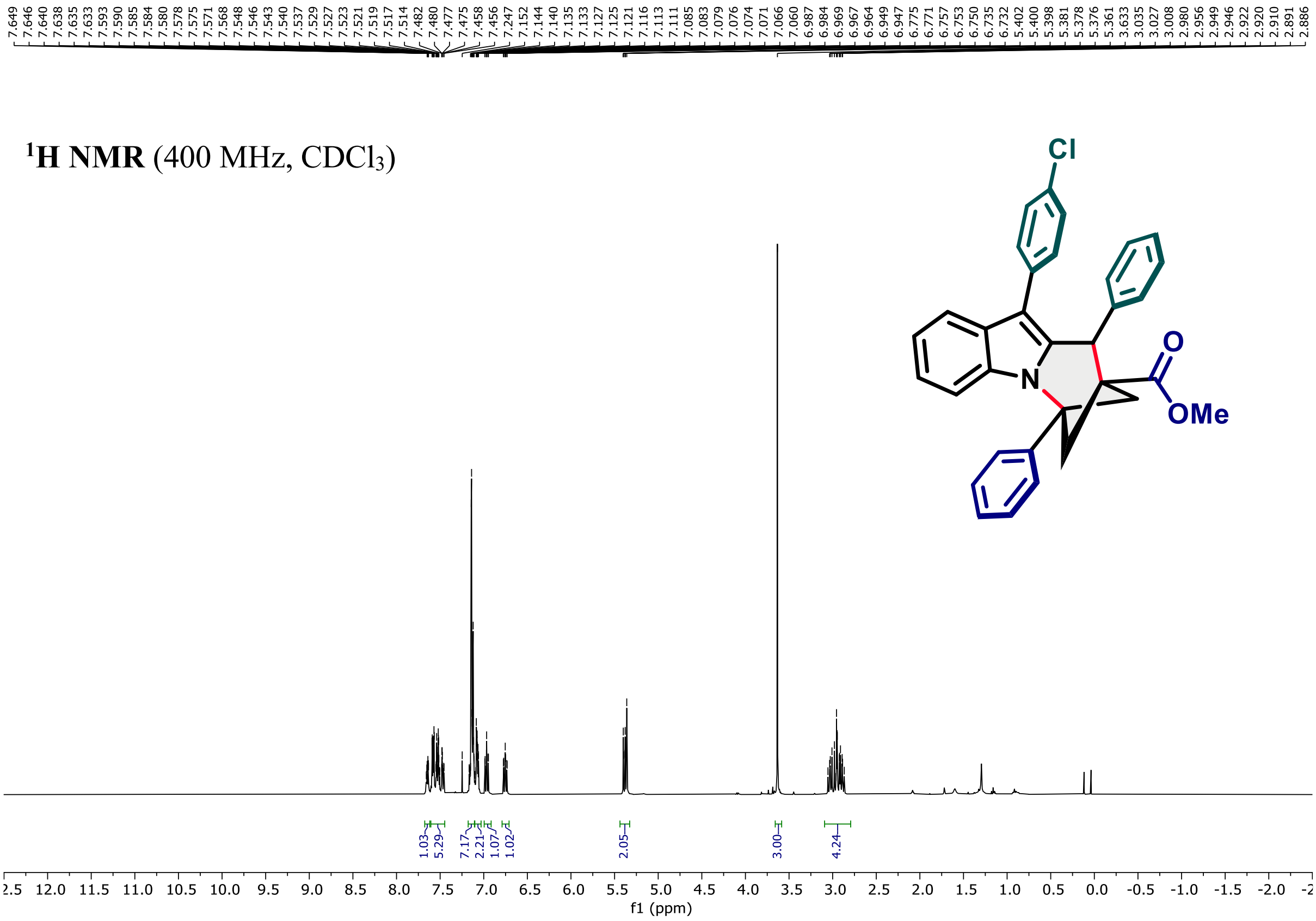

^{13}C {^{1}H} NMR (101 MHz, $CDCl_3$)

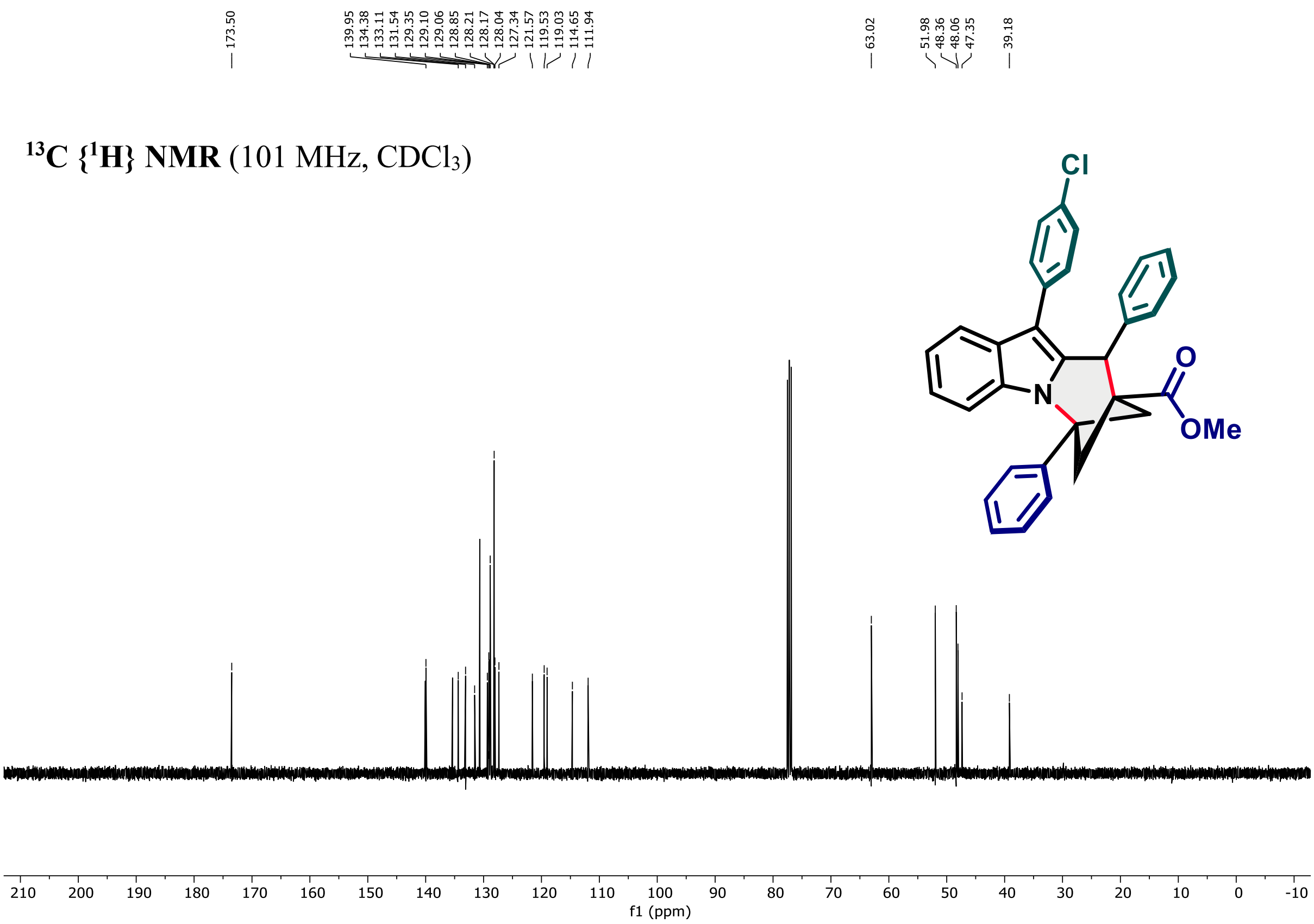

Methyl-10-(naphthalen-2-yl)-6,9-diphenyl-6,7-dihydro-6,8-methanopyrido[1,2-a]indole-8(9*H*)-carboxylate (3q):

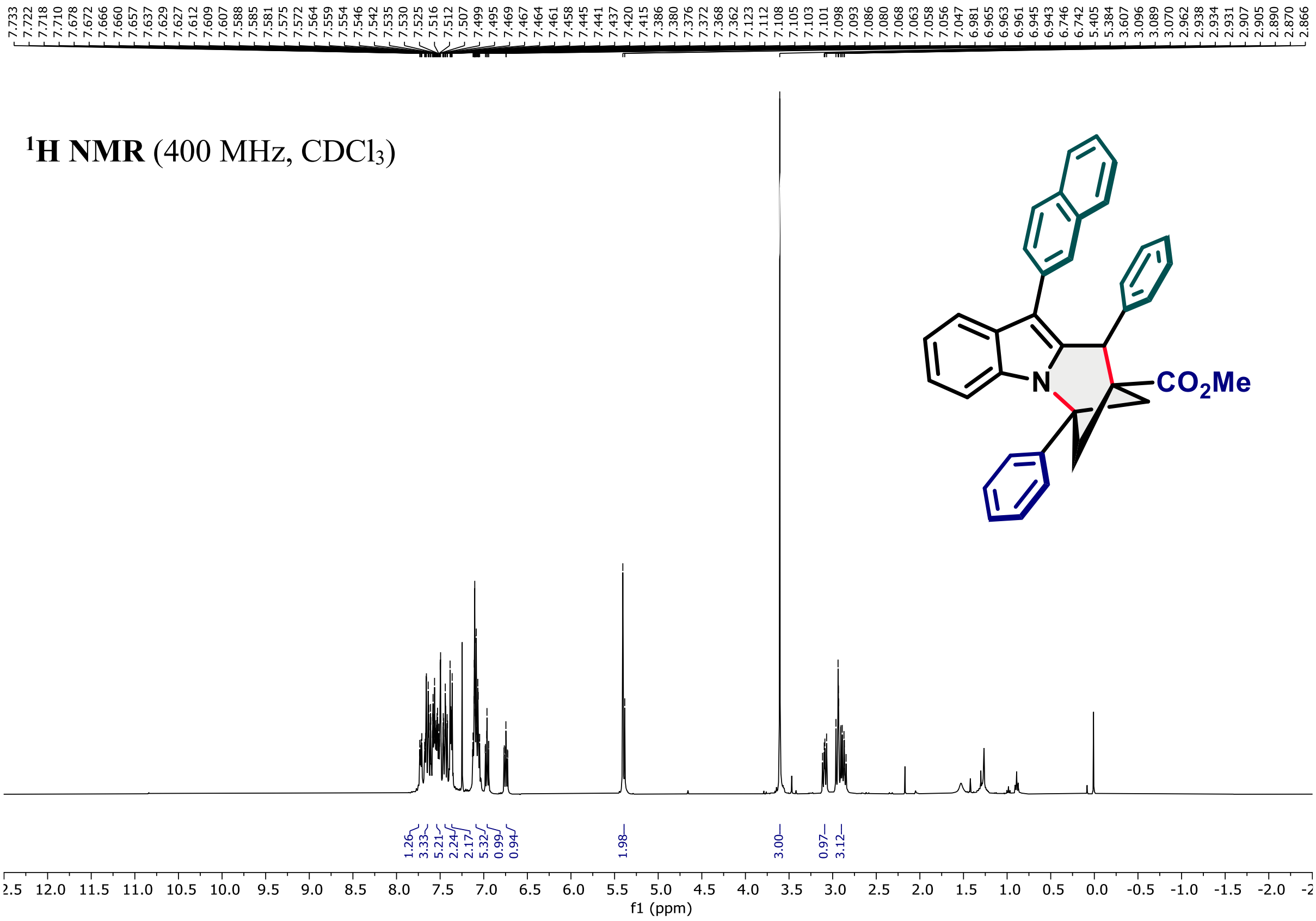

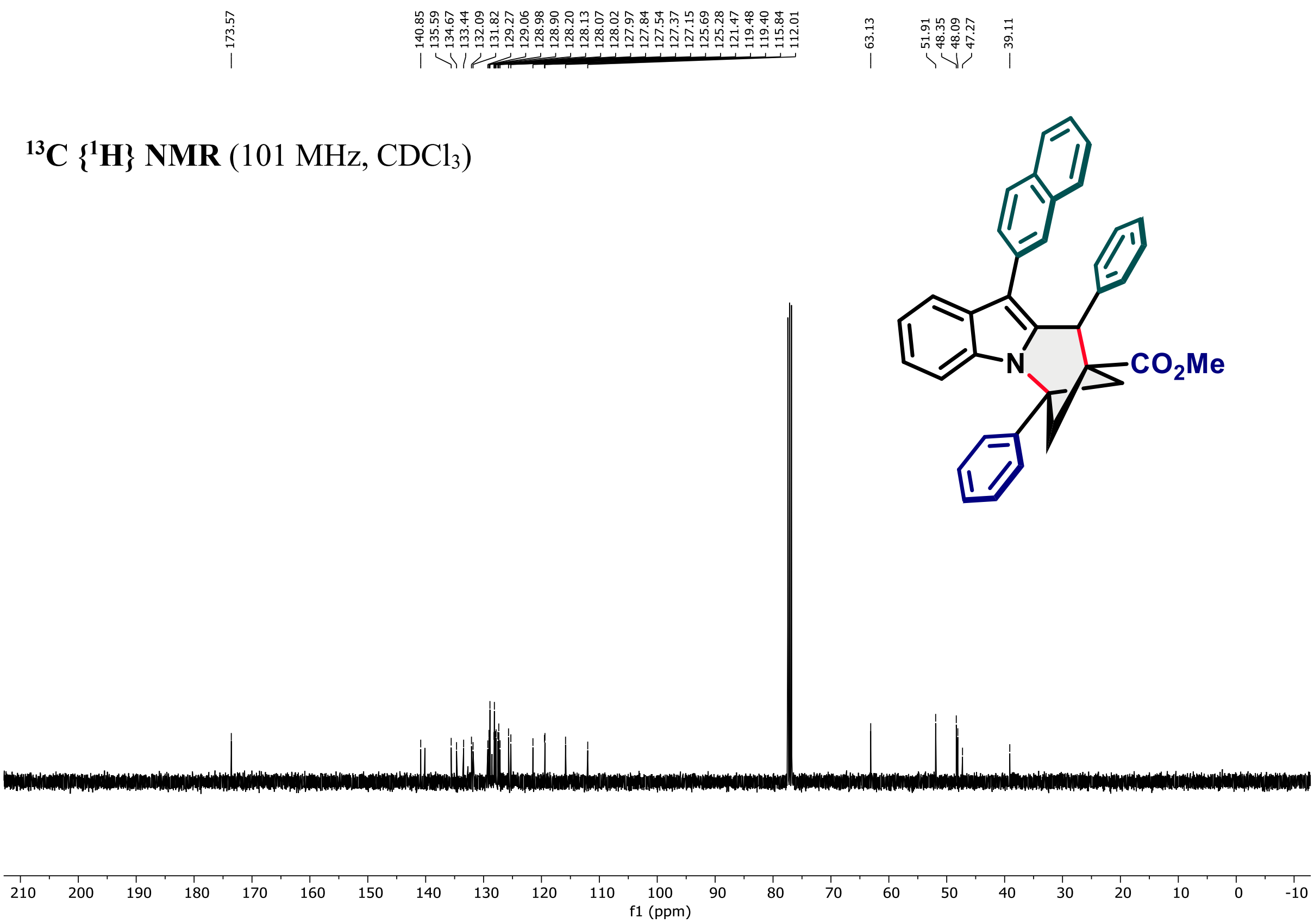

Methyl-2-methoxy-6,9,10-triphenyl-6,7-dihydro-6,8-methanopyrido[1,2-a]indole-8(9*H*)-carboxylatecarboxylate (3r):

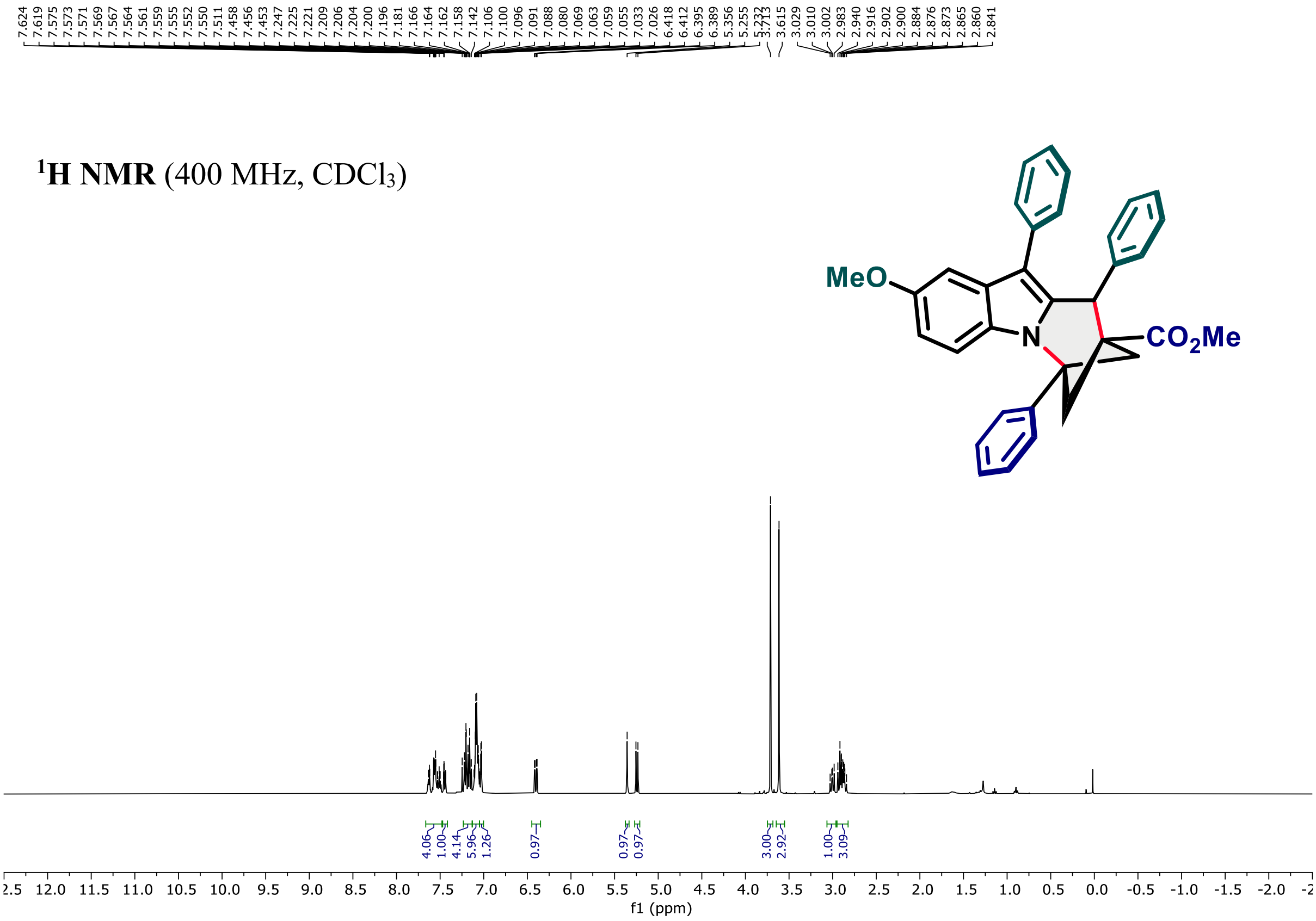

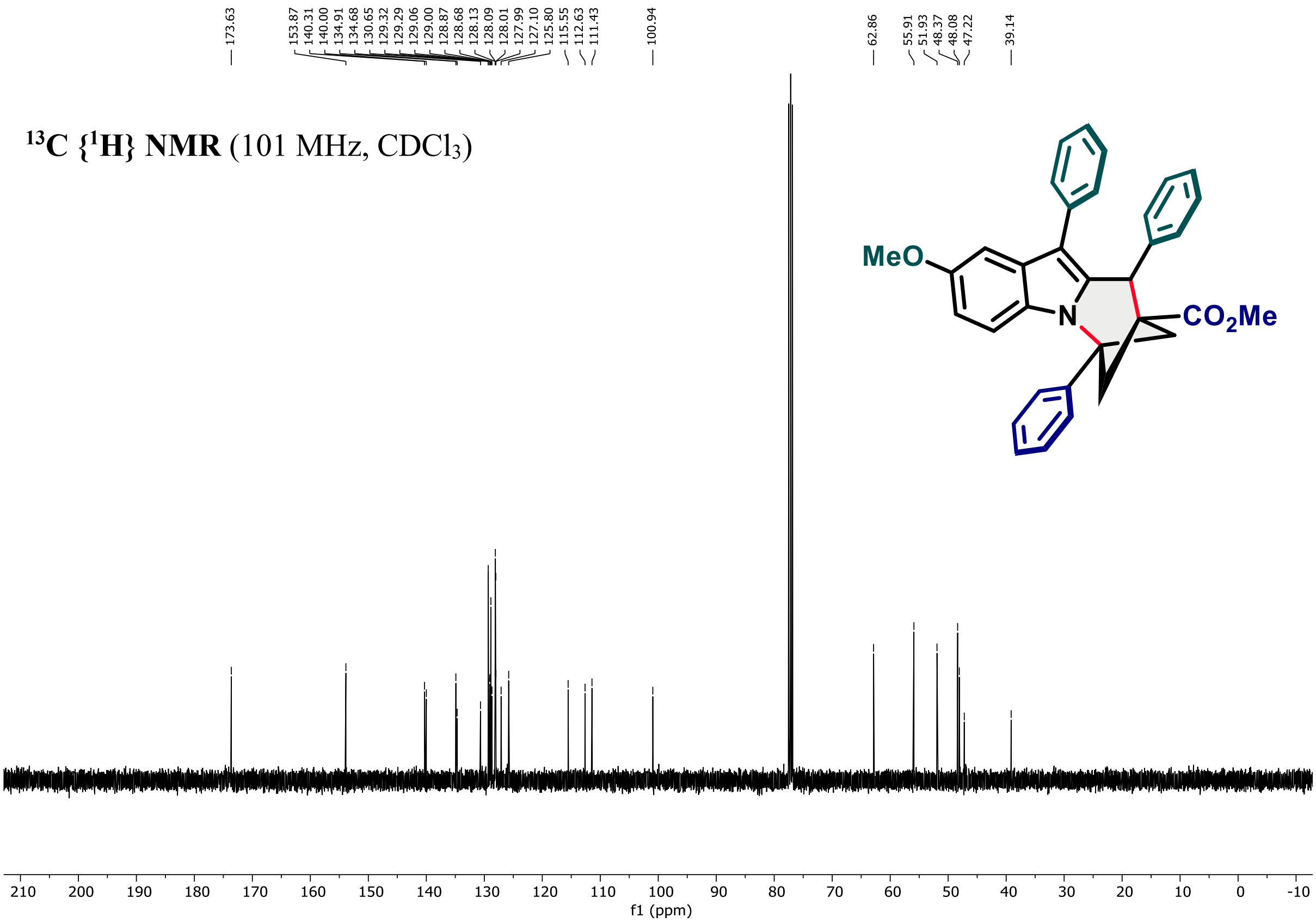

Methyl 2-fluoro-6,9,10-triphenyl-6,7-dihydro-6,8-methanopyrido[1,2-a]indole-8(9*H*)-carboxylate (3s):

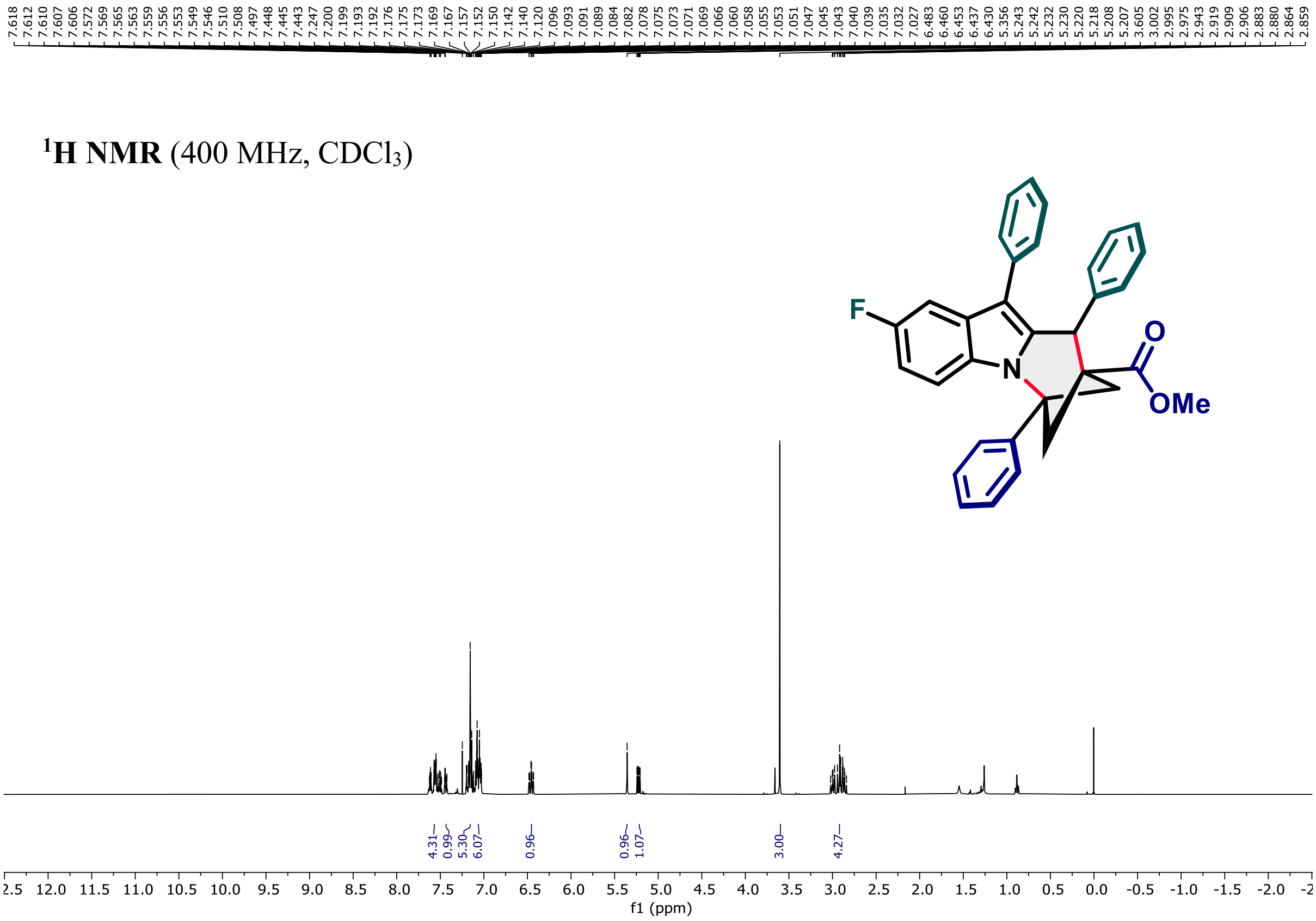

^{1}H NMR (400 MHz, $CDCl_3$)

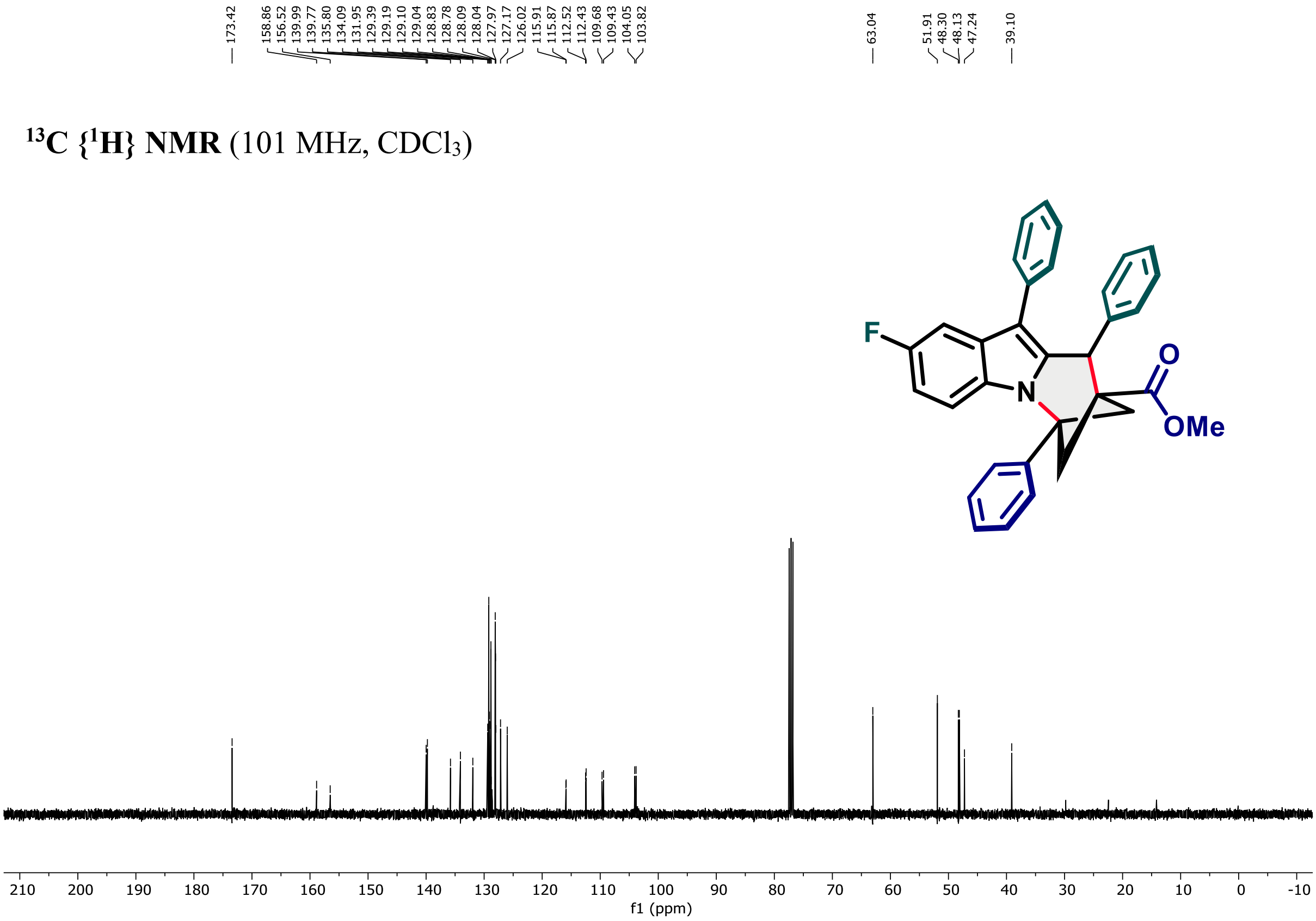

^{13}C {^{1}H} NMR (101 MHz, $CDCl_3$)

^{19}F NMR (376 MHz, $CDCl_3$)

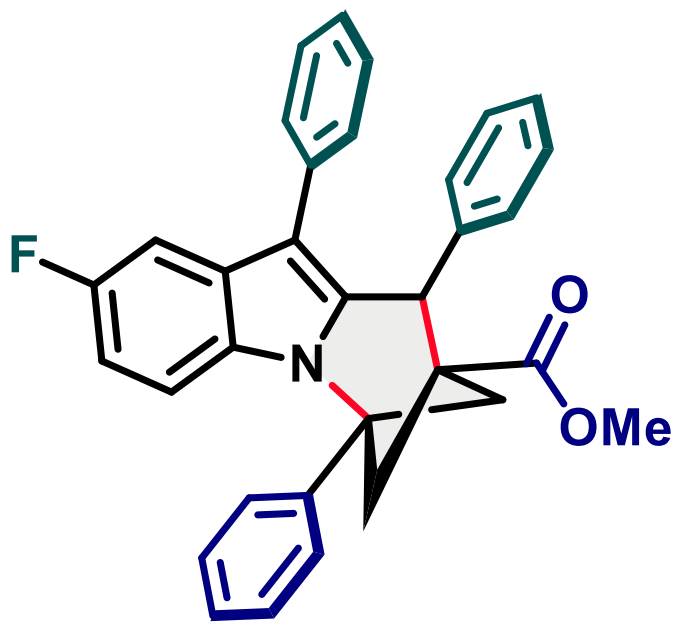

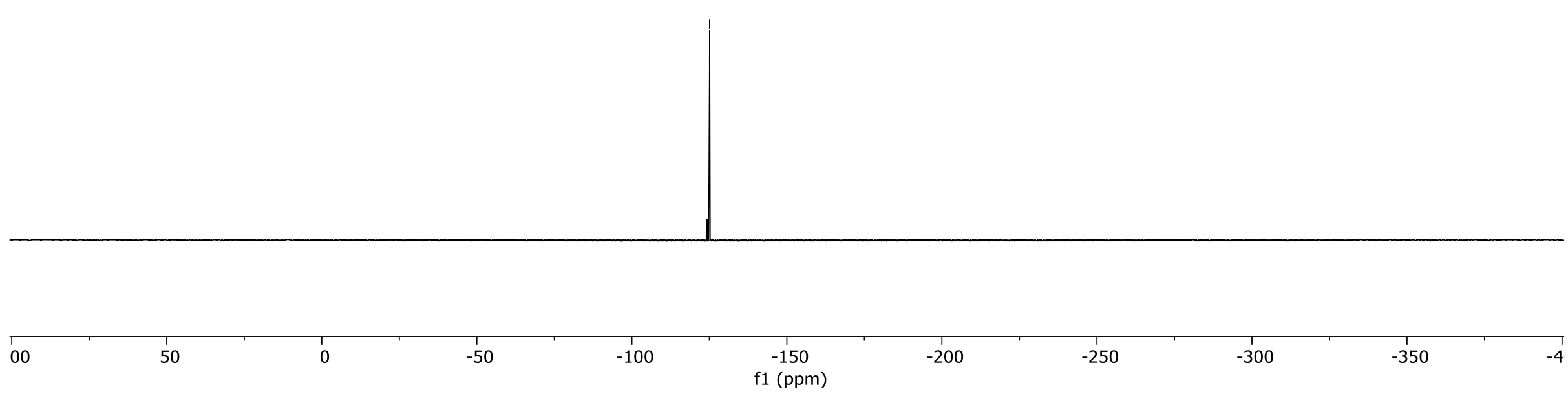

Methyl-2-bromo-6,9,10-triphenyl-6,7-dihydro-6,8-methanopyrido[1,2-a]indole-8(9*H*)-carboxylate (3t):

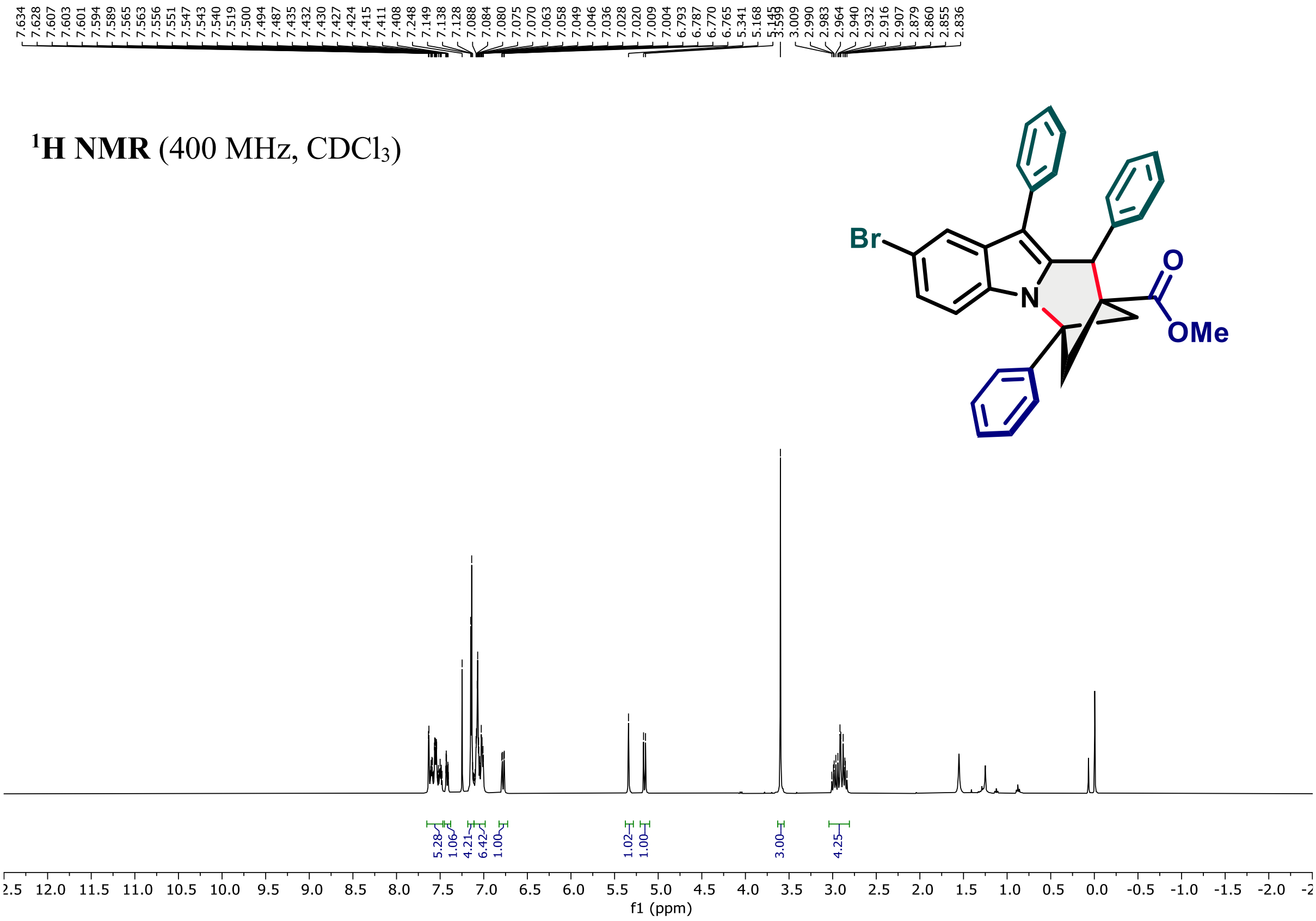

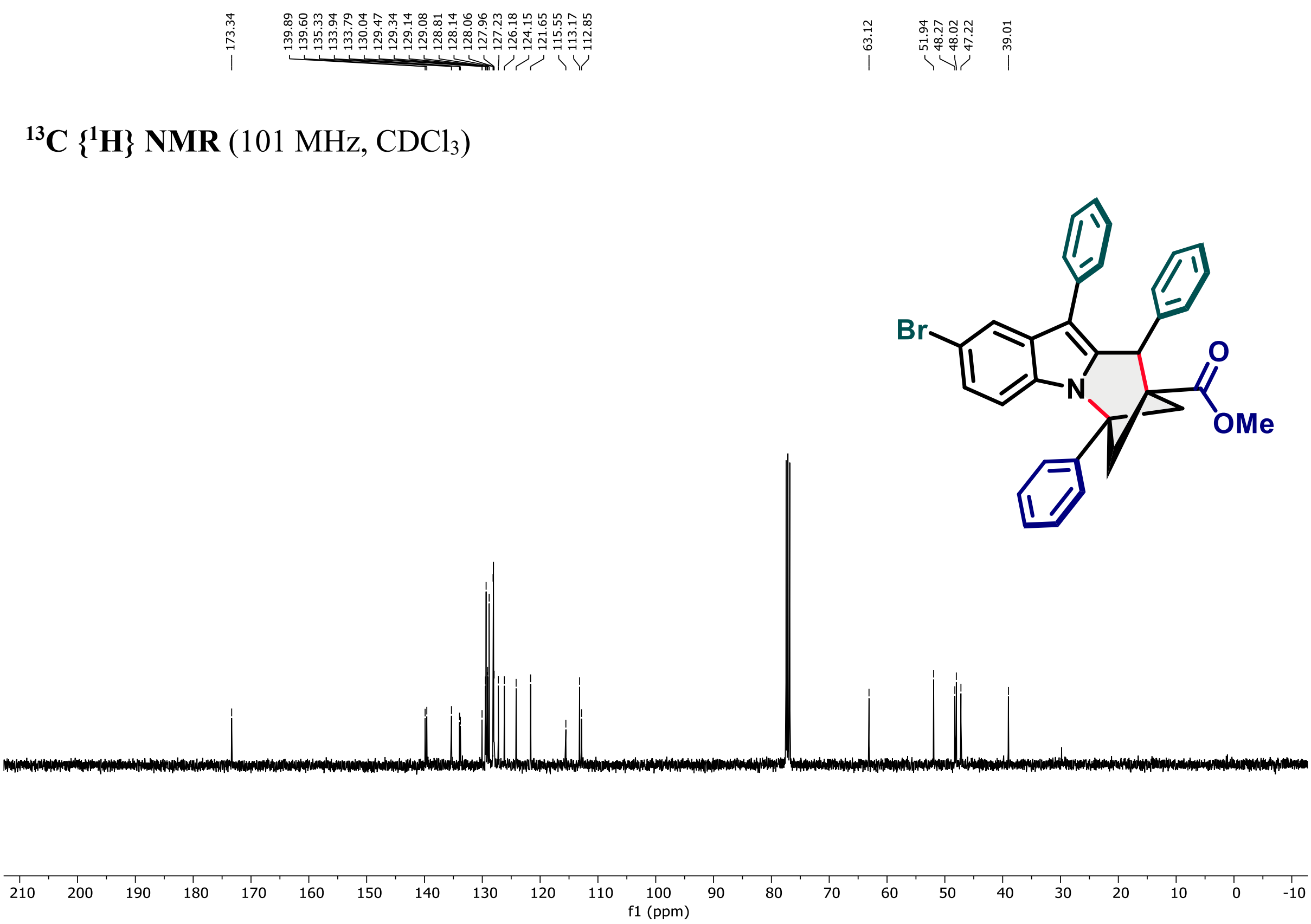

Ethyl-10-methyl-6,9-diphenyl-6,7-dihydro-6,8-methanopyrido[1,2-a]indole-8(9*H*)-carboxylate (3u):

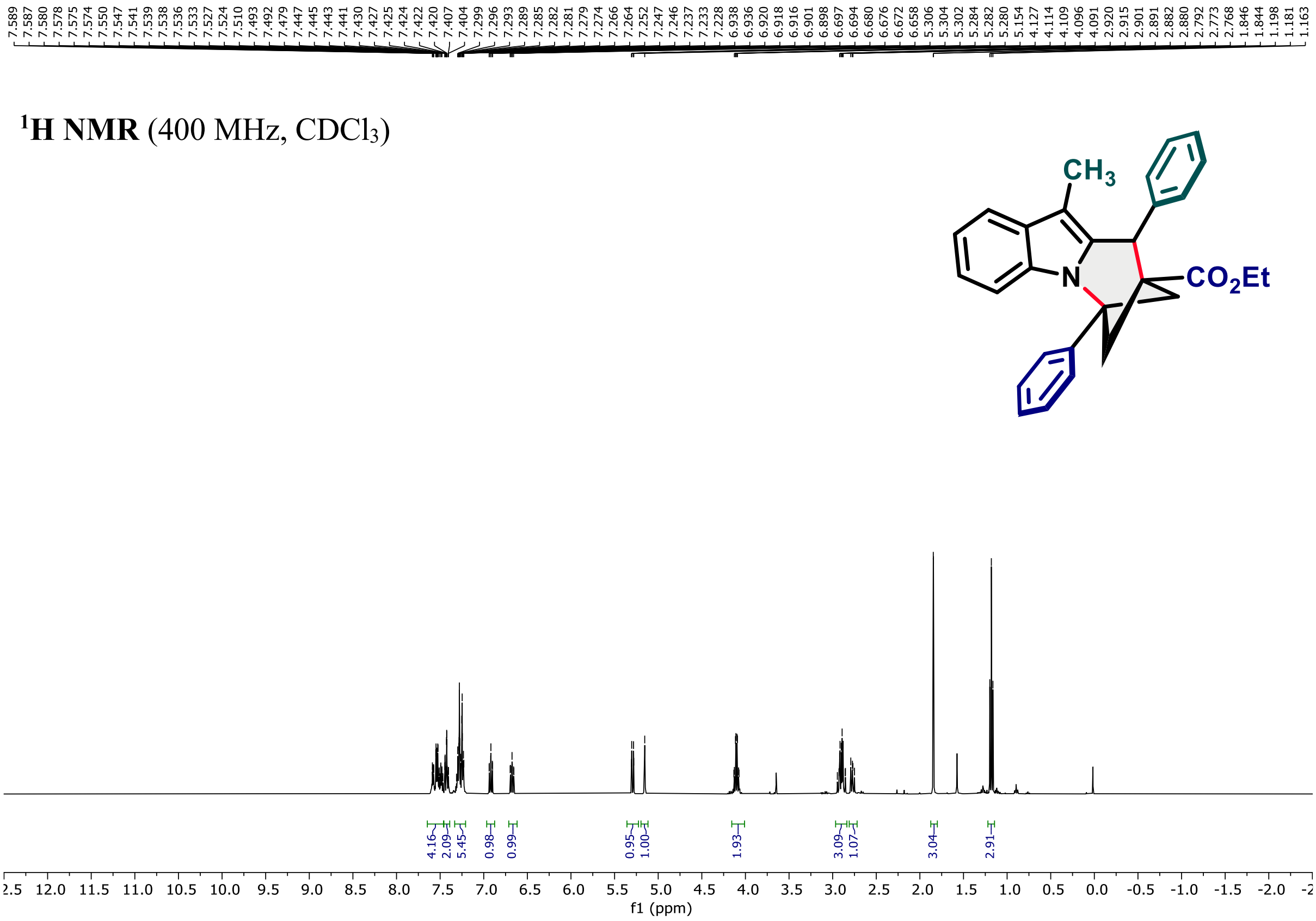

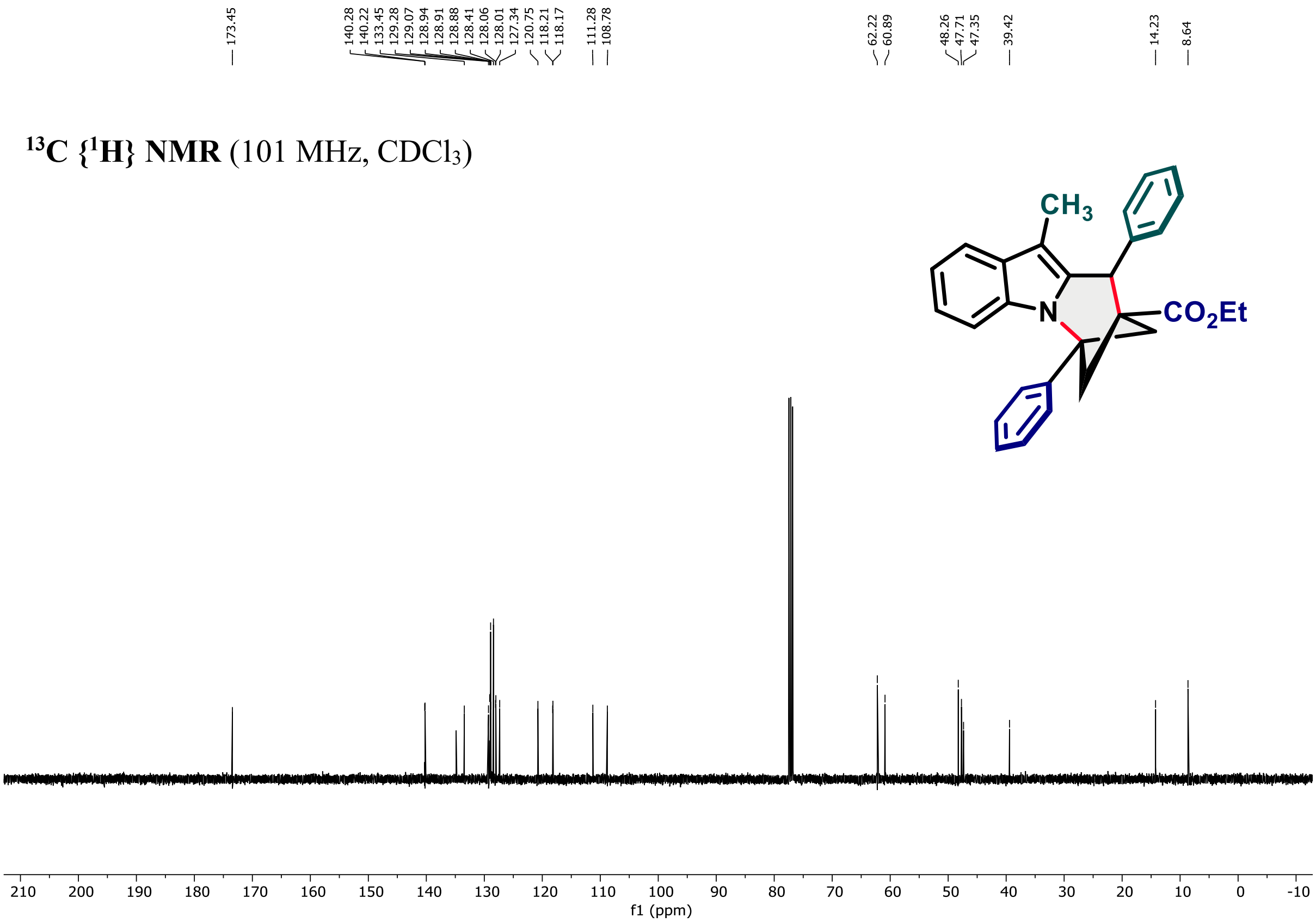

Isopropyl-10-methyl-6,9-diphenyl-6,7-dihydro-6,8-methanopyrido[1,2-a]indole-8(9*H*)-carboxylate (3v):

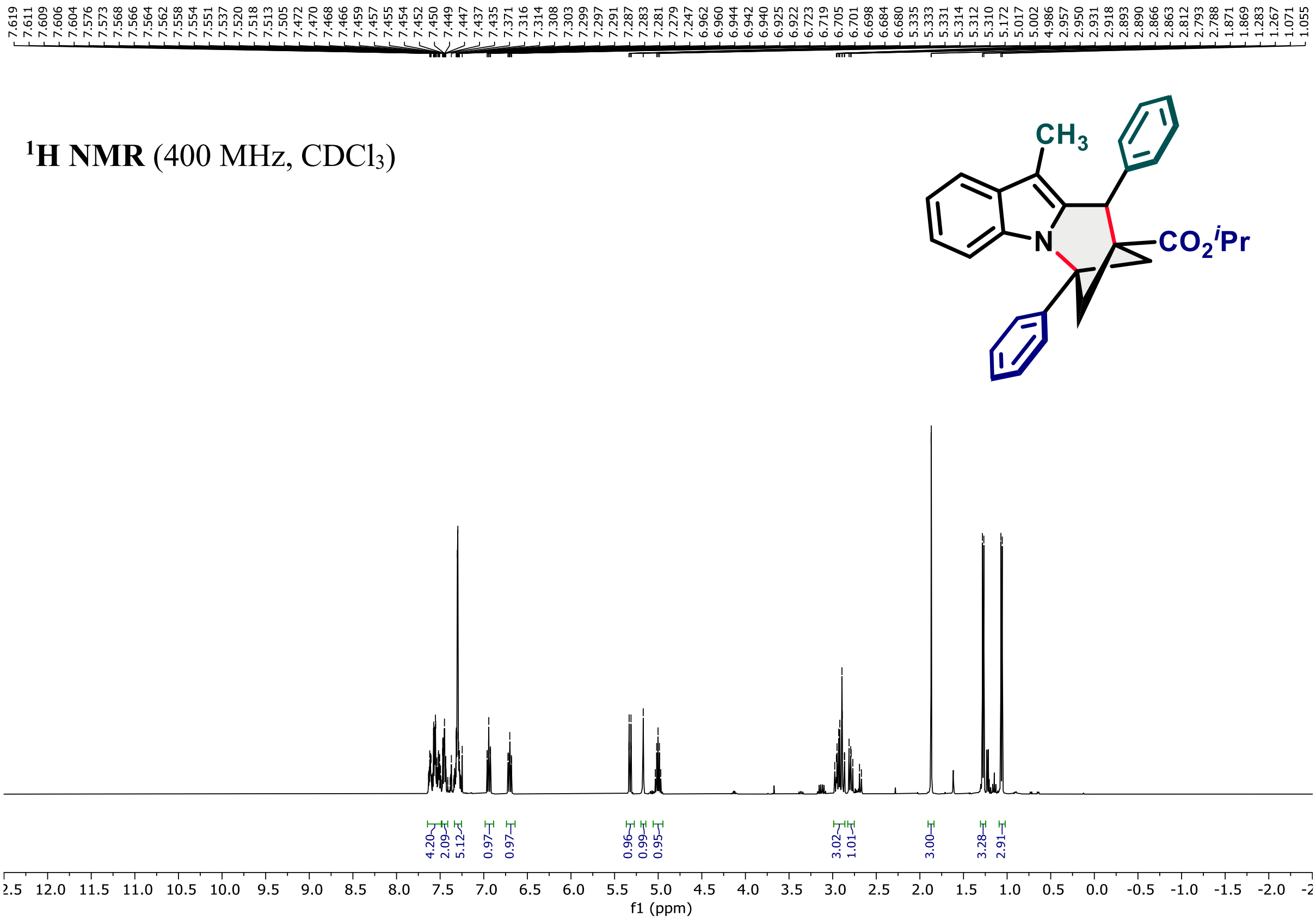

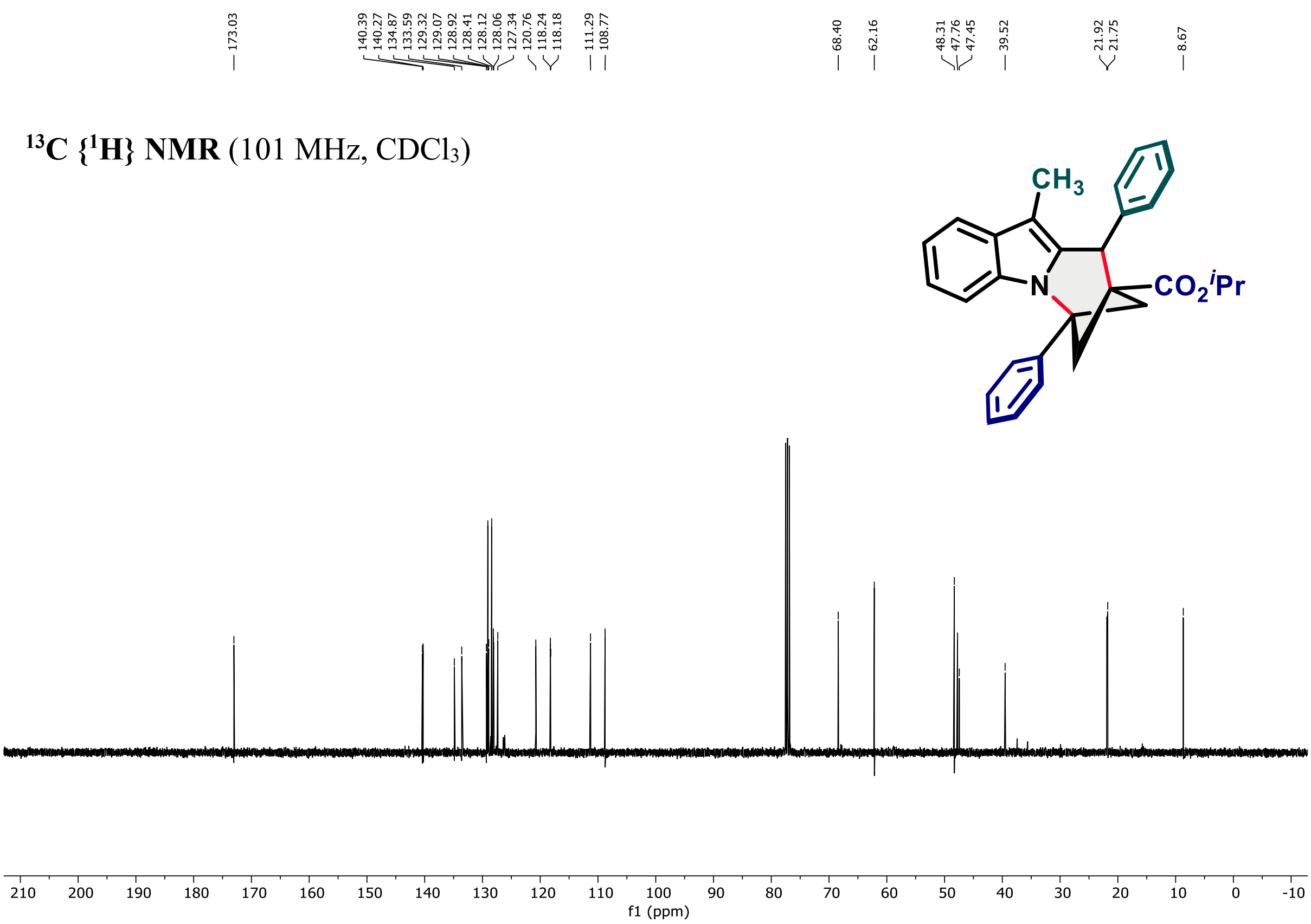

***tert*-butyl 10-methyl-6,9-diphenyl-6,7-dihydro-6,8-methanopyrido[1,2-a]indole-8(9*H*)-carboxylate (3w):**

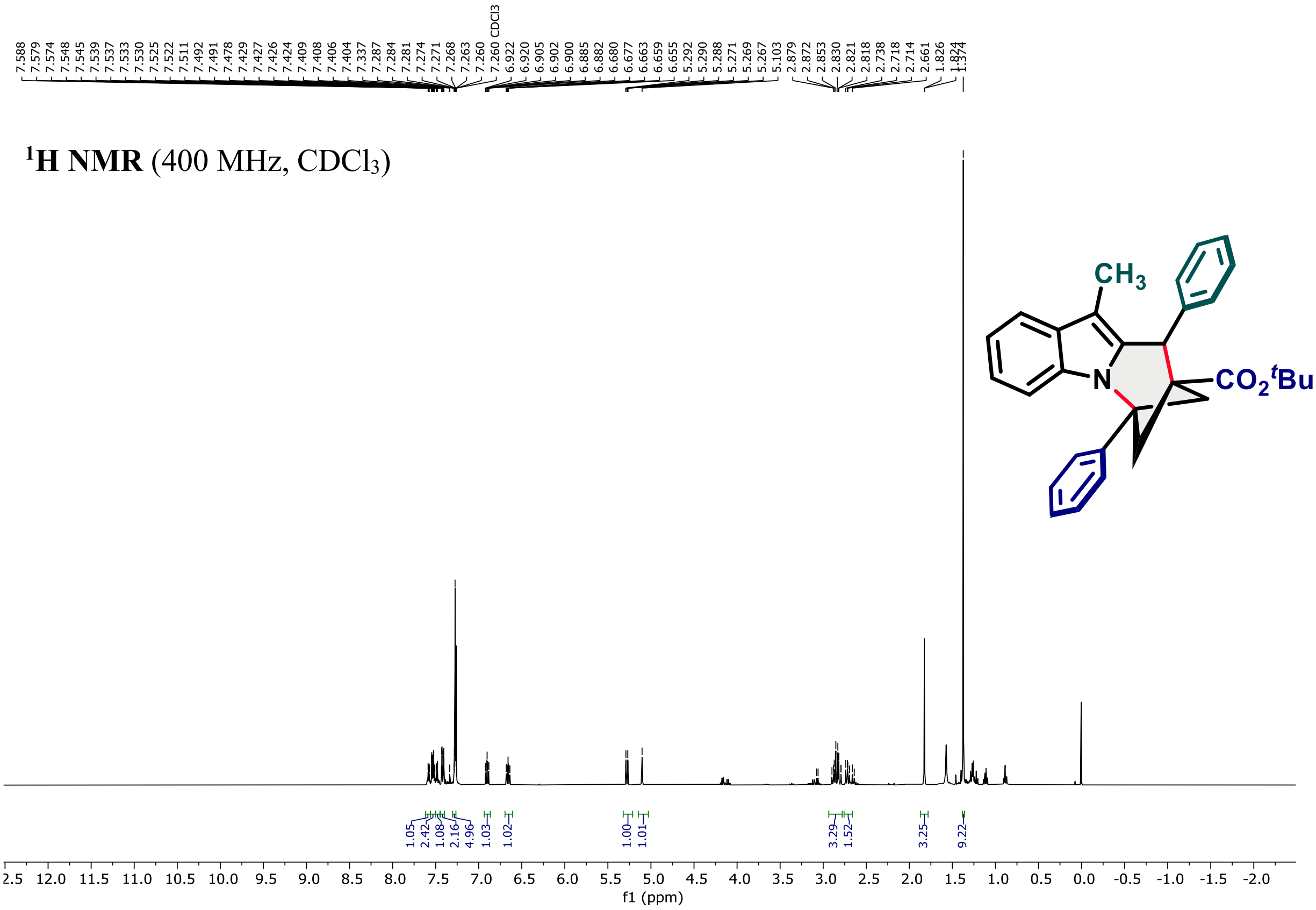

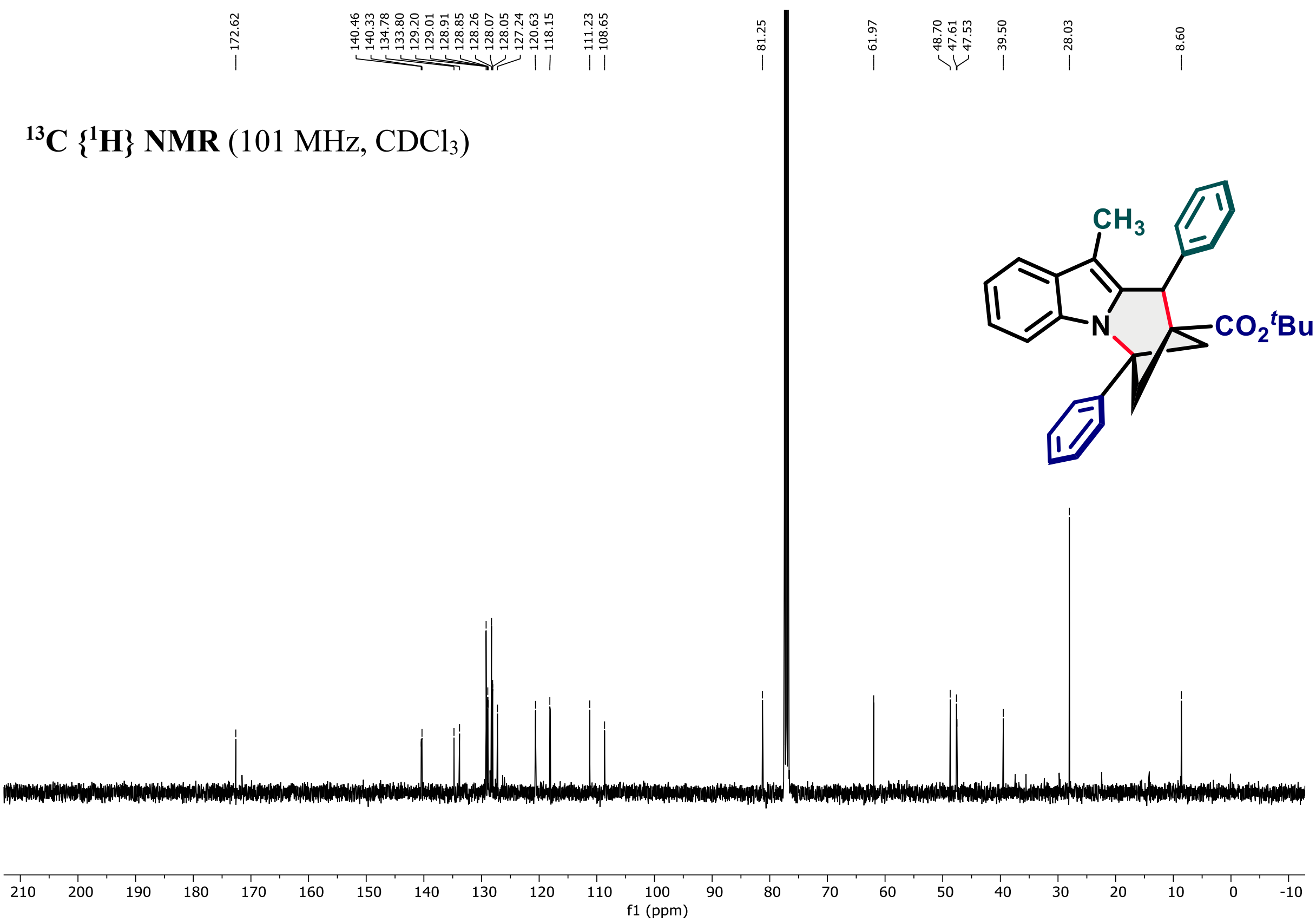

Phenyl-10-methyl-6,9-diphenyl-6,7-dihydro-6,8-methanopyrido[1,2-a]indole-8(9*H*)-carboxylate (3x):

^{1}H NMR (400 MHz, $CDCl_3$)

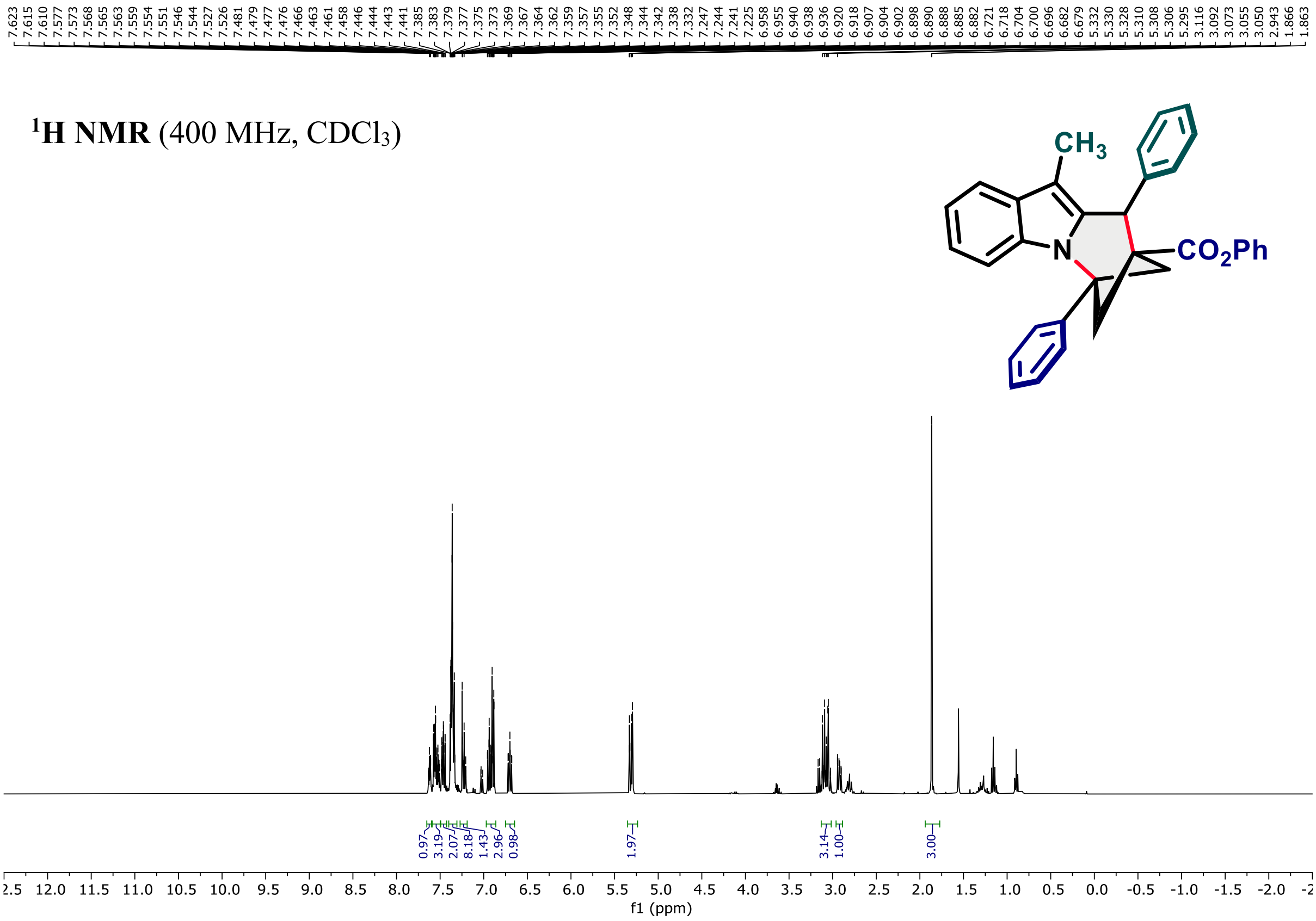

^{13}C {^{1}H} NMR (101 MHz, $CDCl_3$)

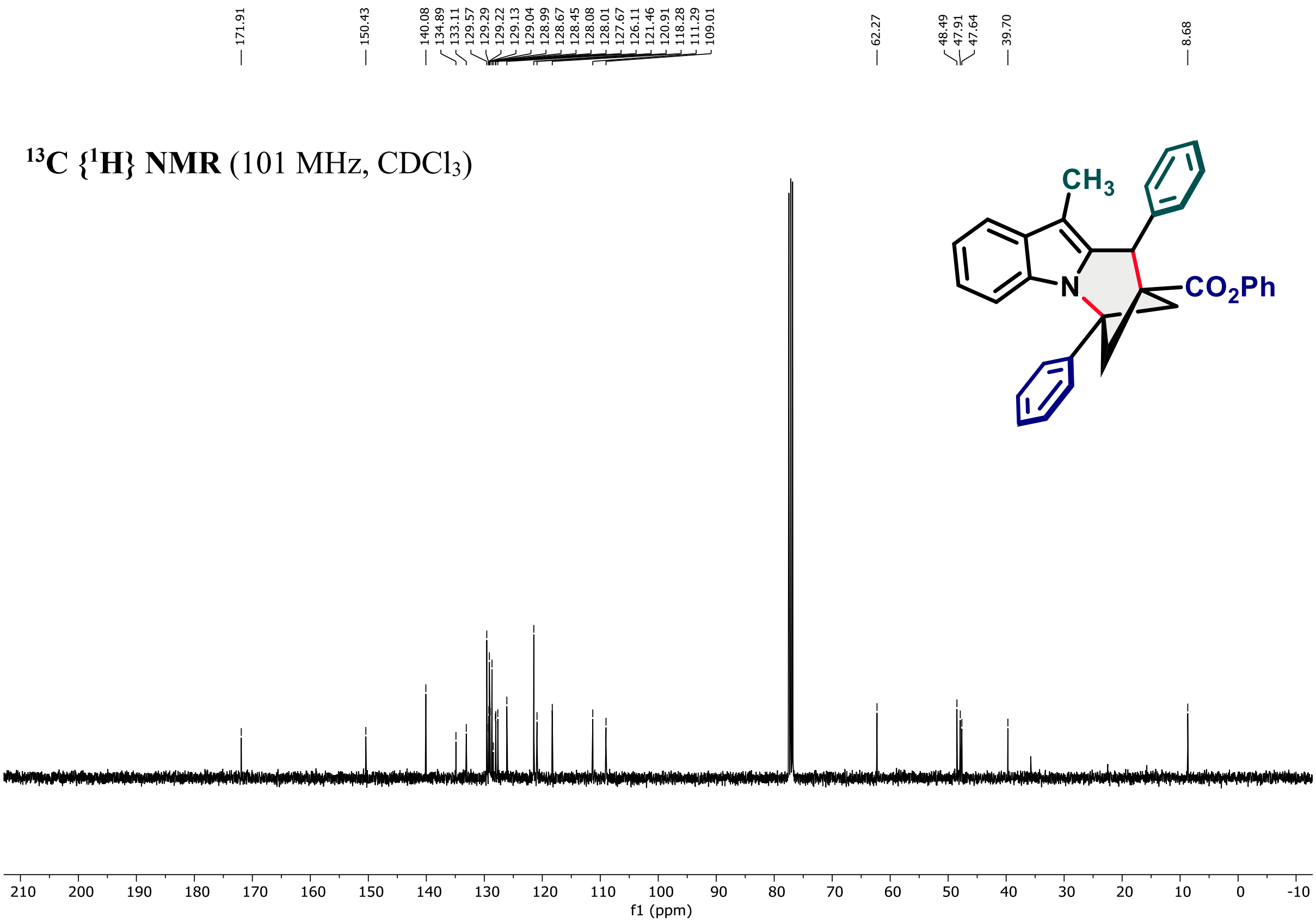

Benzyl-10-methyl-6,9-diphenyl-6,7-dihydro-6,8-methanopyrido[1,2-a]indole-8(9*H*)-carboxylate (3y):

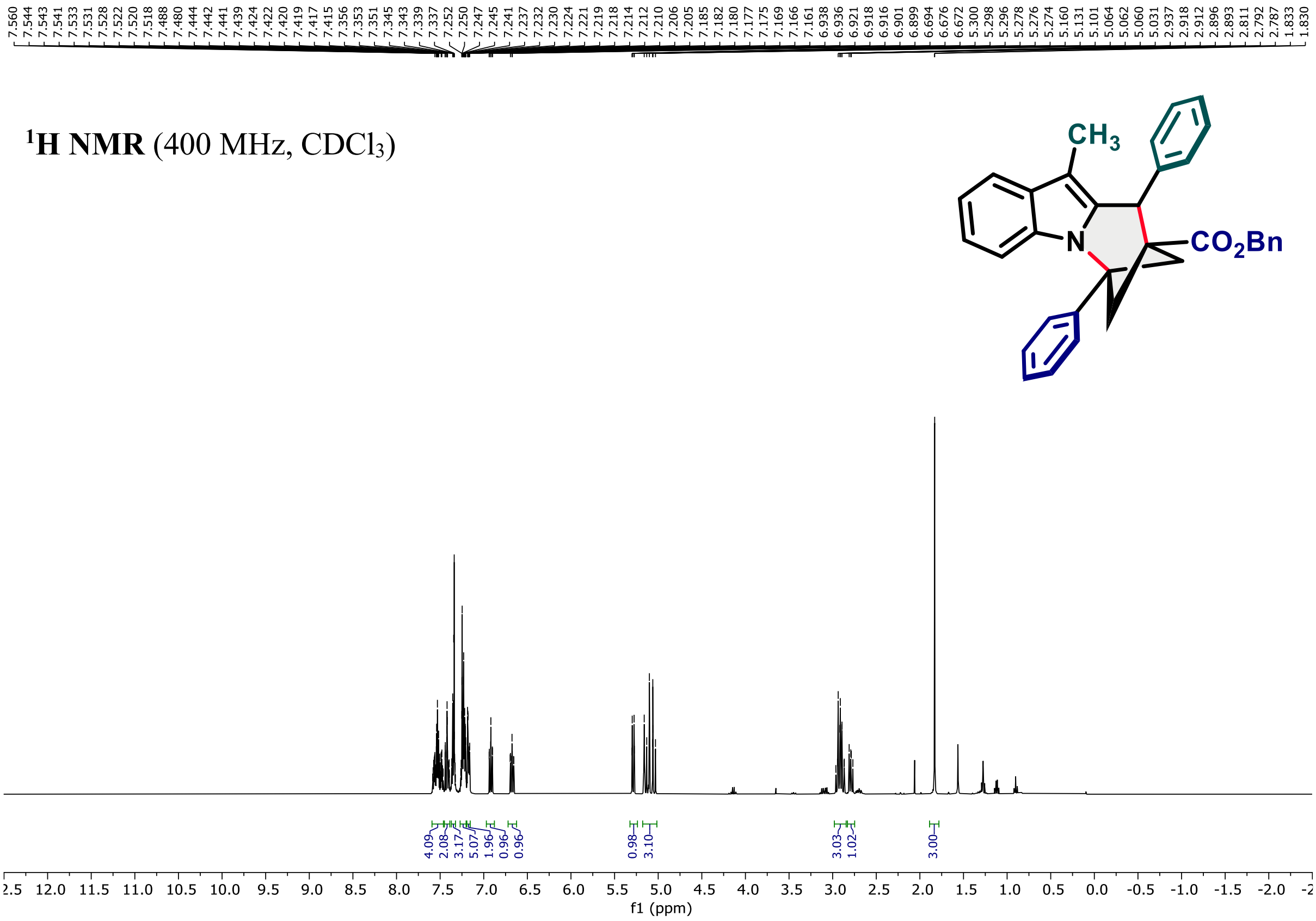

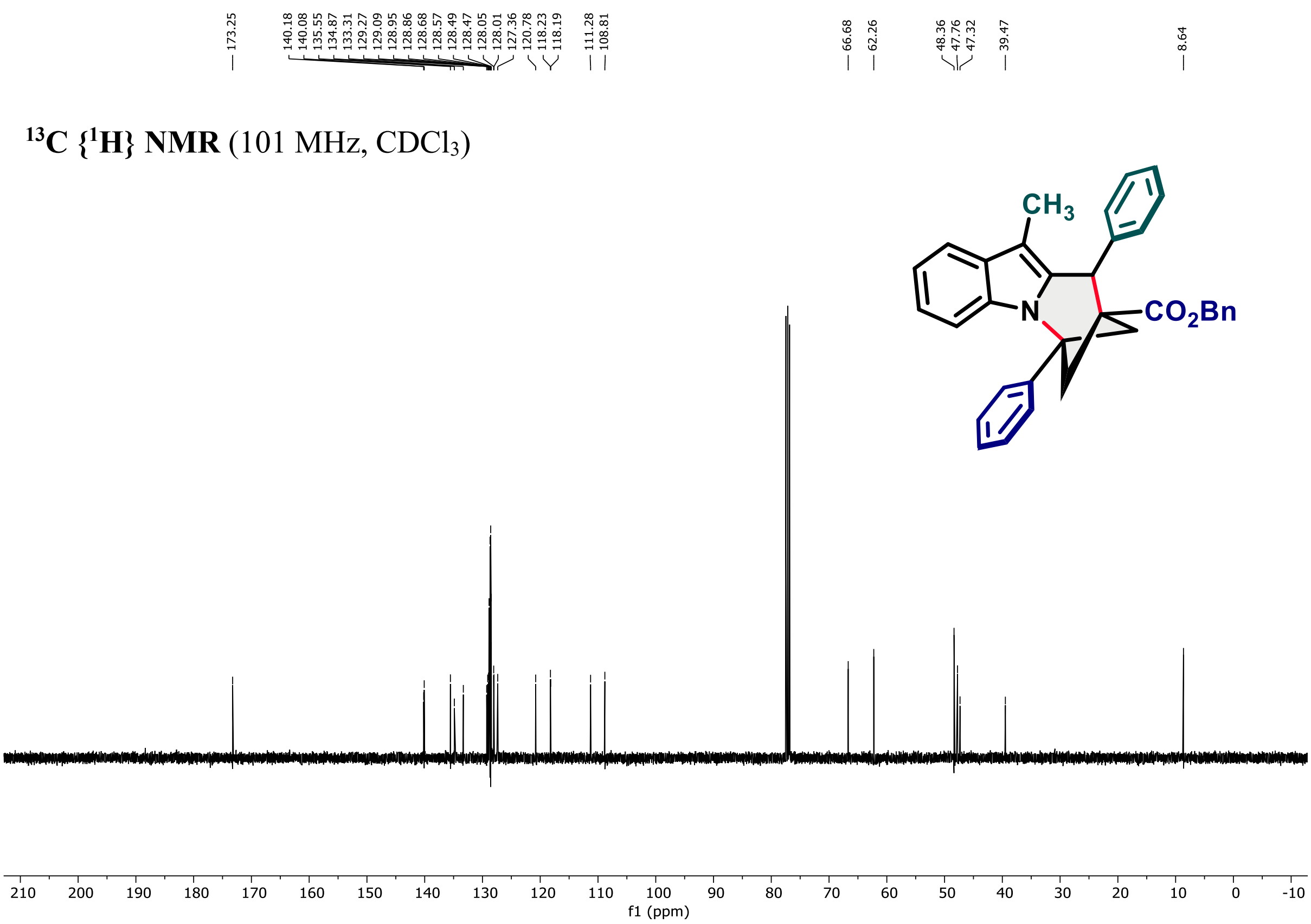

4-Methoxyphenyl 10-methyl-6,9-diphenyl-6,7-dihydro-6,8-methanopyrido[1,2-a] indole-8(9*H*)-carboxylate (3z):

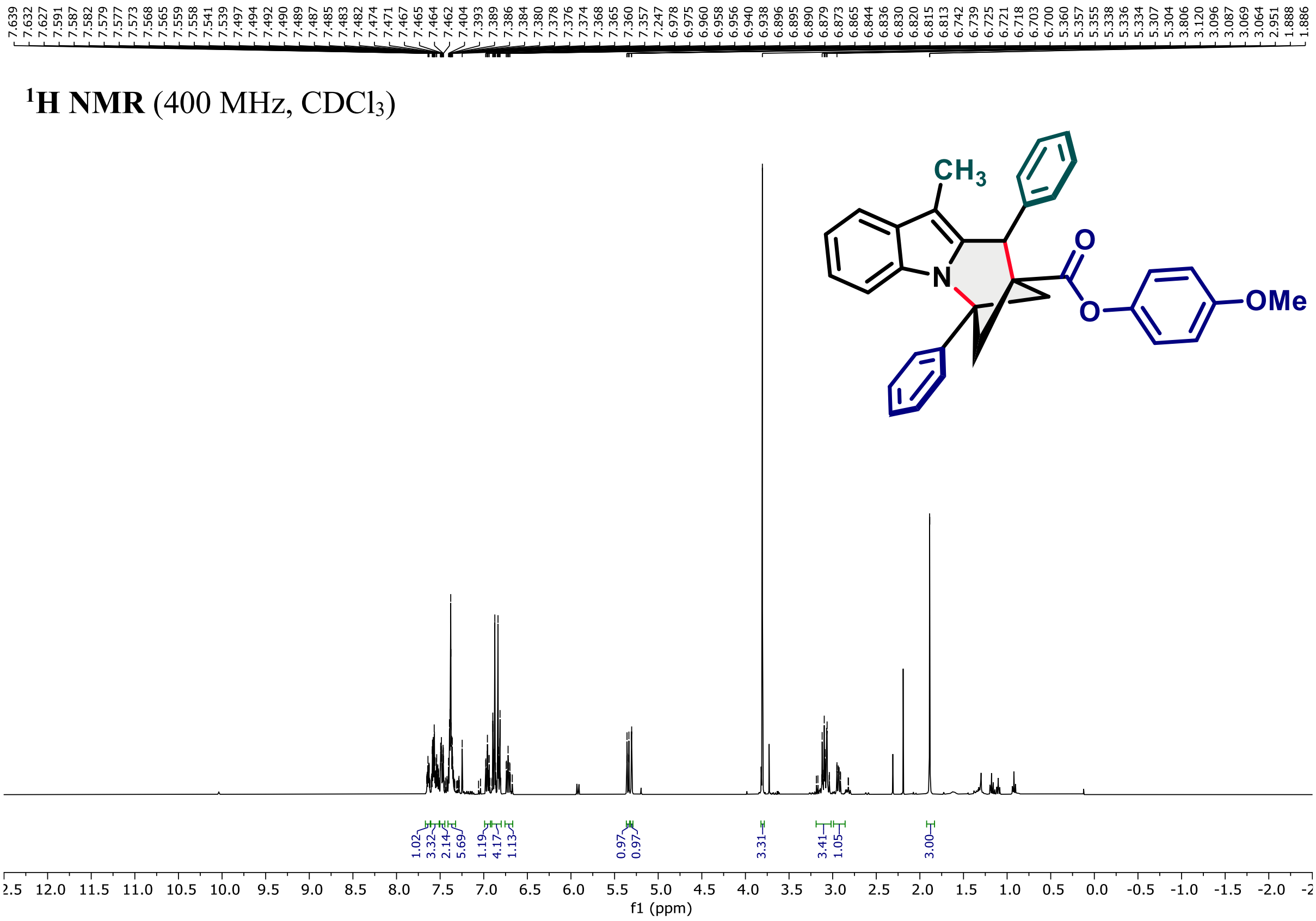

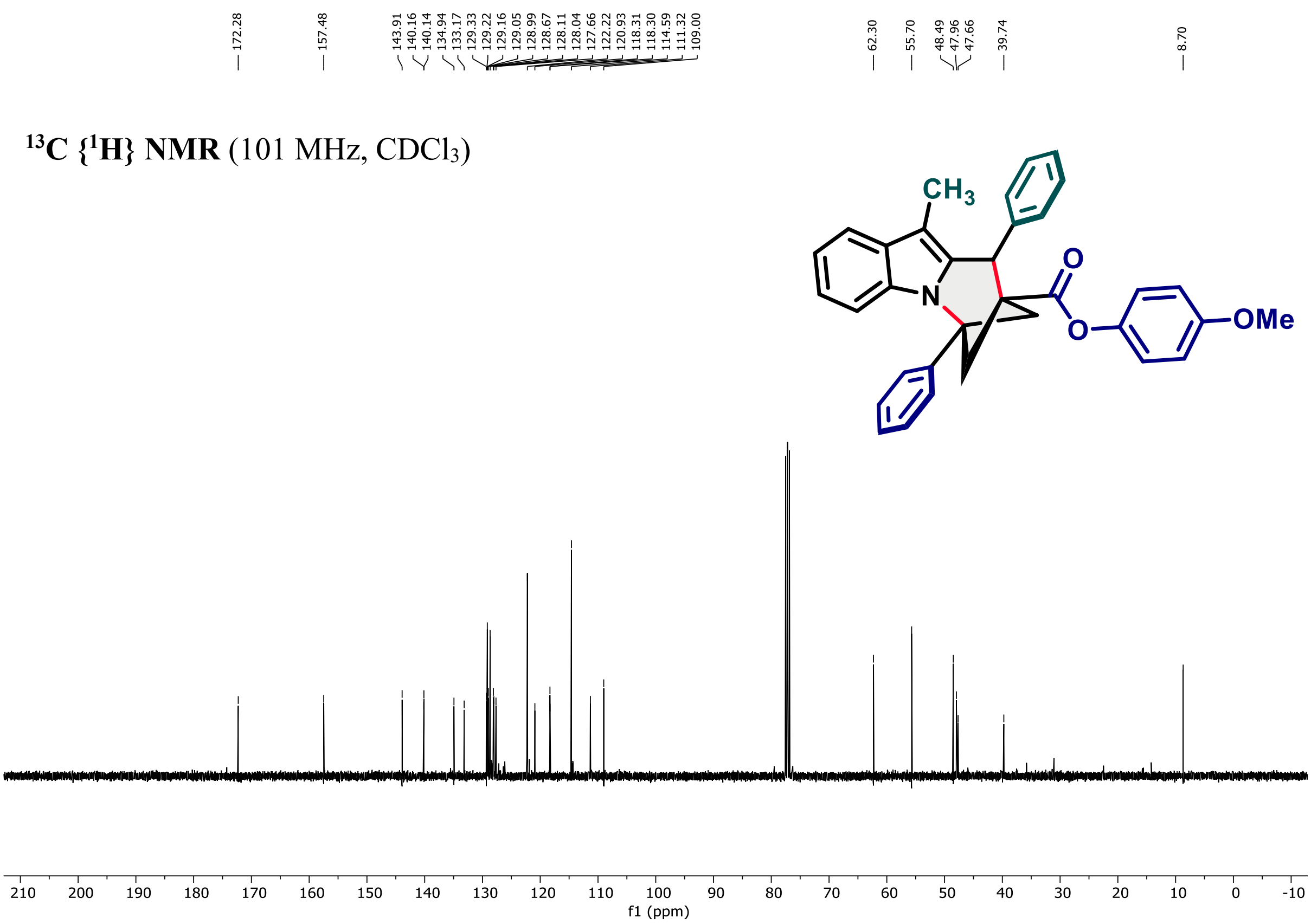

N-Methoxy-N,10-dimethyl-6,9-diphenyl-6,7-dihydro-6,8-methanopyrido[1,2-a]indole-8(9H)-carboxamide (3aa):

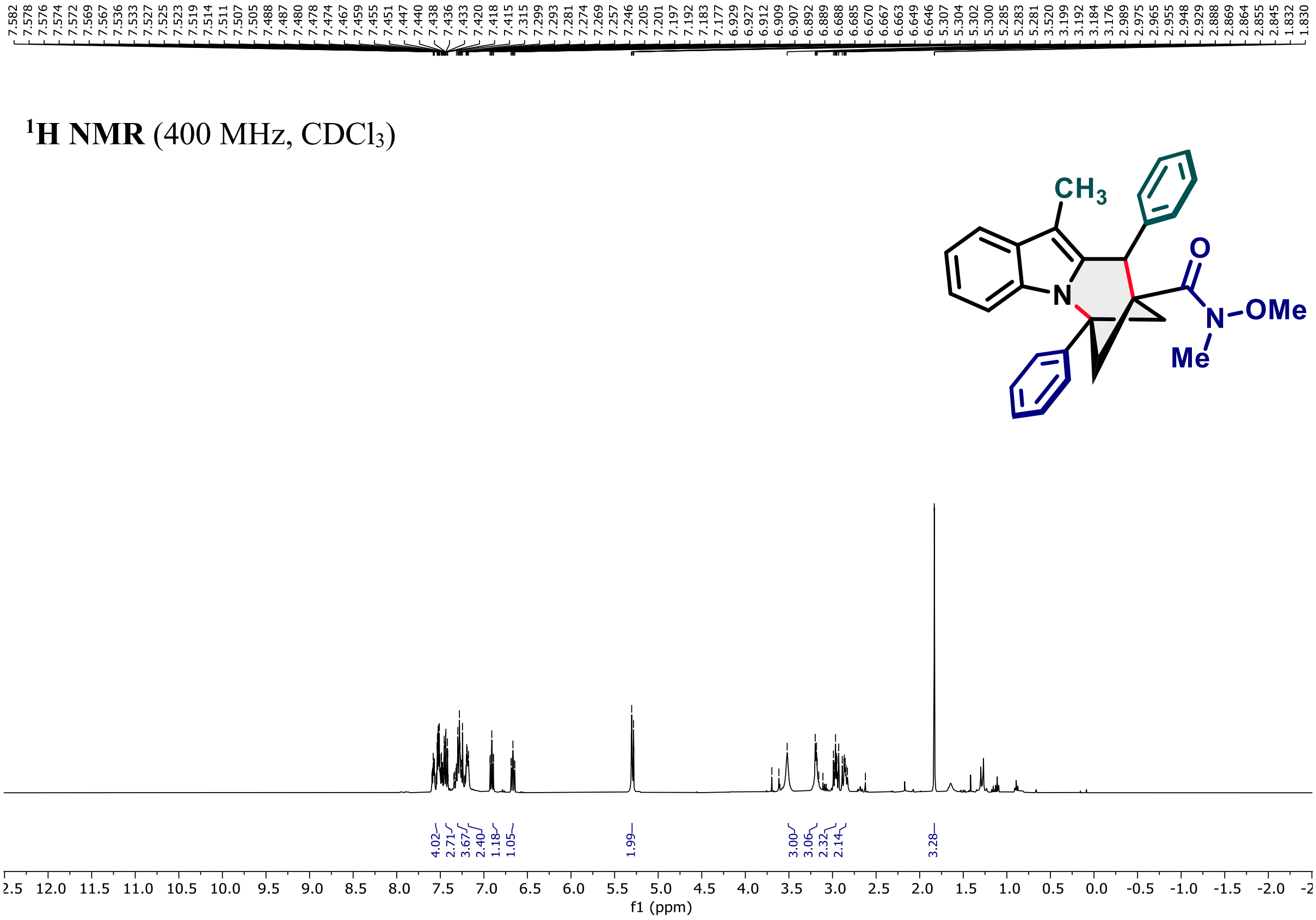

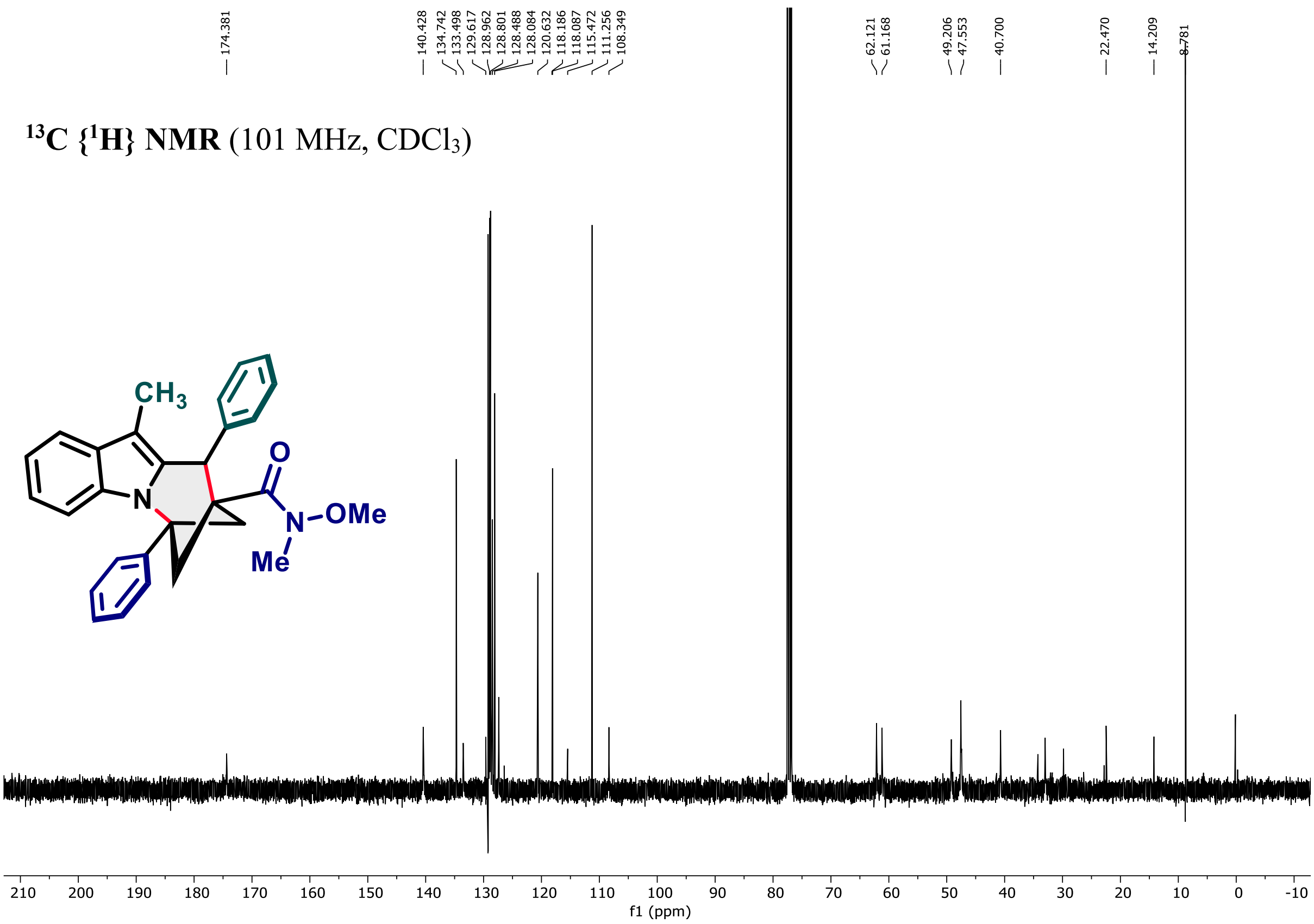

Methyl 10-methyl-6-(naphthalen-2-yl)-9-phenyl-6,7-dihydro-6,8-methanopyrido [1,2-a]indole-8(9*H*)-carboxylate (3ab):

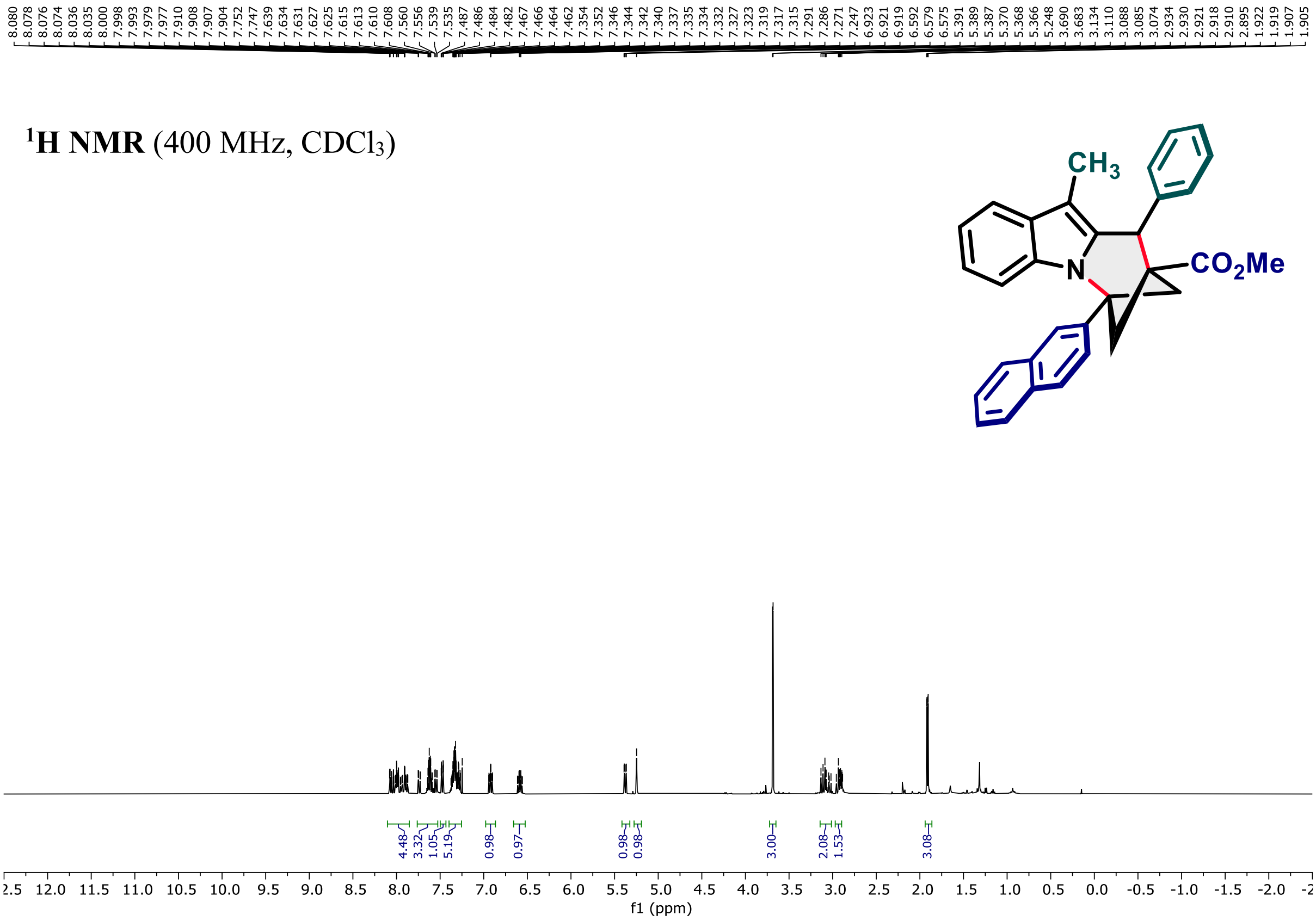

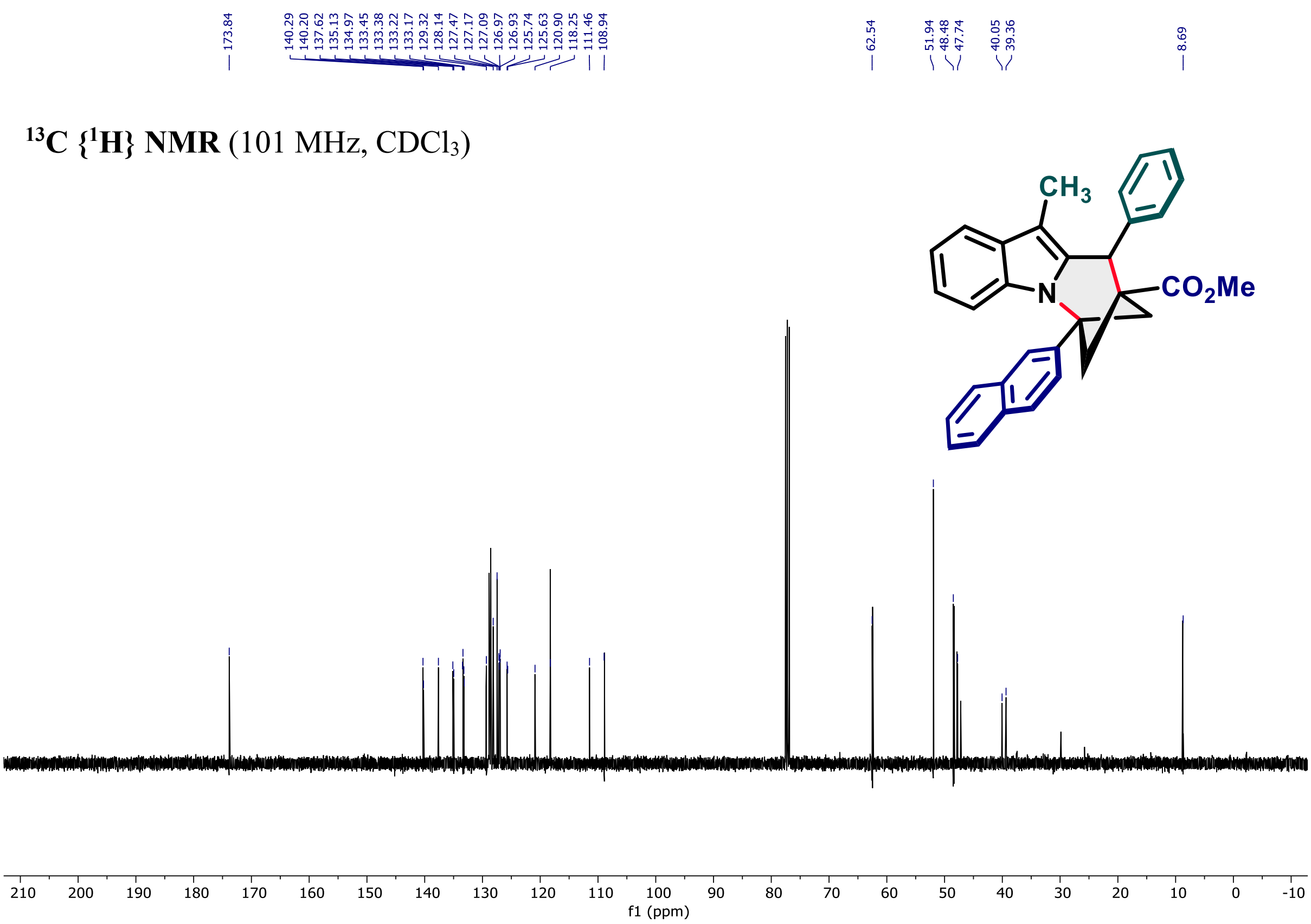

Methyl-6-(4-(*tert*-butyl)phenyl)-10-methyl-9-phenyl-6,7-dihydro-6,8-methanopyrido [1,2-a]indole-8(9*H*)-carboxylate (3ac):

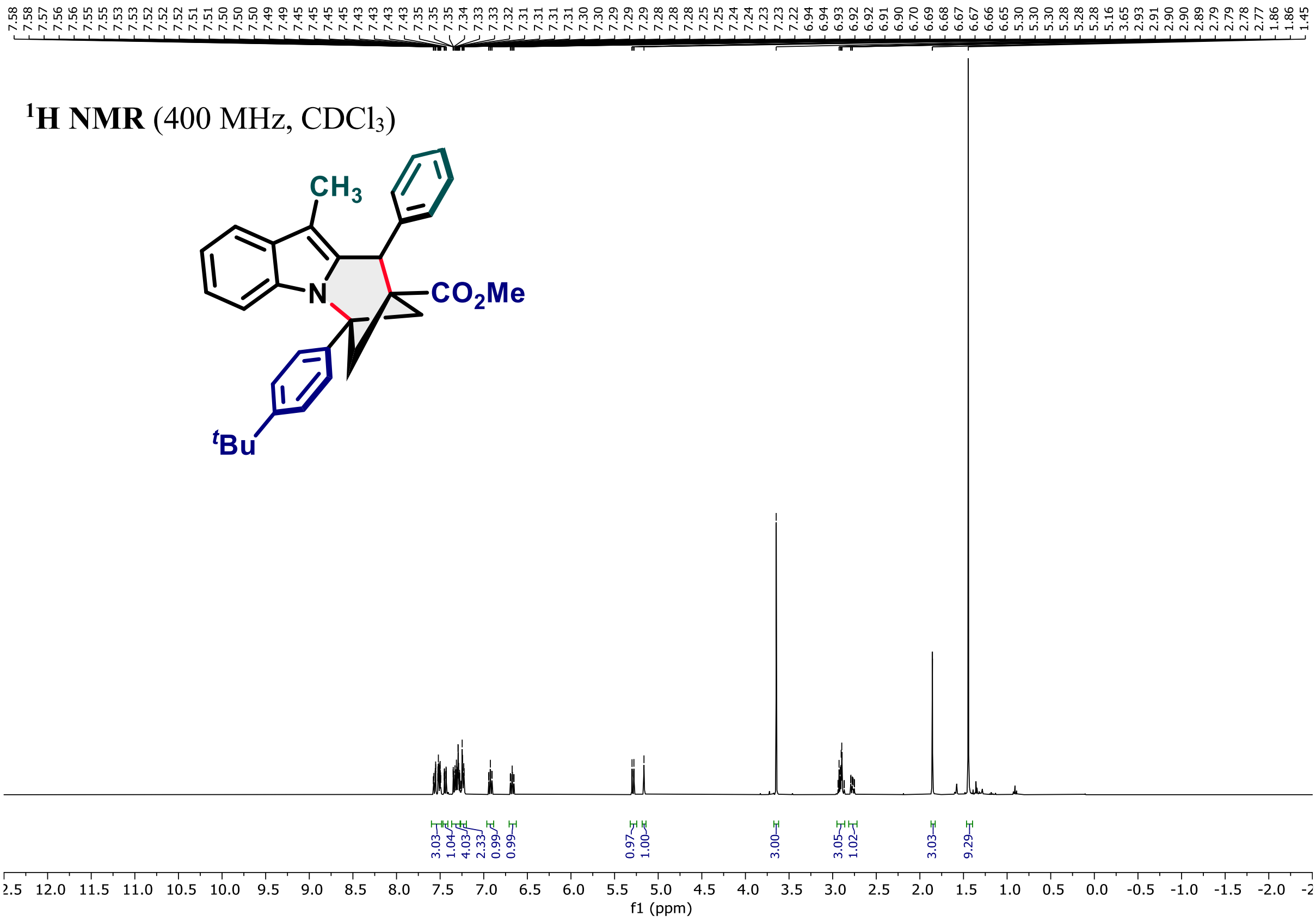

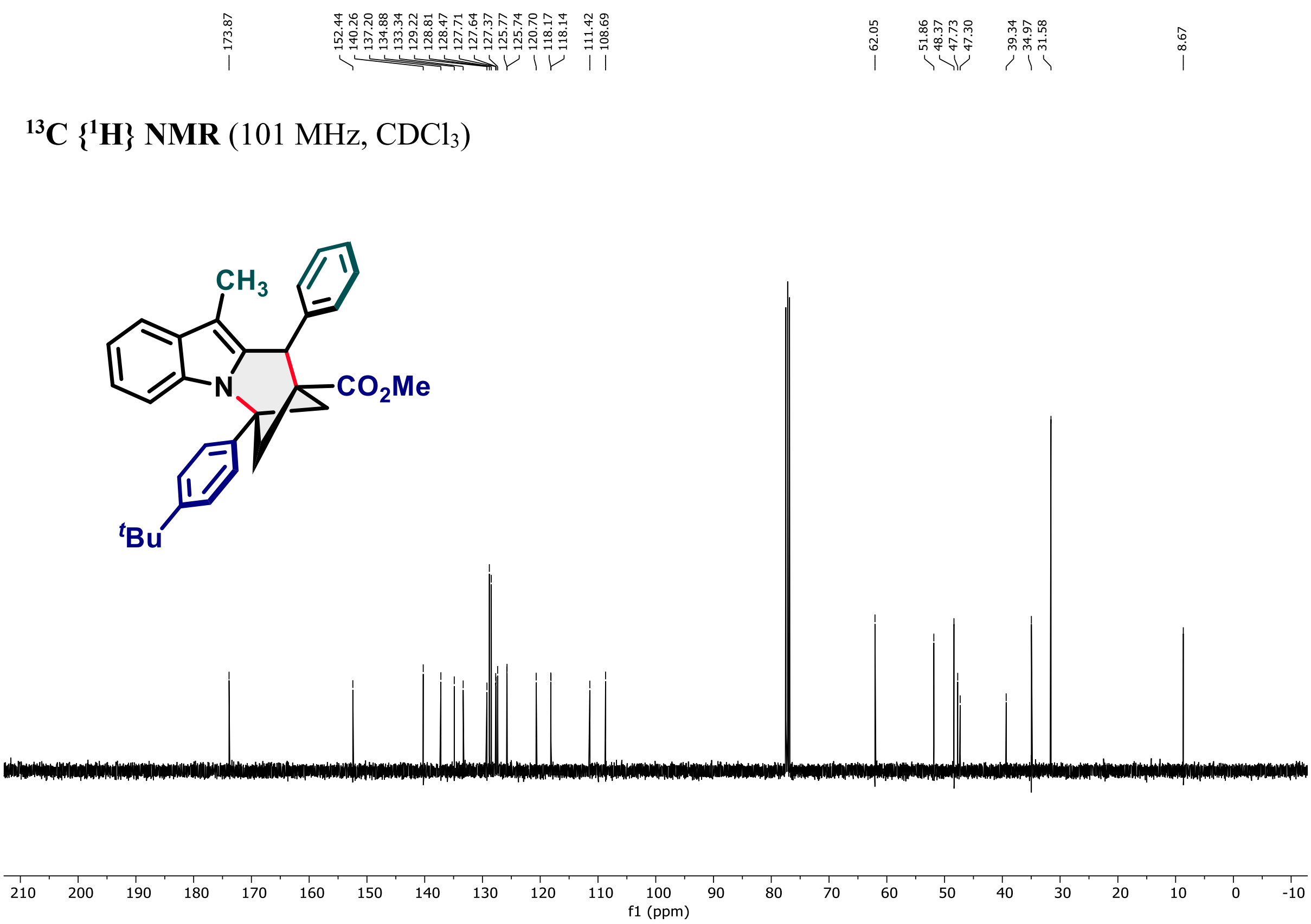

Methyl 6-([1,1'-biphenyl]-4-yl)-10-methyl-9-phenyl-6,7-dihydro-6,8-methanopyrido [1,2-a]indole-8(9*H*)-carboxylate (3ad):

^{1}H NMR (400 MHz, $CDCl_3$)

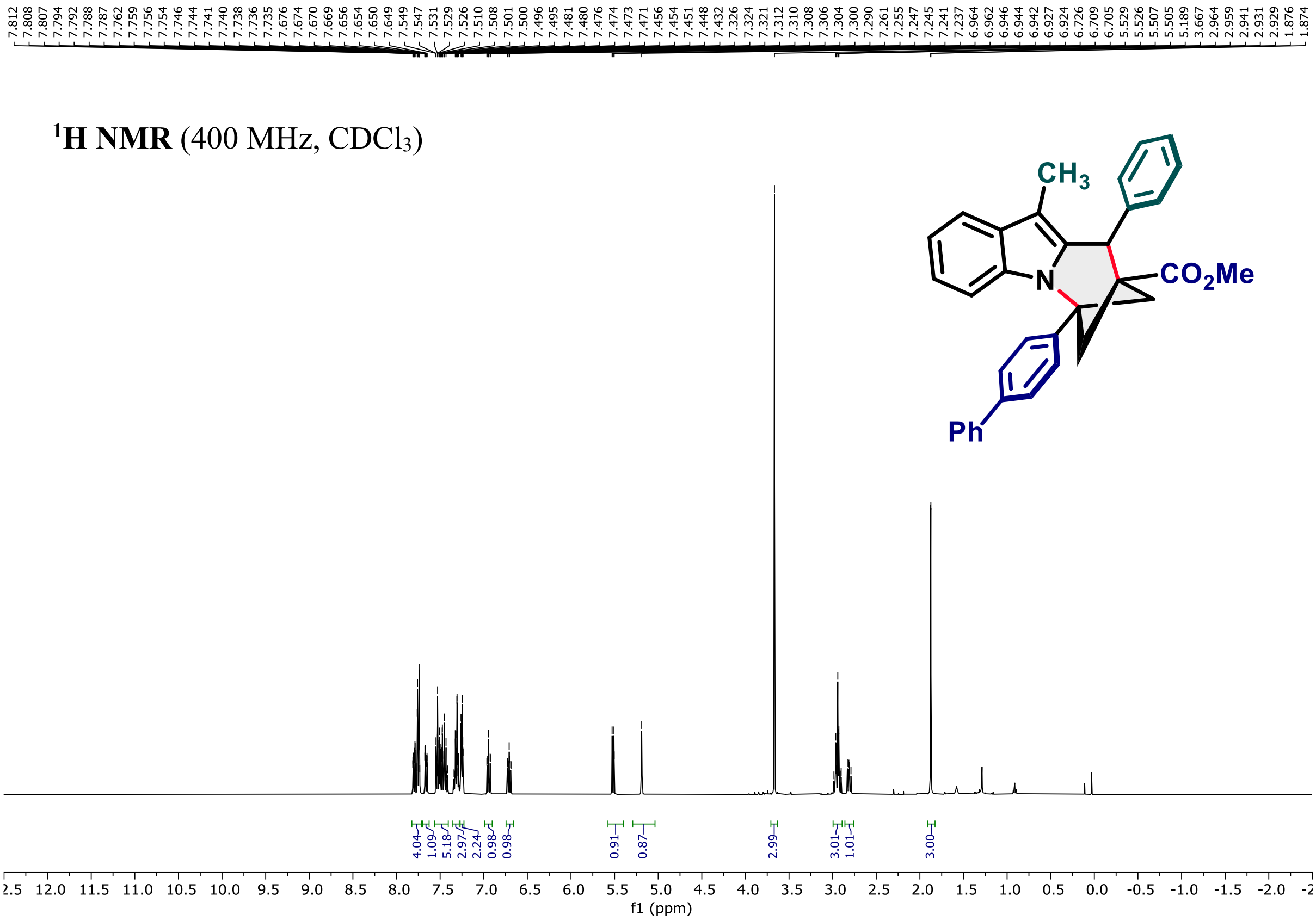

^{13}C {^{1}H} NMR (101 MHz, $CDCl_3$)

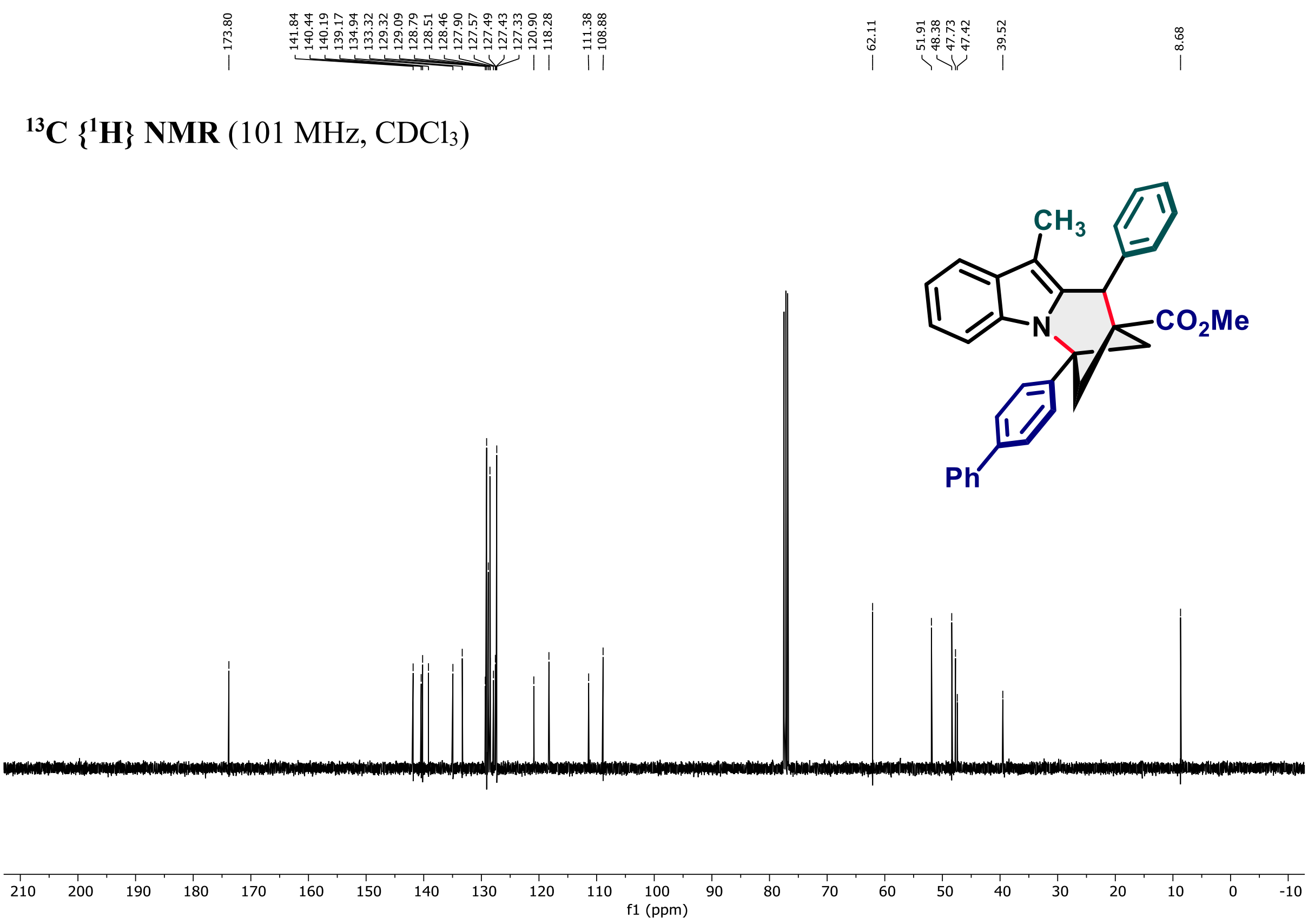

Methyl-6-(3-methoxyphenyl)-10-methyl-9-phenyl-6,7-dihydro-6,8-methanopyrido[1,2-a]indole-8(9*H*)-carboxylate (3ae):

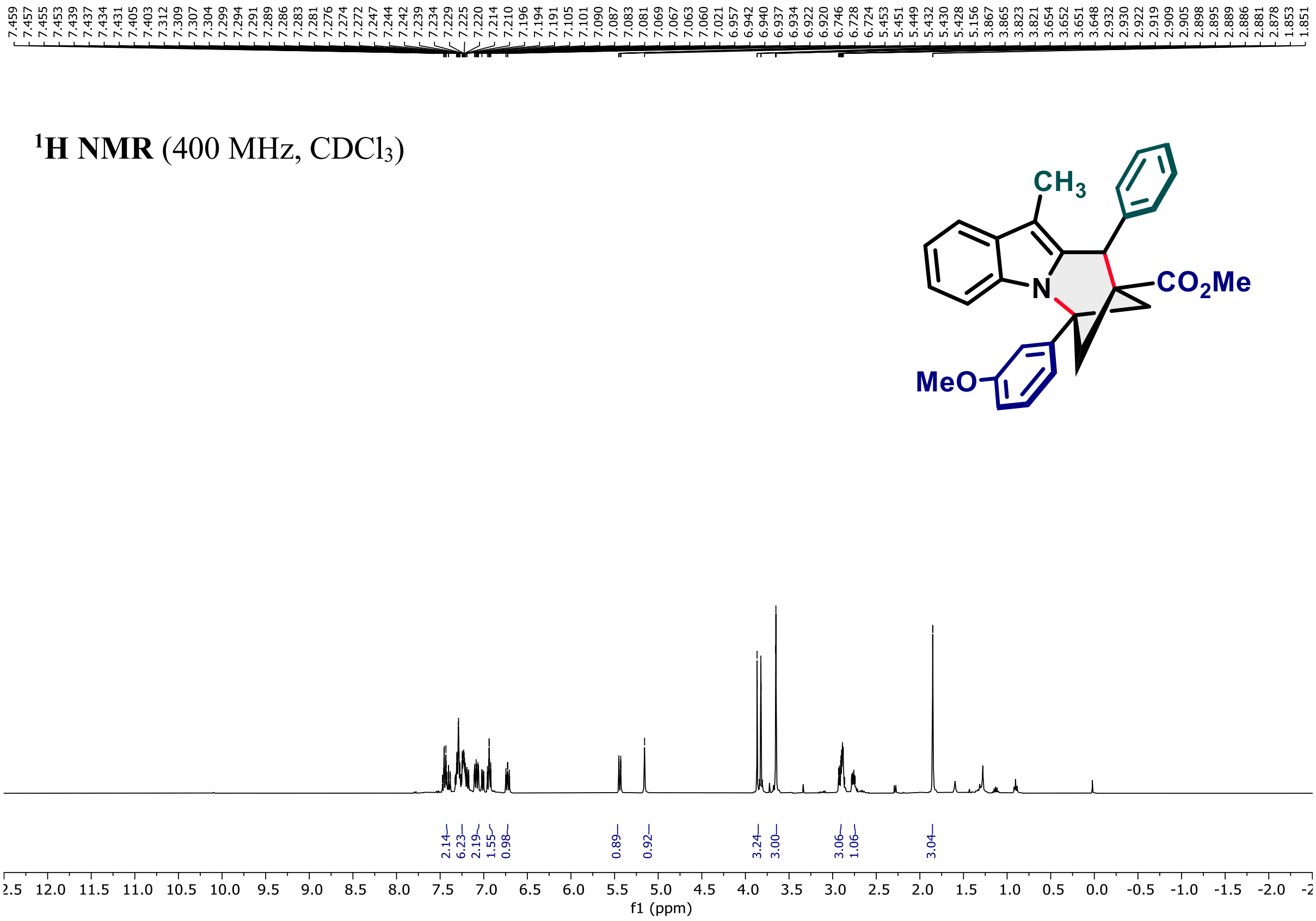

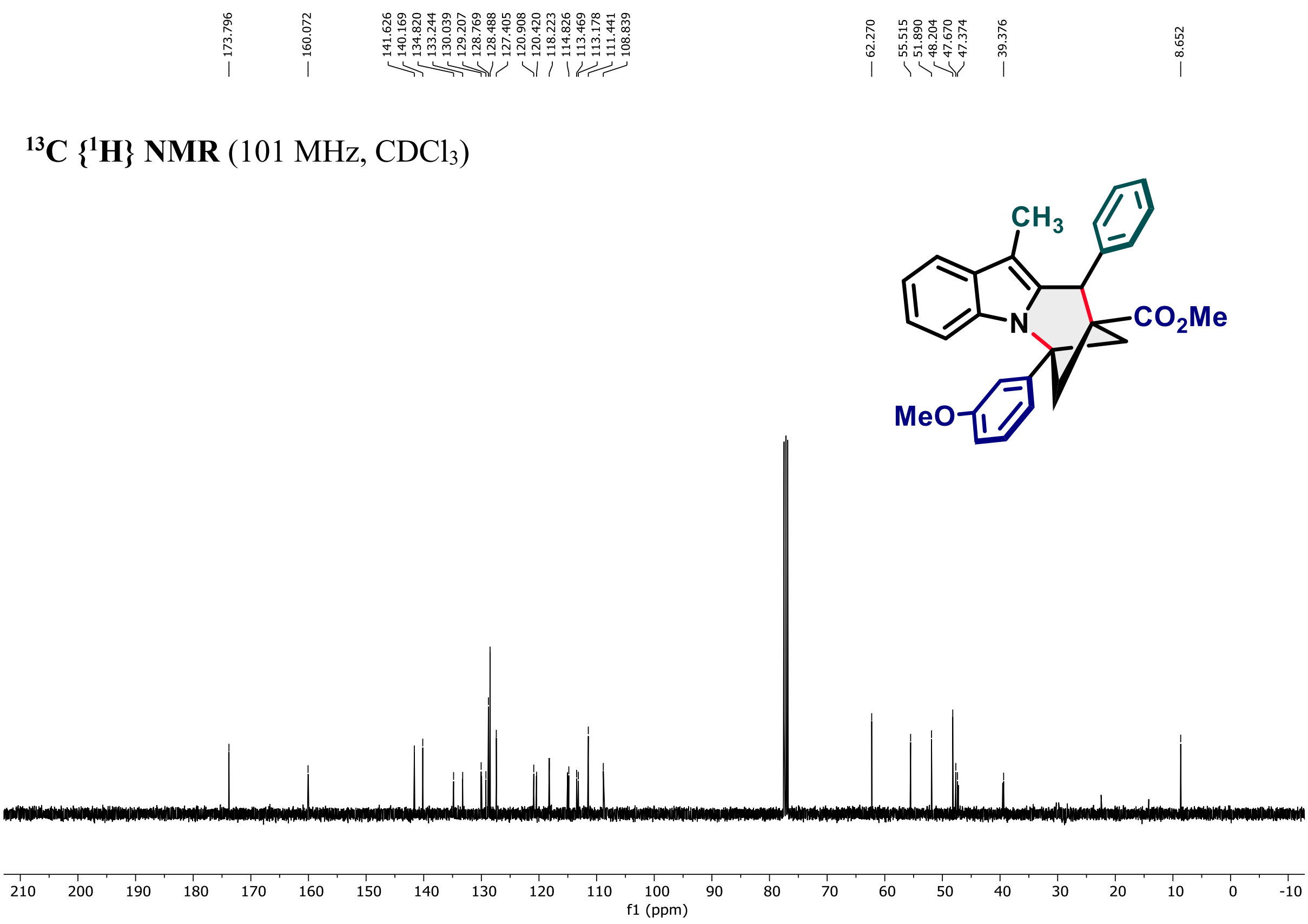

Methyl-6-(4-fluorophenyl)-10-methyl-9-phenyl-6,7-dihydro-6,8-methanopyrido[1,2-a]indole-8(9*H*)-carboxylate (3af):

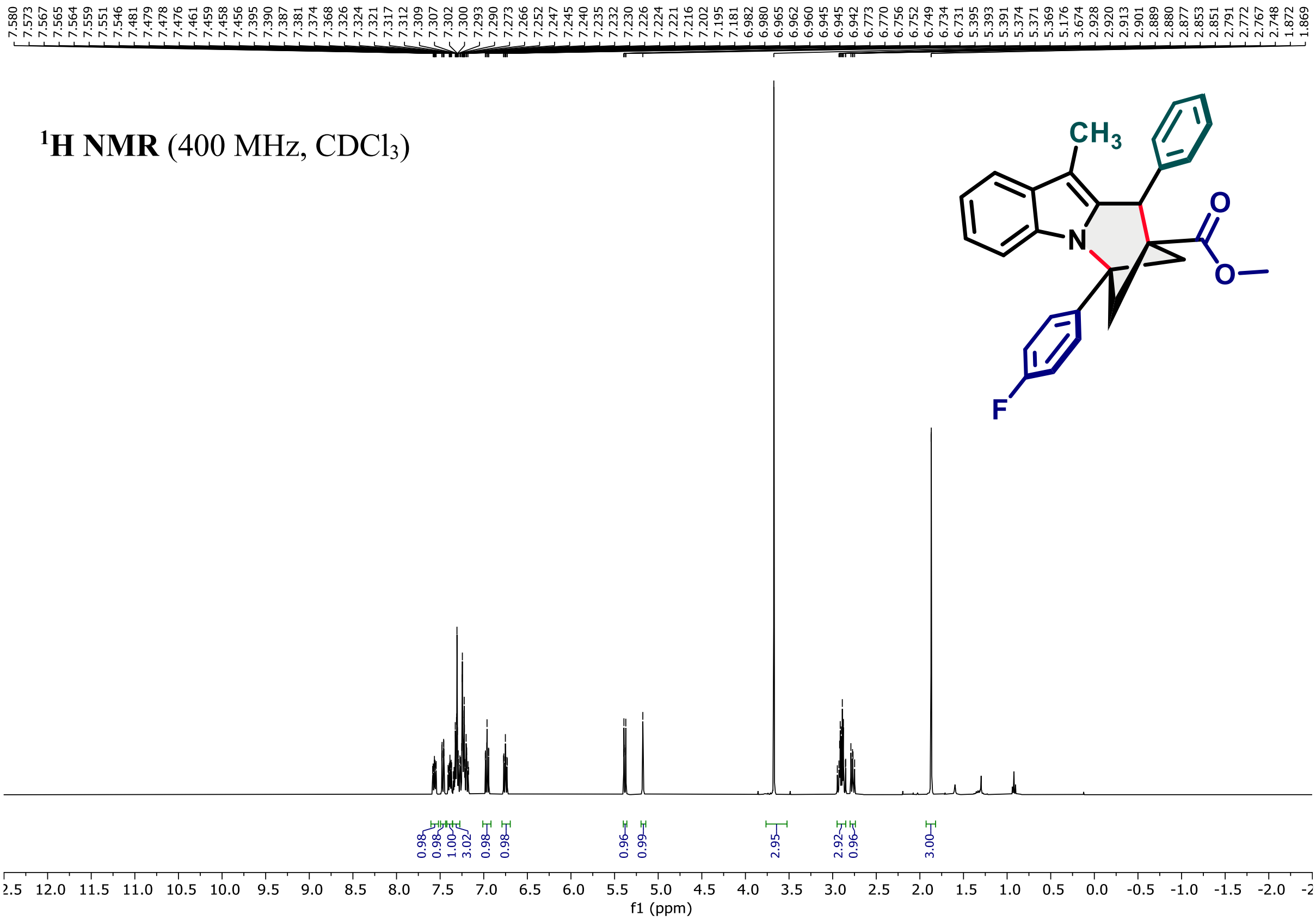

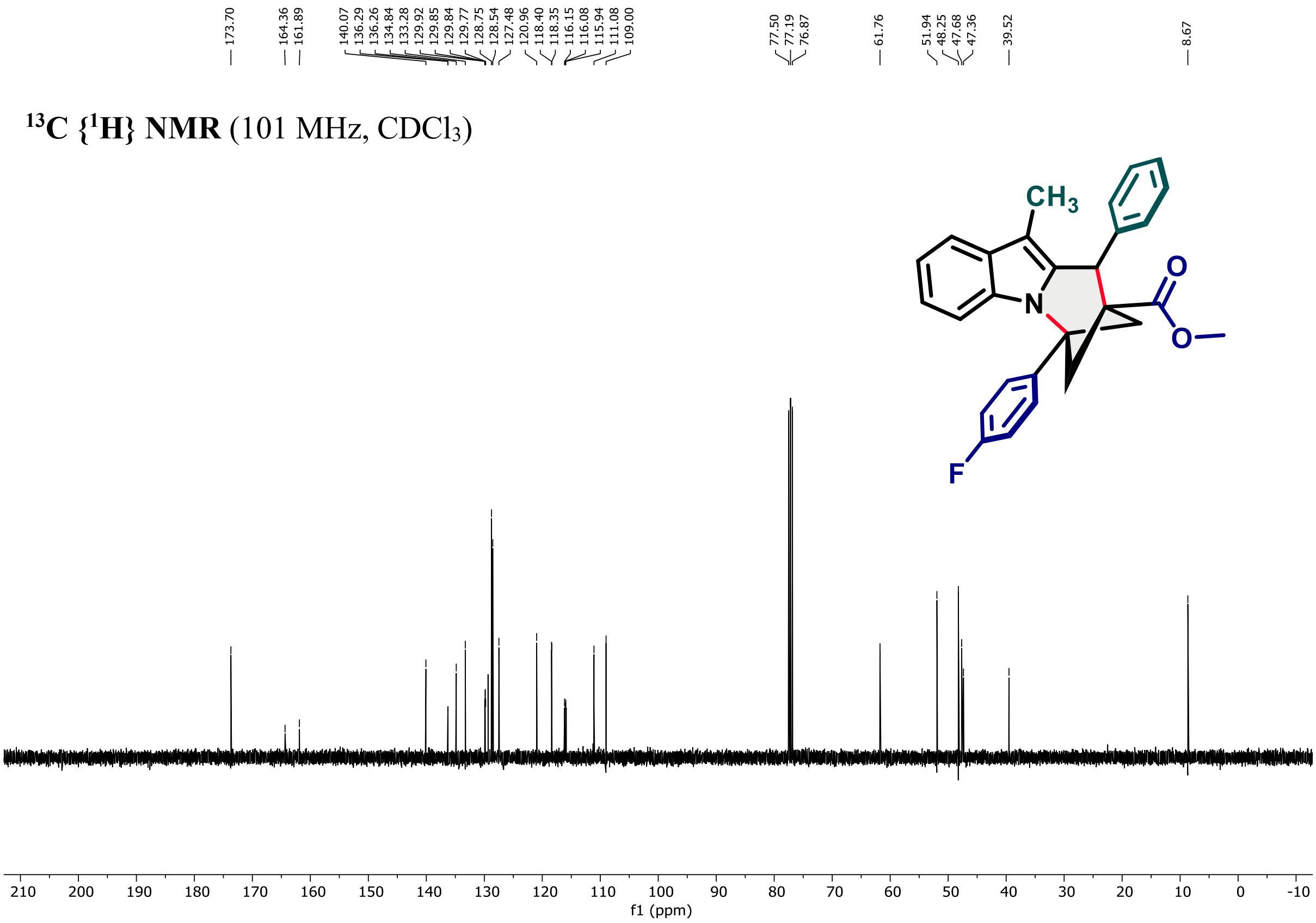

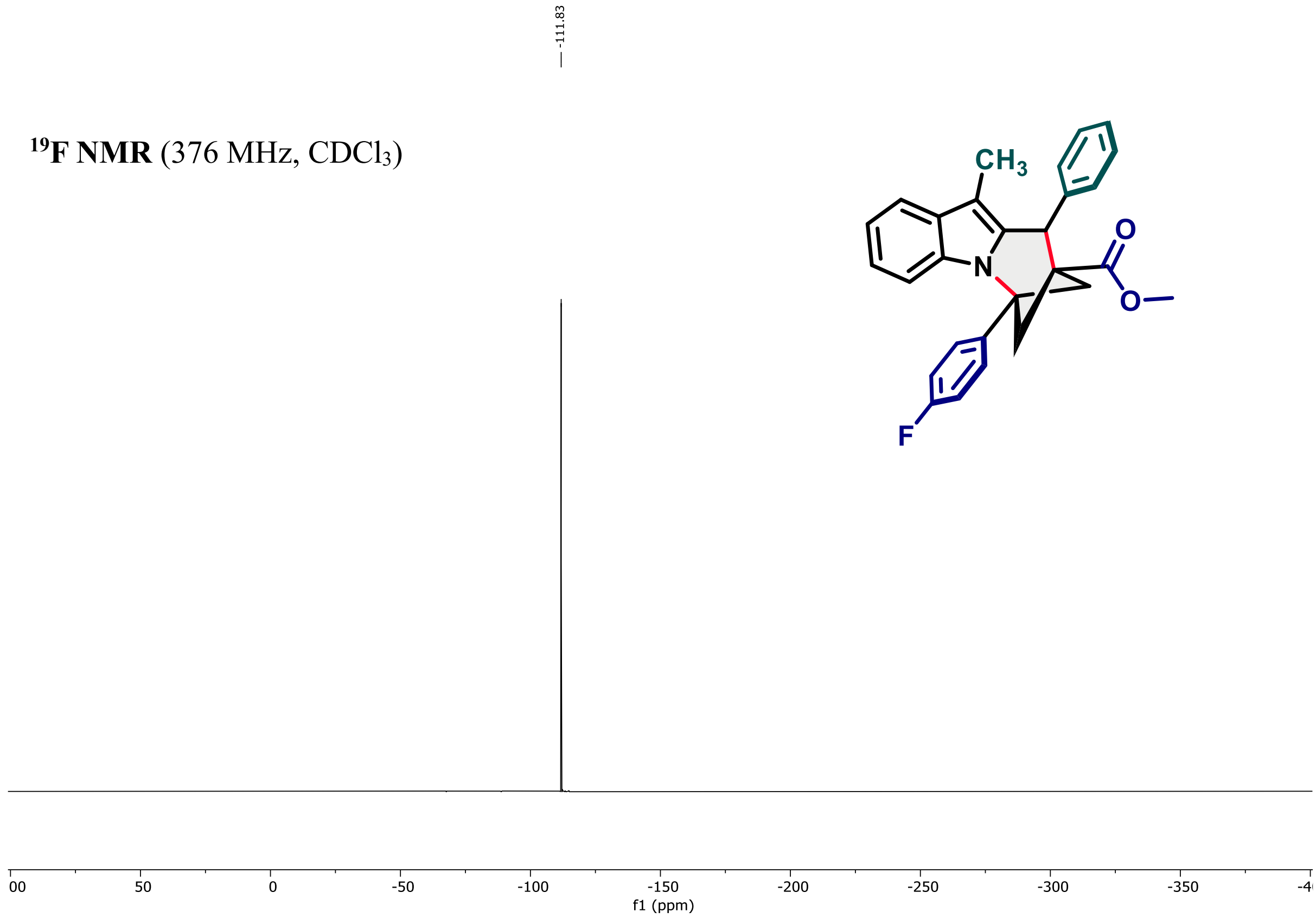

-111.83
^{19}F NMR (376 MHz, CDCl$_3$)
CH3
O
O
N
F
00
50
0
-50
-100
-150
-200
-250
-300
-350
-4
f1 (ppm)

Methyl-6-(4-chlorophenyl)-10-methyl-9-phenyl-6,7-dihydro-6,8-methanopyrido[1,2-a]indole-8(9*H*)-carboxylate (3ag):

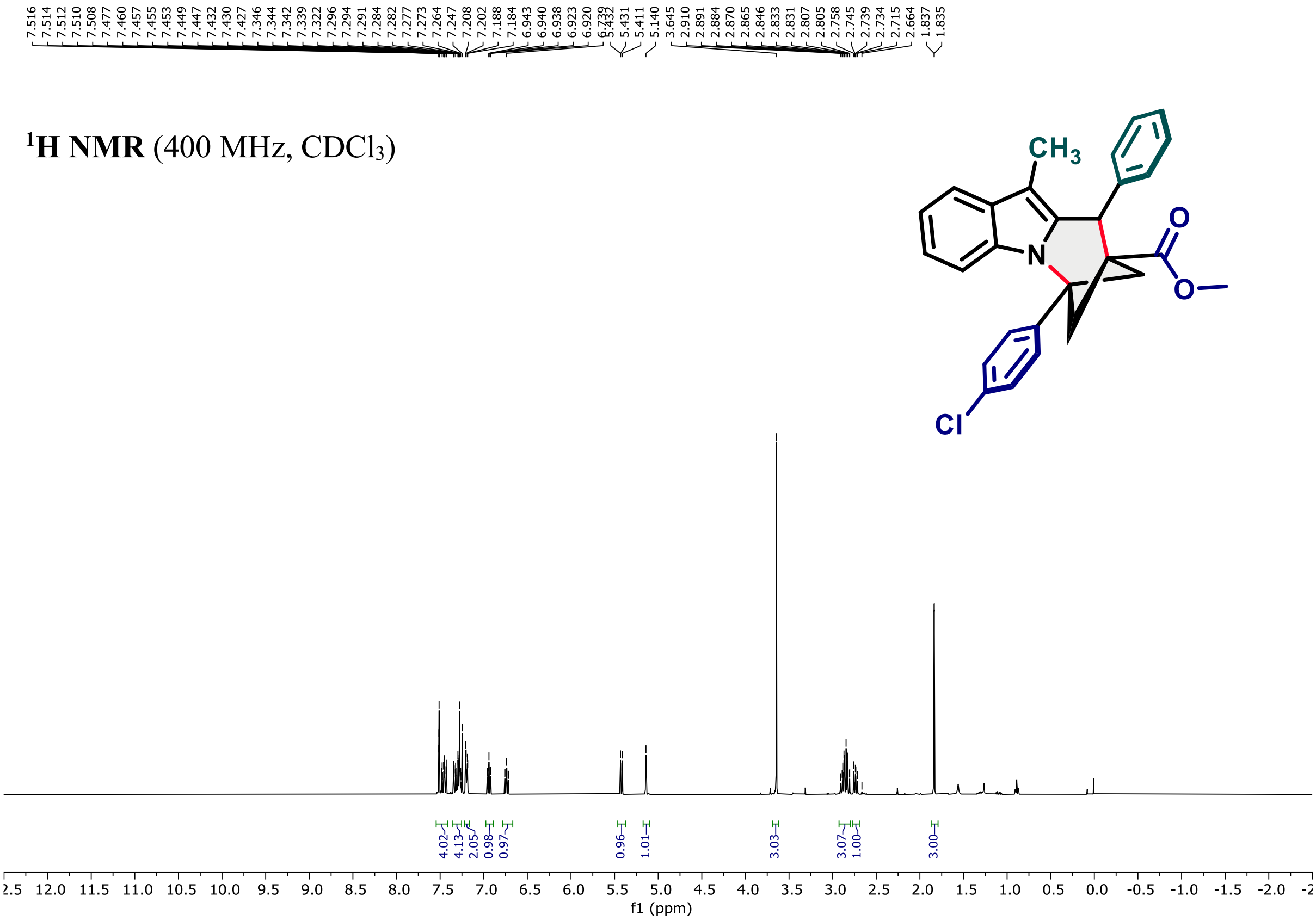

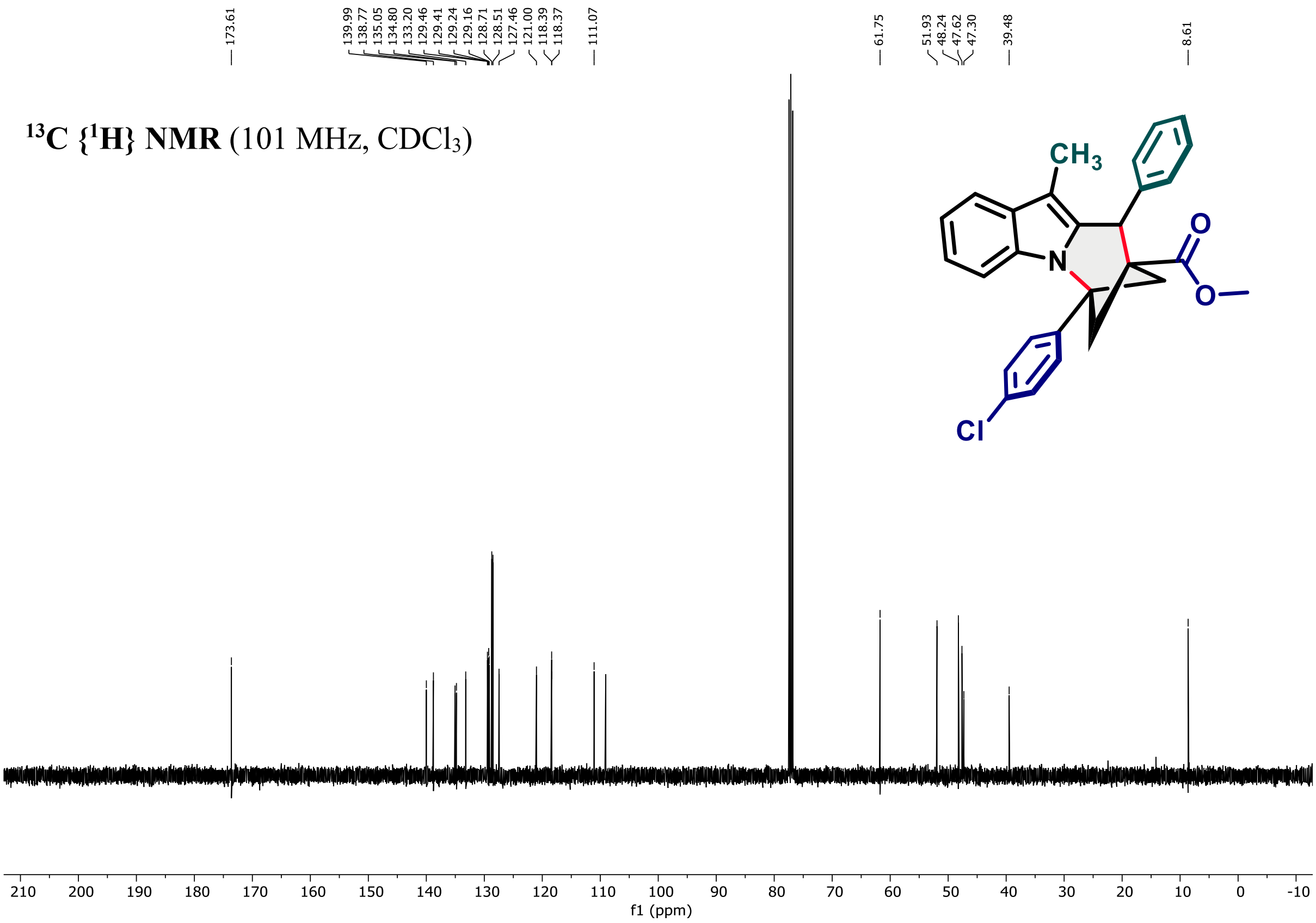

(*Z*)-3,7-dimethylocta-2,6-dien-1-yl-10-methyl-6,9-diphenyl-6,7-dihydro-6,8-methano pyrido[1,2-a]indole-8(9H)-carboxylate (3ai):

^{1}H NMR (400 MHz, $CDCl_3$)

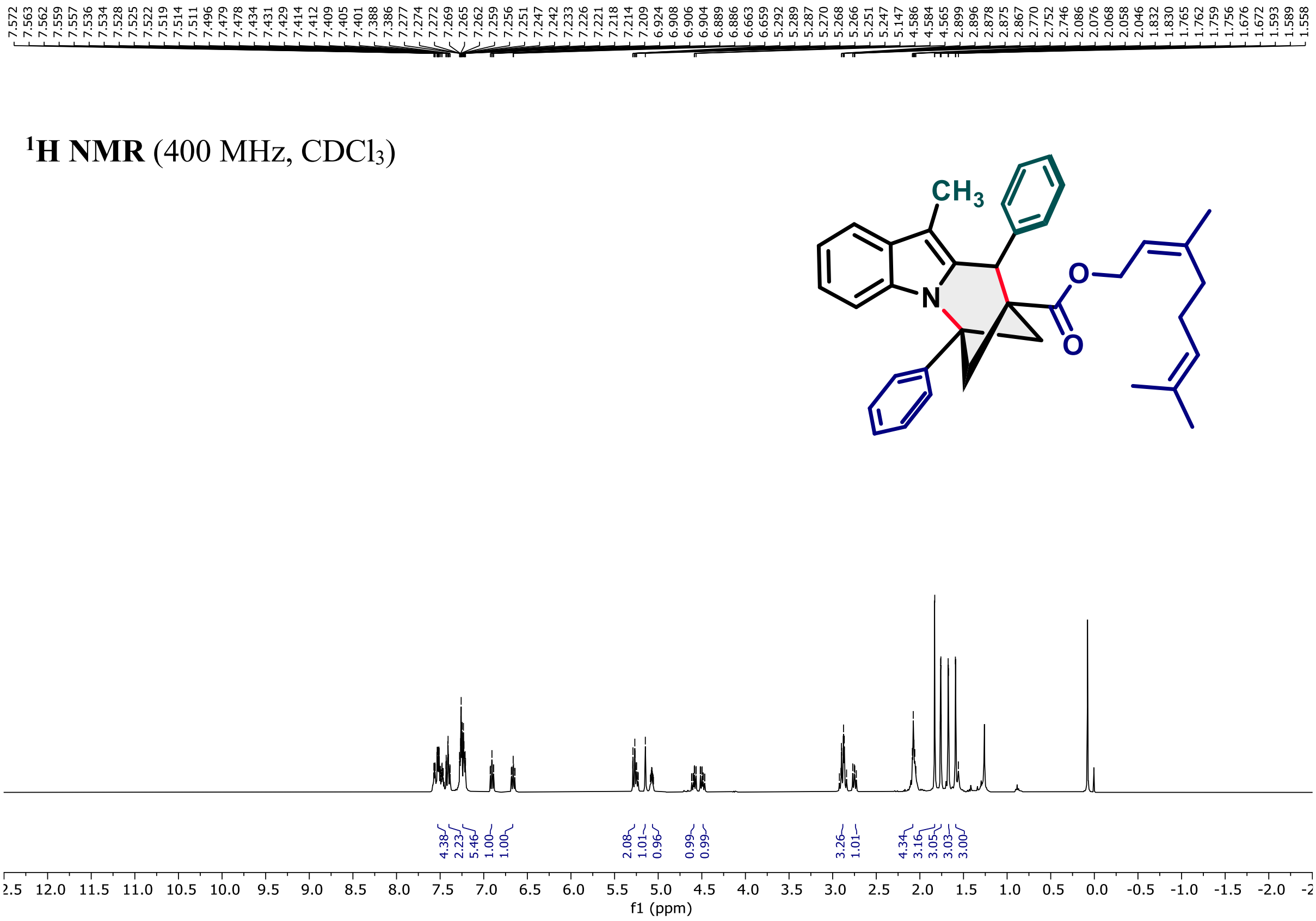

^{13}C {^{1}H} NMR (101 MHz, $CDCl_3$)

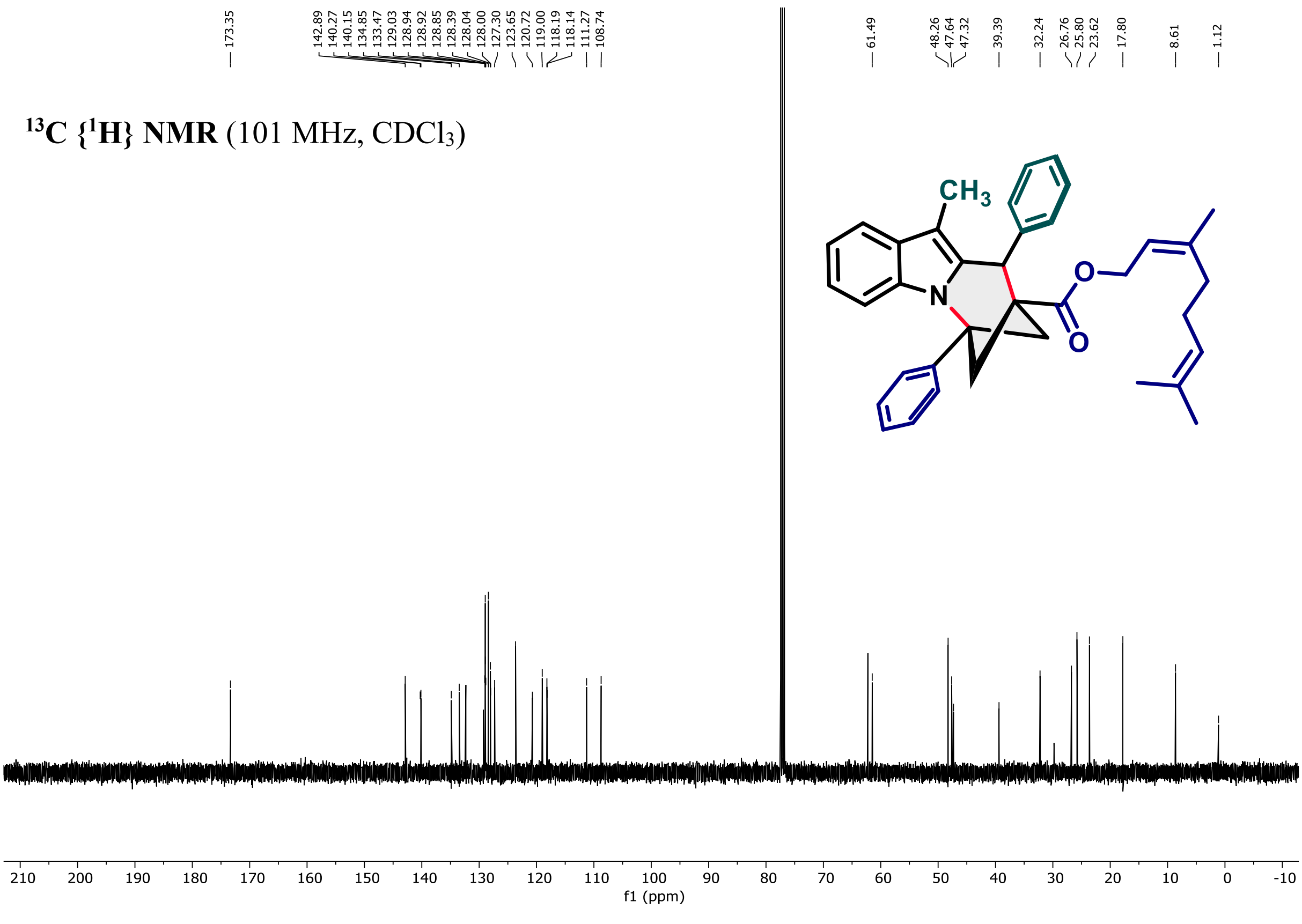

Benzo[d][1,3]dioxol-5-yl-10-methyl-6,9-diphenyl-6,7-dihydro-6,8-methanopyrido[1,2-a]indole-8(9*H*)-carboxylate (3aj):

^{1}H NMR (400 MHz, $CDCl_3$)

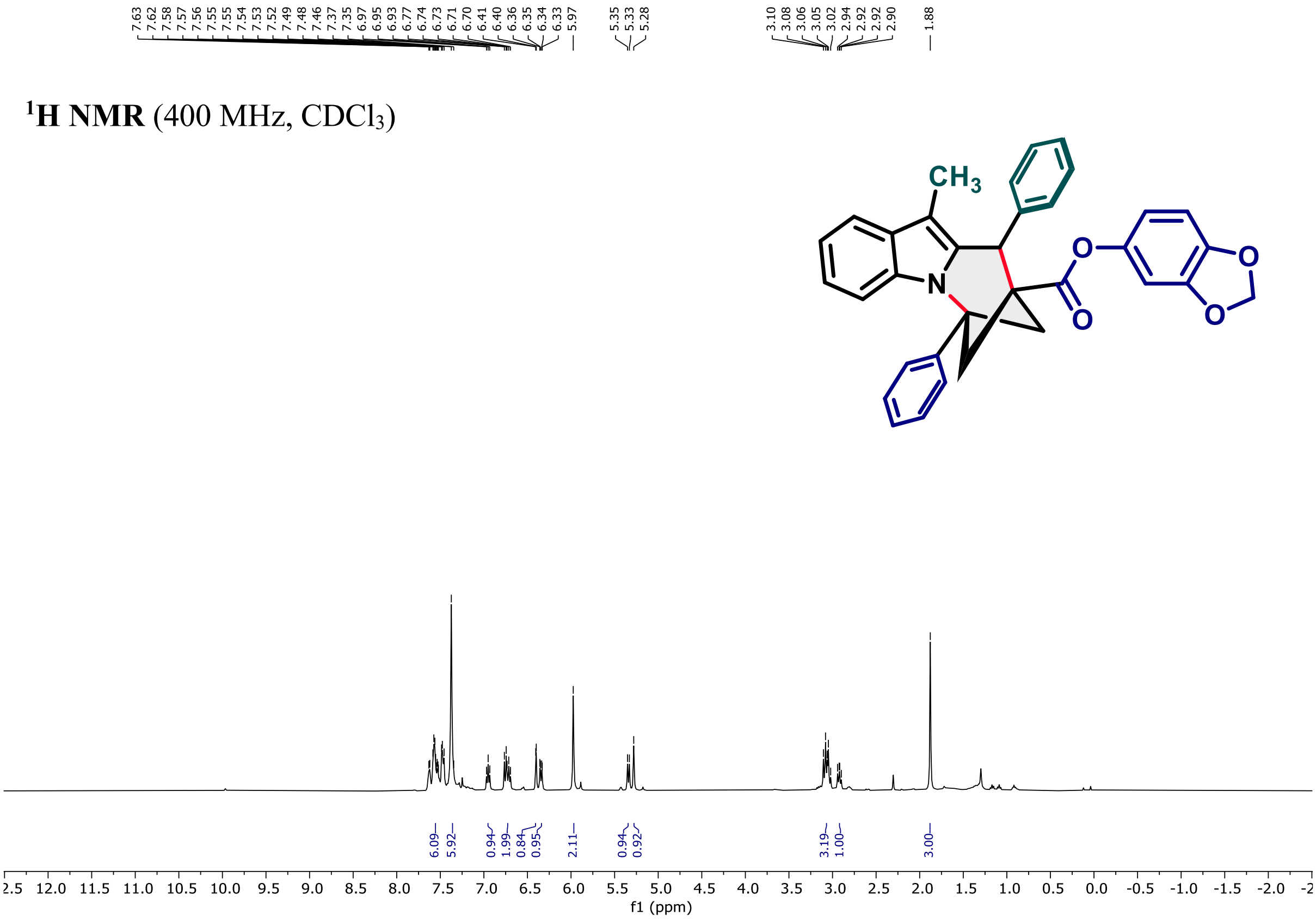

^{13}C {^{1}H} NMR (101 MHz, $CDCl_3$)

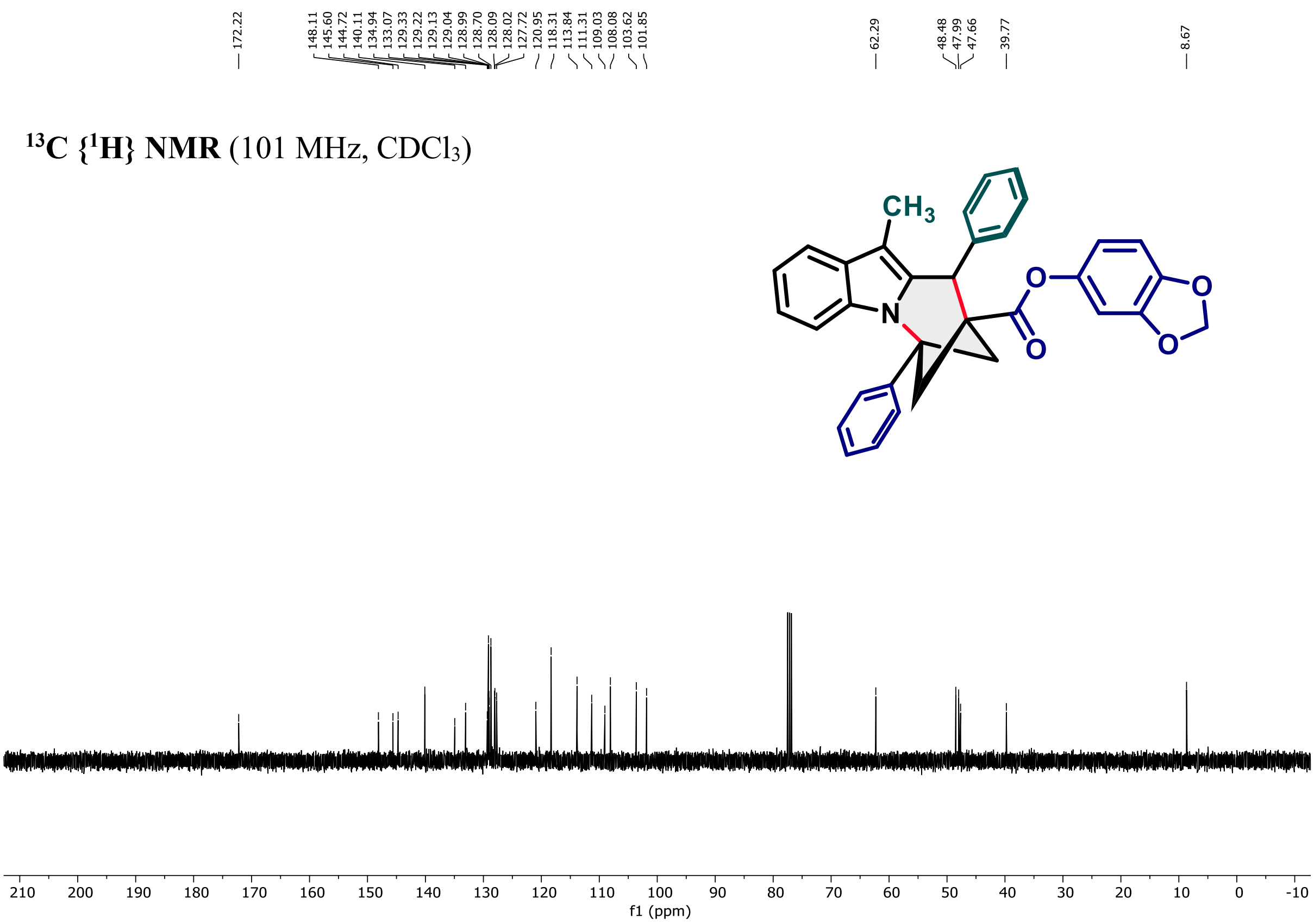

4-Allyl-2-methoxyphenyl-10-methyl-6,9-diphenyl-6,7-dihydro-6,8-methanopyrido[1,2-a]indole-8(9*H*)-carboxylate (3ak):

^{1}H NMR (400 MHz, $CDCl_3$)

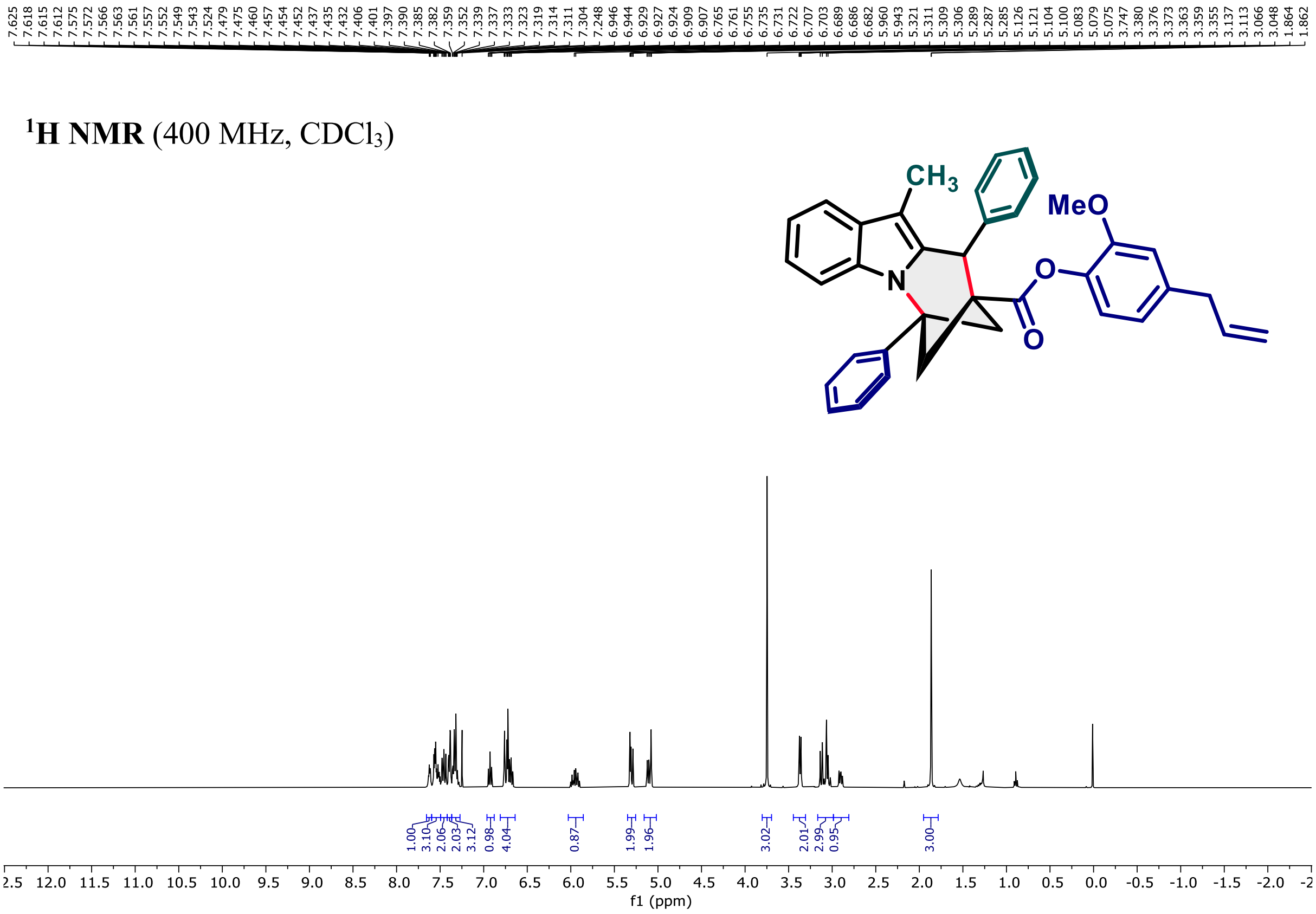

^{13}C {^{1}H} NMR (101 MHz, $CDCl_3$)

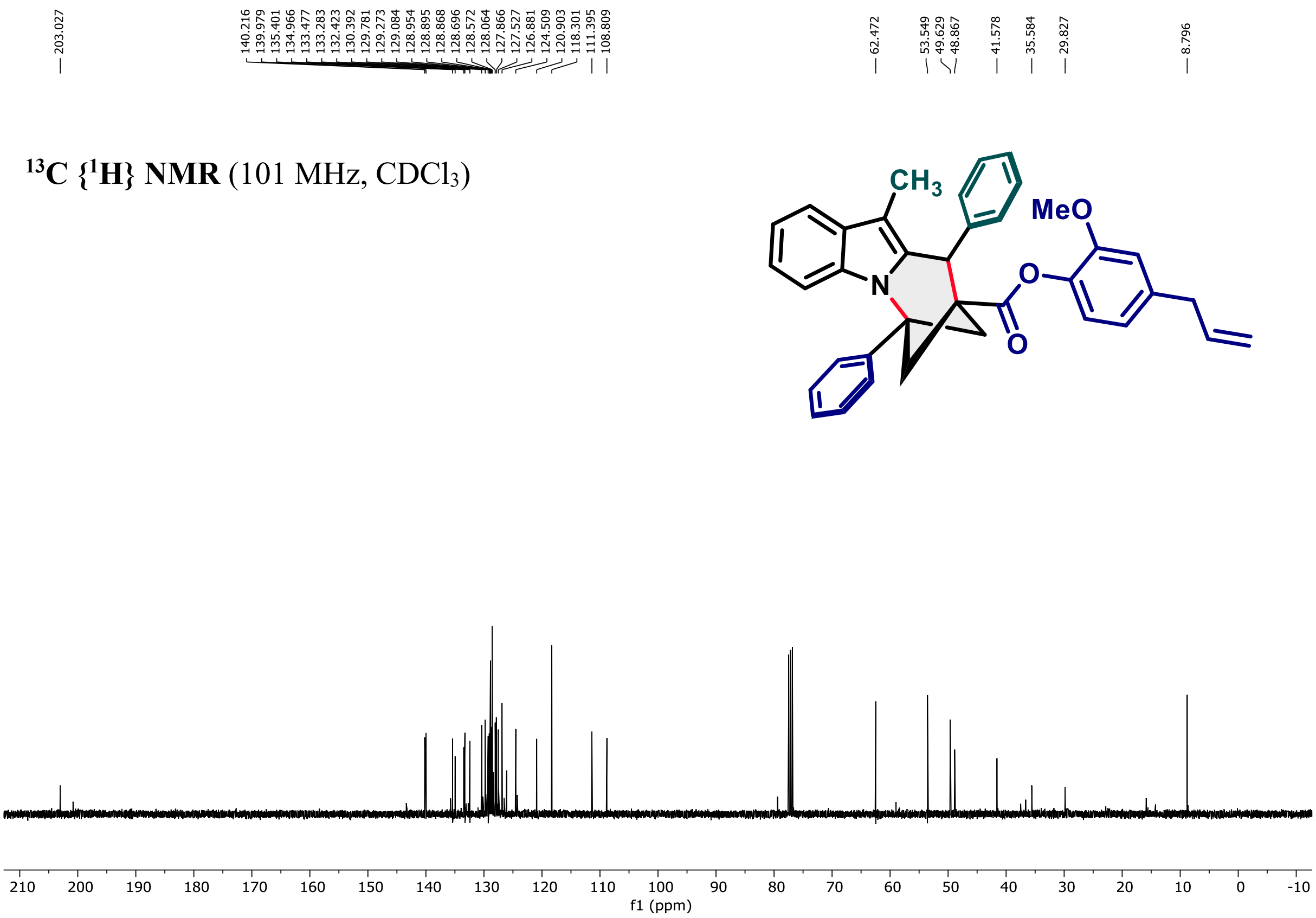

(3,5-dimethyl-1*H*-pyrazol-1-yl)(-10-methyl-6,9-diphenyl-6,7-dihydro-6,8-methanopyrido [1,2-a]indol-8(9*H*)-yl)methanone (3al):

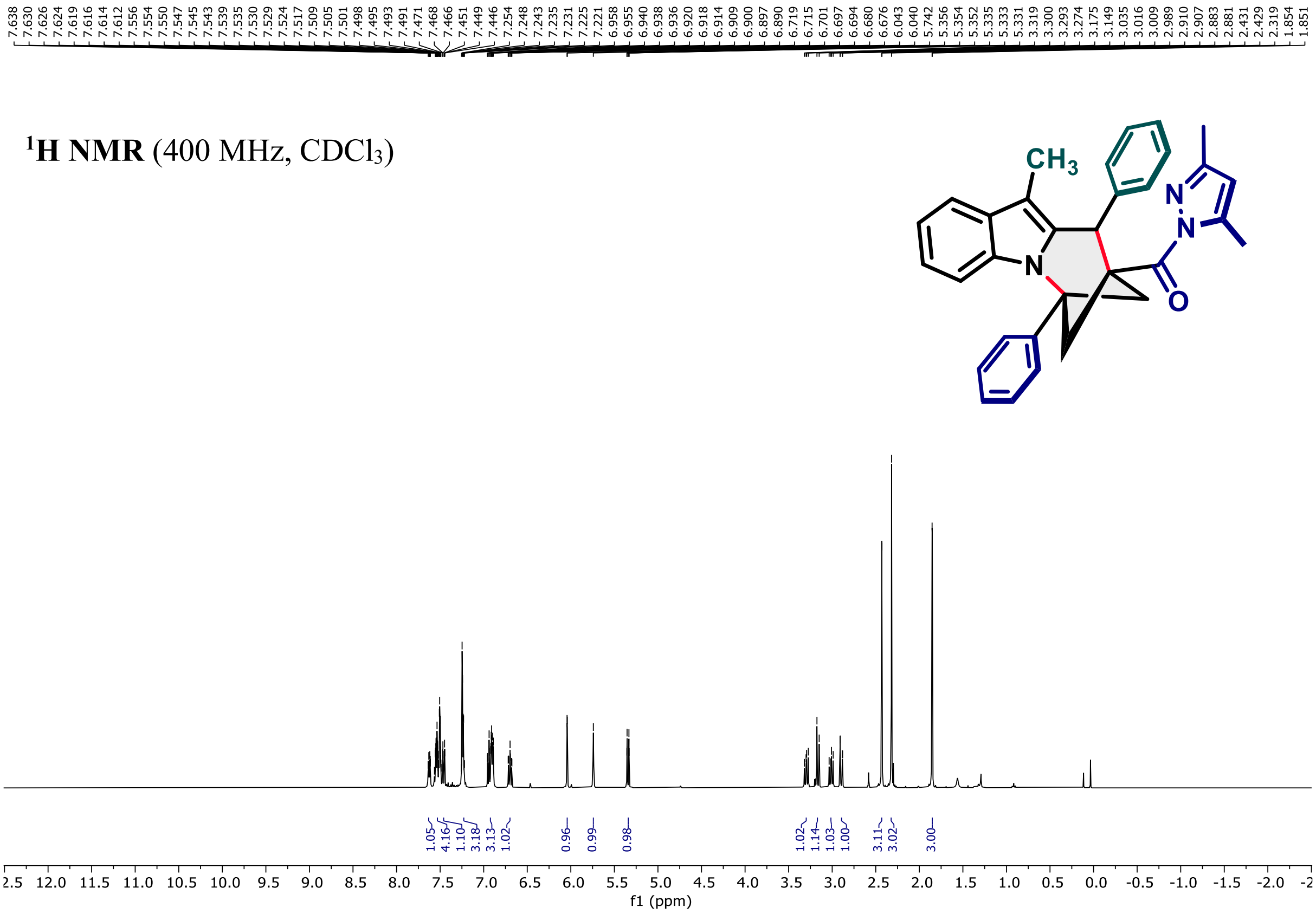

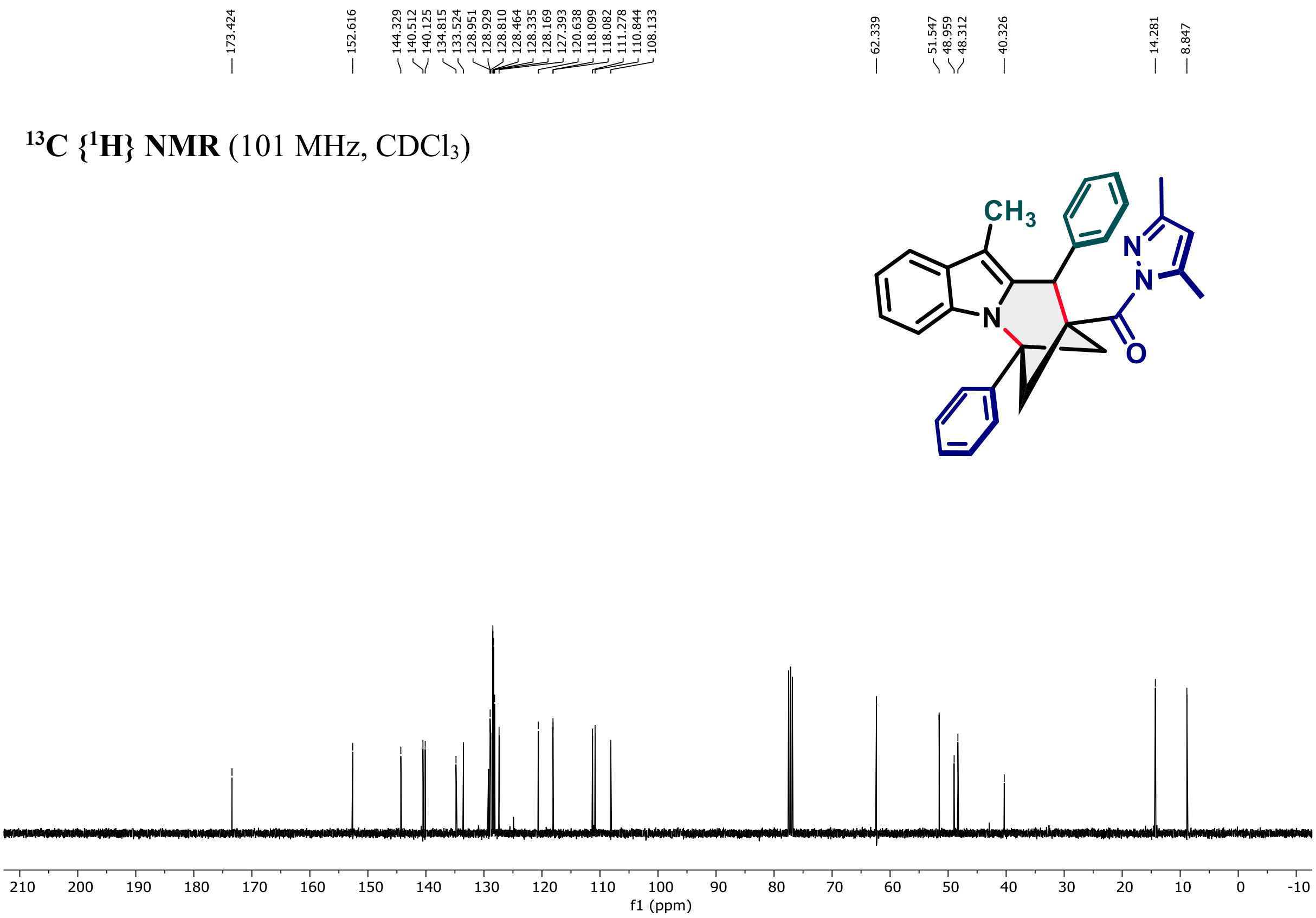

(10-Methyl-6,9-diphenyl-6,7-dihydro-6,8-methanopyrido[1,2-a]indol-8(9*H*)-yl) (naphthalen-2-yl)methanone (3am):

^{1}H NMR (400 MHz, $CDCl_3$)

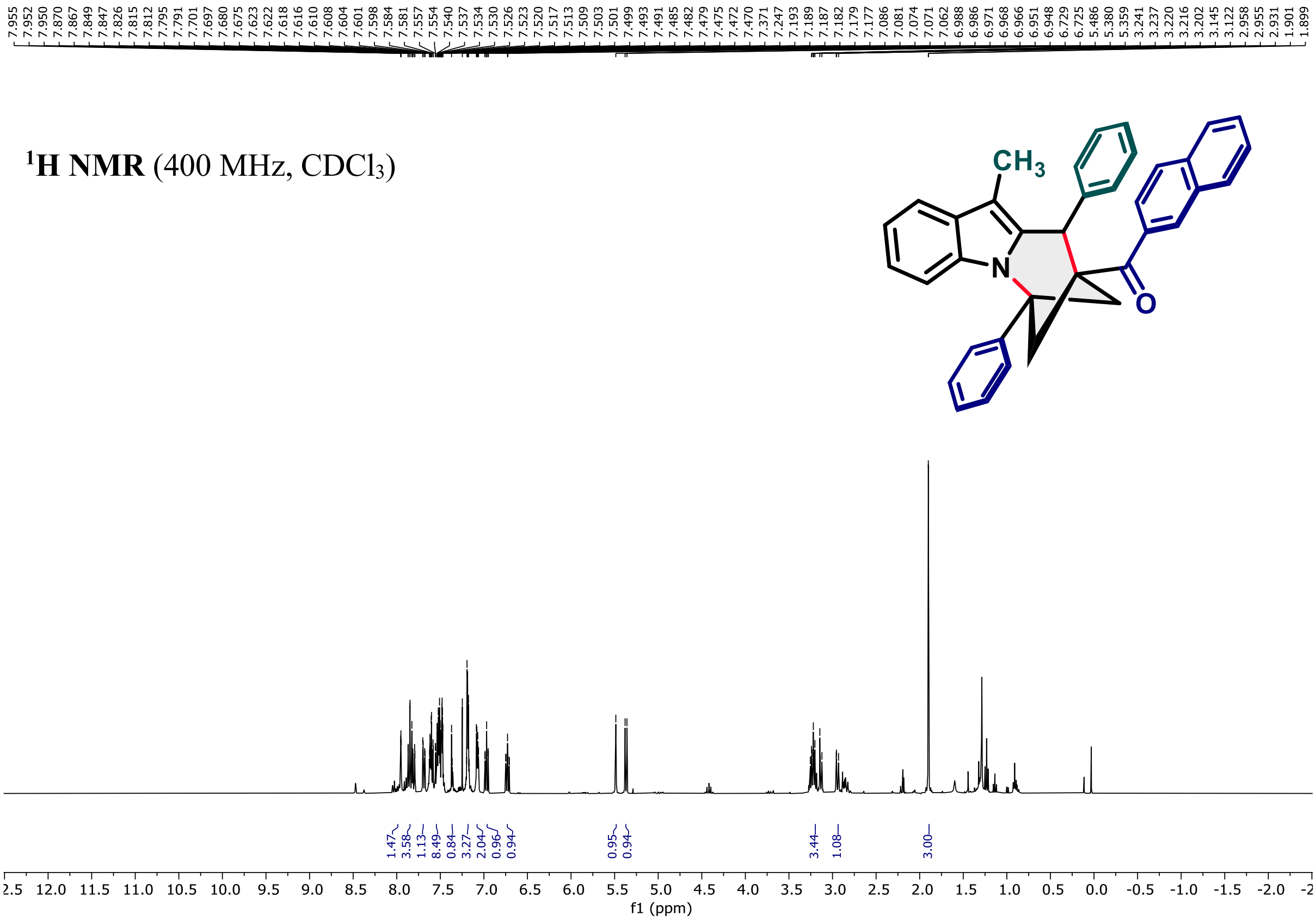

^{13}C {^{1}H} NMR (101 MHz, $CDCl_3$)

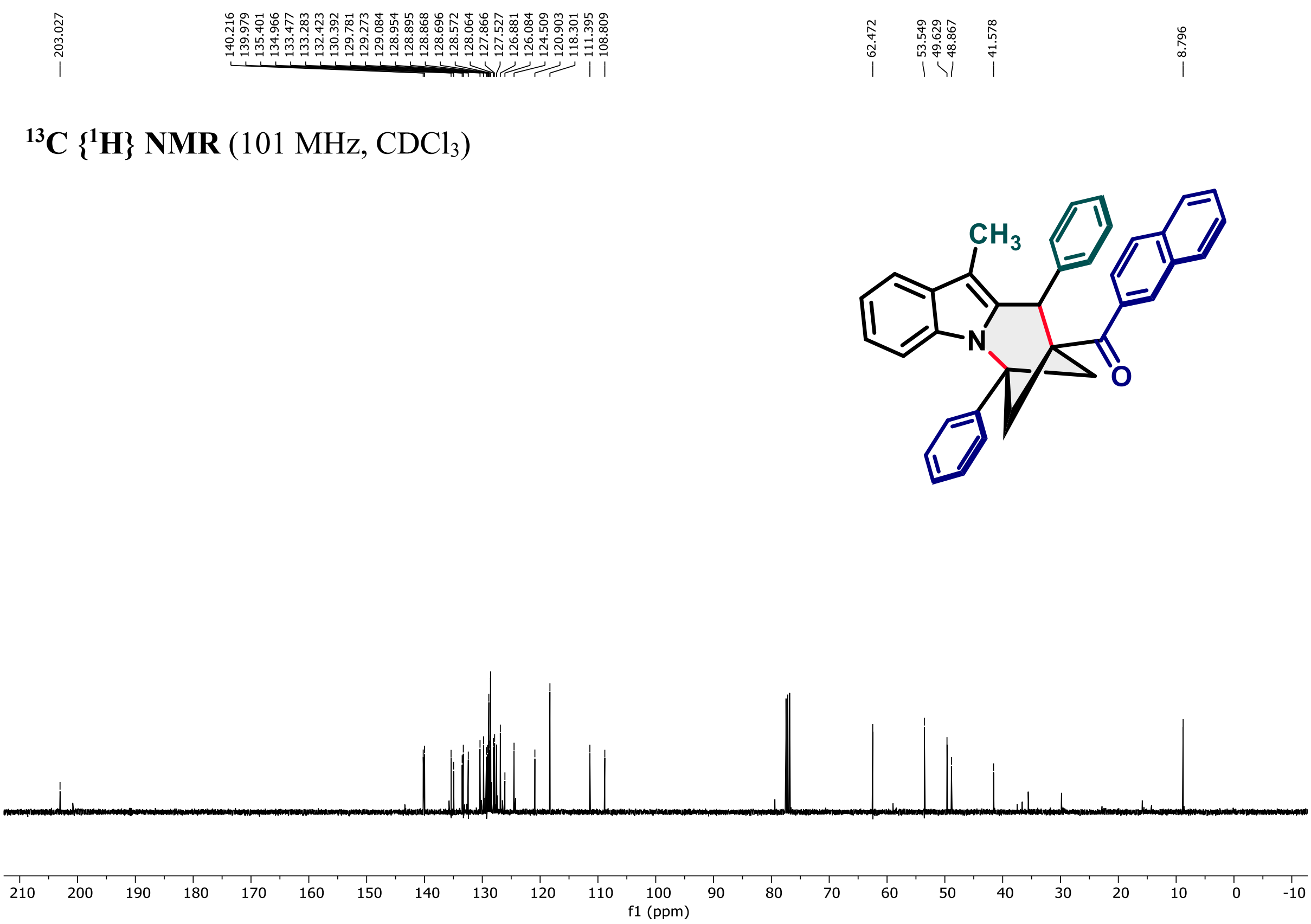

(3-Methoxyphenyl)(10-methyl-6,9-diphenyl-6,7-dihydro-6,8-methanopyrido[1,2-a]indol-8(9*H*)-yl)methanone (3an):

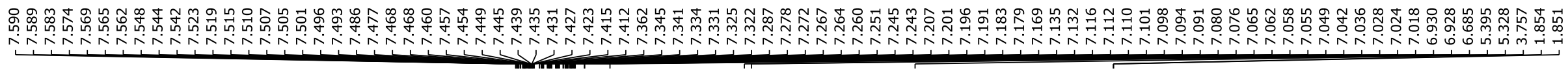

^{1}H NMR (400 MHz, $CDCl_3$)

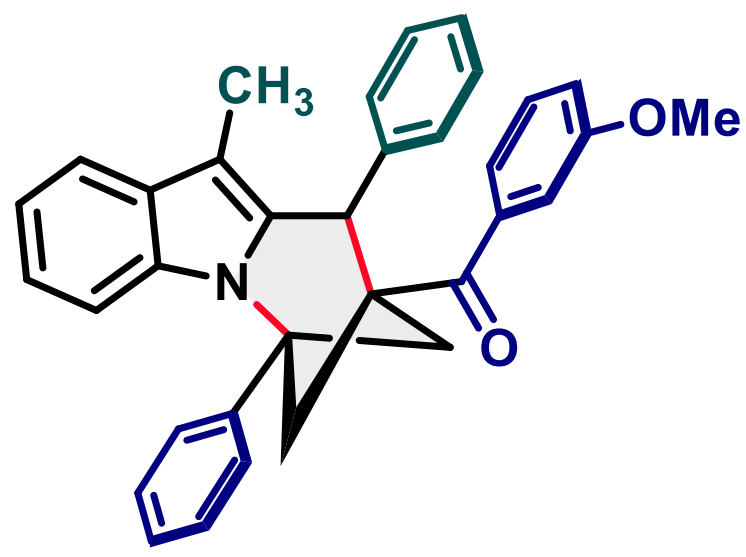

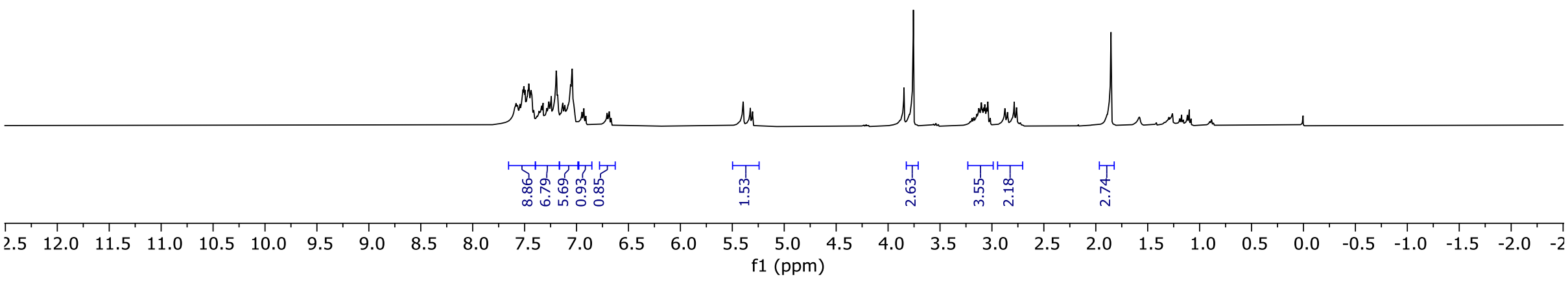

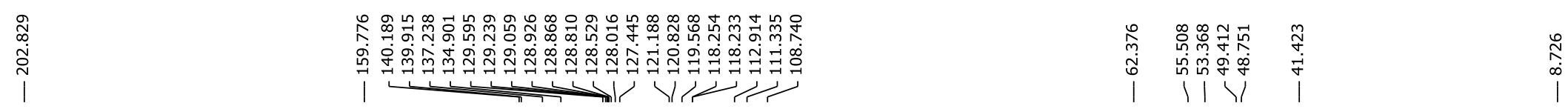

^{13}C {^{1}H} NMR (101 MHz, $CDCl_3$)

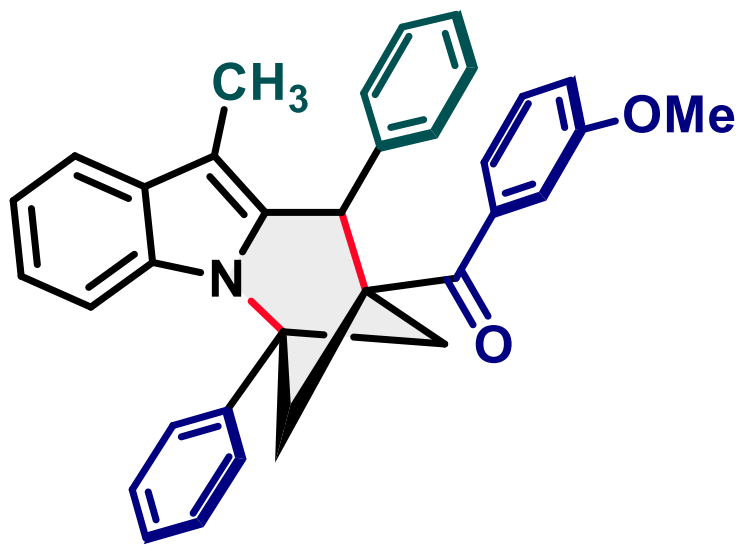

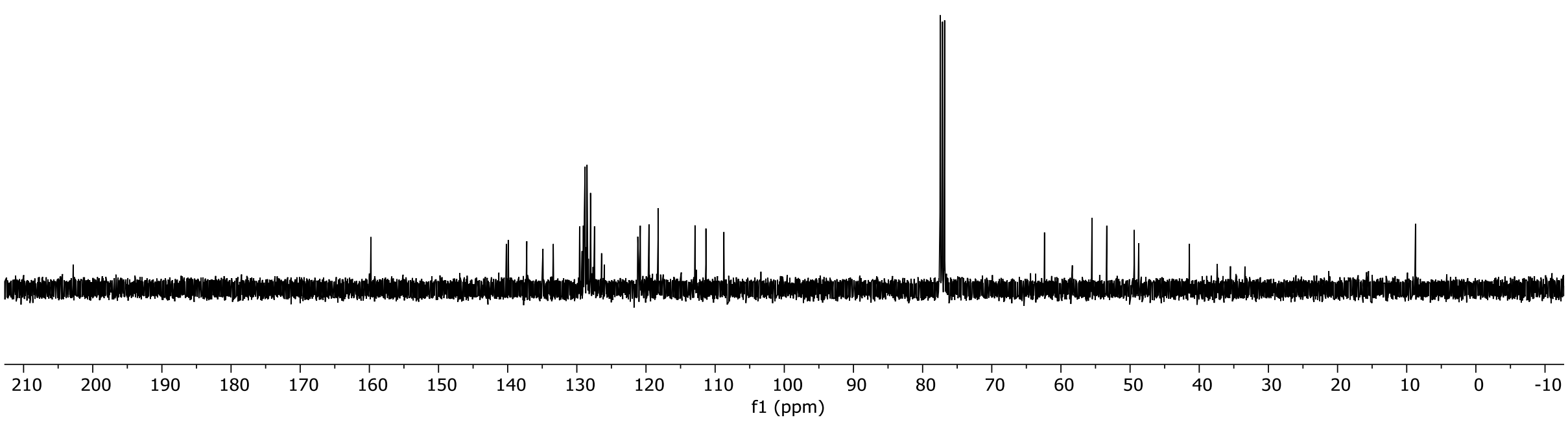

(10-Methyl-6,9-diphenyl-6,7-dihydro-6,8-methanopyrido[1,2-a]indol-8(9*H*)-yl)diphenylmethanol (4):

^{1}H NMR (400 MHz, $CDCl_3$)

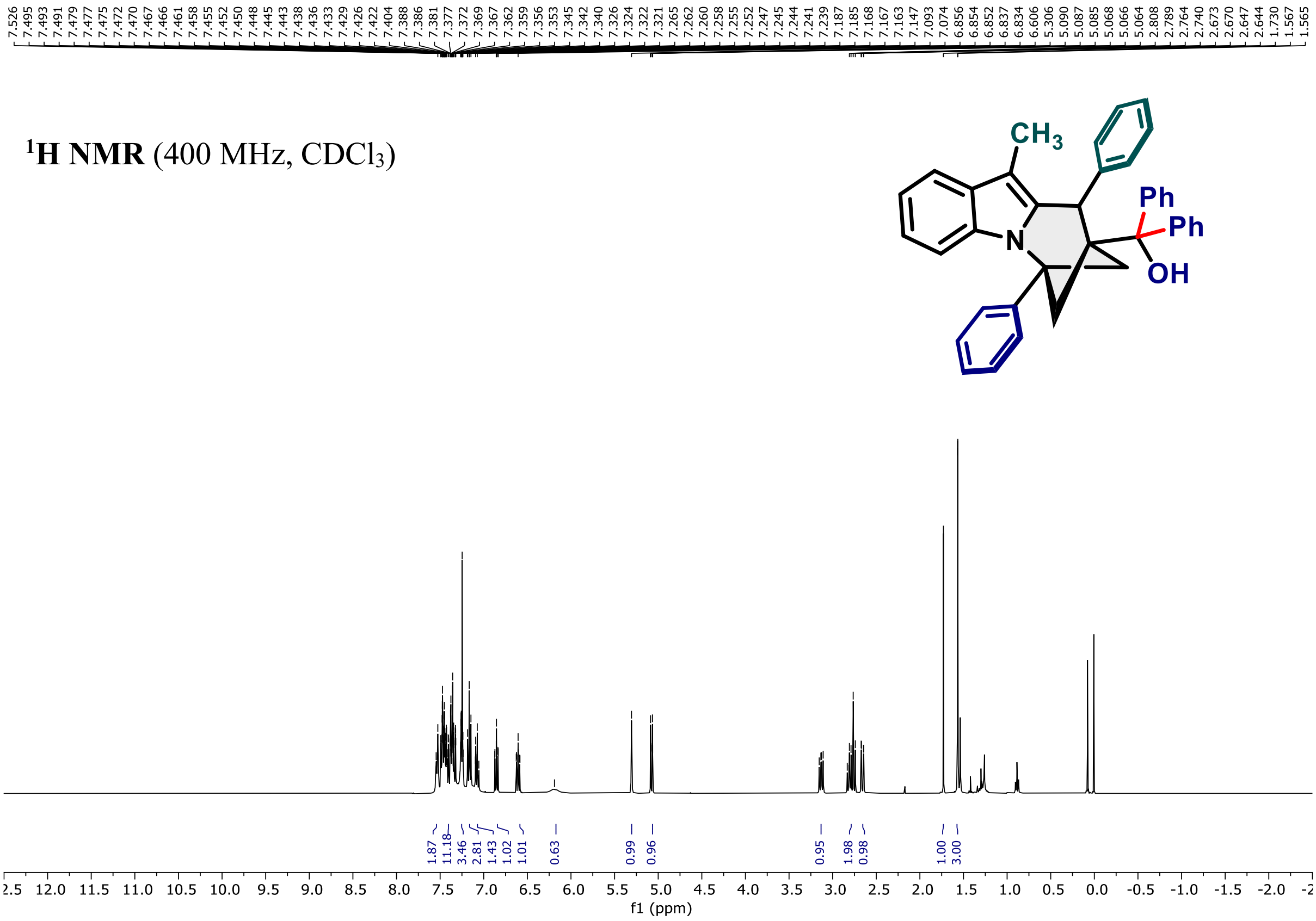

^{13}C {^{1}H} NMR (101 MHz, $CDCl_3$)

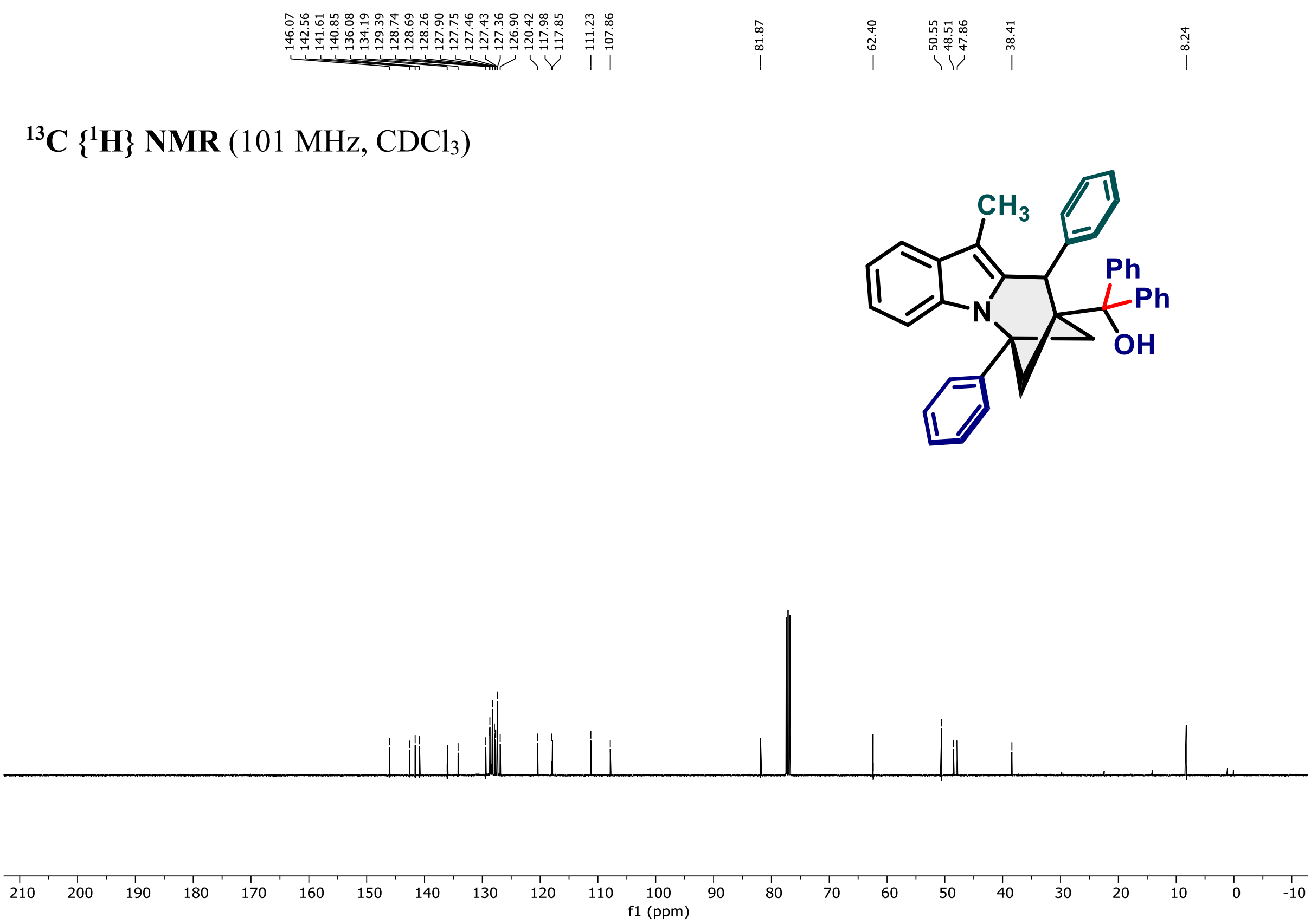

(10-Methyl-6,9-diphenyl-6,7-dihydro-6,8-methanopyrido[1,2-a]indol-8(9*H*)-yl)methanol (5):

^{1}H NMR (400 MHz, $CDCl_3$)

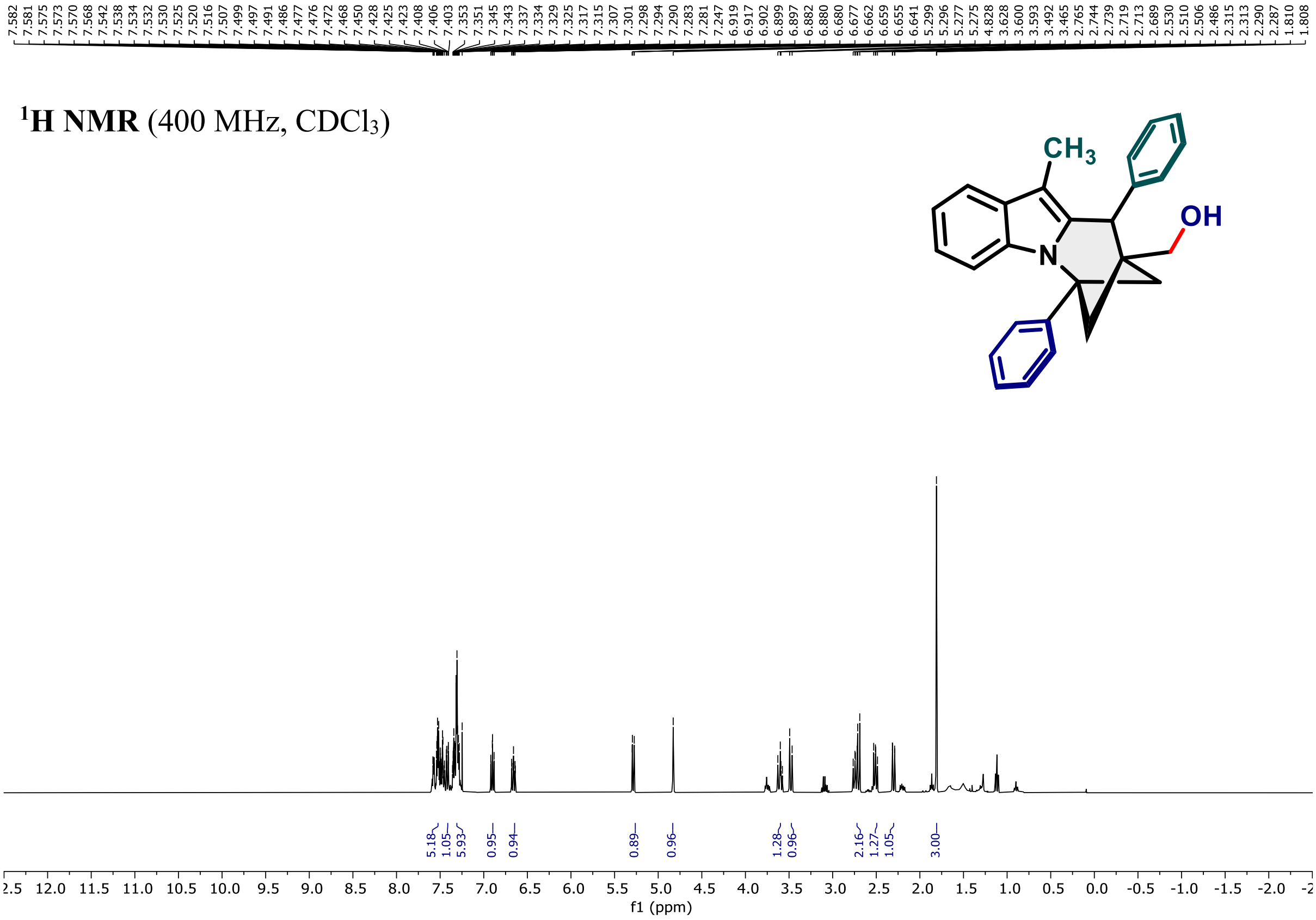

^{13}C {^{1}H} NMR (101 MHz, $CDCl_3$)

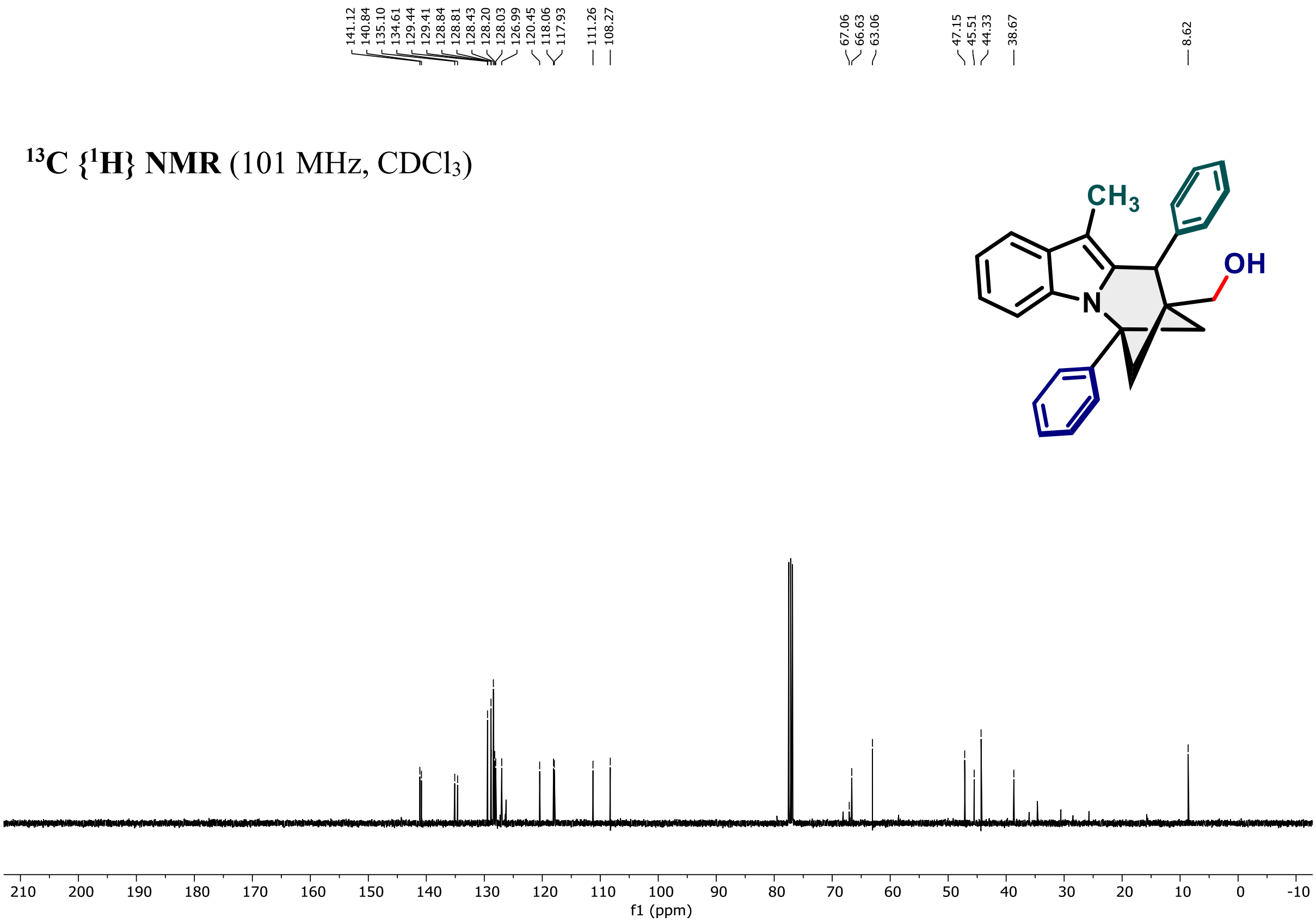

8-(Bromomethyl)-10-methyl-6,9-diphenyl-6,7,8,9-tetrahydro-6,8-methanopyrido[1,2-a]indole (6):

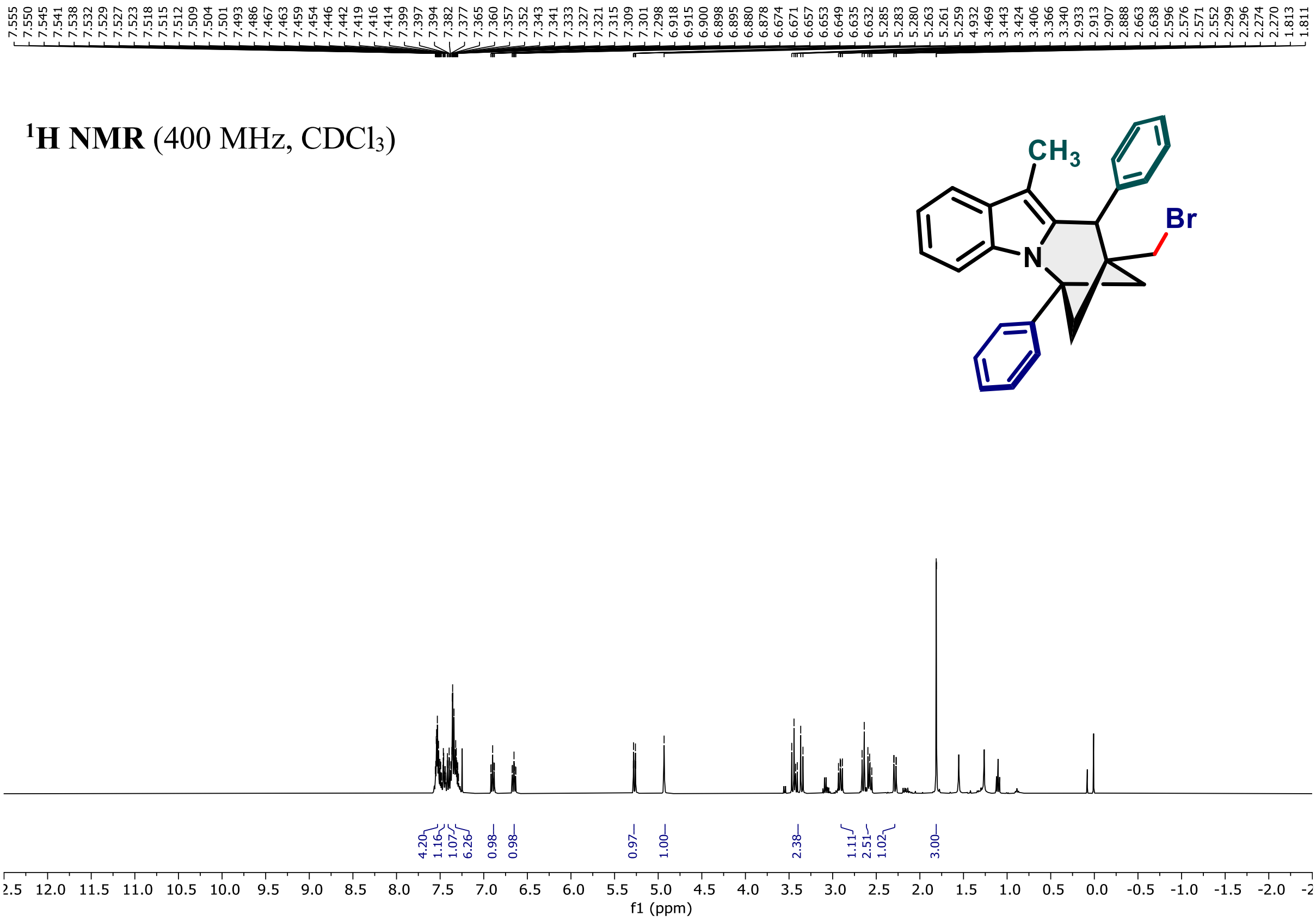

^{1}H NMR (400 MHz, $CDCl_3$)

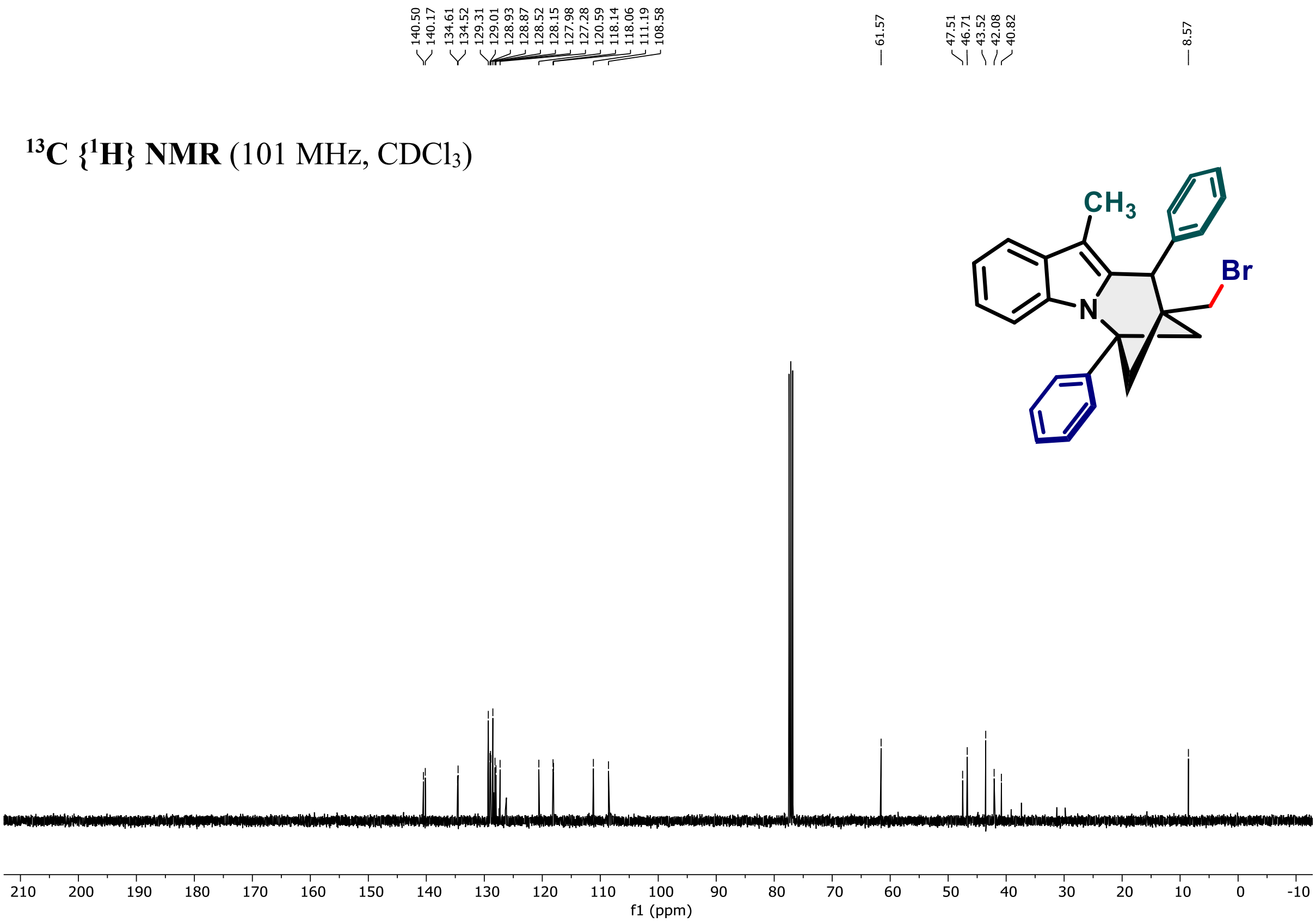

^{13}C {^{1}H} NMR (101 MHz, $CDCl_3$)

Methyl-2,6,9,10-tetraphenyl-6,7-dihydro-6,8-methanopyrido[1,2-a]indole-8(9*H*)-carboxylate (7):

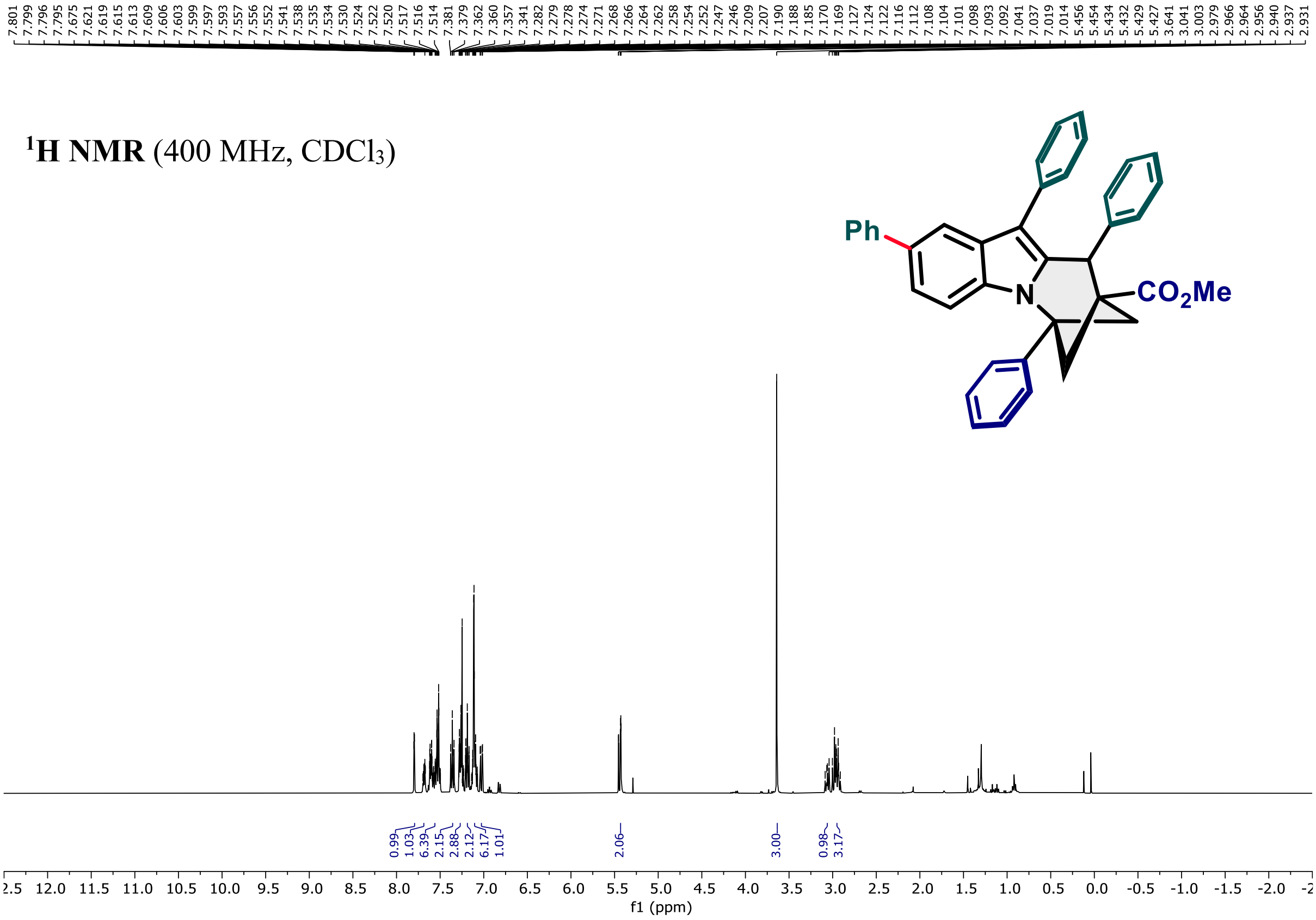

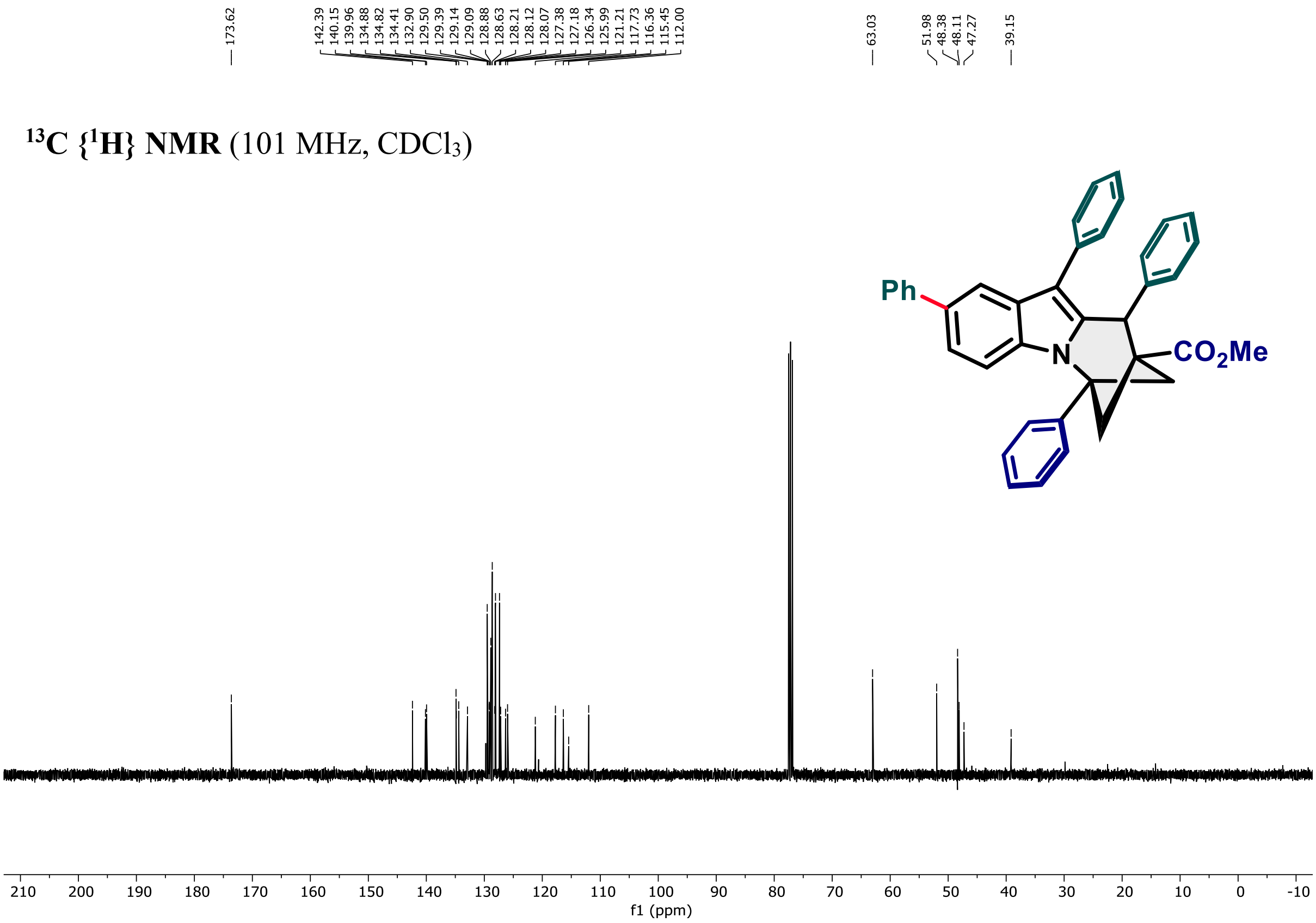

10-Methyl-6,9-diphenyl-6,7-dihydro-6,8-methanopyrido[1,2-a]indole-8(9*H*)-carbaldehyde (8):

^{1}H NMR (400 MHz, $CDCl_3$)

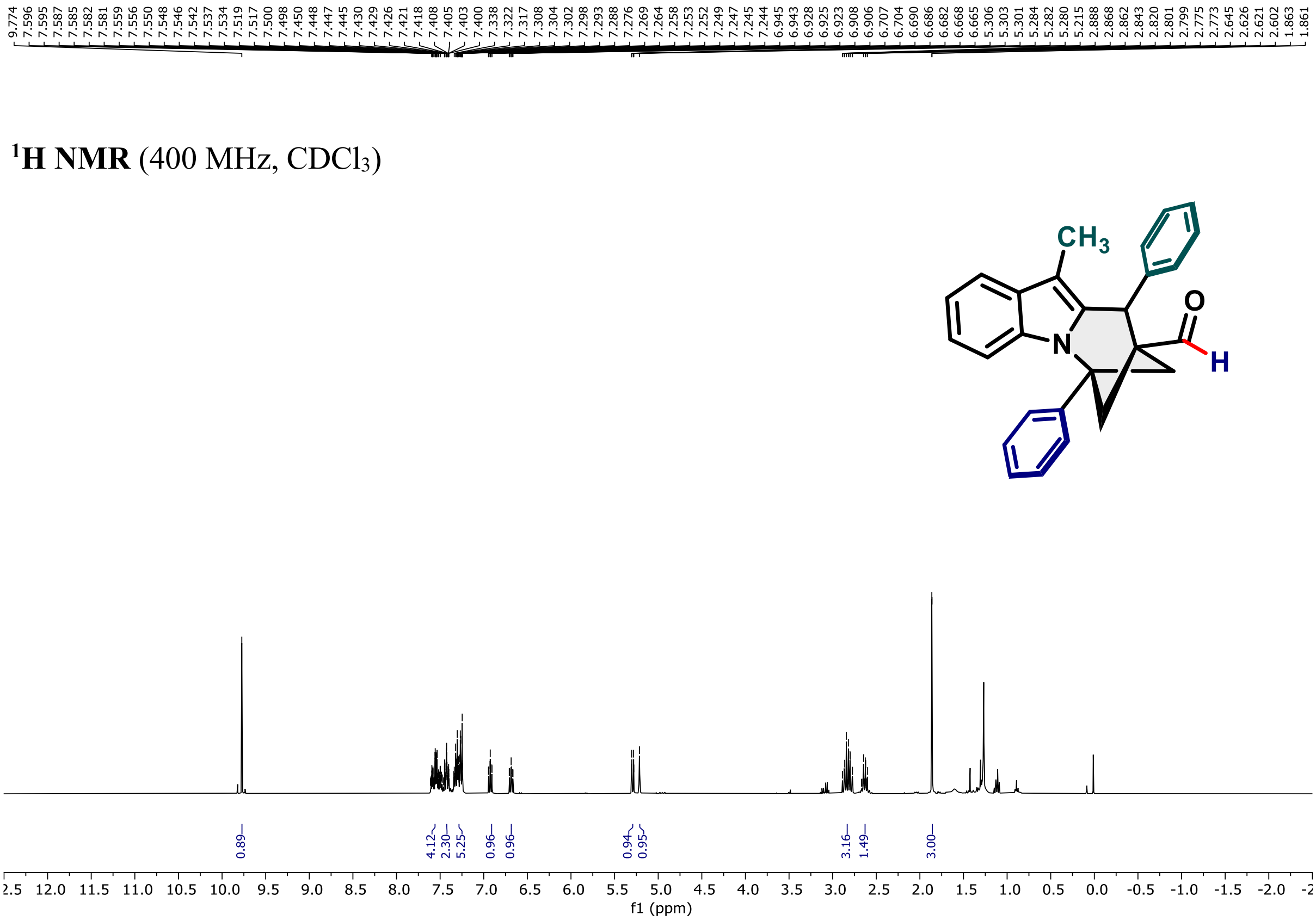

^{13}C {^{1}H} NMR (101 MHz, $CDCl_3$)

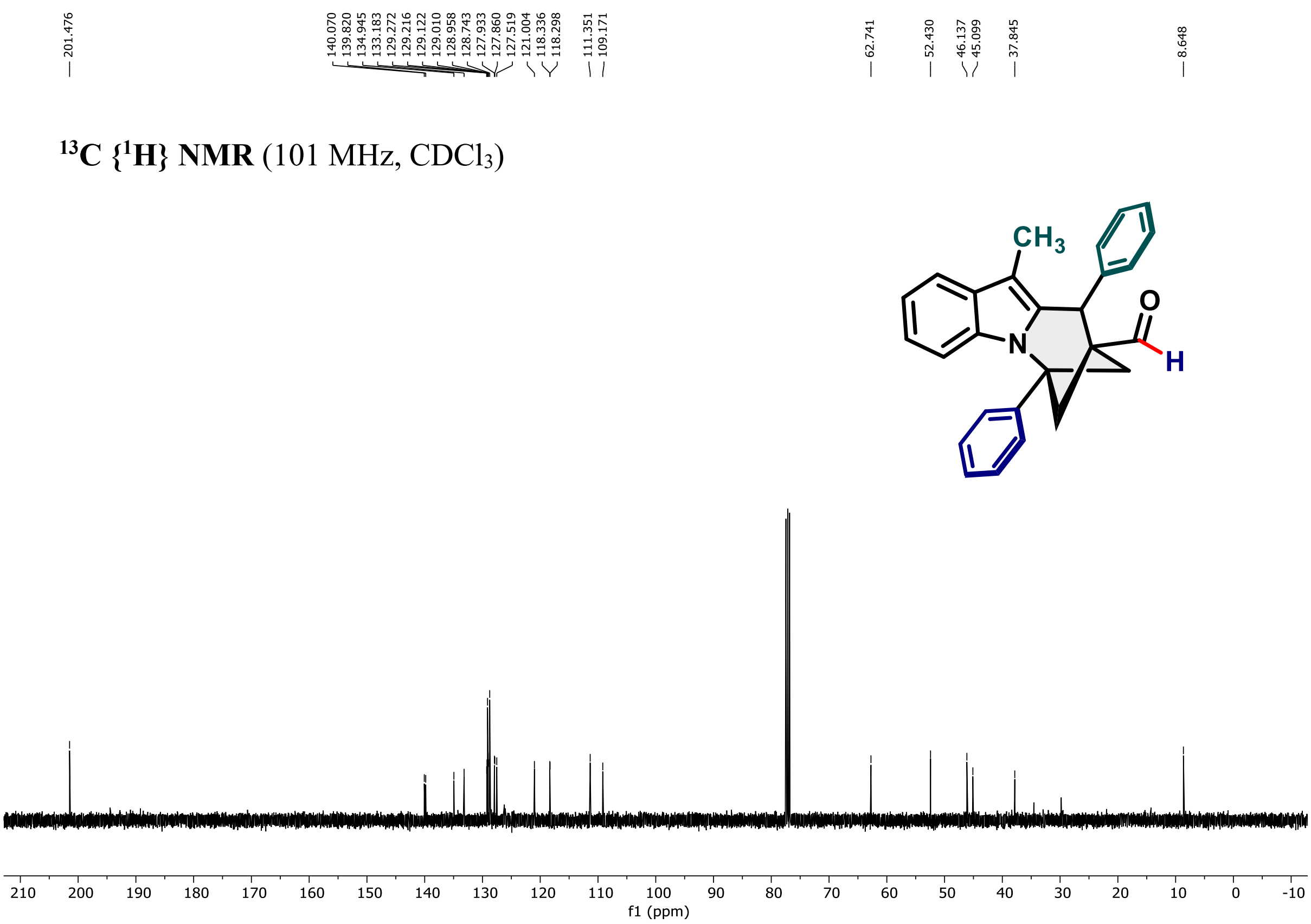

1-(10-Methyl-6,9-diphenyl-6,7-dihydro-6,8-methanopyrido[1,2-a]indol-8(9*H*)-yl)-3-phenylprop-2-yn-1-one (9):

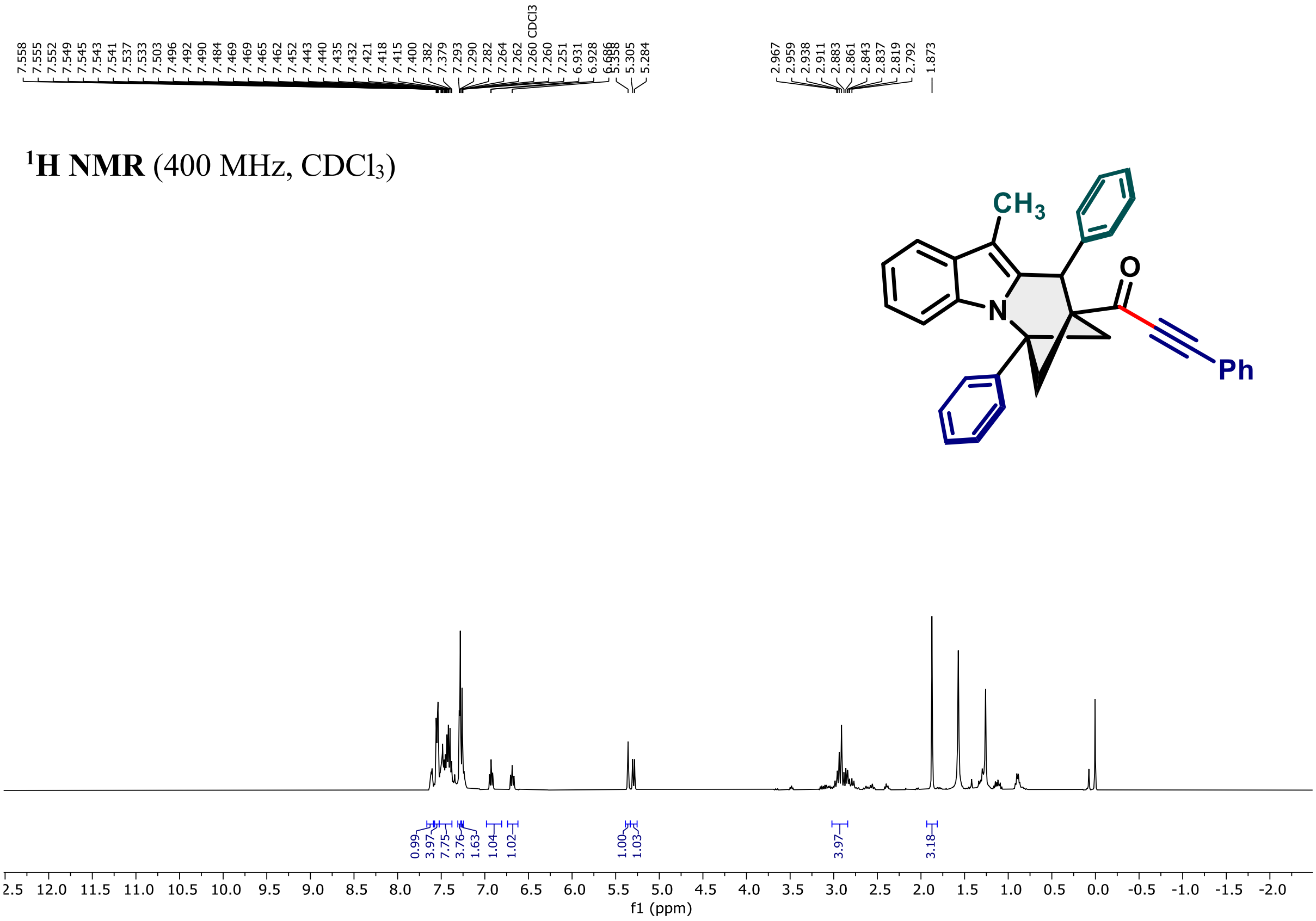

^{1}H NMR (400 MHz, $CDCl_3$)

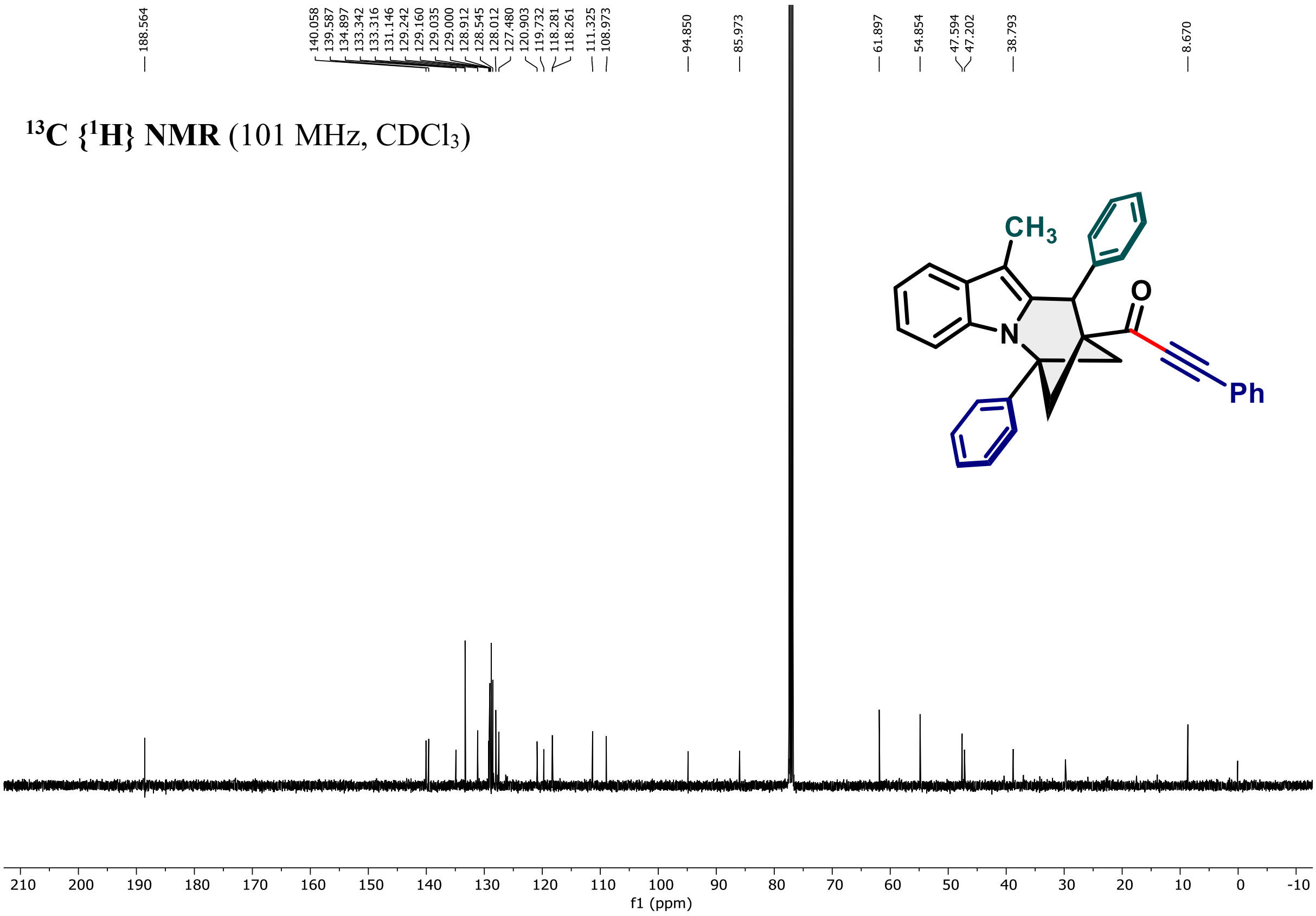

^{13}C {^{1}H} NMR (101 MHz, $CDCl_3$)

Methyl 1,1,2-triphenyl-1,2,3,9-tetrahydro-4*H*-2,4-methanocarbazole-4-carboxylate (10):

^{1}H NMR (400 MHz, $CDCl_3$)

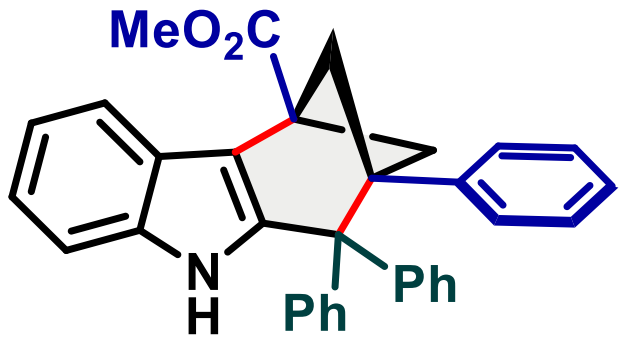

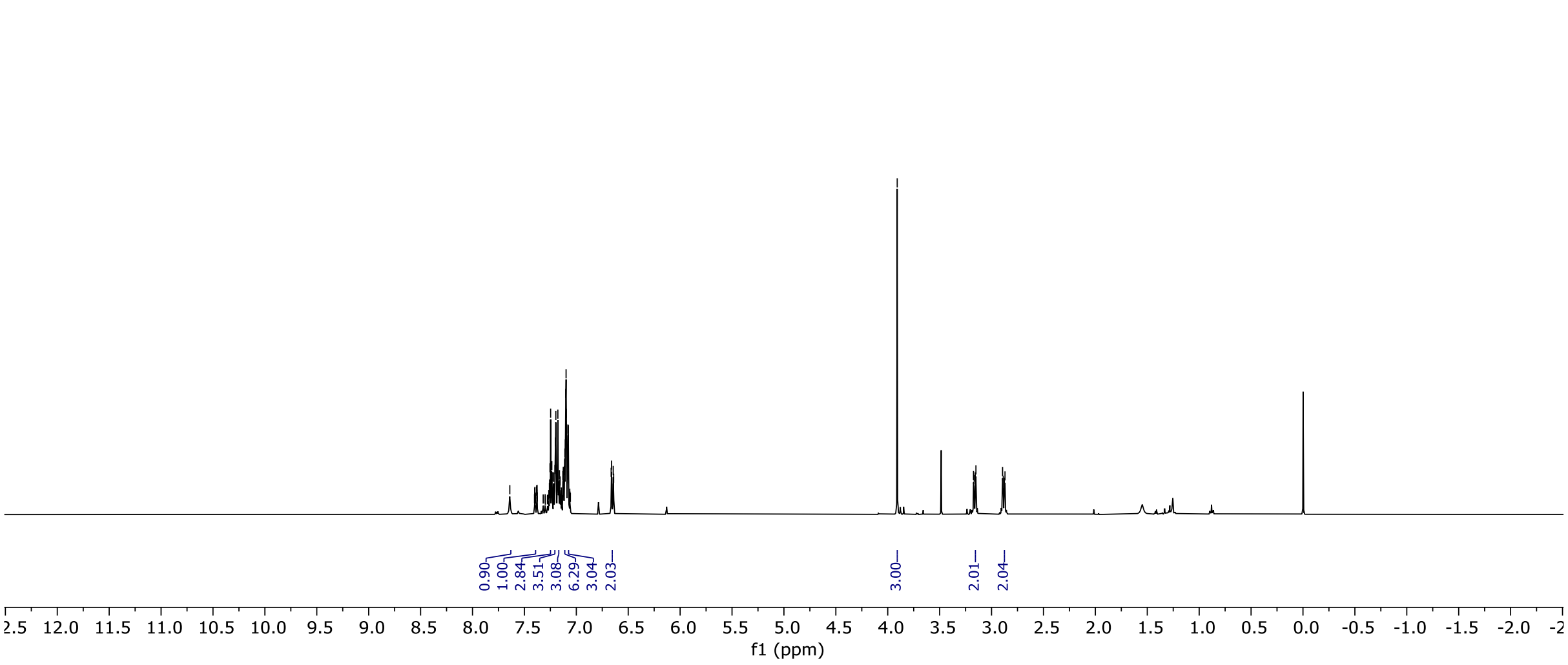

^{13}C {^{1}H} NMR (101 MHz, $CDCl_3$)

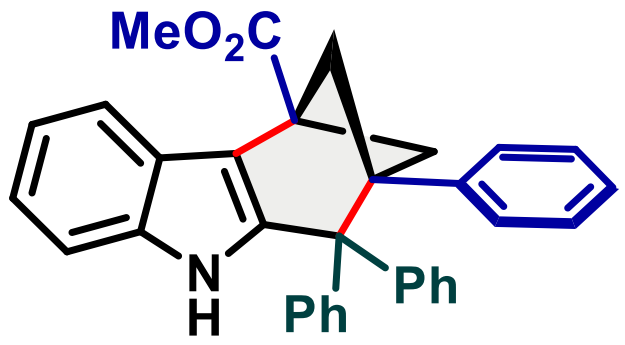

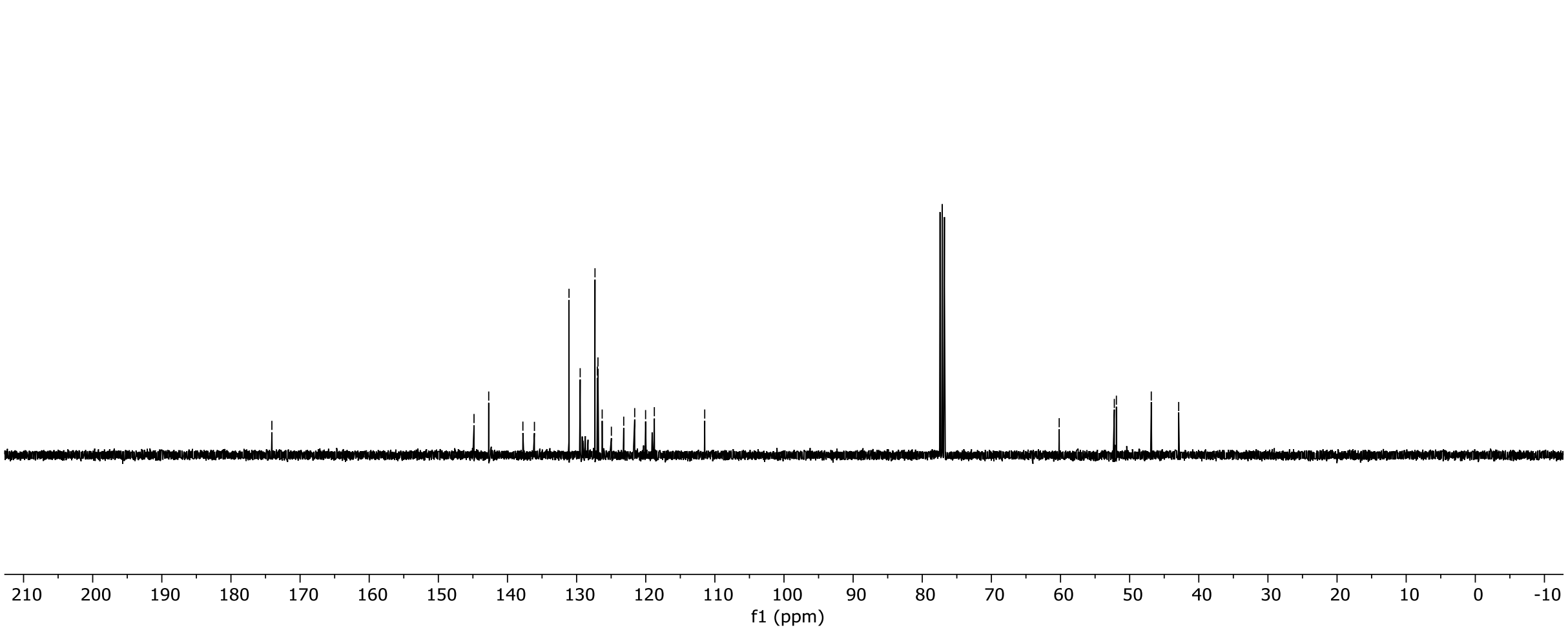

Methyl-10-methyl-9-phenyl-6-(4-(trifluoromethyl)phenyl)-6,7-dihydro-6,8-methano pyrido[1,2-a]indole-8(9*H*)-carboxylate (13):

^{1}H NMR (400 MHz, CDCl$_3$)

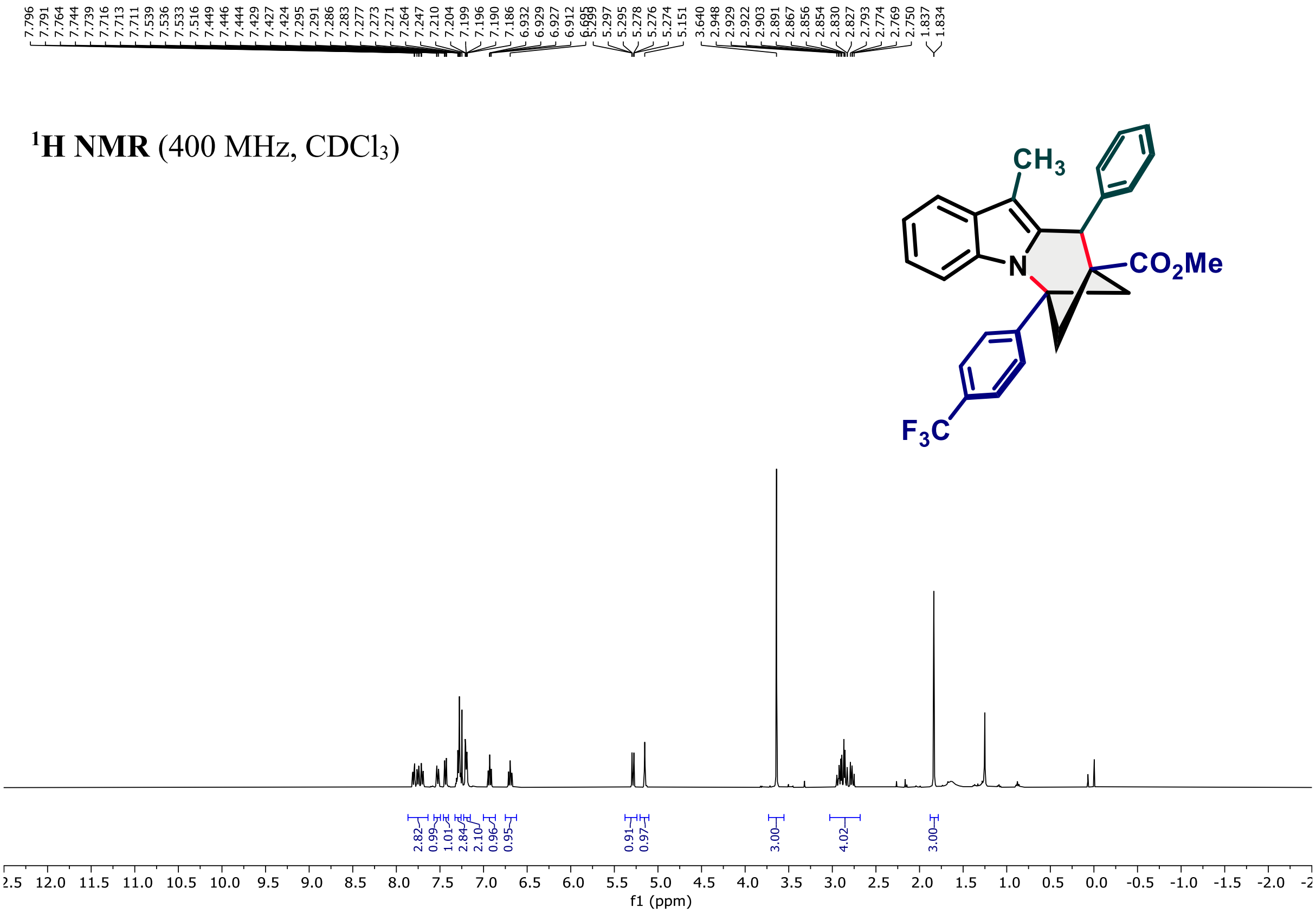

^{13}C {^{1}H} NMR (101 MHz, CDCl$_3$)

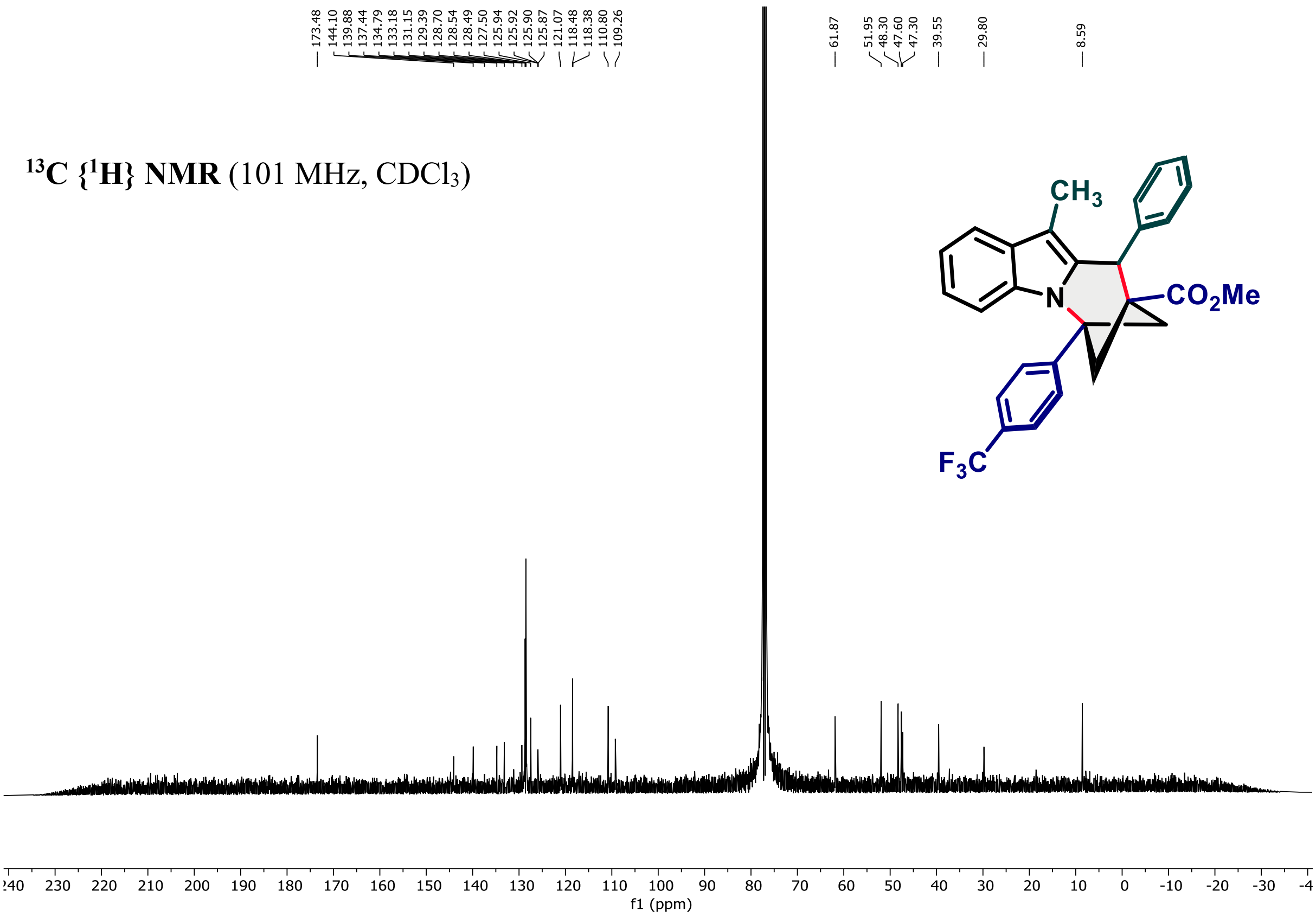

^{19}F NMR (376 MHz, $CDCl_3$)

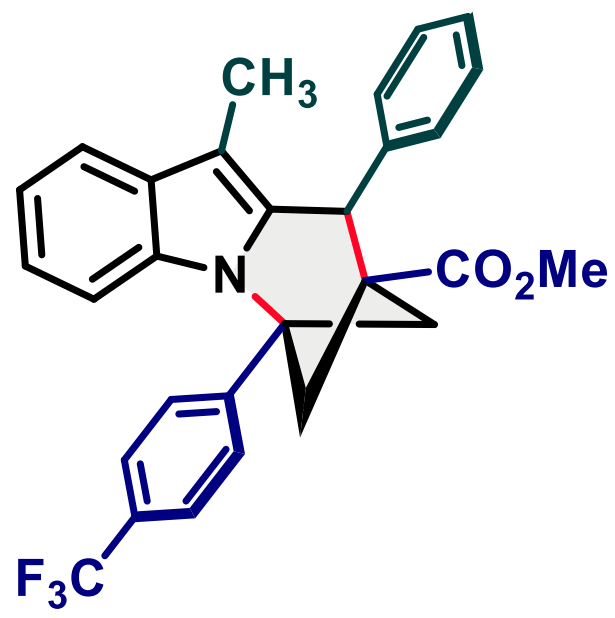

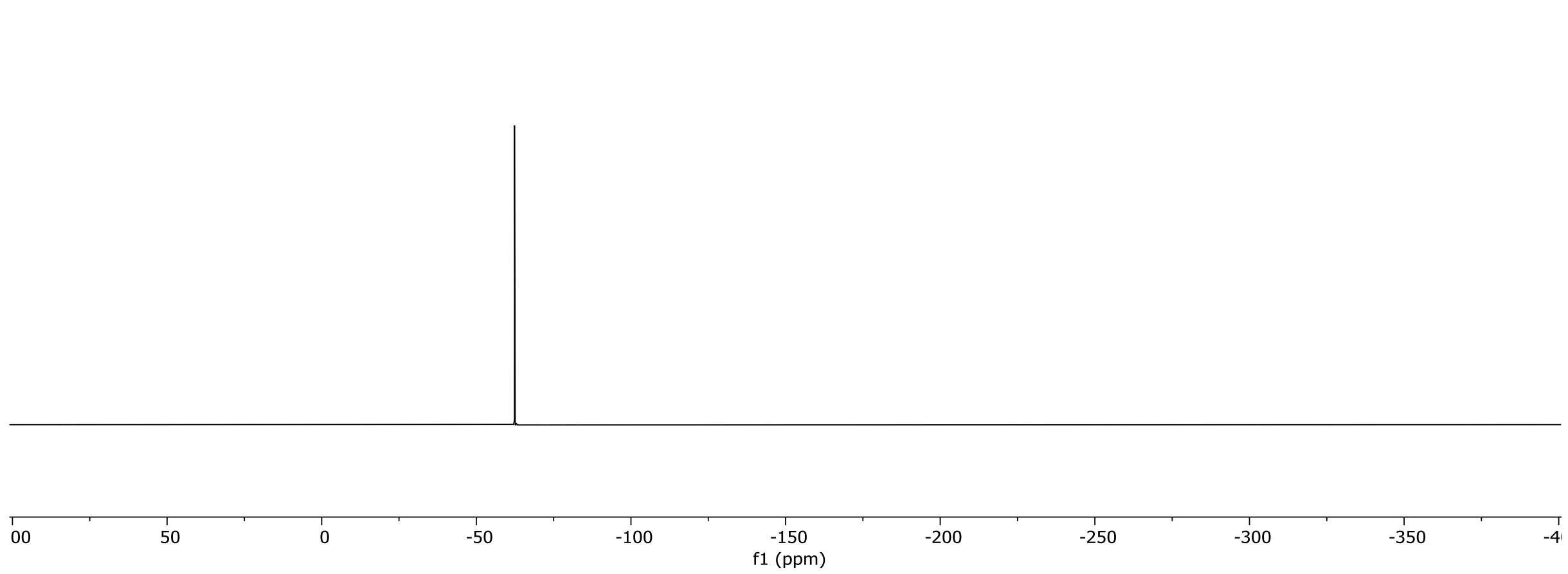

Supplement: Supplementary file 1 [file ADVS-9999-e78354-s001.pdf]
